# Taming nonclassical carbocations to control small ring reactivity

**DOI:** 10.1126/sciadv.adj9695

**Published:** 2024-01-12

**Authors:** Ryan E. McNamee, Nils Frank, Kirsten E. Christensen, Fernanda Duarte, Edward A. Anderson

**Affiliations:** Chemistry Research Laboratory, Department of Chemistry, University of Oxford, 12 Mansfield Road, Oxford OX1 3TA, UK.

## Abstract

Prediction of the outcome of ring opening of small organic rings under cationic conditions can be challenging due to the intermediacy of nonclassical carbocations. For example, the solvolysis of cyclobutyl or cyclopropylmethyl derivatives generates up to four products on nucleophilic capture or elimination via cyclopropylcarbinyl and bicyclobutonium ions. Here, we show that such reaction outcomes can be controlled by subtle changes to the structure of nonclassical carbocation. Using bicyclo[1.1.0]butanes as cation precursors, the regio- and stereochemistry of ring opening is shown to depend on the degree and nature of the substituents on the cationic intermediates. Reaction outcomes are rationalized using computational models, resulting in a flowchart to predict product formation from a given cation precursor.

## Teaser

Reactions of nonclassical carbocations can be controlled by fine-tuning the substituents on the cation precursor.

## INTRODUCTION

**Fig. 1.**
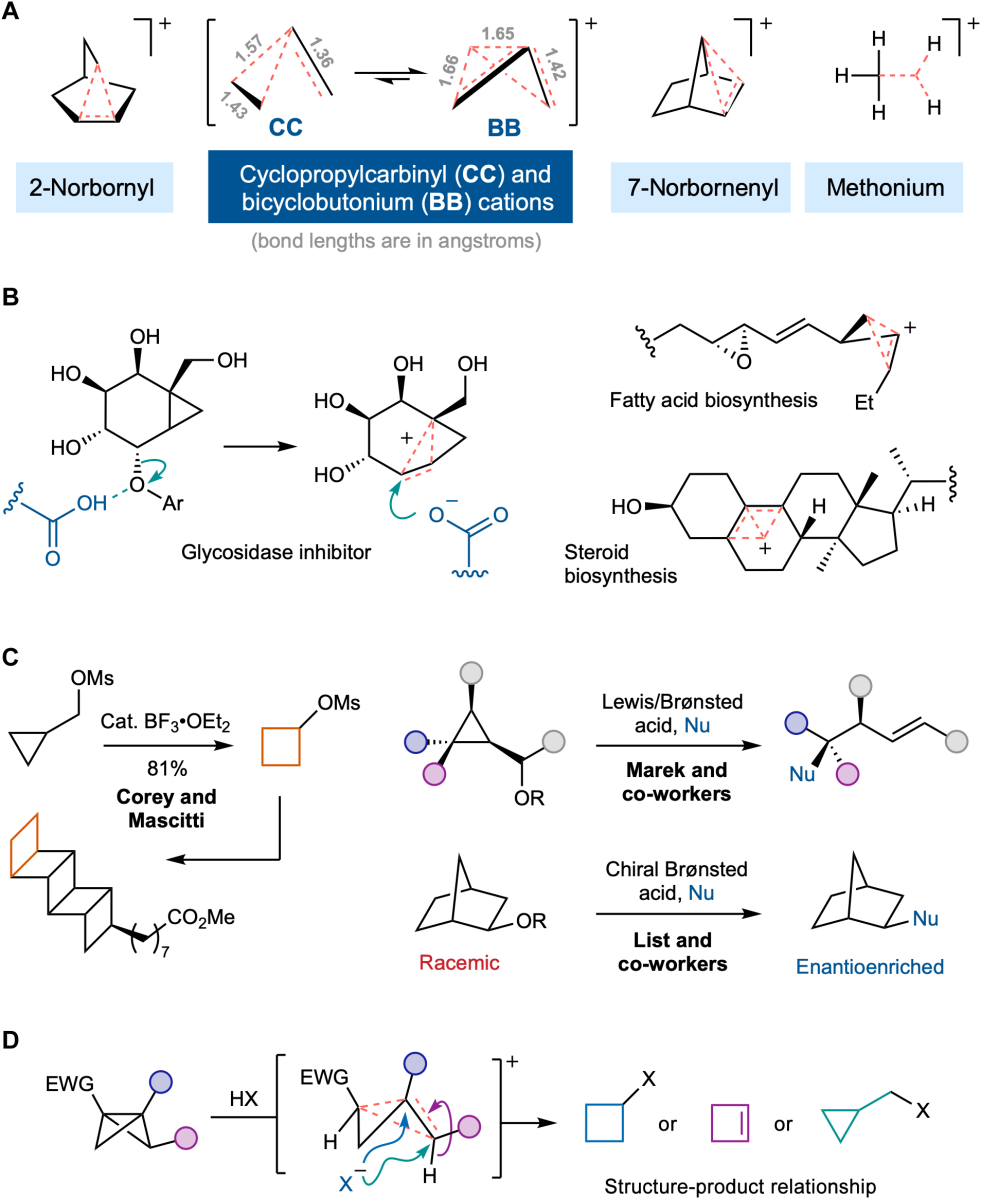
Nonclassical carbocations. (**A**) Examples of nonclassical carbocations (carbonium ions); bond lengths marked are in angstroms. (**B**) Applications of CC-BB cations as a glycosidase inhibitor and in natural product biosynthesis. (**C**) Specific product-selective reactions involving carbonium ion intermediates (*15*–*19*). (**D**) This work: formation of nonclassical carbocations from BCBs, and structure-based prediction of reaction outcomes. EWG, electron-withdrawing group.

Small organic rings are valuable building blocks in organic synthesis, as their inherent strain can facilitate reactions that would not otherwise be possible. Control over the site selectivity of reaction on small rings is typically explained by the release of ring strain or by steric effects, but this predictability can break down under cationic reaction conditions where the reaction pathway involves the formation of nonclassical carbocation intermediates (*1*–*3*). Nonclassical carbocations (Fig. 1A) have long fascinated the chemical community, not least as their characterization is highly challenging and because they have the ability to form multiple products. Among the most notable of these species are the cyclopropylcarbinyl (CC) and bicyclobutonium (BB) cations (Fig. 1A)—two closely related equilibrating structures of formula [C_4_H_7_]^+^—which form upon solvolysis of cyclobutyl and cyclopropylmethyl derivatives. The CC-BB system is the smallest of the nonclassical carbocations but has been described as “the most complex with respect to its molecular weight” (*4*). Roberts *et al.* (*5*, *6*) provided the first experimental insight into its dynamic nature in 1951, and, in later years, Olah* et al.* and Roberts* et al.* characterized its structure by low-temperature nuclear magnetic resonance (NMR) under “stable ion conditions” (*7*, *8*). Subsequent studies and computation have refined and confirmed the existence of the CC and BB species as equilibrating nonclassical ions, with the equilibrium favoring the latter by 1.9 kcal mol^−1^ (at the MP2/6-311G* level of theory). (*9*) The CC-BB system has found applications in glycosidase inhibition by aiding the generation of an aspartate trapping agent through a BB ion (*10*). CC-BB has also been proposed as an intermediate in fatty acid, steroid, and terpene biosynthesis (Fig. 1B) (*11*–*14*). In contrast, its use in chemical synthesis has been relatively restricted because of variable selectivity in product formation by nucleophilic capture or elimination: One or more of cyclopropane, cyclobutane, homoallyl, or cyclobutene products may be formed (*5*). Elegant examples of specific outcomes from systems predisposed to form one product have been described (Fig. 1C) (*15*–*21*), as well as computational explorations (*22*–*24*), but a general predictive model is yet to emerge.

In recent years, bicyclo[1.1.0]butanes (BCBs) have attracted substantial interest as versatile synthetic intermediates, due to the reactivity of the central C1─C3 bond toward nucleophiles, radicals, and electrophiles (*25*–*27*), applications as bioconjugation agents (*28*–*31*), and their ability to access bicyclo[n.1.1]alkanes that are valuable scaffolds in drug discovery (*27*, *32*). In contrast, their synthetic potential as an alternative entry point to the CC-BB cation has been largely overlooked (*33*). Here, we describe the facile generation of these cations by protonation of the BCB framework with a wide variety of Brønsted acids (Fig. 1D). We show how systematic modification of the BCB structure defines product outcome by influencing the structure and reactivity of the cationic intermediate, most notably enabling the stereoselective formation of stereochemically rich cyclopropanes. Using computed structures of these cationic intermediates, we find that product outcome can be related to subtle variations in the structure of the nonclassical intermediates (carbonium ions), or from localized equivalents (carbenium ions). Our computational (modeling) study/investigation reveals the formation of a spectrum of CC-BB intermediates and enables the delineation of factors controlling reaction outcome and small ring formation. As a result, the notoriously unpredictable behavior of the nonclassical CC-BB ion can be tamed and tuned.

## RESULTS

### Experimental results

**Fig. 2.**
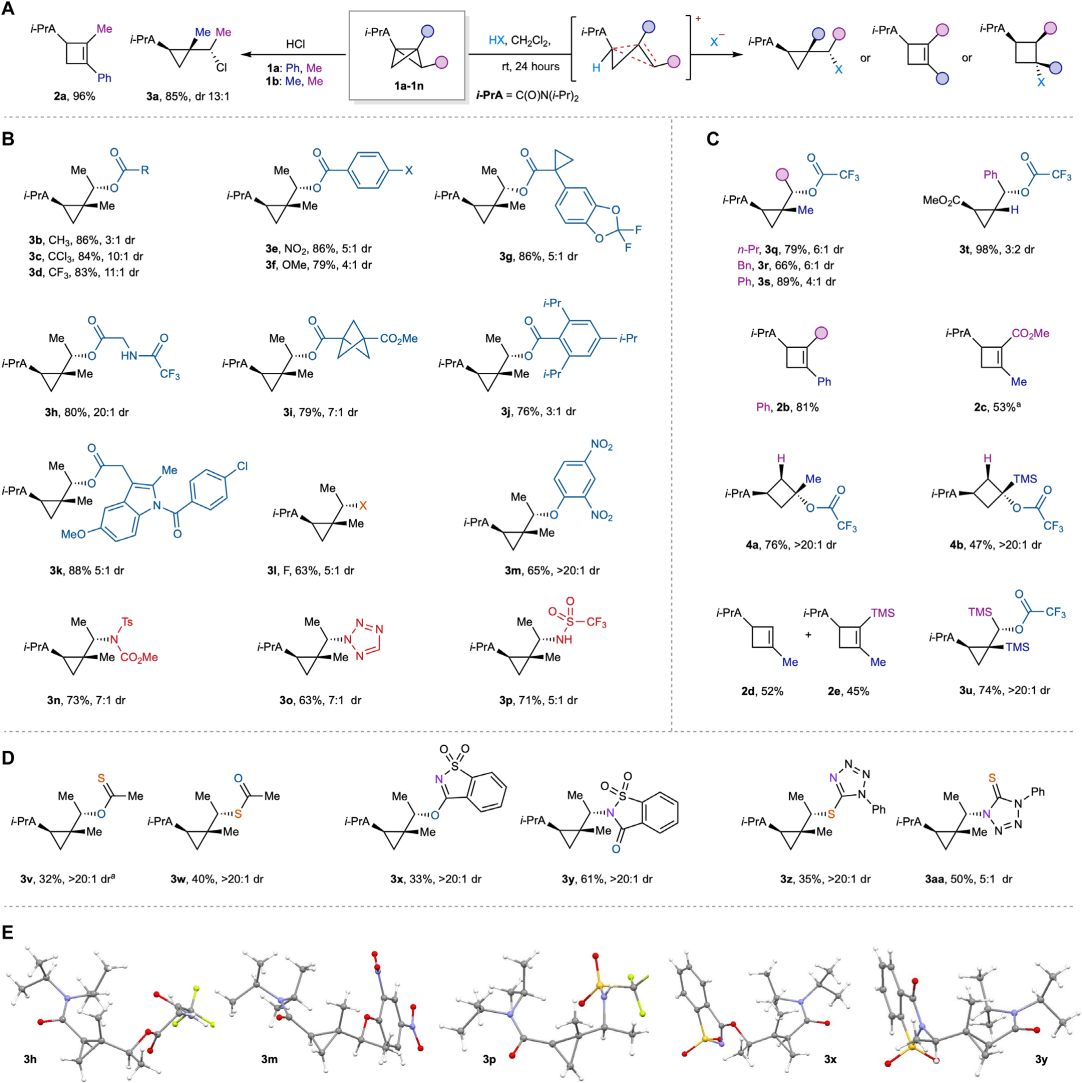
Reactions of BCBs with Brønsted acids. (**A**) Preliminary findings on BCB ring opening and generalized conditions. Reactions run on 0.2-mmol scale using 1.2 equiv. of acid in CH_2_Cl_2_ at room temperature (rt). (**B**) Reaction of BCB **1b** with Brønsted acids to give α-chiral cyclopropanes with high diastereoselectivity. (**C**) Variation of BCB substituents. (**D**) Reactions with ambident nucleophiles retain selectivity for cyclopropane formation. (**E**) Selected single-crystal x-ray structures illustrate consistent diastereoselectivity in cyclopropane formation. ^a^ Small amounts of cyclobutane product were also observed.

In preliminary work, we had observed that trisubstituted BCB **1a**, having a phenyl group at the bridgehead and a methyl group on one of its cyclopropane bridges, underwent isomerization to cyclobutene **2a** in near quantitative yield on treatment with anhydrous hydrochloric acid (Fig. 2A) (*34*, *35*). However, reaction of BCB **1b**, bearing a methyl group at both the bridge and bridgehead positions, instead exclusively afforded cyclopropane **3a** in high yield and diastereoselectivity, as confirmed by x-ray crystallographic analysis (Fig. 2E) (*34*). This selectivity led us to question whether general and predictable control over product outcome might therefore be achieved. To test this, we first explored the treatment of **1b** with a selection of Brønsted acids in dichloromethane at room temperature, which led to the highly chemo- and stereoselective formation of α-chiral cyclopropane products (Fig. 2B). A variety of aliphatic and aromatic carboxylic acids exclusively delivered cyclopropanes with ester substituents on the α-carbon atom (**3b-3k**, 76 to 88%), with diastereoselectivities up to >20:1. Reaction with hydrofluoric acid provided α-fluoro cyclopropane **3l** in good yield [63%, 5:1 diastereomeric ratio (dr)], while other acidic hydroxyl groups were also suitable, such as 2,4-dinitrophenol (**3m**, 65%). Nitrogen-based acids were also effective (**3n-3p**), providing the p*K*_a_ of the acid was less than ~5.

We next explored how the substitution pattern of the BCB (and its associated carbocation intermediate) affects the generality and outcome of this ring-opening chemistry (Fig. 2C). We found that cyclopropane-forming selectivity was maintained for BCBs featuring alkyl or aryl substituents on the bridge, with either an alkyl or hydrogen substituent at the bridgehead (**3q-3t**, 66 to 98%, up to 6:1 dr). However, a switch to cyclobutene formation was observed with either an aryl group at the bridgehead (**2b**) or an electron-withdrawing group at the bridge (**2c**). On the other hand, cyclobutane formation was observed for disubstituted BCBs featuring an alkyl or silyl substituent at the bridgehead only (**4a** and **4b**), which is consistent with ring openings of other disubstituted systems (*33*, *36*, *37*). We found that the cation-stabilizing influence of a silicon group diverted the outcome of the reaction of a bridge-silylated BCB, which afforded a mixture of silylated and non-silylated cyclobutenes **2d** and **2e**. This may suggest the intermediacy of a localized Si-stabilized cyclobutyl cation that undergoes facile elimination of the trimethylsilyl group or a proton. Intriguingly, a bridge- and bridgehead-silylated BCB afforded the cyclopropane product (**3u**), potentially indicating complementary stabilizing effects from the silicon atoms, which is consistent with the behavior of the equivalent dimethylated BCB **1b**. Last, in the case of ambident nucleophiles such as thioacetic acid, saccharin, and thiotetrazole, mixtures of O/S-, N/O-, and N/S-substituted cyclopropane adducts were observed, with both isomers being formed with high diastereoselectivity (**3v-3aa**; Fig. 2D). X-ray crystal structures of **3x**, **3y**, **3z**, and **3aa** showed that the major diastereomer was consistent across all acids, highlighting the generality of this transformation.

### Mechanistic study

**Fig. 3.**
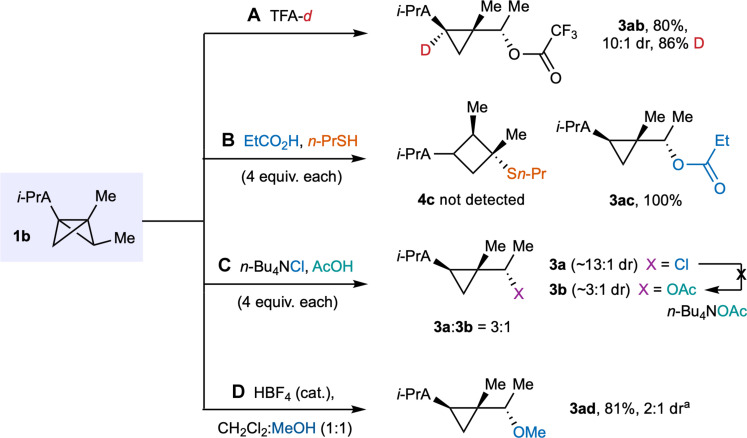
Mechanistic studies. Reaction of BCB **1b** with CF_3_CO_2_D (**A**), competition experiments (**B** and **C**), and use of a non-nucleophilic counterion to promote addition of MeOH (**D**). ^a^ Trace amounts of cyclobutane adduct observed.

To explore the mechanism of this ring-opening chemistry, we conducted a series of experimental and theoretical investigations. We found that reaction of deutero-trifluoroacetic acid with BCB **1b** afforded the corresponding bridgehead-deuterated cyclopropane **3ab** with 86% deuterium incorporation (Fig. 3A). To further investigate the chemoselectivity of cation capture, a competition reaction was run between propanoic acid and *n-*propane thiol, which afforded solely the propanoic acid adduct **3ac**, to the exclusion of the alternative cyclobutyl sulfide **4c** (Fig. 3B). This selectivity is notable, given the typical use of BCBs as thiol-selective bioconjugation agents (*28*), albeit the reaction conditions used here differ from those typically used in the latter application. An additional competition experiment using **1b** was carried out between acetic acid and tetrabutylammonium chloride (Fig. 3C), which delivered a 3:1 mixture of the α-chloro- and α-acetoxy-cyclopropanes **3a** and **3b**, respectively, both of which were formed with comparable diastereoselectivity as observed using the individual acids (i.e., HCl and AcOH; section S9). Product **3b** could not be converted to **3a** on treatment with tetrabutylammonium acetate, suggesting that both arise from competitive trapping of a common intermediate. This result prompted us to test the reaction of **1b** with an external nucleophile (methanol) using a catalytic amount of the non-nucleophilic acid promoter fluoroboric acid (Fig. 3D); this afforded methanol adduct **3ad** in 81% yield and 2:1 dr.

### Computational analysis

**Fig. 4.**
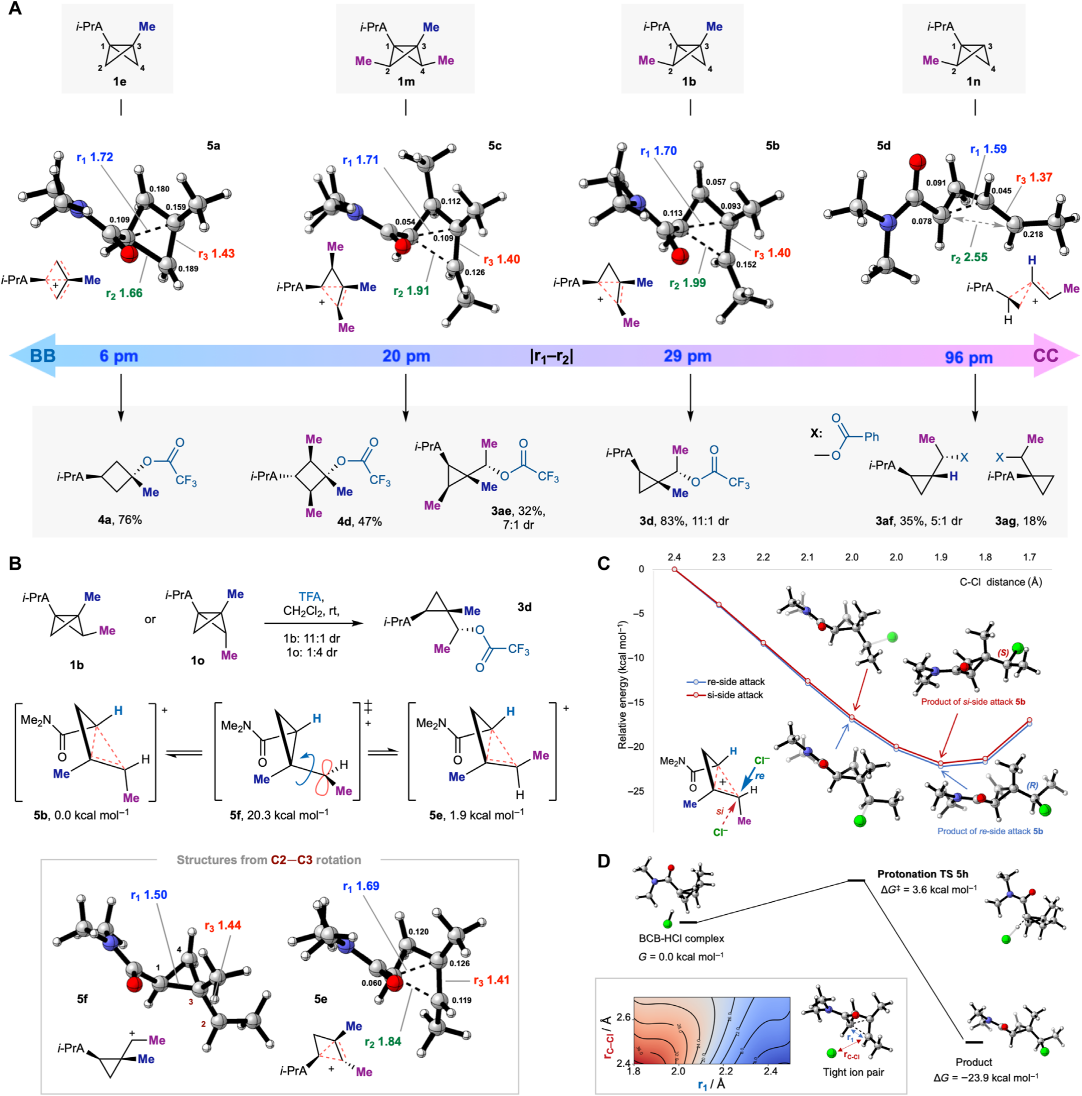
Computational analysis. (**A**) Reactions of BCBs **1b**, **1e**, and **1 m** with CF_3_CO_2_H and of **1n** with benzoic acid. Structures of the intermediate nonclassical carbanions were computed at the CPCM(DCM)-DLPNO-CCSD(T)/cc-pVQZ//CPCM(DCM)-SCS-MP2/cc-pVTZ level of theory at 298.15 K/1 M; distances are measured in angstroms, and Hirshfeld atomic charges relative to the BCB are displayed for C1─C4 (black numbers, summed for charge on specified carbon atoms plus attached hydrogen atoms). Distances r_1_ = C1─C3 (blue); r_2_ = C1─C2 (green); r_3_ = C2─C3 (red). (**B**) Inverse diastereoselectivity in the reactions of *exo*-bridge methyl BCB **1b** and *endo-*bridge methyl BCB **1o** supports the formation of non-interconverting cations. See section S17 for details of the cation **5g** that gives rise to **3ag**. (**C**) Barrierless attack of Cl^−^ on either “face” of cation **5b** [CPCM(DCM)-SCS-MP2/cc-pVTZ level of theory]. (**D**) Calculated protonation transition state (TS) using HCl [CPCM(DCM)-DLPNO-CCSD(T)/cc-pVQZ//CPCM(DCM)-SCS-MP2/cc-pVTZ level of theory, 298.15 K/1 M] with associated potential energy surface [CPCM(DCM)-SCS-MP2/cc-pVDZ level of theory].

To rationalize the product selectivity of these reactions, we performed calculations using simplified dimethylamide BCB analogs to explore how the degree and position of alkylation of the BCB scaffold affects the calculated structure of the nonclassical intermediate and, in turn, the experimental reaction outcome (Fig. 4A). To relate these calculations to our experimental findings and for clarity of discussion, the diisopropylamide BCBs used in the lab are illustrated as substrates, along with their corresponding products. Four BCBs were considered (**1b**, **1e**, **1 m**, and **1n**), which, on protonation, give rise to carbonium ions **5a-5d**. Experimentally, reaction of the bridgehead mono-methylated BCB **1e** affords solely a cyclobutane product **4a**, which computationally proceeds through the near-symmetric BB ion **5a**. The nonclassical nature of **5a** is evidenced by the Mayer bond orders (BOs) and extended bond lengths of C1─C2 (BO 0.66, 1.66 Å) and C1─C3 (BO 0.35, 1.72 Å) and partial double-bond character of C2─C3 (BO 1.02, 1.43 Å) compared to the BCB **1b** (where all C─C bond lengths are ~1.50 Å). Hirshfeld atomic charges relative to the BCB substrate indicate distribution of the positive charge over all four carbon atoms, which is also consistent with a carbonium ion. In contrast, the dimethylated BCB **1b′** affords solely the cyclopropane product **3d**, via the distorted BB ion **5b**. Comparison of these two intermediate cations reveals a lengthening of the bridgehead-bridge bond r_2_ in **5b** (1.99 Å) and increased double-bond character for r_3_ (1.40 Å) compared to **5a**. This is also reflected in the change in relative charge distribution, where a reduction in charge at C3 for **5b** compared to **5a** (0.093 versus 0.159, respectively) appears sufficient to switch the site of nucleophilic attack from cyclobutane to cyclopropane formation. Experimentally, reaction of the trimethylated BCB **1m** affords a mixture of cyclopropane **3ae** and cyclobutane **4d**, which is readily rationalized by a cationic structure **5c** intermediate between **5a** and **5b**, with a smaller difference between the partial positive charges at C2 and C3 (0.126 versus 0.109). Reaction of the regioisomeric disubstituted BCB **1n** also resulted solely in cyclopropane formation (**3af**), via a CC cation **5d** in which substantial positive charge is once again located at C2 (0.218). Interestingly, cyclopropane **3ag** was also formed as a minor product in this reaction, which arises from competing protonation at the C3 bridgehead position. The corresponding nonclassical cation **5g** (see section S17) displays increased BB character, with greatest positive charge development at the bridge carbon atom that undergoes nucleophilic attack and with only a small increase in charge at C1 relative to **5d**. Taken collectively, these four carbonium ions cover a structural continuum from BB (**5a**) to CC (**5d**), which can be characterized by subtle trends in BOs, lengths, and atomic charges. The specific structure of the cation is likely influenced by a variety of factors, including inductive effects from appended alkyl substituents, conjugation (with aryl groups), and hyperconjugation (from antiperiplanar vicinal C─H bonds) (*38*). Our attempts to compute hyperconjugation contributions via NBO analysis were highly basis set dependent; however, a close examination of Hirshfeld charges on the computed cations (section S16) suggests that hyperconjugation could be a contributory factor to cation stabilization, as hydrogen atoms of C─H bonds that are antiperiplanar to partial C─C bonds carry a greater charge compared to others on the same carbon atom.

As noted above, the x-ray crystallographic structures had revealed an intriguing finding: The identity of the major diastereomer from cyclopropane-forming BCB ring opening consistently appears to arise from attack on the nonclassical carbocation from the same “face” as protonation. This outcome could derive from the formation of a tight ion pair upon BCB protonation, such that the counterion is necessarily located in proximity to the cation **5b** and on the same face. This hypothesis is supported by the reaction of the bridge-substituted *endo*-methyl BCB **1o** (Fig. 4B) with trifluoroacetic acid, which afforded product **2e** with 4:1 dr, where the major diastereomer is the opposite to that derived from *exo*-methyl BCB **1b**. This reinforces the potential formation of single diastereomeric nonclassical carbocations from each diastereomer of BCB, each of which reacts with discrete stereoselectivity. In the case of epimeric BCB **1o**, the corresponding nonclassical cation **5e** was found to be 1.9 kcal mol^−1^ higher in free energy than **5b** and displays a shorter C1─C2 bond distance (1.84 Å in **5e** versus 1.99 Å in **5b**), which may well relate to the reduced diastereoselectivity observed in cyclopropane formation, and we therefore questioned whether diastereoselectivity could be affected by interconversion of cations **5b** and **5e**, via a localized “classical” cationic species. However, this transition state **5f** was found to be substantially higher in free energy (by 20.3 kcal mol^−1^) than **5b**, which appears to prohibit interconversion of cations **5b** and **5e**. Further calculations using dichloromethane as implicit solvent, starting from a constrained C─Cl distance of 2.4 Å (Fig. 4C), revealed that attack (by chloride) on either face of intermediate **5b** is barrierless, which would be consistent with a typical protonation/capture event. A general acid protonation transition state **5h** was calculated (Fig. 4D, Δ*G*^‡^ = 3.6 kcal mol^−1^), after which a downhill two-dimensional scan indicates that this tight ion pair immediately collapses into the observed major diastereomer (Δ*G*^‡^ = −23.9 kcal mol^−1^).

Although the diastereoselectivity of addition reactions to **2b** proved insensitive to factors such as solvent, temperature, concentration, and equivalents of acid (see section S3), we also noted a correlation between p*K*_a_ of the carboxylic acid used and diastereomeric excess, where stronger acids resulted in higher selectivity (see Fig. 2B and section S11). The influence of a noncoordinating tetrafluoroborate counterion on the structure of CC and BB cations has been calculated by Larmore and Champagne (*22*); their findings highlight the possibility that different counterions could also have an impact on the stereoselectivity of nucleophilic additions, which may explain the consistent stereoselectivity observed in the individual and competition experiments using acetate and chloride nucleophiles (see Figs. 2 and 3A).

### Linear free energy relationship

**Fig. 5.**
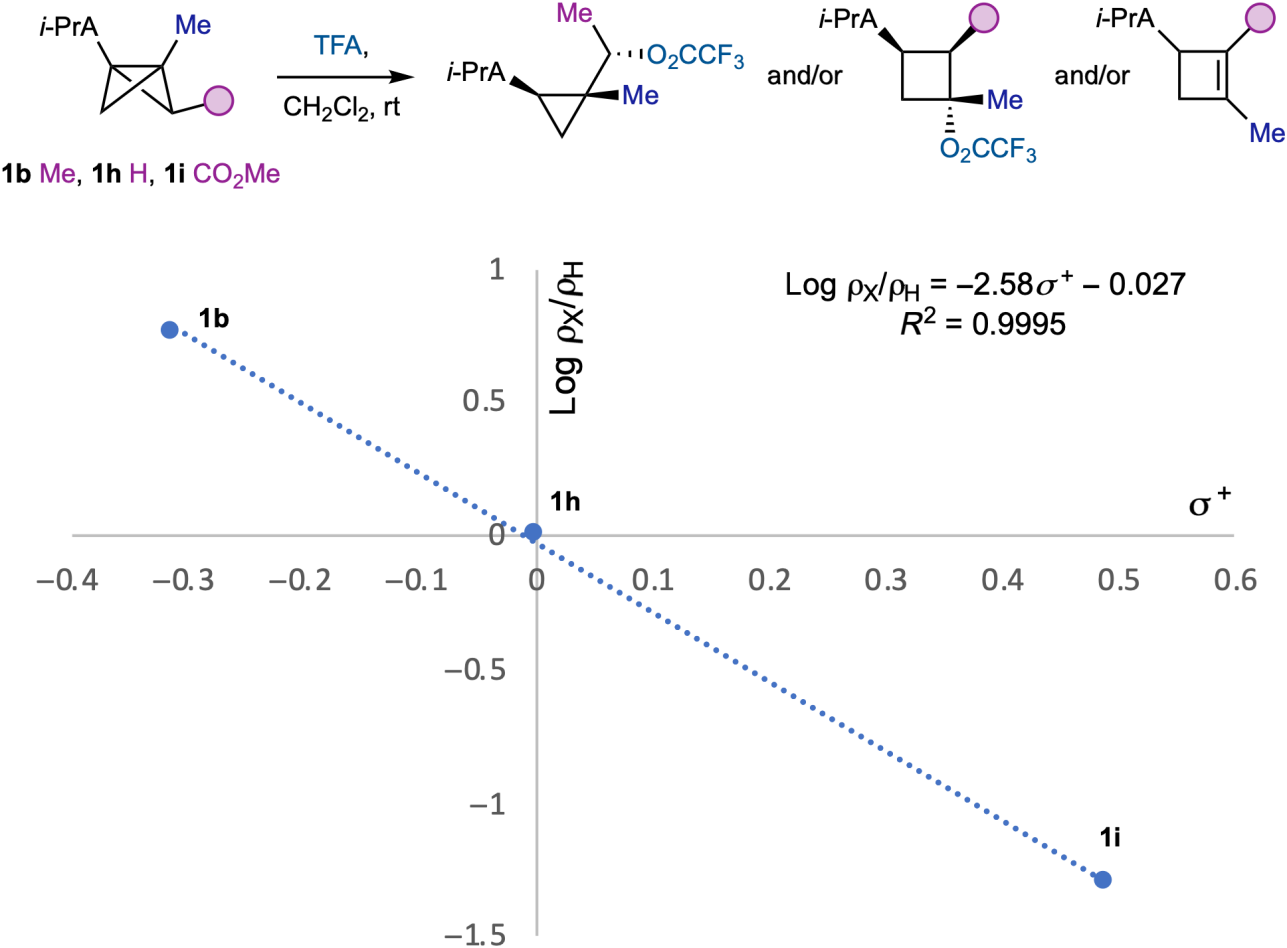
Hammett plot obtained from competition experiments in the reaction of bridge-substituted BCBs 1b, 1h, and 1i.

Having identified how a continuum of nonclassical carbocation intermediates can affect product selectivity, we considered the influence of the BCB substituents on relative reaction rates, due to their effect on developing positive charge during the protonation step. A competition study was carried out between BCB **1h** (Fig. 5) and BCBs bearing electron-donating (**1b**) and electron-withdrawing groups (**1h**), with trifluoroacetic acid under the standard reaction conditions. **1b** was found to react more rapidly than **1h**, which, in turn, outcompeted **1h**. Using σ^+^ substituent parameters, a Hammett plot was constructed, which gave a linear relationship with a ρ^+^ value of −2.6, indicating substantial positive charge buildup on the BCB bridge atom (*39*, *40*). Compared to the ρ^+^ value of −12.3 measured by Oyama and Tidwell (*41*) for formation of a CC cation by vinylcyclopropane protonation, and a value of −7.1 observed by Hoz and co-workers (*42*) for acid-catalyzed hydration of bridgehead-disubstituted BCBs, our findings support a greater delocalization of positive charge in the developing nonclassical cation, with the C3 bridgehead position being the most influential on reaction outcome.

## DISCUSSION

**Fig. 6.**
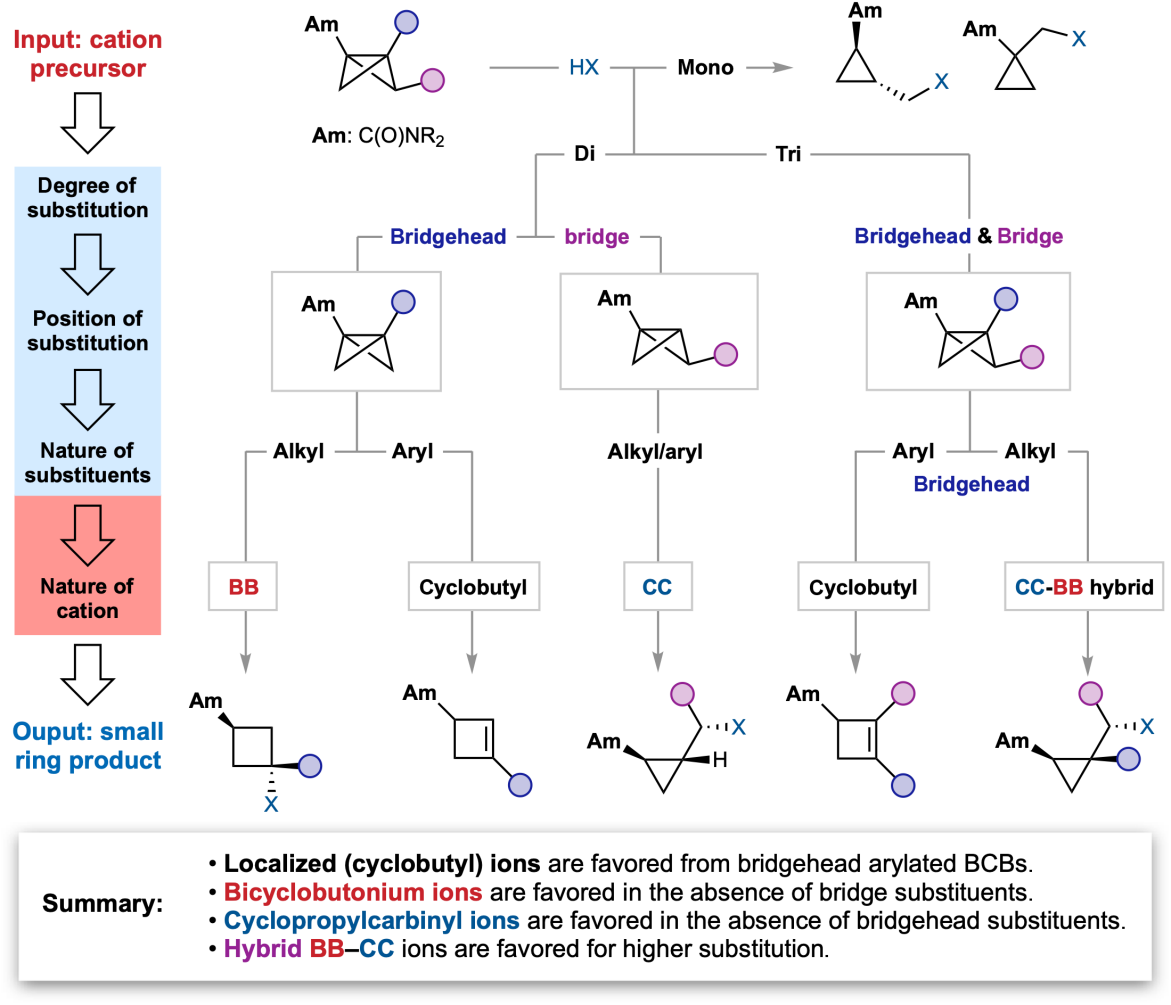
Flowchart to predict product outcome based on the degree, position, and nature of substituents on the BCB carbocation precursor.

Our experimental findings and those from previous studies (*43*) enable the rational prediction of reaction outcome for the acid-promoted formation and capture of the nonclassical BB/CC systems. A flowchart of BCB structure versus product outcome (Fig. 6) reflects the different extents of charge distribution and structure that can be expected on the basis of the degree and nature of substitution on the cationic intermediate. Monosubstituted BCBs exhibit limited selectivity due to the minimal influence of the substituents on the cation, with cyclopropane and/or cyclobutane products observed (see section S12). However, predictable outcomes are found for disubstituted intermediates, which give complementary outputs depending on the disposition of substituents: For bridgehead substitution, cyclobutenes are observed for aryl substituents (via localized cyclobutyl cations), but cyclobutanes arise from alkyl substitution (via BB ions), while, for bridge-functionalized BCBs, cyclopropanes arise irrespective of the nature of the substituent (via CC ions or hybrid BB-CC structures). The outcome for trisubstituted ions is also substituent dependent: Bridgehead aryl groups once again direct the reaction toward cyclobutene products, but all other alkyl/aryl combinations afford cyclopropanes.

Limitations of this model include the presence of an electron-withdrawing group on one of the BCB bridgehead atoms in all cases studied. This group is now necessitated by the directed metalation strategy used to synthesize the polysubstituted BCB substrates and also in conferring stability on the BCB framework against gradual degradation on storage. While offering benefits in terms of the regioselectivity of protonation, it also restricts the structural diversity of the resulting products. A study of the role of the electron-withdrawing group itself would be a useful direction for further study to elucidate the generality of the predictive flow chart; initial studies with monosubstituted BCBs suggest that broadly similar selectivity trends may be expected. The basis of the diastereoselectivity of cation trapping remains to be fully determined, which will likely require extensive computational and experimental studies; toward this end, a more advanced computational model, including simulation of explicit solvent and dynamic nature of the system, is under development in our research groups. Understanding of these factors should open up wider opportunities to tame and tune the reactivity of these elusive but fascinating cationic intermediates.

## MATERIALS AND METHODS

### Experimental design

The objective of the study was to explore the outcome of the reactions of polysubstituted BCBs with Brønsted acids, in terms of the structure of the reaction product, the stereoselectivity and regioselectivity (where relevant), and the factors affecting these selectivities. All BCBs feature an electron-withdrawing substituent at one of the bridgehead positions, which stabilizes the BCB relative to undesired degradation via ring opening. All reactions of BCBs were conducted at room temperature and assessed by tlc, as well as ^1^H NMR spectroscopic analysis of the crude reaction mixture, the latter enabling accurate determination of product composition where more than one product was formed.

### General procedure for addition of Brønsted acids to BCBs

To a solution of BCB (0.20 mmol, 1.0 equiv.) in CH_2_Cl_2_ (1 ml) at room temperature was added the respective Brønsted acid (0.24 mmol, 1.2 equiv.). The reaction was stirred for 24 hours at room temperature before concentrating in vacuo. The residue was purified via flash silica chromatography with Et_2_O/pentane or EtOAc/pentane as eluent.

## Supplementary Materials

### This PDF file includes:

Sections S1 to S21 Figs. S1 to S22 Tables S1 to S13 References

## References

[1] H. C. Brown, *The Nonclassical Ion Problem* (Springer, 2012).

[2] G. A. Olah, 100 years of carbocations and their significance in chemistry. J. Org. Chem. 66, 5943–5957 (2001). 11529717 10.1021/jo010438x

[3] H.-U. Siehl, in *Advances in Physical Organic Chemistry*, I. H. Williams, N. H. Williams, Eds. (Academic Press, 2018), vol. 52, pp. 1–47.

[4] P. D. Bartlett, *Nonclassical Ions* (W. A. Benjamin, 1965).

[5] J. D. Roberts, R. H. Mazur, I. V. Small-Ring Compounds, Small-ring compounds. IV. Interconversion reactions of cyclobutyl, cyclopropylcarbinyl and allylcarbinyl derivatives. J. Am. Chem. Soc. 73, 2509–2520 (1951).

[6] J. D. Roberts, R. H. Mazur, The nature of the intermediate in carbonium ion-type interconversion reactions of cyclobutyl, cyclopropylcarbinyl and allylcarbinyl derivatives. J. Am. Chem. Soc. 73, 3542–3543 (1951).

[7] G. A. Olah, C. L. Jeuell, D. P. Kelly, R. D. Porter, Stable carbocations. CXIV. Structure of cyclopropylcarbinyl and cyclobutyl cations. J. Am. Chem. Soc. 94, 146–156 (1972).

[8] J. S. Staral, I. Yavari, J. D. Roberts, G. K. S. Prakash, D. J. Donovan, G. A. Olah, Low-temperature carbon-13 nuclear magnetic resonance spectroscopic investigation of C4H7+. Evidence for an equilibrium involving the nonclassical bicyclobutonium ion and the bisected cyclopropylcarbinyl cation. J. Am. Chem. Soc. 100, 8016–8018 (1978).

[9] G. A. Olah, G. K. S. Prakash, G. Rasul, Ab initio/GIAO-CCSD(T) study of structures, energies, and ^13^C NMR chemical shifts of C_4_H_7_^+^ and C_5_H_9_^+^ ions: Relative stability and dynamic aspects of the cyclopropylcarbinyl vs bicyclobutonium ions. J. Am. Chem. Soc. 130, 9168–9172 (2008). 18570420 10.1021/ja802445s

[10] S. Chakladar, Y. Wang, T. Clark, L. Cheng, S. Ko, D. J. Vocadlo, A. J. Bennet, A mechanism-based inactivator of glycoside hydrolases involving formation of a transient non-classical carbocation. Nat. Commun. 5, 5590 (2014). 25475952 10.1038/ncomms6590

[11] Y. J. Hong, J.-L. Giner, D. J. Tantillo, Bicyclobutonium ions in biosynthesis – Interconversion of cyclopropyl-containing sterols from orchids. J. Am. Chem. Soc. 137, 2085–2088 (2015). 25607948 10.1021/ja512901a

[12] Y. J. Hong, D. J. Tantillo, The energetic viability of an unexpected skeletal rearrangement in cyclooctatin biosynthesis. Org. Biomol. Chem. 13, 10273–10278 (2015). 26371548 10.1039/c5ob01785h

[13] D. P. Kranz, S. Chiha, A. Meier zu Greffen, J.-M. Neudörfl, H.-G. Schmalz, Synthesis of B-ring-modified steroids through BF3-promoted rearrangement/substitution of 6β-hydroxy-5,19-cyclosteroids. Org. Lett. 14, 3692–3695 (2012). 22765256 10.1021/ol301532w

[14] L. A. Wessjohann, W. Brandt, T. Thiemann, Biosynthesis and metabolism of cyclopropane rings in natural compounds. Chem. Rev. 103, 1625–1648 (2003). 12683792 10.1021/cr0100188

[15] V. Mascitti, E. J. Corey, Enantioselective synthesis of pentacycloanammoxic acid. J. Am. Chem. Soc. 128, 3118–3119 (2006). 16522072 10.1021/ja058370g

[16] V. Lanke, I. Marek, Nucleophilic substitution at quaternary carbon stereocenters. J. Am. Chem. Soc. 142, 5543–5548 (2020). 32141750 10.1021/jacs.0c01133

[17] X. Chen, K. Patel, I. Marek, Stereoselective construction of tertiary homoallyl alcohols and ethers by nucleophilic substitution at quaternary carbon stereocenters. Angew. Chem. Int. Ed. 62, e202212425 (2023). 10.1002/anie.202212425 PMC10107121 36413111

[18] X. Chen, K. Patel, I. Marek, Stereospecific nucleophilic substitution at quaternary carbon stereocenters of cyclopropyl carbinols. Chem 9, 266–279 (2023).

[19] R. Properzi, P. S. J. Kaib, M. Leutzsch, G. Pupo, R. Mitra, C. Kanta De, L. Song, P. R. Schreiner, B. List, Catalytic enantiocontrol over a non-classical carbocation. Nat. Chem. 12, 1174–1179 (2020). 32989271 10.1038/s41557-020-00558-1

[20] A. Bauer, G. Di Mauro, J. Li, N. Maulide, An α-cyclopropanation of carbonyl derivatives by oxidative umpolung. Angew. Chem. Int. Ed. 59, 18208–18212 (2020). 10.1002/anie.202007439 PMC7589340 32808419

[21] J. Xie, G. Dong, Cyclopropylcarbinyl cation chemistry in synthetic method development and natural product synthesis: Cyclopropane formation and skeletal rearrangement. Org. Chem. Front. 10, 2346–2358 (2023).

[22] S. P. Larmore, P. A. Champagne, Cyclopropylcarbinyl-to-homoallyl carbocation equilibria influence the stereospecificity in the nucleophilic substitution of cyclopropylcarbinols. J. Org. Chem. 88, 6947–6954 (2023). 37141426 10.1021/acs.joc.3c00257

[23] S. S. Goh, P. A. Champagne, S. Guduguntla, T. Kikuchi, M. Fujita, K. N. Houk, B. L. Feringa, Stereospecific ring contraction of bromocycloheptenes through dyotropic rearrangements via nonclassical carbocation–anion pairs. J. Am. Chem. Soc. 140, 4986–4990 (2018). 29596748 10.1021/jacs.8b00821 PMC5909176

[24] H. Sato, B.-X. Li, T. Takagi, C. Wang, K. Miyamoto, M. Uchiyama, DFT study on the biosynthesis of verrucosane diterpenoids and mangicol sesterterpenoids: Involvement of secondary-carbocation-free reaction cascades. JACS Au 1, 1231–1239 (2021). 34467361 10.1021/jacsau.1c00178 PMC8397367

[25] C. B. Kelly, J. A. Milligan, L. J. Tilley, T. M. Sodano, Bicyclobutanes: From curiosities to versatile reagents and covalent warheads. Chem. Sci. 13, 11721–11737 (2022). 36320907 10.1039/d2sc03948f PMC9580472

[26] M. A. A. Walczak, T. Krainz, P. Wipf, Ring-strain-enabled reaction discovery: New heterocycles from bicyclo[1.1.0]butanes. Acc. Chem. Res. 48, 1149–1158 (2015). 25775119 10.1021/ar500437h

[27] M. Golfmann, J. C. L. Walker, Bicyclobutanes as unusual building blocks for complexity generation in organic synthesis. Commun. Chem. 6, 9 (2023). 36697911 10.1038/s42004-022-00811-3 PMC9837078

[28] K. Tokunaga, M. Sato, K. Kuwata, C. Miura, H. Fuchida, N. Matsunaga, S. Koyanagi, S. Ohdo, N. Shindo, A. Ojida, Bicyclobutane carboxylic amide as a cysteine-directed strained electrophile for selective targeting of proteins. J. Am. Chem. Soc. 142, 18522–18531 (2020). 33047956 10.1021/jacs.0c07490

[29] B. D. Schwartz, A. P. Smyth, P. E. Nashar, M. G. Gardiner, L. R. Malins, Investigating bicyclobutane–triazolinedione cycloadditions as a tool for peptide modification. Org. Lett. 24, 1268–1273 (2022). 35014844 10.1021/acs.orglett.1c04071

[30] J. M. Lopchuk, K. Fjelbye, Y. Kawamata, L. R. Malins, C.-M. Pan, R. Gianatassio, J. Wang, L. Prieto, J. Bradow, T. A. Brandt, M. R. Collins, J. Elleraas, J. Ewanicki, W. Farrell, O. O. Fadeyi, G. M. Gallego, J. J. Mousseau, R. Oliver, N. W. Sach, J. K. Smith, J. E. Spangler, H. Zhu, J. Zhu, P. S. Baran, Strain-release heteroatom functionalization: Development, scope, and stereospecificity. J. Am. Chem. Soc. 139, 3209–3226 (2017). 28140573 10.1021/jacs.6b13229 PMC5334783

[31] P. Zhang, R. Zhuang, X. Wang, H. Liu, J. Li, X. Su, X. Chen, X. Zhang, Highly efficient and stable strain-release radioiodination for thiol chemoselective bioconjugation. Bioconjug. Chem. 29, 467–472 (2018). 29376327 10.1021/acs.bioconjchem.7b00790

[32] P. K. Mykhailiuk, Saturated bioisosteres of benzene: Where to go next? Org. Biomol. Chem. 17, 2839–2849 (2019). 30672560 10.1039/c8ob02812e

[33] K. Livingstone, K. Siebold, S. Meyer, V. Martín-Heras, C. G. Daniliuc, R. Gilmour, Skeletal ring contractions via I(I)/I(III) catalysis: Stereoselective synthesis of cis-α,α-difluorocyclopropanes. ACS Catal. 12, 14507–14516 (2022). 36504915 10.1021/acscatal.2c04511 PMC9724094

[34] R. E. McNamee, A. L. Thompson, E. A. Anderson, Synthesis and applications of polysubstituted bicyclo[1.1.0]butanes. J. Am. Chem. Soc. 143, 21246–21251 (2021). 34904841 10.1021/jacs.1c11244

[35] R. E. McNamee, M. M. Haugland, J. Nugent, R. Chan, K. E. Christensen, E. A. Anderson, Synthesis of 1,3-disubstituted bicyclo[1.1.0]butanes via directed bridgehead functionalization. Chem. Sci. 12, 7480–7485 (2021). 34163838 10.1039/d1sc01836a PMC8171340

[36] K. B. Wiberg, G. M. Lampman, R. P. Ciula, D. S. Connor, P. Schertler, J. Lavanish, Bicyclo[1.1.0]butane. Tetrahedron 21, 2749–2769 (1965).

[37] L. Guo, A. Noble, V. K. Aggarwal, α-Selective ring-opening reactions of bicyclo[1.1.0]butyl boronic ester with nucleophiles. Angew. Chem. Int. Ed. 60, 212–216 (2021). 10.1002/anie.202011739 32956541

[38] Y. J. Hong, D. J. Tantillo, C–H⋯π interactions as modulators of carbocation structure – Implications for terpene biosynthesis. Chem. Sci. 4, 2512–2518 (2013).

[39] T. Shono, A. Oku, R. Oda, Small ring compounds—XVI: Solvolysis of α(p-substitutedphenyl)cycloprophylcarbinyl p-nitrobenzoate. Tetrahedron 24, 421–425 (1968).

[40] E. N. Peters, H. C. Brown, Solvolysis of 1-aryl-1-cyclopropyl-1-ethyl p-nitrobenzoates. Evidence for major increases in electron supply by the cyclopropyl group with increasing electron demand at the cationic center. J. Am. Chem. Soc. 95, 2397–2398 (1973).

[41] K. Oyama, T. T. Tidwell, Cyclopropyl substituent effects on acid-catalyzed hydration of alkenes. Correlation by .sigma.+ parameters. J. Am. Chem. Soc. 98, 947–951 (1976).

[42] S. Hoz, M. Livneh, D. Cohen, Cyclobutane-bicyclobutane system. 11. Mechanism and stereochemistry of general acid-catalyzed additions to bicyclobutane. J. Org. Chem. 51, 4537–4544 (1986).

[43] G. A. Olah, V. P. Reddy, G. K. S. Prakash, Long-lived cyclopropylcarbinyl cations. Chem. Rev. 92, 69–95 (1992).

[44] R. I. Cooper, A. L. Thompson, D. J. Watkin, CRYSTALS enhancements: Dealing with hydrogen atoms in refinement. J. Appl. Cryst. 43, 1100–1107 (2010).

[45] P. Parois, R. I. Cooper, A. L. Thompson, Crystal structures of increasingly large molecules: Meeting the challenges with CRYSTALS software. Chem. Cent. J. 9, 30 (2015). 26029252 10.1186/s13065-015-0105-4 PMC4448882

[46] E. Vedejs, J. Cabaj, M. J. Peterson, Wittig ethylidenation of ketones: Reagent control of *Z*/*E* selectivity. J. Org. Chem. 58, 6509–6512 (1993).

[47] C. Hansch, A. Leo, R. W. Taft, A survey of Hammett substituent constants and resonance and field parameters. Chem. Rev. 91, 165–195 (1991).

[48] F. Neese, The ORCA program system. WIREs Comput. Mol. Sci. 2, 73–78 (2012).

[49] F. Neese, Software update: The ORCA program system, version 4.0. WIREs Comput. Mol. Sci. 8, e1327 (2017).

[50] S. Grimme, Improved second-order Møller–Plesset perturbation theory by separate scaling of parallel- and antiparallel-spin pair correlation energies. J. Chem. Phys. 118, 9095–9102 (2003).

[51] R. F. Fink, Spin-component-scaled Møller–Plesset (SCS-MP) perturbation theory: A generalization of the MP approach with improved properties. J. Chem. Phys. 133, 174113 (2010). 21054012 10.1063/1.3503041

[52] F. Neese, F. Wennmohs, A. Hansen, U. Becker, Efficient, approximate and parallel Hartree–Fock and hybrid DFT calculations. A ‘chain-of-spheres’ algorithm for the Hartree–Fock exchange. Chem. Phys. 356, 98–109 (2009).

[53] T. H. Dunning Jr., Gaussian basis sets for use in correlated molecular calculations. I. The atoms boron through neon and hydrogen. J. Chem. Phys. 90, 1007–1023 (1989).

[54] D. G. Liakos, M. Sparta, M. K. Kesharwani, J. M. Martin, F. Neese, Exploring the accuracy limits of local pair natural orbital coupled-cluster theory. J. Chem. Theory Comput. 11, 1525–1539 (2015). 26889511 10.1021/ct501129s

[55] S. Grimme, Supramolecular binding thermodynamics by dispersion-corrected density functional theory. Chem. A Eur. J. 18, 9955–9964 (2012). 10.1002/chem.201200497 22782805

[56] T. Young, https://github.com/duartegroup/otherm (2020).

[57] I. Mayer, Charge, bond order and valence in the AB initio SCF theory. Chem. Phys. Lett. 97, 270–274 (1983).

[58] A. J. Bridgeman, G. Cavigliasso, L. R. Ireland, J. Rothery, The Mayer bond order as a tool in inorganic chemistry. J. Chem. Soc. 14, 2095–2108 (2001).

[59] D. H. Aue, Carbocations. WIREs Comput. Mol. Sci. 1, 487–508 (2011).

[60] I. Alkorta, J. L. M. Abboud, E. Quintanilla, J. Z. Dávalos, A theoretical study of (old and new) non-classical carbocations derived from cyclic saturated hydrocarbons. J. Phys. Org. Chem. 16, 546–554 (2003).

[61] F. Weigend, R. Ahlrichs, Balanced basis sets of split valence, triple zeta valence and quadruple zeta valence quality for H to Rn: Design and assessment of accuracy. Phys. Chem. Chem. Phys. 7, 3297–3305 (2005). 16240044 10.1039/b508541a

[62] H.-U. Siehl, M. Fuss, J. Gauss, The 1-(trimethylsilyl) bicyclobutonium Ion: NMR spectroscopy, isotope effects, and quantum chemical ab initio calculations of a new hypercoordinated carbocation. J. Am. Chem. Soc. 117, 5983–5991 (1995).

[63] F. L. Hirshfeld, Bonded-atom fragments for describing molecular charge densities. Theor. Chim. Acta. 44, 129–138 (1977).

