# Supplementary material for: Taming nonclassical carbocations to control small ring reactivity
Source: Sci Adv. 2024 Jan 12;10(2):eadj9695. doi: 10.1126/sciadv.adj9695 (PMC10786418; doi:10.1126/sciadv.adj9695)
Supplement: Supplementary file 1 — Sections S1 to S21 Figs. S1 to S22 Tables S1 to S13 References [file sciadv.adj9695_sm.pdf]

Science Advances
AAAS

Supplementary Materials for

Taming nonclassical carbocations to control small ring reactivity

Ryan E. McNamee *et al.*

Corresponding author: Fernanda Duarte, fernanda.duartegonzalez@chem.ox.ac.uk; Edward A. Anderson, edward.anderson@chem.ox.ac.uk

Sci. Adv. **10**, eadj9695 (2024)
DOI: 10.1126/sciadv.adj9695

This PDF file includes:

Sections S1 to S21
Figs. S1 to S22
Tables S1 to S13
References

Other Supplementary Material for this manuscript includes the following:

Data files S1 and S2

1. Materials and Methods

NMR Spectroscopy: Proton (^{1}H), carbon (^{13}C) and fluorine (^{19}F) NMR spectra were recorded on Bruker AVIII HD 400, NEO 400, AVIII HD 500, AVII 500, AVII 600 or AVII 700 spectrometers. ^{1}H, ^{13}C, and ^{19}F chemical shifts (δ) are quoted in parts per million (ppm). ^{1}H NMR spectra were recorded using an internal deuterium lock for the residual protons in chloroform-*d* (δ = 7.26) or dimethylsulfoxide-*d* (δ = 2.50). ^{13}C NMR spectra were recorded using an internal deuterium lock in chloroform-*d* (δ = 77.16) or dimethylsulfoxide-*d* (δ = 39.52). Assignments were determined either based on unambiguous chemical shift or coupling patterns, COSY, HSQC, HMBC and/or NOESY experiments. Peak multiplicities are defined as s (singlet), d (doublet), t (triplet), q (quartet), quin (quintet), sext (sextet), sept (septet), m (multiplet) and br (broad). Coupling constants (*J*) are reported to the nearest 0.1 Hz.

Mass Spectroscopy: High-resolution mass spectra (HRMS) were recorded by the Departmental Mass Spectrometry Service, University of Oxford on a Thermo Scientific Exactive Mass Spectrometer (Waters Equity autosampler and pump) for electrospray ionization (ESI) and an Agilent 7200 Accurate Mass QTOF GCMS (using a SIM Direct Insertion Probe) for electron ionization (EI) and chemical ionization (CI). High-resolution values are calculated to 4 decimal places from the molecular formula, and all values are within a tolerance of 5 ppm.

Infrared Spectroscopy: Infrared spectra were obtained on a Bruker Tensor 27 FT-IR spectrometer. The samples were prepared as a thin film on a diamond ATR module. Wavelengths of maximum absorbance (ν_{max}) are quoted in cm^{-1}. Only selected, characteristic IR absorption data are provided for each compound.

Chromatography: Column chromatography referred to normal phase column chromatography and was performed on silica gel obtained from Merck (Silica gel Si 60, 0.040-0.063 mm) under positive nitrogen pressure, using the stated solvent system. Analytical thin-layer chromatography was performed on pre-coated aluminium-backed plates (Merck Kieselgel 60 F_{254} plates) with visualization by ultraviolet light (254 nm) and/or by staining with phosphomolybdic acid and potassium permanganate. Retention factors (R_f) are reported with the solvent system in parentheses.

Materials/procedures: All air- or moisture-sensitive reactions were carried out in anhydrous solvents in heatgun-dried glassware under a nitrogen atmosphere. Light-sensitive reactions were carried out under aluminium foil protection. Heating was performed using an oil bath. Dry tetrahydrofuran, CH_2Cl_2, and diethyl ether were collected fresh from an mBraun SPS-800 solvent purification system, having been passed through anhydrous alumina columns. All other commercially available reagents and solvents, where appropriate, were dried and purified before use using standard procedures.

Single Crystal X-ray Diffraction Studies: data were collected using a (Rigaku) Oxford Diffraction SuperNova diffractometer for **3b, 3m, 3n, 3p, 3x, 3y** and **3aa**, or beamline I19-1, Diamond Light Source for **3z**. Raw frame data were reduced using CrysAlisPro, and the structures were solved using 'Superflip'21 before refinement with CRYSTALS (*44, 45*) as per the SI (CIF).

2. Synthesis of bicyclo[1.1.0]butanes

Bicyclo[1.1.0]butanes were prepared according to *J. Am. Chem. Soc.* **143**, 21246 (2021); *J. Am. Chem. Soc.* **135**, 9283 (2013); *Org. Lett.* **15**, 310 (2013), or from processes under development or outlined below.

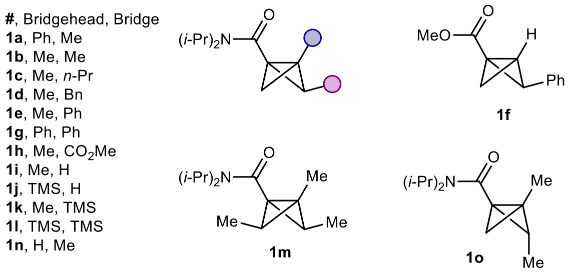

tert-Butyl(((1*S**,3*S**)-2,2-dibromo-1,3-dimethylcyclopropyl)methoxy)dimethylsilane, S1

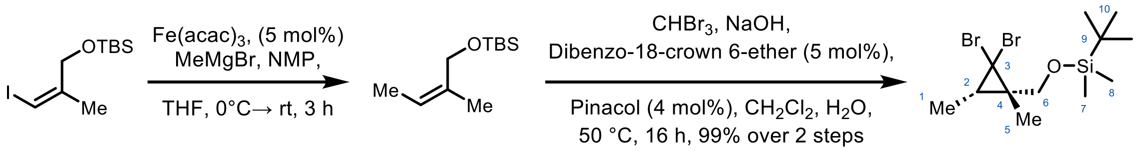

To a solution of alkenyl iodide (1.56 g, 5.0 mmol, 1.0 eq.) and $Fe(acac)_3$ (88 mg, 0.25 mmol, 5 mol%) in a mixture of NMP (8 mL) and THF (10 mL) was added MeMgBr (5.0 mL, 3.0 M in Et_2O, 15.0 mmol, 3.0 eq.) at 0 °C. The reaction was brought to rt and stirred for 3 h before cooling to 0 °C and quenching with sat. aq. NH_4Cl (5.0 mL). The reaction was diluted with Et_2O (100 mL), washed with water (3 x 100 mL), dried with $MgSO_4$ and filtered. The reaction mixture was concentrated *in vacuo* (400 mbar, 35 °C) to afford the trisubstituted alkene as a light-yellow oil. Data identical to literature values (*46*).

The oil was dissolved in a mixture of CH_2Cl_2 (6.2 mL) and $CHBr_3$ (3.5 mL), and to this solution dibenzo-18-crown-6-ether (91 mg, 0.25 mmol, 5.0 mol%) and pinacol (24 mg, 0.20 mmol, 4 mol%) were added. The solution was heated to 50 °C and aq. NaOH (3.8 mL, 50% wt in water) was added dropwise via the reflux condenser with rapid stirring. The mixture was stirred for 16 h at 50 °C before cooling to rt and diluting with Et_2O (100 mL) and water (100 mL). The layers were separated, and the aqueous phase was extracted with Et_2O (2 x 100 mL). The organic phases were combined, dried with $MgSO_4$ and filtered. The mixture was concentrated *in vacuo*, and the residue was filtered through a silica pad with pentane elution. The mixture was concentrated *in vacuo* to afford the title compound (1.85 g, 5.0 mmol, 99%) as a clear oil.

R_f = 0.39 (pentane)

IR (thin film, ν_{max} / cm^{-1}): 3393, 1034, 998, 763

HRMS (ESI^+) calc. for $C_{12}H_{24}{}^{79}Br_2OSi$ $[M+H]^+$ 291.0780, not found

^{1}H NMR (400 MHz, Chloroform-*d*): δ 3.66 (d, *J* = 10.5 Hz, 1H, 6), 3.59 (d, *J* = 10.5 Hz, 1H, 6), 1.40 (s, 3H, 5), 1.37 (q, *J* = 6.5 Hz, 1H, 2), 1.17 (d, *J* = 6.5 Hz, 3H, 1), 0.90 (s, 9H, 10), 0.07 (s, 3H, 7/8), 0.05 (s, 3H, 7/8)

^{13}C NMR (101 MHz, Chloroform-*d*): δ 65.2 (6), 46.3 (3), 34.8 (2), 32.2 (4), 26.1 (10), 23.0 (5), 18.5 (9), 12.3 (1), -5.1 (7/8), -5.2 (7/8)

((1*S,3*S**)-2,2-Dibromo-1,3-dimethylcyclopropyl)methanol, S2**

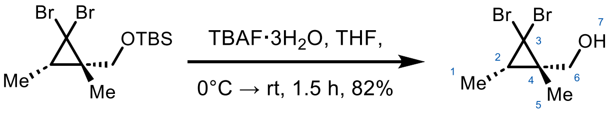

To a solution of **S1** (1.85 g, 5.0 mmol, 1.0 eq.) in THF (50 mL) was added TBAF trihydrate (2.21 g, 7.0 mmol, 1.4 eq.), at 0 °C. The reaction was brought to rt and stirred for 1.5 h before diluting with Et_2O (100 mL) and water (100 mL). The layers were separated, and the aqueous phase was extracted with Et_2O (2 x 100 mL). The organic phases were combined, dried with $MgSO_4$, filtered and concentrated *in vacuo*. The residue was purified via flash chromatography (20→40% Et_2O in pentane) to yield the title compound (1.07 g, 4.15 mmol, 83%) as a colourless solid.

R_f = 0.24 (40% Et_2O in pentane)

IR (thin film, ν_{max} / cm^{-1}): 3393

HRMS (ESI$^+$) calc. for $C_6H_{10}{}^{79}Br_2ONa$ $[M+Na]^+$ 278.8991, found 278.8932

^{1}H NMR (400 MHz, Chloroform-*d*): δ 3.74 (dd, J = 11.9, 5.1 Hz, 1H, 6), 3.69 (dd, J = 11.8, 7.8 Hz, 1H, 6), 1.53 (dd, J = 7.8, 5.2 Hz, 1H, 7), 1.49 (s, 3H, 5), 1.44 (q, J = 6.6 Hz, 1H, 2), 1.21 (d, J = 6.6 Hz, 3H, 1).

^{13}C NMR (101 MHz, Chloroform-*d*): δ 65.7 (6), 45.6 (3), 35.3 (2), 32.3 (4), 22.6 (5), 12.1 (1)

(2*S,3*S**)-1,1-Dibromo-2-(bromomethyl)-2,3-dimethylcyclopropane, S3**

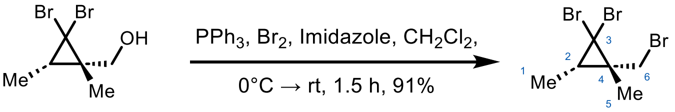

To a solution of PPh_3 (197 mg, 0.75 mmol, 1.5 eq.) in CH_2Cl_2 (0.6 mL) was added Br_2 (39 µL, 0.75 mmol, 1.5 eq.) at 0 °C. The reaction was stirred for 10 min. at 0 °C before addition of imidazole (68 mg, 1.00 mmol, 2.0 eq.) at 0 °C. The mixture was stirred for a further 5 min at 0 °C before addition of **S2** in CH_2Cl_2 (0.5 mL) at 0 °C. The reaction was stirred for 1.5 h at rt followed by dilution with pentane (20 mL) and filtering through a silica pad with pentane elution (20 mL). The filtrate was concentrated *in vacuo* to yield the title compound (145 mg, 0.46 mmol, 91%) as a colourless oil.

IR (thin film, ν_{max} / cm^{-1}): no characteristic peak

HRMS (ESI$^+$) calc. for $C_6H_{10}{}^{79}Br_3$ $[M+H]^+$ 178.0477, not found

^{1}**H NMR** (400 MHz, Chloroform-*d*): δ 3.55 (d, *J* = 10.7 Hz, 1H, 6), 3.51 (d, *J* = 10.6 Hz, 1H, 6), 1.57 (s, 3H, 5), 1.54 (q, *J* = 6.6 Hz, 1H, 2), 1.23 (d, *J* = 6.5 Hz, 3H, 1)

^{13}**C NMR** (101 MHz, Chloroform-*d*): δ 45.7 (3), 38.1 (6), 36.7 (2), 31.4 (4), 24.3 (5), 12.1 (1)

(1*S,3*S**)-*N*,*N*-Diisopropyl-2,3-dimethylbicyclo[1.1.0]butane-1-carboxamide, 1p**

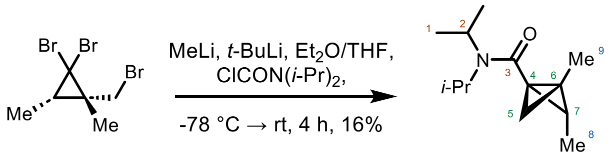

To a solution of **S3** (140 mg, 0.44 mmol, 1.0 eq.) in Et_2O (4.4 mL) at −78 °C was added MeLi (0.24 mL, 2.2 M LiBr complex in Et_2O, 0.53 mmol, 1.2 eq.), the reaction was stirred at −78 °C for 1 h before addition of *t*-BuLi (0.35 mL, 1.5 M in pentane, 0.53 mmol, 1.2 eq.). The reaction was stirred for 1 h before transferring to a solution of *N*-diisopropylcarbamoyl chloride (360 mg, 2.20 mmol, 5.0 eq.) in THF (4.4 mL) at 0 °C. The solution was stirred for 2 h at 0 °C before being diluted with Et_2O (20 mL) and washed with water (2 x 20 mL). The organic phase was dried with $MgSO_4$, filtered and concentrated *in vacuo*. The residue was purified via flash chromatography (10→30% Et_2O in pentane, 1% NEt_3) to yield the title compound (15 mg, 0.07 mmol, 16%) as a colourless oil.[a]

[a]Metal-halogen exchange was performed using PhLi, *n*-BuLi, *t*-BuLi, *i*-PrMgCl and *i*-PrMgBr•LiCl in Et_2O/THF with temperatures ranging from −100 °C to –45 °C, and in all cases the reaction led to decomposition. It was later discovered that the first metal-Halogen exchange is highly sensitive and must be carried out with MeLi. Yields of >50% were obtained with chloroformate esters as electrophiles.

R_f = 0.24 (20% Et_2O in pentane)

IR (thin film, ν_{max} / cm^{-1}): 1623, 1442, 1377, 1343

HRMS (ESI$^+$) calc. for $C_{13}H_{24}NO$ [M+H]$^+$ 210.1852, found 210.1850

^{1}**H NMR** (400 MHz, Chloroform-*d*): δ 4.62 (sept, *J* = 6.7 Hz, 1H, 2), 3.34 (sept, *J* = 6.8 Hz, 1H, 2), 2.59 (qd, *J* = 5.9, 4.0 Hz, 1H, 7), 2.16 (dd, *J* = 4.1, 1.4 Hz, 1H, 5-exo), 1.75 (s, 0H, 5-endo), 1.46 (s, 3H, 9), 1.40 (d, *J* = 6.8 Hz, 3H, 1), 1.43 (d, *J* = 6.8 Hz, 3H, 1), 1.19 (d, *J* = 6.1 Hz, 3H, 1), 1.17 (d, *J* = 6.1 Hz, 3H, 1) 0.89 (d, *J* = 5.9 Hz, 3H, 8)

^{13}**C NMR** (101 MHz, Chloroform-*d*): δ 171.0 (3), 49.5 (2), 47.2 (7), 45.9 (2), 37.0 (5), 29.1 (4), 21.4 (1), 21.3 (1), 21.2 (1), 21.1 (1), 16.8 (6), 13.1 (9), 9.2 (8)

3. Screening of conditions for acid-mediated ring-opening additions

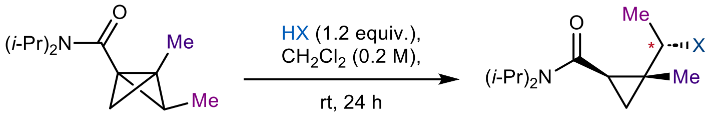

Entry	Acid	Variation to conditions	*dr*	Conversion (%)
1	AcOH	–	1 : 3	100
2	TFA	–	1 : 9	100
3	HCl	–	1: 13	100
4	AcOH	$CHCl_3$, $ClCH_2CH_2Cl$	1 : 3	100
5	AcOH	EtOAc	1 : 4	47
6	AcOH	THF	1 : 3	35
7	AcOH	Et_2O	1 : 3	50
8	AcOH	-30 °C	1 : 4	71
9	AcOH	0 °C	1 : 4	100
10	AcOH	40 °C	1 : 3	100
11	AcOH	0.025, 0.05, 0.1, 0.4 M	1 : 3	100
12	AcOH	1, 2, 4, 8 equiv. of Bu_4NOAc	1 : 3	100

Reactions were conducted on 0.1 mmol scale (0.1 M). Conversion based on starting material to product ratio as determined by ^{1}H NMR spectroscopic analysis of the crude reaction mixture. Diastereomeric ratio (*dr*) arises from indicated stereocenter.

4. General procedure for addition of Brønsted acids

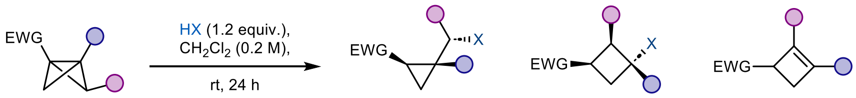

To a solution of BCB (0.20 mmol, 1.0 eq.) in CH_2Cl_2 (1 mL) was added acid (0.24 mmol, 1.2 eq.) at rt. The reaction was stirred for 24 h at rt before concentrating *in vacuo*. The residue was purified *via* flash silica chromatography.

Note: We discovered in this work that our original stereochemistry assignment for **3b** (J. Am. Chem. Soc. 2021, 143, 21246) was incorrect. The stereochemistry of nucleophilic ring opening of **3b**, **3m**, **3n**, **3p**, **3x**, **3y**, **3aa** and **3z** has been assigned based on X-ray crystallography, and by analogy, in this manuscript, including **3b**.

5. Procedures and characterisation for ring-opening of BCBs

N,N-Diisopropyl-2,3-*d*iphenylcyclobut-2-ene-1-carboxamide, 2b

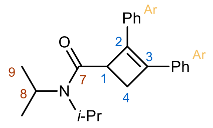

Prepared according to the general procedure using trifluoroacetic acid (19 µL, 0.24 mmol, 1.2 equiv.). Upon completion, the reaction mixture was concentrated *in vacuo,* and the residue was purified *via* flash chromatography (20→30% Et_2O in pentane) to yield the title compound (54 mg, 0.16 mmol, 81%) as a colourless solid.

R$_f$ = 0.16 (20% Et_2O in pentane)

IR (thin film, ν_{max} / cm^{-1}): 1635, 1443, 1340

HRMS (ESI^+) calc. for $C_{23}H_{28}ON$ $[M+H]^+$ 334.2165, found 334.2156

^{1}H NMR (600 MHz, Chloroform-*d*): δ 7.54 – 7.51 (m, 2H, Ar), 7.49 – 7.46 (m, 2H, Ar), 7.33 – 7.27 (m, 4H, Ar), 7.26 – 7.22 (m, 2H, Ar), 4.08 (sept, *J* = 6.5 Hz, 1H, 8), 4.05 (dd, *J* = 5.2, 2.2 Hz, 1H, 1), 3.52 (br, 1H, 8), 3.00 (dd, *J* = 12.5, 5.2 Hz, 1H, 4), 2.87 (dd, *J* = 12.5, 2.2 Hz, 1H, 4), 1.41 (d, *J* = 6.8 Hz, 3H, 9), 1.39 (d, *J* = 6.7 Hz, 3H, 9), 1.25 (d, *J* = 6.7 Hz, 3H, 9), 1.18 (d, *J* = 6.7 Hz, 3H, 9).

^{13}C NMR (151 MHz, Chloroform-*d*): δ 171.3 (7), 139.0 (2/3), 138.7 (2/3), 135.7 (Ar), 135.4 (Ar), 128.4 (Ar), 128.4 (Ar), 128.0 (Ar), 127.7 (Ar), 127.0 (Ar), 126.4 (Ar), 48.1 (8), 45.9 (8), 43.1 (1), 32.2 (4), 21.5 (9), 21.3 (9), 21.0 (9), 20.7 (9).

Methyl 4-(diisopropylcarbamoyl)-2-methylcyclobut-1-ene-1-carboxylate, 2c

Methyl (1*R**,2*S**,4*S**)-4-(diisopropylcarbamoyl)-2-methyl-2-(2,2,2-trifluoroacetoxy)cyclobutane-1-carboxylate, S6

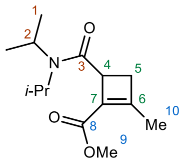

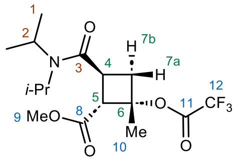

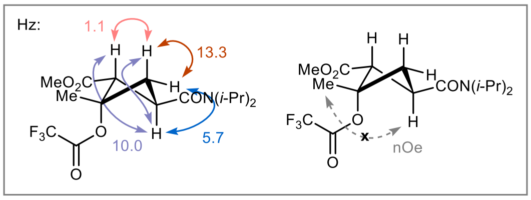

Prepared according to the general procedure using trifluoroacetic acid (19 µL, 0.24 mmol, 1.2 eq.). Upon completion, the reaction mixture was concentrated *in vacuo,* and the residue was purified *via* flash chromatography (15→40% EtOAc in pentane) to yield **2c** (13 mg, 0.05 mmol, 36%) colourless oil and **S6** (4 mg, 0.01 mmol, 16%) as a colourless oil.

2c: ***R$_f$*** = 0.50 (40% EtOAc in pentane)

IR (thin film, ν_{max} / cm^{-1}): 1783, 1746, 1641, 1222, 1172

HRMS (ESI^+) calc. for $C_{16}H_{25}O_5NF_3$ $[M+H]^+$ 368.1679, found 368.1685

^{1}H NMR (600 MHz, Chloroform-*d*): δ 3.70 (s, 3H, 9), 3.68 (d, *J* = 10.0 Hz, 1H, 5), 3.66 (sept, *J* = 6.7 Hz, 1H, 2), 3.53 (td, *J* = 10.1, 5.7 Hz, 1H, 4), 3.47 (br, 1H, 2), 2.76 (dd, *J* = 13.2, 5.7 Hz, 1H, 7b), 2.61 (ddd, *J* = 13.0, 9.9, 1.1 Hz, 1H, 7a), 1.82 (s, 3H, 10), 1.37 (d, *J* = 6.8 Hz, 6H, 1), 1.18 (d, *J* = 6.7 Hz, 3H, 1), 1.15 (d, *J* = 6.7 Hz, 3H, 1)

^{13}C NMR (151 MHz, Chloroform-*d*): δ 169.3 (3/8), 169.2 (3/8), 156.1 (q, *J* = 42.2 Hz, 11), 114.4 (q, *J* = 286.3 Hz, 12), 85.9 (6), 51.7 (9), 50.1 (4), 48.5 (2), 46.0 (2), 36.1 (7), 34.6 (5), 21.4 (1), 20.8 (1), 20.7 (1), 20.3 (1), 20.1 (10)

S6: ***R$_f$*** = 0.25 (40% EtOAc in pentane)

IR (thin film, ν_{max} / cm^{-1}): 1716, 1637, 1439, 1337

HRMS (ESI^+) calc. for $C_{14}H_{23}O3H$ $[M+H]^+$ 254.1762, not found 338.1937

^{1}H NMR (600 MHz, Chloroform-*d*): δ 4.07 (sept, *J* = 6.8 Hz, 1H, 2), 3.82 (dt, *J* = 4.3, 2.1 Hz, 1H, 4), 3.72 (s, 3H, 9), 3.49 (br, 1H, 2), 2.58 – 2.48 (m, 2H, 5), 2.06 (q, *J* = 1.5 Hz, 3H, 10), 1.38 (d, *J* = 3.0 Hz, 3H, 1), 1.37 (d, *J* = 3.0 Hz, 3H, 1), 1.25 (d, *J* = 6.6 Hz, 3H, 1), 1.20 (d, *J* = 6.7 Hz, 3H, 1)

^{13}C NMR (151 MHz, Chloroform-*d*): δ 170.6 (3), 163.0 (8), 159.9 (6), 130.4 (7), 51.1 (9), 48.3 (2), 45.8 (2), 41.4 (4), 35.3 (5), 21.4 (1), 21.2 (1), 20.9 (1), 20.7 (1), 16.7 (10)

***N,N*-Diisopropyl-3-methyl-2-(trimethylsilyl)cyclobut-2-ene-1-carboxamide, 2e**

***N,N*-Diisopropyl-3-methylcyclobut-2-ene-1-carboxamide, 2d**

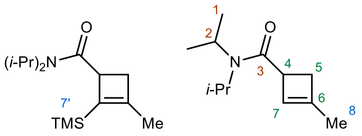

Prepared according to the general procedure using trifluoroacetic acid (19 µL, 0.24 mmol, 1.2 eq.). Upon completion, the reaction mixture was concentrated *in vacuo,* and the residue was purified *via* flash chromatography (10→40% Et_2O in pentane) to yield **2e** (18 mg, 0.09 mmol, 45%) colourless oil and **2d** (15 mg, 0.10 mmol, 52%) as a yellow oil.

2e: ***R$_f$*** = 0.64 (20% Et_2O in pentane)

IR (thin film, ν_{max} / cm^{-1}): 1639, 843

HRMS (ESI^+) calc. for $C_{15}H_{30}ONSi$ $[M+H]^+$ 268.2091, found 268.2091

^{1}H NMR (600 MHz, Chloroform-*d*): δ 3.90 (sept, *J* = 6.7 Hz, 1H, 2), 3.51 – 3.45 (m, 2H, 2 and 4), 2.73 (ddd, *J* = 13.4, 4.9, 1.4 Hz, 1H, 5), 2.55 (d, *J* = 13.4 Hz, 1H, 5), 1.79 (s, 3H, 8), 1.37 (d, *J* = 6.8 Hz, 3H, 1), 1.35 (d, *J* = 6.8 Hz, 3H, 1), 1.19 (d, *J* = 6.8 Hz, 6H, 1), 0.11 (s, 9H, 7’)

^{13}C NMR (151 MHz, Chloroform-*d*): δ 172.9 (3), 157.3 (6), 144.7 (7), 47.7 (2), 45.5 (2), 42.7 (4), 38.5 (5), 21.5 (1), 21.3 (1), 21.1 (1), 20.6 (1), 18.1 (8), -0.8 (7')

2d: ***R_f*** = 0.12 (20% Et_2O in pentane)

IR (thin film, ν_{max} / cm^{-1}): 1638, 1440

HRMS (ESI$^+$) calc. for $C_{12}H_{22}ON$ $[M+H]^+$ 196.1692, found 196.1696

^{1}H NMR (600 MHz, Chloroform-*d*): δ 5.81 – 5.78 (m, 1H, 7), 3.93 (sept, *J* = 6.8 Hz, 1H, 2), 3.53 – 3.50 (m, 1H, 4), 3.37 (br, 1H, 2), 2.63 – 2.61 (m, 2H, 5), 1.76 – 1.71 (m, 3H, 8), 1.40 (d, *J* = 1.9 Hz, 3H, 1), 1.39 (d, *J* = 1.9 Hz, 3H, 1), 1.18 (d, *J* = 6.7 Hz, 6H, 1)

^{13}C NMR (151 MHz, Chloroform-*d*): δ 172.3 (3), 147.2 (6), 127.6 (7), 48.1 (2), 45.7 (2), 42.7 (4), 36.5 (5), 21.1 (1), 21.0 (1), 20.9 (1), 20.8 (1), 16.9 (8)

(*S)-1-((1*R**,2*R**)-2-(Diisopropylcarbamoyl)-1-methylcyclopropyl)ethyl acetate, 3b**

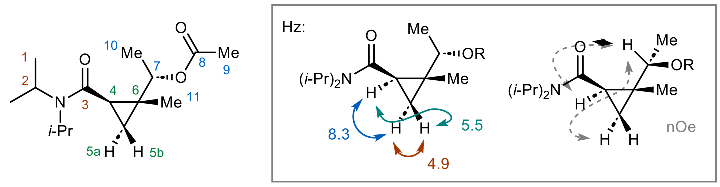

Prepared according to the general procedure using acetic acid (14 µL, 0.24 mmol, 1.2 eq.). Upon completion, the reaction mixture was concentrated *in vacuo,* and the residue was purified *via* flash chromatography (20→60% Et_2O in pentane) to yield the title compound (47 mg, 0.17 mmol, *dr* 4:1, 86%) as a colourless oil.

R_f = 0.24 (50% Et_2O in pentane)

IR (thin film, ν_{max} / cm^{-1}): 1735, 1639, 1246

HRMS (ESI$^+$) calc. for $C_{15}H_{28}O_3N$ $[M+H]^+$ 270.2064, found 270.2058

^{1}H NMR (600 MHz, Chloroform-*d*): δ 4.73 (q, *J* = 6.4 Hz, 0.25H, 7'), 4.38 (sept, *J* = 6.7 Hz, 1.25H, 2 and 2'), 4.33 (q, *J* = 6.5 Hz, 1H, 7), 3.39 (br, 1H, 2 and 2'), 2.05 (s, 3H, 9), 2.04 (s, 0.75H, 9'), 1.76 (dd, *J* = 8.3, 5.5 Hz, 1H, 4), 1.69 (dd, *J* = 8.4, 5.4 Hz, 0.25H, 4'), 1.40 (d, *J* = 6.8 Hz, 3.75H, 1 and 1'), 1.37 (d, *J* = 6.7 Hz, 3.75H, 1 and 1'), 1.29 (d, *J* = 6.5 Hz, 3H, 10), 1.24 – 1.15 (m, 9.5H, 1, 1', 5b, 5b', 10'), 1.06 (s, 3.75H, 11 and 11'), 0.83 (dd, *J* = 8.4, 4.7 Hz, 0.25H, 5a'), 0.69 (dd, *J* = 8.3, 4.9 Hz, 1H, 5a). Numbers denoted with a prime correspond to the minor diastereomer (epimer at position 7). Integration of 0.25H for the minor diastereomer corresponds to 1H'. The diastereomer ratio was determined by ^{1}H integration of signals 4/4', and combined integration of signals 10/10 and 11/11'.

^{13}C NMR (151 MHz, Chloroform-*d*): δ 170.6 (8'), 170.5 (8), 168.6 (3'), 168.4 (3), 78.0 (7), 75.1 (7'), 48.6 (2'), 48.5 (2), 45.9 (2 and 2'), 29.1 (4), 26.9 (6), 26.7 (6'), 25.6 (4'), 21.4 (9'), 21.3 (9), 21.2 (1, 1 and 1'), 20.9 (1 and 1'), 20.6 (1 and 1'), 17.2 (10), 17.1 (10'), 15.6 (5), 15.4 (5'), 14.0 (11'), 11.5 (11).

Single Crystal Data for **3b**: C17H27F3N2O4, Mr =380.41. 150 K – monoclinic, P $2_1/c$, a = 9.5839(2) Å, b = 19.9028(5) Å, c = 10.5952(2) Å, β = 100.972(2)°, V = 1984.05(8) Å^3, Data/restraints/parameters – 4110/334/273, Rint = 0.019, Final R1 = 0.0397, wR2 = 0.0945 (I>2σ(I)).

(*S)-1-((1*R**,2*R**)-2-(Diisopropylcarbamoyl)-1-methylcyclopropyl)ethyl 2,2,2-trichloroacetate, 3c**

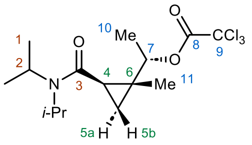

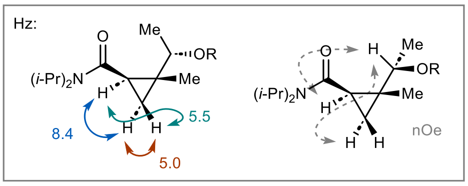

Prepared according to the general procedure using trichloroacetic acid (39 mg, 0.24 mmol, 1.2 eq.). Upon completion, the reaction mixture was concentrated *in vacuo,* and the residue was purified *via* flash chromatography (15→40% Et_2O in pentane) to yield the title compound (64 mg, 0.17 mmol, *dr* 10:1, 84%) as a colourless oil.

***R**$_f$* = 0.26 (40% Et_2O in pentane)

IR (thin film, ν_{max} / cm^{-1}): 1760, 1639, 1251

HRMS (ESI$^+$) calc. for $C_{12}H_{25}O_3{}^{35}Cl_3N$ [M+H]$^+$ 372.0895, found 372.0892

^{1}H NMR (600 MHz, Chloroform-*d*): δ 4.88 (q, *J* = 6.4 Hz, 0.1H, 7'), 4.44 (q, *J* = 6.4 Hz, 1H, 7'), 4.29 (hept, *J* = 6.6 Hz, 1.1H, 2 and 2'), 3.41 (br, 1.1H, 2 and 2'), 1.88 (dd, *J* = 8.4, 5.4 Hz, 1H, 4), 1.82 (dd, *J* = 8.5, 5.5 Hz, 0.1H, 4'), 1.46 (d, *J* = 6.4 Hz, 3H, 10), 1.42 – 1.34 (m, 8H, 1, 1', 10', 5b, 5b'), 1.29 – 1.23 (m, 0.3H, 1'), 1.20 (d, *J* = 6.7 Hz, 3.3H, 1 and 1'), 1.17 (d, *J* = 6.7 Hz, 3H, 1), 1.15 (s, 3H, 11), 1.13 (s, 0.3H, 11'), 0.92 (dd, *J* = 8.5, 4.9 Hz, 0.1H, 5a'), 0.74 (dd, *J* = 8.4, 5.0 Hz, 1H, 5a). Numbers denoted with a prime correspond to the minor diastereomer (epimer at position 7). Integration of 0.1H for the minor diastereomer corresponds to 1H'. The diastereomer ratio was determined by ^{1}H integration of signals 7/7', 4/4', 11/11' and combined integration of signals 2/2'.

^{13}C NMR (151 MHz, Chloroform-*d*): δ 168.0 (3'), 167.8 (3), 161.8 (8), 161.7 (8'), 90.3 (9), 84.4 (7), 81.2 (7'), 48.8 (2 and 2'), 46.0 (2 and 2'), 29.1 (4), 27.0 (6), 26.6 (6'), 25.3 (4'), 21.5 (1 and 1'), 21.2 (1 and 1'), 21.0 (1 and 1'), 20.6 (1 and 1'), 16.7 (10), 16.6 (10'), 15.6 (5), 15.2 (5'), 13.9 (11'), 11.2 (11). Carbon 9' of the minor diastereomer could not be assigned due to low-intensity/overlapping signals.

(S^*)-1-((1R^*,2R^*)-2-(Diisopropylcarbamoyl)-1-methylcyclopropyl)ethyl 2,2,2-trifluoroacetate, 3d

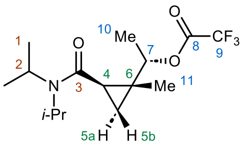

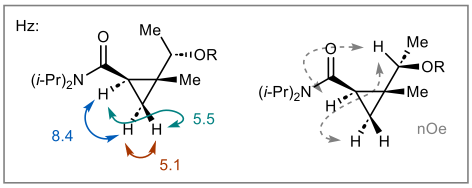

Prepared according to the general procedure using trifluoroacetic acid (19 µL, 0.24 mmol, 1.2 eq.). Upon completion, the reaction mixture was concentrated *in vacuo,* and the residue was purified *via* flash chromatography (20→60% Et_2O in pentane) to yield the title compound (54 mg, 0.17 mmol, *dr* 10:1, 83%) as a colourless oil.

R_f = 0.32 (50% Et_2O in pentane)

IR (thin film, ν_{max} / cm^{-1}): 1782, 1641, 1221, 1172

HRMS (ESI$^+$) calc. for $C_{15}H_{24}F_3O_3N$ [M+H]$^+$ 324.1781, found 324.1865

^{1}H NMR (600 MHz, Chloroform-*d*): δ 4.95 (q, *J* = 6.4 Hz, 0.1H, 7'), 4.44 (q, *J* = 6.4 Hz, 1H, 7), 4.33 – 4.20 (m, 1.1H, 2 and 2'), 3.40 (sept, *J* = 6.8 Hz, 1.1H, 2 and 2'), 1.79 (dd, *J* = 8.4, 5.5 Hz, 1.1H, 4 and 4'), 1.46 (d, *J* = 6.3 Hz, 3H, 10), 1.42 – 1.32 (m, 8H, 1, 1', 10', 5b, 5b'), 1.23 – 1.19 (m, 0.6H, 1'), 1.19 – 1.13 (m, 6H, 1), 1.11 (s, 3.3H, 11 and 11'), 0.88 (dd, *J* = 8.5, 5.2 Hz, 0.1H, 5a'), 0.73 (dd, *J* = 8.4, 5.1 Hz, 1H, 5a). Numbers denoted with a prime correspond to the minor diastereomer (epimer at position 7). Integration of 0.1H for the minor diastereomer corresponds to 1H'. The diastereomer ratio was determined by ^{1}H integration of signals 7/7', combined integration of signals 2/2' and 11/11', and ^{19}F integration of CF_3.

^{13}C NMR (151 MHz, Chloroform-*d*): δ 167.7 (3'), 167.5 (3), 157.3 (q, *J* = 42.0 Hz, 8 and 8'), 114.8 (q, *J* = 286.0 Hz, 9 and 9'), 84.0 (7), 81.0 (7'), 48.8 (2 and 2'), 46.0 (2 and 2'), 29.4 (4), 26.5 (6), 26.2 (6'), 25.2 (4'), 21.2 (1 and 1'), 20.7 (1, 1 and 1'), 20.5 (1 and 1'), 16.9 (10), 16.5 (10'), 15.8 (5'), 15.7 (5), 13.0 (11'), 11.0 (11).

^{19}F NMR (565 MHz, Chloroform-*d*): δ -75.1 (9), -75.2 (9')

(R^*)-1-((1R^*,2R^*)-2-(Diisopropylcarbamoyl)-1-methylcyclopropyl)ethyl 4-nitrobenzoate, 3e

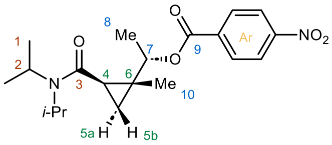

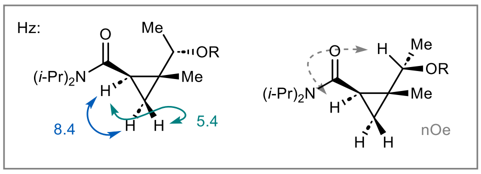

Prepared according to the general procedure using 4-Nitroxybenzoic acid (40 mg, 0.24 mmol, 1.2 eq.). Upon completion, the reaction mixture was concentrated *in vacuo*, dry loaded onto silica and purified *via* flash chromatography (20→40% EtOAc in pentane) to yield the title compound (67 mg, 0.18 mmol, *dr* 5.9:1, 89%) as a colourless solid.

R_f = 0.47 (40% EtOAc in pentane)

IR (thin film, ν_{max} / cm^{-1}): 1721, 1636, 1529, 1275

HRMS (ESI^{+}) calc. for $C_{12}H_{21}O_2NNa$ [M+Na]$^{+}$ 377.2071, found 377.2072

^{1}H NMR (600 MHz, Chloroform-*d*): δ 8.33 – 8.28 (m, 2.34H, Ar and Ar'), 8.23 (d, *J* = 9.1 Hz, 2H, Ar), 8.19 (d, *J* = 9.1 Hz, 0.34H, Ar), 4.92 (q, *J* = 6.4 Hz, 0.17H, 7'), 4.70 (q, *J* = 6.5 Hz, 1H, 7), 4.34 (sept, *J* = 6.7 Hz, 0.17H, 2'), 4.20 (sept, *J* = 6.8 Hz, 1H, 2), 3.44 (br, 0.17H, 2'), 3.31 (br, 1H, 2), 1.89 (dd, *J* = 8.4, 5.5 Hz, 1H, 4), 1.76 (dd, *J* = 8.4, 5.4 Hz, 0.17H, 4'), 1.46 (d, *J* = 6.5 Hz, 3H, 8), 1.43 (d, *J* = 6.4 Hz, 0.51H, 8'), 1.40 – 1.35 (m, 3H, 1), 1.34 – 1.30 (m, 4H, 1 and 5b), 1.27 (t, *J* = 5.1 Hz, 0.17H, 5b'), 1.23 (d, *J* = 6.7 Hz, 0.51H, 1'), 1.21 – 1.16 (m, 3.51H, 10 and 1'), 1.17 (s, 0.51H, 10'), 0.99 (d, *J* = 6.7 Hz, 3H, 1), 0.93 (dd, *J* = 8.5, 4.8 Hz, 0.17H, 5a'), 0.83 – 0.78 (m, 3.51H, 5a, 1 and 1'). Numbers denoted with a prime correspond to the minor diastereomer (epimer at position 7). Integration of 0.17H for the minor diastereomer corresponds to 1H'. The diastereomer ratio was determined by ^{1}H integration of signals 2/2', 4/4' and 7/7'

^{13}C NMR (151 MHz, Chloroform-*d*): δ 168.1 (3'), 168.1 (3), 164.2 (9 and 9'), 150.8 (Ar), 150.7 (Ar), 136.0 (Ar'), 135.8 (Ar), 130.8 (Ar and Ar'), 123.8 (Ar), 123.7 (Ar'), 79.5 (7 and 7'), 48.8 (2'), 48.5 (2), 46.0 (2'), 45.9 (2), 29.33 (4), 27.3 (6), 27.0 (6'), 26.0 (4), 21.3 (1'), 21.23 (1), 21.2 (1 and 1'), 21.0 (1'), 20.7 (1), 20.6 (1'), 20.5 (1), 17.5 (8'), 17.3 (8), 16.0 (5'), 15.6 (5), 13.3 (10'), 11.4 (10)

(*R**)-1-((1*R**,2*R**)-2-(Diisopropylcarbamoyl)-1-methylcyclopropyl)ethyl 4-methoxybenzoate, 3f

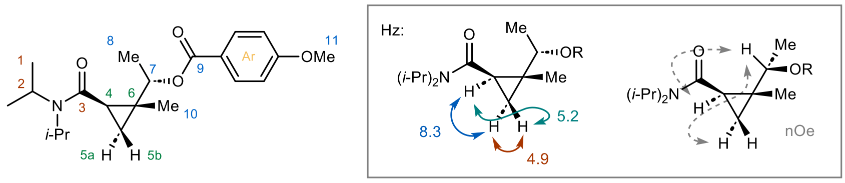

Prepared according to the general procedure using 4-methoxybenzoic acid (37 mg, 0.24 mmol, 1.2 eq.). Upon completion the reaction mixture was concentrated *in vacuo*, dry loaded onto silica and purified *via* flash chromatography (20→40% EtOAc in pentane) to yield the title compound (57 mg, 0.16 mmol, *dr* 4:1, 79 %) as a colourless oil.

***R**$_f$* = 0.47 (40% EtOAc in pentane)

IR (thin film, ν_{max} / cm^{-1}): 1708, 1637, 1606, 1258

HRMS (ESI^{+}) calc. for $C_{21}H_{32}O_4N$ [M+H]$^{+}$ 362.2324, found 362.2321

^{1}H NMR (600 MHz, Chloroform-*d*): δ 8.01 (d, *J* = 8.9 Hz, 2H, Ar), 7.97 (d, *J* = 8.9 Hz, 0.5H, Ar'), 6.93 (dq, *J* = 8.9, 2.5 Hz, 2.5H, Ar and Ar'), 4.96 (q, *J* = 6.4 Hz, 0.25H, 7'), 4.64 (q, *J* = 6.5 Hz, 1H, 7), 4.35 (s, 0.25H, 2'), 4.24 (sept, *J* = 6.7 Hz, 1H, 2), 3.87 (s, 3H, 10), 3.86 (s, 0.75H, 10'), 3.42 (br, 0.25H, 2'), 3.29 (br, 1H, 2), 1.87 (dd, *J* = 8.3, 5.4 Hz, 1H, 4), 1.75 (dd, *J* = 8.4, 5.4 Hz, 0.25H, 4'), 1.42 – 1.35 (m, 8.25H, 8, 8', 1, 1' and 1'), 1.33 (d, *J* = 6.7 Hz, 3H, 1), 1.27 (t, *J* = 5.2 Hz, 1H, 5b), 1.22 – 1.20 (m, 1H, 5b' and 1'), 1.17 – 1.15 (m, 3.75H, 10 and 1'), 1.14 (s, 1H, 10'), 0.96 (d, *J* = 6.7 Hz, 3H, 1), 0.94 (dd, *J* = 8.5, 3.7 Hz, 0.25H, 5a'), 0.80 (d, *J* = 6.6 Hz, 3H, 1), 0.77 (dd, *J* = 8.3, 4.9 Hz, 1H, 5a). Numbers denoted with a prime correspond to the minor diastereomer (epimer

at position 7). Integration of 0.25H for the minor diastereomer corresponds to 1H'. The diastereomer ratio was determined by ^{1}H integration of signals 2/2', 4/4' and 7/7'

^{13}C NMR (151 MHz, Chloroform-*d*): δ 168.7 (3'), 168.5 (3), 165.9 (9), 165.8 (9'), 163.6 (Ar), 163.6 (Ar'), 131.8 (Ar), 131.7 (Ar'), 123.0 (Ar'), 122.9 (Ar), 113.9 (Ar'), 113.8 (Ar), 77.6 (7), 74.8 (7'), 55.6 (11 and 11'), 48.7 (2'), 48.6 (2), 45.9 (2'), 45.8 (2), 29.5 (4), 27.4 (6), 27.2 (6'), 25.8 (4'), 21.3 (1'), 21.2 (1 and 1'), 21.1 (1), 20.9 (1'), 20.6 (1), 20.6 (1'), 20.5 (1), 17.5 (8'), 17.3 (8), 15.6 (5), 15.2 (5'), 14.0 (10'), 11.4 (10)

(*R)-1-((1*R**,2*R**)-2-(Diisopropylcarbamoyl)-1-methylcyclopropyl)ethyl 1-(2,2-difluorobenzo[d][1,3]dioxol-5-yl)cyclopropane-1-carboxylate, 3g**

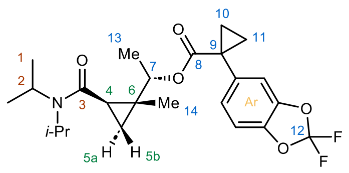

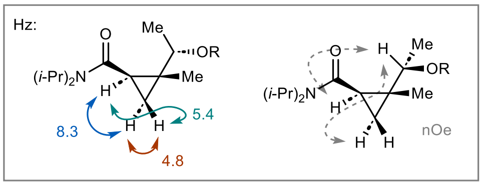

Prepared according to the general procedure using (2,2-Difluoro-benzo[1,3]dioxol-5-yl)-cyclopropanecarboxylicacid (58 mg, 0.24 mmol, 1.2 eq.). Upon completion, the reaction mixture was concentrated *in vacuo,* and the residue was purified *via* flash chromatography (25→60% Et_2O in pentane) to yield the title compound (75 mg, 0.17 mmol, *dr* 5:1, 83%) as a colourless oil.

R$_f$ = 0.43 (60% Et_2O in pentane)

IR (thin film, ν_{max} / cm^{-1}): 1718, 1637, 1504, 1443, 1241

HRMS (ESI$^+$) calc. for $C_{24}H_{42}O_5NF_2$ $[M+H]^+$ 452.2243, found 452.2234

^{1}H NMR (600 MHz, Chloroform-*d*): δ 7.06 – 7.00 (m, 2.4H, Ar and Ar'), 7.00 – 6.96 (m, 1.2H, Ar and Ar'), 4.64 (q, *J* = 6.3 Hz, 0.2H, 7'), 4.47 (q, *J* = 6.4 Hz, 1H, 7), 4.21 (sept, *J* = 6.7 Hz, 1.2H, 2 and 2'), 3.41 (br, 1.2H, 1 and 1'), 1.70 (dd, *J* = 8.3, 5.4 Hz, 1H, 4), 1.66 – 1.61 (m, 1H), 1.61 – 1.54 (m, 1.6H), 1.41 – 1.32 (m, 7.2H, 1 and 1'), 1.27 – 1.08 (m, 14.2H), 0.95 (s, 3H, 14), 0.93 (s, 0.6H, 14'), 0.67 (dd, *J* = 8.4, 4.6 Hz, 0.2H, 5a'), 0.62 (dd, *J* = 8.4, 4.8 Hz, 1H, 5a). Numbers denoted with a prime correspond to the minor diastereomer (epimer at position 7). Integration of 0.2H for the minor diastereomer corresponds to 1H'. The diastereomer ratio was determined by ^{1}H integration of signals 7/7', 4/4' and combined integration of signals 2/2' and Ar/Ar'. Complete assignment of all signals could not be performed due to the resolution of 2D data and overlapping diastereomer signals.

^{13}C NMR (151 MHz, Chloroform-*d*): 173.4 (8 and 8'), 168.5 (3 and 3'), 143.6 (Ar and Ar'), 143.0 (Ar and Ar'), 135.8 (Ar'), 135.7 (Ar), δ 131.83 (t, *J* = 255.3 Hz, 12 and 12'), 125.8 (Ar'), 125.6 (Ar), 112.0 (Ar and Ar'), 109.1 (Ar and Ar'), 77.1 (7), 75.2 (7'), 48.6 (2'), 48.4 (2), 45.9 (2 and 2'), 29.3 (9 and 9'), 28.0 (4), 27.2 (6), 26.9 (6'), 25.7 (4'), 21.5 (1), 21.3 (1 and 1'), 21.2 (1'), 20.9 (1 and 1'), 20.6 (1 and 1'), 17.4 (13'), 17.3 (13), 17.1 (10/11), 17.0 (10/11), 16.9 (10'/11'), 16.9 (10'/11'), 15.3 (5), 14.8 (5'), 13.4 (14'), 11.9 (14)

^{19}F NMR (565 MHz, Chloroform-*d*): δ -49.9 (d, *J* = 97.0 Hz, 12 and 12'), -50.1 (d, *J* = 97.0 Hz, 12 and 12')

(R^*)-1-((1R^*,2R^*)-2-(Diisopropylcarbamoyl)-1-methylcyclopropyl)ethyl (2,2,2-trifluoroacetyl) glycinate, 3h

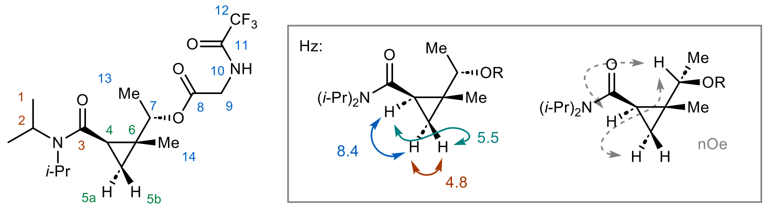

Prepared according to the general procedure using *N*-(trifluoroacetyl)glycine (41 mg, 0.24 mmol, 1.2 eq.). Upon completion, the reaction mixture was concentrated *in vacuo*, dry loaded onto silica and purified *via* flash chromatography (20→50% EtOAc in pentane) to yield the title compound (62 mg, 0.16 mmol, *dr* 20:1, 82%) as a colourless solid.

***R**$_f$* = 0.27 (40% EtOAc in pentane)

Recrystallization: hot Et_2O

IR (thin film, ν_{max} / cm^{-1}): 1754, 1728, 1619, 1184

HRMS (ESI^+) calc. for $C_{17}H_{28}O_4N_2F_3$ $[M+H]^+$ 381.1996, found 381.1994

^{1}H NMR (600 MHz, Chloroform-*d*): δ 6.90 (s, 1.05H, 10 and 10'), 4.75 (q, *J* = 6.4 Hz, 0.05H, 7'), 4.46 (q, *J* = 6.4 Hz, 1H, 7), 4.28 (sept, *J* = 6.7 Hz, 1.05H, 2 and 2'), 4.16 (dd, *J* = 18.3, 5.7 Hz, 1H, 9), 4.05 (d, *J* = 5.2 Hz, 0.1H, 9'), 4.05 (dd, *J* = 18.3, 4.5 Hz, 1H, 9), 3.41 (br, 1.05H, 2 and 2'), 1.78 (dd, *J* = 8.4, 5.5 Hz, 1H, 4), 1.72 (dd, *J* = 8.5, 5.5 Hz, 0.05H, 4'), 1.42 – 1.33 (m, 9.3H, 1, 1' and 13), 1.30 (d, *J* = 6.5 Hz, 0.15H, 13'), 1.28 (t, *J* = 5.2 Hz, 1H, 5b), 1.18 (dd, *J* = 12.7, 6.7 Hz, 6.35H, 1, 1, 5b'), 1.08 (s, 3.15H, 14 and 14'), 0.83 (dd, *J* = 8.4, 4.8 Hz, 0.05H, 5a'), 0.72 (dd, *J* = 8.4, 5.0 Hz, 1H, 5a). Numbers denoted with a prime correspond to the minor diastereomer (epimer at position 7). Integration of 0.05H for the minor diastereomer corresponds to 1H'. The diastereomer ratio was determined by ^{1}H integration of signals 2/2', 9/9', 7/7', 4/4' and combined integration of signals 2/2' and 14/14'.

^{13}C NMR (151 MHz, Chloroform-*d*): δ 168.0 (8), 167.9 (3), 157.3 (q, *J* = 37.9 Hz, 11), 115.7 (q, *J* = 287.4 Hz, 12), 80.6 (7), 78.4 (7'), 48.7 (2), 46.0 (2), 41.6, (9'), 41.5 (9), 29.0 (4), 26.8 (6), 26.6 (6'), 25.8 (4'), 21.2 (1, 1), 20.9 (1), 20.6 (1), 17.2 (14), 16.2 (14'), 15.6 (14), 13.2 (5'), 11.5 (5). Carbons of the minor diastereomer could not be fully assigned due to low-intensity/overlapping signals.

^{19}F NMR (565 MHz, Chloroform-*d*): δ -75.8 (12 and 12')

1-((*R)-1-((1*R**,2*R**)-2-(Diisopropylcarbamoyl)-1-methylcyclopropyl)ethyl) 3-methyl bicyclo [1.1.1]pentane-1,3-dicarboxylate, 3i**

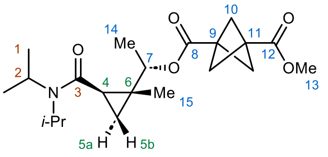

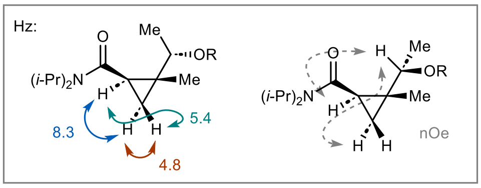

Prepared according to the general procedure using 3-(Methoxycarbonyl)bicyclo[1.1.1]pentane-1-carboxylic acid (41 mg, 0.24 mmol, 1.2 eq.). Upon completion, the reaction mixture was concentrated *in vacuo,* and the residue was purified *via* flash chromatography (20→60% Et_2O in pentane) to yield the title compound (60 mg, 0.16 mmol, *dr* 6.7:1, 79%) as a colourless oil.

R_f = 0.37 (50% Et_2O in pentane)

IR (thin film, ν_{max} / cm^{-1}): 1729, 1638, 1291, 1212

HRMS (ESI^+) calc. for $C_{21}H_{34}O_5N$ $[M+H]^+$ 380.2431, found 380.2437

^{1}H NMR (600 MHz, Chloroform-*d*): δ 4.72 (q, *J* = 6.4 Hz, 0.15H, 7'), 4.36 (q, *J* = 6.4 Hz, 1H, 7), 4.33 – 4.22 (m, 1.15H, 2 and 2'), 3.69 (s, 3H, 13), 3.69 (s, 0.45H, 13'), 3.42 (br, 1.15H, 2 and 2'), 2.31 (s, 6H, 10), 2.30 (s, 0.9H, 10'), 1.80 (dd, *J* = 8.3, 5.4 Hz, 1H, 4), 1.64 (dd, *J* = 8.4, 5.4 Hz, 0.15H, 4'), 1.44 – 1.33 (m, 6.9H, 1 and 1'), 1.28 (d, *J* = 6.5 Hz, 3H, 14), 1.27 – 1.24 (m, 1.6H, 5b, 5b' and 14'), 1.23 – 1.14 (m, 6.9H, 1 and 1'), 1.06 (s, 3H, 15), 1.04 (s, 0.45H, 15'), 0.81 (dd, *J* = 8.4, 4.7 Hz, 0.15H, 5a'), 0.67 (dd, *J* = 8.3, 4.8 Hz, 1H, 5a). Numbers denoted with a prime correspond to the minor diastereomer (epimer at position 7). Integration of 0.15H for the minor diastereomer corresponds to 1H'. The diastereomer ratio was determined by ^{1}H integration of signals 7/7', 4/4' and combined integration of signals 13/13'.

^{13}C NMR (151 MHz, Chloroform-*d*): δ 169.8 (12'), 169.7 (12), 169.0 (8), 168.9 (8'), 168.5 (3'), 168.3 (3), 78.0 (7), 74.8 (7'), 53.0 (10), 52.9 (10'), 52.0 (13), 52.0 (13'), 48.6 (2'), 48.4 (2), 45.9 (2 and 2'), 38.1 (9), 38.0 (9'), 37.7 (11'), 37.7 (11), 29.0 (4), 27.1 (6), 26.9 (6'), 25.6 (4'), 21.5 (1 and 1'), 21.3 (1 and 1'), 21.0 (1 and 1'), 20.6 (1 and 1'), 17.4 (14'), 17.3 (14), 15.5 (5), 14.9 (5'), 13.7 (15'), 11.5 (15)

(*R)-1-((1*R**,2*R**)-2-(Diisopropylcarbamoyl)-1-methylcyclopropyl)ethyl 2,4,6-triisopropylbenzoate, 3j**

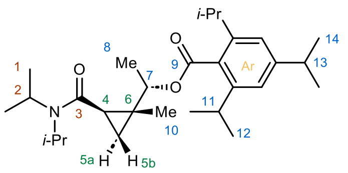

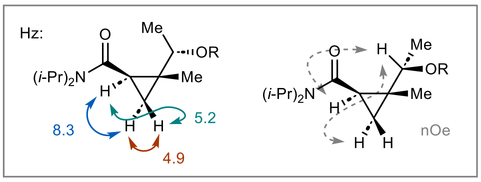

Prepared according to the general procedure using triisopropylbenzoic acid (60 mg, 0.24 mmol, 1.2 eq.). Upon completion, the reaction mixture was concentrated *in vacuo,* and the residue was purified *via* flash chromatography (10→30% EtOAc in pentane) to yield the title compound (70 mg, 0.15 mmol, *dr* 3:1, 76%) as a colourless oil.

R$_f$ = 0.47 (20% EtOAc in pentane)

IR (thin film, ν_{max} / cm^{-1}): 1722, 1639, 1439, 1251

HRMS (ESI^+) calc. for $C_{29}H_{48}O_3N$ $[M+H]^+$ 458.3629, found 458.3623

^{1}H NMR (600 MHz, Chloroform-*d*): δ 7.02 – 6.99 (m, 2.6H, Ar and Ar’), 4.90 (q, *J* = 6.4 Hz, 0.3H, 7’), 4.75 (q, *J* = 6.3 Hz, 1H, 7), 4.39 – 4.28 (m, 1.3H, 2 and 2’), 3.36 (br, 1.3H, 2 and 2’), 2.97 – 2.84 (m, 3.9H, 13, 13’, 11, 11, 11’ and 11’), 1.84 – 1.77 (m, 1.3H, 4 and 4’), 1.45 (d, *J* = 6.3 Hz, 3H, 8), 1.40 (d, *J* = 6.6 Hz, 0.9H, 1’), 1.39 – 1.36 (m, 4.2H, 1 and 1’, 8’ COSY), 1.34 (d, *J* = 6.9 Hz, 3H, 1), 1.28 – 1.23 (m, 23.7H, 14, 14, 14’, 14’, 12, 12, 12, 12, 12’, 12’, 12’ 12’ COSY and 5b’ HMBC), 1.21 (t, *J* = 5.1 Hz, 1H, 5b), 1.17 (d, *J* = 6.8 Hz, 0.9H, 1’), 1.13 (s, 3H, 10), 1.09 (s, 0.9H, 10’), 1.09 – 1.05 (m, 3.9H, 1 and 1’), 0.99 – 0.94 (m, 3.3H, 1 and 5a’), 0.84 (dd, *J* = 8.4, 4.7 Hz, 1H, 5a). Integration of 0.30H for the minor diastereomer corresponds to 1H’. The diastereomer ratio was determined by ^{1}H integration of signals 7/7’ and the combined integration of signals 4/4’ and 2/2’.

^{13}C NMR (151 MHz, Chloroform-*d*): δ 170.6 (9’), 170.4 (9), 168.6 (3), 168.3 (3’), 150.4 (Ar), 150.2 (Ar’), 145.2 (Ar), 144.7 (Ar’), 130.7 (Ar’), 130.2 (Ar), 121.0 (Ar), 120.9 (Ar’), 77.6 (7), 76.9 (7’), 48.8 (2’), 48.6 (2), 46.0 (2’), 45.9 (2), 34.6 (13’), 34.6 (13), 31.5 (11’), 31.5 (11), 26.8 (4), 26.7 (6’), 26.7 (6), 26.0 (4’), 24.8 (14), 24.5 (14’), 24.2 (12 and 12’), 24.1 (12’), 24.1 (12), 21.2 (1’), 21.2 (1), 21.0 (1 and 1’), 21.0 (1 and 1’), 20.7 (1 and 1’), 17.0 (8’), 16.9 (8), 16.3 (5’), 15.3 (5), 13.9 (10), 13.5 (10’)

(*R)-1-((1*R**,2*R**)-2-(Diisopropylcarbamoyl)-1-methylcyclopropyl)ethyl 2-(1-(4-chlorobenzoyl)-5-methoxy-1H-indol-3-yl)acetate, 3k**

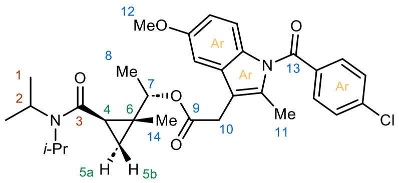

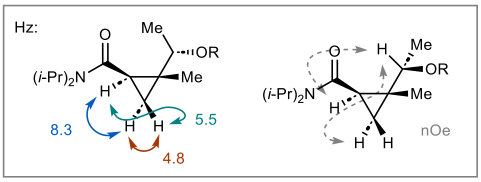

Prepared according to the general procedure using indomethacin (83 mg, 0.24 mmol, 1.2 eq.). Upon completion, the reaction mixture was concentrated *in vacuo,* and the residue was purified *via* flash chromatography (25→60% Et_2O in pentane) to yield the title compound (98 mg, 0.18 mmol, *dr* 5:1, 88%) as a light yellow-green solid.

***R**$_f$* = 0.35 (40% EtOAc in pentane)

IR (thin film, ν_{max} / cm^{-1}): 1733, 1685, 1634, 1478, 1318,

HRMS (ESI$^+$) calc. for $C_{32}H_{39}{}^{35}ClO_5N_2$ [M+H]$^+$ 567.2626, found 567.2618

^{1}H NMR (600 MHz, Chloroform-*d*): δ 7.65 (d, *J* = 8.4 Hz, 2.4H, Ar and Ar’), 7.47 (d, *J* = 8.5 Hz, 2.4H, Ar and Ar’), 6.94 (d, *J* = 2.5 Hz, 1.2H, Ar and Ar’), 6.86 (d, *J* = 8.9 Hz, 0.2H, Ar’), 6.85 (d, *J* = 9.0 Hz, 1.0H, Ar), 6.67 (dd, *J* = 9.0, 2.6 Hz, 1.2H, Ar and Ar’), 4.69 (q, *J* = 6.4 Hz, 0.2H, 7’), 4.46 (q, *J* = 6.4 Hz, 1H, 7), 4.37 (sept, *J* = 6.7 Hz, 1H, 2), 4.21 (sept, *J* = 6.7 Hz, 0.2H, 2’), 3.83 (s, 3.6H, 12 and 12’), 3.66 (d, *J* = 15.4 Hz, 1.2H, 10 and 10’), 3.62 (d, *J* = 15.4 Hz, 1.2H, 10 and 10’), 3.40 (br, 1.2H, 2 and 2’), 2.40 (s, 3H, 11), 2.39 (s, 0.6H, 11’), 1.77 (dd, *J* = 8.3, 5.5 Hz, 1H, 4), 1.58 – 1.54 (m, 0.2H, 4’), 1.42 – 1.33 (m, 7.2H, 1, 1, 1’ and 1’), 1.28 – 1.24 (m, 3.6H, 8 and 8’), 1.21 (t, *J* = 5.2 Hz, 1H, 5b), 1.20 – 1.15 (m, 6.6H, 1, 1 and 1’), 1.11 (t, *J* = 5.0 Hz, 0.2H, 5b’), 1.09 – 1.05 (m, 3.6H, 14, 1’), 1.01 (s, 0.6H, 14’), 0.75 (dd, *J* = 8.4, 4.7 Hz, 0.2H, 5a’), 0.67 (dd, *J* = 8.3, 4.8 Hz, 1H, 5a). Numbers denoted with a prime correspond to the minor diastereomer (epimer at position 7). Integration of 0.20H for the minor diastereomer corresponds to 1H’. The diastereomer ratio was determined by ^{1}H integration of signals 2/2’, 4/4’ and 7/7’

^{13}C NMR (151 MHz, Chloroform-*d*): δ 170.4 (9’), 170.4 (9), 168.5 (3’ or 13’), 168.4 (3 or 13), 168.3 (3 and 3’ or 13 and 13’), 156.2 (Ar’), 156.2 (Ar), 139.6 (Ar), 139.5 (Ar’), 136.0 (Ar), 135.9 (Ar’), 134.0 (Ar’), 133.9 (Ar), 131.3 (Ar), 131.2 (Ar’), 131.0 (Ar and Ar’), 130.7 (Ar and Ar’), 129.3 (Ar and Ar’), 115.1 (Ar and Ar’), 112.7 (Ar’), 112.5 (Ar), 111.9 (Ar’), 111.8 (Ar), 101.5 (Ar), 101.4 (Ar’), 77.8 (7), 75.4 (7’), 55.8 (12 and 12’), 48.7 (2’), 48.5 (2), 45.9 (2), 45.8 (2’), 30.8 (10’), 30.7 (10), 28.3 (4), 26.9 (6), 26.8 (6’), 25.7 (4’), 21.3 (1), 21.2 (1 and 1’), 21.2 (1’), 21.0 (1), 20.8 (1’), 20.6 (1 and 1’), 17.5 (11’), 17.2 (11), 15.3 (5), 15.1 (5’), 13.6 (8’), 13.5 (14), 13.4 (14’), 12.2 (8)

(1*R,2*R**)-2-((*S**)-1-fluoroethyl)-*N*,*N*-diisopropyl-2-methylcyclopropane-1-carboxamide, 3l**

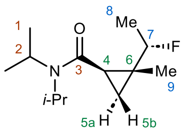

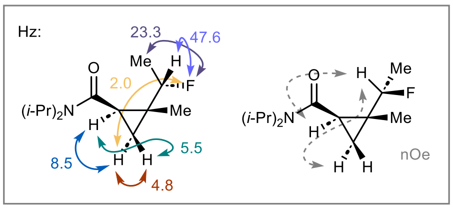

Prepared according to the general procedure using 70% HF in pyridine (x mg, 0.24 mmol, 1.2 eq.). Upon completion, the reaction mixture was concentrated *in vacuo,* and the residue was purified *via* flash chromatography (20→50% Et_2O in pentane) to yield the title compound (29 mg, 0.13 mmol, *dr* 5:1, 63%) as a colourless solid.

***R**$_f$* = 0.22 (40% Et_2O in pentane)

IR (thin film, ν_{max} / cm^{-1}): 1639, 1444, 1310

HRMS (ESI$^+$) calc. for $C_{13}H_{25}ONF$ [M+H]$^+$ 230.1915, found 230.1915

^{1}**H NMR** (600 MHz, Chloroform-*d*): δ 4.55 (sept, *J* = 7.0 Hz, 0H, 2), 4.47 (dq, *J* = 47.9, 6.3 Hz, 1H, 7'), 4.30 (sept, *J* = 6.8 Hz, 0H, 2'), 3.99 (dq, *J* = 47.6, 6.3 Hz, 1H, 7), 3.40 (br, 1H, 2 and 2'), 1.84 (dd, *J* = 8.4, 5.5 Hz, 0H, 4'), 1.79 (dd, *J* = 8.3, 5.6 Hz, 1H, 4), 1.43 – 1.40 (m, 5H, 1, 1' and 8), 1.39 (dd, *J* = 23.3, 6.3 Hz, 0H, 8), 1.38 – 1.35 (m, 5H, 1 and 1'), 1.30 (dd, *J* = 24.1, 6.3 Hz, 0H, 8'), 1.23 – 1.17 (m, 8H, 1, 1, 1', 1', 5b and 5b'), 1.06 (s, 1H, 9'), 1.04 (s, 3H, 9), 0.85 (dd, *J* = 8.5, 4.8 Hz, 0H, 5a'), 0.68 (ddd, *J* = 8.3, 4.9, 2.0 Hz, 1H, 5a). Numbers denoted with a prime correspond to the minor diastereomer (epimer at position 7). Integration of 0.20H for the minor diastereomer corresponds to 1H'. The diastereomer ratio was determined by ^{1}H integration of signals 7/7' and 4/4'.

^{13}**C NMR** (151 MHz, Chloroform-*d*): δ 168.7 (3'), 168.3 (3), 97.4 (d, *J* = 169.6 Hz, 7), 93.7 (d, *J* = 170.2 Hz, 7'), 48.6 (2 and 2'), 45.9 (2 and 2'), 28.7 (d, *J* = 1.9 Hz, 4), 26.9 (d, *J* = 21.4 Hz, 6'), 26.8 (d, *J* = 23.4 Hz, 6'), 24.4 (d, *J* = 6.5 Hz, 4'), 21.3 (1'), 21.2 (1 and 1'), 21.0 (1 and 1'), 20.9 (1), 20.7 (1'), 20.6 (1), 17.9 (d, *J* = 24.7 Hz, 8), 17.8 (d, *J* = 23.8 Hz, 8'), 14.5 (d, *J* = 8.4 Hz, 5 and 5'), 14.0 (d, *J* = 2.8 Hz, 9'), 11.0 (9 d, *J* = 1.3 Hz)

^{19}**F NMR** (565 MHz, Chloroform-*d*): δ -172.50 (dq, *J* = 47.3, 23.6 Hz), -175.2 (br)

(1*R,2*R**)-2-((*S**)-1-(2,4-Dinitrophenoxy)ethyl)-*N*,*N*-diisopropyl-2-methylcyclopropane-1-carboxamide, 3m**

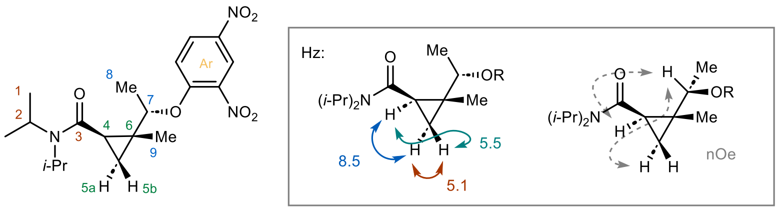

Prepared according to the general procedure using 2,4-dinitrophenol (44 mg, 0.24 mmol, 1.2 eq.). Upon completion, the reaction mixture was concentrated *in vacuo,* and the residue was purified *via* flash chromatography (10→80% EtOAc in pentane) to yield the title compound (53 mg, 0.13 mmol, 67%) as a colourless solid.

R$_f$ = 0.26 (80% EtOAc in pentane)

Recrystallization: hot Et_2O

IR (thin film, ν_{max} / cm^{-1}): 1607, 1536, 1344, 1282

HRMS (ESI$^+$) calc. for $C_{19}H_{28}O_6N_3$ [M+H]$^+$ 394.1973, found 394.1969

^{1}**H NMR** (600 MHz, Chloroform-*d*): δ 8.75 (d, *J* = 2.8 Hz, 1H, Ar), 8.42 (dd, *J* = 9.3, 2.8 Hz, 1H, Ar), 7.16 (d, *J* = 9.3 Hz, 1H, Ar), 4.31 (sept, *J* = 6.4 Hz, 1H, 2), 4.15 (q, *J* = 6.2 Hz, 1H, 7), 3.42 (br, 1H, 2), 1.83 (dd, *J* = 8.5, 5.5 Hz, 1H, 4), 1.49 (d, *J* = 6.1 Hz, 3H, 8), 1.39 (d, *J* = 6.7 Hz, 6H, 1 and 1), 1.35 (t, *J* = 5.3 Hz, 1H, 5b), 1.21 (s, 3H, 9), 1.15 (d, *J* = 6.6 Hz, 3H, 1), 1.08 (d, *J* = 6.7 Hz, 3H, 1), 0.80 (dd, *J* = 8.4, 5.1 Hz, 1H, 5a).

^{13}C NMR (151 MHz, Chloroform-*d*): δ 167.8 (3), 155.6 (Ar), 140.2 (Ar), 139.8 (Ar), 129.0 (Ar), 122.4 (Ar), 115.4 (Ar), 83.8 (7), 48.8 (2), 46.0 (2), 28.5 (4), 27.3 (6), 21.2 (1), 20.9 (1), 20.8 (1), 20.6 (1), 16.4 (8), 15.4 (5), 12.0 (9)

Single Crystal Data for **3m**: C19H27N3O6, Mr =393.44. 150 K – monoclinic, P 2_1/c, a = 21.2948(5) Å, b = 8.7337(2) Å, c = 11.3459(2) Å, β = 95.084(2)°, V = 2101.84(8) Å^3, Data/restraints/parameters – 4353/0/254, Rint = 0.045, Final R1 = 0.0470, wR2 = 0.1275 (I>2σ(I)).

Methyl ((*S)-1-((1*R**,2*R**)-2-(diisopropylcarbamoyl)-1-methylcyclopropyl)ethyl)(tosyl) carbamate, (*S**)-3n; Methyl ((*R**)-1-((1*R**,2*R**)-2-(diisopropylcarbamoyl)-1-methylcyclopropyl) ethyl)(tosyl) carbamate, (*R**)-3n**

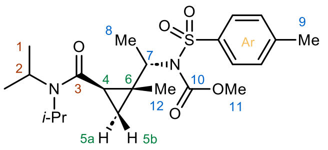

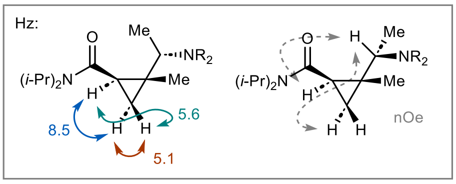

Prepared according to the general procedure using methyl tosylcarbamate (55 mg, 0.24 mmol, 1.2 eq.). Upon completion, the reaction mixture was concentrated *in vacuo,* and the residue was purified *via* flash chromatography (20→60% EtOAc in pentane) to yield the title compound (56 mg, 0.13 mmol, 64%) as a colourless solid and its epimer at position 7 (8 mg, 0.02 mmol, 9%) as a colourless solid.

(*S)-2p, *R$_f$*** = 0.40 (50% EtOAc in pentane)

Recrystallization: hot Et_2O:pentane, 1:4

IR (thin film, ν_{max} / cm^{-1}): 1736, 1632, 1440, 1303, 1214, 1172

HRMS (ESI$^+$) calc. for $C_{22}H_{35}O_5N_2S$ [M+H]$^+$ 439.2261, found 439.2243

^{1}H NMR (600 MHz, Chloroform-*d*): δ 7.80 (d, *J* = 8.3 Hz, 2H, Ar), 7.31 (d, *J* = 8.1 Hz, 2H, Ar), 4.49 – 4.39 (m, 2H, 2 and 7), 3.68 (s, 3H, 11), 3.42 (br, 1H, 2), 2.44 (s, 3H, 9), 1.96 (dd, *J* = 8.5, 5.6 Hz, 1H, 4), 1.42 (d, *J* = 6.8 Hz, 3H, 1), 1.38 (d, *J* = 6.9 Hz, 3H, 1), 1.36 (d, *J* = 6.9 Hz, 3H, 8), 1.27 – 1.20 (m, 7H, 1, 1 and 5b), 1.08 (s, 3H, 12), 0.98 (dd, *J* = 8.5, 5.1 Hz, 1H, 5a)

^{13}C NMR (151 MHz, Chloroform-*d*): δ 168.8 (3), 153.0 (11), 144.7 (Ar), 137.2 (Ar), 129.5 (Ar), 128.4 (Ar), 61.5 (7), 53.5 (11), 49.2 (2), 45.9 (2), 27.4 (4), 27.0 (6), 21.8 (9), 21.2 (1), 21.1 (1), 20.9 (1), 20.6 (1), 17.8 (5), 15.8 (8), 15.7 (12)

(*R)-2p**, ***R$_f$*** = 0.46 (50% EtOAc in pentane)

IR (thin film, ν_{max} / cm^{-1}): 1736, 1633, 1440, 1358, 1215, 1173

HRMS (ESI$^+$) calc. for $C_{22}H_{35}O_5N_2S$ [M+H]$^+$ 439.2261, found 439.2243

^{1}H NMR (600 MHz, Chloroform-*d*): δ 7.81 – 7.78 (m, 2H, Ar), 7.31 (d, *J* = 8.1 Hz, 2H, Ar), 4.59 (q, *J* = 7.2 Hz, 1H, 7), 4.53 (sept, *J* = 6.6 Hz, 1H, 2), 3.64 (s, 3H, 11), 3.40 (br, 1H, 2), 2.44 (s, 3H, 9), 1.92 (dd, *J* = 8.6, 5.7 Hz, 1H, 4), 1.51 (d, *J* = 7.2 Hz, 3H, 8), 1.41 (d, *J* = 6.8 Hz, 3H, 1), 1.37 (d, *J* = 6.7 Hz, 3H, 1), 1.24 – 1.18 (m, 7H, 1, 1 and 5b), 1.13 (s, 3H, 12), 1.03 (dd, *J* = 8.6, 4.9 Hz, 1H, 5a).

^{13}C NMR (151 MHz, Chloroform-*d*): δ 168.7 (3), 152.9 (Ar), 144.6 (Ar), 137.4 (Ar), 129.5 (Ar), 128.3 (Ar), 61.1 (7), 53.4 (11), 48.7 (2), 46.0 (2), 28.7 (4), 27.8 (6), 21.8 (9), 21.3 (1), 21.2 (1), 21.0 (1), 20.7 (1), 17.1 (8), 16.2 (5), 15.9 (12)

Single Crystal Data for **3n**: C22H34N2O5S, Mr =438.59. 150 K – monoclinic, P 2_1/c, a = 14.6520(4) Å, b = 10.4813(2) Å, c = 16.6855(4) Å, β = 114.474(3)°, V = 2332.19(11) Å^3, Data/restraints/parameters – 6194/0/271, Rint = 0.042, Final R1 = 0.0409, wR2 = 0.0913 (I>2σ(I)).

(1*R,2*R**)-2-((*S**)-1-(2*H*-Tetrazol-2-yl)ethyl)-*N*,*N*-diisopropyl-2-methylcyclopropane-1-carboxamide, 3o**

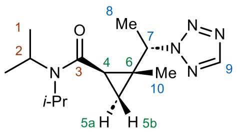

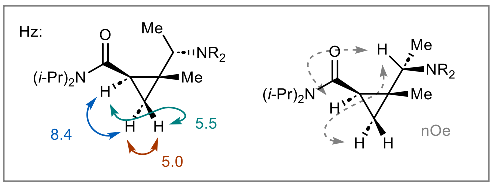

Prepared according to the general procedure using tetrazole (17 mg, 0.24 mmol, 1.2 eq.). Upon completion, the reaction mixture was concentrated *in vacuo,* and the residue was purified *via* flash chromatography (20→60% EtOAc in pentane) to yield the title compound (35 mg, 0.12 mmol, 63%) as a colourless oil.

R$_f$ = 0.39 (50% EtOAc in pentane)

IR (thin film, ν_{max} / cm^{-1}): 1635, 1447, 1302

HRMS (ESI$^+$) calc. for $C_{14}H_{26}ON_5$ [M+H]$^+$ 280.2132, found 280.2131

^{1}H NMR (600 MHz, Chloroform-*d*): δ 8.52 (s, 1H, 9), 8.50 (s, 0.15H, 9'), 4.78 (q, *J* = 7.0 Hz, 0.15H, 7'), 4.36 (q, *J* = 7.1 Hz, 1.15H, 2' and 7), 3.61 (sept, *J* = 6.7 Hz, 1H, 2), 3.40 (br, 0.15H, 2'), 3.25 (sept, *J* = 6.8 Hz, 1H, 2), 2.05 (dd, *J* = 8.4, 5.5 Hz, 1H, 4), 1.81 (d, *J* = 7.1 Hz, 3H, 8), 1.72 (d, *J* = 6.9 Hz, 0.6H, 4' and 8'), 1.44 (t, *J* = 5.3 Hz, 1H, 5b), 1.39 (d, *J* = 6.8 Hz, 0.45H, 1'), 1.35 (d, *J* = 6.8 Hz, 3.45H, 1 and 1'), 1.26 (d, *J* = 6.7 Hz, 3.15H, 1 and 5b'), 1.22 (d, *J* = 6.7 Hz, 0.45H, 1'), 1.18 (d, *J* = 6.7 Hz, 0.45H, 1'), 1.07 (d, *J* = 6.7 Hz, 3.15H, 1 and 5a'), 1.06 (s, 3.45H, 10 and 10'), 0.88 (dd, *J* = 8.4, 5.0 Hz, 1H, 5a), 0.73 (d, *J* = 6.6 Hz, 3H, 1). Numbers denoted with a prime correspond to the minor diastereomer (epimer at position 7). Integration of 0.17H for the minor diastereomer corresponds to 1H'. The diastereomer ratio was determined by ^{1}H integration of signals 2/2', 7/7' and 9/9'.

^{13}C NMR (151 MHz, Chloroform-*d*): δ 167.5 (3'), 167.3 (3), 152.6 (9 and 9), 68.7 (7), 66.6 (7'), 48.8 (2'), 48.6 (2), 46.1 (2'), 45.9 (2), 28.9 (4), 28.0 (6), 27.4 (6'), 26.6 (4'), 21.3 (1'), 21.1 (1'), 21.0 (1), 20.9 (1 and 1'), 20.7 (1), 20.6 (1'), 20.5 (1), 17.4 (5), 16.7 (8), 16.6 (8'), 16.0 (5'), 14.0 (10'), 11.3 (10)

(1*R,2*R**)-*N*,*N*-propyl-2-methyl-2-((*S**)-1-((trifluoromethyl)sulfonamido)ethyl)cyclopropane-1-carboxamide, 3p**

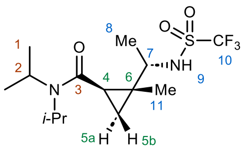

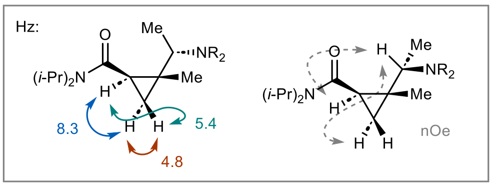

Prepared according to the general procedure using trifluoromethanesulfonamide (36 mg, 0.24 mmol, 1.2 eq.). Upon completion, the reaction mixture was concentrated *in vacuo,* and the residue was purified *via* flash chromatography (10→60% EtOAc in pentane) to yield the title compound (51 mg, 0.14 mmol, 71%) as a colourless solid.

***R**$_f$* = 0.25 (30% EtOAc in pentane)

Recrystallization: hot Et_2O

IR (thin film, ν_{max} / cm^{-1}): 1608, 1449, 1371, 1192

HRMS (ESI$^+$) calc. for $C_{14}H_{26}O_3N_2F_3S$ [M+H]$^+$ 359.1611, found 359.1608

^{1}H NMR (600 MHz, Chloroform-*d*): δ 5.29 (br, 1.13H, 9 and 9'), 4.58 (sept, *J* = 6.7 Hz, 1H, 2), 4.38 – 4.30 (sept, *J* = 6.6 Hz, 0.13H, 2'), 3.41 (d, *J* = 6.7 Hz, 1.13H, 2 and 2'), 3.34 (q, *J* = 6.9 Hz, 0.13H, 7'), 3.09 – 2.96 (m, 1H, 7), 1.97 (dd, *J* = 8.4, 5.4 Hz, 1H, 4), 1.82 (dd, *J* = 8.6, 5.4 Hz, 0.13H, 4'), 1.41 (d, *J* = 6.8 Hz, 3.39H, 1 and 1'), 1.37 (d, *J* = 6.9 Hz, 3.78H, 1, 1' and 8'), 1.36 (d, *J* = 6.9 Hz, 3H, 8), 1.32 (t, *J* = 5.2 Hz, 1H, 5b), 1.22 (d, *J* = 6.7 Hz, 3.39H, 1 and 1'), 1.20 (d, *J* = 6.7 Hz, 3.52H, 1, 1' and 5b'), 1.05 (s, 0.39H, 11'), 1.04 (s, 3H, 11), 0.87 (dd, *J* = 8.5, 5.3 Hz, 0.13H, 5a'), 0.70 (dd, *J* = 8.4, 5.0 Hz, 1H, 5a). Numbers denoted with a prime correspond to the minor diastereomer (epimer at position 7). Integration of 0.13H for the minor diastereomer corresponds to 1H'. The diastereomer ratio was determined by ^{1}H integration of signals 2/2', 7/7' 10/10' and combined integration of 9/9'.

^{13}C NMR (151 MHz, Chloroform-*d*): δ 168.1 (3'), 168.0 (3), 119.83 (q, *J* = 321.1 Hz, 10 and 10'), 60.8 (7), 58.4 (7'), 49.1 (2), 48.9 (2'), 46.0 (2), 45.9 (2'), 29.3 (4), 28.4 (6), 27.7 (6'), 27.1 (4'), 21.2 (1), 21.1 (1'), 21.0 (1), 20.9 (1'), 20.8 (1'), 20.7 (1), 20.6 (1'), 20.5 (1), 19.4 (8'), 18.8 (8), 17.1 (5'), 15.0 (5), 13.4 (11'), 10.9 (11)

^{19}F NMR (377 MHz, Chloroform-*d*): δ -77.4 (10'), -77.5 (10)

Single Crystal Data for **3p**: C14H25F3N2O3S, Mr =358.42. 150 K – monoclinic, Cc, a = 11.9581(3) Å, b = 14.3089(3) Å, c = 10.9176(3) Å, β = 101.375(3)°, V = 1831.39(8) Å^3, Data/restraints/parameters – 3281/230/223, Rint = 0.041, Final R1 = 0.0370, wR2 = 0.0960 (I>2σ(I)).

(*S)-1-((1*R**,2*R**)-2-(propylcarbamoyl)-1-methylcyclopropyl)butyl 2,2,2-trifluoroacetate, 3q**

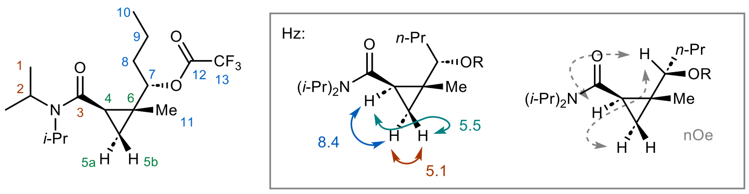

Prepared according to the general procedure using trifluoroacetic acid (19 µL, 0.24 mmol, 1.2 eq.). Upon completion, the reaction mixture was concentrated *in vacuo,* and the residue was purified *via* flash chromatography (10→40% Et_2O in pentane) to yield the title compound (56 mg, 0.16 mmol, *dr* 5:1, 79%) as a colourless solid.

Rf = 0.30 (40% Et_2O in pentane)

IR (thin film, ν_{max} / cm^{-1}): 1754, 1728, 1619, 1207

HRMS (ESI^+) calc. for $C_{17}H_{29}O_3NF_3$ $[M+H]^+$ 352.2094, found 352.2098

^{1}H NMR (600 MHz, Chloroform-*d*): δ 4.85 (dd, *J* = 10.2, 2.9 Hz, 0.15H, 7’), 4.41 (dd, *J* = 8.8, 5.1 Hz, 1H, 7), 4.26 (sept, *J* = 6.4 Hz, 0.15H, 2’), 4.15 (sept, *J* = 6.4 Hz, 1H, 2), 3.38 (br, 1.15H, 2 and 2’), 1.92 – 1.84 (m, 1.15H, 8 and 8’), 1.81 (dd, *J* = 8.4, 5.5 Hz, 1H, 4), 1.79 – 1.69 (m, 1.3H, 8, 8’ and 4’), 1.42 – 1.30 (m, 11.3H, 9, 8, 8’, 8’, 5b, 1 and 1), 1.27 – 1.18 (m, 1.75H, 7’, 7’, 5b’, 1’, 1’, 1’ and 1’), 1.14 (d, *J* = 6.7 Hz, 6H, 1 and 1), 1.09 (s, 3.45H, 11 and 11’), 0.94 (t, *J* = 7.3 Hz, 3.45H, 10 and 10’), 0.91 (dd, *J* = 8.6, 5.0 Hz, 0.15H, 5a’), 0.77 (dd, *J* = 8.4, 5.1 Hz, 1H, 5a). Numbers denoted with a prime correspond to the minor diastereomer (epimer at position 7). Integration of 0.15H for the minor diastereomer corresponds to 1H’. The diastereomer ratio was determined by ^{1}H integration of signals 2/2’ and 7/7’, and ^{19}F integration of CF_3.

^{13}C NMR (151 MHz, Chloroform-*d*): δ 167.7 (3’), 167.6 (3), 157.71 (q, *J* = 41.8 Hz, 12 and 12’), 114.87 (q, *J* = 286.2 Hz, 13 and 13’), 87.3 (7), 84.0 (7’), 48.8 (2 and 2’), 46.1 (2’), 46.0 (2), 33.9 (8), 33.6 (8’), 28.6 (4), 26.3 (6), 26.1 (6), 25.5 (4’), 21.2 (1, 1’ and 1’), 20.9 (1’), 20.7 (1 and 1), 20.6 (1’), 20.5 (1), 19.1 (9), 19.0 (9’), 16.3 (5), 15.9 (5’), 13.9 (10 and 10’), 13.8 (11’), 11.1 (11).

^{19}F NMR (565MHz, Chloroform-*d*): δ -75.0 (13), -74.8 (13’)

(*S)-1-((1*R**,2*R**)-2-(propylcarbamoyl)-1-methylcyclopropyl)-2-phenylethyl 2,2,2-trifluoroacetate, 3r**

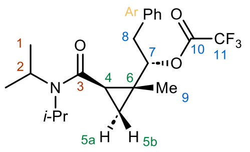

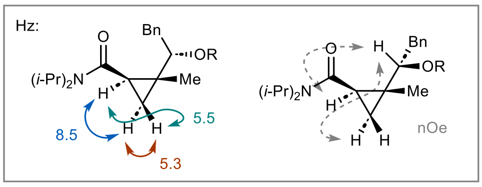

Prepared according to the general procedure using trifluoroacetic acid (19 µL, 0.24 mmol, 1.2 eq.). Upon completion, the reaction mixture was concentrated *in vacuo,* and the residue was purified *via* flash chromatography (10→40% Et_2O in pentane) to yield the title compound (53 mg, 0.13 mmol, *dr* 5:1, 89%) as a colourless solid.

R_f = 0.34 (50% Et_2O in pentane)

IR (thin film, ν_{max} / cm^{-1}): 1718, 1637, 1241

HRMS (ESI^+) calc. for $C_{21}H_{29}O_3NF_3$ $[M+H]^+$ 400.2094, found 400.2089

^{1}H NMR (600 MHz, Chloroform-*d*): δ 7.31 – 7.27 (m, 2.45H, Ar and Ar’), 7.25 – 7.22 (m, 1.15H, Ar and Ar’), 7.19 – 7.15 (m, 2.15H, Ar and Ar’), 5.03 (dd, *J* = 10.2, 3.2 Hz, 0.15H, 7’), 4.58 (dd, *J* = 9.2, 4.5 Hz, 1H, 7), 4.26 (sept, *J* = 6.5 Hz, 0.15H, 2’), 4.10 (sept, *J* = 6.7 Hz, 1H, 2), 3.44 (br, 0.15H, 2’), 3.39 (sept, *J* = 6.2 Hz, 1H, 2), 3.11 (dd, *J* = 14.1, 9.2 Hz, 1H, 8), 3.06 (dd, *J* = 14.1, 4.6 Hz, 1H, 8), 3.01 (dd, *J* = 14.4, 3.3 Hz, 0.15H, 8’), 2.95 (dd, *J* = 14.3, 10.2 Hz, 0.15H, 8’), 1.84 (dd, *J* = 8.5, 5.5 Hz, 1H, 4), 1.80 (dd, *J* = 8.7, 5.6 Hz, 0.15H, 4’), 1.41 (d, *J* = 7.7 Hz, 0.45H, 1’), 1.39 (d, *J* = 7.1 Hz, 3.45H, 1 and 1’), 1.37 (d, *J* = 6.6 Hz, 4H, 1, 5b), 1.30 (t, *J* = 5.3 Hz, 0.15H, 5b’), 1.26 – 1.22 (m, 3.45H, 1’ and 1’), 1.21 (s, 3H, 9), 1.20 (s, 0.45H, 9’), 1.13 (d, *J* = 7.2 Hz, 3H, 1), 1.12 (d, *J* = 7.1 Hz, 3H, 1), 0.98 (dd, *J* = 8.7, 5.1 Hz, 0.15H, 5a’), 0.71 (dd, *J* = 8.5, 5.3 Hz, 1H, 5a). Numbers denoted with a prime correspond to the minor diastereomer (epimer at position 7). Integration of 0.15H for the minor diastereomer corresponds to 1H’. The diastereomer ratio was determined by ^{1}H integration of signals 2/2’, 4/4’ and 7/7’, 8/8’ and ^{19}F integration of signals 11/11’.

^{13}C NMR (151 MHz, Chloroform-*d*): δ 167.6 (3’), 167.4 (3), 157.23 (q, *J* = 42.0 Hz, 10 and 10’), 136.2 (Ar), 136.0 (Ar’), 129.2 (Ar), 129.1 (Ar’), 128.9 (Ar and Ar’), 127.3 (Ar’), 127.3 (Ar), 114.70 (q, *J* = 286.1 Hz, 11 and 11’).87.9 (7), 84.2 (7’), 48.8 (2 and 2’), 46.1 (2), 46.0 (2’), 38.2 (8), 38.1 (8’), 29.0 (4), 26.3 (6’), 26.0 (6), 25.6 (4’), 21.3 (1’), 21.2 (1), 20.9 (1’), 20.7 (1 and 1), 20.6 (1’), 20.5 (1), 16.2 (5), 15.8 (5’), 14.0 (9’), 11.3 (9)

^{19}F NMR (565 MHz, Chloroform-*d*): δ -75.0 (11’), -74.7 (11)

(R*)-((1R*,2R*)-2-(propylcarbamoyl)-1-methylcyclopropyl)(phenyl)methyl 2,2,2-trifluoroacetate, 3s

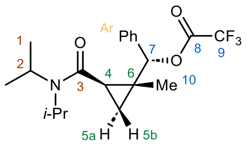

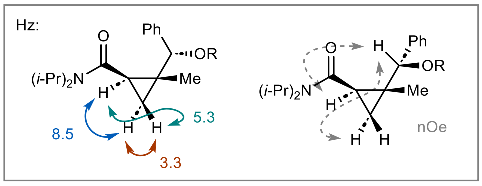

Prepared according to the general procedure using trifluoroacetic acid (19 µL, 0.24 mmol, 1.2 eq.). Upon completion, the reaction mixture was concentrated *in vacuo,* and the residue was purified *via* flash chromatography (10→40% Et_2O in pentane) to yield the title compound (69 mg, 0.18 mmol, *dr* 4:1, 89%) as a colourless oil.

R_f = 0.30 (40% Et_2O in pentane)

IR (thin film, ν_{max} / cm^{-1}): 1786, 1637, 1221, 1156

HRMS (ESI^+) calc. for $C_{20}H_{27}O_3NF_3$ $[M+H]^+$ 386.1938, found 386.1936

^{1}H NMR (400 MHz, Chloroform-*d*): δ 7.43 – 7.26 (m, 6.25H, Ar and Ar), 5.69 (s, 0.25H, 7'), 5.43 (s, 1H, 7), 4.29 (sept, J = 6.3 Hz, 1H, 2), 4.01 (sept, J = 6.8 Hz, 0.25H, 2'), 3.41 (sept, J = 6.7 Hz, 1H, 2), 3.32 (sept, J = 6.7 Hz, 0.25H, 2'), 1.90 (dd, J = 8.5, 5.6 Hz, 1H, 4), 1.78 (dd, J = 8.6, 5.6 Hz, 0.25H, 4), 1.40 (d, J = 6.8 Hz, 3H, 1), 1.39 – 1.34 (m, 4.25H, 5b, 5b' and 1), 1.31 (d, J = 6.7 Hz, 0.75H, 1'), 1.19 (d, J = 4.5 Hz, 3H, 1), 1.17 (d, J = 4.6 Hz, 3H, 1), 1.15 (s, 0.75H, 10'), 1.11 (d, J = 7.0 Hz, 0.75H, 1'), 1.08 (dd, J = 5.3, 3.3 Hz, 1H, 5a), 1.04 (s, 3H, 10), 0.89 – 0.80 (m, 1H, 1' and 5a'). Numbers denoted with a prime correspond to the minor diastereomer (epimer at position 7). Integration of 0.25H for the minor diastereomer corresponds to 1H'. The diastereomer ratio was determined by ^{1}H integration of signals 2/2', 4/4' and 7/7', and ^{19}F integration of signals 9/9'.

^{13}C NMR (101 MHz, Chloroform-*d*): δ 167.5 (3), 136.2 (Ar), 129.3 (Ar'), 128.9 (Ar), 128.8 (Ar'), 128.7 (Ar), 127.1 (Ar'), 126.0 (Ar), 87.0 (7), 85.5 (7'), 49.0 (2 and 2'), 46.0 (2), 45.9 (2'), 28.41 (4), 27.4 (6), 25.1 (4'), 21.2 (1), 21.1 (1'), 20.8 (1), 20.7 (1), 20.6 (1'), 20.6 (1'), 20.5 (1), 20.5 (1'), 16.2 (5'), 16.0 (5), 14.1 (10'), 12.4 (10). Peaks 9/9', 8/8' and 6' could not be located due to signal intensity.

^{19}F NMR (377 MHz, Chloroform-*d*): δ -75.1 (9), -75.0 (9')

Methyl (1*R,2*R**)-2-((*R**)-phenyl(2,2,2-trifluoroacetoxy)methyl)cyclopropane-1-carboxylate, 3t**

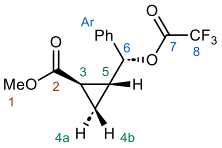

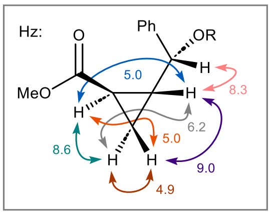

Prepared according to the general procedure using trifluoroacetic acid (19 µL, 0.24 mmol, 1.2 eq.). Upon completion, the reaction mixture was concentrated *in vacuo* to yield the title compound (60 mg, 0.20 mmol, *dr* 1:1, 98%) as a colourless solid.

Rf = 0.53 (30% Et_2O in pentane)

IR (thin film, ν_{max} / cm^{-1}): 1729, 1209, 1173, 701

HRMS (ESI^+) calc. for $C_{14}H_{26}O_3N_2F_3S$ $[M+H]^+$ 303.0682, found 303.0839

^{1}H NMR (600 MHz, Chloroform-*d*): δ 7.43 – 7.34 (m, 9H, Ar and Ar'), 5.60 (d, *J* = 7.6 Hz, 0.8H, 6'), 5.49 (d, *J* = 8.3 Hz, 1H, 6), 3.71 (s, 3H, 1), 3.65 (s, 2.4H, 1'), 2.13 – 2.05 (m, 1.8H, 3 and 3'), 1.87 (ddd, *J* = 9.0, 5.1, 4.1 Hz, 1H, 5), 1.73 (ddd, *J* = 9.0, 5.0, 4.2 Hz, 0.8H, 5'), 1.36 (dt, *J* = 8.7, 4.9 Hz, 0.8H, 4b'), 1.31 (dt, *J* = 9.0, 5.0 Hz, 1H, 4b), 1.13 (ddd, *J* = 8.7, 6.3, 4.8 Hz, 0.8H, 4a'), 1.00 (ddd, *J* = 8.6, 6.2, 4.9 Hz, 1H, 4a). Numbers denoted with a prime correspond to the minor diastereomer (epimer at position 6). Integration of 0.8H for the minor diastereomer corresponds to 1H'. The diastereomer ratio was determined by ^{1}H integration of signals 1/1', 4/4' and 6/6'.

^{13}C NMR (151 MHz, Chloroform-*d*): δ 173.2 (2), 173.1 (2'), 156.9 (q, *J* = 42.6 Hz, 7), 156.4 (q, *J* = 42.5 Hz, 7'), 136.6 (Ar), 136.5 (Ar'), 129.5 (Ar), 129.4 (Ar'), 129.1 (Ar and Ar'), 126.8 (Ar), 126.7 (Ar'), 114.7 (q, *J* = 285.9 Hz, 8 and 8'), 81.8 (6), 81.3 (6'), 52.3 (1), 52.2 (1'), 26.0 (3), 25.7 (3'), 19.4 (5), 18.1 (5'), 13.6 (4'), 12.6 (4)

^{19}F NMR (565 MHz, Chloroform-*d*): δ -75.1 (8 and 8')

((1*S,2*S**)-2-(propylcarbamoyl)-1-(trimethylsilyl)cyclopropyl)(trimethylsilyl)methyl 2,2,2-trifluoroacetate, 3u**

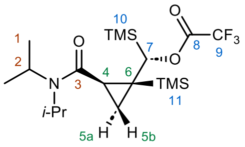

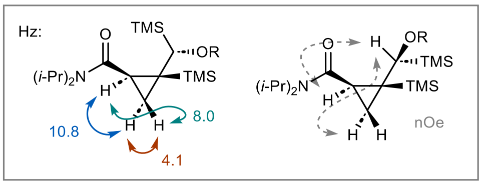

Prepared according to the general procedure using trifluoroacetic acid (19 µL, 0.24 mmol, 1.2 eq.). Upon completion, the reaction mixture was concentrated *in vacuo,* and the residue was purified *via* flash chromatography (5→15% Et_2O in pentane) to yield the title compound (33 mg, 0.15 mmol, 74%) as a colourless oil.

Rf = 0.74 (10% Et_2O in pentane)

IR (thin film, ν_{max} / cm^{-1}): 1775, 1632, 1149, 847

HRMS (ESI^+) calc. for $C_{19}H_{37}O_3NF_3Si_2$ $[M+H]^+$ 440.2259, found 440.2261

^{1}H NMR (600 MHz, Chloroform-*d*): δ 4.57 (sept, *J* = 6.7 Hz, 1H, 2), 4.46 (s, 1H, 7), 3.36 (sept, *J* = 6.9 Hz, 1H, 2), 1.44 (d, *J* = 6.8 Hz, 3H, 1), 1.36 (d, *J* = 6.8 Hz, 3H, 1), 1.21 (d, *J* = 6.5 Hz, 3H, 1), 1.11 (d, *J* = 6.6 Hz, 3H, 1), 1.06 (dd, *J* = 10.8, 4.1 Hz, 1H, 5a), 0.92 (dd, *J* = 8.0, 4.1 Hz, 1H, 5b), 0.15 (s, 9H, 10), 0.07 – 0.01 (m, 10H, 4 and 11)

^{13}C NMR (151 MHz, Chloroform-*d*): δ 169.1 (3), 158.4 (q, *J* = 41.6 Hz, 8), 115.0 (q, *J* = 286.1 Hz, 9), 87.3 (7), 49.1 (2), 46.4 (2), 33.2 (6), 21.2 (1), 21.1 (1), 20.9 (1), 20.8 (1), 16.8 (5), 15.5 (4), -1.1 (11), -2.6 (10)

^{19}F NMR (565 MHz, Chloroform-*d*): δ -74.3 (9)

O-((*S**)-1-((1*R**,2*R**)-2-(propylcarbamoyl)-1-methylcyclopropyl)ethyl) ethanethioate, 3v

S-((1*R**,2*R**,3*S**)-3-(propylcarbamoyl)-1,2-dimethylcyclobutyl) ethanethioate, 3w

S-((*S**)-1-((1*R**,2*R**)-2-(propylcarbamoyl)-1-methylcyclopropyl)ethyl) ethanethioate, S4

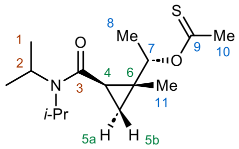

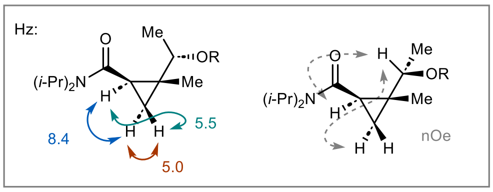

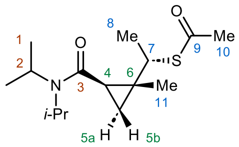

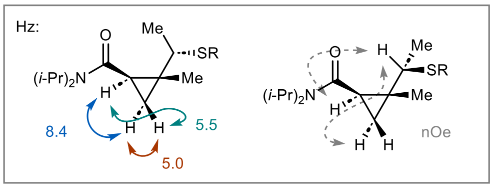

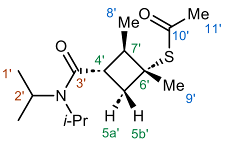

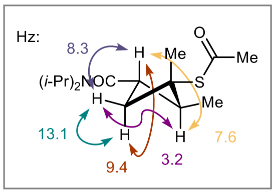

Prepared according to the general procedure using thioacetic acid (17 µL, 0.24 mmol, 1.2 eq.). Upon completion, the reaction mixture was concentrated *in vacuo,* and the residue was purified *via* flash chromatography (5→50% Et_2O in pentane) to yield the title compounds **3v** and **S4** (18 mg, 0.06 mmol, 32%) as a colourless oil and **3w** (23 mg, 0.08 mmol, 40%) as a colourless oil.

3v and S4, R_f = 0.27 (30% Et_2O in pentane)

IR (thin film, ν_{max} / cm^{-1}): 1641, 1440, 1270

HRMS (ESI^+) calc. for $C_{15}H_{28}O_2NS$ $[M+H]^+$ 286.18635, found 286.1834

^{1}**H NMR** (600 MHz, Chloroform-*d*): δ 5.10 (q, J = 6.4 Hz, 1H, 7), 4.32 (sept, J = 6.8 Hz, 1H, 2), 3.77 (sept, J = 6.4 Hz, 0H, 2'), 3.49 – 3.44 (m, 0.14H, 4'), 3.39 (br, 3.42H, 2 and 2'), 2.82 (pd, *J* = 7.6, 3.1 Hz, 0.14H, 7'), 2.74 (dd, J = 13.1, 9.4 Hz, 0.14H, 5b'), 2.57 (s, 3H, 10), 2.29 (s, 0.42H, 11'), 2.02 (ddd, *J* = 12.8, 8.3, 3.2 Hz, 0.14H, 5a'), 1.82 (dd, J = 8.3, 5.5 Hz, 1H, 4), 1.45 (s, 0.42H, 9'), 1.39 (d, *J* = 6.7 Hz, 3.42H, 1 and 1'), 1.37 (d, *J* = 6.6 Hz, 6.42H, 1, 1' and 8), 1.27 (t, J = 5.2 Hz, 1H, 5b), 1.19 (d, J = 6.7 Hz, 3.42H, 1 and 1'), 1.14 (d, *J* = 6.9 Hz, 3.42H, 1 and 1'), 1.13 (s, 3H, 11), 1.08 (d, J = 7.4 Hz, 0.42H, 8'), 0.76 (dd, J = 8.3, 5.0 Hz, 1H, 5a). Numbers denoted with a prime correspond to **S4**. Integration of 0.14H for the minor diastereomer corresponds to 1H'. The ratio was determined by ^{1}H integration of signals 2/2', 10/11' and combined integration of 4/5a'.

^{13}**C NMR** (151 MHz, Chloroform-*d*): δ 219.0 (9, HMBC), 196.9 (10', HMBC), 170.4 (3'), 168.2 (3), 85.6 (7), 50.0 (6'), 48.7 (2), 48.2 (2'), 45.9 (2), 45.8 (2'), 43.4 (7'), 38.2 (4'), 35.1 (10), 33.2 (5'), 31.2 (11'), 29.2 (4), 27.1 (6), 22.9 (9'), 21.4 (1'), 21.3 (1), 21.2 (1), 21.0 (1'), 20.9 (1), 20.8 (1'), 20.7 (1'), 20.6 (1), 16.2 (8), 15.5 (5), 11.9 (8'), 11.7 (11)

3w, $\boldsymbol{R_f}$ = 0.35 (30% Et_2O in pentane)

IR (thin film, ν_{max} / cm^{-1}): 1725, 1642, 1470, 1305

HRMS (ESI^+) calc. for $C_{15}H_{28}O_2NS$ $[M+H]^+$ 286.18635, found 286.1833

^{1}**H NMR** (600 MHz, Chloroform-*d*): 4.42 (sept, *J* = 6.7 Hz, 1.08H, 2 and 2'), 3.35 (sept, *J* = 7.1 Hz, 1.08H, 2 and 2'), 3.26 (q, *J* = 7.1 Hz, 0.09H, 7'), 3.14 (q, *J* = 7.2 Hz, 1H, 7), 2.33 (s, 3.24H, 10 and 10'), 1.82 (dd, *J* = 8.3, 5.5 Hz, 1H, 4), 1.79 (dd, *J* = 8.4, 5.5 Hz, 0.08H, 4'), 1.39 (d, *J* = 6.8 Hz, 3H, 1 and 1'), 1.37 (d, *J* = 7.2 Hz, 3H, 8 and 8'), 1.36 (d, *J* = 6.5 Hz, 2H, 1, 1'), 1.30 (t, *J* = 5.2 Hz, 3H, 5b), 1.26 – 1.19 (m, 1H, 1', 1' and 5b'), 1.18 (d, *J* = 6.7 Hz, 3H, 1), 1.12 (d, *J* = 6.7 Hz, 3H, 1), 1.05 (s, 0H, 11'), 1.02 (s, 3H, 11), 0.79 (dd, *J* = 8.3, 4.6 Hz, 1H, 5a and 5a'). Numbers denoted with a prime correspond to the minor diastereomer (epimer at position 7). Integration of 0.08H for the minor diastereomer corresponds to 1H'. The diastereomer ratio was determined by ^{1}H integration of signals 7/7' and combined integration of 2/2' and 4/4'.

^{13}**C NMR** (151 MHz, Chloroform-*d*): δ 195.6 (9'), 195.5 (9), 168.5 (3), 168.3 (3'), 49.1 (7), 49.0 (2'), 48.7 (2), 48.6 (7'), 46.0 (2'), 45.9 (2), 30.9 (10'), 30.8 (10), 30.5 (4'), 29.8 (4), 28.6 (6), 28.1 (6'), 21.3 (1), 21.2 (1, 1', 1', and 1'), 20.9 (1), 20.6 (1 and 1'), 19.3 (8'), 19.2 (8), 18.7 (5), 17.9 (5'), 14.3 (11'), 13.0 (11)

1*R,2*R**)-2-((*S**)-1-((1,1-dioxidobenzo[d]isothiazol-3-yl)oxy)ethyl)-*N*,*N*-diisopropyl-2-methylcyclopropane-1-carboxamide, 3y**

(1*R,2*R**)-2-((*S**)-1-(1,1-Dioxido-3-oxobenzo[*d*]isothiazol-2(3*H*)-yl)ethyl)-*N*,*N*-diisopropyl-2-methylcyclopropane-1-carboxamide, 3x**

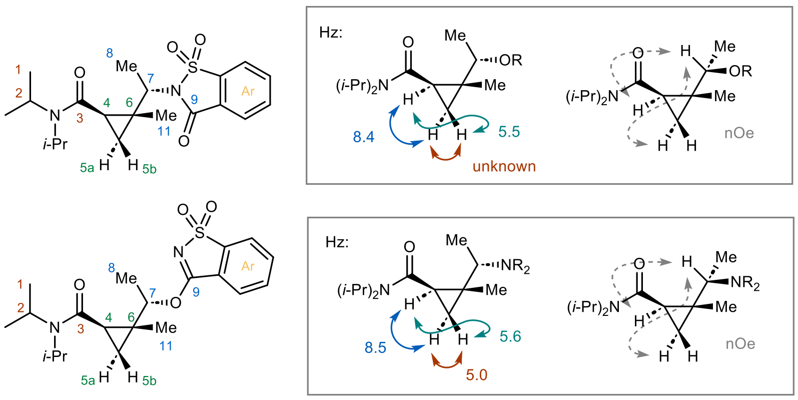

Prepared according to the general procedure using saccharin (44 mg, 0.24 mmol, 1.2 eq.). Upon completion, the reaction mixture was concentrated *in vacuo,* and the residue was purified *via* flash chromatography (1st: 1% MeOH in DCM; 2nd: 20→60% EtOAc in pentane) to yield the title compound **3y** (48 mg, 0.12 mmol, 61%) as a colourless solid and **3x** (26 mg, 0.07 mmol, 33%) as a colourless solid.

3y, ***R**f* = 0.47 (60% EtOAc in pentane)

Recrystallization: hot Et_2O

IR (thin film, ν_{max} / cm^{-1}): 1633, 1613, 1555, 1335, 1176

HRMS (ESI^+) calc. for $C_{22}H_{35}O_5N_2S$ $[M+H]^+$ 439.2261, found 439.2243

^{1}H NMR (600 MHz, Chloroform-*d*): δ 7.92 (d, *J* = 7.6 Hz, 1H, Ar), 7.80 (td, *J* = 7.4, 1.3 Hz, 1H, Ar), 7.77 (d, *J* = 7.3 Hz, 1H, Ar), 7.73 (td, *J* = 7.4, 1.0 Hz, 1H, Ar), 4.87 (q, *J* = 6.5 Hz, 1H, 7'), 4.20 (sept, *J* = 6.7 Hz, 1H, 2'), 3.32 (br, 1H, 2'), 2.07 (dd, *J* = 8.4, 5.5 Hz, 1H, 4'), 1.56 (d, *J* = 6.5 Hz, 3H, 8'), 1.40 – 1.35 (m, 4H, 1' and 5b'), 1.33 (d, *J* = 6.7 Hz, 3H, 1'), 1.21 (s, 3H, 11'), 1.04 (d, *J* = 6.7 Hz, 3H, 1'), 0.84 – 0.78 (m, 4H, 1' and 5a')

^{13}C NMR (151 MHz, Chloroform-*d*): δ 168.8 (9), 167.6 (3), 143.8 (Ar), 134.5 (Ar), 133.6 (Ar), 127.2 (Ar), 123.3 (Ar), 122.2 (Ar), 87.1 (7), 48.9 (2), 46.0 (2), 29.0 (4), 27.2 (6), 21.1 (1), 20.9 (1), 20.7 (1), 20.5 (1), 17.1 (8), 15.6 (5), 11.0 (11)

3x, *Rf* = 0.41 (60% EtOAc in pentane)

Recrystallization: EtOAc-pentane vapour diffusion

IR (thin film, ν_{max} / cm^{-1}): 1728, 1633, 1337, 1187

HRMS (ESI^+) calc. for $C_{20}H_{29}O_4N_2S$ $[M+H]^+$ 393.1843, found 393.1833

^{1}H NMR (600 MHz, Chloroform-*d*): δ 8.05 (d, *J* = 7.5 Hz, 1H, Ar), 7.92 (d, *J* = 7.6 Hz, 1H, Ar), 7.89 (t, *J* = 7.4 Hz, 1H, Ar), 7.85 (t, *J* = 7.4 Hz, 1H, Ar), 4.27 (sept, *J* = 6.8 Hz, 1H, 2), 3.96 (q, *J* = 7.2 Hz, 1H, 7), 3.30 (sept, *J*

= 6.9 Hz, 1H, 2), 2.09 (dd, J = 8.5, 5.6 Hz, 1H, 4), 1.67 (d, J = 7.2 Hz, 3H, 8), 1.37 (d, J = 6.8 Hz, 3H, 1), 1.32 (d, J = 6.9 Hz, 4H, 1 and 5b), 1.24 (s, 3H, 11), 1.02 (d, J = 6.6 Hz, 3H, 1), 0.97 (dd, J = 8.5, 5.0 Hz, 1H, 5a), 0.86 (d, J = 6.7 Hz, 3H, 1)

^{13}C NMR (151 MHz, Chloroform-*d*): δ 168.4 (3), 159.6 (9), 137.7 (Ar), 135.0 (Ar), 134.6 (Ar), 127.2 (Ar), 125.1 (Ar), 120.9 (Ar), 58.7 (7), 48.6 (2), 45.8 (2), 28.0 (4), 27.2 (6), 21.1 (1), 20.9 (1), 20.8 (1), 20.6 (1), 18.0 (5), 15.1 (8), 14.8 (11)

Single Crystal Data for **3y**: C20H28N2O4S, Mr =392.52. 150 K – monoclinic, P 2_1/c, a = 10.31090(10) Å, b = 15.87850(10) Å, c = 12.40960(10) Å, β = 94.0629(8)°, V = 2026.61(5) Å^3, Data/restraints/parameters – 4194/0/245, Rint = 0.021, Final R1 = 0.0298, wR2 = 0.0781 (I>2σ(I)).

Single Crystal Data for **3x**: C20H28N2O4S, Mr =392.54. 300 K – monoclinic, P 2_1/c, a = 10.94120(10) Å, b = 15.4773(2) Å, c = 12.5974(2) Å, β = 100.2166(11)°, V = 2099.42(5) Å^3, Data/restraints/parameters – 4382/0/244, Rint = 0.039, Final R1 = 0.0457, wR2 = 0.1252 (I>2σ(I)).

(1*R,2*R**)-*N*,*N*-Diisopropyl-2-methyl-2-((*S**)-1-((1-phenyl-1*H*-tetrazol-5-yl)thio)ethyl) cyclopropane-1-carboxamide, 3aa**

(1*R,2*R**)-*N*,*N*-Diisopropyl-2-methyl-2-((*S**)-1-(4-phenyl-5-thioxo-4,5-dihydro-1*H*-tetrazol-1-yl) ethyl)cyclopropane-1-carboxamide, 3z**

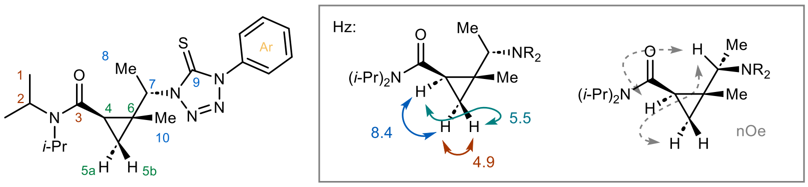

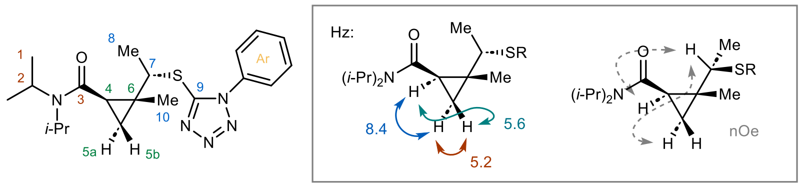

Prepared according to the general procedure using 1-phenyl-1H-tetrazole-5-thiol (35 mg, 0.24 mmol, 1.2 eq.). Upon completion, the reaction mixture was concentrated *in vacuo,* and the residue was purified *via* flash chromatography (10→70% Et_2O in pentane) to yield the title compound **3aa** (27 mg, 0.07 mmol, 35%) as a colourless solid and **3z** (39 mg, 0.10 mmol, 50%) as a colourless solid.

3aa, R_f = 0.45 (50% Et_2O in pentane)

Recrystallization: hot Et_2O

IR (thin film, ν_{max} / cm^{-1}): 1633, 1500, 1300

HRMS (ESI$^+$) calc. for $C_{20}H_{30}O_1N_5S$ [M+H]$^+$ 388.2166, found 388.2172

^{1}**H NMR** (600 MHz, Chloroform-*d*): δ 8.03 – 7.99 (m, 2H, Ar), 7.58 (t, *J* = 7.7 Hz, 2H, Ar), 7.52 (tt, *J* = 7.5, 1.1 Hz, 2H, 1H, Ar), 4.67 (q, *J* = 7.1 Hz, 1H, 7), 4.11 (sept, *J* = 6.7 Hz, 1H, 2), 3.26 (sept, *J* = 6.7 Hz, 1H, 2), 2.42 (dd, *J* = 8.4, 5.5 Hz, 1H, 4), 1.70 (d, *J* = 7.1 Hz, 3H, 8), 1.39 – 1.34 (m, 4H, 1 and 5b), 1.30 (d, *J* = 6.7 Hz, 3H, 1), 1.22 (s, 3H, 10), 1.05 (d, *J* = 6.6 Hz, 3H, 1), 0.93 (dd, *J* = 8.4, 4.9 Hz, 1H, 5a), 0.77 (d, *J* = 6.7 Hz, 3H, 1).

^{13}**C NMR** (151 MHz, Chloroform-*d*): δ 167.8 (3), 163.7 (9), 135.0 (Ar), 129.8 (Ar), 129.5 (Ar), 123.5 (Ar), 61.9 (7), 48.7 (2), 45.9 (2), 28.3 (4), 27.6 (6), 21.1 (1), 21.0 (1), 20.7 (1), 20.5 (1), 17.2 (5), 16.9 (8), 11.7 (10)

3z, $\boldsymbol{R_f}$ = 0.17 (50% Et_2O in pentane)

IR (thin film, ν_{max} / cm^{-1}): 1636, 1300, 738

Recrystallization: Et_2O-pentane vapour diffusion

HRMS (ESI^+) calc. for $C_{20}H_{30}O_1N_5S$ $[M+H]^+$ 388.2166, found 388.2166

^{1}**H NMR** (600 MHz, Chloroform-*d*): δ 7.62 – 7.53 (m, 5.75H, Ar and Ar'), 4.43 – 4.30 (m, *J* = 7.3, 6.7 Hz, 1.15H, 2 and 2'), 3.81 (sept, *J* = 6.9 Hz, 0.15H, 7'), 3.75 (q, *J* = 7.0 Hz, 1H, 7), 3.41 (br, 0.15H, 2'), 3.32 (sept, *J* = 6.8 Hz, 1H, 2), 2.12 (dd, *J* = 8.4, 5.6 Hz, 1H, 4), 1.88 (dd, *J* = 8.5, 5.4 Hz, 0.15H, 4'), 1.63 (d, *J* = 7.0 Hz, 0.45H, 8'), 1.62 (d, *J* = 7.0 Hz, 3H, 8), 1.40 (t, *J* = 5.2 Hz, 1.45H, 5b and 1'), 1.38 (d, *J* = 6.8 Hz, 3.45H, 1 and 1'), 1.33 (d, *J* = 6.7 Hz, 3.15H, 1 and 5b' COSY), 1.22 (d, *J* = 6.2 Hz, 0.45H, 1'), 1.20 (d, *J* = 6.2 Hz, 0.45H, 1'), 1.11 (s, 3.45H, 10 and 10'), 1.00 (d, *J* = 6.7 Hz, 3H, 1), 0.95 (d, *J* = 6.6 Hz, 3.15H, 1 and 5a' COSY), 0.88 (dd, *J* = 8.4, 4.7 Hz, 1H, 5a). Numbers denoted with a prime correspond to the minor diastereomer (epimer at position 7). Integration of 0.15H for the minor diastereomer corresponds to 1H'. The diastereomer ratio was determined by ^{1}H integration of signals 2/2', 7/7' and 4/4'.

^{13}**C NMR** (151 MHz, Chloroform-*d*): δ 168.0 (3), 167.6 (3'), 154.4 (9), 154.3 (9'), 133.8 (Ar and Ar'), 130.4 (Ar), 130.3 (Ar'), 130.0 (Ar), 129.9 (Ar'), 124.1 (Ar'), 124.0 (Ar), 55.2 (7), 55.1 (7'), 49.0 (2'), 48.7 (2), 46.1 (2'), 46.0 (2), 30.1 (4), 29.8 (4'), 28.6 (6), 27.9 (6'), 21.2 (1, 1' and 1'), 21.1 (1), 20.9 (1'), 20.7 (1), 20.6 (1'), 20.5 (1), 19.8 (8), 19.6 (8'), 18.9 (5), 18.8 (5'), 13.4 (10'), 12.6 (10)

Single Crystal Data for **3aa**: C20H29N5OS, Mr =387.55. 150 K – monoclinic, P 2_1/n, a = 6.40000(10) Å, b = 25.5799(3) Å, c = 12.54350(10) Å, β = 95.4338(10)°, V = 2044.29(4) $Å^3$, Data/restraints/parameters – 4202/0/244, Rint = 0.022, Final R1 = 0.0287, wR2 = 0.0717 (I>2σ(I)).

Single Crystal Data for **3z**: C20H29N5OS, Mr =387.55. 150 K – triclinic, P -1, a = 10.9405(2) Å, b = 14.6165(2) Å, c = 14.6449(2) Å, α = 103.7063(13)°, β = 104.5995(13)°, γ = 96.1543(13)°, V = 2166.75(6) $Å^3$, Data/restraints/parameters – 19183/0/487, Rint = 0.046, Final R1 = 0.0471, wR2 = 0.0906 (I>2σ(I)).

(*R)-1-((1*R**,2*R**)-2-(Diisopropylcarbamoyl)-1-methylcyclopropyl-2-*d*)ethyl 2,2,2-trifluoroacetate, 3ab**

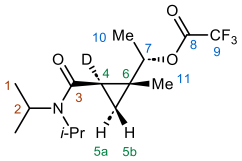

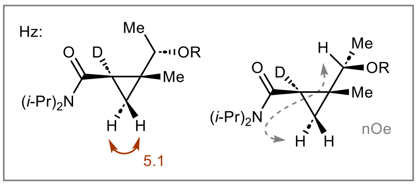

Prepared according to the general procedure using trifluoroacetic acid-*d* (19 µL, 0.24 mmol, 1.2 eq.). Upon completion, the reaction mixture was concentrated *in vacuo,* and the residue was purified *via* flash chromatography (20→60% Et_2O in pentane) to yield the title compound (53 mg, 0.16 mmol, *dr* 10:1, 86% deuterium incorporation, 80%) as a colourless oil.

R_f = 0.32 (50% Et_2O in pentane)

IR (thin film, ν_{max} / cm^{-1}): 1782, 1637, 1221, 1171

HRMS (ESI^+) calc. for $C_{15}H_{24}DO_3NF_3$ $[M+H]^+$ 325.1844, found 325.1845

^{1}H NMR (600 MHz, Chloroform-*d*): δ 4.95 (q, *J* = 6.4 Hz, 0.1H, 7'), 4.44 (q, *J* = 6.4 Hz, 1H, 7), 4.33 – 4.19 (m, *J* = 6.9 Hz, 1.1H, 2 and 2'), 3.40 (sept, *J* = 7.1 Hz, 1.1H, 2 and 2'), 1.79 (dd, *J* = 8.5, 5.5 Hz, 0.1H, 4, non *d*-product), 1.46 (d, *J* = 6.4 Hz, 3H, 10), 1.43 – 1.31 (m, 8H, 1, 1', 10', 5b, 5b'), 1.24 – 1.13 (m, 0.6H, 1 and 1'), 1.11 (s, 3.3H, 11 and 11'), 0.87 (d, *J* = 5.2 Hz, 0.1H, 5a'), 0.73 (d, *J* = 5.1 Hz, 1H, 5a). Numbers denoted with a prime correspond to the minor diastereomer (epimer at position 7). Integration of 0.1H for the minor diastereomer corresponds to 1H'. The diastereomer ratio was determined by ^{1}H integration of signals 7/7', combined integration of signals 2/2' and 11/11', and ^{19}F integration of CF_3. Due to low intensity, hydrogen 4' of the non-deuterated minor diastereomer could not be assigned.

^{13}C NMR (151 MHz, Chloroform-*d*): δ 167.7 (3'), 167.5 (3), 157.3 (q, *J* = 42.1 Hz, 8 and 8'), 114.8 (q, *J* = 286.0 Hz, 9 and 9'), 84.0 (7), 80.9 (7'), 48.8 (2 and 2'), 46.0 (2 and 2'), 29.4 (4, non *d*-product), 29.1 (t, *J* = 24.0 Hz, 4), 26.4 (6 and 4' [t, *J* = 11.8 Hz]), 26.1 (6'), 21.2 (1 and 1'), 20.7 (1, 1, 1' and 1'), 20.5 (1 and 1'), 16.9 (10), 16.5 (10'), 15.7 (5), 15.6 (5'), 13.9 (11'), 11.0 (11)

^{19}F NMR (565 MHz, Chloroform-*d*): δ -75.1 (9), -75.1 (9')

(1*R,2*R**)-*N*,*N*-Diisopropyl-2-((*S**)-1-methoxyethyl)-2-methylcyclopropane-1-carboxamide, (*S**)-3ad; (1*R**,2*R**)-*N*,*N*-Diisopropyl-2-((*R**)-1-methoxyethyl)-2-methylcyclopropane-1-carboxamide, (*R**)-3ad; (1*R**,2*R**,3*R**)-*N*,*N*-Diisopropyl-3-methoxy-2,3-dimethylcyclobutane-1-carboxamide, S7**

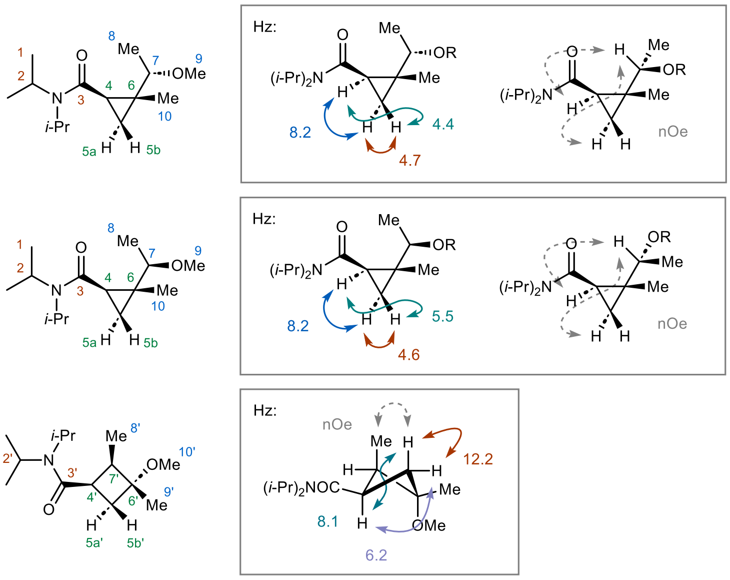

Prepared according to the general procedure using $HBF_4 \cdot Et_2O$ (1 drop) and CH_2Cl_2:MeOH (1:1, 1mL) as solvent. Upon completion, the reaction mixture was diluted with Et_2O (5 mL) and filtered through a silica pad. The filtrate was concentrated *in vacuo,* and the residue was purified *via* flash chromatography (10→60% Et_2O in pentane) to yield the title compounds **(*R**)-3ad** and **S7** (13 mg, 0.05 mmol, 27%, **(*R**)-3ad**:**S7**, 10:1) as a colourless oil and, **(*S**)-3ad** and **S7** (26 mg, 0.11 mmol, 54%, **(*S**)-3ad**:**S7**, 10:1) as a colourless oil.

(*S)-3ad** and **S7**, ***R**f* = 0.26 (50% Et_2O in pentane)

IR (thin film, ν_{max} / cm^{-1}): 2972, 1638, 1443

HRMS (ESI^+) calc. for $C_{14}H_{27}O_2NNa$ $[M+Na]^+$ 264.1934, found 264.1942

^{1}H NMR (600 MHz, Chloroform-*d*): δ 4.38 (sept, *J* = 6.8 Hz, 1H, 2), 3.86 (sept, *J* = 6.8 Hz, 0.11H, 2'), 3.42 (br, 1.11H, 2 and 2'), 3.35 (s, 3.11H, 9 and 4'), 3.20 (s, 0.33H, 10'), 2.88 (q, *J* = 6.3 Hz, 1H, 7), 2.58 (sept, *J* = 8.3 Hz, 0.11H, 7'), 2.45 (dd, *J* = 12.6, 8.2 Hz, 0.11H, 5b'), 2.02 (dd, *J* = 12.2, 6.2 Hz, 0.11H, 5a'), 1.61 (dd, *J* = 8.2, 5.5 Hz, 1H, 4), 1.41 (d, *J* = 7.0 Hz, 3.33H, 1 and 1'), 1.37 (d, *J* = 6.8 Hz, 3.33H, 1 and 1'), 1.25 (t, *J* = 4.7 Hz, 1H, 5b), 1.23 (s, 0.33H, 9'), 1.20 (d, *J* = 5.5 Hz, 6H, 8 and 1), 1.18 (d, *J* = 6.3 Hz, 3.33H, 1 and 1'), 1.14 (d, *J* = 6.6 Hz, 0.33H, 1'), 1.01 (s, 3H, 10), 0.95 (d, *J* = 7.5 Hz, 0.33H, 8'), 0.82 (dd, *J* = 8.3, 4.4 Hz, 1H 5a). Numbers denoted with a prime correspond to the **S7**. Integration of 0.11H for **S7** corresponds to 1H'. The ratio of **(*S**)-3ad** and **S7** was determined by ^{1}H integration of signals 1/1', 4/7' and 10/8'.

^{13}C NMR (151 MHz, Chloroform-*d*): δ 171.4 (3'), 169.2 (3), 82.4 (7), 79.2 (6'), 57.1 (9), 50.1 (10'), 48.6 (2), 45.9 (2 and 2'), 45.7 (2'), 41.4 (7'), 36.5 (4'), 31.0 (5'), 29.4 (9'), 26.7 (6), 24.0 (4), 21.4 (1'), 21.3 (1 and 1), 21.1 (1'), 21.0 (1), 20.9 (1'), 20.8 (1'), 20.7 (1), 17.2 (5), 18.1 (8), 12.7 (10), 12.0 (8')

(*R)-3ad** and **S7***,* ***R**f* = 0.27 (60% Et_2O in pentane)

IR (thin film, ν_{max} / cm^{-1}): 2968, 1638, 1440

HRMS (ESI^+) calc. for $C_{14}H_{27}O_2NNa$ $[M+Na]^+$ 264.1934, found 264.1937

^{1}H NMR (600 MHz, Chloroform-*d*): δ 4.70 (sept, *J* = 6.7 Hz, 1H, 2), 3.85 (sept, *J* = 6.6 Hz, 0.08H, 2’), 3.44 – 3.33 (m, 1.16H, 2, 2’ and 4’), 3.31 (s, 3H, 9), 3.20 (s, 0.24H, 10’), 2.70 (q, *J* = 6.3 Hz, 1H, 7), 2.62 – 2.55 (m, 0.08H, 7’), 2.45 (dd, *J* = 12.5, 8.0, Hz, 0.08H, 5b’), 2.02 (ddd, *J* = 12.3, 8.7, 3.3 Hz, 0.08H, 5a’), 1.67 (dd, *J* = 8.2, 5.5 Hz, 1H, 4), 1.41 (d, *J* = 6.8 Hz, 3.24H, 1 and 1’), 1.37 (d, *J* = 6.8 Hz, 3.24H, 1 and 1’), 1.25 (s, 0.24H, 9’), 1.22 – 1.16 (m, 9.24H, 8, 1, 1, and 1’), 1.14 (d, *J* = 6.7 Hz, 0.24H, 1’), 1.10 (t, *J* = 5.0 Hz, 1H, 5b), 0.98 (s, 3H, 10), 0.95 (d, *J* = 7.5 Hz, 0.24H, 8’), 0.65 (dd, *J* = 8.2, 4.6 Hz, 1H, 5a). Numbers denoted with a prime correspond to the **S7**. Integration of 0.08H for **S7** corresponds to 1H’. The ratio of **(*R**)-3ad** and **S7** was determined by ^{1}H integration of signals 1/1’, 4/7’ and 10/8’.

^{13}C NMR (151 MHz, Chloroform-*d*): δ 171.3 (3’), 169.2 (3), 84.0 (7), 79.2 (6’), 57.0 (9), 50.1 (10’), 48.4 (2), 45.8 (2, 2’ and 2’), 41.4 (7’), 36.5 (4’), 31.0 (5’), 30.5 (9’), 28.5 (4), 27.9 (6), 21.4 (1’), 21.3 (1 and 1’), 21.1 (1), 21.0 (1’), 20.9 (1), 20.9 (1’), 20.7 (1), 18.1 (x), 15.8 (8), 15.1 (5), 12.2 (10), 11.9 (8’)

(*S)-1-((1*R**,2*R**)-2-(Diisopropylcarbamoyl)cyclopropyl)ethyl benzoate, 3af**

1-(1-(diisopropylcarbamoyl)cyclopropyl)ethyl benzoate, 3ag

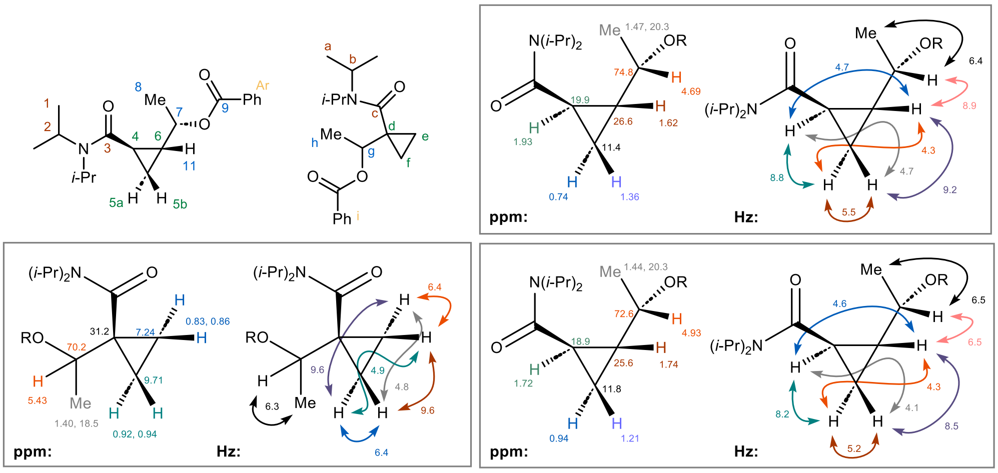

Prepared according to the general procedure using benzoic acid (29 mg, 0.24 mmol, 1.2 eq.). Upon completion, the reaction mixture was concentrated *in vacuo,* and the residue was purified *via* flash chromatography (10→30% Et_2O in pentane) to yield the title compound (34 mg, 0.11 mmol, 53%) as a colourless oil.

R_f = 0.32 (30% Et_2O in pentane)

IR (thin film, ν_{max} / cm^{-1}): 1715, 1635

HRMS (ESI^+) calc. for $C_{19}H_{28}O_3N$ $[M+H]^+$ 318.2064, found 318.2057

^{1}H NMR (600 MHz, Chloroform-*d*, 298 K): δ 8.07 – 8.01 (m, 2.7), 8.01 – 7.96 (m, 1.6), 7.59 – 7.52 (m, 2.2), 7.48 – 7.39 (m, 4.5), 5.43 (q, *J* = 6.3 Hz, 0.8), 4.93 (sept, *J* = 6.5 Hz, 0.2), 4.78 – 4.65 (m, 1.8), 4.15 (br, 1.1), 3.83 (br, 0.1), 3.66 (b, 0.61), 3.32 (s, 0.68), 1.93 (dt, *J* = 8.8, 4.7 Hz, 1.1), 1.77 – 1.70 (m, 0.6), 1.66 – 1.59 (m, 1.1),

1.47 (d, J = 6.4 Hz, 3.7), 1.44 – 1.32 (m, 10.8), 1.31 – 1.22 (m, 14.7), 1.21 – 1.10 (m, 6.8), 1.05 – 0.97 (m, 3.9), 0.96 – 0.89 (m, 1.3), 0.89 – 0.80 (m, 2.2), 0.76 – 0.72 (m, 1.2).

^{1}H NMR (500 MHz, Dimethylsulfoxide-*d*, 393 K): δ 7.99 – 7.92 (m, 3.4), 7.67 – 7.61 (m, 1.6), 7.55 – 7.49 (m, 3.5), 5.38 (q, J = 6.4 Hz, 0.8, g), 4.95 (quin, J = 6.5 Hz, 0.2, 7'), 4.75 (dq, J = 8.2, 6.4 Hz, 7), 4.15 – 3.83 (m, 4.0), 1.98 – 1.90 (m, 1.3), 1.63 – 1.51 (m, 1.2), 1.43 (d, J = 6.3 Hz, 3.4), 1.42 – 1.38 (m, 1.4), 1.32 (d, J = 6.4 Hz, 2.7), 1.30 – 1.22 (m, 14.7), 1.20 (d, J = 6.8 Hz, 6.8), 1.17 – 1.11 (m, 7.9), 1.09 – 1.00 (m, 0.5), 0.97 – 0.92 (m, 0.9), 0.92 – 0.79 (m, 4.5). Numbers denoted with a prime correspond to the minor diastereomer of **2ac**. The ratio of diastereomers and structural isomers was determined by ^{1}H integration of signals 7/7'/g, in Dimethylsulfoxide-*d* at 398 K. Integration of 0.20H for the minor diastereomer corresponds to 1H' and integration of 0.80H for the structural isomer corresponds to 1H. The figure describes key characterisation and assignments for cyclopropane connectivity and stereochemistry for clarity. See Section **S6 Variable temperature ^{1}H NMR of 3af and 3ag**, for improved resolution.

^{13}C NMR (151 MHz, Chloroform-*d,* 298 K): δ 170.9, 170.7, 169.7, 166.4, 166.2, 133.3, 133.2, 133.1, 130.7, 130.6, 130.3, 129.8, 128.7, 128.6, 128.5, 74.8, 72.7, 70.2, 48.7, 47.5, 46.0, 45.7, 34.4, 32.1, 31.2, 30.5, 29.8, 29.5, 26.6, 25.7, 22.8, 21.8, 21.5, 21.2, 21.0, 20.7, 20.4, 20.3, 20.2, 19.0, 18.5, 14.3, 11.9, 11.5, 9.9, 7.4

3-(Diisopropylcarbamoyl)-1-methylcyclobutyl 2,2,2-trifluoroacetate, 4a

(*R)-*N*,*N*-Diisopropyl-3-methylcyclobut-2-ene-1-carboxamide, S5**

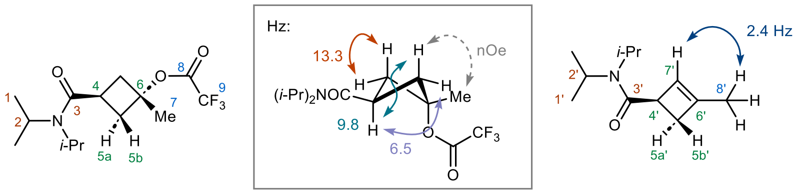

Prepared according to the general procedure using trifluoroacetic acid (17 µL, 0.24 mmol, 1.2 eq.). Upon completion, the reaction mixture was concentrated *in vacuo,* and the residue was purified *via* flash chromatography (10→40% Et_2O in pentane) to yield the title compounds (47 mg, 0.15 mmol, 76%) as a colourless oil. The ratio of **4a** and **S5** were determined by ^{1}H integration of signals 2/2'.

R$_f$ = 0.34 (30% Et_2O in pentane)

IR (thin film, ν_{max} / cm^{-1}): 1782, 1640, 1221, 1177, 1146

HRMS (ESI$^+$) calc. for $C_{14}H_{23}O_3NF_3$ [M+H]$^+$ 310.1625, found 310.1623

^{1}H NMR (600 MHz, Chloroform-*d*): δ 4.78 (q, J = 2.4 Hz, 0H, 7'), 3.85 (sept, J = 7.0 Hz, 0H, 2'), 3.72 (sept, J = 6.7 Hz, 1H, 2), 3.42 (br, 1H, 2), 3.24 (tt, J = 9.8, 6.5 Hz, 1H, 4), 3.16 (sept, J = 8.5 Hz, 0H, 2'), 3.09 – 3.02 (m, 0H, 5a'/5b'), 2.82 – 2.75 (m, 0H, 5a'/5b'), 2.66 (dd, J = 13.1, 10.5 Hz, 1H, 5b), 2.62 – 2.56 (m, 2H, 5a), 1.63 (s, 3H, 7 and 8'), 1.39 (d, J = 6.8 Hz, 6H, 1 and 1'), 1.16 (d, J = 6.7 Hz, 6H, 1 and 1'). The signal corresponding to peak 4' could not be located.

^{13}C NMR (151 MHz, Chloroform-*d*): δ 172.6 (3'), 171.7 (3), 156.4 (q, J = 41.6 Hz, 8), 145.0 (6'), 114.5 (q, J = 286.4 Hz, 9), 106.1 (7'), 84.9 (6), 48.3 (2 and 2'), 45.9 (2), 45.7 (2'), 37.0 (5), 35.3 (5'), 33.9 (4'), 31.0 (4), 24.1 (7), 21.0 (1 and 1'), 20.8 (1'), 20.7 (1), 16.7 (8', HSQC)

^{19}F NMR (565 MHz Chloroform-*d*) δ -75.6 (9)

3-(Diisopropylcarbamoyl)-1-(trimethylsilyl)cyclobutyl 2,2,2-trifluoroacetate, 4b

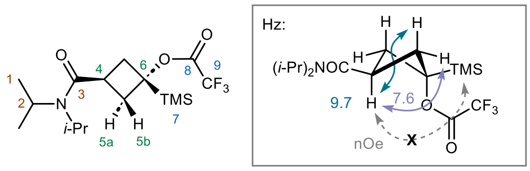

Prepared according to the general procedure using trifluoroacetic acid (17 µL, 0.24 mmol, 1.2 eq.). Upon completion, the reaction mixture was concentrated *in vacuo,* and the residue was purified *via* flash chromatography (5→20% Et_2O in pentane) to yield the title compound (34 mg, 0.09 mmol, 46%) as a colourless oil.

R_f = 0.32 (20% Et_2O in pentane)

IR (thin film, ν_{max} / cm^{-1}): 1775, 1641, 1254, 1170

HRMS (ESI$^+$) calc. for $C_{16}H_{29}O_3NF_3Si$ $[M+H]^+$ 368.1863, found 368.1862

^{1}**H NMR** (600 MHz, Chloroform-*d*): δ 3.70 (sept, J = 6.7 Hz, 1H, 2), 3.53 (tt, J = 9.7, 7.6 Hz, 1H, 4), 3.40 (br, 1H, 2), 2.80 – 2.73 (m, 2H, 5a), 2.59 – 2.53 (m, 2H, 5b), 1.39 (d, J = 6.8 Hz, 6H, 1), 1.16 (d, J = 6.7 Hz, 6H, 1), 0.11 (s, 9H, 7).

^{13}**C NMR** (151 MHz, Chloroform-*d*): δ 171.7 (3), 157.3 (q, J = 41.4 Hz, 8), 118.3 – 110.6 (q, J = 286.5 Hz, 9), 84.2 (6), 48.2 (2), 45.8 (2), 34.5 (4), 33.3 (5), 21.1 (1), 20.7 (1), -3.1 (7)

^{19}**F NMR** (565 MHz Chloroform-*d*) δ -75.2 (9)

(2*R,4*S**)-3-(Diisopropylcarbamoyl)-1,2,4-trimethylcyclobutyl 2,2,2-trifluoroacetate, 4d**

(*S)-1-((1*R**,2R*,3*S**)-2-(Diisopropylcarbamoyl)-1,3-dimethylcyclopropyl)ethyl 2,2,2-trifluoroacetate, 3ae**

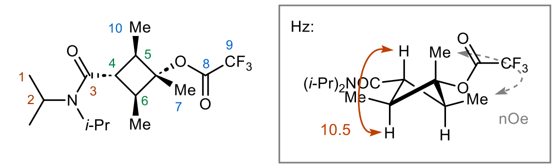

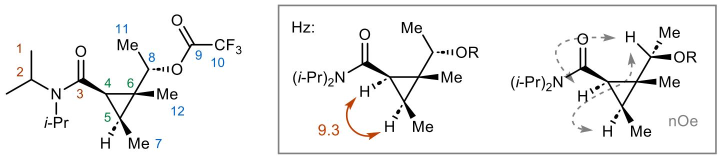

Prepared according to the general procedure using trifluoroacetic acid (19 µL, 0.24 mmol, 1.2 eq.). Upon completion, the reaction mixture was concentrated *in vacuo,* and the residue was purified *via* flash chromatography (10→40% Et_2O in pentane) to yield **4d** (16 mg, 0.05 mmol, 47%) as a colourless solid and **3ae** (11 mg, 0.03 mmol, 32%) as a colourless oil.

4d: ***R****f* = 0.48 (20% Et_2O in pentane)

IR (thin film, ν_{max} / cm^{-1}): 1778, 1637, 1219, 1168

HRMS (ESI^+) calc. for $C_{16}H_{27}O_3NF_3$ $[M+H]^+$ 338.1938, found 338.1937

^{1}H NMR (600 MHz, Chloroform-*d*): δ 3.81 – 3.69 (br, 2H, 2), 3.47 (t, *J* = 10.5 Hz, 1H, 4), 2.91 (dq, *J* = 10.4, 7.2 Hz, 2H, 5), 1.65 (s, 3H, 7), 1.30 (s, 6H, 1), 1.21 (d, *J* = 6.8 Hz, 6H, 1), 1.15 (d, *J* = 7.2 Hz, 6H, 10)

^{13}C NMR (151 MHz, Chloroform-*d*): δ 170.6 (3), 156.2 (q, *J* = 41.3 Hz, 8), 114.7 (q, *J* = 286.6 Hz, 9), 92.0 (6), 47.5 (2), 45.7 (2), 40.9 (5), 37.9 (4), 21.9 (1), 21.0 (1), 13.8 (7), 10.3 (10)

^{19}F NMR (565 MHz, Chloroform-*d*): δ -75.7 (9)

3ae: ***R****f* = 0.26 (20% Et_2O in pentane)

IR (thin film, ν_{max} / cm^{-1}): 1782, 1643, 1222, 1172

HRMS (ESI^+) calc. for $C_{16}H_{27}O_3NF_3$ $[M+H]^+$ 338.1938, found 338.1936

^{1}H NMR (600 MHz, Chloroform-*d*): δ 4.62 (q, *J* = 6.4 Hz, 0.15H, 8'), 4.40 (q, *J* = 6.4 Hz, 1H, 8), 4.23 (br, 1.15H, 2' and 2), 3.41 (br, 1H, 2' and 2), 1.56 (d, *J* = 9.3 Hz, 1.15H, 4' and 4), 1.44 – 1.32 (m, 10.35H, 1', 1, 11' and 11), 1.27 (d, *J* = 6.6 Hz, 3H, 7), 1.23 (s, 0.45H, 12'), 1.21 – 1.09 (m, 10.25H, 1', 1, 7', 12), 1.07 – 0.99 (m, 1.15H, 5' and 5). Numbers denoted with a prime correspond to the minor diastereomer (epimer at position 8). Integration of 0.15H for the minor diastereomer corresponds to 1H'. The ratio of diastereomers was determined by ^{1}H integration of signals 8/8', combined integration of signals 2/2' and 4/4' and ^{19}F integration of CF_3.

^{13}C NMR (151 MHz, Chloroform-*d*): δ 168.0 (3'), 167.8 (3), 157.5 (q, *J* = 42.3 Hz, 9'), 157.3 (q, *J* = 41.9 Hz, 9), 114.8 (q, *J* = 286.3 Hz, 10'), 114.7 (q, *J* = 286.1 Hz, 10'), 85.3 (8'), 83.4 (8), 49.3 (2'), 48.4 (2), 45.8 (2), 45.5 (2'), 30.5 (4'), 30.4 (4), 28.5 (6'), 27.8 (6), 22.2 (5'), 21.4 (5), 21.2 (1' and 1), 20.9 (1' and 1), 20.7 (1' and 1), 16.5 (11' and 11), 9.5 (7'), 9.3 (7), 9.0 (12'), 8.0 (12)

^{19}F NMR (565 MHz, Chloroform-*d*): δ -75.0 (10), -75.3 (10')

6. Variable temperature ^{1}H NMR spectra of 3af and 3ag

d6-Dimethylsulfoxide, 500 MHz, 298K

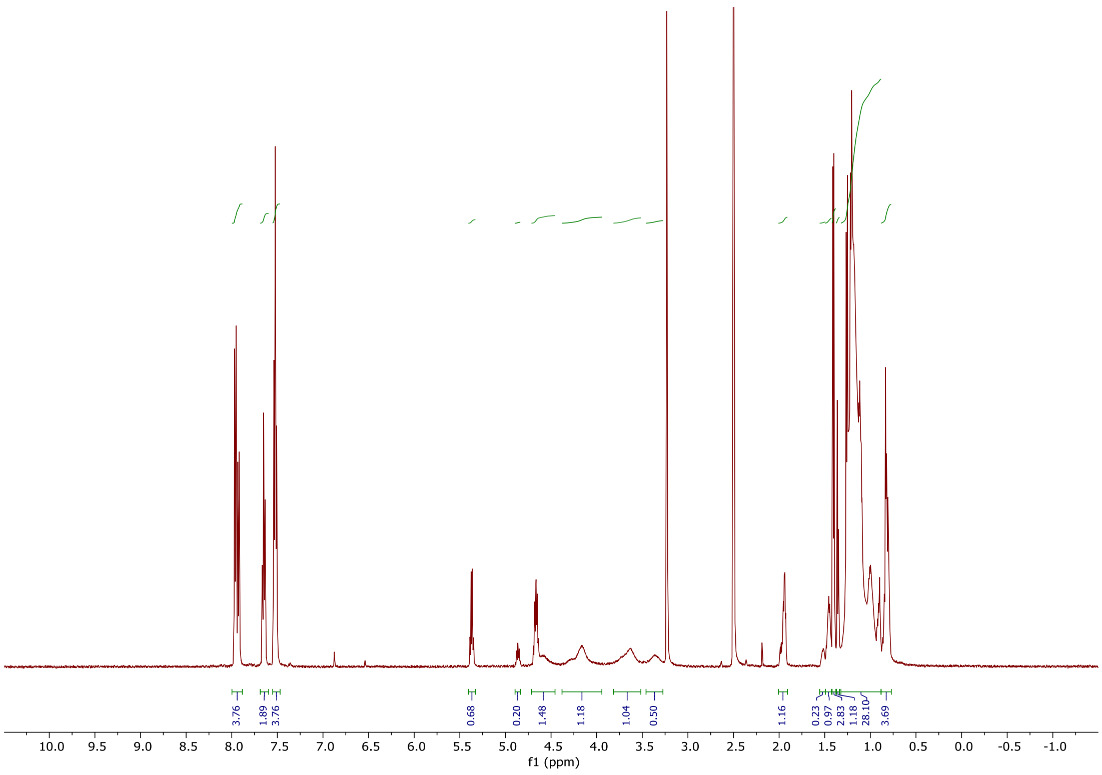

d6-Dimethylsulfoxide, 500 MHz, 393K

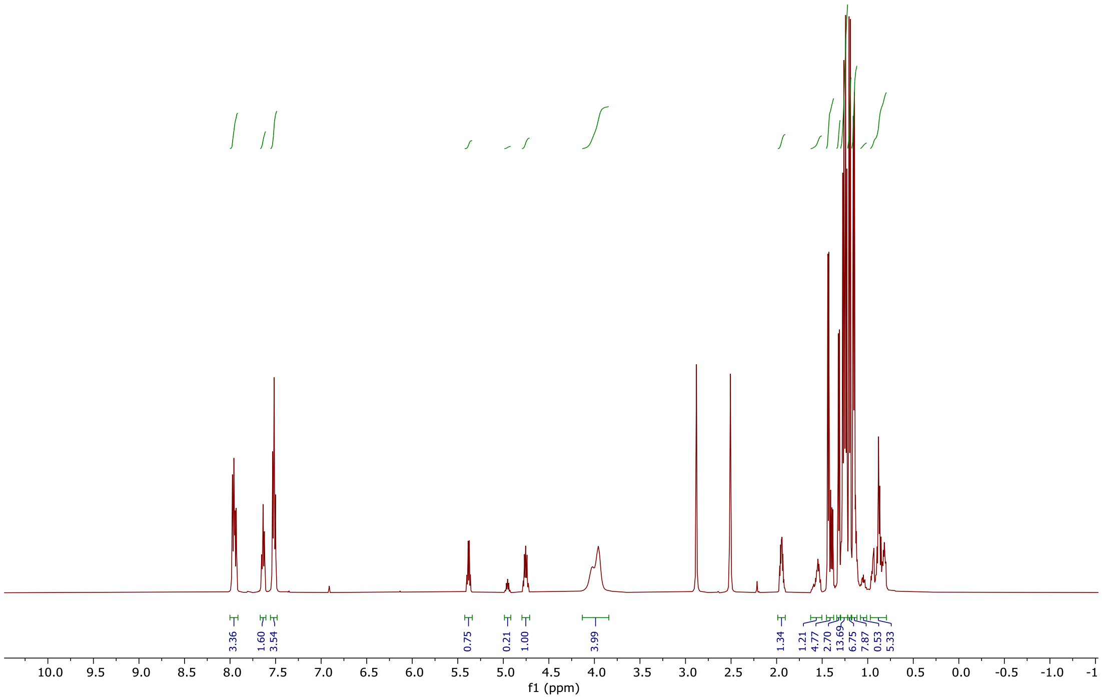

d6-Dimethylsulfoxide, 500 MHz, 298 K - 393K

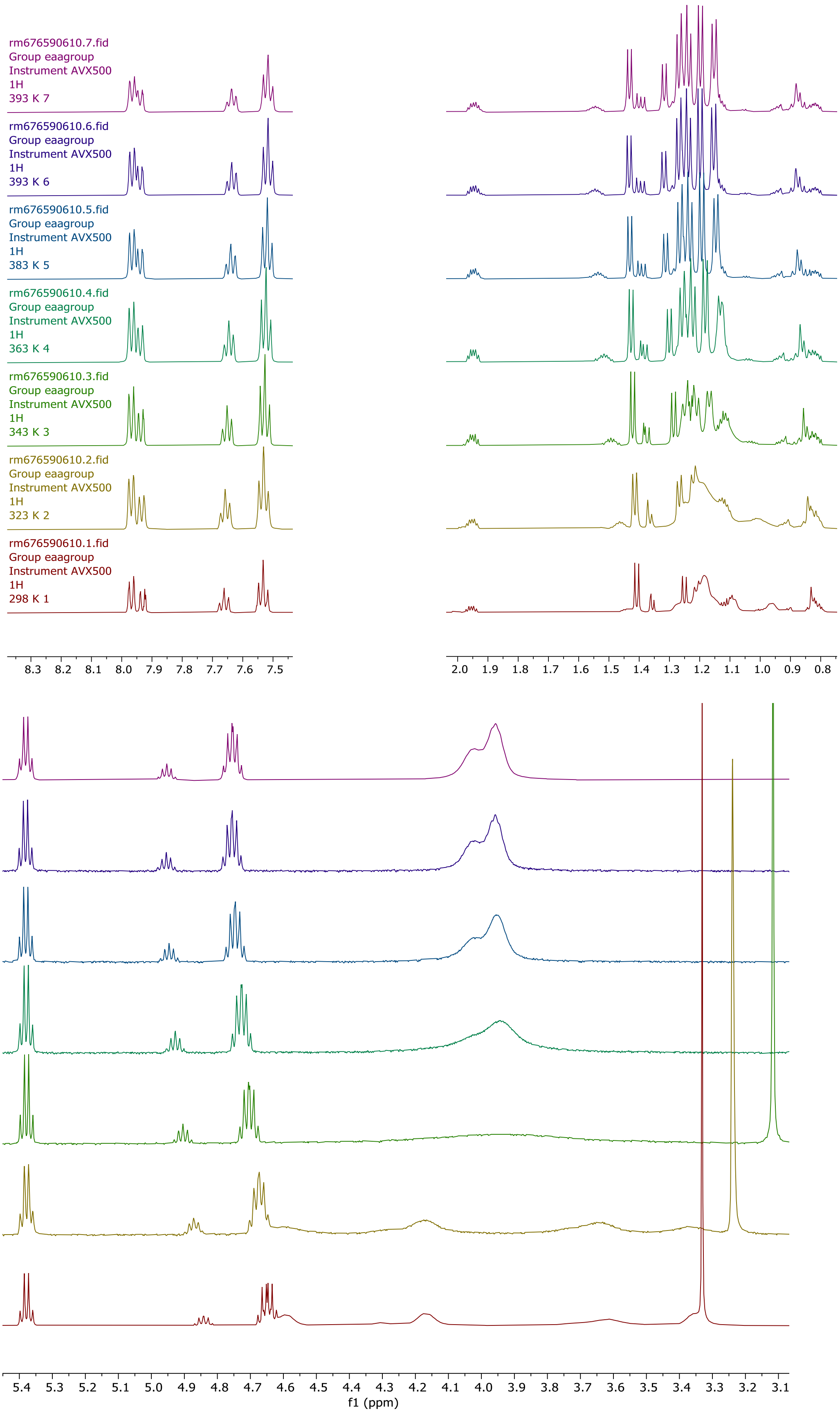

7. Ring opening of 1o with TFA

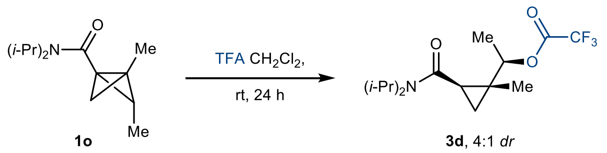

^{1}H NMR (600 MHz, Chloroform-*d*) of the crude reaction mixture and purified **3d**. Note: crude reaction profile is less clean relative to **1b** and TFA, with cyclobutyl-like peaks between 3.2 – 2.2 ppm.

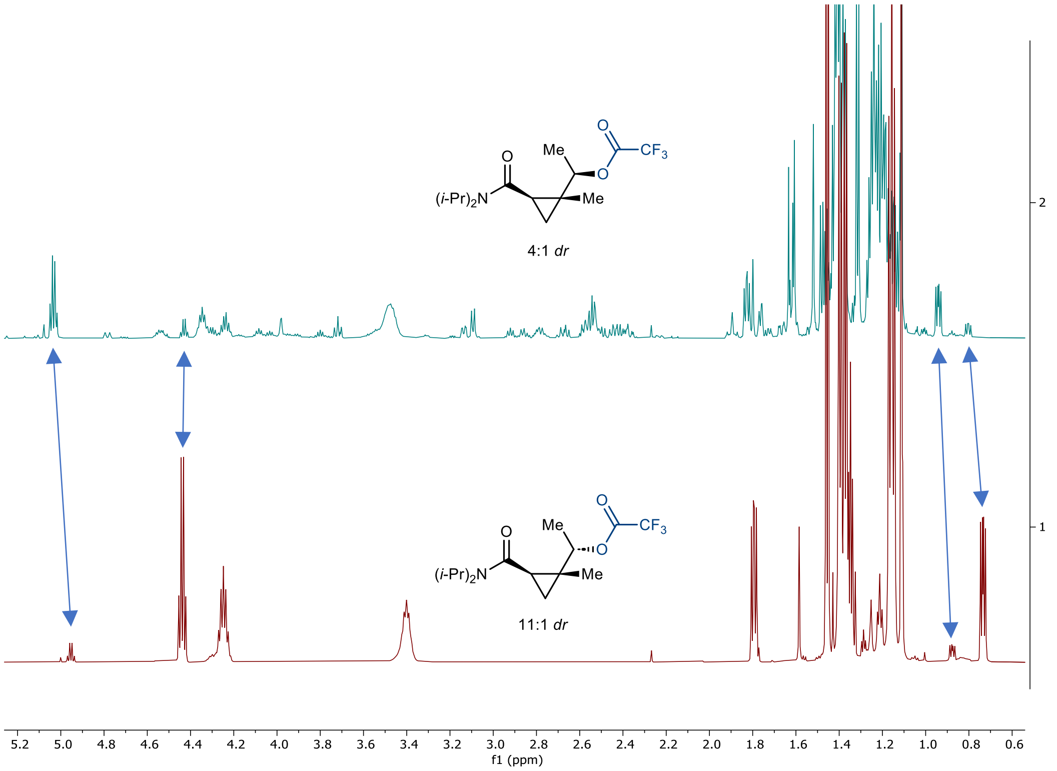

Diastereomeric ratio based on integration of cyclopropane peaks in the ^{1}H NMR spectrum.

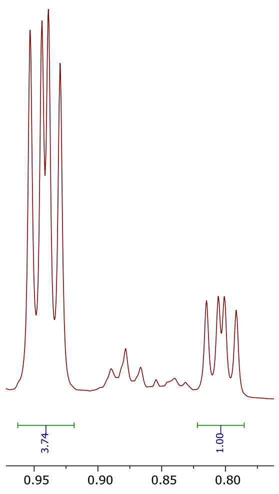

8. Competition reaction between propionic acid and propanethiol with 1b

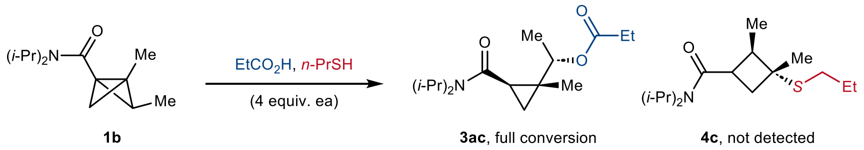

^{1}H NMR spectrum (600 MHz, Chloroform-d) of the crude reaction mixture. The reaction shows complete conversion to **3ac**, and ^{1}H NMR or LRMS could not detect **4c**.

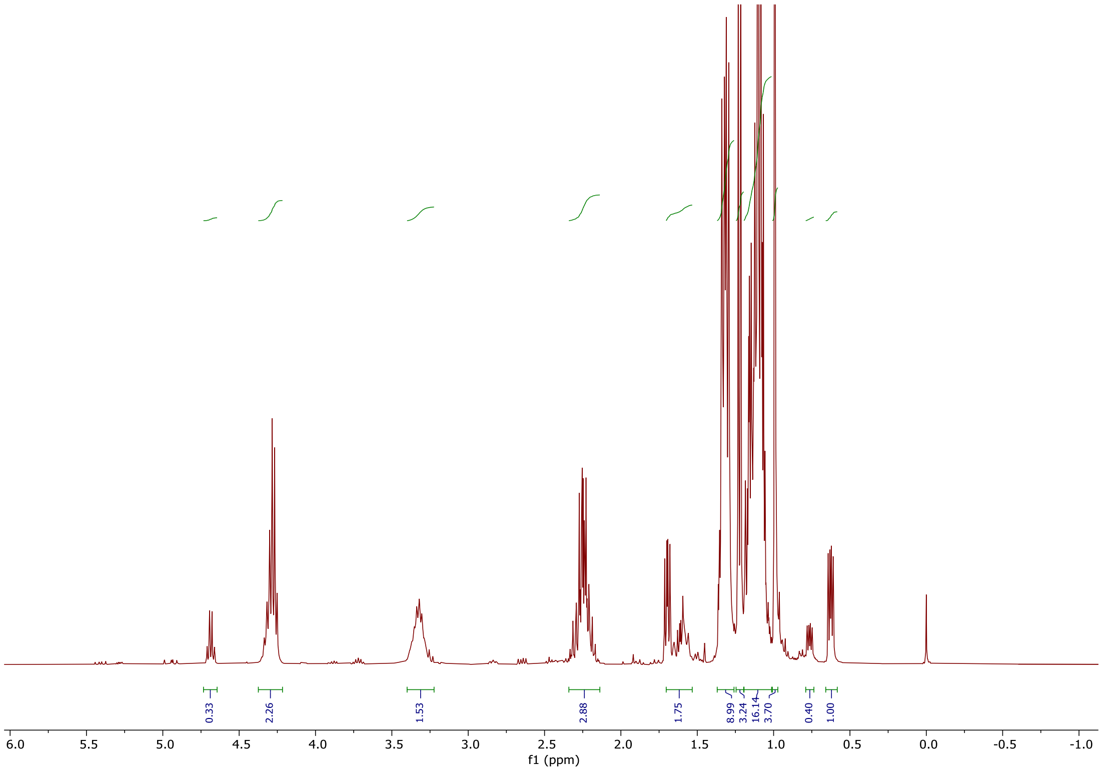

9. Crossover reaction between chloride and acetate

^{1}H NMR (600 MHz, Chloroform-*d*) of the crude reaction mixture and purified **3a** and **3b**.

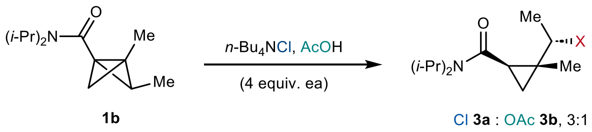

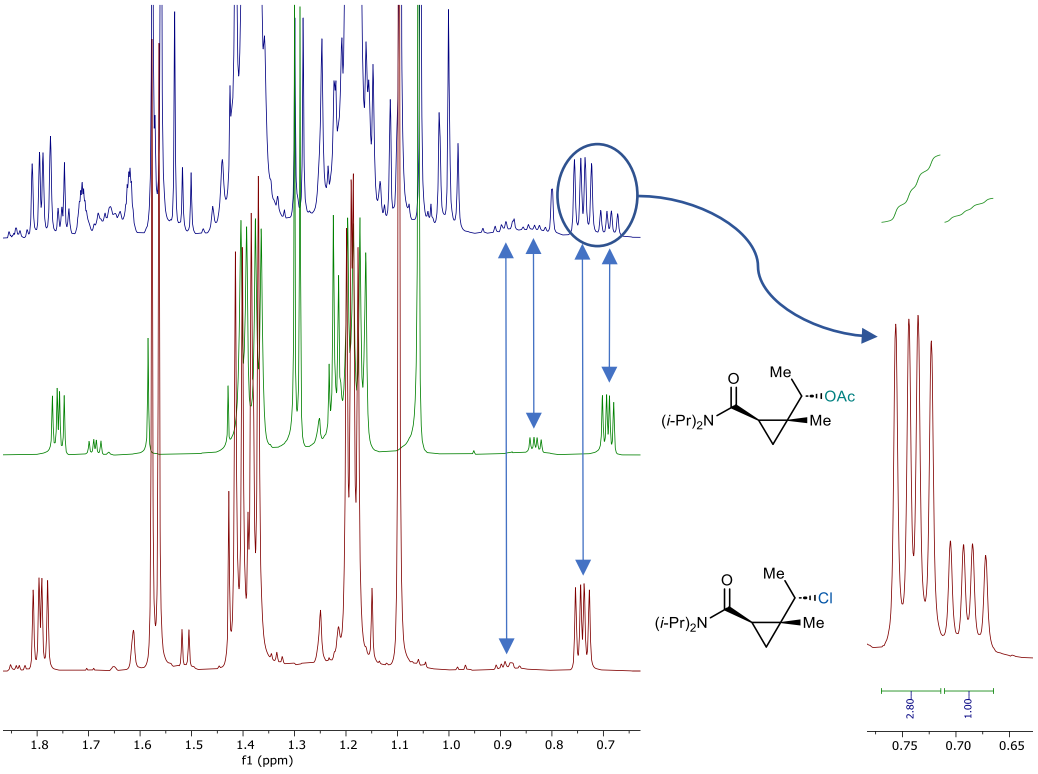

Treatment of **3a** with excess tetrabutylammonium acetate in CH_2Cl_2 at rt overnight did not form **3b.**

10. Hammett plot

A Hammett plot was constructed through competition reactions of **1b**, **1i** and **1h** with TFA. Reactions were carried out using standard conditions (CH_2Cl_2, room temperature), 4.0 equiv. of each BCB (0.40 mmol) and 1 equiv. of TFA (0.10 mmol). Competition experiments: **1b** *vs* **1i** and **1i** *vs* **1h**.

The ratio of **1b** and **1i** (5.7:1.0) was determined by the integration of ^{19}F and ^{1}H NMR. Ratio of **1i** and **1h** was determined by ^{19}F and ^{1}H NMR. No signals for **1h** was observed, therefore, the ratio for **1i** and **1h** was determined to be ≥20.0:1.0 and a lower limit (20.0:1.0) was used in calculating ρ+.

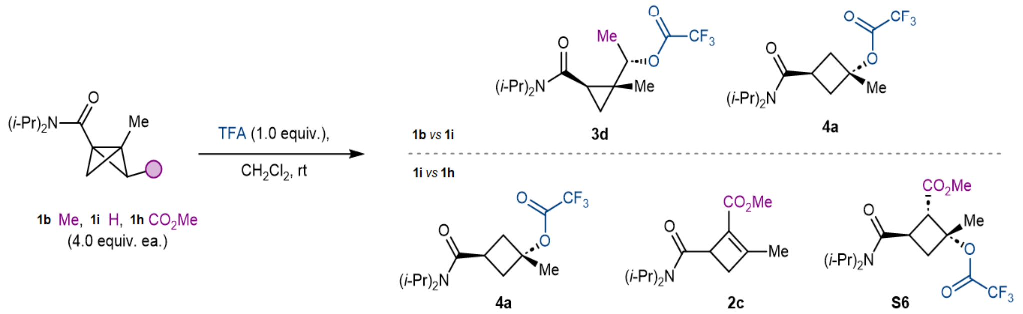

Table S1. σ^+ constants and log of relative rates of acid addition.(*47*)

X	σ^+	log([PX]/[PH])
Me	-0.311	0.76
H	0	0
CO_2Me	0.489	-1.3

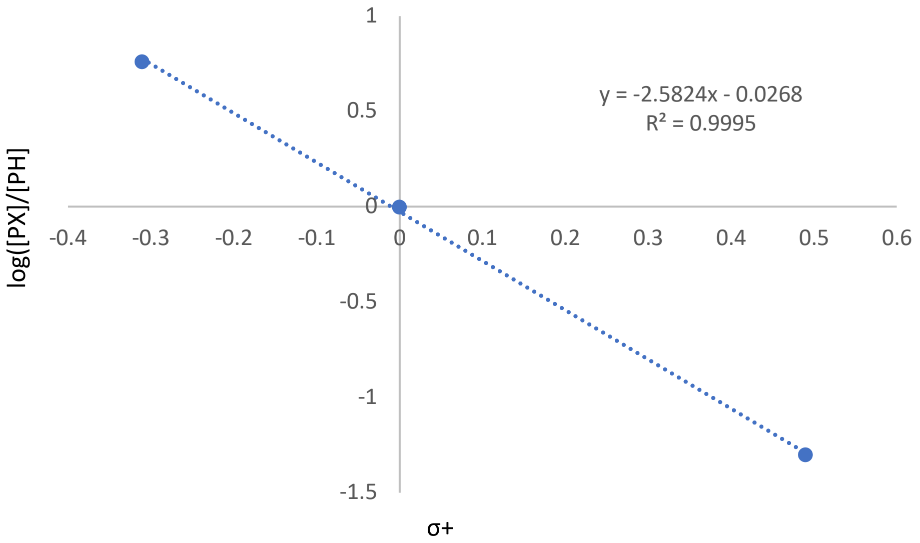

Fig. S1. Correlation of relative rates of acid addition of BCBs with σ^+ constants.

Crude ^{19}F NMR spectrum (377 MHz, Chloroform-*d*) of competition reaction between **1b** and **1i**.

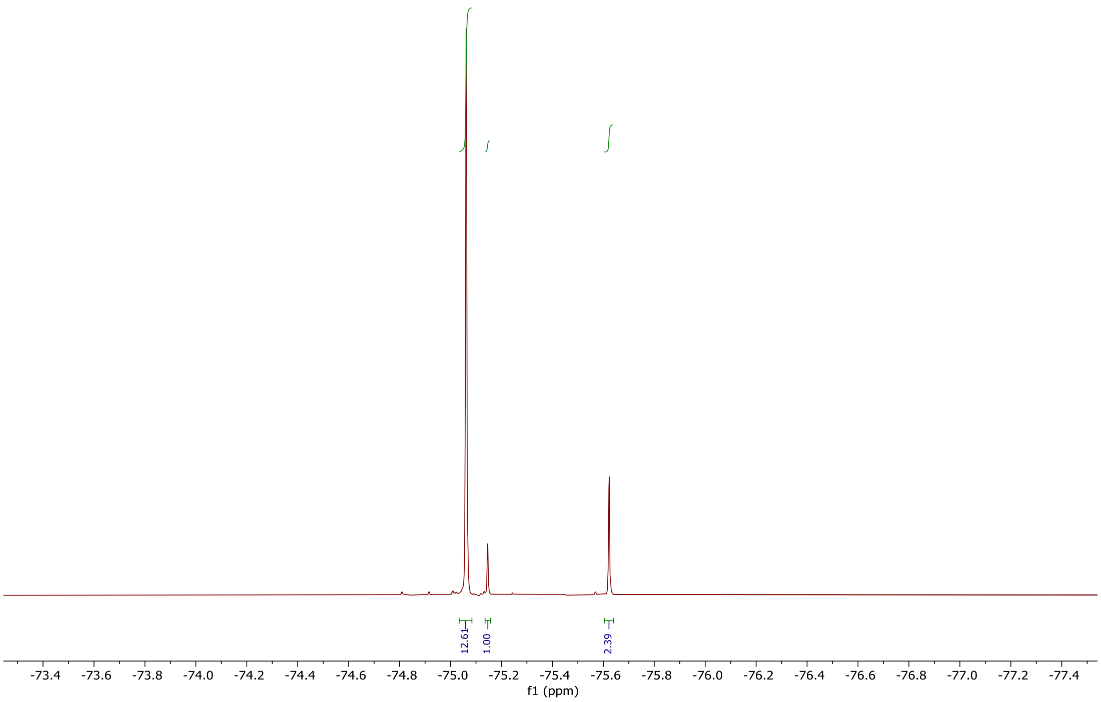

^{19}F NMR spectrum (377 MHz, Chloroform-*d*) of **1b** and **1i** competition reaction.

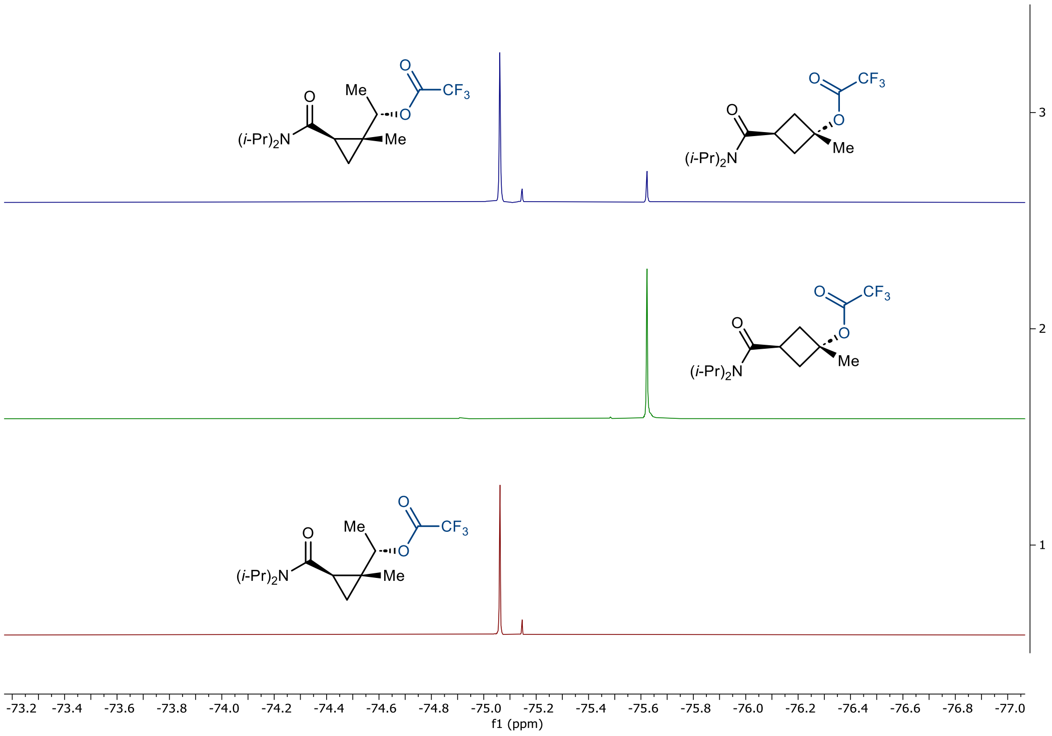

Crude ^{19}F NMR spectrum (377 MHz, Chloroform-*d*) of competition reaction between **1i** and **1h**.

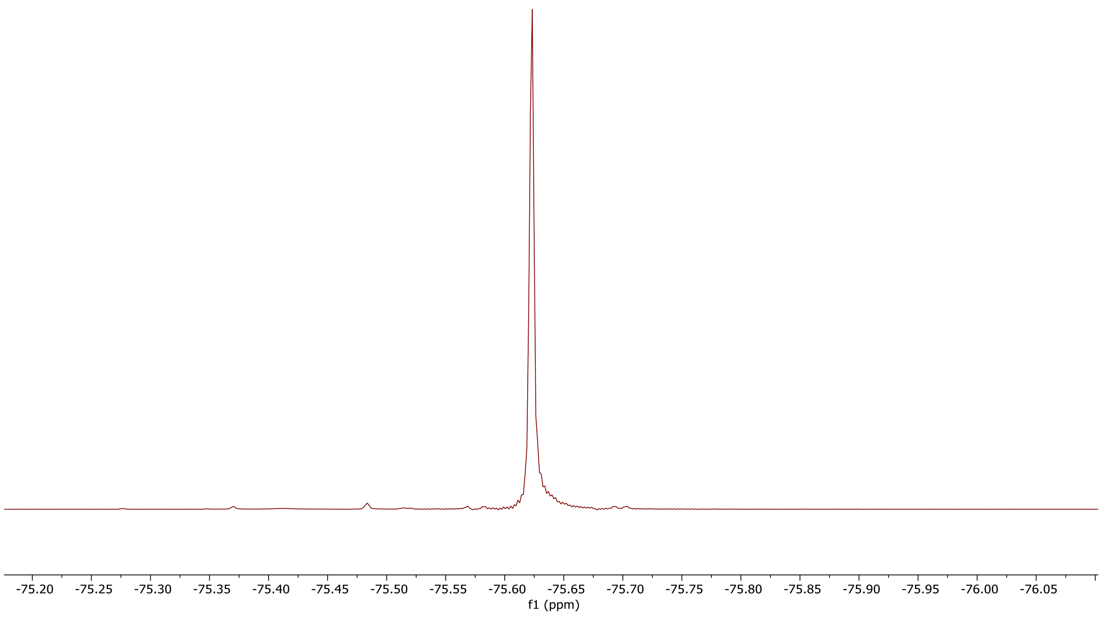

^{1}H NMR spectrum (400 MHz, Chloroform-*d*) of **S6**, **2c**, **4a** and competition reaction between **1i** and **1h**

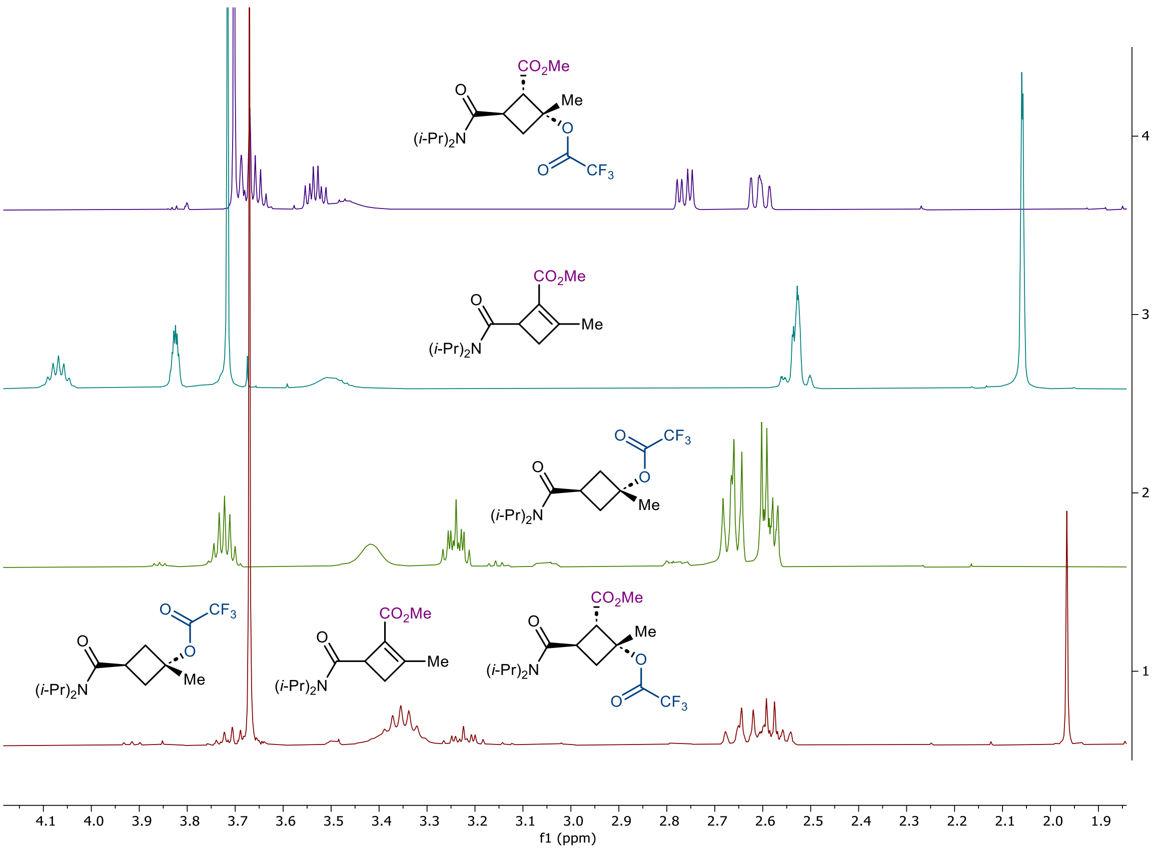

11. Diastereoselectivity vs p*K*a of Carboxylic acid

A linear plot was constructed by plotting diastereomeric excess vs p*K*a of reactions of **1b** with a selection of carboxylic acids. Reactions were carried out using standard conditions (CH_2Cl_2, room temperature, 1.2 equiv. of acid).

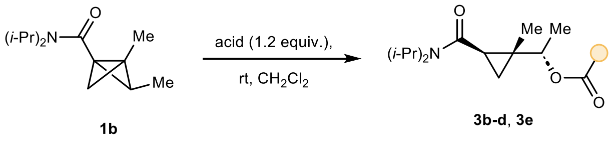

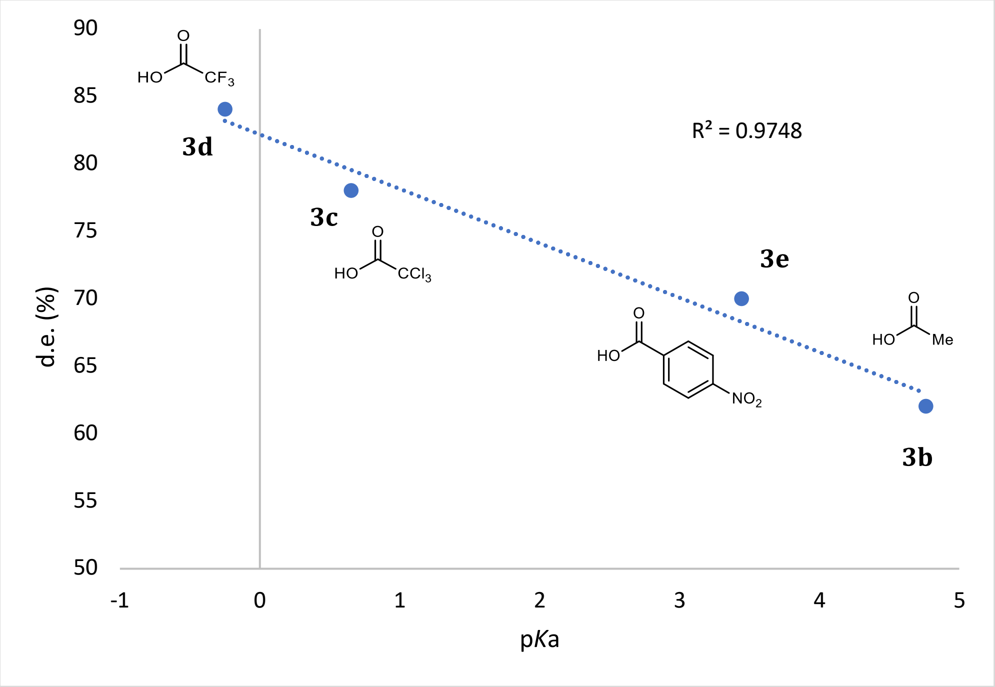

Fig. S2. Correlation between diastereoselectivity vs p*K*a of carboxylic acid.

12. Reactions of monosubstituted BCBs with acids

Monosubstituted BCBs bearing an amide, ester, nitrile or sulfone group at the bridgehead have previously, or in this work were, treated with acid in methanol or neat acid.

The parent BCB scaffold, when treated with acid in methanol, produces mainly a mixture of cyclopropane and cyclobutane, with the former being favoured. Introducing an electron-withdrawing group to the bridgehead, such as nitrile, similarly produces a mixture of the two ring systems but favouring cyclobutanes. The ester is also reported to afford a mixture of both ring systems, but the ratio or yield of the two products is not given. Switching the electron-withdrawing group to a sulphone drastically switches this preference to cyclopropane.

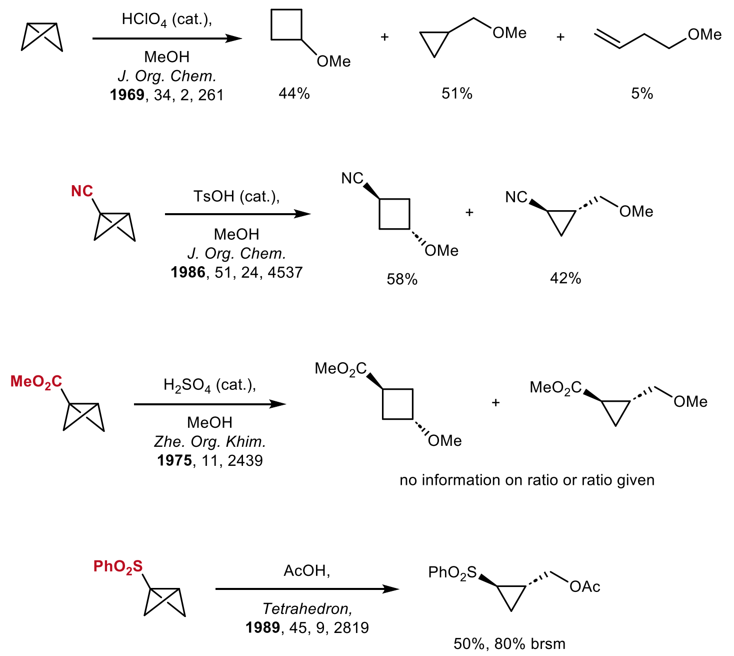

This exclusive cyclopropane formation with sulfone was surprising and was therefore repeated with sulfone (**6a**); it confirms this exceptional preference. Four compounds were produced – three of which could be characterised by analysis of the crude ^{1}H NMR spectrum. A further minor component is to contain a cyclobutane, but its low abundance made it challenging to confirm.

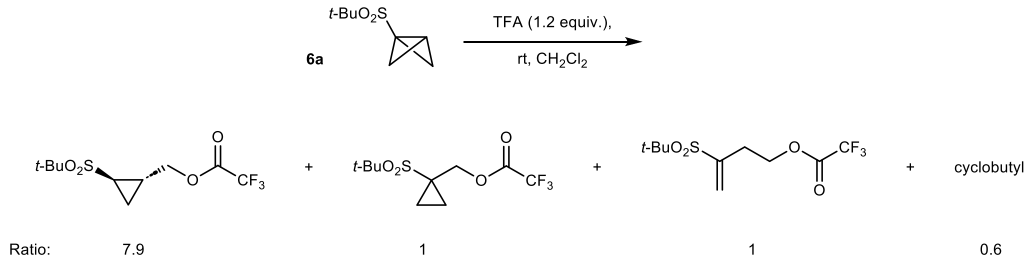

We next tested the amide (**6e**) to gain more insight into the carbonyl-based electron-withdrawing groups. To our surprise, the reaction produced a total of 6 compounds, five of which could be characterised.

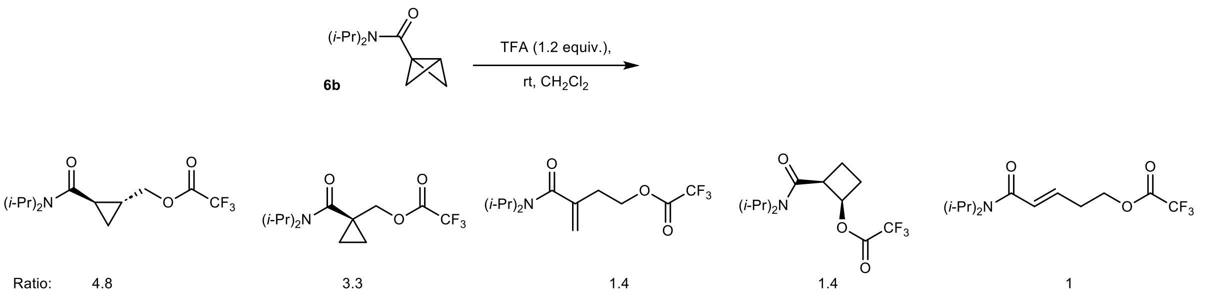

The number of compounds produced in each reaction was determined through analysis of the ^{19}F and ^{1}H NMR spectra of the crude reaction mixture. Key peaks were assigned either through 1D or 2D experiments. See the characterisation below.

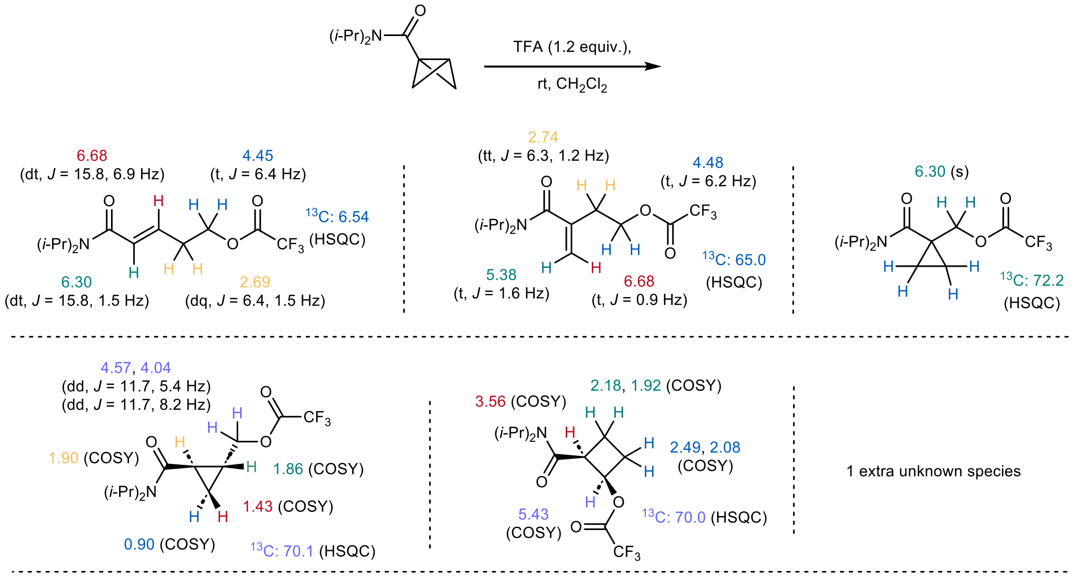

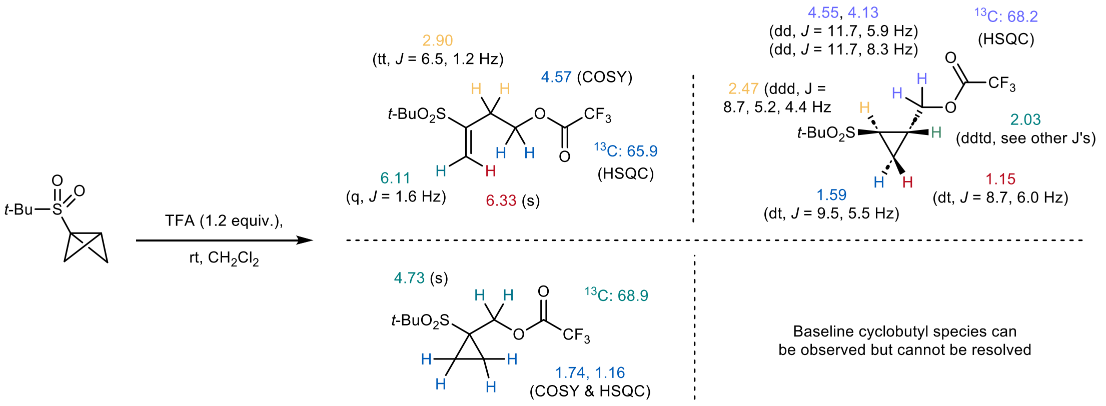

^{1}H NMR spectrum (500 MHz, Chloroform-*d*) of crude reaction from **6d**

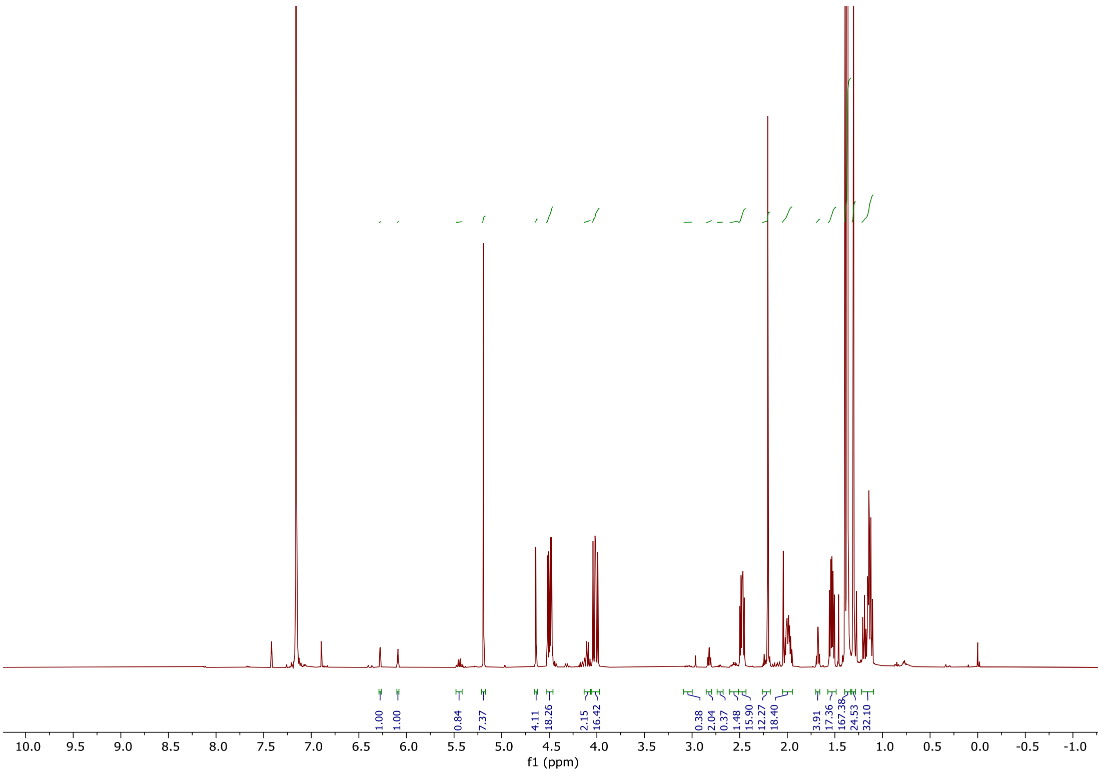

Expansion from 6.4 to 3.9 ppm

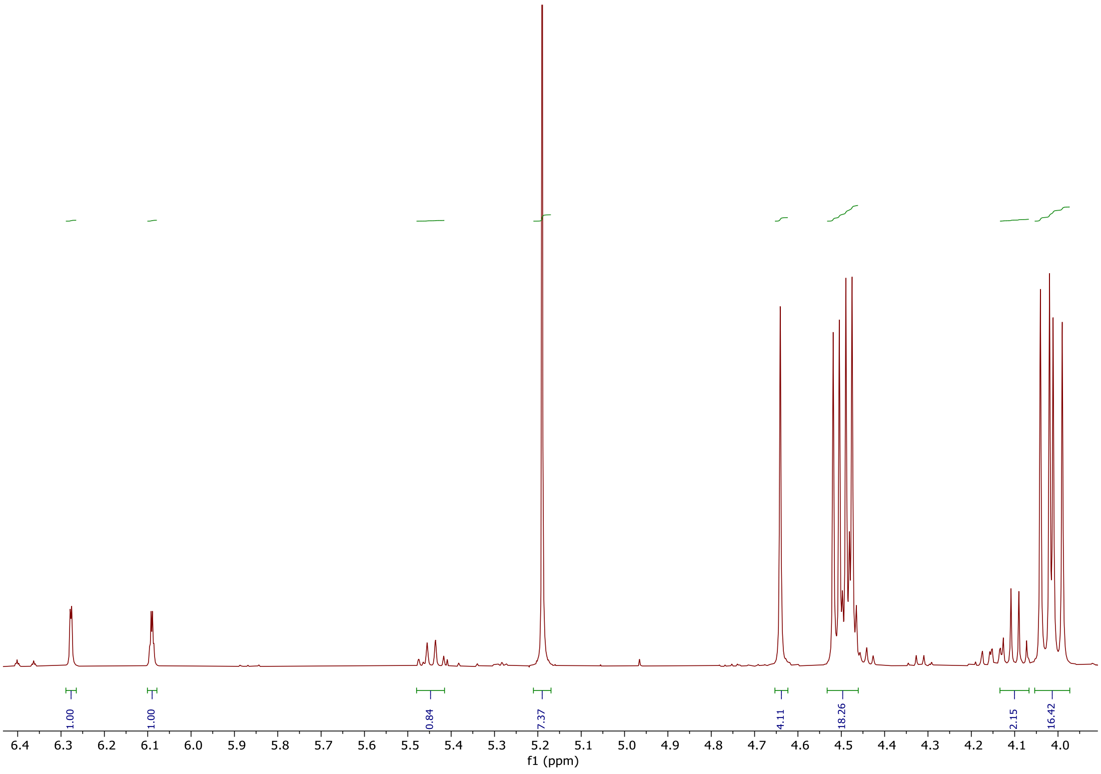

Expansion from 3.2 to 0.9 ppm

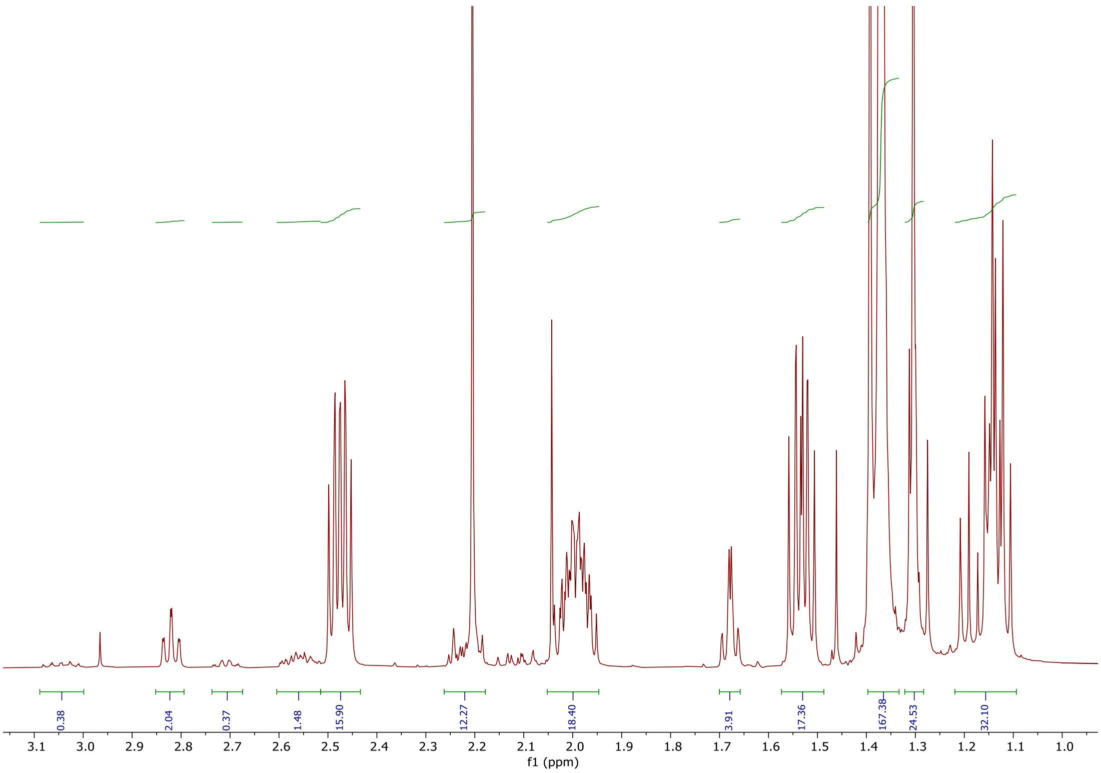

COSY (500 MHz, Chloroform-*d*) of the crude reaction mixture from **6d**

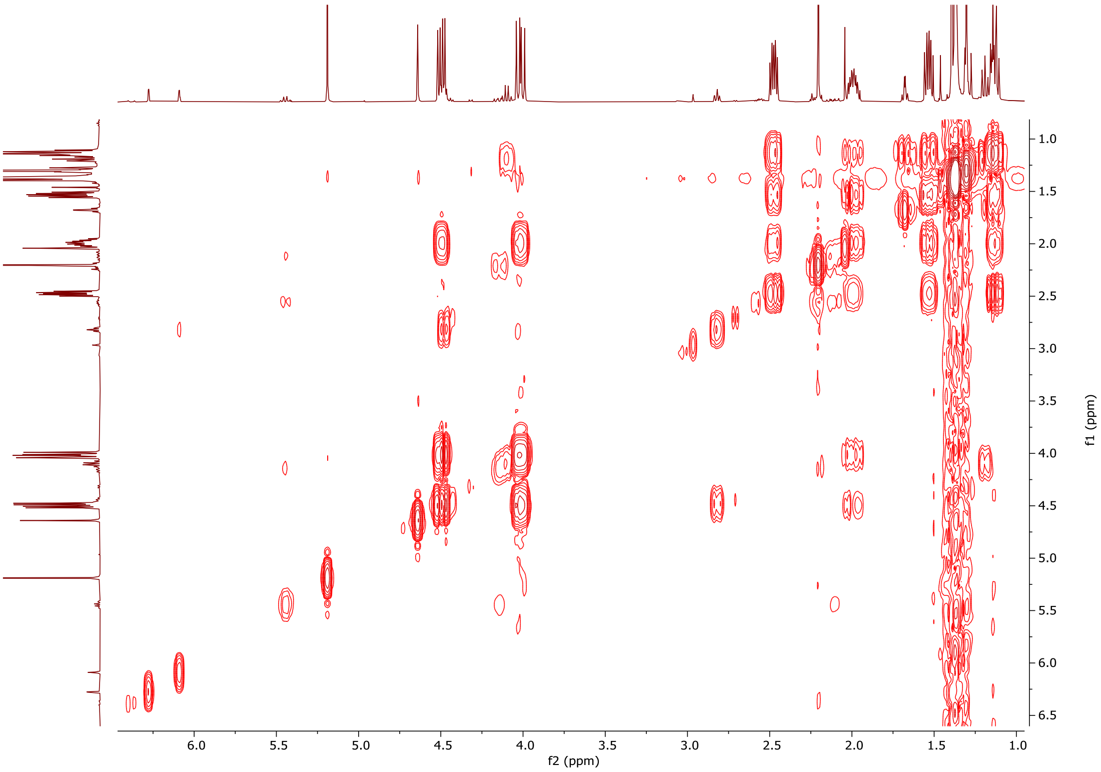

HSQC (500/126 MHz, Chloroform-*d*) of crude reaction mixture from **6d**

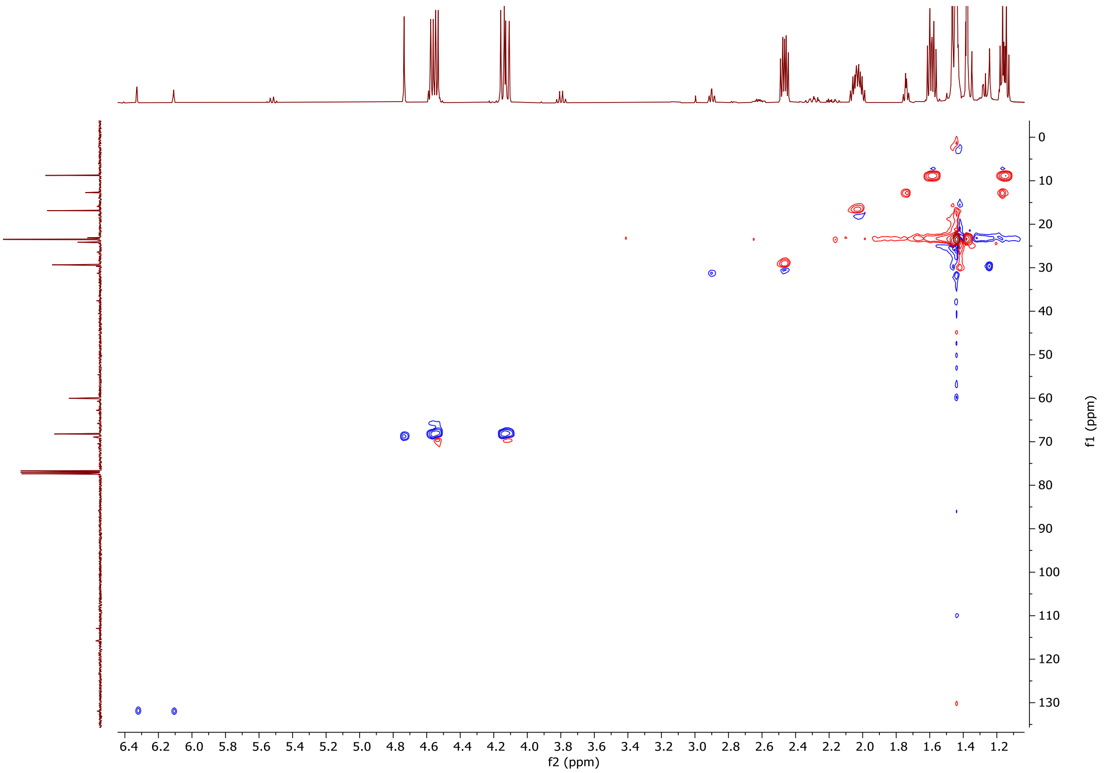

^{19}F NMR (377 MHz, Chloroform-*d*) of the crude reaction mixture from **6d**

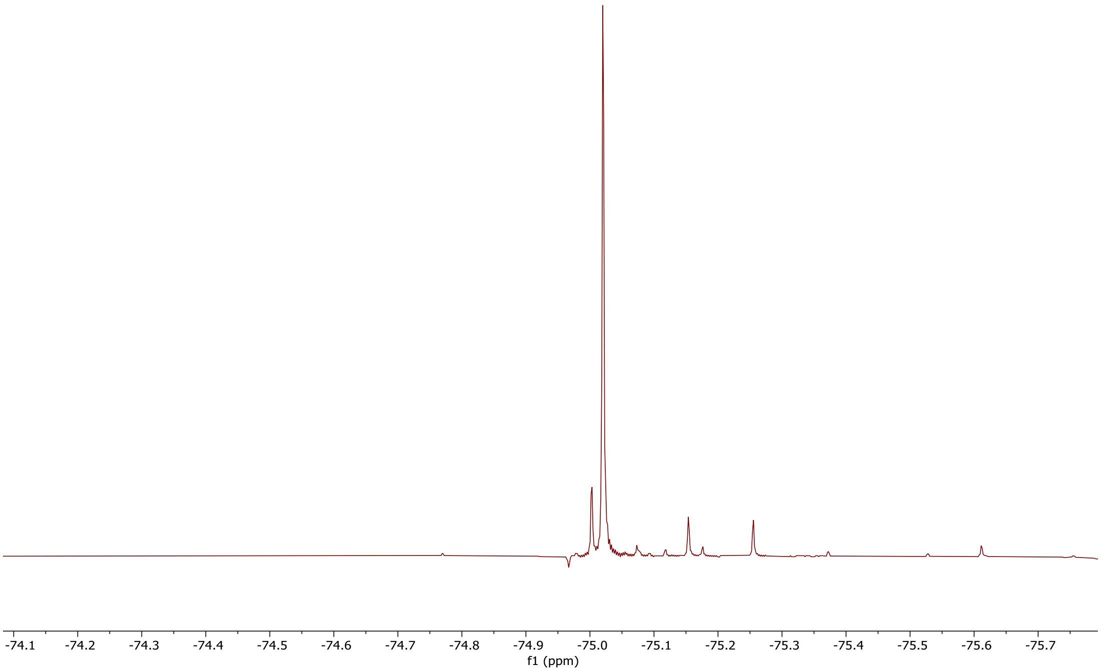

^{1}H NMR (500 MHz, Chloroform-*d*) of the crude reaction mixture from **6e**

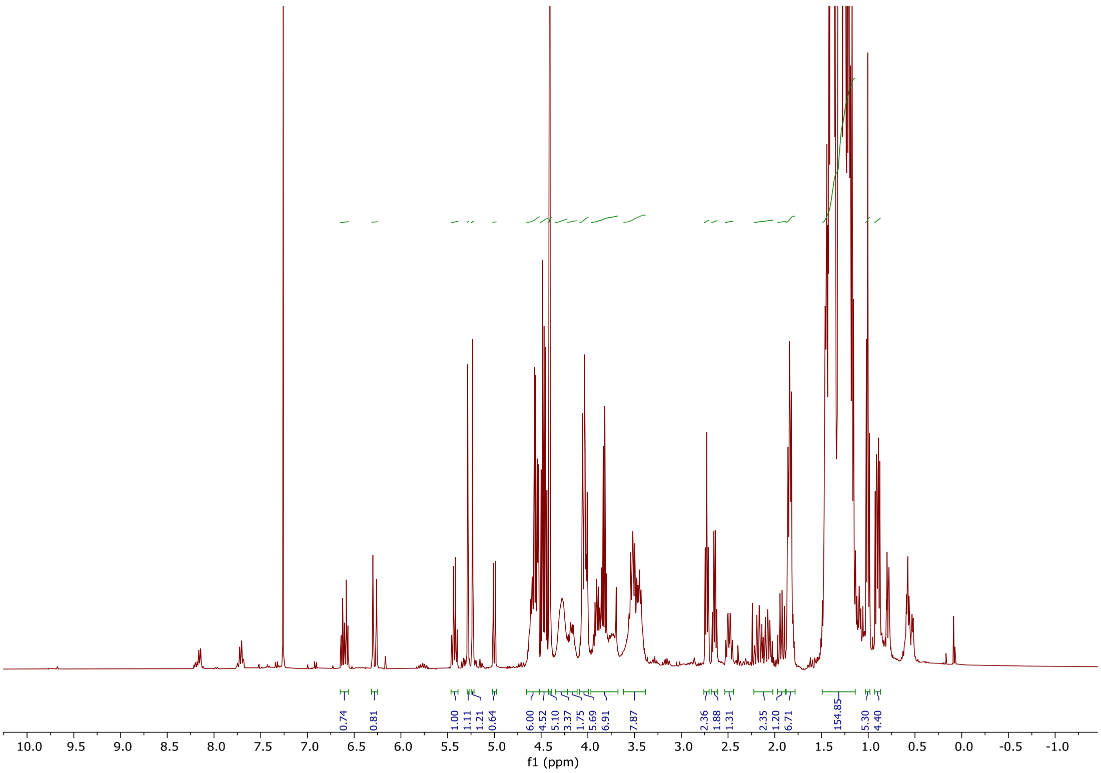

Expansion from 6.7 to 3.3 ppm

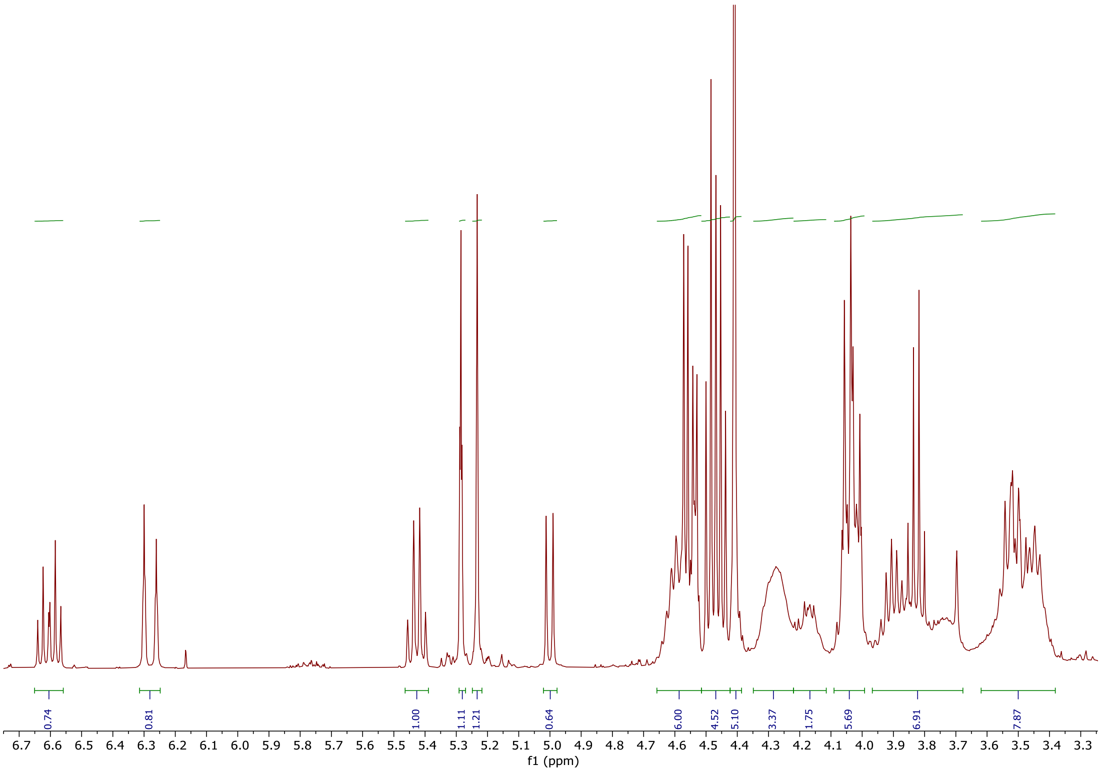

Expansion from 2.9 to 0.4 ppm

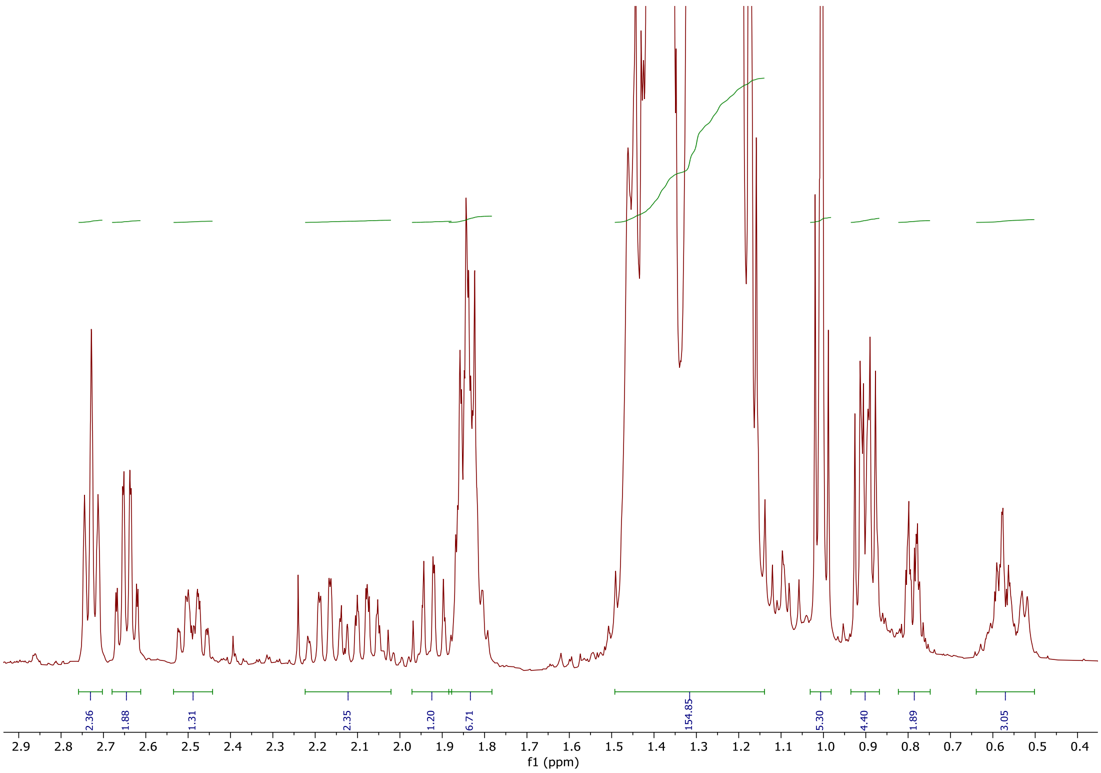

COSY (500 MHz, Chloroform-*d*) of the crude reaction mixture from **6e**

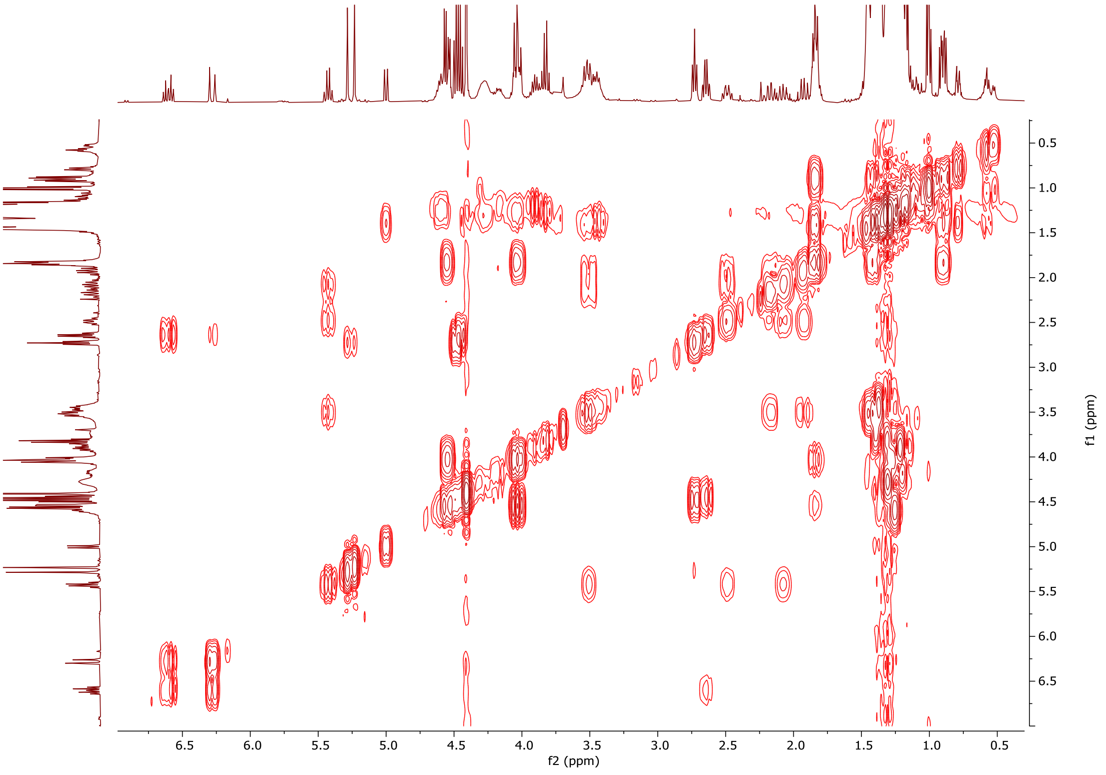

HSQC (500/126 MHz, Chloroform-*d*) of the crude reaction mixture from **6e**

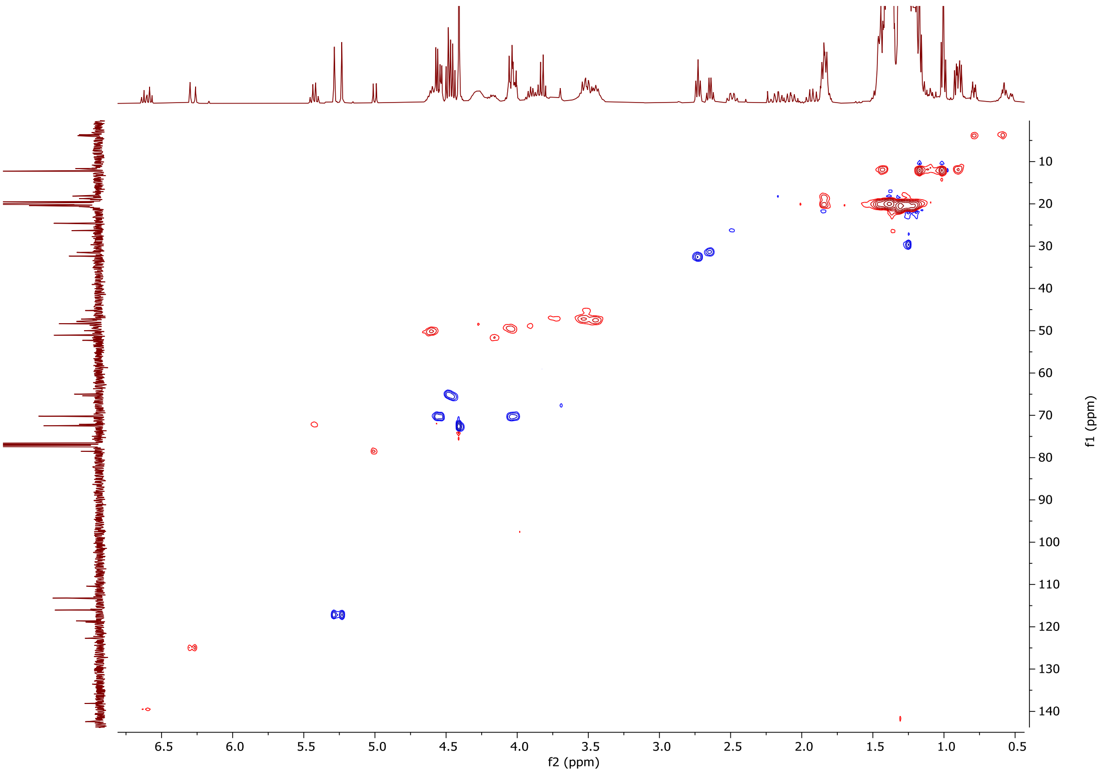

^{19}F NMR (377 MHz, Chloroform-*d*) of the crude reaction mixture from **6e**

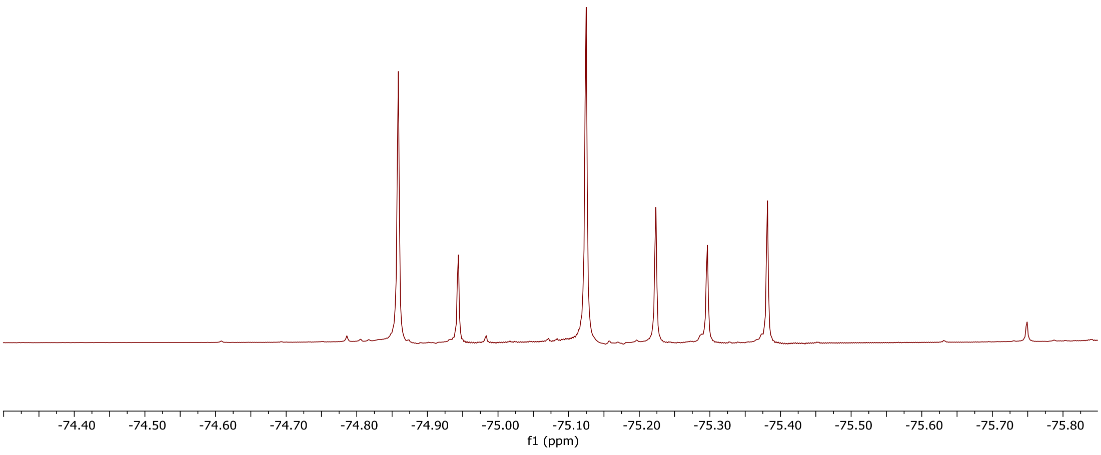

13. Computational Details

All calculations were carried out using the ORCA suite of programs (version 4.2.1) (*48, 49*). Optimizations and single-point energy calculations were carried out using "VeryTight" convergence criteria, corresponding to tolerances of 10^{-9} Hartrees for the SCF energy change, and an energy change of $2 \cdot 10^{-7}$ Hartrees for the optimisation step. A "Tight" optimisation criterion was not sufficient, resulting in the minimisation algorithm stopping at different points on the potential energy surface (PES) depending on different starting points due to a shallow minimum, especially along r_2 (Fig. S6).

RI-SCS-MP2 (Spin-Component Scaled Møller–Plesset perturbation theory) was used as the central method for geometry optimisation (see Section 14) (*50, 51*). To speed up the calculations, the resolution-of-identity (RI) approximation was employed (*52*). For calculations of triple-zeta and quadruple-zeta basis set quality, correlation integrals used the cc-pVTZ/C and cc-pVQZ/C auxiliary basis set, respectively (*53*).

The Domain-based Local Pair Natural Orbital coupled cluster method with singles, doubles and perturbative triples (DLPNO-CCSD(T)) was used as a final single point providing reliable electronic energies using the cc-pVQZ basis set (*53*). DLPNO-CCSD(T) calculations were run using "NormalPNO" cut-offs, corresponding to TCutPairs = 10^{-5} Hartrees, TCutPNO = 3.33 10^{-7} and TCutMKN = 10^{-3}, where TCutPairs is the correlation threshold for inclusion of a given electron pair in the CCSD calculation, TCutPNO is the occupation threshold for each PNO (pair natural orbital) formed from an expansion of projected atomic orbitals (PAOs), and TCutMKN determines the threshold for the inclusion of each localized MO in the PNO expansion based on Mulliken population (*54*).

Vibrational frequencies were computed at the optimization level of theory to confirm whether the structures correspond to minima or transition states. All intermediate structures were verified to be minima by the absence of imaginary frequencies upon calculation of the Hessian. Grimme's quasiRRHO approach was used to calculate free energies at 298.15 K (*55*). A standard state correction from 1 atm to 1 M was applied by adding RTln(1/24.5) (T = 298.15 K) to the calculated free energy of each species. For calculating thermodynamic data the python-script *OTherm.py* was used with $\omega_0 = 100$ cm^{-1} replacing harmonic oscillators with free-rotors below ω_0 (*55, 56*).

The 2D surface (More O'Ferrall-Jencks plot, Fig. S6) was generated in the space defined by the forming r_1 and r_2 bonds distances using a grid of (0.05 Å, 0.10 Å). The script used for visualization of the PES employed a cubic spline (*scipy.interp2d*) function.

Mayer bond orders (*57*) of **5a** were directly used from the ORCA output files and are discussed in the main text. It should be noted that Mayer bond orders possess a similar basis set dependency as Wiberg bond orders and therefore care should be taken in comparing absolute values (*58*).

14. Evaluation of level of theory

Nonclassical carbocations have been object of numerous computational studies (*59, 60*), often performed in the pre-2000s where computational power was limited. More recently, a similar system was studied by Larmore et al. at ωB97X-D/def2-TZVPP and M06-2X/def2-TZVPP levels of theory (*22*). Also of relevance is the computational work by Olah et al. on a non-substituted bicyclobutonium ion, which was performed at the MP4(SDTQ)/cc-pVTZ//MP2/cc-pVTZ level of theory (*9*). To investigate if a similar high level of theory was needed, basis set and computational methods were screened.

Simplifying the substrate: To reduce computational cost the experimental ^{i}Pr-substitutents on the amide were replaced by Me-groups (see Fig. S3). After protonation of the (simplified) bicyclobutane derivative **1b'**, carbocation **5b** is formed. This structure was subjected to a method screen using as a reference the geometry obtained at CPCM(DCM)-SCS-MP2/cc-pVQZ level of theory.

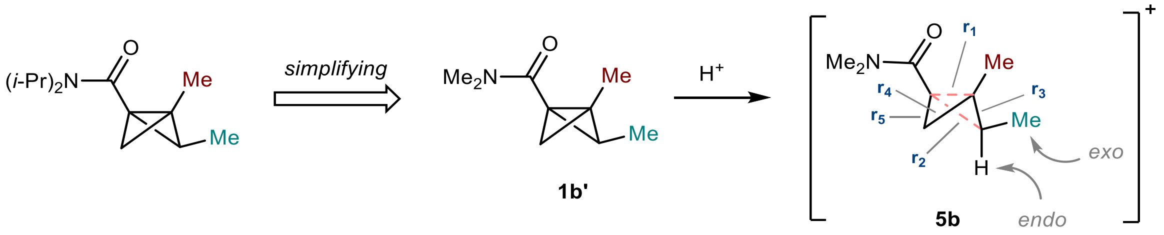

Fig. S3. Simplification of the substrate, and the nomenclature (r_1, r_2 etc.) used in this work.

Basis set and DFT level of theory Firstly, the Karlsruhe (a def2-SVP and def2-TZVPP) (*61*) and Dunning's correlation consistent (cc-pVTZ) (*53*) basis sets were screened (at CPCM(DCM)-SCS-MP2 level of theory). Both cc-pVTZ and a def2-TZVPP delivered consistent results with deviations below 1 pm from the reference CPCM(DCM)-SCS-MP2/cc-pVQZ level of theory (Fig. S4).

Secondly, different DFT functionals were tested to evaluate whether they deliver consistent results. For all tested functionals, r_3 and r_4 were within a few pm between the different functionals and consistent with the SCS-MP2 reference geometry. However, deviations of several deca-picometers in r_1 and r_2 were observed for the GGA functional BLYP, as well as for the hybrid GGA functional B3LYP. In contrast, the hybrid GGA functional PBE0 delivered consistent geometric bond lengths compared to the SCS-MP2 reference. Even double hybrid functionals, such as B2PLYP deviated significantly in r_2. Therefore, a decision was made to abandon DFT since it could not achieve consistent results at higher levels of theory and instead rely on the SCS-MP2 method at still acceptable computational costs.

Singlet-Triplet gap: A vertical (unrelaxed) singlet-triplet gap of 63.4 kcal mol^{-1} (2.75 eV) excludes a possible diradicaloid contribution.

In summary, a CPCM(DCM)-SCS-MP2/cc-pVTZ level of theory was chosen to investigate the geometries of this system **5a**. A CPCM(DCM)-DLPNO-CCSD(T)/cc-PVQZ level of theory was chosen for electronic energy evaluations.

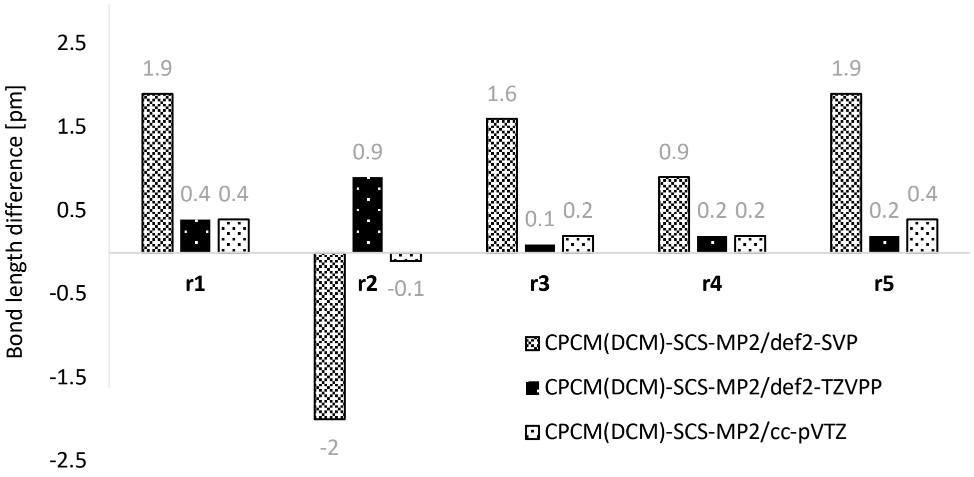

Fig. S4. Effect of basis set on r_1, r_2, r_3, r_4 and r_5 relative to a geometry of **5b** at CPCM(DCM)-SCS-MP2/cc-pVQZ level of theory.

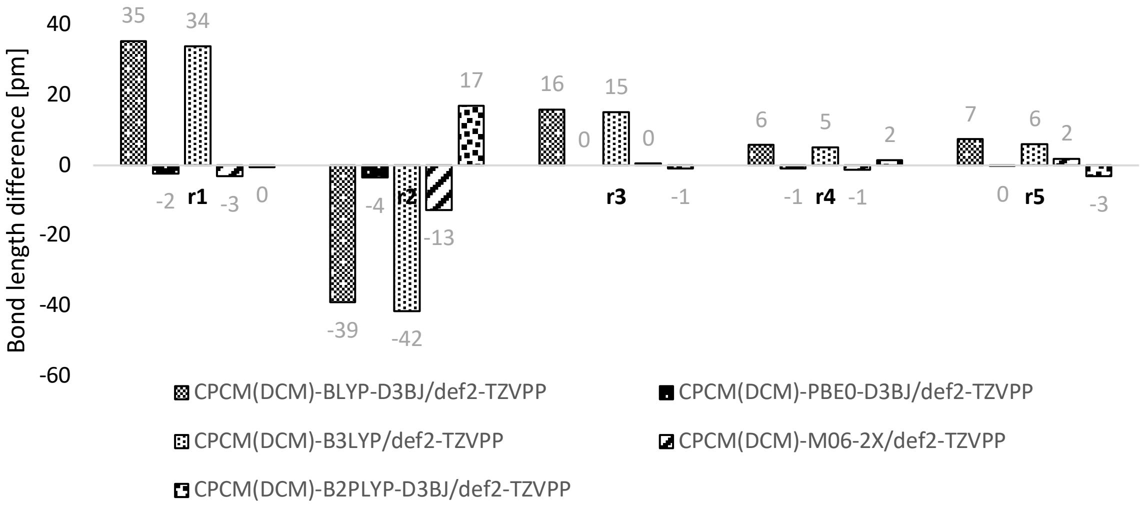

Fig. S4. Effect of the DFT functional on r_1, r_2, r_3, r_4 and r_5 relative to a geometry of **5b** obtained at CPCM(DCM)-SCS-MP2/cc-pVQZ level of theory.

15. Exploration of carbocation geometry

Different conformations of the Lewis-structure were probed, building on the results of the groups of Olah (*9*) and Siehl(*62*). The parent (unsubstituted) protonated bicyclobutane exists in an equilibrium of two different (localized) structures, a cyclopropylcarbinyl (**CC**) cation, and a bicyclobutonium (**BB**) cation for which r_2 is regarded as a bond in the latter, but not in the former (r_1 is similar in both cases). Both are classified as non-classical carbocations.(*9*)

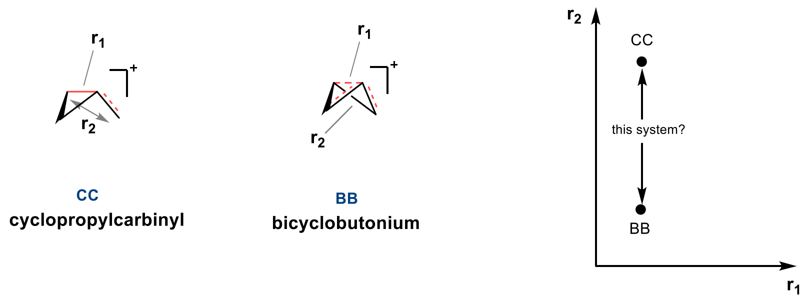

These two "extreme" geometries (**CC** and **BB**) span the geometric space of the cations studied herein, with the protonated substituted BCB structures lying between these two extremes. Therefore, geometry optimizations were initiated from a geometry resembling each of these structures. Our results suggest that **BB** is not a stationary point on the PES, instead it converges to **5b**. This excludes the hypothesis of the existence of a persistent **BB** structure (see also Section 16). When geometry optimisation is initiated from a geometry resembling the proposed Lewis-structure of **CC**, the optimised geometry also converges to **5b**. This indicates that no distinct equilibrium exists between **CC** and **BB** in the structures investigated herein.

To exclude undiscovered features of the carbocation PES, a systematic two-dimensional PES scan along the bonds r_1 and r_2 ([r_1, r_2] from [1.5 Å; 1.75 Å] to [1.5 Å; 2.0 Å]) was performed. The surface reveals the abovementioned single minimum **5b** at (1.70 Å, 1.99 Å), however, no other stationary point is observed in this [r_1, r_2] range. For the r_2 direction in particular, the PES is flat – a common characteristic of carbocation PESs. Extending the scan in the r_2 direction (1.5 to 2.4 Å) to capture potential CC cations did not locate any additional minima, with the CC states instead converging back to **5b**. The exploration of conformational flexibility is subject of Section 16.

Although r_1 in structure **5b** is too long to invoke a 'pure' BB cation, it is still noticeable shorter than the bond lengths in typical CC cations, thus placing this structure in between the two extremes. In summary, no discrete, nonclassical BB or CC carbocations were found, which is in contrast to computational investigations by Olah and co-workers on the 'parent' unsubstituted BB cation ($[C_4H_7]^+$) and the bridgehead methylated BB cation ($[C_5H_{10}]^+$).(*9*) Section 16 shows that the BB structure is, surprisingly, a saddle point rather than a minimum, for the herein investigated BCB-derived cations. The computationally obtained energy minimum **5b** can therefore be considered as a blend of the BB and CC cations, distinct from their usual equilibrium of discrete structures.

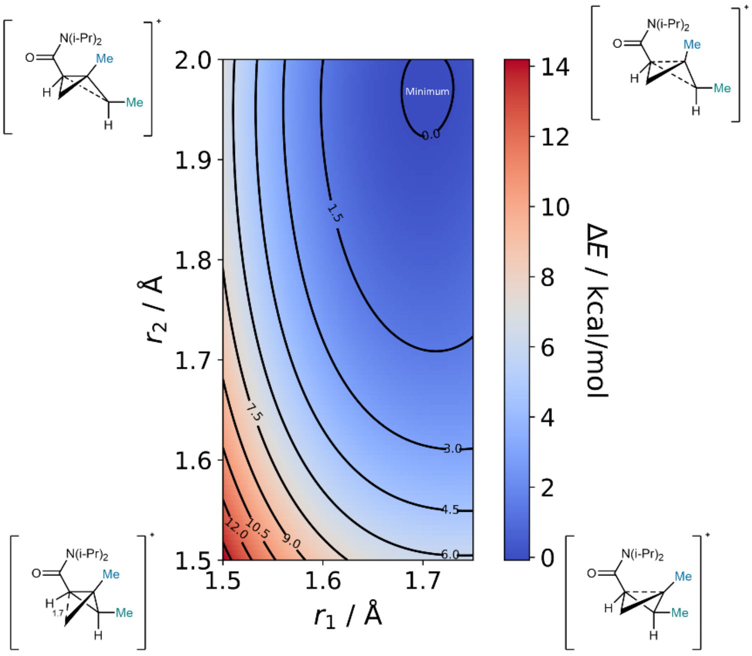

Fig. S6. More-O'Ferrall Jencks plot of **5b** varying r_1 and r_2 showing a shallow minimum at CPCM(DCM)-SCS-MP2/cc-pVTZ level of theory.

16. Structural investigation of BCB-derived non-classical carbocations

A non-classical carbocation is defined by the positive charge of a carbon-based skeleton being delocalised (via hypercoordinate bonding) rather than located on one single carbon centre. In the simplest form, this manifests as a 3-centre-2-electron bond, or, in the classical Lewis picture, results in a pentavalent carbon atom. Computationally, the nature of the bonds in the cation can be investigated *via* bond order and atomic charge analyses.

Charge and bond length analysis

Charge analysis was performed using the Hirshfeld charge scheme and was compared to Loewdin charges calculated with a cc-pVTZ, cc-pVQZ and a def2-TZVP basis set (at a CPCM(DCM)-SCS-MP2/cc-pVTZ optimised geometry). For easier comparison, the atomic charges of the hydrogen atoms are added to the carbon they are bonded to $\delta'(CH) = \delta_C + \Sigma_{1\text{-}4}\,\delta_H$. The results are shown in Fig. S7 for the system **5b**.

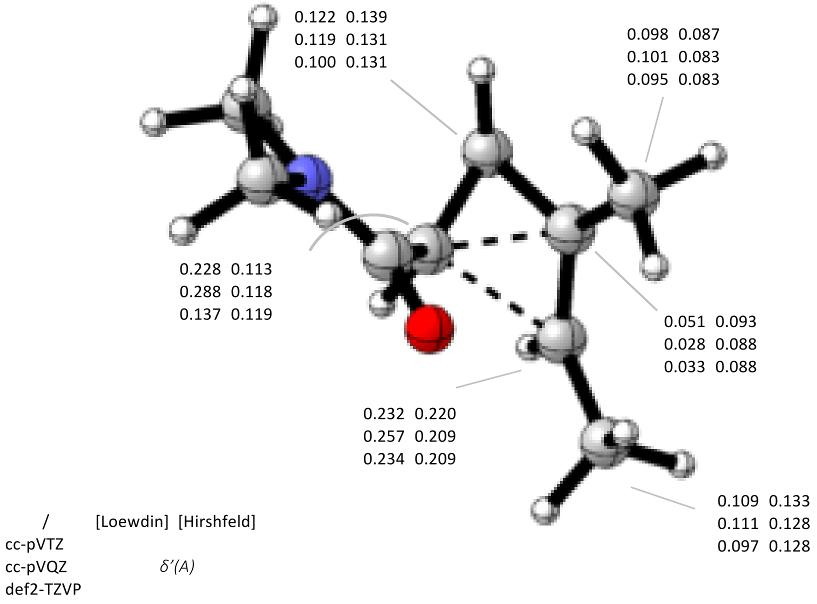

Fig. S7. Collected Loewdin, and Hirshfeld-charges δ'(A) as a sum of charge of carbon atom and directly bonded hydrogen atoms $\delta'(A) = \delta_C(A) + \Sigma\ \delta_H(A)$.

The Loewdin and Hirshfeld charges provide qualitatively similar results. Since the Hirshfeld charges showed the smallest basis set dependency (*63*), they were used further for interpretation. This was achieved by comparing the atomic charges of each cationic structure to its corresponding 'parent' bicyclobutane structure. The relative change of each individual atom reflects how the positive charge is delocalised over the molecule; the higher the value, the more (positive) charge is located on this atom (group). Fig. S8 shows the relative charges Δδ(A) for **5a** to **5e**, as well as the relative cumulated charges Δδ'(A).

The bridgehead methyl-substituted carbocation **5a** shows a highly symmetric charge distribution between both bridge carbon atoms. The reason for the subtle asymmetry in the structure can be ascribed to the conformation of the carbonyl group, which is preferentially oriented 'towards' one bridge. Significant relative charge Δδ' is distributed on the bridges (0.18-0.19), similar to the relative charge Δδ' on the bridgeheaded atom (0.16). This leads to a classic BB-type structure with partial double bonds at r_3 and r_4, and lengthened bonds at r_2 and r_5.

5b and **5e** represent *exo*- and *endo*- configurations of bridge and bridgehead methyl-disubstituted carbons. Different positive charge distributions δ(A) were found between these structures. The *endo*-isomer **5e** distributes significant portions of positive charge symmetrically on both bridges, even though one bridge is methyl-substituted whereas the other is not. For the *exo*-isomer **5b** the positive charge is less equally distributed, concentrating Δδ'(A) on the methyl-substituted bridge and significantly less on the other.

For the trimethylated cation **5c**, comparable Hirshfeld charge differences Δδ' are observed on both bridges (0.193 and 0.160). One bridge is extensively lengthened (Δδ' 0.193, lengthened r_2, shortened r_3) whereas the other one (Δδ' 0.160, r_4/r_5 only slightly distorted) stays intact.

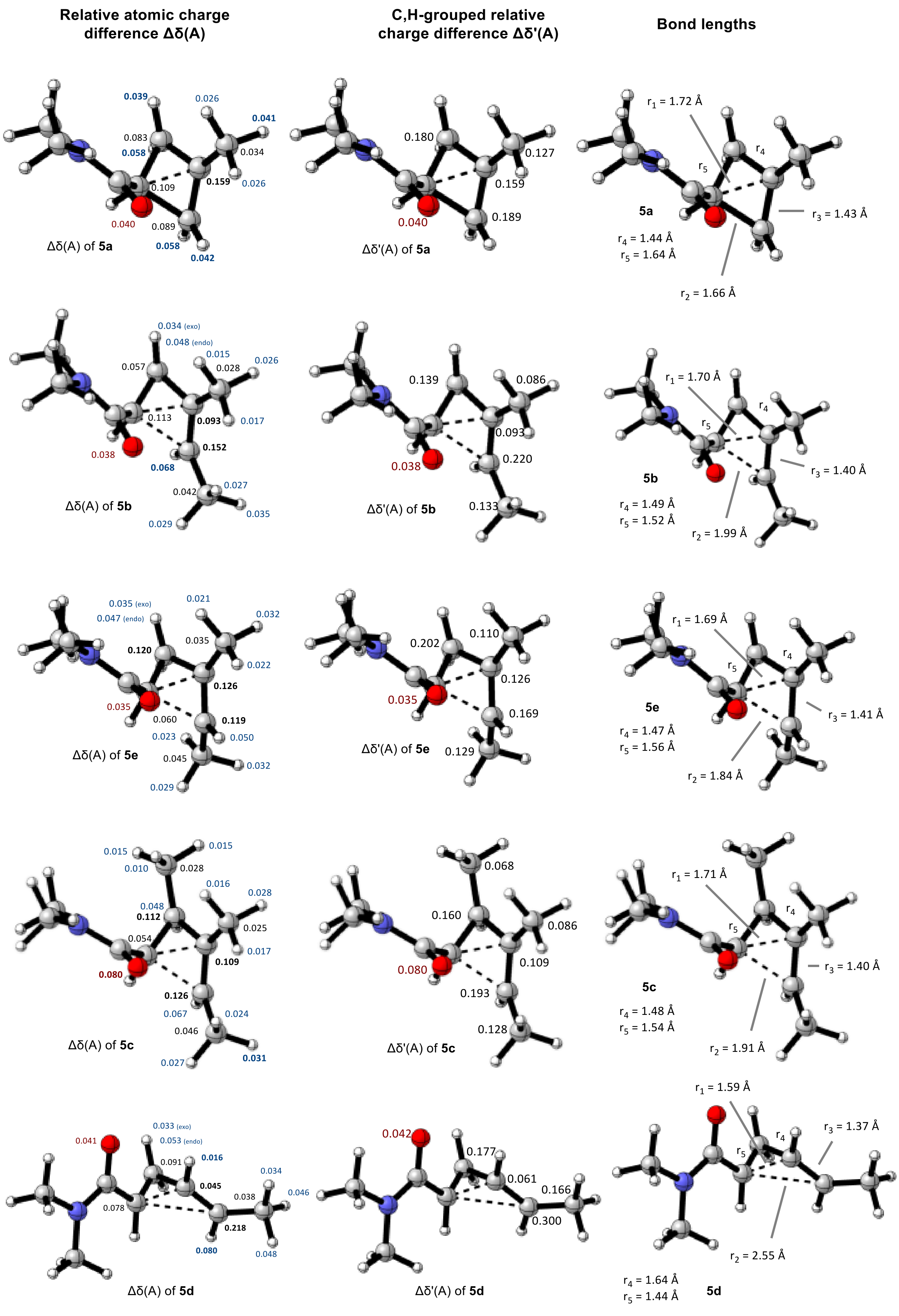

Fig. S8. Relative Hirshfeld charges Δδ(A) and Δδ'(A) at CPCM(DCM)-SCS-MP2/cc-pVTZ level of theory. Black numbers correspond to carbon-, blue to hydrogen- and red for the carbonyl oxygen-atoms. The SCF-density was used for calculated the Hirshfeld charges. Bond lengths are shown in column 3.

Cation stabilization may be enhanced by hyperconjugation effects. We did attempt to compute hyperconjugation contributions via NBO analysis, but found this to be highly basis set dependent. However, close examination of Hirshfeld charges on several of the computed cations suggests that hyperconjugation could be a contributory factor to cation stabilization; the examples shown below each feature a C–H bond antiperiplanar to a partial C–C bond where that hydrogen atom carries greater charge compared to others on the same carbon atoms.

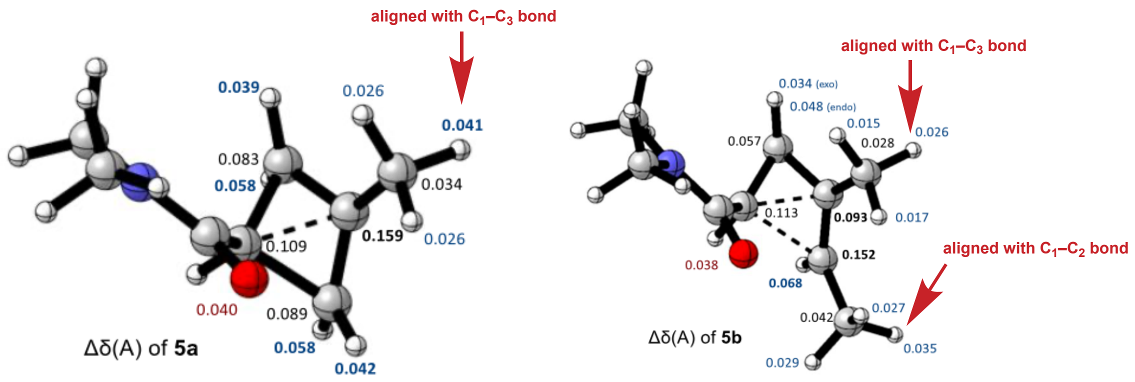

Conformational analysis

Aside from rotations of the C–C(methyl) bonds, two main axes of conformational freedom can be identified for the investigated cations: Firstly, the rotation around the bridgehead–bridge carbon (blue arrow, Fig. S9) and secondly, rotation around the bridgehead carbon–C(carbonyl) bond (red arrow, Fig. S9).

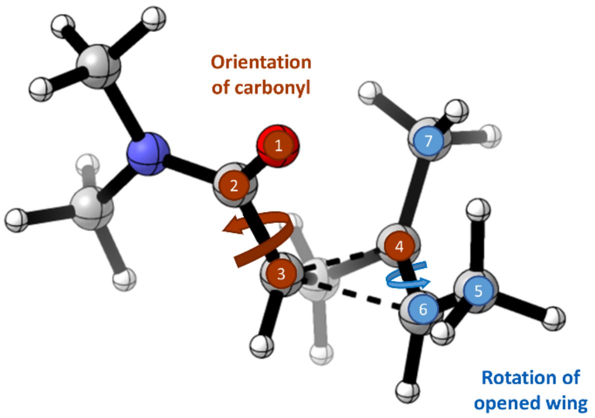

Fig. S9. Visualisation of the two investigated axis of conformational freedom. Color-coded atoms ①-② ③-④ and ⑤-⑥-④-⑦ were used for the torsional scan in ORCA.

The results of the rotational scan around ⑤-⑥-④-⑦ are discussed in the main text and the associated energy data and geometries can be found in Section 18.

The torsional scan around the carbonyl axis was performed for **5c**, and confirmed the hypothesis that the carbonyl group plays a crucial role in desymmetrisation of the structures, such that only one C1–bridge bond is lengthened and no symmetric charge distribution over the two bridge atoms is observed (see Section 16). A torsional scan of

the C1–C(carbonyl) bond revealed that the C1–bridge bond is lengthened towards which the carbonyl oxygen atom is oriented towards. Further, a symmetrical, achiral transition state **TS(5c)** was identified which interconnects these two conformers **5c** and **5c'** with an electronic activation barrier of 1.9 kcal mol^{-1} (at the CPCM(DCM)-SCS-MP2/cc-pVTZ level of theory; the TS in Fig. S10 shown differences in r_3 and r_3' with respect to r_2 and r_5 of up to 1 pm are within the error of the optimisation algorithm operating on the flat potential energy surface of the carbocation). This achiral **TS(5c)** lies geometrically in between the two enantiomers **5c** and **5c'** with shortened r_2, lengthened r_5 and slightly widened r_1 and r_3.

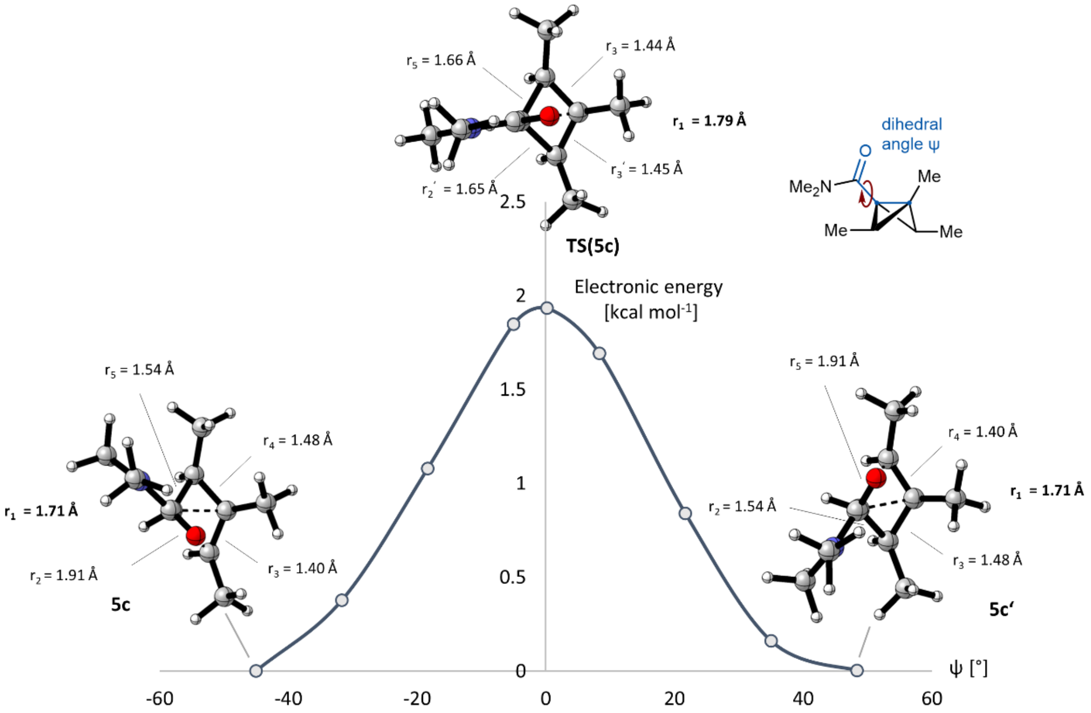

Fig. S10. Torsional scan of the carbonyl of **5c** and identification of a transition state **TS(5c)**. Calculation of electronic energies were performed at CPCM(DCM)-SCS-MP2/cc-pVTZ level of theory.

Stereoselectivity in the attack of Chloride Nucleophile

The attack of a chloride nucleophile from the *re*- and *si*-face of the bridge atom of **5b** was investigated *via* a constrained scan (Fig. S11, constrained atom distance C---Cl^- highlighted in pale grey). No transition state could be found for attack of the chloride ion, which appears to be diffusion controlled. Comparing the relative energies of the two constrained scans, no major difference in energy was found along either reaction coordinate. The two diastereomeric products *(R)* and *(S)* differ in Gibbs free energy by only $\Delta G = 0.5$ kcal mol^{-1}, although experimental evidence suggests this addition is not reversible under the reaction conditions.

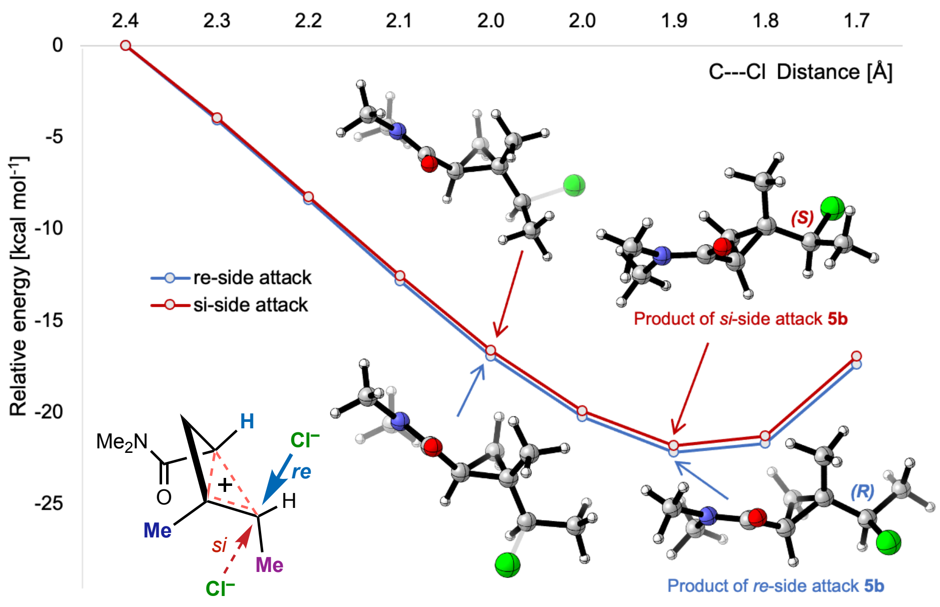

Fig. S11. Constrained scan of a chloride counter anion attacking from the *re*- and *si*-side. Optimisation and calculation of electronic energies were performed CPCM(DCM)-SCS-MP2/cc-pVDZ level of theory and depicted products optimised unconstrained CPCM(DCM)-SCS-MP2/cc-pVTZ level of theory (298.15 K / 1 M).

Further calculations were carried out as shown in Figure S12, which located a general acid protonation transition state **5h** (ΔG‡ = 3.6 kcal mol-1) for the reaction of **1b** with HCl. After this transition state, a downhill 2D-scan indicates immediate collapse of this tight ion pair into the observed major diastereomer (ΔG‡= −23.9 kcal mol^{-1}).

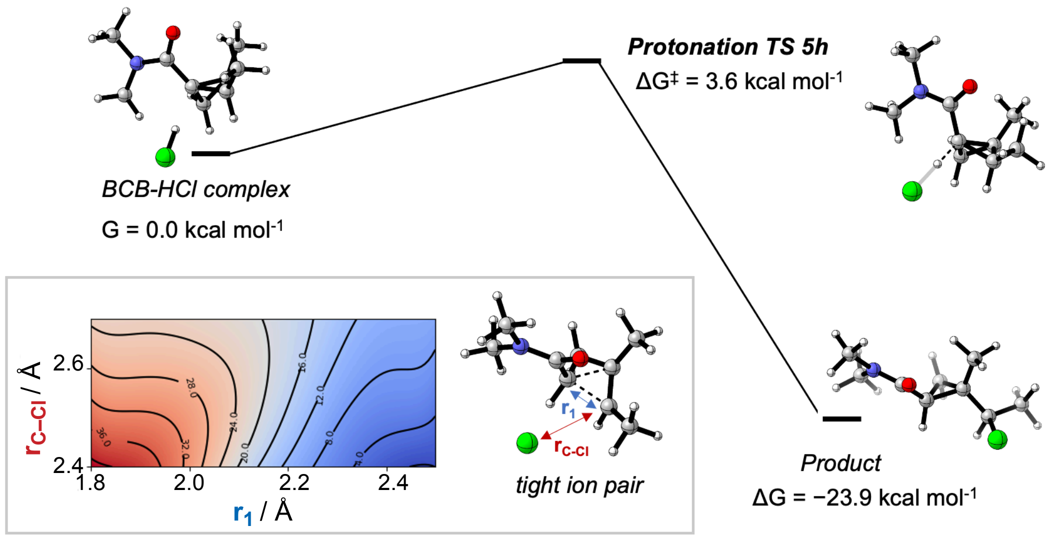

Figure S12. Protonation pathway and subsequent 2D surface showing a "downhill" pathway towards the product. Formation of the BCB-HCl complex is endotherm by 6.4 kcal mol^{-1} (see Table S2). Stationary points calculated at CPCM(DCM)-DLPNO-CCSD(T)/cc-pVQZ//CPCM(DCM)-SCS-MP2/cc-pVTZ level of theory (298.15 K / 1 M) and electronic energy surface at CPCM(DCM)-SCS-MP2/cc-pVDZ level of theory.

Table S2. Thermodynamic quantities of Figure S12**.** Thermodynamic calculations were carried out at the CPCM(DCM)-SCS-MP2/cc-pVTZ level of theory. Free energy G was calculated using the electronic energy of CPCM(DCM)-DLPNO-CCSD(T)/cc-pVQZ//CPCM(DCM)-SCS-MP2/cc-pVTZ level of theory (298.15 K / 1 M) via G = Eel(DLPNO-CCSD(T)) + [G(SCS-MP2) − Eel(SCS-MP2)].

	**Electronic energy [Ha] SCS-MP2**	**Electronic energy DLPNO-CCSD(T) [Ha]**	**Enthalpy H SCS-MP2 [Ha]**	**Free energy G [Ha]**	**ΔG [kcal mol^{-1}]**
BCB	-480.899541	-481.21187	-480.658229	-481.019664	
HCl	-460.312859	-460.366006	-460.302714	-460.377062	-6.7
Complex	-941.217448	-941.583415	-940.96423	-941.38599	0.0
TS **5h**	-941.209035	-941.57722	-940.958846	-941.380317	3.6
Product	-941.260707	-941.629289	-941.003173	-941.424143	-23.9

17. Structure of cation 5g, precursor to cyclopropane 3ag

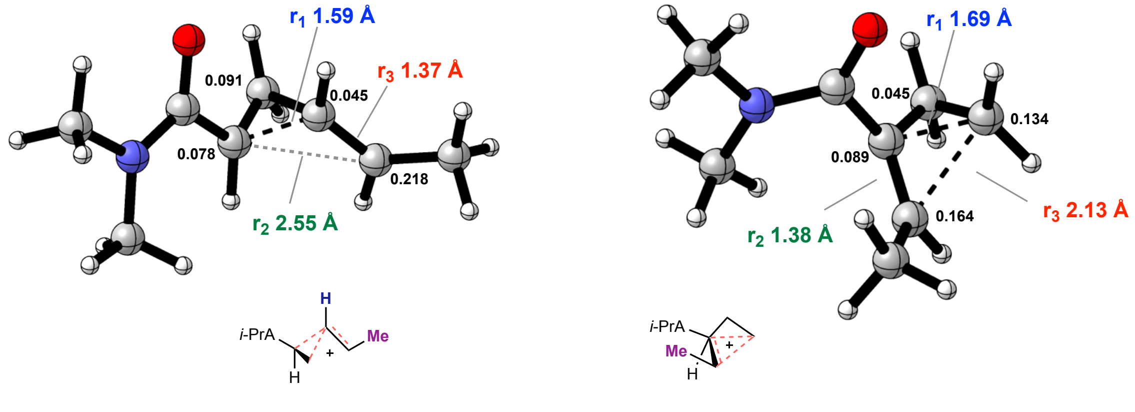

Cation **5d** (0.0 kcal mol^{-1}) Cation **5g** (0.45 kcal mol^{-1})

Fig. S13. Comparison of relative Hirshfeld charges Δδ(A) and bond lengths for the cations of **5d** and **5g** at CPCM(DCM)-SCS-MP2/cc-pVTZ level of theory as well as relative Gibbs free energies at CPCM(DCM)-DLPNO-CCSD(T)/cc-pVQZ//CPCM(DCM)-SCS-MP2/cc-pVTZ level of theory (298.15 K / 1 M).

18. Geometries and associated Hirshfeld charges

Note: Below atomic sequence is different between protonated and parental, unprotonated bicyclobutane and requires manual mapping of corresponding atoms.

Cation (dimethylated, exo), **5b**

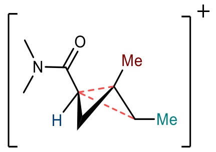

Electronic energy -481.324761357637

Number of imaginary frequencies: 0

C	2.59409700603150	0.09955016145230	-1.41691557162671
C	3.00169482056641	0.02609479427896	0.02160182575165
C	3.46873284268783	1.25039923049688	0.72196898039888
C	4.62709261916453	0.31459514492258	0.44413378499384
C	5.49874791885063	0.29010496642212	-0.80220764797508
N	6.22003325583077	1.39824836406495	-1.03135345934360
C	7.06983620867412	1.45040250537962	-2.21954044869799
C	6.38632049790526	2.50235808210278	-0.08985096754826
O	5.50676107369090	-0.71770503513798	-1.50902336558938
C	3.30987174982199	-1.14823575613430	0.71272081629304
C	3.36815997087498	-2.51631271909318	0.13697627688948
H	3.37371438273650	-1.07862406950415	1.79317641195475
H	3.07157228955066	0.94333681991460	-1.91070989767900
H	1.51708578979955	0.27908515809726	-1.41265420598691
H	2.80295481000184	-0.81265572158596	-1.96376987951023
H	3.24123702721803	1.35818750802556	1.77408290403574
H	3.40499889421423	2.15173192290149	0.12904075949804
H	6.67507755192284	0.77925460676351	-2.97388949653363

H	8.09047615670271	1.15911824913679	-1.96945736343709
H	7.07015046771760	2.47180317667500	-2.59422362063878
H	5.91285919484767	2.28359100584007	0.86089069221081
H	5.96335172774579	3.41677177546011	-0.50444561592583
H	7.45100089406035	2.65138255591229	0.08667887851424
H	3.44888088645557	-2.52302140158116	-0.94300188175736
H	2.44754646838084	-3.02387395911506	0.43953497755106
H	4.20028771293141	-3.06263542217366	0.57719313186950
H	5.10794182411526	-0.08509885882143	1.32940020148884

Atom	Hirshfeld charge
0 C	-0.03218
1 C	0.09549
2 C	0.012146
3 C	0.07875
4 C	0.250224
5 N	-0.082234
6 C	0.021193
7 C	0.026118
8 O	-0.394155
9 C	0.147631
10 C	-0.027978
11 H	0.09105
12 H	0.04949
13 H	0.056087
14 H	0.046536
15 H	0.074805

16 H	0.073398
17 H	0.038071
18 H	0.03815
19 H	0.043075
20 H	0.042891
21 H	0.042997
22 H	0.045551
23 H	0.047679
24 H	0.062162
25 H	0.056351
26 H	0.096707
TOTAL	1.000007

Bicyclobutane (dimethylated, exo), **1b**

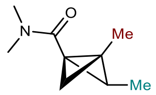

Electronic energy -480.910486585353

Number of imaginary frequencies: 0

C	2.07617504595469	0.04612750910483	-1.44804802242952
C	2.81754965514587	0.20732495629113	-0.16105876662259
C	3.39130400275756	1.44692779106066	0.45335971636863
C	4.30584210598137	0.31483163744736	0.07790267039496
C	5.34101192619155	0.24665181731623	-0.97320814748306
N	6.22667548310653	1.27444031016682	-1.02040316316523
C	7.19009553230424	1.32082588300432	-2.11179744429189
C	6.48546536133187	2.19468590260572	0.07937480893142
O	5.37304572039819	-0.67978687904048	-1.79740421798341

C	3.49172970369761	-0.80005501423736	0.71260643225053
C	3.62851856415838	-2.23793718230106	0.27154843336917
H	3.34782247763771	-0.67714100570197	1.78622779611509
H	0.99859827713131	0.05846259874576	-1.27758403489421
H	2.33606901512442	-0.89457436149678	-1.93189268384656
H	2.32205183451713	0.86143741302588	-2.13006386868258
H	3.22030105874342	1.65042433627535	1.50834365271226
H	3.42280063454971	2.32183480275715	-0.18698423119274
H	6.75603653720992	0.87365470781192	-2.99959785324265
H	8.10431493957898	0.78239438318021	-1.85277129187352
H	7.43835626855963	2.36218550825801	-2.30893940803045
H	5.90209771648046	1.91848737991392	0.94918968887139
H	6.24357812893802	3.21818387212792	-0.20932607530592
H	7.54433158355852	2.14721818804118	0.33823732085915
H	3.75471313349373	-2.31642836214614	-0.80403301446846
H	2.74337531464907	-2.80030531788672	0.57127802449575
H	4.49458048729990	-2.69776275002386	0.74826984364447

Atom	Hirshfeld charge
0 C	-0.060322
1 C	0.002035
2 C	-0.044459
3 C	-0.034721
4 C	0.16992
5 N	-0.064733
6 C	-0.006258

7 C	-0.000837
8 O	-0.355755
9 C	-0.004022
10 C	-0.069609
11 H	0.022842
12 H	0.029643
13 H	0.031395
14 H	0.032417
15 H	0.026376
16 H	0.039014
17 H	0.031142
18 H	0.032786
19 H	0.038759
20 H	0.036951
21 H	0.035357
22 H	0.037308
23 H	0.020299
24 H	0.027246
25 H	0.027115
TOTAL	-0.00011

Cation (dimethylated, endo), **5e**

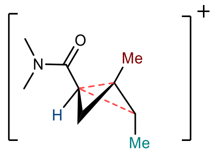

Electronic energy -481.322561068656

Number of imaginary frequencies: 0

C	2.60446806836483	0.14032756045460	-1.38432672437497
C	2.98545166969962	0.16046654068340	0.06061777734699
C	3.45789815792156	1.39848028716563	0.68639762869142
C	4.61390503029709	0.37846736665222	0.46102615011599
C	5.50153783585461	0.29413408675988	-0.77289498960408
N	6.22516423693533	1.38712348495360	-1.05860410277955
C	7.07558882255239	1.37473000359108	-2.24770462161927
C	6.38637315980664	2.54546137342168	-0.18388321699998
O	5.51050163501114	-0.74853999248713	-1.42770952120766
C	3.42689593033452	-0.99644432777204	0.73353516472926
H	3.57156341240562	-1.86070409207224	0.09551138953918
C	3.34996019492245	-1.25887552118305	2.20808677292082
H	3.05324519729868	0.96919862637359	-1.92785061980034
H	1.52036153629000	0.27669897891334	-1.39681772771908
H	2.85238212946220	-0.80631302379413	-1.85391607357849
H	3.27796024534275	1.58012912369123	1.73606259651239
H	3.42863382853368	2.26988744678223	0.04798995254702
H	6.67510819964694	0.67191763933482	-2.96946731026387
H	8.09371510566264	1.08698096533956	-1.98379886010245
H	7.08457255089755	2.37688142865901	-2.67085799456010
H	5.91943029381645	2.37963722402575	0.78037976350535
H	5.95482768404452	3.43104080044473	-0.64935491530303

H	7.45055682349831	2.71260270624222	-0.02169603990019
H	5.10857359018014	0.14538877660392	1.39644180913222
H	3.25360161637225	-0.34981390929092	2.79566202865036
H	4.21900675610180	-1.82380644205388	2.53864257661372
H	2.46387637664615	-1.87518198513914	2.36815964870835

Atom	Hirshfeld charge
0 C	-0.027688
1 C	0.12563
2 C	0.020965
3 C	0.065377
4 C	0.24901
5 N	-0.082511
6 C	0.021112
7 C	0.026035
8 O	-0.398469
9 C	0.114335
10 H	0.079387
11 C	-0.023913
12 H	0.052763
13 H	0.062062
14 H	0.050238
15 H	0.077149
16 H	0.075371
17 H	0.03795
18 H	0.038097
19 H	0.043045

20 H	0.043026
21 H	0.042849
22 H	0.045749
23 H	0.097812
24 H	0.051188
25 H	0.054974
26 H	0.058567
TOTAL	1.000109

Bicyclobutane (dimethylated, endo), **1o**

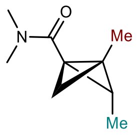

Electronic energy -480.900615008335

Number of imaginary frequencies: 0

C	2.33049092974453	0.05483755525931	-1.39458663428111
C	2.95206524292456	0.17844549531440	-0.04033324345309
C	3.48066487871299	1.41385870217739	0.61796863341426
C	4.44783842950677	0.31320935072569	0.26323499461568
C	5.44100835367253	0.25573274196163	-0.82916796918766
N	6.19275318202640	1.36593162012633	-1.04756469118218
C	7.08802684253254	1.38580779536201	-2.19665616458085
C	6.42725311250517	2.41208037501319	-0.05989533118021
O	5.54069688579367	-0.75482341882719	-1.54069321834471
C	3.64814241180465	-0.86985282527378	0.76046122465917
C	3.41629554795634	-1.10617370991792	2.23831865518278
H	2.54995049031324	0.93799067901768	-1.99735765818099
H	1.24664118202134	-0.04261892110802	-1.31851725115718

H	2.71984345936426	-0.82351219118684	-1.91040776051816
H	3.27225634575514	1.62055755221027	1.66218979732968
H	3.50831605815106	2.29527422178285	-0.01392947129869
H	6.66172727000239	0.79411703809910	-2.99960720827024
H	8.07013399509168	0.98186817813968	-1.93982227390495
H	7.20702804223939	2.41734015811900	-2.52424593766469
H	5.94709521942026	2.16237413782358	0.87865540851025
H	6.05184949762197	3.37301004822071	-0.41443369897038
H	7.50140698947713	2.50117687952121	0.11170077993033
H	3.23664712673802	-0.17747954264335	2.77682697142392
H	4.28507963454220	-1.59426110768696	2.68228728338273
H	2.55155547385417	-1.75555898155457	2.38384301839230
H	3.83165131572742	-1.77779133447540	0.19224897103400

Atom	Hirshfeld charge
0 C	-0.063099
1 C	0.005493
2 C	-0.039936
3 C	-0.038404
4 C	0.167478
5 N	-0.066432
6 C	-0.006274
7 C	-0.000961
8 O	-0.363091
9 C	-0.005056
10 C	-0.068588

11 H	0.03164
12 H	0.029821
13 H	0.027767
14 H	0.03043
15 H	0.040508
16 H	0.031336
17 H	0.032358
18 H	0.03873
19 H	0.036691
20 H	0.035497
21 H	0.037452
22 H	0.027828
23 H	0.025836
24 H	0.026154
25 H	0.029778
TOTAL	0.002954

Rotation endo-exo TS, **5f**

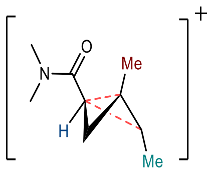

Electronic energy -481.287147298430

Number of imaginary frequencies: 1

Lowest imaginary frequency: -187.5 cm^{-1}

C	2.05188918999351	0.61782548352681	0.96610110752733
C	2.70368953759656	-0.53696897482808	0.17316283396599

C	3.13098168796480	-0.28468119258405	-1.25335753678268
C	2.00180709742313	-1.25654665122720	-0.93376616052116
C	0.54545002512713	-0.96157295926129	-1.20958742591131
N	0.18769974136092	-0.77910141747806	-2.49731707606714
C	-1.20673526427245	-0.49388588964475	-2.81636713556590
C	1.04774356072948	-1.02747232627894	-3.64723528614038
O	-0.25511489476252	-0.90838576699629	-0.26971705636082
C	3.54730672234672	-1.24110106676906	1.10806538374173
C	4.87072697813319	-0.82535334438228	1.45723121474891
H	3.16140282275444	-2.14670911204078	1.57413435977970
H	1.40312786345112	1.14899714455829	0.27364279502912
H	2.81773471986423	1.29684302361461	1.33705402770699
H	1.44873415756286	0.24533268814992	1.79104295558018
H	4.06207735196428	-0.70634284689289	-1.60554327931821
H	2.86680212137060	0.68443682373188	-1.65385976510888
H	-1.69466598675861	-0.06590448020168	-1.94822541893296
H	-1.72798661067402	-1.40610271956050	-3.11164616213194
H	-1.23552881233540	0.21314545826780	-3.64367276600533
H	2.02508505824062	-1.37401920509697	-3.33453692123851
H	1.16001235093933	-0.11496399206196	-4.23332283097458
H	0.59163200251282	-1.79264632797222	-4.27661861755960
H	5.14748308055006	0.16750300300280	1.12098021338416
H	5.40748285219858	-1.57180672278015	0.81634906866060
H	5.16262512865292	-1.08263074824801	2.47264849842777
H	2.26632141206565	-2.30251136054695	-1.04857492493303

Trimethylated bicyclobutonium **5c**

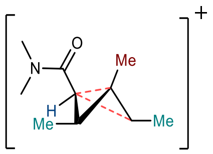

Electronic energy -520.550581370378

Number of imaginary frequencies: 0

C	2.41895452142746	-0.02957291109121	-1.41638890306151
C	2.93269626467343	0.02223164124603	-0.01375960441122
C	3.36974082715695	1.29063033336449	0.61909591501776
C	4.57132493431952	0.36613537353228	0.33577662771828
C	5.41792714832611	0.35090250107984	-0.92783308648332
N	6.29055166125846	1.36631217074307	-1.04286342723947
C	7.16236131865682	1.41418825993932	-2.21496298345814
C	6.59043306217121	2.34272760984353	-0.00068919206377
O	5.28993385597693	-0.55811680694632	-1.74836752120178
C	3.39566398041887	-1.09193907153979	0.70258908129284
C	3.55538336589681	-2.47320074051981	0.16689602856792
H	3.45886325545930	-0.98738841451709	1.77982014680267
H	2.86426505307040	0.72859398026449	-2.05191042856218
H	1.35085794761916	0.18755943473808	-1.33131450231018
H	2.54826297363165	-1.00588346174653	-1.86797802799056
H	3.19960683446540	1.34175916771196	1.68927436195430
C	3.18686327468665	2.60254045936266	-0.10758946501622
H	6.69787441232351	0.87245709089859	-3.03100482401121
H	8.13112558249161	0.96856636514845	-1.98612422957805
H	7.30420873485702	2.45625458025435	-2.49353118200444
H	5.95864949228099	2.19975545120051	0.86894326384227
H	6.43750684991030	3.34879515724303	-0.38882764277881
H	7.63155420625618	2.23408295334819	0.30404842655202

H	3.65182956591320	-2.49888941301806	-0.91167604776977
H	2.67328206050773	-3.04193877439144	0.47162182811288
H	4.42390109964623	-2.94235171110826	0.62532270058829
H	5.10822169960158	0.09104886300381	1.23642289961698
H	3.57367299217791	2.57087758056746	-1.12344667228087
H	3.70689944548351	3.38995420445788	0.43374871357103
H	2.12767337083497	2.85511821233045	-0.14349075491572

Atom	Hirshfeld charge
0 C	-0.032446
1 C	0.108602
2 C	0.037846
3 C	0.066102
4 C	0.25041
5 N	-0.082776
6 C	0.020527
7 C	0.025772
8 O	-0.387497
9 C	0.127896
10 C	-0.030764
11 H	0.087457
12 H	0.04829
13 H	0.057035
14 H	0.048311
15 H	0.068398
16 C	-0.038235

17 H	0.037574
18 H	0.037539
19 H	0.04251
20 H	0.043521
21 H	0.043733
22 H	0.043431
23 H	0.04443
24 H	0.057969
25 H	0.053116
26 H	0.096214
27 H	0.03719
28 H	0.043498
29 H	0.044323
TOTAL	0.999975

Bicyclobutane, Trimethylated **1m**

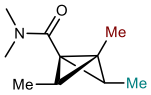

Electronic energy -520.126497442367

Number of imaginary frequencies: 0

C	1.93852517269685	-0.16530800575385	-1.33328856094227
C	2.81759650827284	0.07407668204890	-0.14818351520430
C	3.32447285738711	1.35686142700895	0.45472594756627
C	4.29541764713217	0.30979598968320	-0.02180648218239
C	5.31107390971438	0.34143977459998	-1.09739402891213
N	6.26613899222671	1.30148727984908	-0.99575649982212
C	7.25351093257710	1.42270058787153	-2.05940126333365

C	6.58005842747766	2.01722682054802	0.23270308709187
O	5.27549588377981	-0.44683877711075	-2.05374825471758
C	3.64178669996469	-0.87885059080276	0.65697705178249
C	3.88909276471043	-2.28231014804472	0.15617935536799
H	1.99497697756074	0.66542776803192	-2.03653104634551
H	0.89812391091988	-0.27580983928900	-1.02154702023135
H	2.23445828717954	-1.07286529869070	-1.85829511678005
H	3.22126845276941	1.47113526126271	1.53539990930212
C	3.18751808939773	2.63984909027954	-0.33246351258974
H	6.80107176936721	1.15007201378121	-3.00702340261054
H	8.11292440463610	0.77336045542018	-1.87667234298022
H	7.59491784822236	2.45579891990647	-2.09797003173948
H	5.85876045985706	1.77452600958212	1.00452551585627
H	6.57064249871823	3.09276684023204	0.05358394870941
H	7.57610335550721	1.73179834596570	0.57822201085031
H	4.00275311771880	-2.30103827700434	-0.92467855042988
H	3.06136088884946	-2.93435386331896	0.43887490566133
H	4.79992554908504	-2.68681554743394	0.59923201075087
H	3.36074937747319	2.47129513770418	-1.39545761661502
H	3.90732940271390	3.38206702057437	0.01385652539564
H	2.18835663022935	3.06071864141805	-0.21158208225573
H	3.56974954225487	-0.79335637111916	1.74225677385743

Atom	Hirshfeld charge
0 C	-0.057867
1 C	-0.004296

2 C	-0.0162
3 C	0.007003
4 C	0.162873
5 N	-0.060576
6 C	-0.004278
7 C	0.008526
8 O	-0.307429
9 C	0.002226
10 C	-0.077216
11 H	0.031924
12 H	0.029051
13 H	0.030868
14 H	0.020538
15 C	-0.066234
16 H	0.031493
17 H	0.032761
18 H	0.039287
19 H	0.037469
20 H	0.036797
21 H	0.036049
22 H	0.020175
23 H	0.02697
24 H	0.026603
25 H	0.027549
26 H	0.028153
27 H	0.029021
28 H	0.020676

TOTAL 0.091915

Bridgehead-methylated cation ***5a***

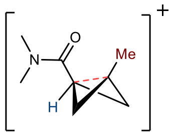

Electronic energy -442.094653055011

Number of imaginary frequencies: 0

C	2.51175047630198	0.09384191641778	-1.41415888560476
C	2.91130883552619	0.08755600861320	0.01702260249651
C	3.33466014501524	1.28751276456666	0.68967655359998
C	4.56584831592147	0.23591962603449	0.45337822997136
C	5.44599954630219	0.23209705467920	-0.78900856324888
N	6.20128856549584	1.32293325211106	-0.99135734373524
C	7.05266197002359	1.38091669685338	-2.17842753335622
C	6.35696904806277	2.43897196488215	-0.06326179042573
O	5.42153042852332	-0.75242404803461	-1.52855047901677
C	3.52277777044217	-1.03705114655019	0.65227475204944
H	3.42943549764226	-1.16430756935422	1.72149124389146
H	2.85965433700171	0.98866302252763	-1.92558573729271
H	1.41771880691022	0.12805398978560	-1.38294626212292
H	2.82653544836888	-0.80672919059764	-1.92961396147458
H	6.68047073699874	0.68635125969629	-2.92242260047577
H	8.07973872768300	1.12345029336236	-1.91834289661357
H	7.02688478975722	2.39548572356546	-2.57092593830467
H	5.86064646921534	2.24628737430989	0.88040940609668
H	5.95159337150198	3.34920442322222	-0.50446123376153

H	7.41874064459286	2.58363011129095	0.13299760054370
H	5.08033747334870	0.23460411356614	1.40703746179295
H	3.20057295394195	1.37718135552263	1.75885440161292
H	3.31458926111343	2.20403429791738	0.11684616503019
H	3.68196513610878	-1.92208579698783	0.05456374784817

Atom	Hirshfeld charge
0 C	-0.022764
1 C	0.172493
2 C	0.043934
3 C	0.062519
4 C	0.248062
5 N	-0.08296
6 C	0.020798
7 C	0.025847
8 O	-0.39471
9 C	0.048005
10 H	0.088141
11 H	0.059634
12 H	0.071252
13 H	0.054598
14 H	0.037675
15 H	0.038341
16 H	0.042266
17 H	0.043816
18 H	0.042741
19 H	0.045761

20 H	0.106376
21 H	0.088294
22 H	0.081428
23 H	0.078428
TOTAL	0.999977

*Bicyclobutane, bridgehead-methylated **1e***

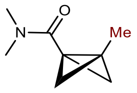

Electronic energy -441.675271423974

Number of imaginary frequencies: 0

C	2.31329664579609	0.01208154613820	-1.42984456517702
C	2.94209934153920	0.11297385045502	-0.07847311006578
C	3.43691108851770	1.32446101048334	0.64578286488403
C	4.42333482817825	0.26171201088449	0.23860124769226
C	5.43584939056338	0.23838049851312	-0.83506803164392
N	6.17683041859817	1.36096100062768	-1.02009477523277
C	7.08688939263925	1.41576640208166	-2.15636164431976
C	6.37859188690303	2.39385057521143	-0.01155387815528
O	5.55653812518294	-0.75777040036391	-1.56310065618819
C	3.63787980592082	-0.93526729147214	0.71782419750964
H	3.45036163441597	-1.00627568595434	1.78567861290286
H	2.50068343241009	0.92054642112770	-2.00453279926190
H	1.23392997702194	-0.12250577274877	-1.34902202466770
H	2.72673446200284	-0.83585873252689	-1.97655146284723
H	6.68367641056055	0.82713155793545	-2.97330835410993
H	8.07331305267713	1.02820605505465	-1.89131595177276

H	7.18926033477704	2.45414940423262	-2.46756631342751
H	5.89182326352164	2.11837977577103	0.91628432391434
H	5.98913925851918	3.35354164474176	-0.35400541260906
H	7.44854249413097	2.50021447606662	0.17512565530948
H	3.22012050207727	1.44145129200966	1.70485139326927
H	3.44647539028560	2.24581943555566	0.07429667159287
H	3.83106014616078	-1.87245569012409	0.21080549000407

Atom	Hirshfeld charge
0 C	-0.056583
1 C	0.013718
2 C	-0.038723
3 C	-0.046028
4 C	0.167219
5 N	-0.067436
6 C	-0.005936
7 C	0.00003
8 O	-0.354496
9 C	-0.040583
10 H	0.029975
11 H	0.033259
12 H	0.030362
13 H	0.028663
14 H	0.031528
15 H	0.032686
16 H	0.038873
17 H	0.037138

18 H	0.035713
19 H	0.037782
20 H	0.030445
21 H	0.041939
22 H	0.036428
TOTAL	0.015974

*Bridge-methylated cation **5d***

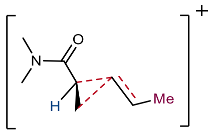

Electronic energy -442.098010459817

Number of imaginary frequencies: 0

C	3.63494103967442	-0.55164691632171	-0.13017048875750
C	2.91874946487550	0.90750258235680	0.08753383693183
C	4.35252492691727	0.85537485791524	0.06189651368288
C	5.04261745613918	1.24891067705224	-1.23355111983333
N	6.29474840827416	1.71597407329479	-1.11793535173307
C	6.98557143225114	2.21242724175021	-2.30736010215690
C	7.02483681384352	1.81522032598140	0.14979873377872
O	4.42025827204096	1.13417167309003	-2.29161454950503
C	3.47691557077236	-1.36174458741328	0.96064943529067
C	3.23757496449132	-2.81055247463402	0.89699163396241
H	3.52232076097406	-0.90529975405792	1.94557534808374
H	6.31611295066187	2.15104399999897	-3.15671248192637
H	7.87371299083971	1.60953581849336	-2.49262710205859
H	7.28371618503144	3.24732193675509	-2.14500618499559
H	6.63721661615320	2.62094660671334	0.77212315697103

H	8.06223995546529	2.03015171820537	-0.08872615337028
H	6.99484887020010	0.87623036847225	0.69669762751196
H	3.18815807433901	-3.18470010224570	-0.12024829155465
H	2.31688947534445	-3.03592538623380	1.44173743084299
H	4.04065281951095	-3.30573411412364	1.45188843877827
H	4.86968354968574	0.93405779869169	1.00358620063589
H	2.40154505610930	1.00698043140718	1.02872239701278
H	2.38627451956722	1.13896710112864	-0.82325364281733
H	3.59555840033738	-0.92463208537654	-1.14063976267451

Atom	Hirshfeld charge
0 C	0.001685
1 C	0.065622
2 C	0.04106
3 C	0.252778
4 N	-0.082182
5 C	0.020428
6 C	0.027341
7 O	-0.405652
8 C	0.228917
9 C	-0.014143
10 H	0.105405
11 H	0.036787
12 H	0.038523
13 H	0.039356
14 H	0.041073
15 H	0.051435
16 H	0.043359
17 H	0.060688
18 H	0.073604
19 H	0.075579
20 H	0.072444
21 H	0.082285
22 H	0.077904
23 H	0.065754

TOTAL	1.000049

Bridge-methylated cation ***5g***

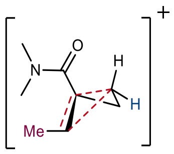

Electronic energy -442.096885115810

C	2.65371564909671	1.34081838755254	0.42702749629042
C	3.81232279723367	0.43745894566352	0.10986868672034
C	4.59115169156694	0.69373076059100	-1.17309913550660
N	5.88874260950437	0.97763218511457	-0.99391218375962
C	6.73693162378256	1.20911879024494	-2.16150807771232
C	6.53487653735470	1.17293646667583	0.30095252886581
O	4.01606642358970	0.64534272297016	-2.26104920908612
C	3.90040191652113	-0.75449164472846	0.80768865252944
C	4.74325569024591	-1.90595322395274	0.41281113714890
H	6.24491644621756	0.81845880824387	-3.04464479490339
H	7.68496211384648	0.69749699463945	-2.00626469476463
H	6.92099185299440	2.27645908729798	-2.28385408456732
H	5.80388597293381	1.22298053769617	1.10161042986665
H	7.07843688326154	2.11638530170864	0.27783946519621
H	7.23743270526450	0.36317183110139	0.49720793726629
H	5.17348317189000	-1.79746355701857	-0.57825036336825
H	4.15942528325093	-2.82372987256990	0.47783427801515
H	5.54356884786014	-1.98699259986830	1.15506053632918
C	2.18505523222792	0.05966648038169	-0.15493497400840
H	2.60778156490084	2.21144065036659	-0.21149757781747

H	2.42924905074826	1.49381922877478	1.47330922452154
H	3.35592494531826	-0.83435736201120	1.74270725250602
H	2.10849421859287	-0.06002955630758	-1.22571503227790
H	1.65685946099665	-0.63790733666636	0.47878601771606

Atom	Hirshfeld charge
0 C	0.018852
1 C	0.051451
2 C	0.25159
3 N	-0.079377
4 C	0.022556
5 C	0.024463
6 O	-0.393499
7 C	0.174652
8 C	-0.023174
9 H	0.039129
10 H	0.042938
11 H	0.039929
12 H	0.040448
13 H	0.04763
14 H	0.042476
15 H	0.051045
16 H	0.066351
17 H	0.070696
18 C	0.091093
19 H	0.076031
20 H	0.076171
21 H	0.101129
22 H	0.075254
23 H	0.092194
TOTAL	1.000028

*Bicyclobutane, bridge-methylated **1n***

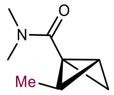

Electronic energy -441.672738034836

Number of imaginary frequencies: 0

C	2.59673671719034	-0.22551685845886	-0.09589685356421
C	2.43518005607416	1.20637243569249	0.27615828127594
C	3.81613939027540	0.67098612716812	0.00028770714833
C	4.65099428961681	1.04199221891029	-1.16826405386394
N	5.99526713017269	0.97310459386383	-1.00750371744430
C	6.85016630864193	1.27758425642061	-2.14717907557833
C	6.66738019754999	0.93911923570702	0.28403572681224
O	4.13114145907946	1.38477651640094	-2.23857459824136
C	3.73389329300647	-0.59384701427389	0.81229462266895
C	4.59599467346430	-1.77685808240170	0.43848910908156
H	6.35629754308095	0.97135964759577	-3.06314135514179
H	7.78520443260403	0.73204136450037	-2.03277494123378
H	7.06858051462992	2.34650735385267	-2.20118477960797
H	5.94200652602601	0.85206927576614	1.08465286561674
H	7.23497944829365	1.86172666811415	0.42229751100178
H	7.35837068780457	0.09703732164362	0.32700414165123
H	4.72794082085465	-1.83388874908284	-0.64254149689226
H	4.13046383092130	-2.70290764637345	0.77655446060006
H	5.57944799284933	-1.70741438062119	0.90261713519962
H	2.19114748451795	1.45472052073913	1.30521273965774
H	2.01984681446467	1.86246169264825	-0.47812465253221
H	2.29354607132158	-0.71425082927332	-1.00643505482006
H	3.56141309365966	-0.46989832103815	1.88109129980602

Atom	Hirshfeld charge
0 C	-0.042937

1 C	-0.025797
2 C	-0.037105
3 C	0.238935
4 N	-0.095532
5 C	0.011678
6 C	0.016213
7 O	-0.445731
8 C	0.010493
9 C	-0.051762
10 H	0.030786
11 H	0.03522
12 H	0.028317
13 H	0.035315
14 H	0.032382
15 H	0.033596
16 H	0.026195
17 H	0.028027
18 H	0.027467
19 H	0.029171
20 H	0.039291
21 H	0.049977
22 H	0.025849
TOTAL	0.000046

(S)-chloride-addition product of **5b**

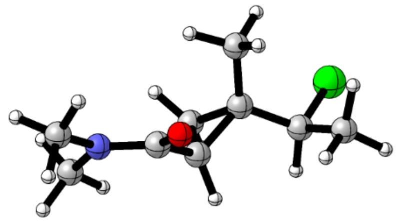

Electronic energy -941.261390822221

Number of imaginary frequencies: 0

C	2.77388489010649	-0.12718015520300	-1.44966108279831
C	3.27993846303581	-0.14967512906834	-0.02471238648194
C	3.63363782189778	1.16943101630662	0.61169464809494

C	4.73148861447170	0.19985983088785	0.24570237281776
C	5.66179801211541	0.37436699434484	-0.92497721038562
N	6.27868588169837	1.56967624708383	-1.08286311512135
C	7.14358410835855	1.75963236142405	-2.24079229863974
C	6.40130040495733	2.58436053484720	-0.04406911521905
O	5.83477327699238	-0.56181376878820	-1.71554607936833
C	2.72731997246371	-1.21702962353145	0.88650338296291
C	2.88718862270997	-2.63133405499825	0.35678544841722
H	3.18806557526997	-1.12367316272509	1.86809791581745
H	3.19823904276357	0.72036086270639	-1.98718795718346
H	1.68806638310714	-0.02465764050578	-1.45897988507482
H	3.04674997546730	-1.03399912076902	-1.98727771682926
H	3.42296364534125	1.30947626657508	1.66367696035223
H	3.51725921953462	2.04930728456609	-0.00658250093414
H	6.75228336377204	1.19801078316781	-3.08236389244707
H	8.16164561852787	1.42400141206154	-2.03168959854309
H	7.16457892378929	2.82039710612772	-2.48391469396371
H	5.89053873295605	2.27201565398311	0.85807687114327
H	5.98326463082255	3.53143044096622	-0.38723092052782
H	7.45750567888197	2.73377443069266	0.18649857713148
H	2.32127054220958	-2.77075917262787	-0.56241437365420
H	2.54372472690003	-3.35678048088551	1.09197753172939
H	3.94314227235437	-2.81146883480822	0.14814897860930
H	5.19224939740160	-0.33218305756634	1.07031814697924
Cl	0.95592052649267	-0.90041964516401	1.20376144401678

(R)-chloride-addition product of **5b**

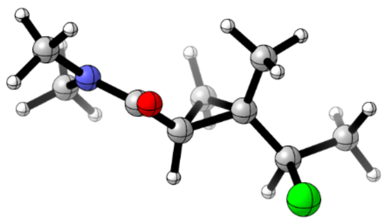

Electronic energy -941.260706971035

Number of imaginary frequencies: 0

C	3.25590735850439	-0.61806902938165	-1.59215816373974
C	3.86509065189068	-0.60830557402429	-0.20809086654935
C	3.89548283506570	0.71363417014130	0.52861770641260
C	5.17898457863916	0.09850918147265	0.02425922034604
C	5.95288905610789	0.63420764045649	-1.15168090550442
N	6.28695694533243	1.94739607447856	-1.14764075147486
C	7.01359631050858	2.48588078447033	-2.29056291652302
C	6.24571596374749	2.81146548562194	0.02446544679802
O	6.26193839984922	-0.11637922872787	-2.08482885834544
C	3.61705845331862	-1.81474386585441	0.66371670334199
C	2.15252889509781	-2.18073924263853	0.83280511016514
H	4.07917769732231	-1.65258211775669	1.63584175627755
H	3.50355328812481	0.30063560254869	-2.12176833353500
H	2.16934226495283	-0.68673721591536	-1.53090183062232
H	3.62802014248010	-1.45666972356940	-2.17982081805252
H	3.72848827332839	0.72145156563862	1.59758754588308
H	3.50741269540269	1.56937737441580	-0.00780058548447
H	6.69864772606198	1.97605934727755	-3.19469768654602
H	8.09144218211068	2.35897778176579	-2.16797912330603
H	6.79027288394645	3.54816247400275	-2.37057661520933
H	5.85317161539662	2.27716096560402	0.88043808140270
H	5.62367763408044	3.68559516581054	-0.17184074771650

H	7.25684058468984	3.15012573913031	0.25746771589458
H	1.70748895283954	-2.47211383306654	-0.11655100044588
H	1.61400234616884	-1.31524804302099	1.22280285123423
H	2.04537262668700	-3.00527447003397	1.53528374754984
H	5.81639482425014	-0.35248462902676	0.77671062495391
Cl	4.50954320349465	-3.25578513251894	-0.01467189640471

TS(5c)

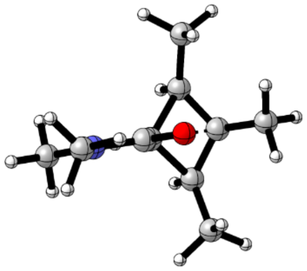

Electronic energy -941.261390822221

Number of imaginary frequencies: 2

Imaginary frequencies: -187.5 cm^{-1} (and -15.8 cm^{-1})

Note: The second small residual frequency corresponds to CH_3-rotation of the methyl-amide.

C	1.99757614863044	-0.26629463762565	-1.30615970555754
C	2.74391356984322	0.01798985155355	-0.08182000600195
C	3.11153555519493	1.33094069175493	0.44243384615439
C	4.51973302569717	0.53194798622494	0.39210843390379
C	5.26165175276404	0.65377608885650	-0.91796850703831
N	6.43887165759543	1.29758053097017	-0.88620295079304
C	7.25320044861963	1.36948273977326	-2.09797291079761
C	6.99400505307428	1.94702270354501	0.30341246789532
O	4.75238872880334	0.17358864501594	-1.93803430370061
C	3.68337818913413	-0.85252222500021	0.60511228118025
C	4.09573419122376	-2.19074377425582	0.04819686585742
H	3.54457270056216	-0.84421697601727	1.68394565529789
H	1.93813670351240	0.57881661867522	-1.98313945738919
H	0.98708853943704	-0.43317353930151	-0.89097252834523
H	2.31609301547142	-1.17442443057240	-1.80390596651093
H	2.92390627117359	1.42761264405139	1.50908880750288
C	2.86378258518988	2.58966275090672	-0.35095909448809
H	7.29554602729583	2.39740304525501	-2.45735100951745

H	6.81641305048565	0.73331567016926	-2.85838283445546
H	8.26224438301380	1.03101219893125	-1.86699699004158
H	6.22032533267651	2.46006699052637	0.86744929686292
H	7.70985703733707	2.69220693265897	-0.03316265500520
H	7.50874179682147	1.23126783564133	0.94446252421568
H	4.31078120536692	-2.12643228027058	-1.01473121589274
H	3.29769816671375	-2.91485436126175	0.21095356447356
H	4.98429050251011	-2.54036961469147	0.57089613790999
H	5.07278352788936	0.72243410803077	1.30105670526038
H	3.16912467879121	2.47080682754673	-1.38903358200682
H	3.42531869866050	3.41172591439289	0.08917856913652
H	1.80458477011046	2.84367387201636	-0.32324167600917

BCB-HCl complex

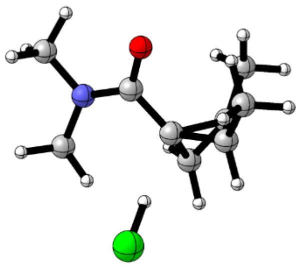

Electronic energy -941.217448132937

C	7.90133864617993	8.97803877153520	5.98355933317865
C	7.98094936866443	9.14735276018650	7.46615313623810
C	8.52656560759428	10.30158401656724	8.23698472228742
C	9.21671650084958	8.96277318343897	8.35115270527962
C	10.51233568627863	8.52834673192739	7.78219071500719
N	11.55842854267929	9.38714793231102	7.90336371842866
C	12.81559870201963	9.03428423596081	7.25505989852076
C	11.64827448629784	10.48354538738766	8.85978936174969

O	10.62354952961644	7.44800121940093	7.18614852387967
C	7.93922492601501	8.13402912505906	8.55142983251275
C	7.87249736821653	6.65567629766387	8.25495549400559
H	7.39094738634100	8.42710239873161	9.44728185395072
H	8.60549565091252	9.64978830733070	5.49029824845208
H	6.89805343366319	9.21505194036476	5.62661206978221
H	8.13886801146584	7.95589519874570	5.69249876178121
H	7.98529138146368	10.66646889772256	9.10690354038447
H	9.03480904494019	11.06145318519079	7.65462640222634
H	12.61335953011633	8.47849301583441	6.34577629669027
H	13.44059069263228	8.42547624835960	7.91224276198790
H	13.34778630217779	9.95288647870834	7.01422118475583
H	10.74917056247448	10.54758255250957	9.45936440509510
H	11.80185313814107	11.43022091722146	8.34020052065160
H	12.49716881275933	10.30899907615732	9.52337818570176
H	8.39005063257304	6.40409299319808	7.33537125059618
H	6.82817174503630	6.35013707034573	8.17937671989441
H	8.32993225277465	6.08968054718097	9.06697410151324
H	9.52495730052560	8.52745214397045	10.39692118309599
Cl	9.79247475758887	8.28615936698735	11.63908507235072

BCB-HCl transition state 5h

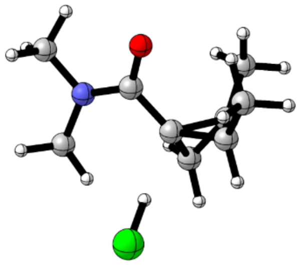

Electronic energy -941.209034822547

C	8.09389772514444	9.00604140880277	6.10584920186122
C	7.99108129159885	9.11587736190450	7.59201264431613
C	8.51173102805567	10.23891517429686	8.37317429289787
C	9.26137768779612	8.89235842276086	8.57415542529245
C	10.50915326246452	8.48780683834595	7.84559077425618
N	11.54998342079790	9.34753475461702	7.91892673324832
C	12.76589374760881	9.03402470346011	7.17593035509712
C	11.66451852486848	10.47136508359677	8.84246231000874
O	10.56063321530764	7.42529529270554	7.21764405069306
C	7.89433799806480	8.03775228676655	8.55443028027763
C	7.81651751023652	6.58207180820736	8.18399188197774
H	7.39037639758948	8.28458912450417	9.48588622072972
H	8.85376960082299	9.68724496404773	5.72383012950304
H	7.13068102180366	9.29856093649996	5.68387517735419
H	8.32315928414741	7.99300782592384	5.78833015239291
H	7.98709893395845	10.55803317433794	9.26776855964847
H	9.01554583401671	11.01716399994494	7.81489722186084
H	12.51824994546394	8.41875118898844	6.31815954436901
H	13.47891848075986	8.50134688399957	7.80783301969943
H	13.21634281323149	9.96763049819518	6.84350097301321
H	10.83421142899081	10.49490028316951	9.53682462798703
H	11.70963041164591	11.41171973316055	8.29183527372168
H	12.58417427029701	10.35800698908960	9.41745509097131
H	8.30177471911837	6.36521953765901	7.23994365146103
H	6.76513105104879	6.29703737100375	8.13107850131645
H	8.28830384248561	5.98373991347170	8.96258959600192

H 9.39857076639773 8.81080517866339 9.90301206244273

Cl 9.54939578627645 8.75091926187511 11.46093224759930

19. Thermodynamic data

Table S3. Raw energy values of constrained scan of Chloride-attack (see Section S16 / Fig. S11)

C-Cl distance [Å]	Relative energy *re*-Side [kcal mol^{-1}]	Relative energy *si*-side [kcal mol^{-1}]
2.40	0.00	0.00
2.31	-4.05	-3.96
2.22	-8.42	-8.24
2.13	-12.83	-12.58
2.04	-16.92	-16.62
1.96	-20.25	-19.90
1.87	-22.16	-21.79
1.78	-21.69	-21.29
1.69	-17.37	-16.95

Table S4. Conformational scan identifying **TS(5c)** (see Section S16 / Fig. S9 and Fig. S10)

Torsional angle ①-② ③-④ [°]	Electronic energy [Ha]	Relative energy [kcal mol^{-1}]
-45.0	-520.550581	0.0
-31.7	-520.549980	0.4
-18.3	-520.548861	1.1
-5.0	-520.547637	1.8
0.2	-520.547501	1.9
8.3	-520.547884	1.7
21.7	-520.549242	0.8
35.0	-520.550326	0.2
48.3	-520.550577	0.0

Table S5. Thermodynamic quantities of **5b**, **5e**, **5f**; and **5g**, **5g**. Thermodynamic calculations were carried out at the CPCM(DCM)-SCS-MP2/cc-pVTZ level of theory. Free energy G was calculated using the electronic energy of CPCM(DCM)-DLPNO-CCSD(T)/cc-pVQZ//CPCM(DCM)-SCS-MP2/cc-pVTZ level of theory via G = Eel(DLPNO-CCSD(T)) + [G(SCS-MP2) − Eel(SCS-MP2)].

	Electronic energy [Ha] SCS-MP2	Electronic energy DLPNO-CCSD(T) [Ha]	Enthalpy H SCS-MP2 [Ha]	Free energy G [Ha]	ΔG rel [kcal mol^{-1}]
**5b**	-481.324761	-481.638419	-481.070983	-481.435109	
**5e**	-481.322561	-481.636394	-481.068844	-481.432127	1.9[a]
**5f**	-481.287147	-481.603781	-481.036528	-481.402784	20.3[a]
**5d**	-442.098010	-442.381929	-441.873865	-442.205974	
**5g**	-442.096885	-442.380624	-441.872882	-442.205263	0.45[b]

[a] ΔG rel [kcal mol^{-1}] is relative to cation **5b.** [b] ΔG rel [kcal mol^{-1}] is relative to cation **5d.**

Raw data of 2D scan

1.75000000 2.00000000 -481.32437008

1.75000000 1.87500000 -481.32393903

1.75000000 1.75000000 -481.32267373

1.75000000 1.62500000 -481.32032183

1.75000000 1.50000000 -481.31485202

1.68750000 2.00000000 -481.32470467

1.68750000 1.87500000 -481.32434900

1.68750000 1.75000000 -481.32291917

1.68750000 1.62500000 -481.32011972

1.68750000 1.50000000 -481.31453175

1.62500000 2.00000000 -481.32338955

1.62500000 1.87500000 -481.32312075

1.62500000 1.75000000 -481.32158770

1.62500000 1.62500000 -481.31852440

1.62500000 1.50000000 -481.31276847

1.56250000 2.00000000 -481.31995127

1.56250000 1.87500000 -481.31976767

1.56250000 1.75000000 -481.31817308

1.56250000 1.62500000 -481.31486394

1.56250000 1.50000000 -481.30883152

1.50000000	2.00000000	-481.31373652
1.50000000	1.87500000	-481.31364314
1.50000000	1.75000000	-481.31203395
1.50000000	1.62500000	-481.30846517
1.50000000	1.50000000	-481.30208443

Comparison of Loewdin and Hirshfeld charges

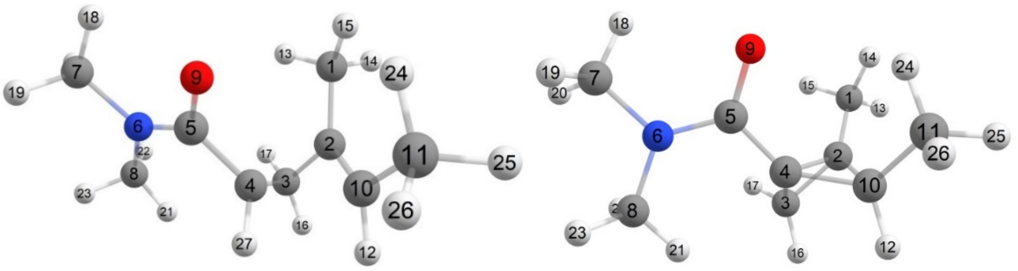

Fig. S14. Mapping of atomic sequence in protonated bicyclobutane **5b** (left) and the unprotonated bicyclobutane **1b**. All other structures were mapped similarly manually.

20. X-ray crystallography

Crystallographic data have been deposited with the CCDC as entries CCDC 2240514 - 2240521

Table S6. Crystal data and structure refinement for **3h.**

Empirical formula	C17 H27 F3 N2 O4	
Formula weight	380.41	
Temperature	150 K	
Wavelength	1.54184 Å	
Crystal system	Monoclinic	
Space group	P 21/c	
Unit cell dimensions	a = 9.5839(2) Å	α = 90°.
	b = 19.9028(5) Å	β = 100.972(2)°.
	c = 1984.05(8) Å	γ = 90°.
Volume	1350.32(9) $Å^3$	
Z	4	
Density (calculated)	1.273 Mg/m^3	
Absorption coefficient	0.928 mm^{-1}	
F(000)	808	
Crystal size	0.22 x 0.20 x 0.17 mm^3	
Theta range for data collection	4.443 to 76.257°.	
Index ranges	-11<=h<=9, -24<=k<=20, -13<=l<=10	
Reflections collected	11137	
Independent reflections	4111 [R(int) = 0.018]	
Completeness to theta = 74.731°	99.5 %	
Absorption correction	Semi-empirical from equivalents	
Max. and min. transmission	0.85 and 0.70	
Refinement method	Full-matrix least-squares on F^2	
Data / restraints / parameters	4110 / 334 / 273	
Goodness-of-fit on F^2	0.9957	
Final R indices [I>2sigma(I)]	R1 = 0.0397, wR2 = 0.0945	
R indices (all data)	R1 = 0.0435, wR2 = 0.0984	
Extinction coefficient	33(5)	
Largest diff. peak and hole	0.37 and -0.29 e.$Å^{-3}$	

Table S7. Crystal data and structure refinement for **3m.**

Empirical formula	C19 H27 N3 O6	
Formula weight	393.44	
Temperature	150 K	
Wavelength	1.54184 Å	
Crystal system	Monoclinic	
Space group	P 21/c	
Unit cell dimensions	a = 21.2948(5) Å	α = 90°.
	b = 8.7337(2) Å	β = 90.084(2)°.
	c = 11.3459(2) Å	γ = 90°.
Volume	2101.84(8) $Å^3$	
Z	4	
Density (calculated)	1.243 Mg/m^3	
Absorption coefficient	0.774 mm^{-1}	
F(000)	840	
Crystal size	0.22 x 0.20 x 0.08 mm^3	
Theta range for data collection	4.169 to 76.269°.	
Index ranges	-26<=h<=27, -10<=k<=10, -13<=l<=14	
Reflections collected	21551	
Independent reflections	4354 [R(int) = 0.018]	
Completeness to theta = 74.744°	99.7 %	
Absorption correction	Semi-empirical from equivalents	
Max. and min. transmission	0.94 and 0.84	
Refinement method	Full-matrix least-squares on F^2	
Data / restraints / parameters	4353 / 0 / 254	
Goodness-of-fit on F^2	0.9918	
Final R indices [I>2sigma(I)]	R1 = 0.0470, wR2 = 0.1275	
R indices (all data)	R1 = 0.0534 wR2 = 0.1391	
Extinction coefficient	90(12)	
Largest diff. peak and hole	0.32 and -0.26 e.$Å^{-3}$	

Table S8. Crystal data and structure refinement for **3n.**

Empirical formula	C22 H34 N2 O5 S	
Formula weight	438.59	
Temperature	150 K	
Wavelength	0.71073 Å	
Crystal system	Monoclinic	
Space group	P $2_{1/c}$	
Unit cell dimensions	a = 14.6520(4) Å	α = 90°.
	b = 10.4813(2) Å	β = 114.474(3)°.
	c = 10.4813(2) Å	γ = 90°.
Volume	2332.19(11) $Å^3$	
Z	4	
Density (calculated)	1.249 Mg/m^3	
Absorption coefficient	0.173 mm^{-1}	
F(000)	944	
Crystal size	0.20 x 0.18 x 0.13 mm^3	
Theta range for data collection	3.055 to 29.160°.	
Index ranges	-20<=h<=20, -14<=k<=14, -22<=l<=23	
Reflections collected	48054	
Independent reflections	6646 [R(int) = 0.042]	
Completeness to theta = 25.298°	99.3 %	
Absorption correction	Semi-empirical from equivalents	
Max. and min. transmission	0.98 and 0.87	
Refinement method	Full-matrix least-squares on F^2	
Data / restraints / parameters	6194 / 0 / 271	
Goodness-of-fit on F^2	1.0144	
Final R indices [I>2sigma(I)]	R1 = 0.0409, wR2 = 0.0913	
R indices (all data)	R1 = 0.0589, wR2 = 0.1046	
Absolute structure parameter	-0.032(12)	
Extinction coefficient	20(4)	
Largest diff. peak and hole	0.38 and -0.47 e.$Å^{-3}$	

Table S9. Crystal data and structure refinement for **3p.**

Empirical formula	C14 H25 F3 N2 O3 S	
Formula weight	358.42	
Temperature	150 K	
Wavelength	1.54184 Å	
Crystal system	Monoclinic	
Space group	Cc	
Unit cell dimensions	a = 11.9581(3) Å	α = 90°.
	b = 14.3089(3) Å	β = 101.375(3).
	c = 10.9176(3) Å	γ = 90°.
Volume	1831.39(8) $Å^3$	
Z	4	
Density (calculated)	1.300 Mg/m^3	
Absorption coefficient	1.965 mm^{-1}	
F(000)	760	
Crystal size	0.27 x 0.07 x 0.03 mm^3	
Theta range for data collection	4.877 to 76.112°.	
Index ranges	-14<=h<=14, -17<=k<=17, -13<=l<=12	
Reflections collected	9964	
Independent reflections	3283 [R(int) = 0.041]	
Completeness to theta = 74.590°	99.8 %	
Absorption correction	Semi-empirical from equivalents	
Max. and min. transmission	0.94 and 0.67	
Refinement method	Full-matrix least-squares on F^2	
Data / restraints / parameters	3281 / 230 / 223	
Goodness-of-fit on F^2	1.0005	
Final R indices [I>2sigma(I)]	R1 = 0.0370, wR2 = 0.0960	
R indices (all data)	R1 = 0.0386, wR2 = 0.0990	
Absolute structure parameter	-0.065(12)	
Extinction coefficient	21(3)	
Largest diff. peak and hole	0.16 and -0.18 e.$Å^{-3}$	

Table S10. Crystal data and structure refinement for **3x.**

Empirical formula	C20 H28 N2 O4 S	
Formula weight	392.52	
Temperature	300 K	
Wavelength	1.54184 Å	
Crystal system	Monoclinic	
Space group	P 2_1/c	
Unit cell dimensions	a = 10.94120(10) Å	α = 90°.
	b = 15.4773(2) Å	β = 100.2166(11)°.
	c = 12.5974(2) Å	γ = 90°.
Volume	2099.42(5) $Å^3$	
Z	4	
Density (calculated)	1.242 Mg/m^3	
Absorption coefficient	1.591 mm^{-1}	
F(000)	840	
Crystal size	0.25 x 0.19 x 0.09 mm^3	
Theta range for data collection	4.106 to 76.200°.	
Index ranges	-13<=h<=13, -17<=k<=19, -15<=l<=15	
Reflections collected	53838	
Independent reflections	4384 [R(int) = 0.039]	
Completeness to theta = 76.200°	99.8 %	
Absorption correction	Semi-empirical from equivalents	
Max. and min. transmission	0.87 and 0.80	
Refinement method	Full-matrix least-squares on F^2	
Data / restraints / parameters	4382 / 0 / 244	
Goodness-of-fit on F^2	1.0031	
Final R indices [I>2sigma(I)]	R1 = 0.0457, wR2 = 0.1252	
R indices (all data)	R1 = 0.0501, wR2 = 0.1299	
Largest diff. peak and hole	0.40 and -0.62 e.$Å^{-3}$	

Table S11. Crystal data and structure refinement for **3y.**

Empirical formula	C20 H28 N2 O4 S	
Formula weight	392.52	
Temperature	150 K	
Wavelength	1.54184 Å	
Crystal system	Monoclinic	
Space group	P 21/c	
Unit cell dimensions	a = 10.31090(10) Å	α = 90°.
	b = 15.87850(10) Å	β = 90°.
	c = 12.40960(10) Å	γ = 90°.
Volume	2026.61(3) $Å^3$	
Z	4	
Density (calculated)	1.286 Mg/m^3	
Absorption coefficient	1.648 mm^{-1}	
F(000)	840	
Crystal size	0.22 x 0.20 x 0.10 mm^3	
Theta range for data collection	4.299 to 76.068°.	
Index ranges	-12<=h<=12, -18<=k<=19, -15<=l<=13	
Reflections collected	14443	
Independent reflections	4194 [R(int) = 0.021]	
Completeness to theta = 74.547°	99.8 %	
Absorption correction	Semi-empirical from equivalents	
Max. and min. transmission	0.85 and 0.75	
Refinement method	Full-matrix least-squares on F^2	
Data / restraints / parameters	4194 / 0 / 245	
Goodness-of-fit on F^2	1.0019	
Final R indices [I>2sigma(I)]	R1 = 0.0298, wR2 = 0.0781	
R indices (all data)	R1 = 0.0310, wR2 = 0.0792	
Extinction coefficient	48(4)	
Largest diff. peak and hole	0.34 and -0.35 e.$Å^{-3}$	

Table S12. Crystal data and structure refinement for **3z.**

Empirical formula	C20 H29 N5 O S	
Formula weight	387.55	
Temperature	100 K	
Wavelength	0.68890 Å	
Crystal system	Triclinic	
Space group	P -1	
Unit cell dimensions	a = 10.9405(2) Å	α = 103.7063(13)°.
	b = 14.6165(2) Å	β = 104.5995(13)°.
	c = 14.6449(2) Å	γ = 96.1543(13)°.
Volume	1350.32(9) $Å^3$	
Z	4	
Density (calculated)	1.188 Mg/m^3	
Absorption coefficient	0.153 mm^{-1}	
F(000)	832	
Crystal size	0.06 x 0.02 x 0.01 mm^3	
Theta range for data collection	1.723 to 36090°.	
Index ranges	-18<=h<=18, -24<=k<=23, -25<=l<=24	
Reflections collected	43534	
Independent reflections	19183 [R(int) = 0.046]	
Completeness to theta = 33.622°	93.7 %	
Absorption correction	Semi-empirical from equivalents	
Max. and min. transmission	1.00 and 0.73	
Refinement method	Full-matrix least-squares on F^2	
Data / restraints / parameters	19183 / 0 / 487	
Goodness-of-fit on F^2	0.9980	
Final R indices [I>2sigma(I)]	R1 = 0.0471, wR2 = 0.0906	
R indices (all data)	R1 = 0.0703, wR2 = 0.0963	
Absolute structure parameter	-0.032(12)	
Extinction coefficient	20(4)	
Largest diff. peak and hole	0.59 and -0.49 e.$Å^{-3}$	

Table S13. Crystal data and structure refinement for **3aa.**

Empirical formula	C20 H29 N5 O S	
Formula weight	387.55	
Temperature	150 K	
Wavelength	1.54184 Å	
Crystal system	Monoclinic	
Space group	P $2_{1/n}$	
Unit cell dimensions	a = 6.40000(10) Å	α = 90°.
	b = 25.5799(3) Å	β = 95.4338(10)°.
	c = 12.54350(10) Å	γ = 90°.
Volume	2044.29(4) $Å^3$	
Z	4	
Density (calculated)	1.259 Mg/m^3	
Absorption coefficient	1.557 mm^{-1}	
F(000)	832	
Crystal size	0.23 x 0.20 x 0.19 mm^3	
Theta range for data collection	3.456 to 75.967°.	
Index ranges	-6<=h<=7, -37<=k<=30, -15<=l<=15	
Reflections collected	14954	
Independent reflections	4203 [R(int) = 0.018]	
Completeness to theta = 74.447°	99.5 %	
Absorption correction	Semi-empirical from equivalents	
Max. and min. transmission	0.74 and 0.73	
Refinement method	Full-matrix least-squares on F^2	
Data / restraints / parameters	4202 / 0 / 244	
Goodness-of-fit on F^2	0.9993	
Final R indices [I>2sigma(I)]	R1 = 0.0287, wR2 = 0.0717	
R indices (all data)	R1 = 0.0306, wR2 = 0.0735	
Largest diff. peak and hole	0.28 and -0.20 e.$Å^{-3}$	

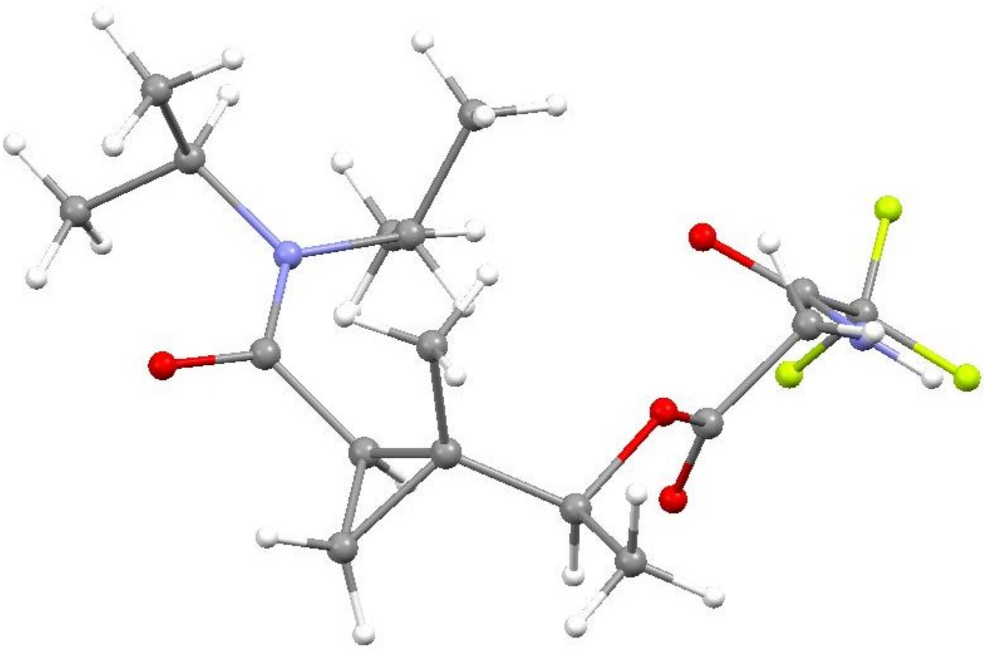

Fig. S15. Solid state structure of **3h**. Displacement ellipsoid plots are drawn at 50% probability. Hydrogen atoms are hidden for clarity.

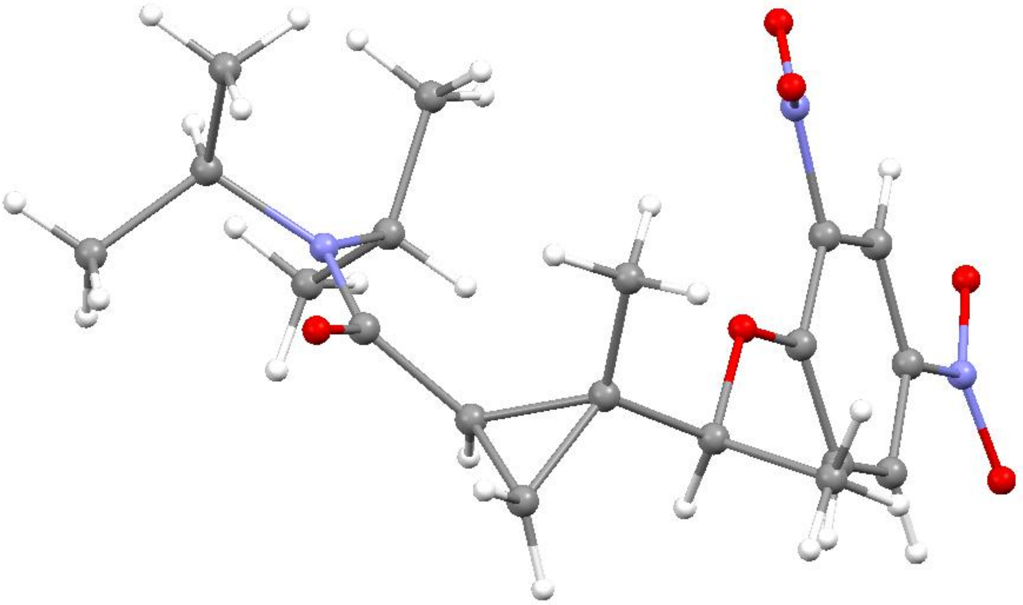

Fig. S16. Solid state structure of **3m**. Displacement ellipsoid plots are drawn at 50% probability. Hydrogen atoms are hidden for clarity.

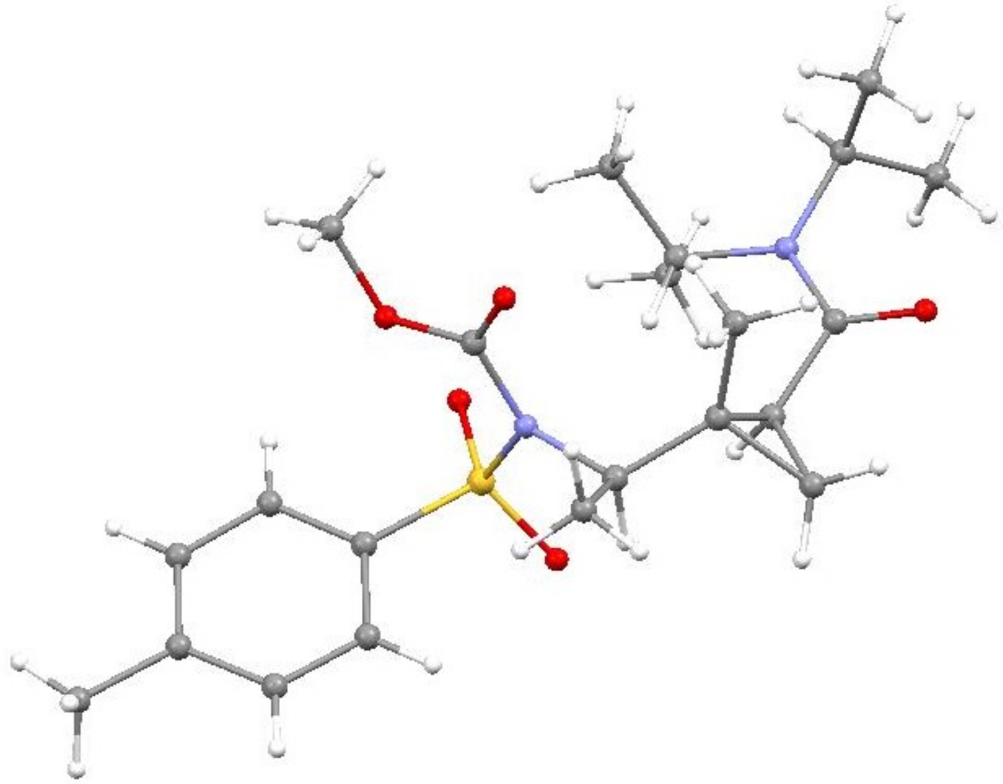

Fig. S17. Solid state structure of **3n**. Displacement ellipsoid plots are drawn at 50% probability. Hydrogen atoms are hidden for clarity.

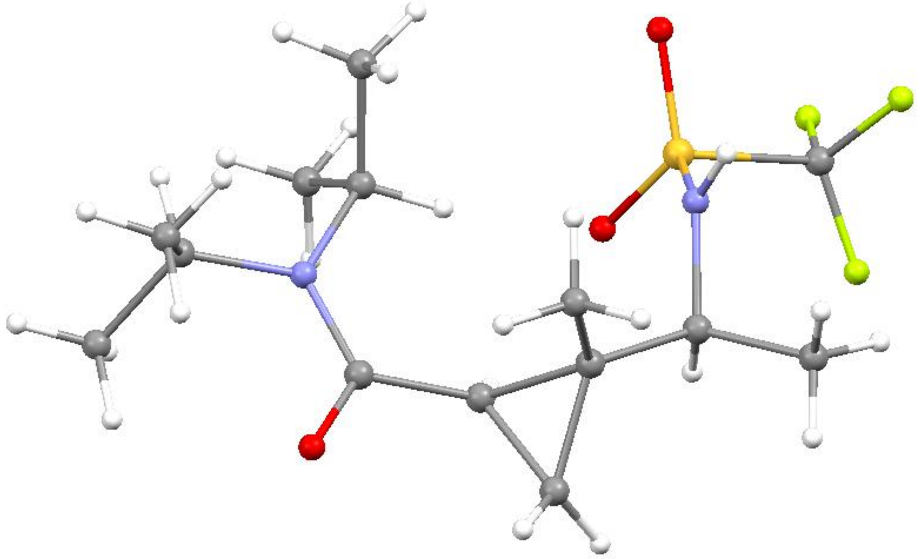

Fig. S18. Solid state structure of **3p**. Displacement ellipsoid plots are drawn at 50% probability. Hydrogen atoms are hidden for clarity.

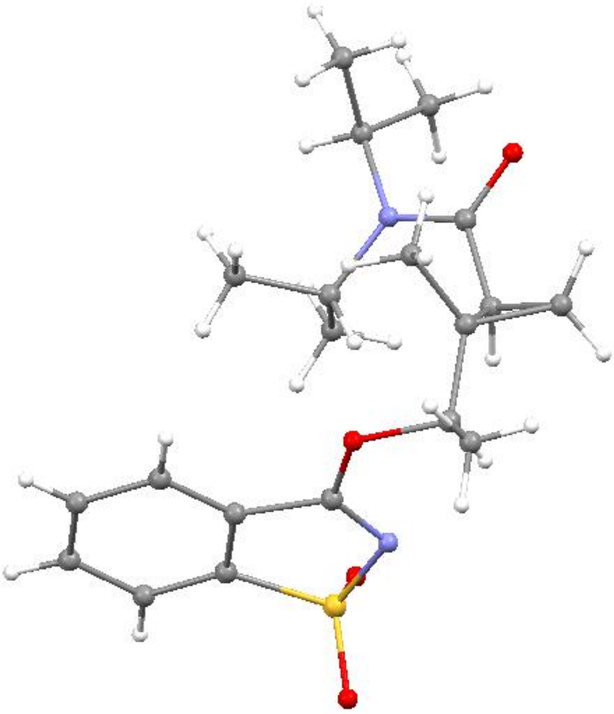

Fig. S19. Solid state structure of **3x**. Displacement ellipsoid plots are drawn at 50% probability. Hydrogen atoms are hidden for clarity.

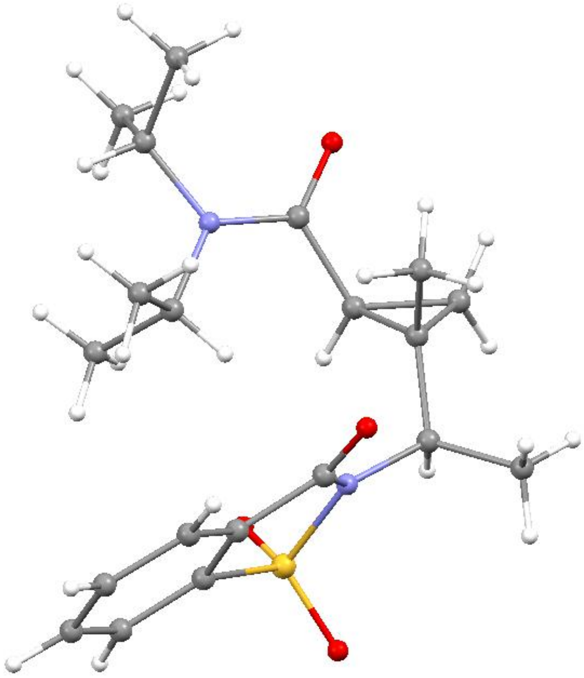

Fig. S20. Solid state structure of **3y**. Displacement ellipsoid plots are drawn at 50% probability. Hydrogen atoms are hidden for clarity.

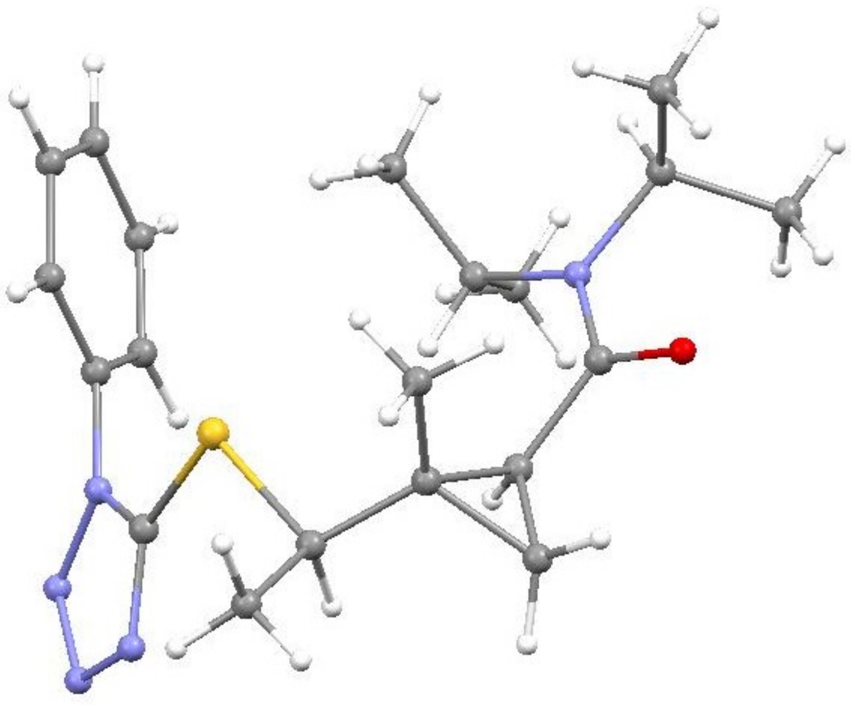

Fig. S21. Solid state structure of **3z**. Displacement ellipsoid plots are drawn at 50% probability. Hydrogen atoms are hidden for clarity.

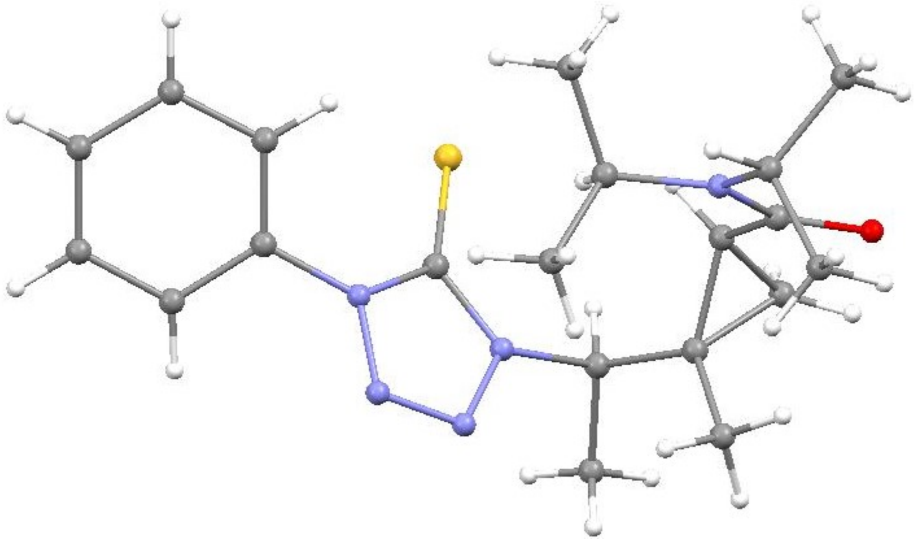

Fig. S22. Solid state structure of **3aa**. Displacement ellipsoid plots are drawn at 50% probability. Hydrogen atoms are hidden for clarity.

21. Copies of NMR spectra

tert-butyl(((1*S**,3*S**)-2,2-dibromo-1,3-dimethylcyclopropyl)methoxy)dimethylsilane, S1

^{1}H NMR (400 MHz, Chloroform-*d*)

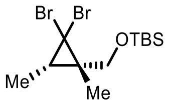

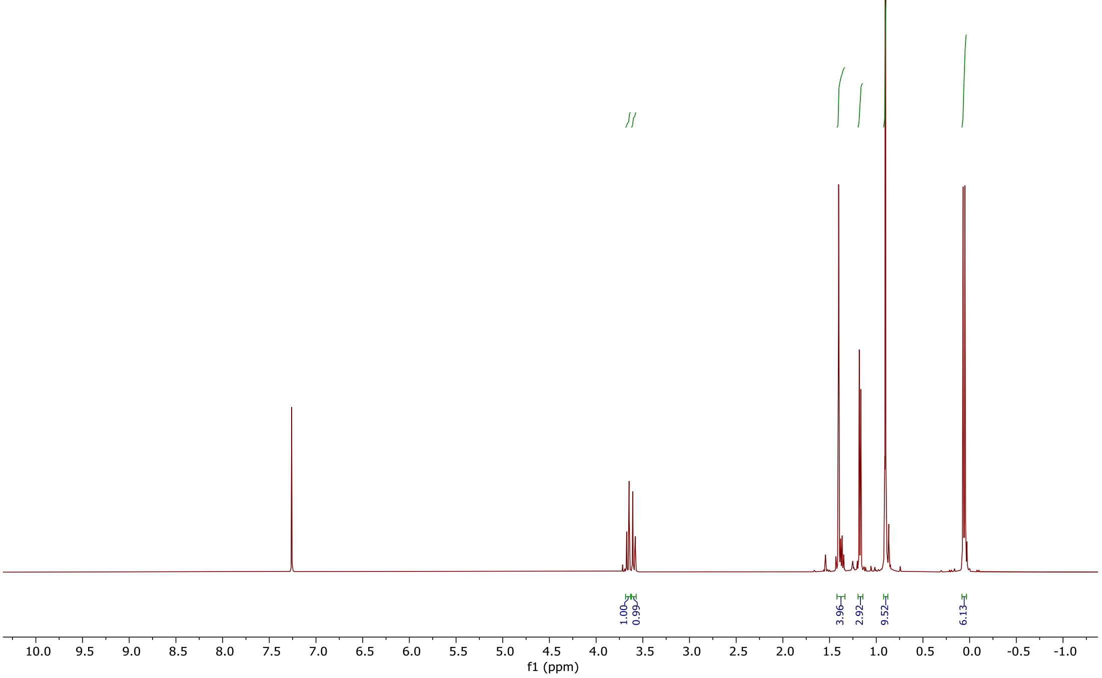

^{13}C NMR (101 MHz, Chloroform-*d*)

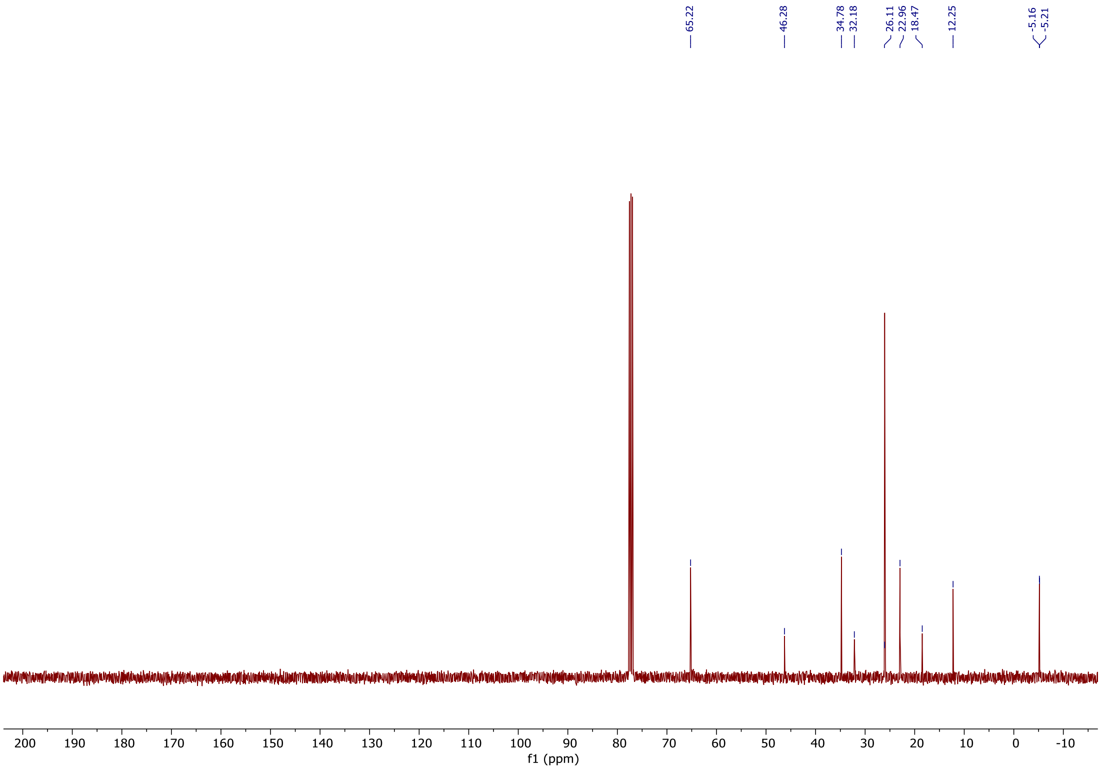

¹H COSY (400 MHz, Chloroform-*d*)

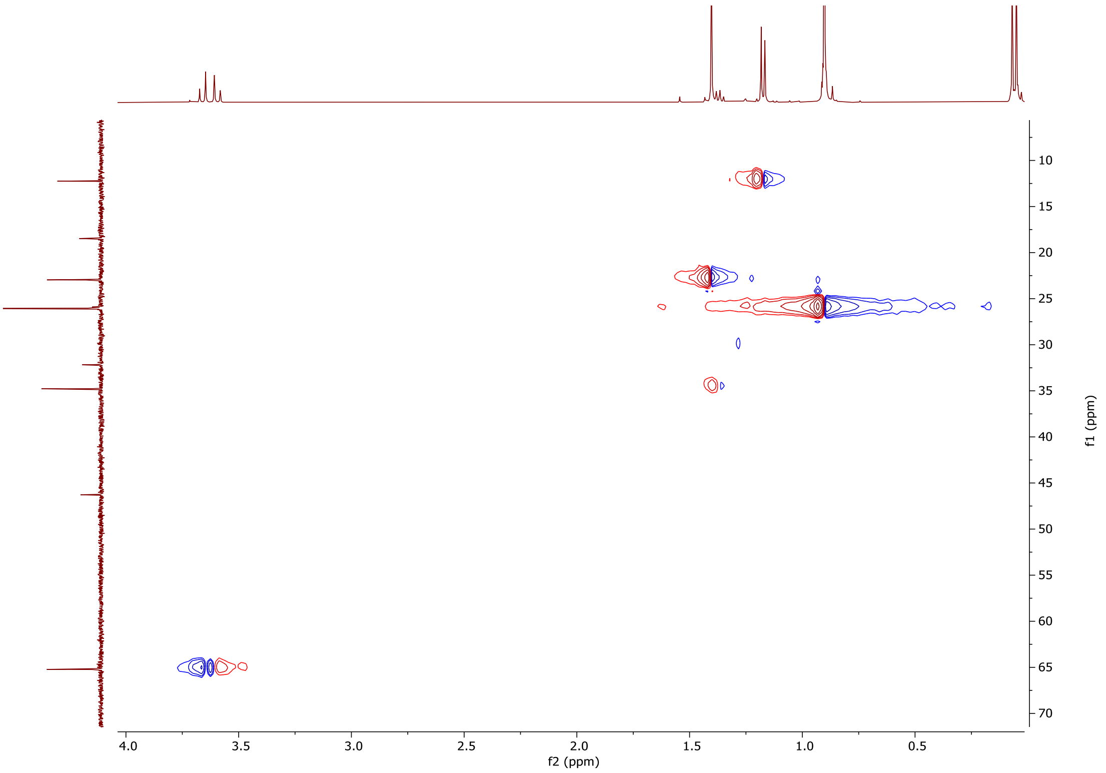

¹H/¹³C HSQC (400/101 MHz, Chloroform-*d*)

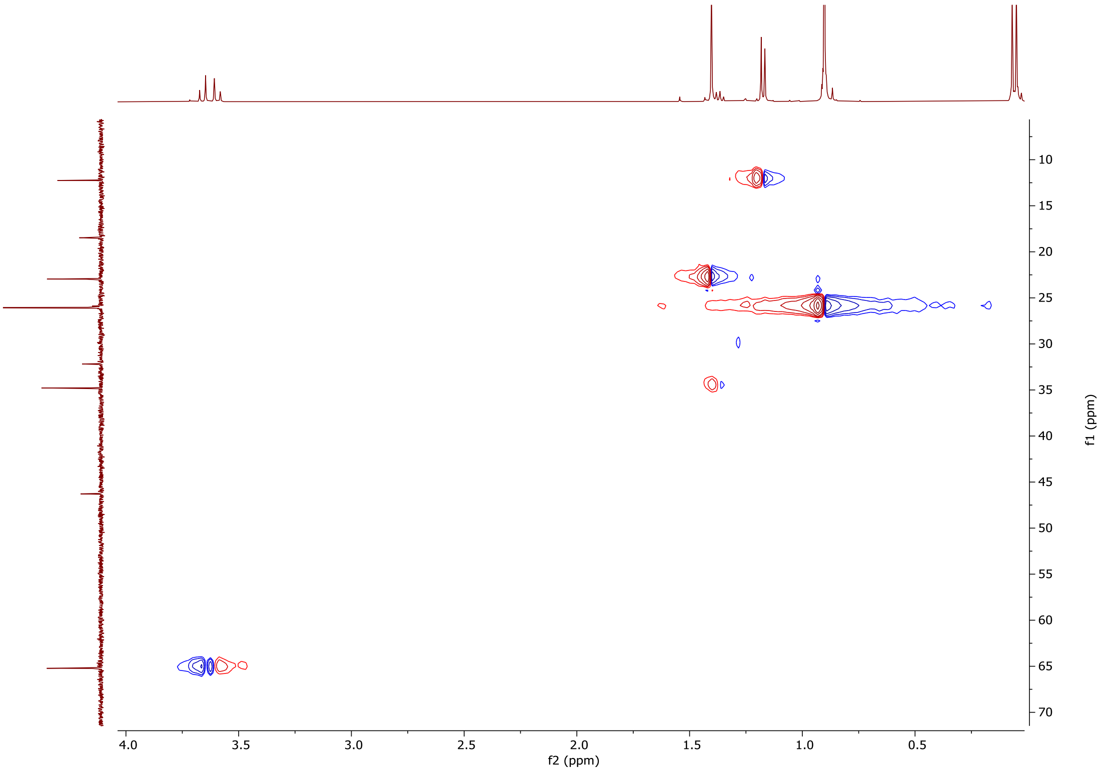

((1*S,3*S**)-2,2-dibromo-1,3-dimethylcyclopropyl)methanol, S2**

^{1}H NMR (400 MHz, Chloroform-*d*)

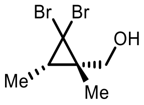

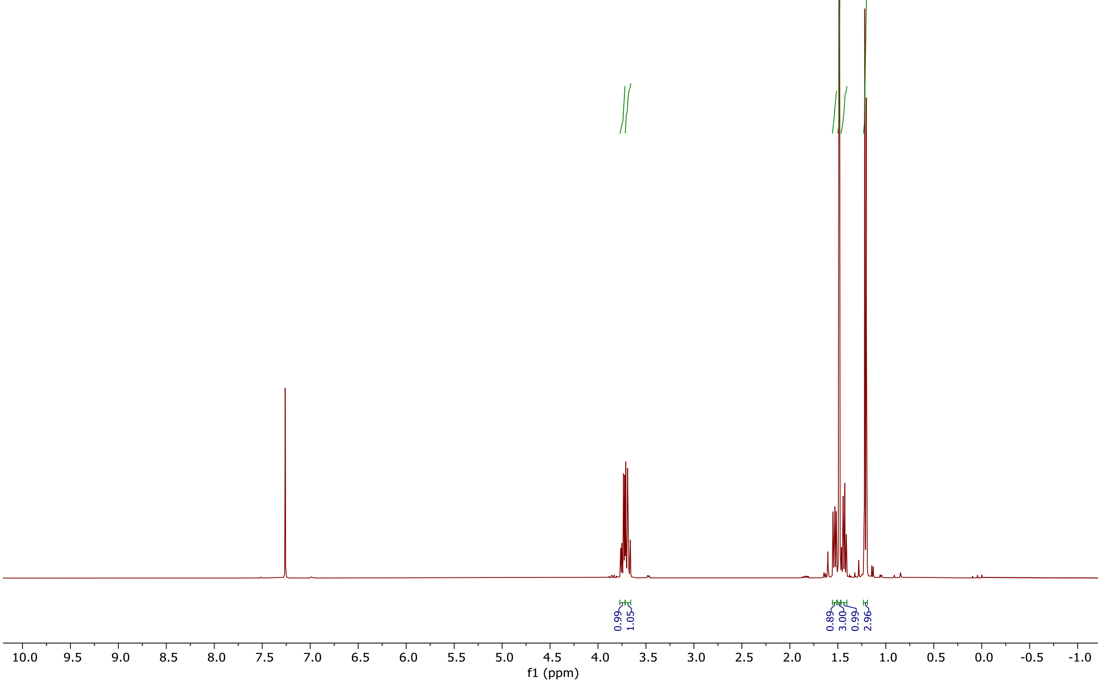

^{13}C NMR (101 MHz, Chloroform-*d*)

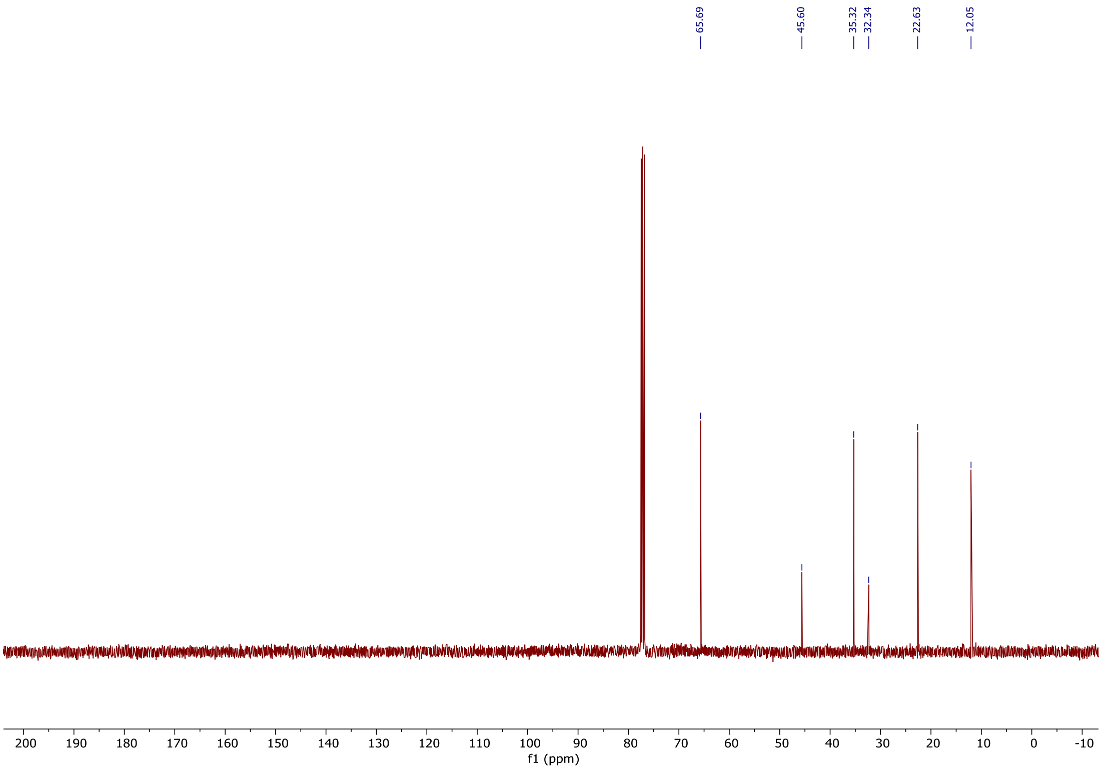

^{1}**H COSY** (400 MHz, Chloroform-*d*)

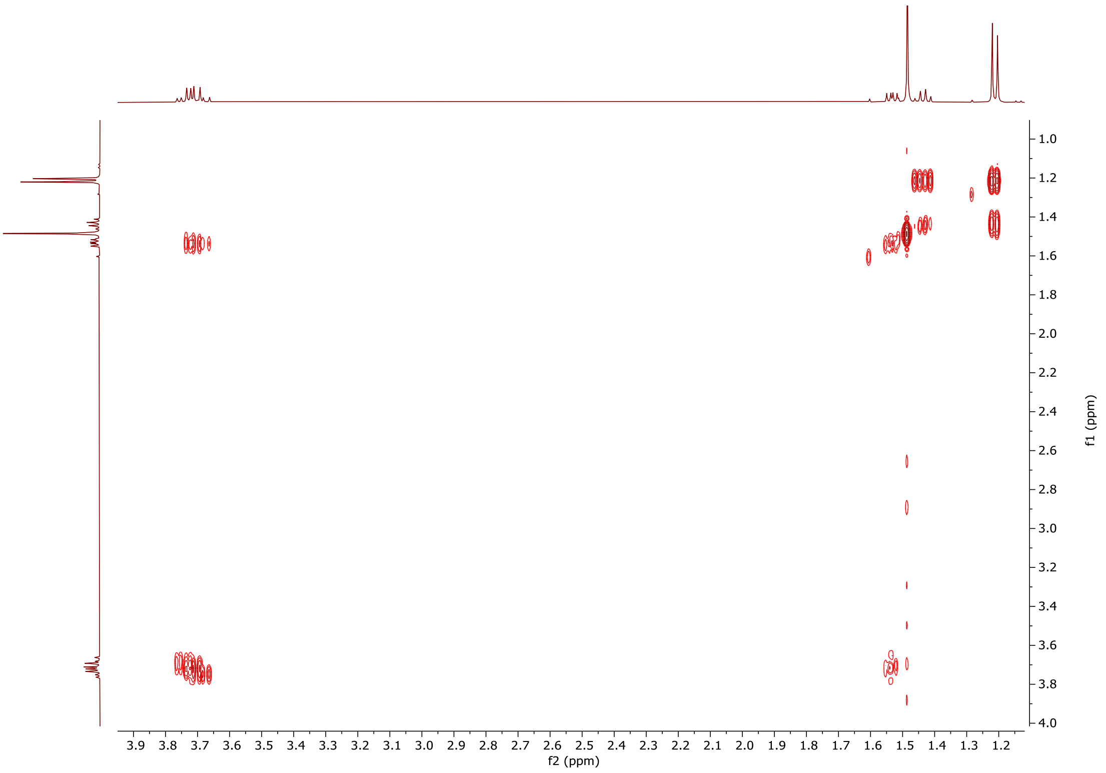

^{1}**H/**^{13}**C HSQC** (400/101 MHz, Chloroform-*d*)

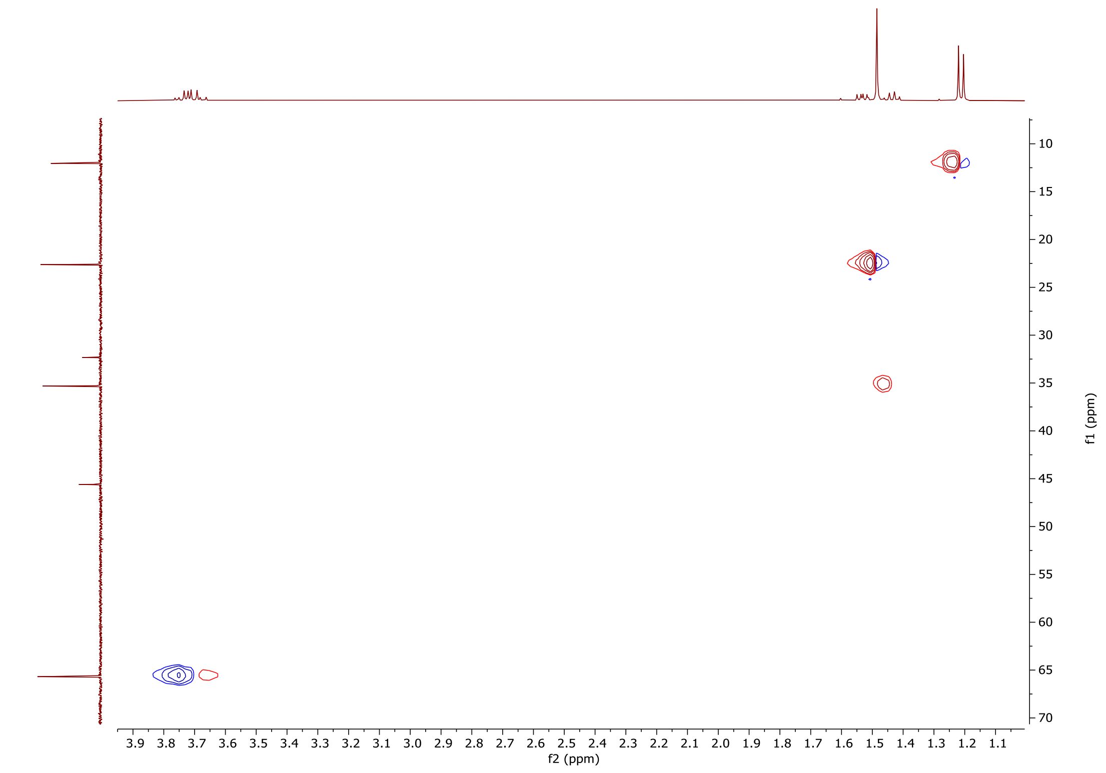

(2*S,3*S**)-1,1-dibromo-2-(bromomethyl)-2,3-dimethylcyclopropane, S3**

^{1}**H NMR** (400 MHz, Chloroform-*d*)

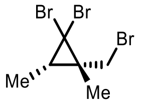

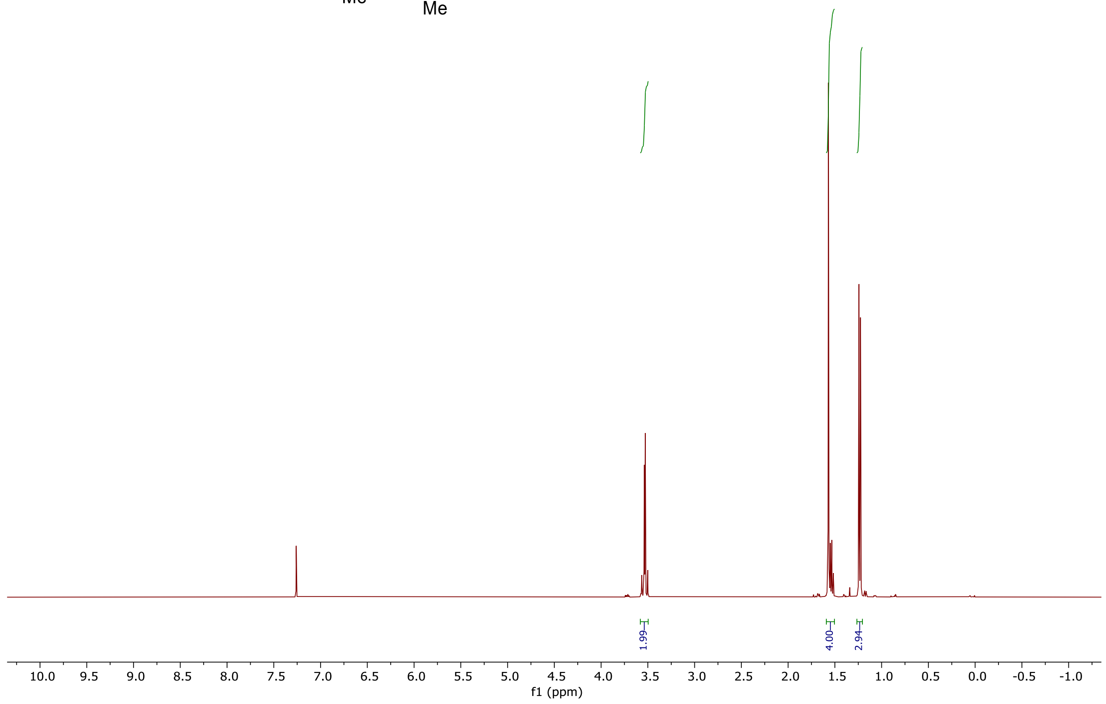

^{13}**C NMR** (101 MHz, Chloroform-*d*)

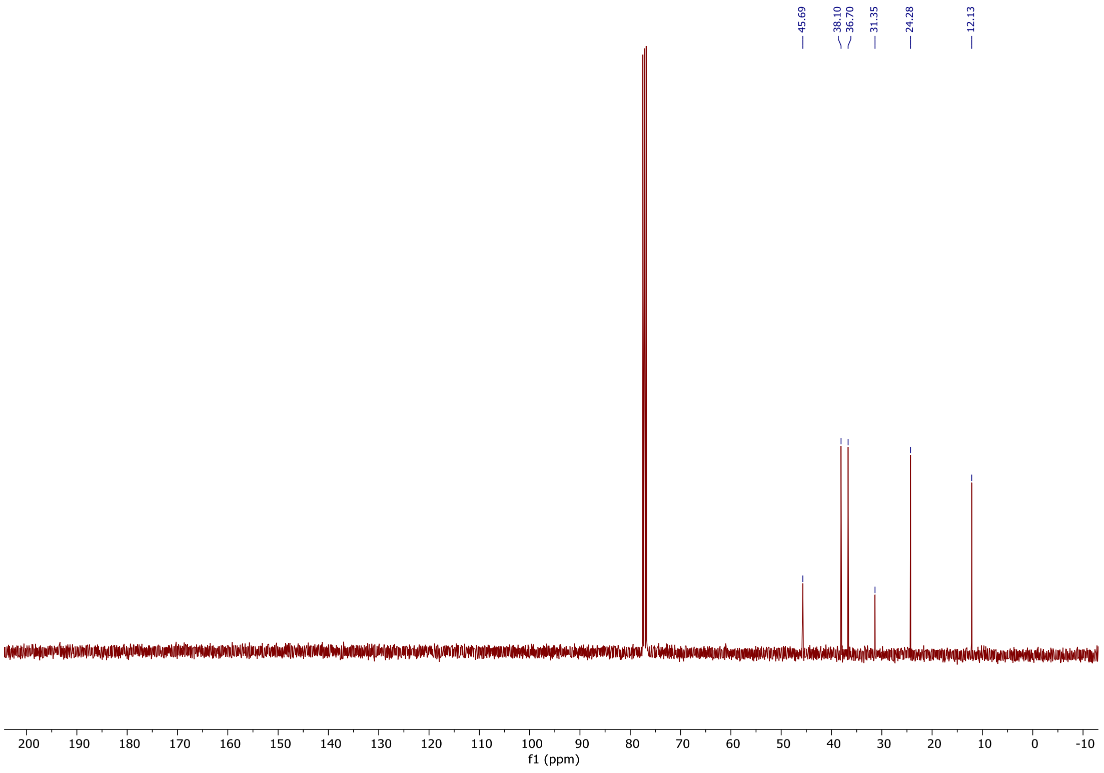

^{1}**H COSY** (400 MHz, Chloroform-*d*)

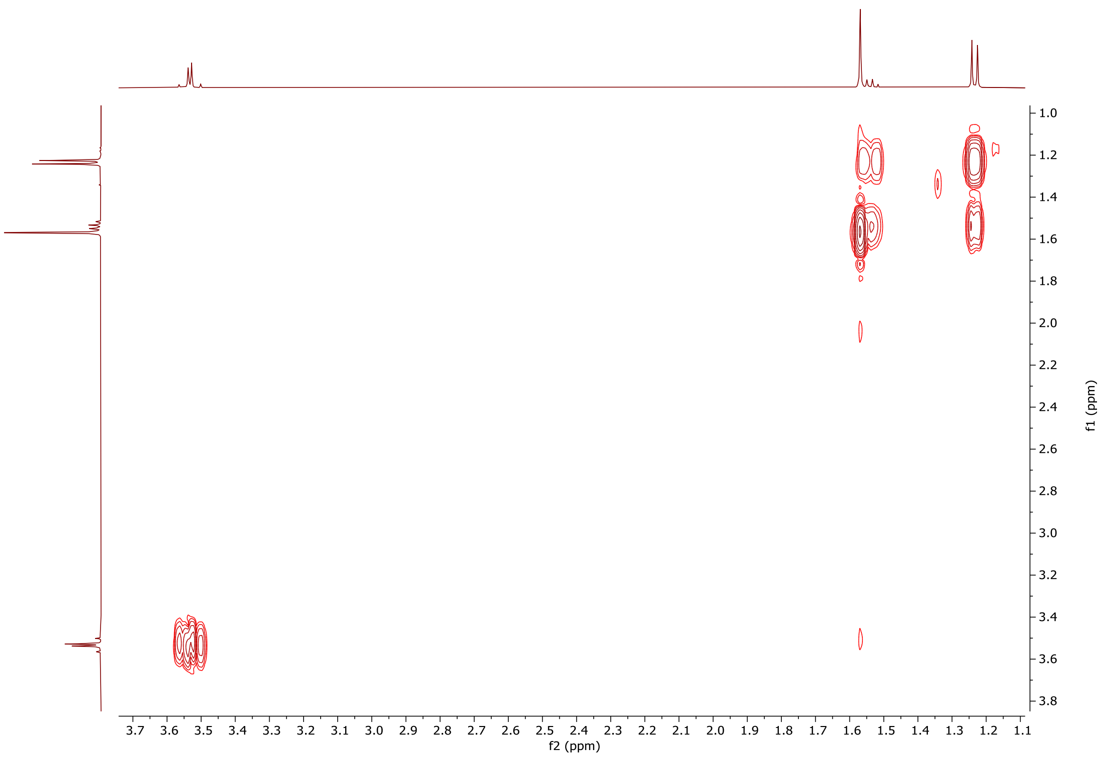

^{1}**H/**^{13}**C HSQC** (400/101 MHz, Chloroform-*d*)

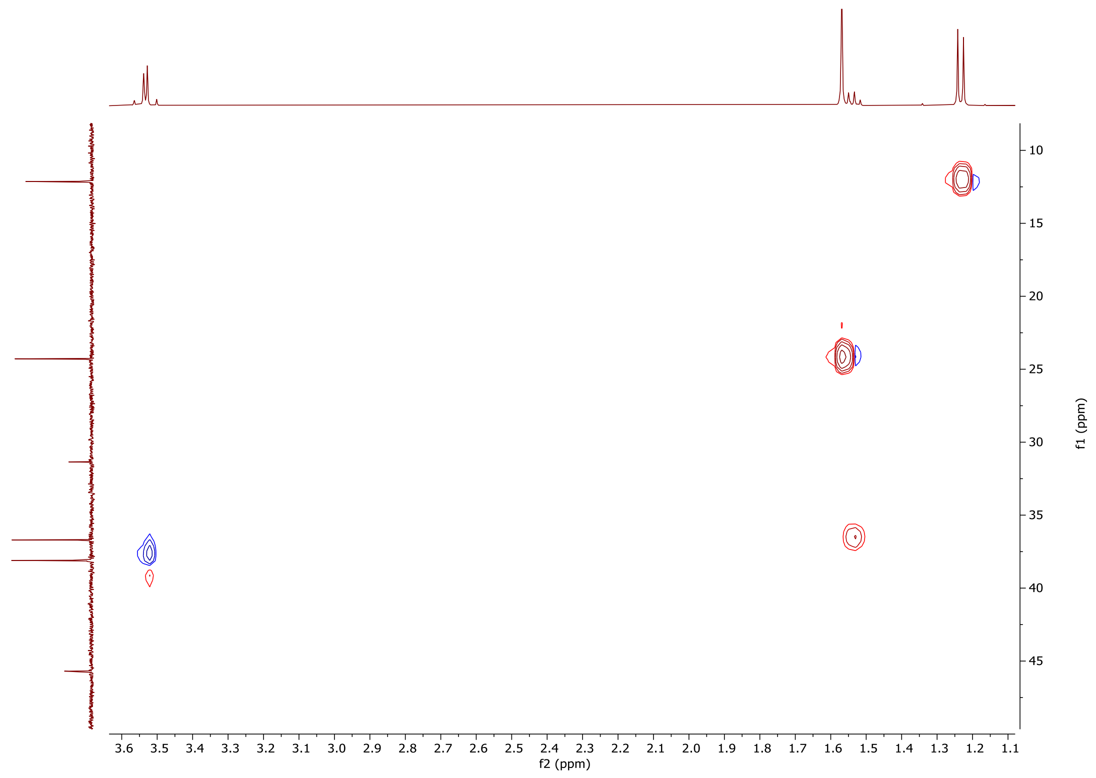

Methyl (1*R,2*S**,4*S**)-4-(diisopropylcarbamoyl)-2-methyl-2-(2,2,2-trifluoroacetoxy)cyclobutane-1-carboxylate, S6**

^{1}H NMR (600 MHz, Chloroform-*d*)

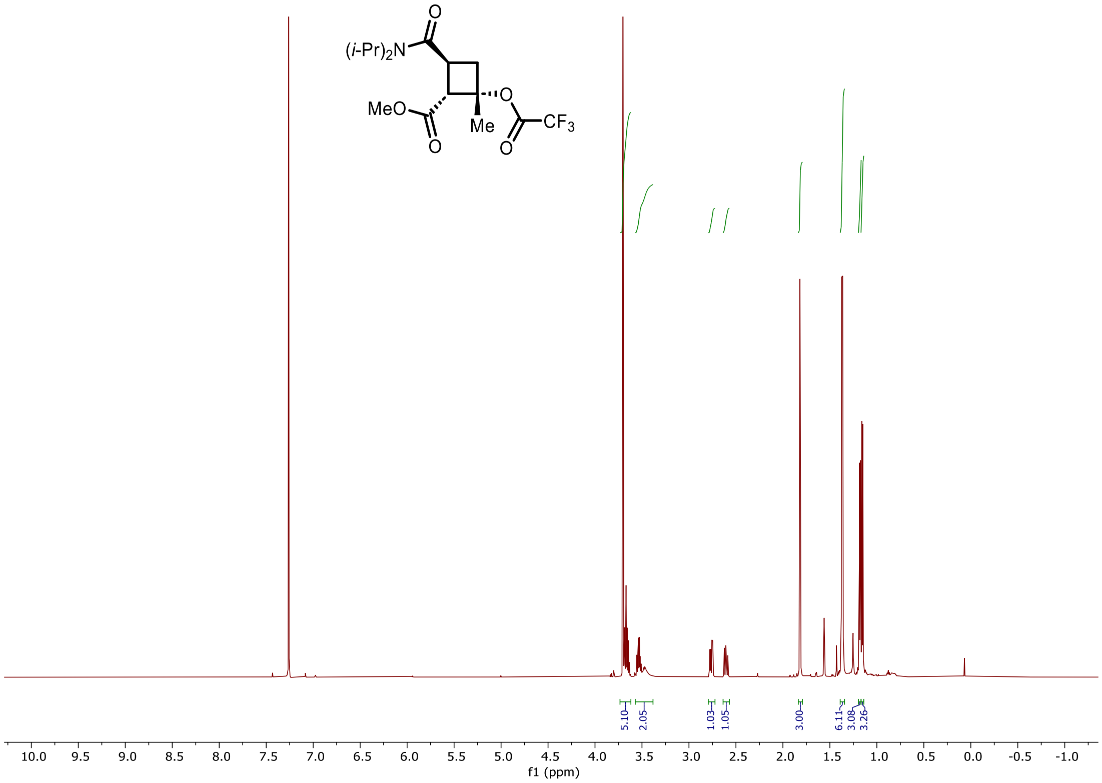

^{13}C NMR (151 MHz, Chloroform-*d*)

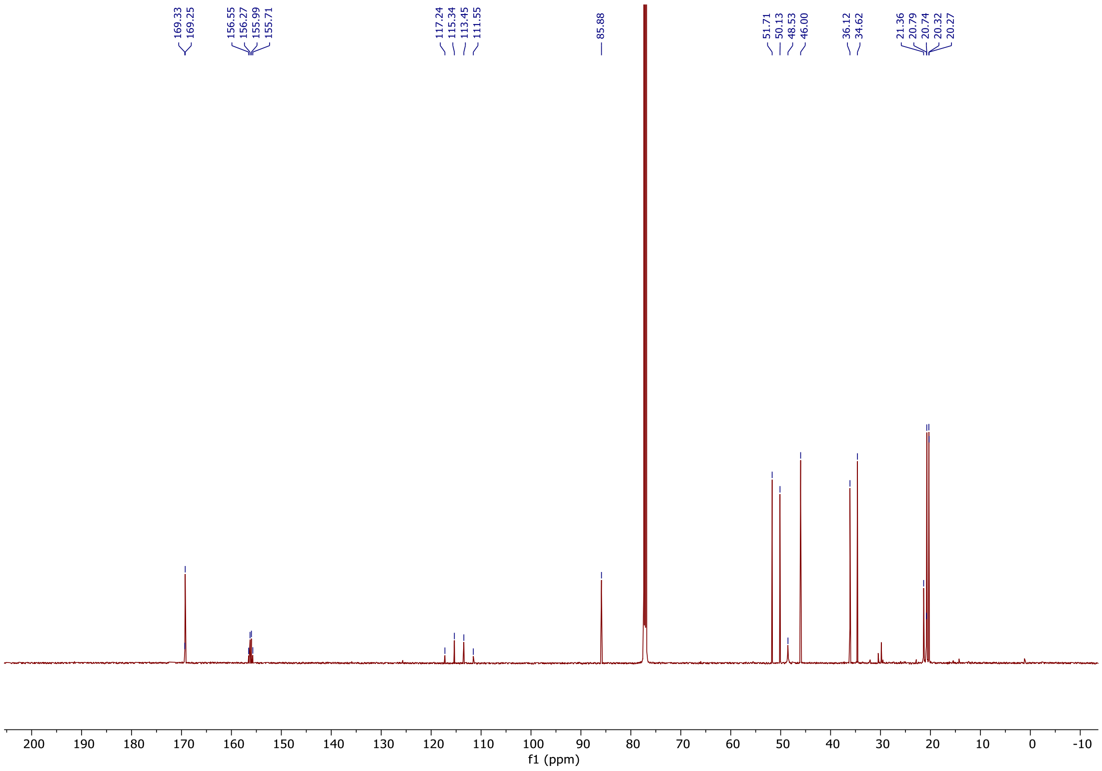

^{1}H COSY (600 MHz, Chloroform-*d*)

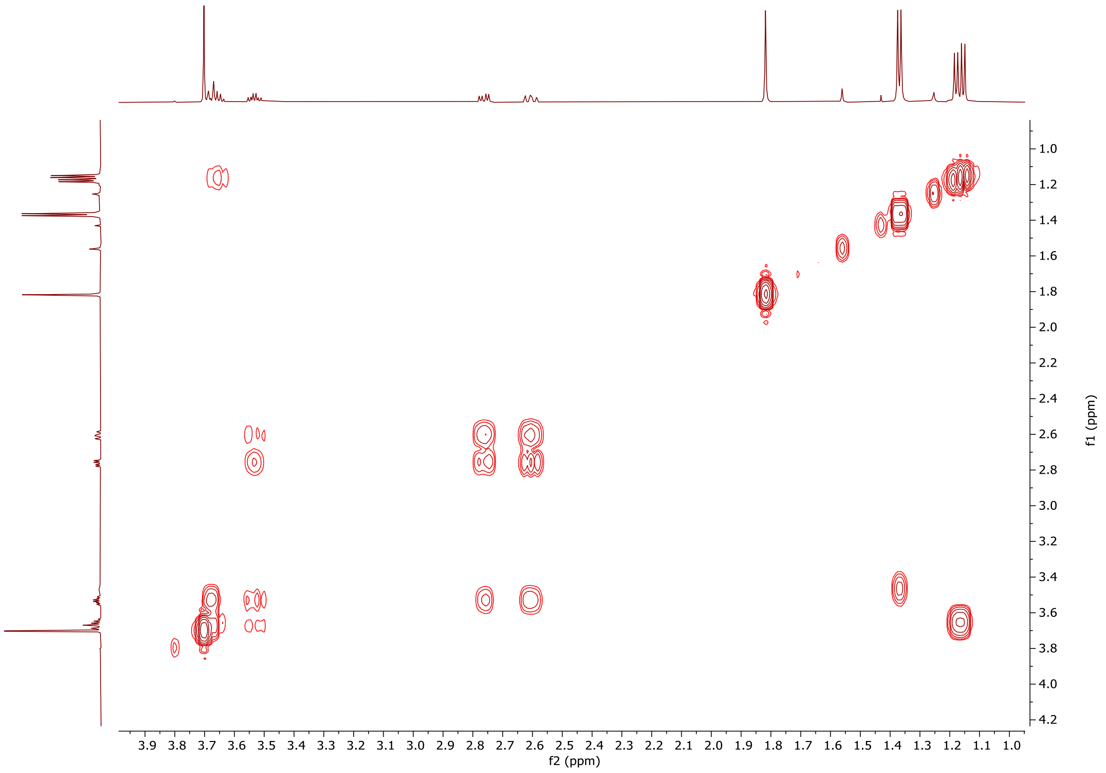

^{1}H/^{13}C HSQC (600/151 MHz, Chloroform-*d*)

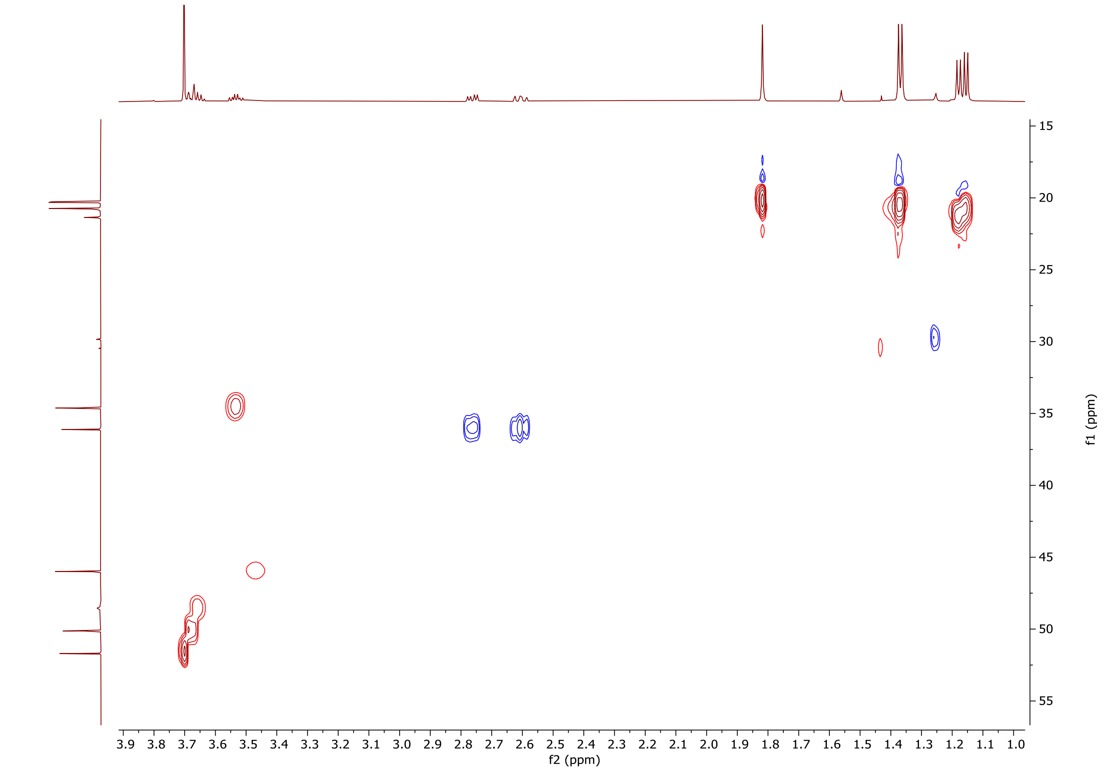

$^{1}H/^{13}C$ **HMBC** (600/151 MHz, Chloroform-*d*)

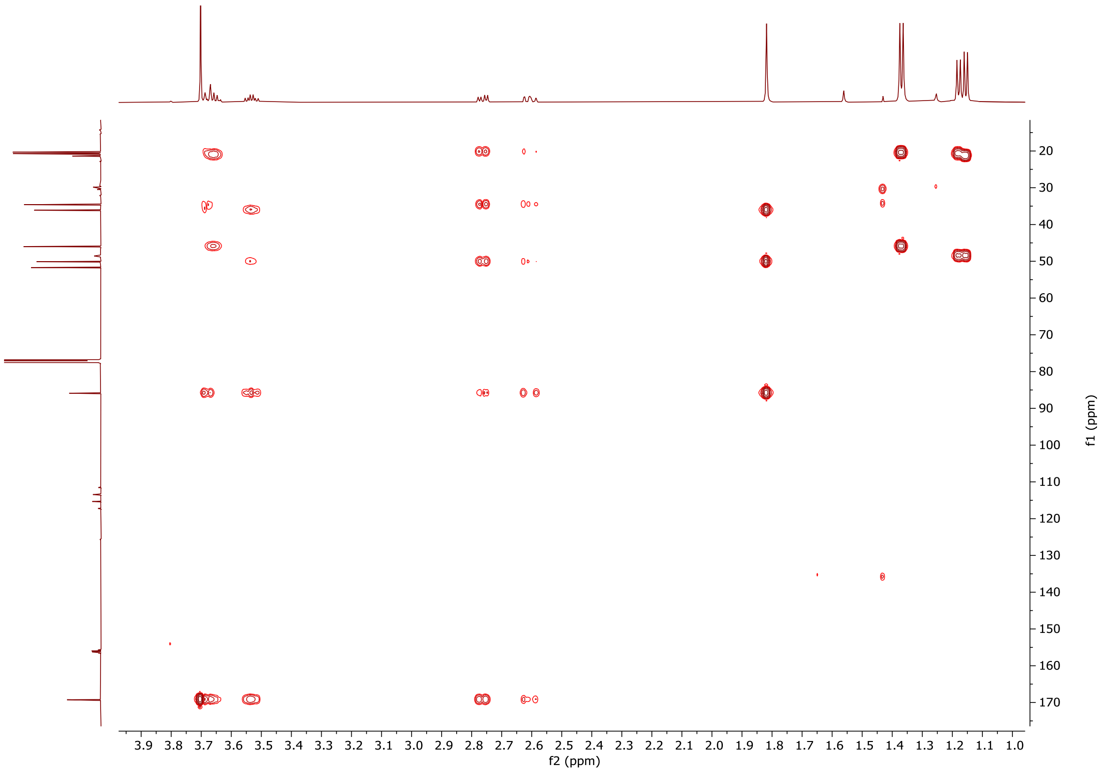

^{1}H **NOSEY** (600 MHz, Chloroform-*d*)

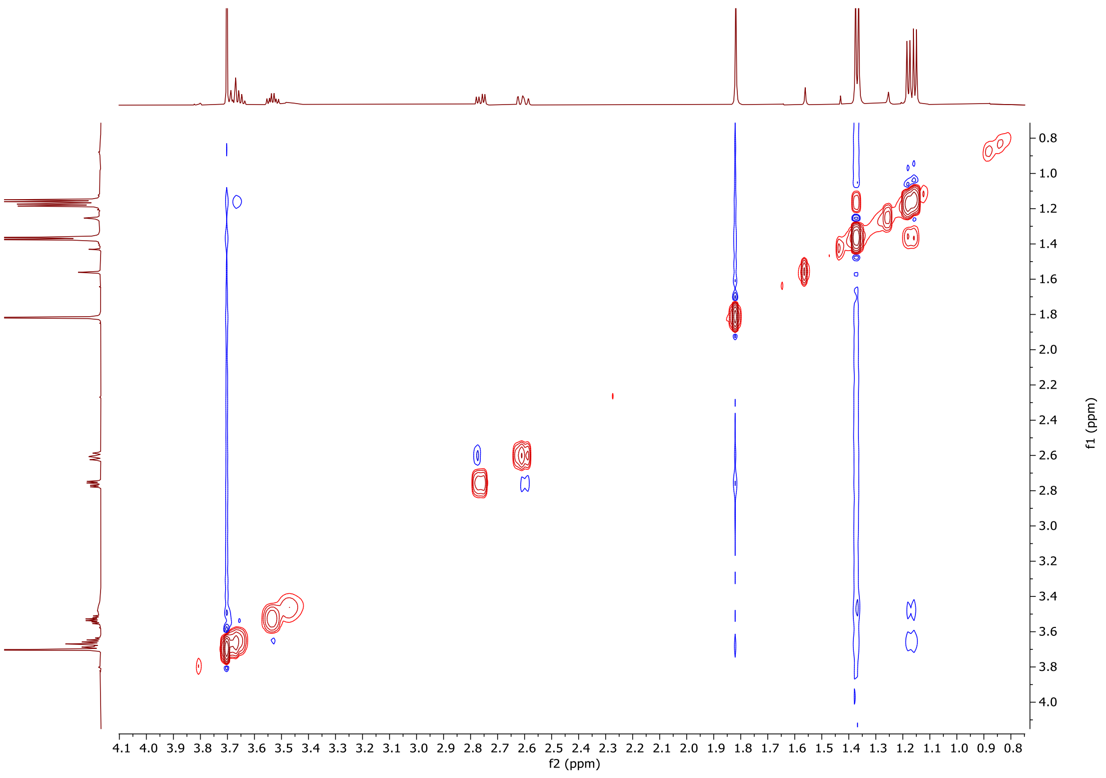

^{19}F NMR (565 MHz, Chloroform-*d*)

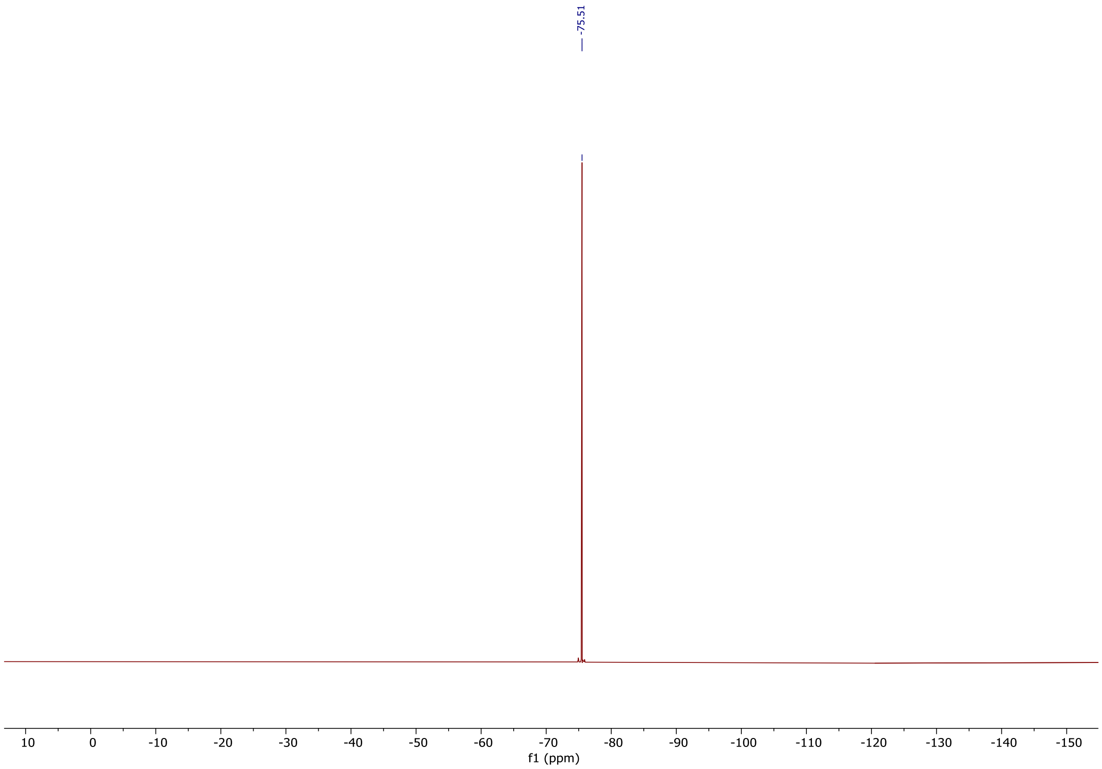

(1*S,3*S**)-*N*,*N*-diisopropyl-2,3-dimethylbicyclo[1.1.0]butane-1-carboxamide, 1o**

^{1}H NMR (600 MHz, Chloroform-*d*)

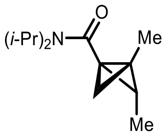

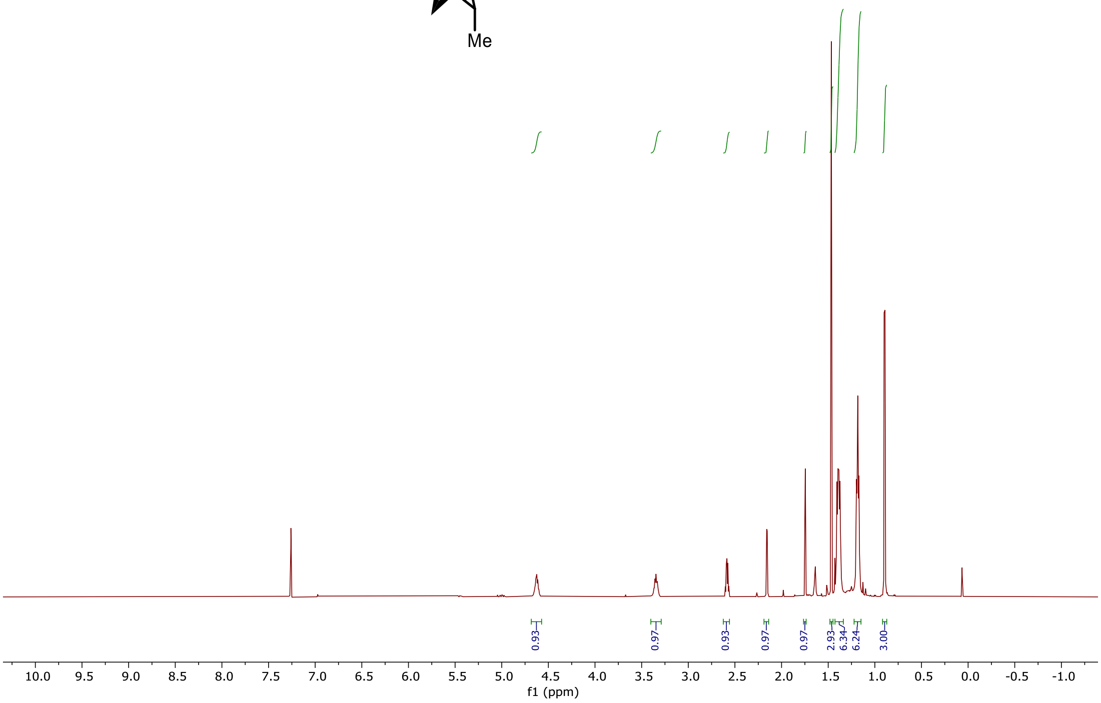

^{13}C NMR (151 MHz, Chloroform-*d*)

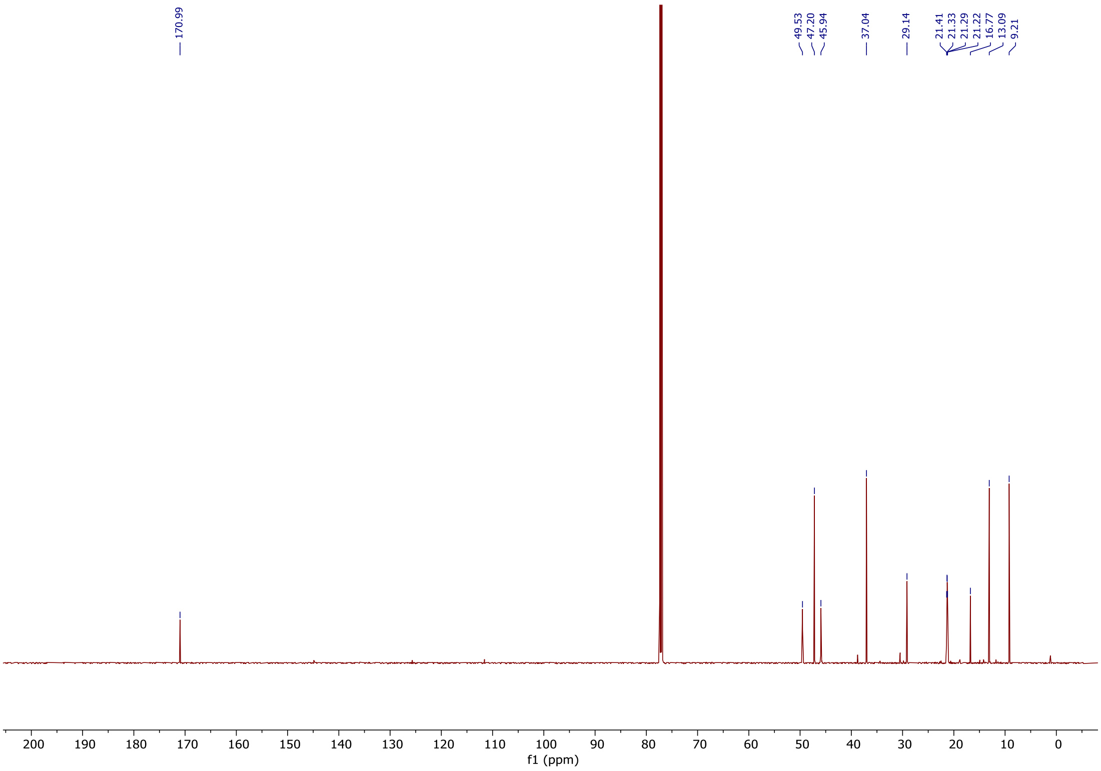

^{1}H COSY (600 MHz, Chloroform-*d*)

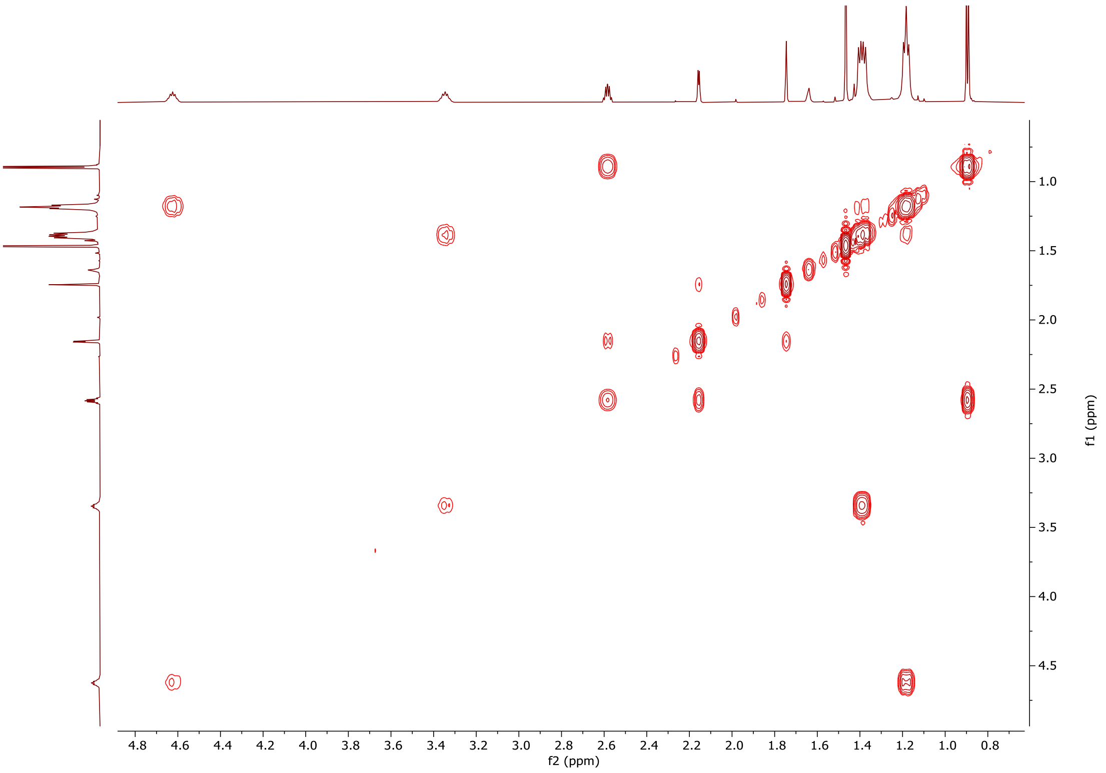

^{1}H/^{13}C HSQC (600/151 MHz, Chloroform-*d*)

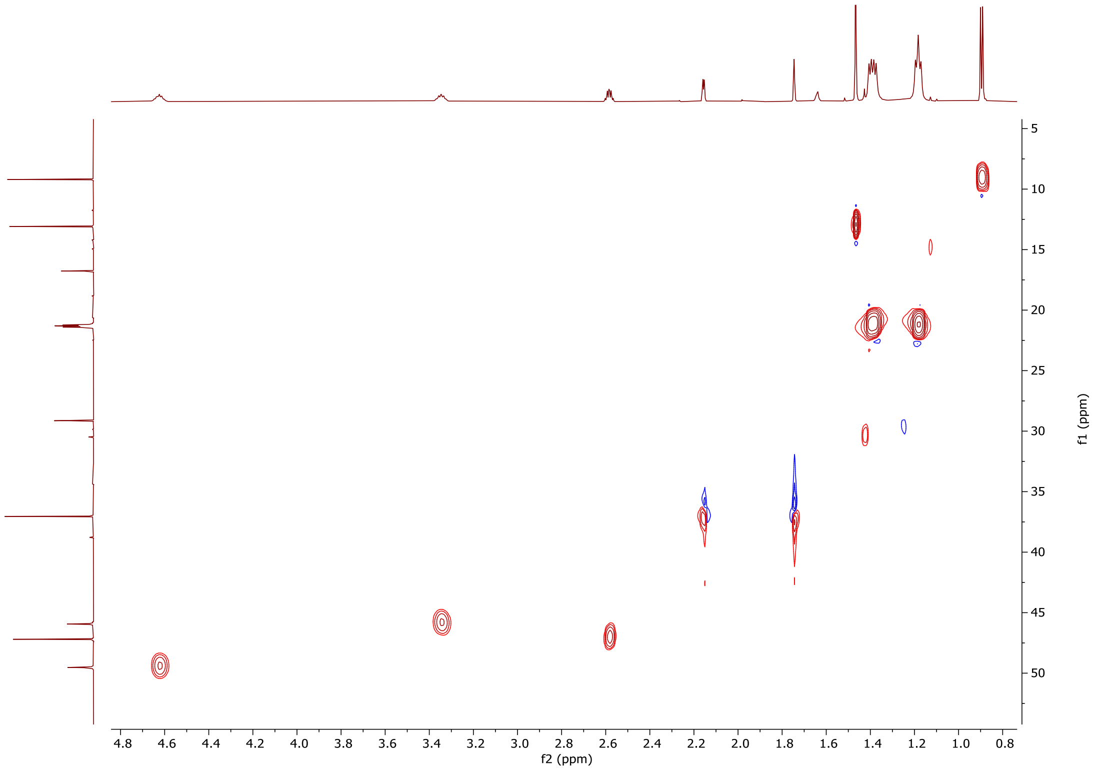

$^{1}H/^{13}C$ HMBC (600/151 MHz, Chloroform-*d*)

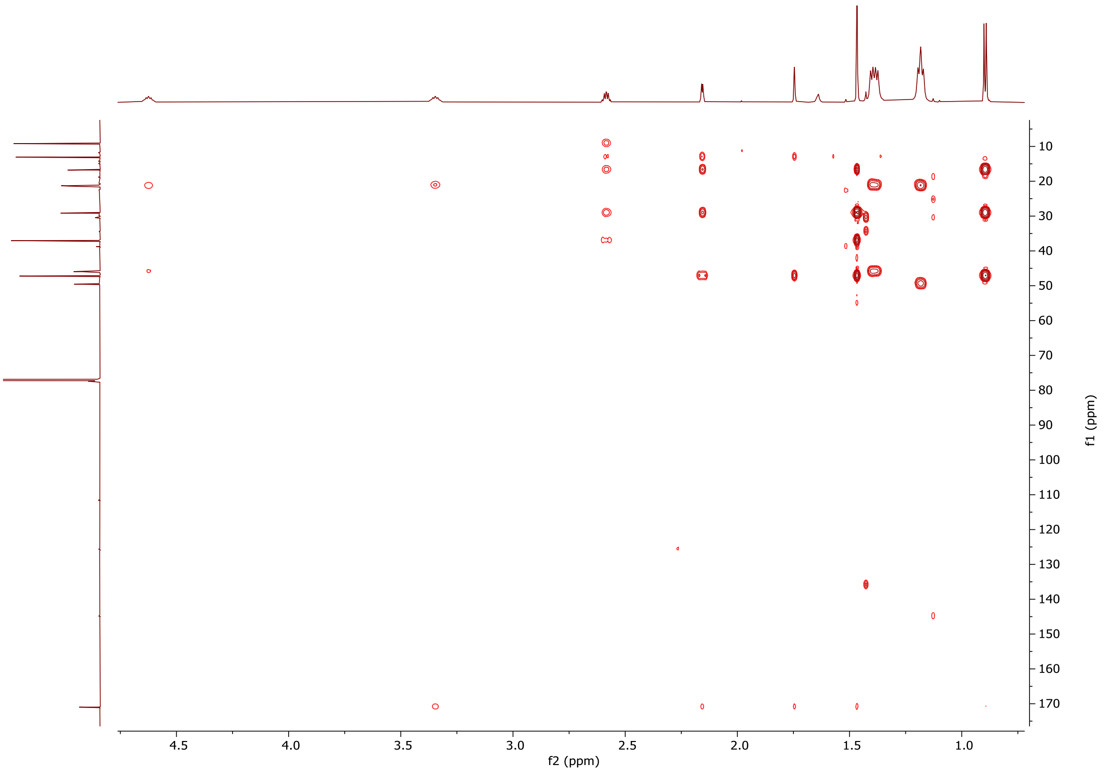

^{1}H NOSEY (600 MHz, Chloroform-*d*)

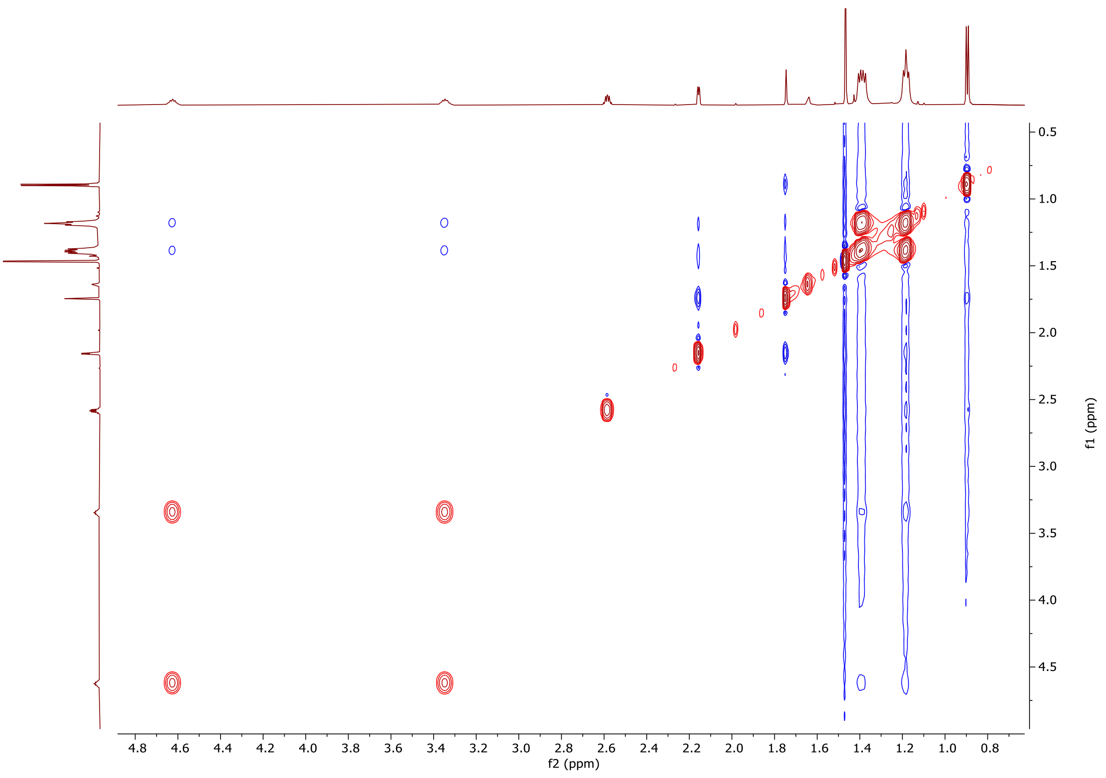

***N*,*N*-diisopropyl-2,3-diphenylcyclobut-2-ene-1-carboxamide, 2b**

^{1}H NMR (600 MHz, Chloroform-*d*)

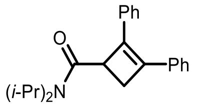

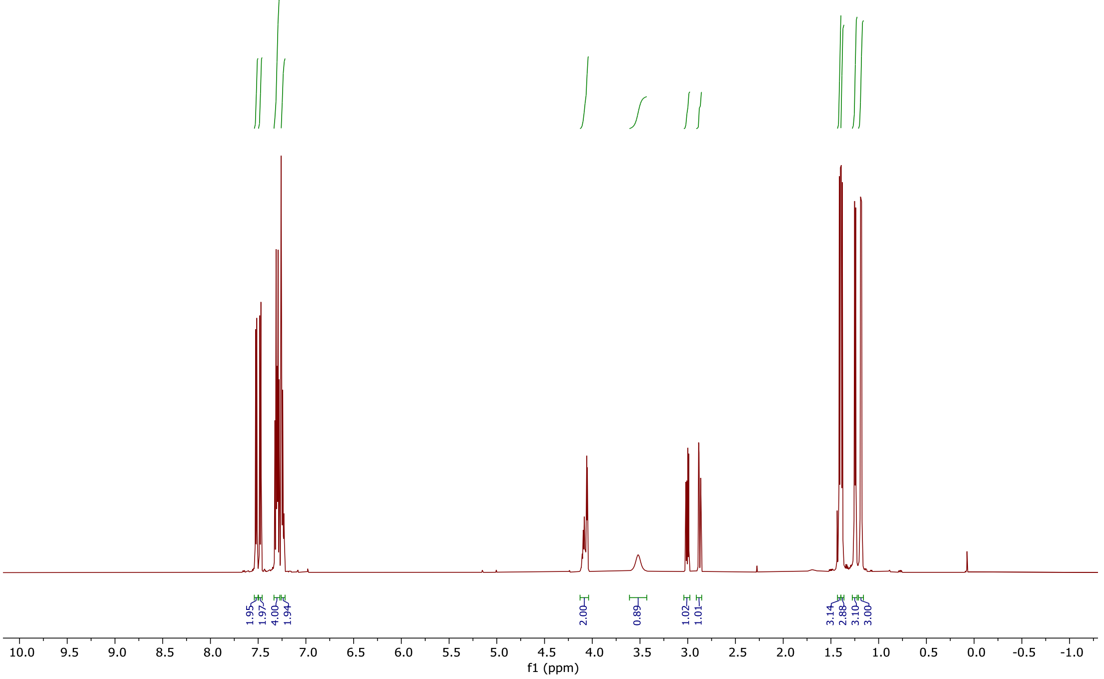

^{13}C NMR (151 MHz, Chloroform-*d*)

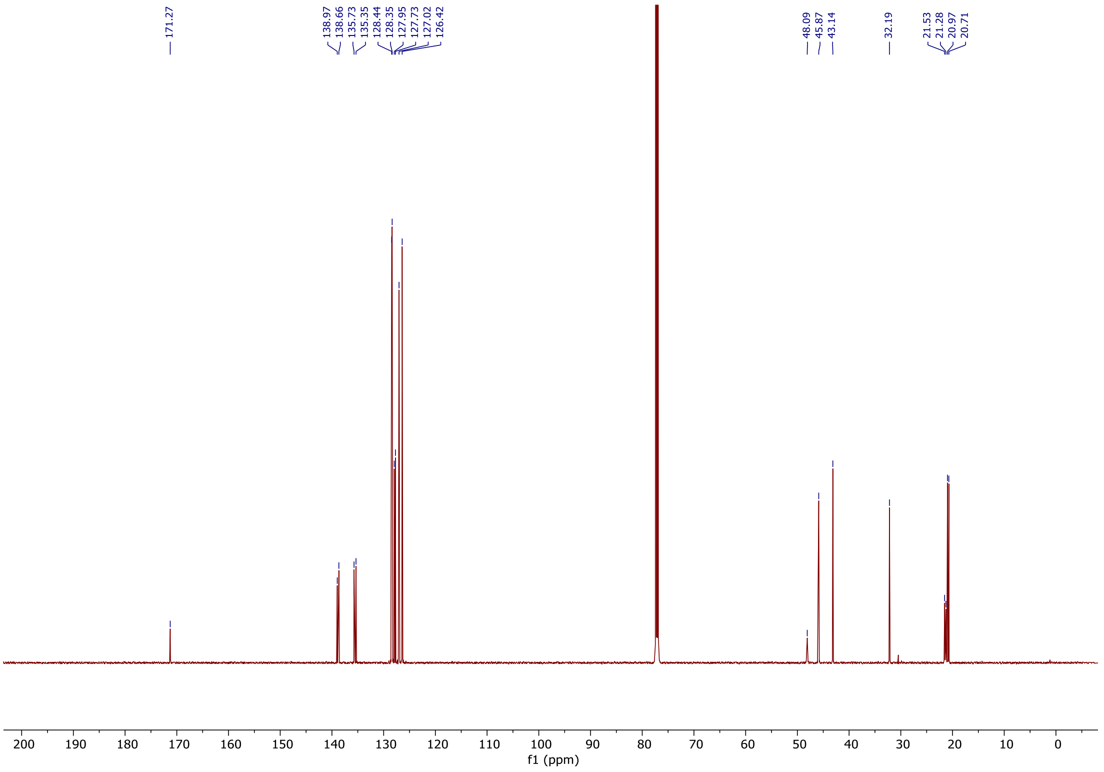

^{1}**H COSY** (600 MHz, Chloroform-*d*)

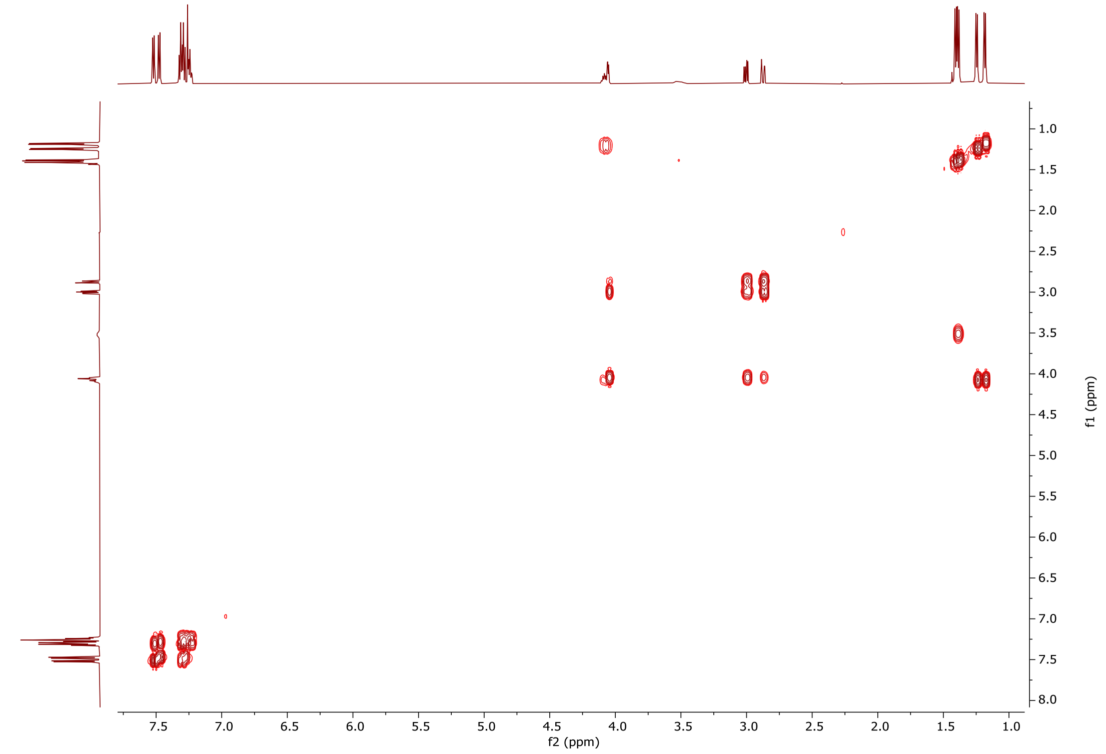

^{1}**H/**^{13}**C HSQC** (600/151 MHz, Chloroform-*d*)

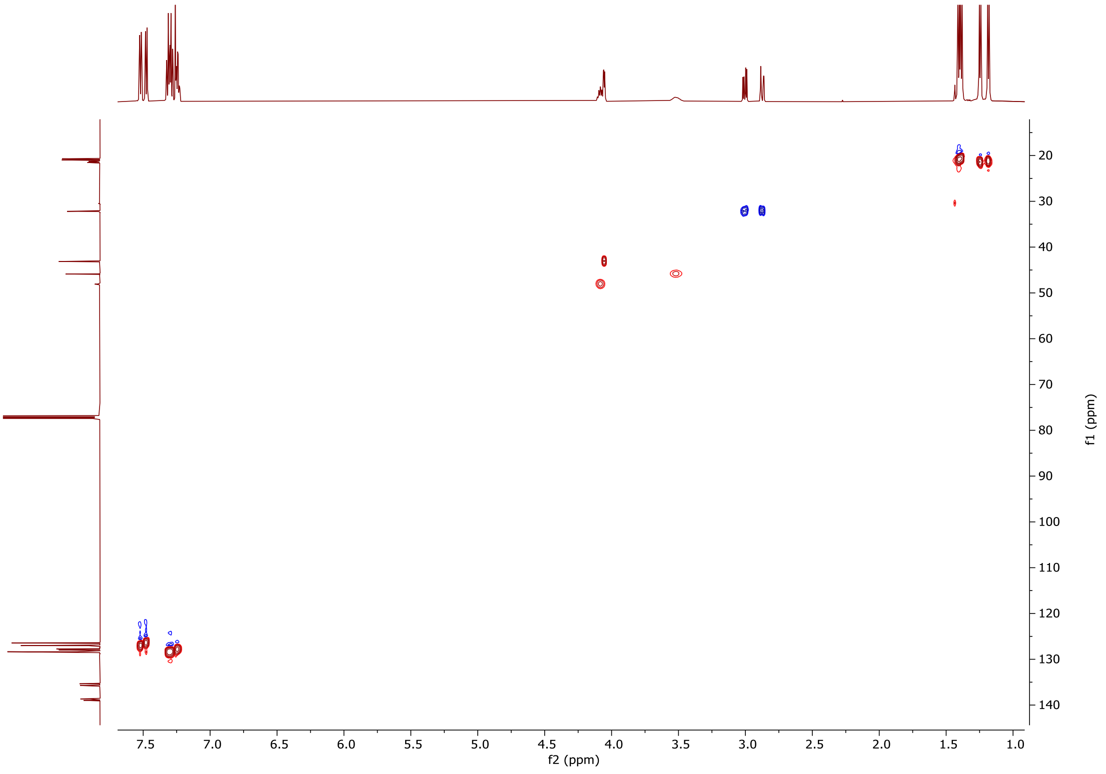

$^{1}H/^{13}C$ HMBC (600/151 MHz, Chloroform-*d*)

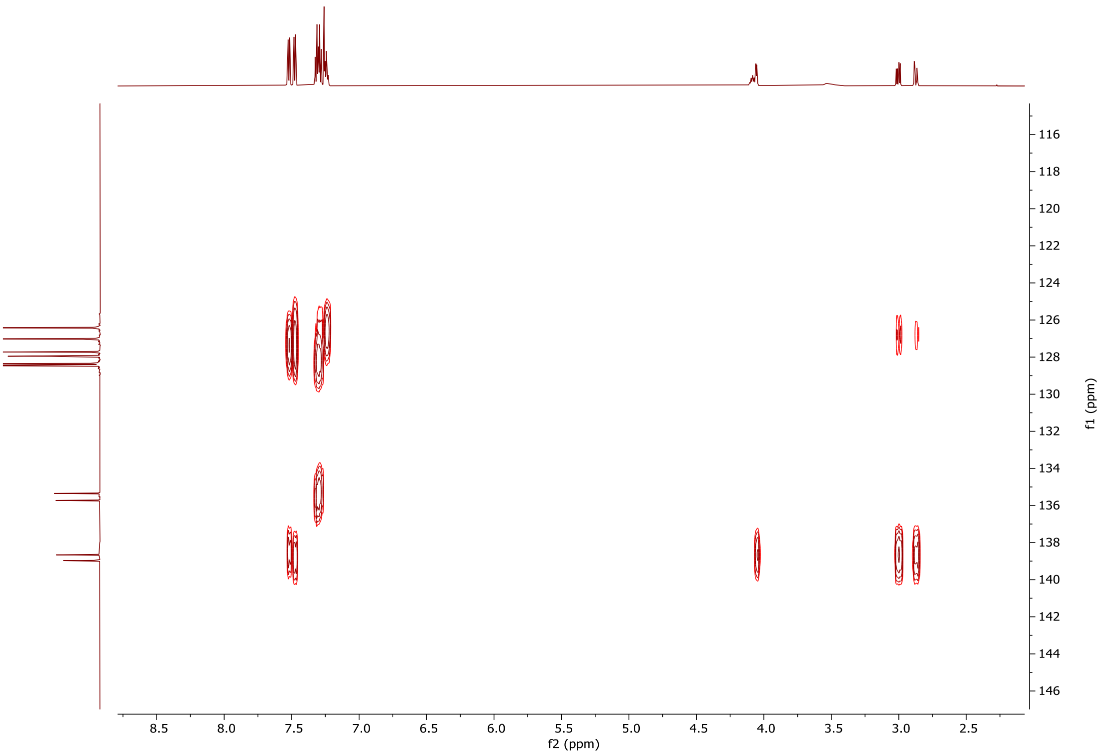

Methyl 4-(diisopropylcarbamoyl)-2-methylcyclobut-1-ene-1-carboxylate, 2c

^{1}H NMR (600 MHz, Chloroform-*d*)

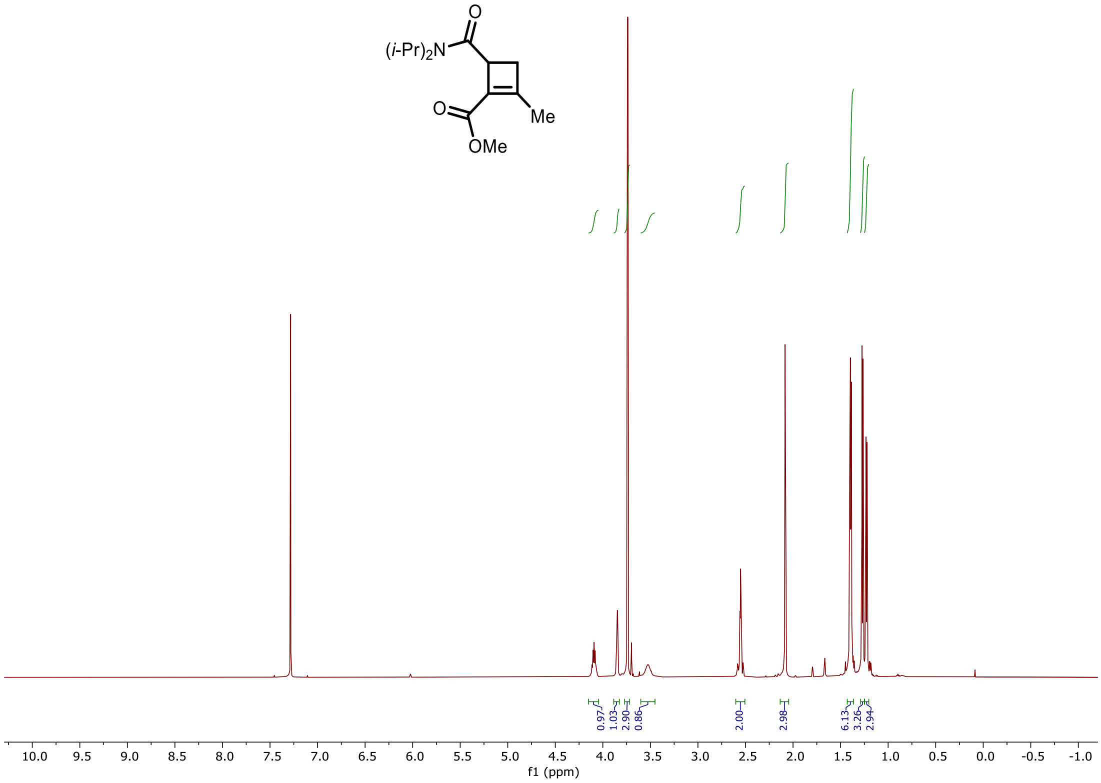

^{13}C NMR (151 MHz, Chloroform-*d*)

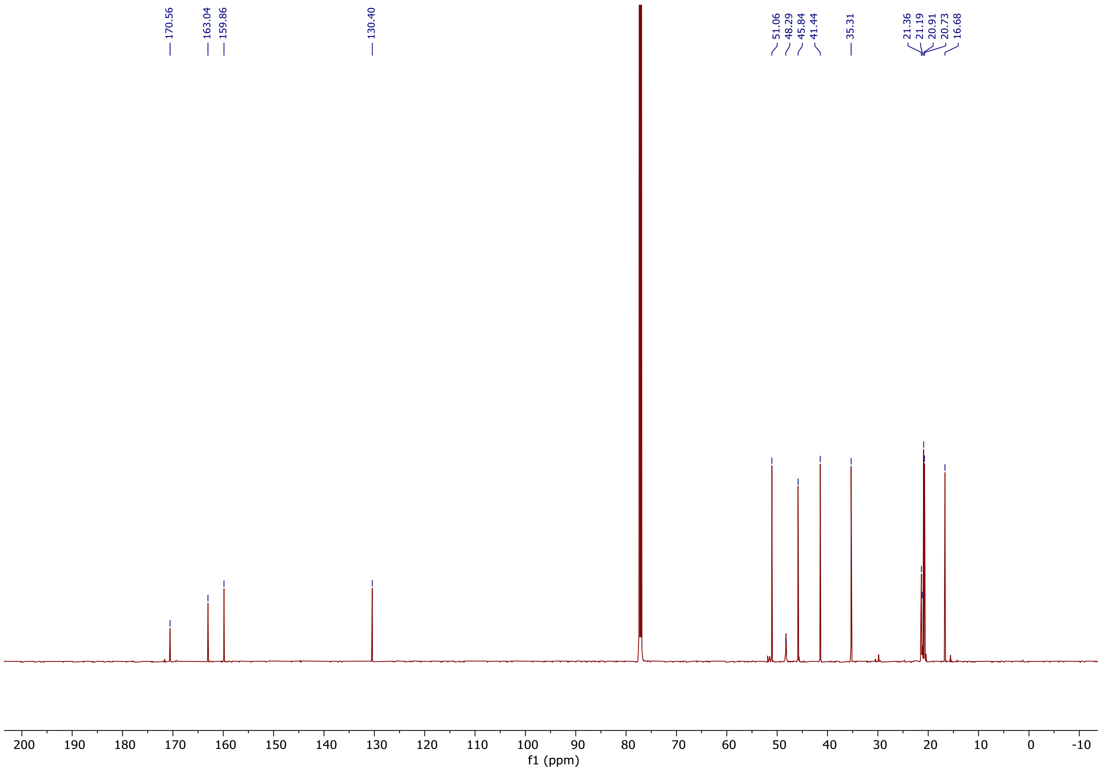

^{1}H COSY (600 MHz, Chloroform-*d*)

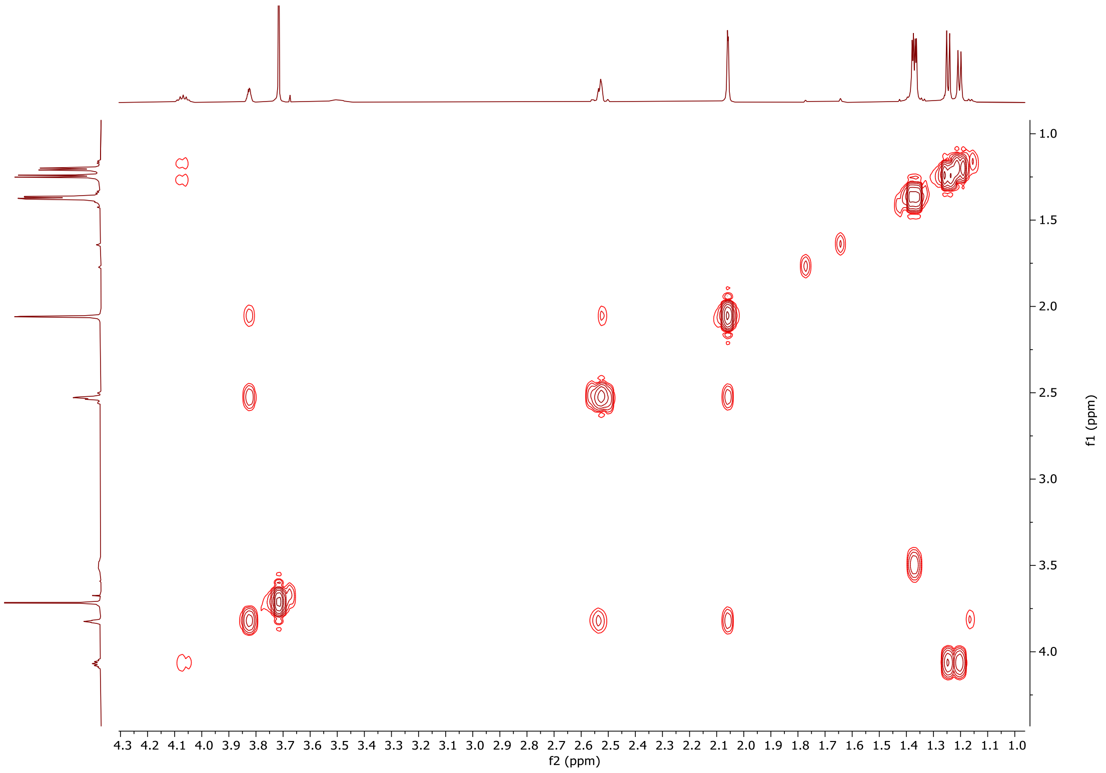

$^{1}H/^{13}C$ HSQC (600/151 MHz, Chloroform-*d*)

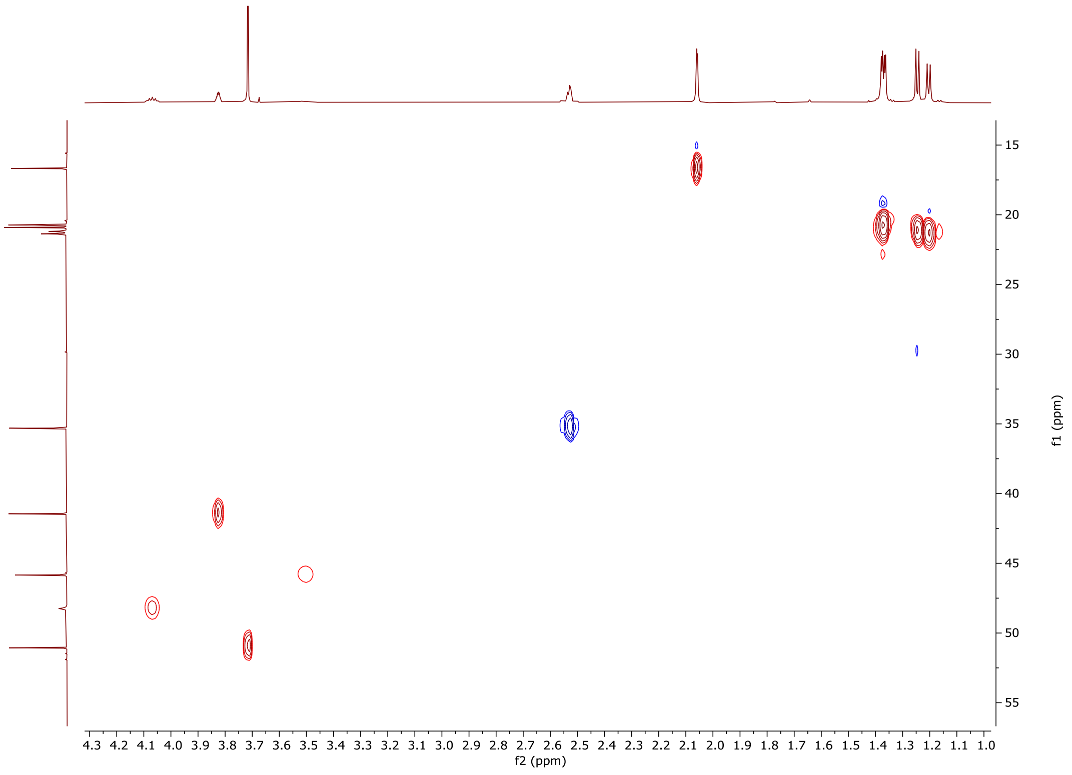

$^{1}H/^{13}C$ HMBC (600/151 MHz, Chloroform-*d*)

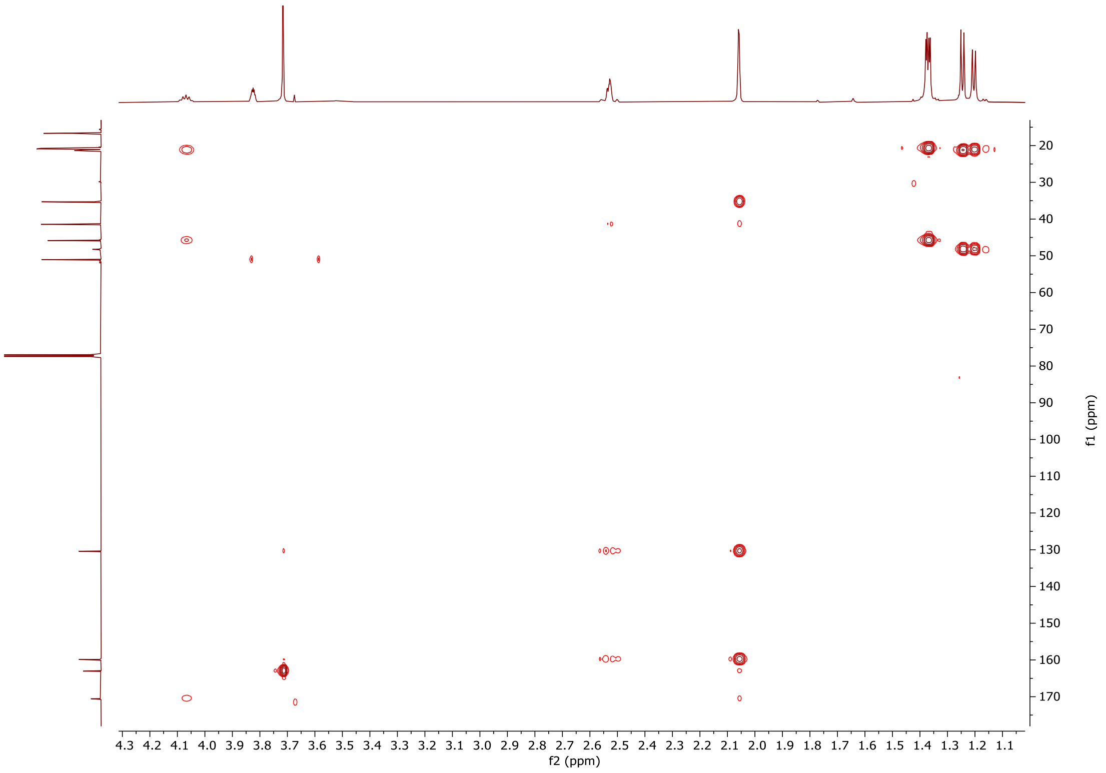

***N*,*N*-diisopropyl-3-methylcyclobut-2-ene-1-carboxamide, 2d**

^{1}H NMR (600 MHz, Chloroform-*d*)

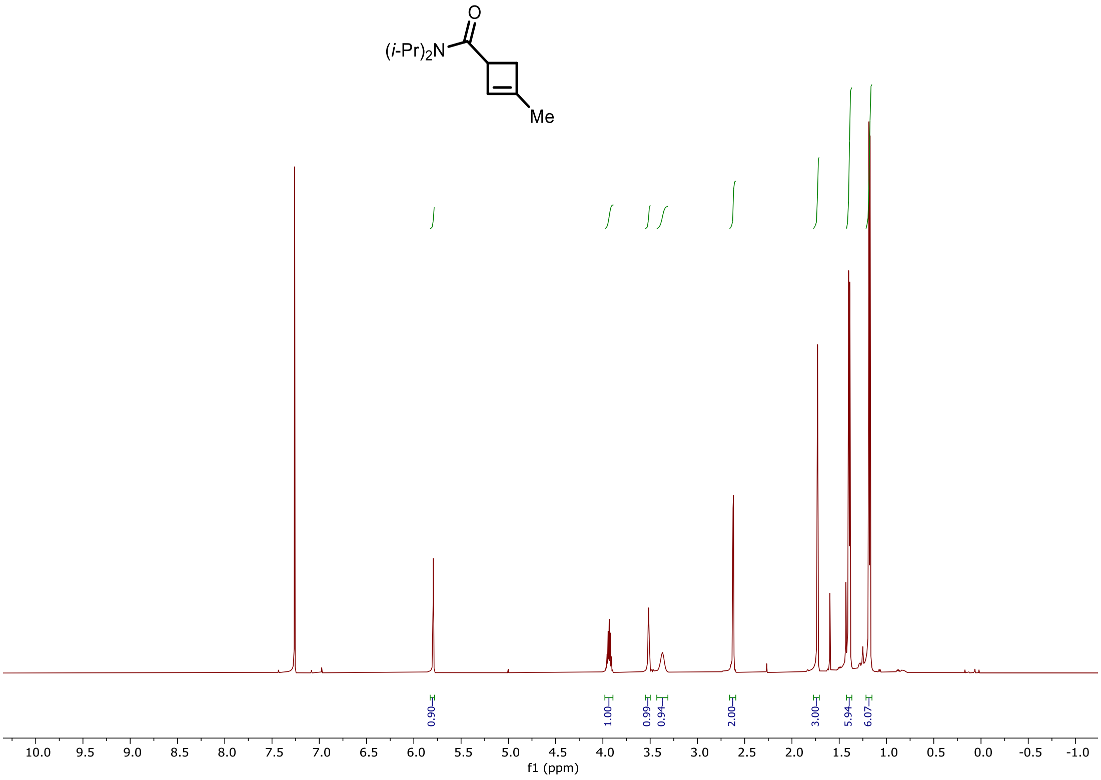

^{13}C NMR (151 MHz, Chloroform-*d*)

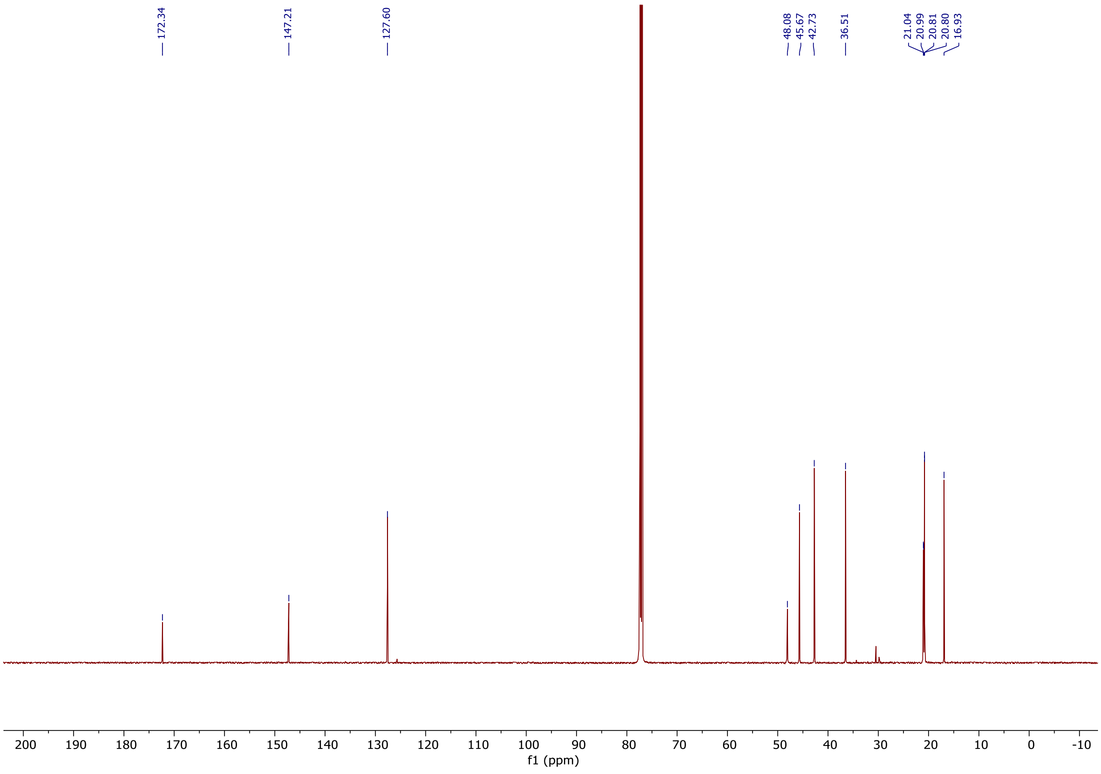

^{1}H COSY (600 MHz, Chloroform-*d*)

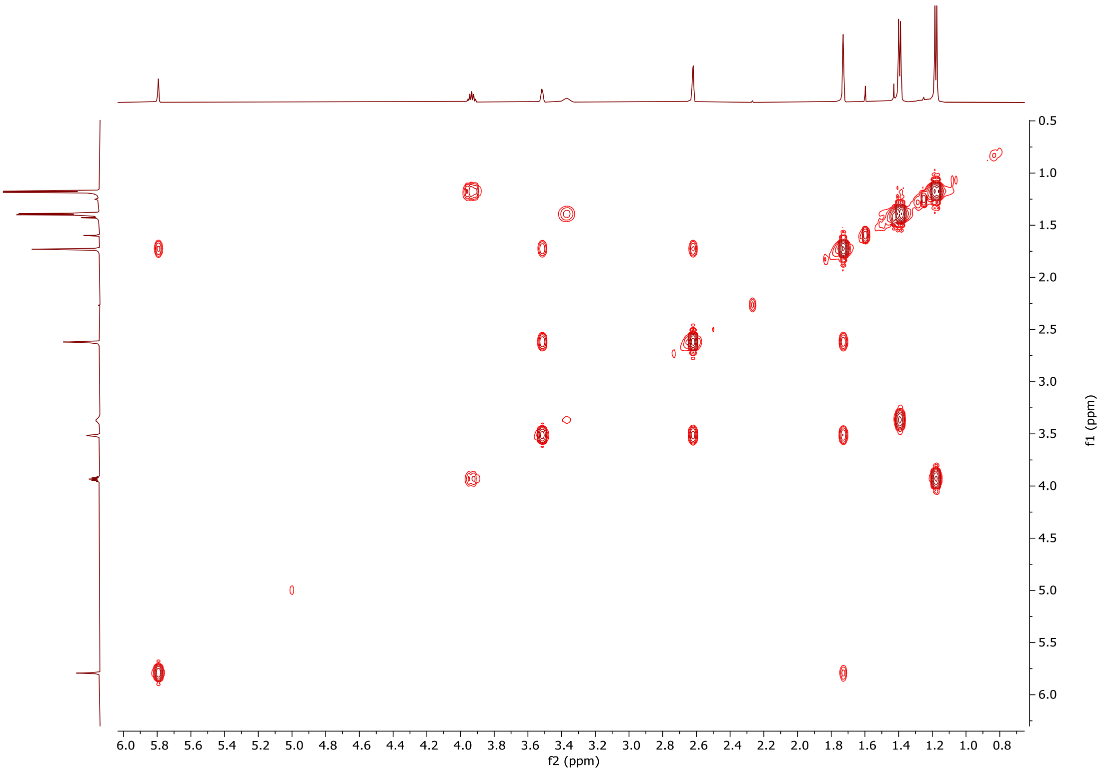

^{1}H/^{13}C HSQC (600/151 MHz, Chloroform-*d*)

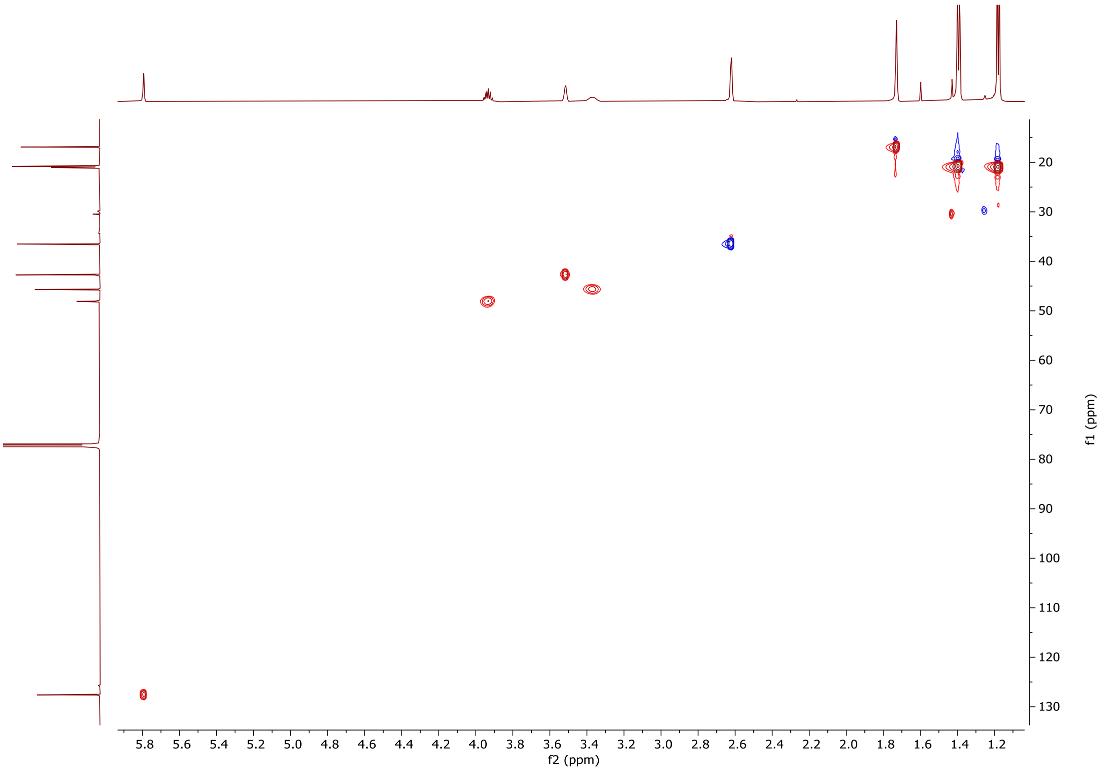

^{1}H/^{13}C HMBC (600/151 MHz, Chloroform-*d*)

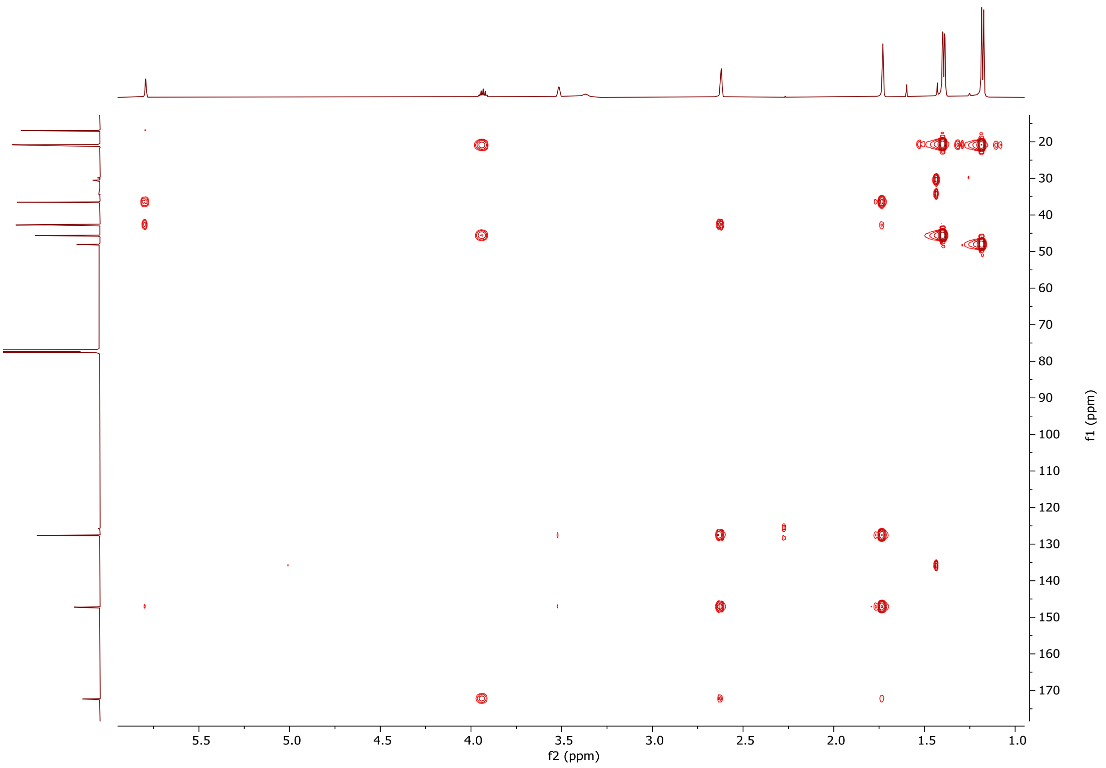

_N_,_N_-diisopropyl-3-methyl-2-(trimethylsilyl)cyclobut-2-ene-1-carboxamide, 2e

^{1}H NMR (600 MHz, Chloroform-*d*)

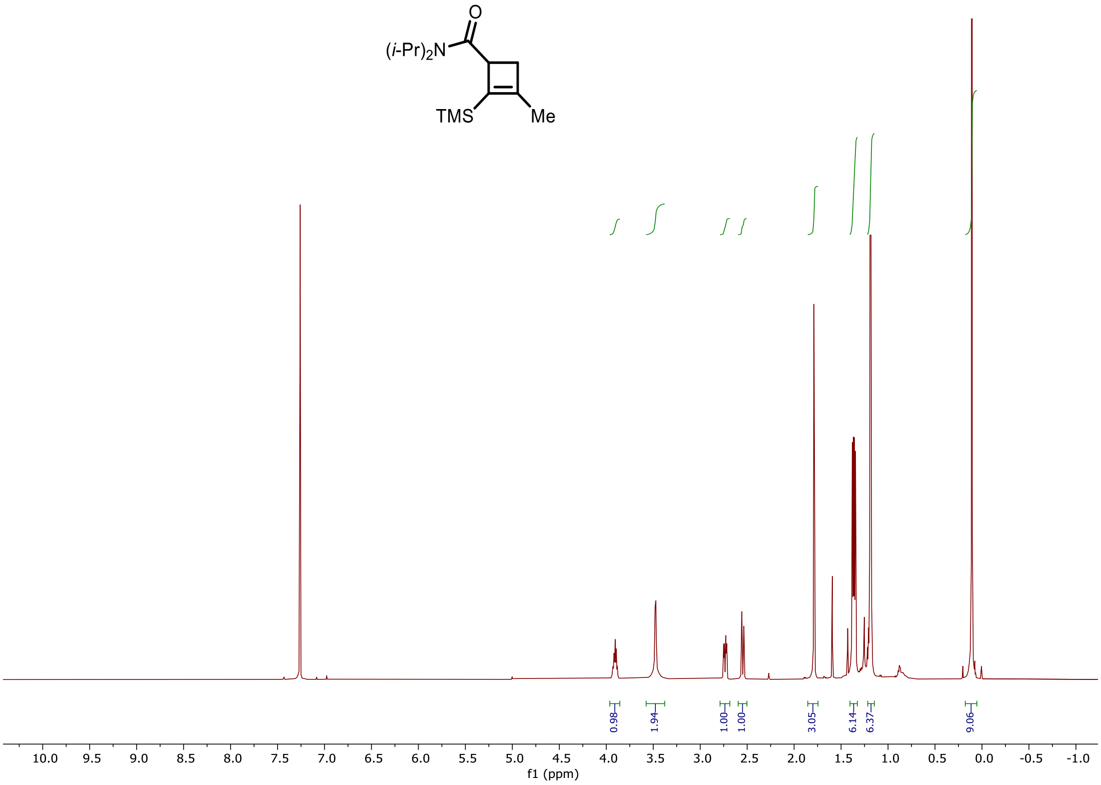

^{13}C NMR (151 MHz, Chloroform-*d*)

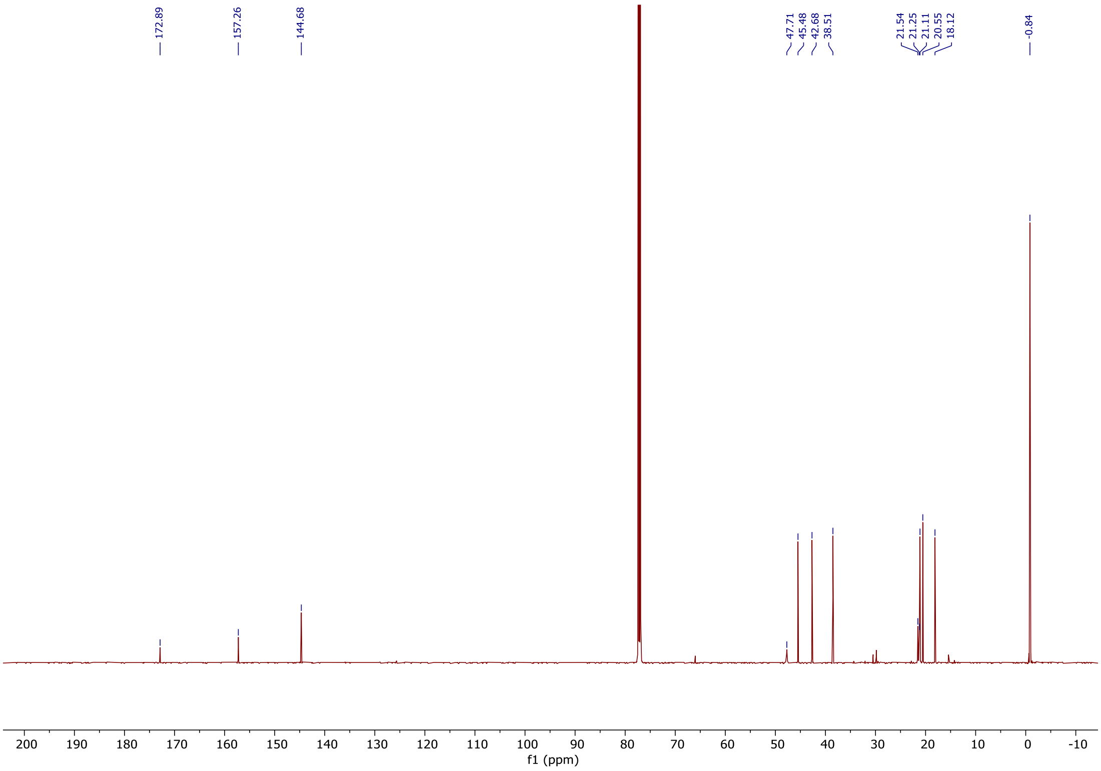

^{1}H COSY (600 MHz, Chloroform-*d*)

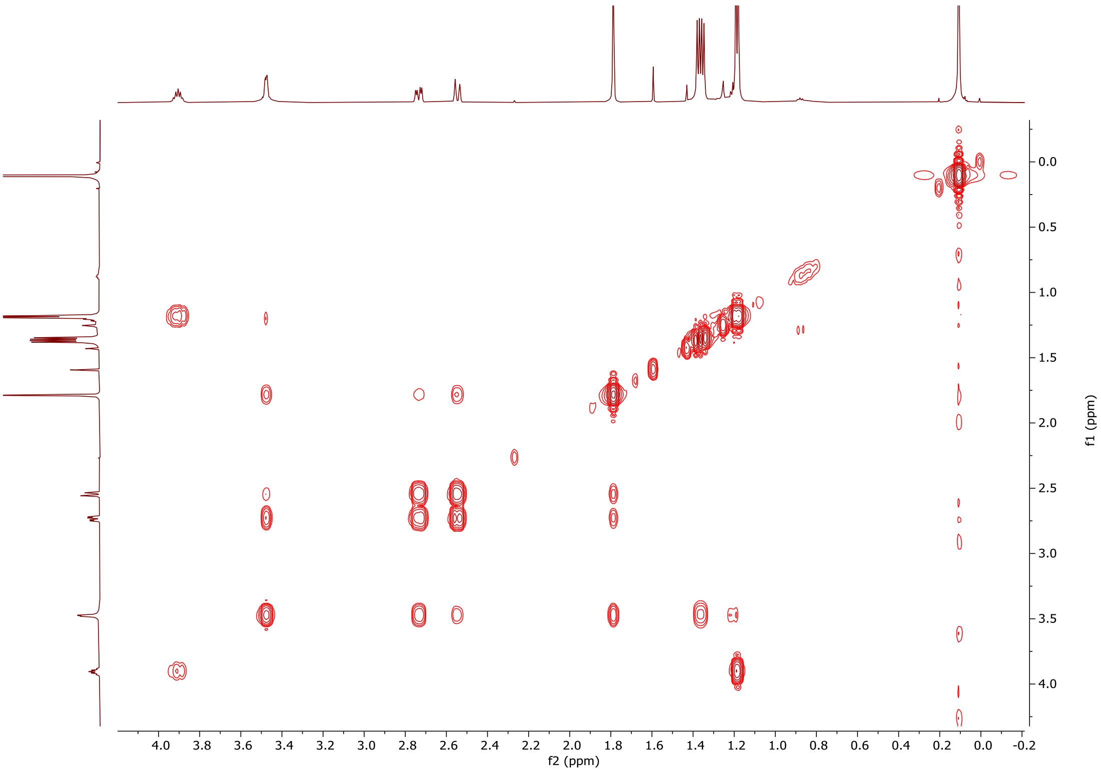

^{1}H/^{13}C HSQC (600/151 MHz, Chloroform-*d*)

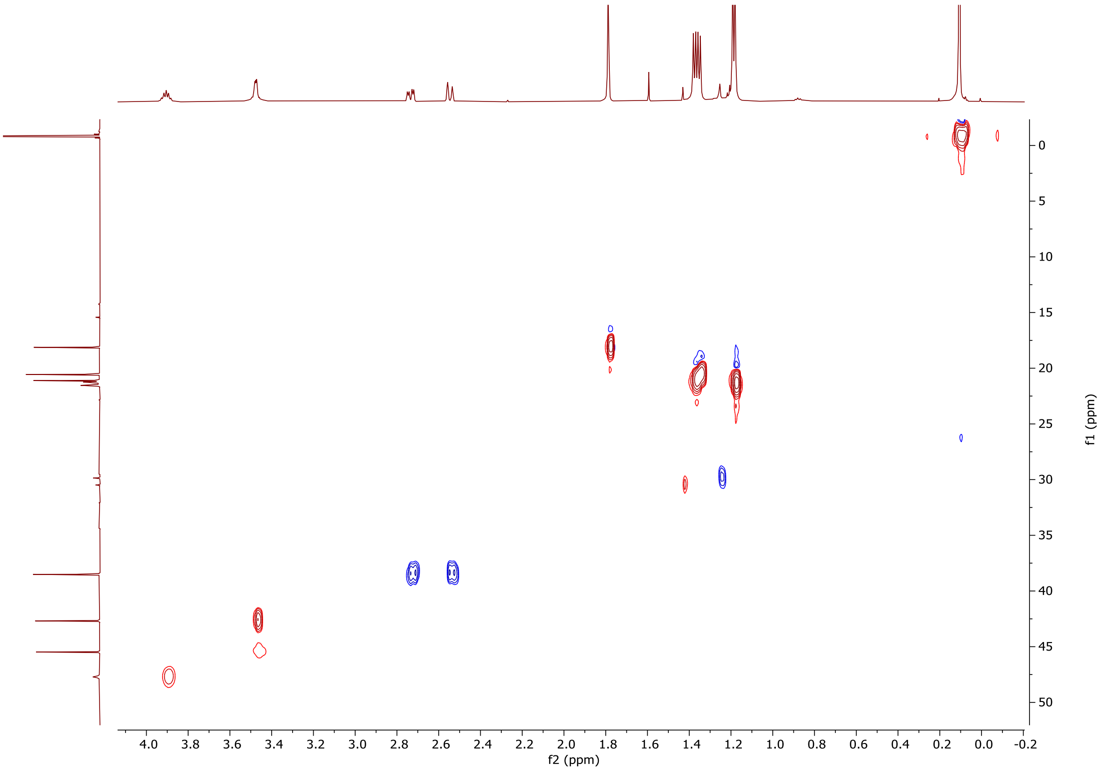

$^{1}H/^{13}C$ **HMBC** (600/151 MHz, Chloroform-*d*)

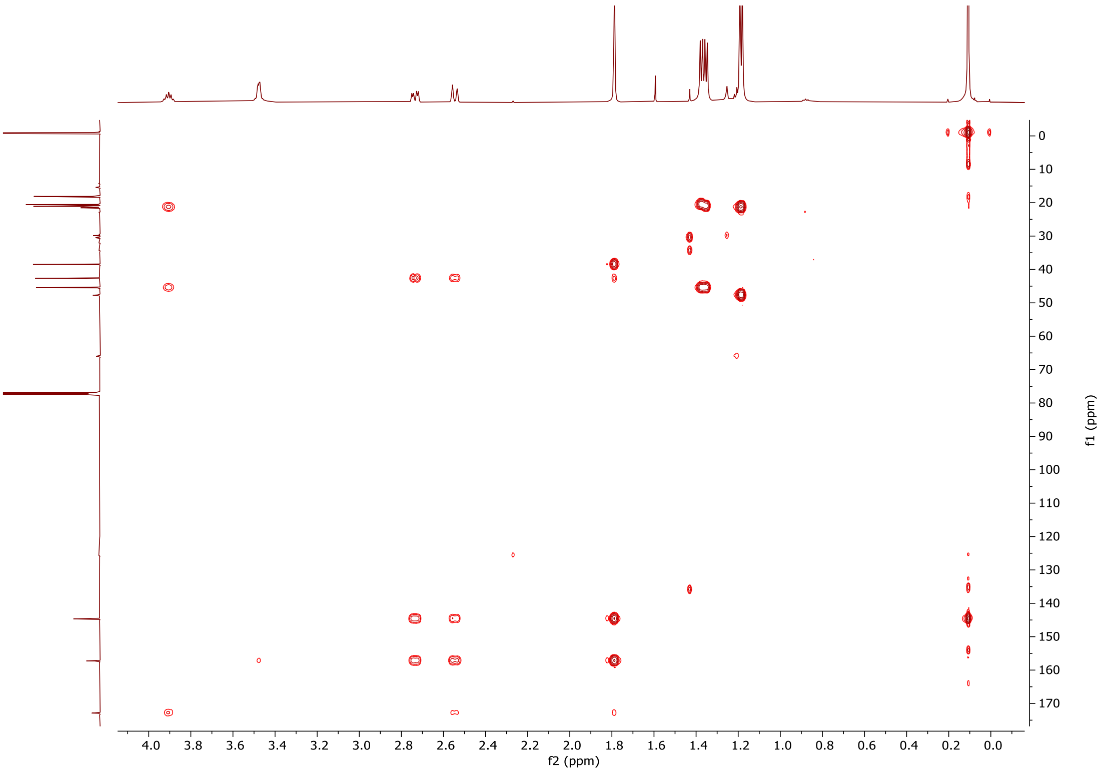

(*S)-1-((1*R**,2*R**)-2-(diisopropylcarbamoyl)-1-methylcyclopropyl)ethyl acetate, 3b**

^{1}H NMR (600 MHz, Chloroform-*d*)

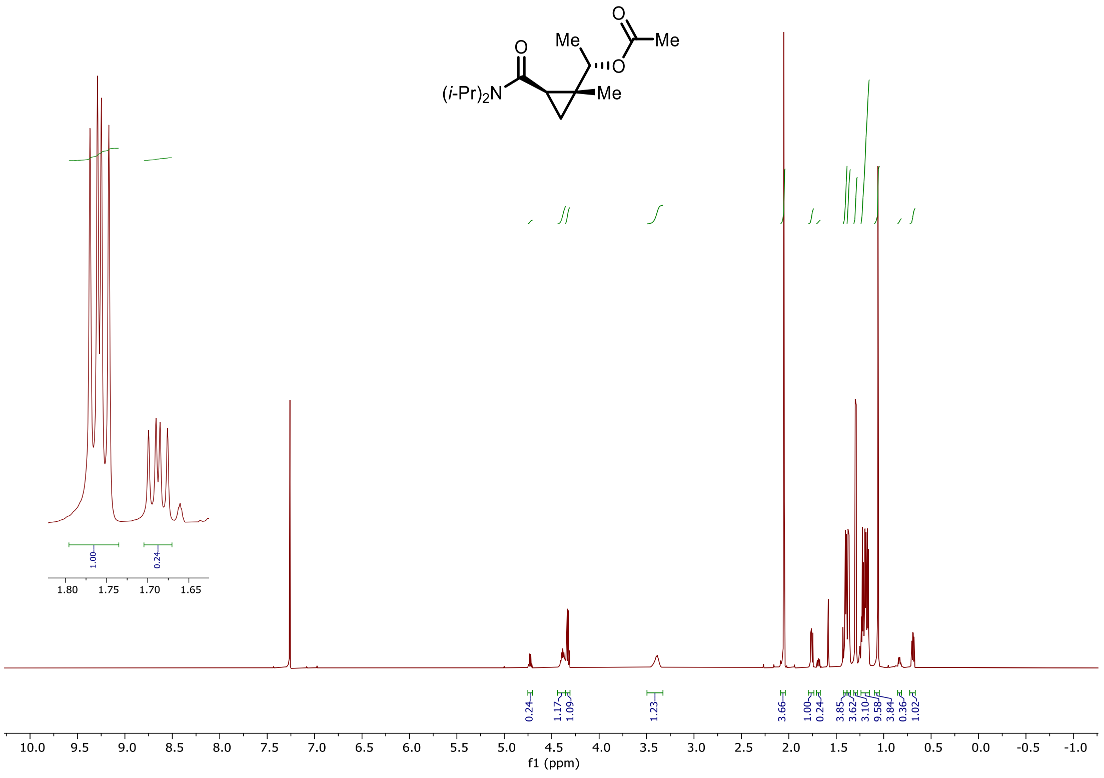

^{13}C NMR (151 MHz, Chloroform-*d*)

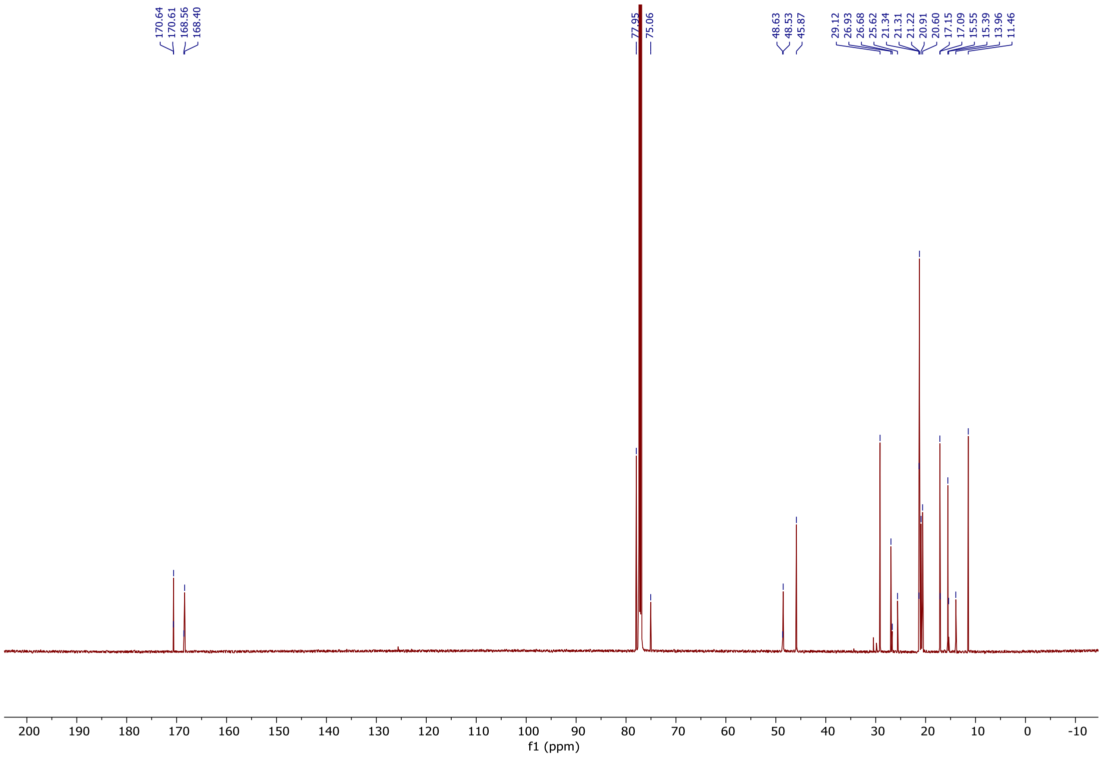

^{1}H COSY (600 MHz, Chloroform-*d*)

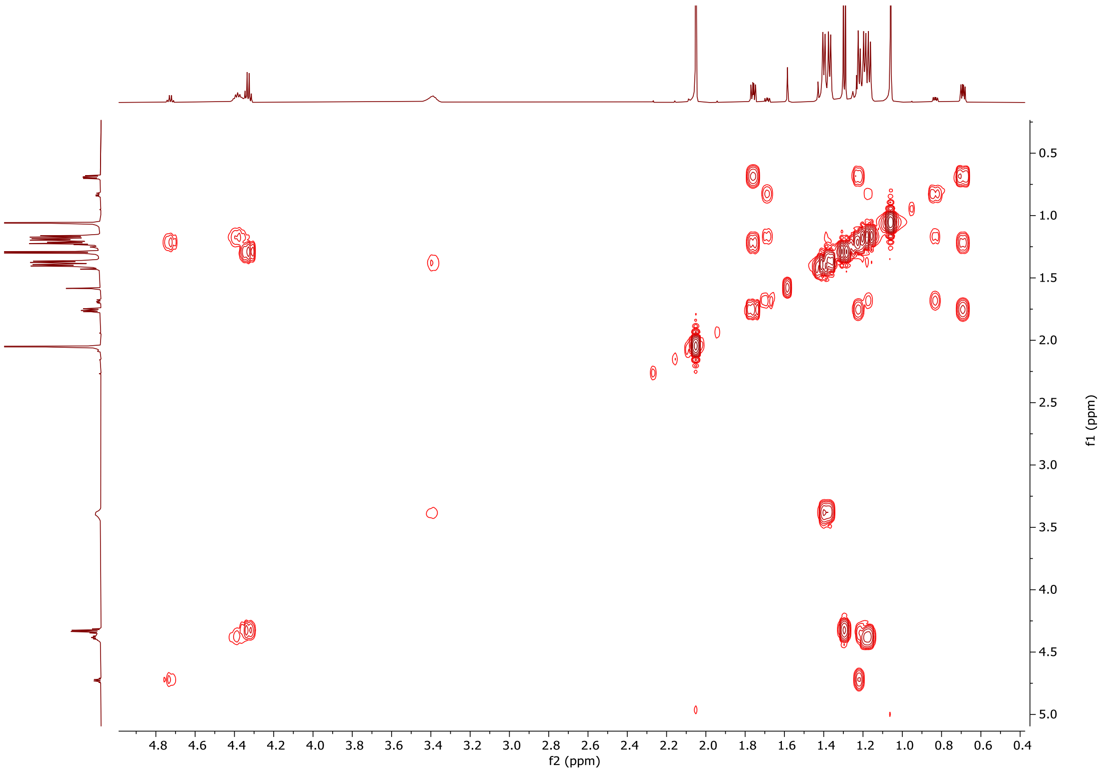

^{1}H/^{13}C HSQC (600/151 MHz, Chloroform-*d*)

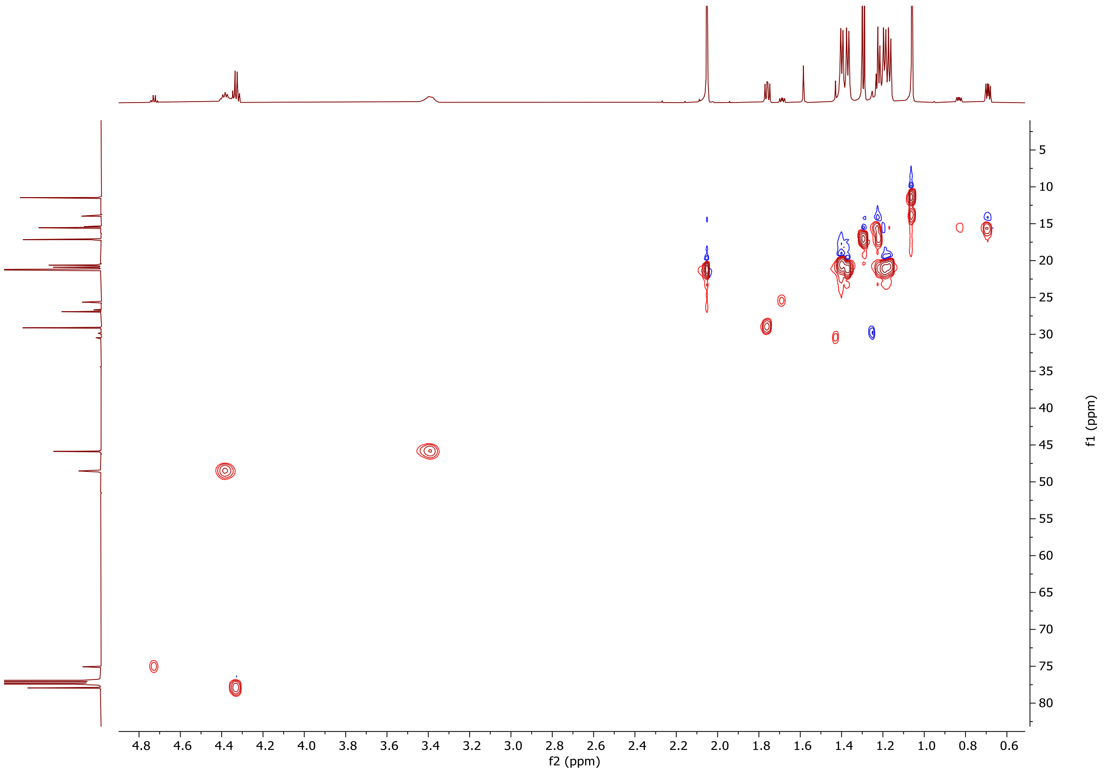

^{1}H/^{13}C HMBC (600/151 MHz, Chloroform-*d*)

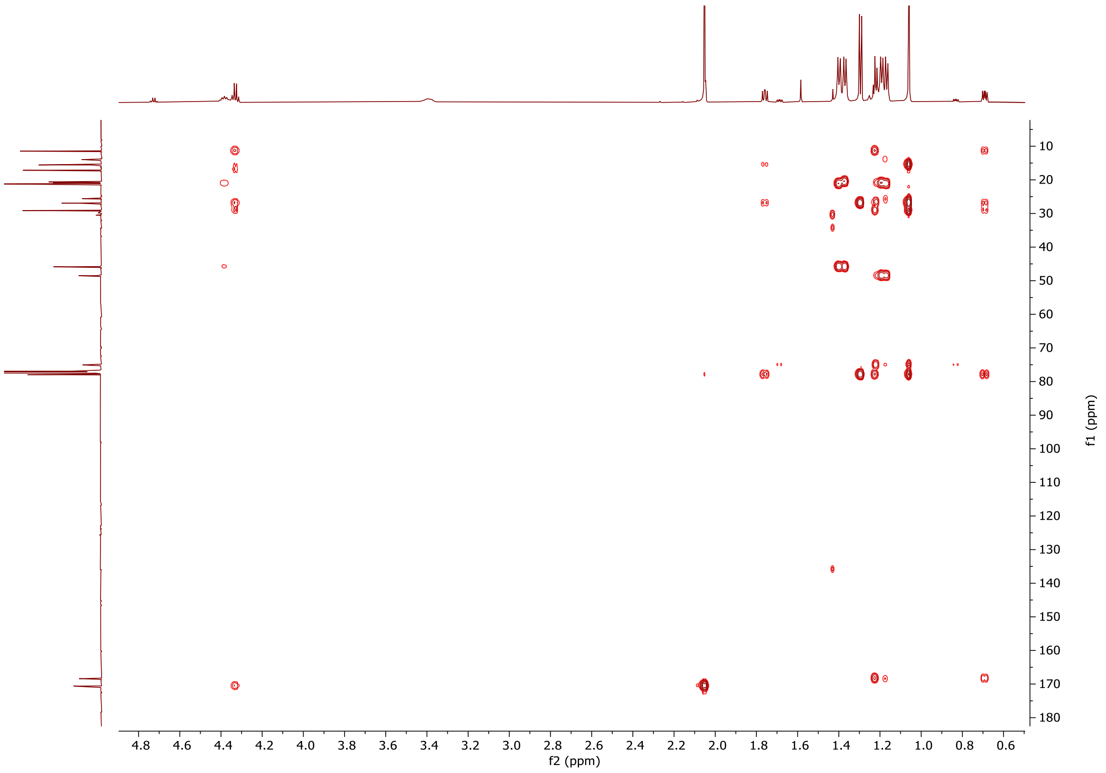

^{1}H NOSEY (600 MHz, Chloroform-*d*)

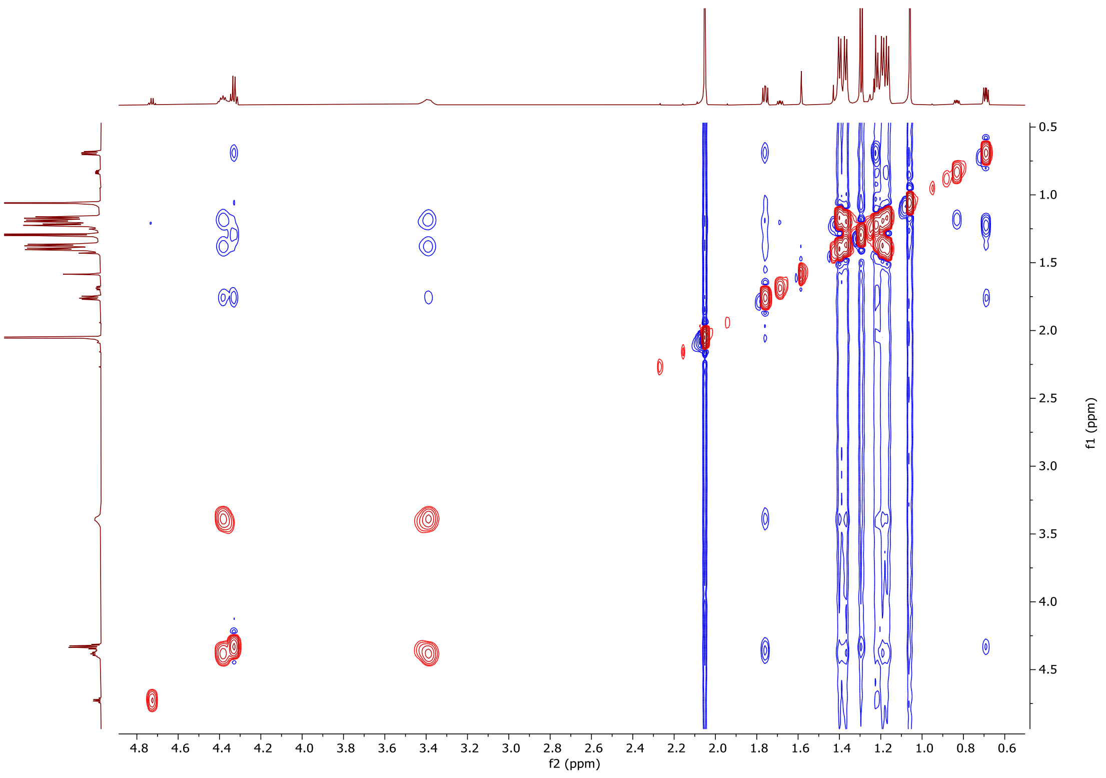

(*S)-1-((1*R**,2*R**)-2-(diisopropylcarbamoyl)-1-methylcyclopropyl)ethyl 2,2,2-trichloroacetate, 3c**

^{1}H NMR (600 MHz, Chloroform-*d*)

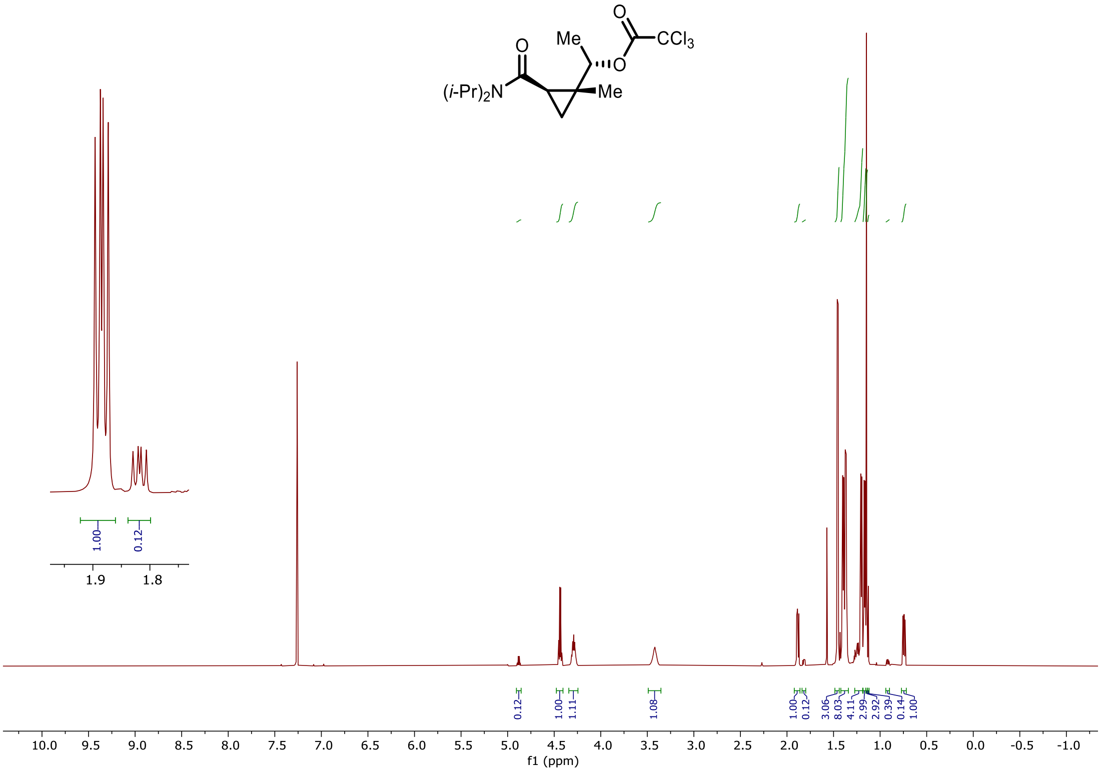

^{13}C NMR (151 MHz, Chloroform-*d*)

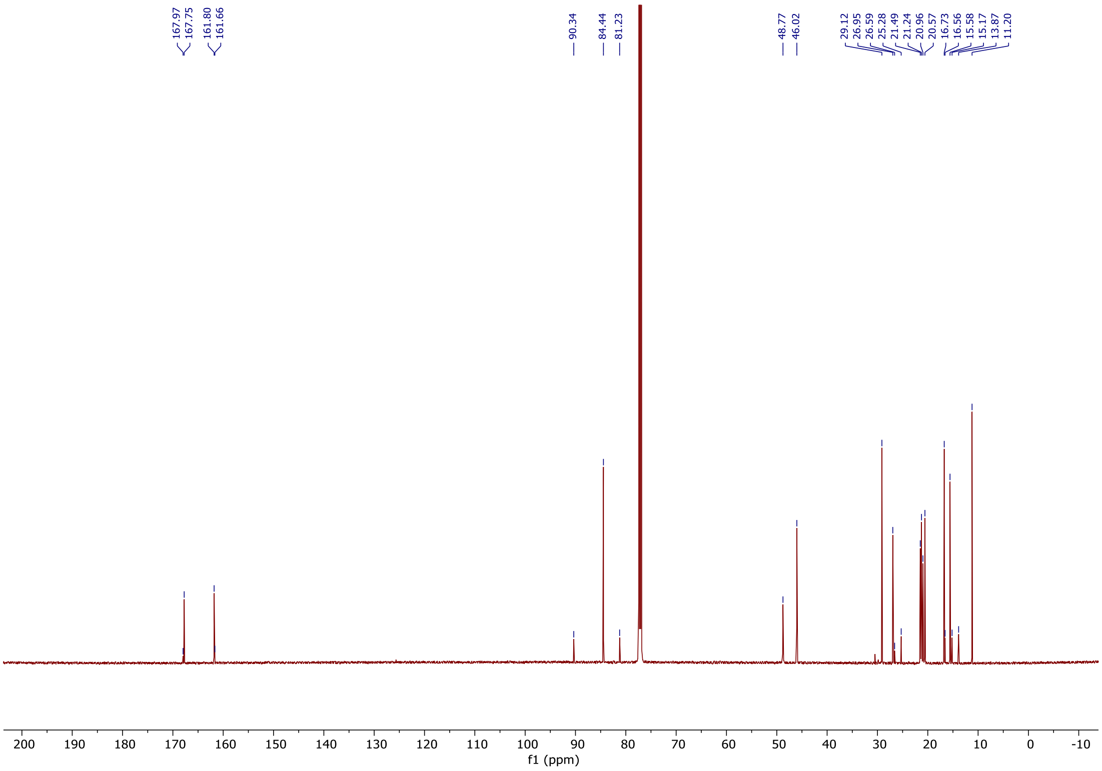

^{1}H COSY (600 MHz, Chloroform-*d*)

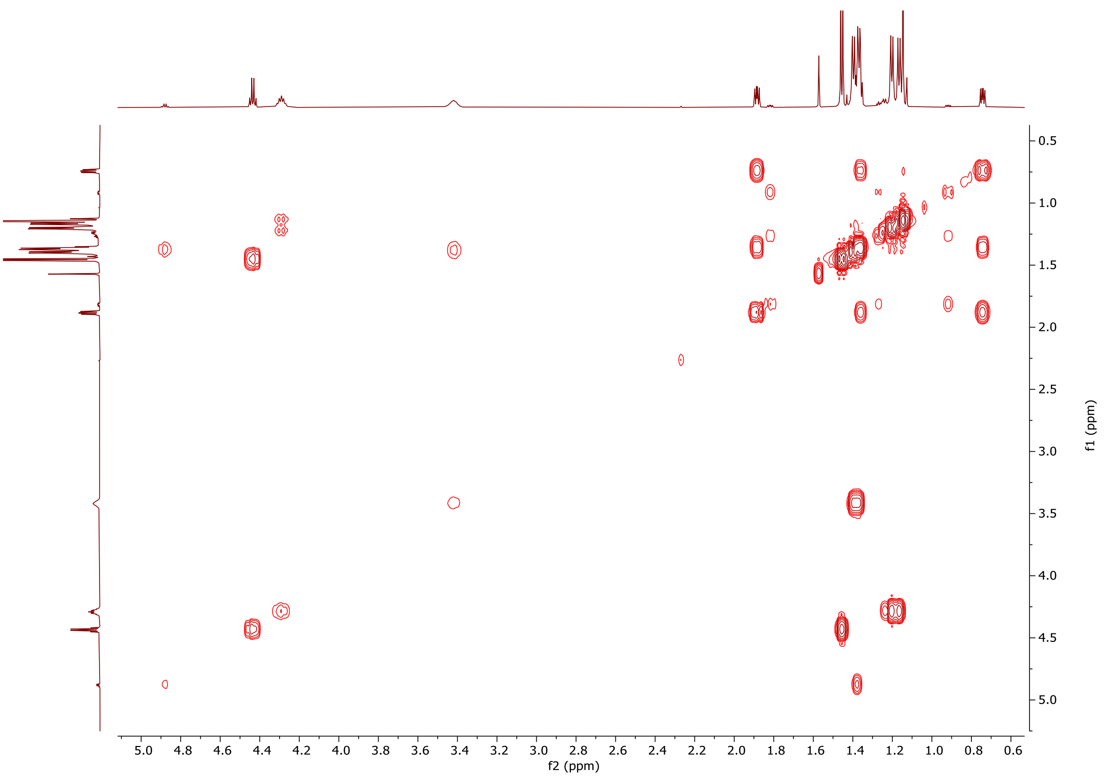

^{1}H/^{13}C HSQC (600/151 MHz, Chloroform-*d*)

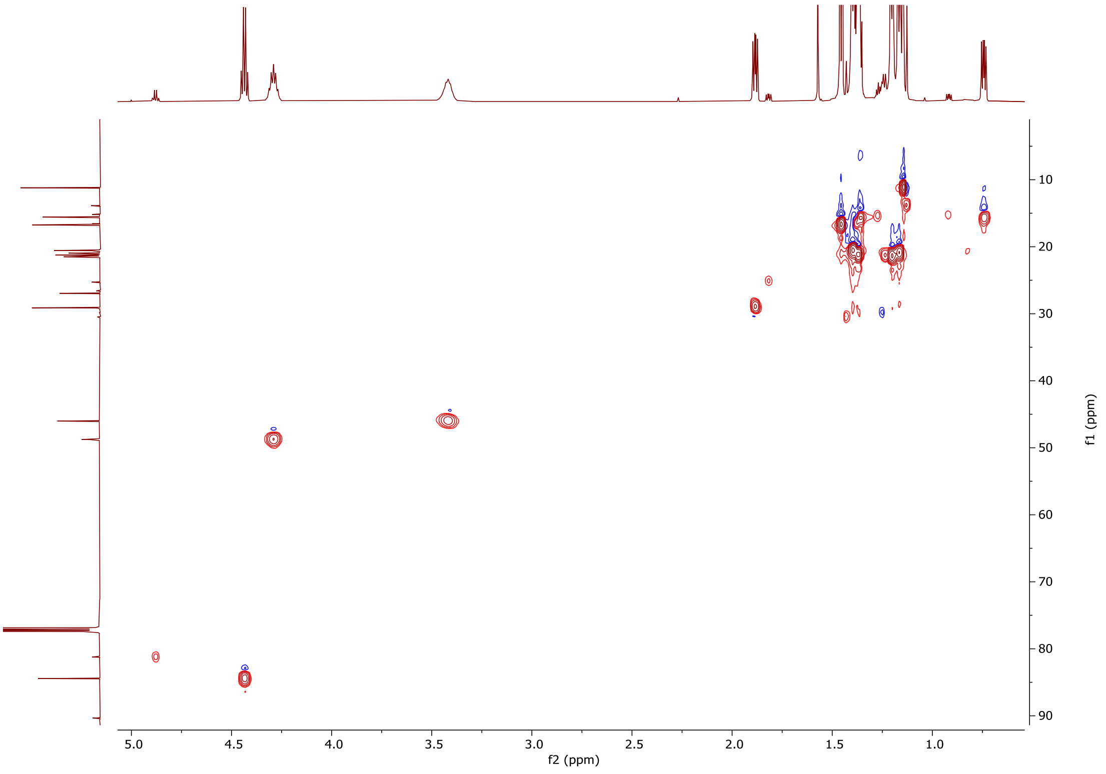

$^{1}H/^{13}C$ HMBC (600/151 MHz, Chloroform-*d*)

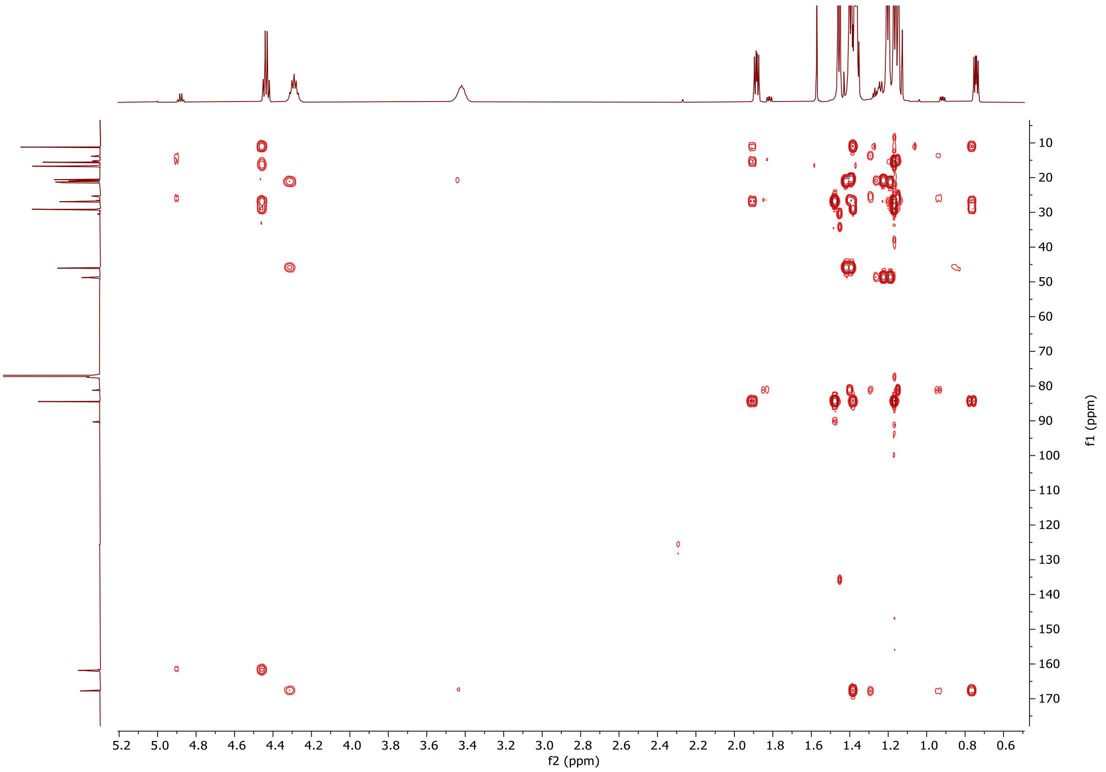

^{1}H NOSEY (600 MHz, Chloroform-*d*)

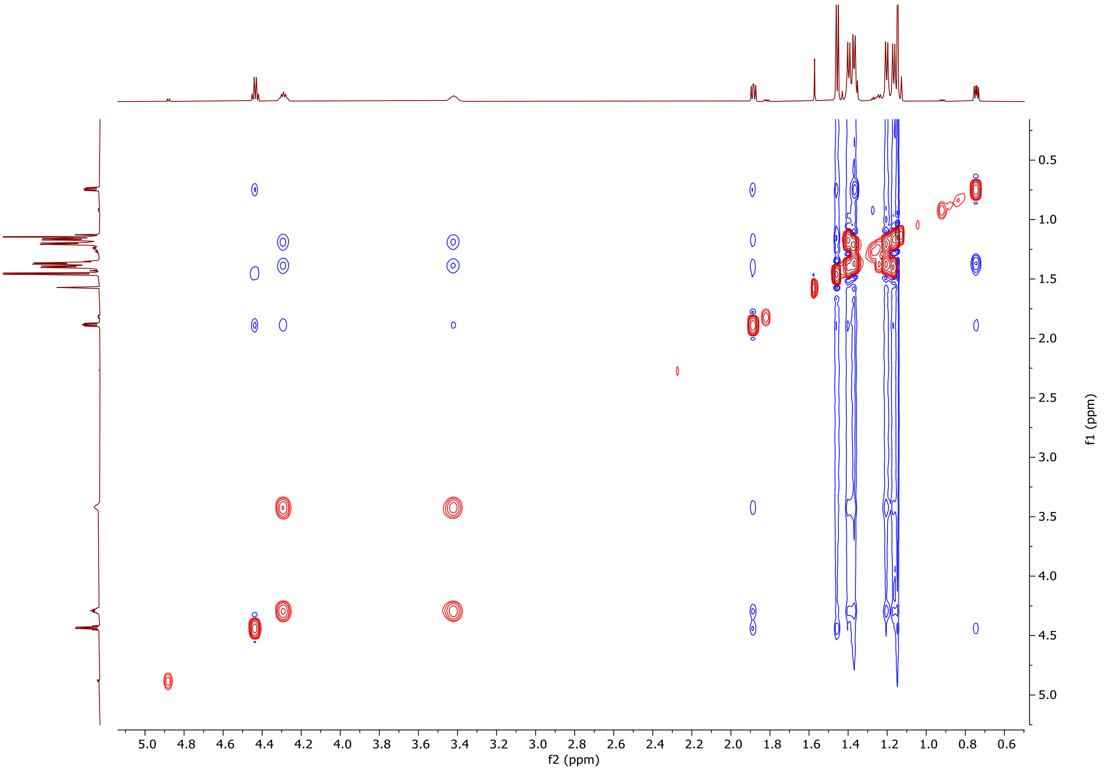

(*S)-1-((1*R**,2*R**)-2-(diisopropylcarbamoyl)-1-methylcyclopropyl)ethyl 2,2,2-trifluoroacetate, 3d**

^{1}H NMR (600 MHz, Chloroform-*d*)

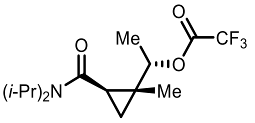

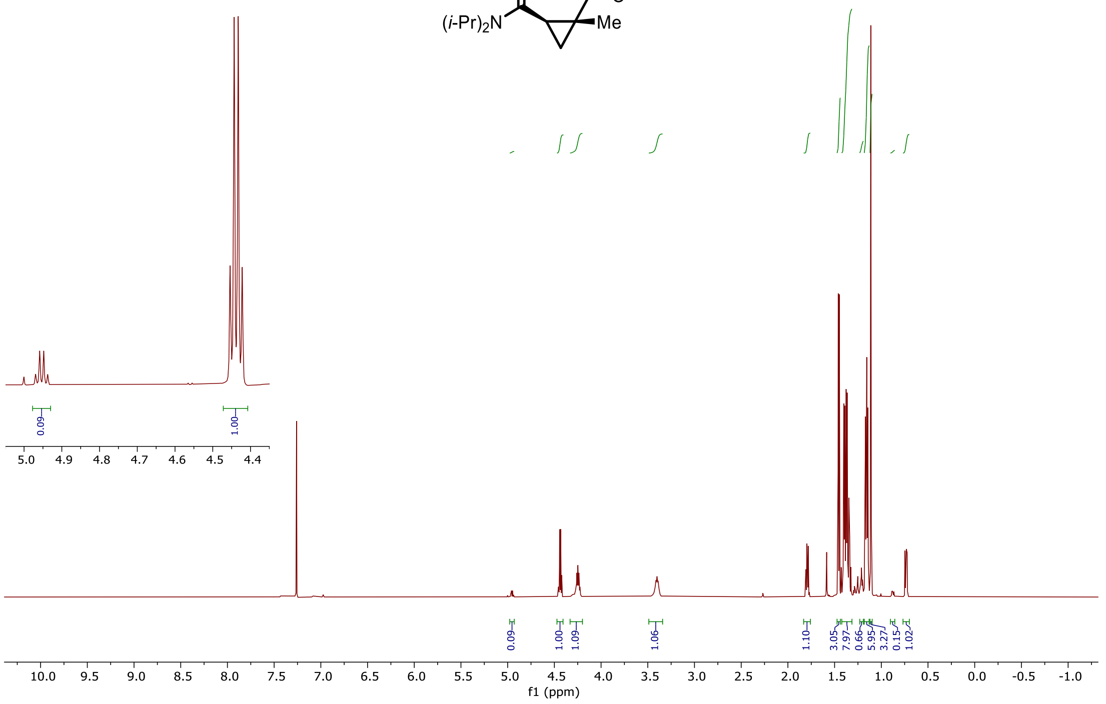

^{13}C NMR (151 MHz, Chloroform-*d*)

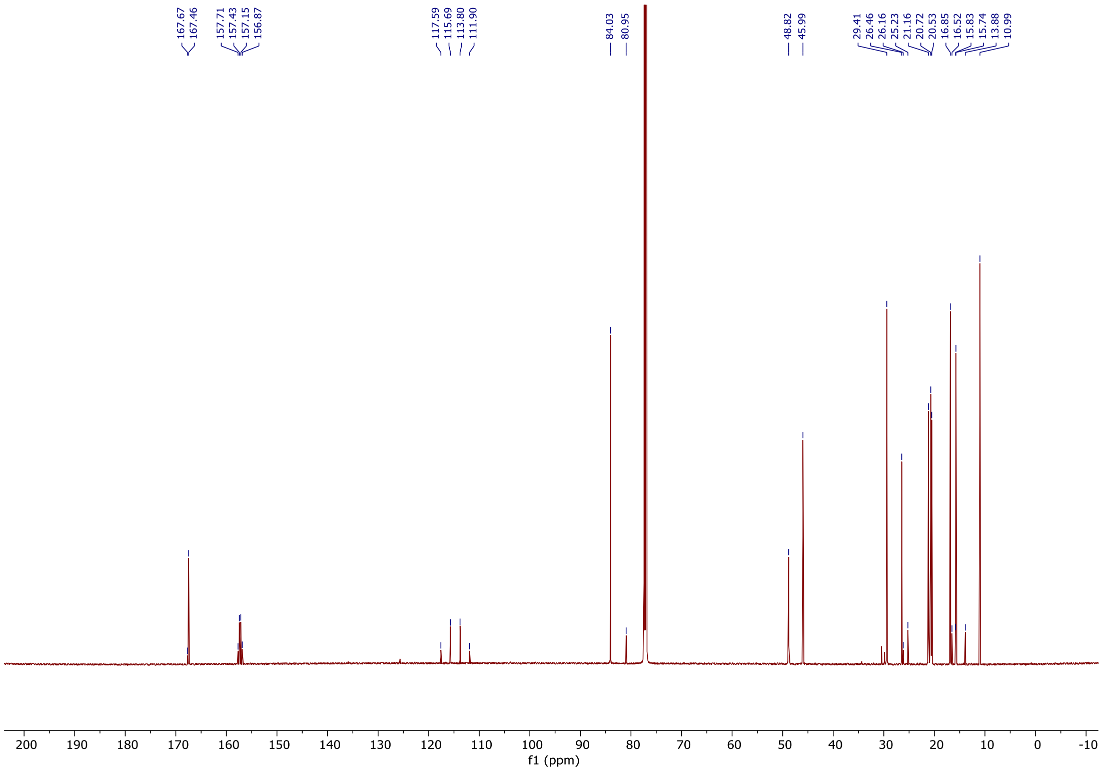

^{1}H COSY (600 MHz, Chloroform-*d*)

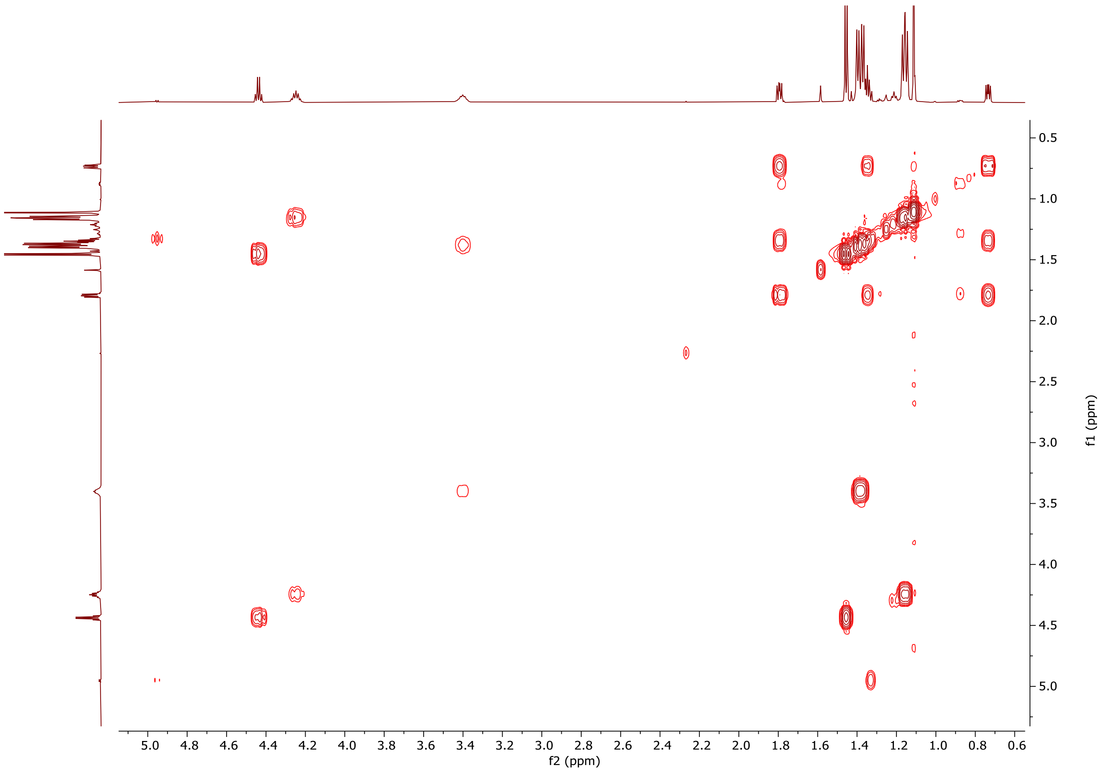

^{1}H/^{13}C HSQC (600/151 MHz, Chloroform-*d*)

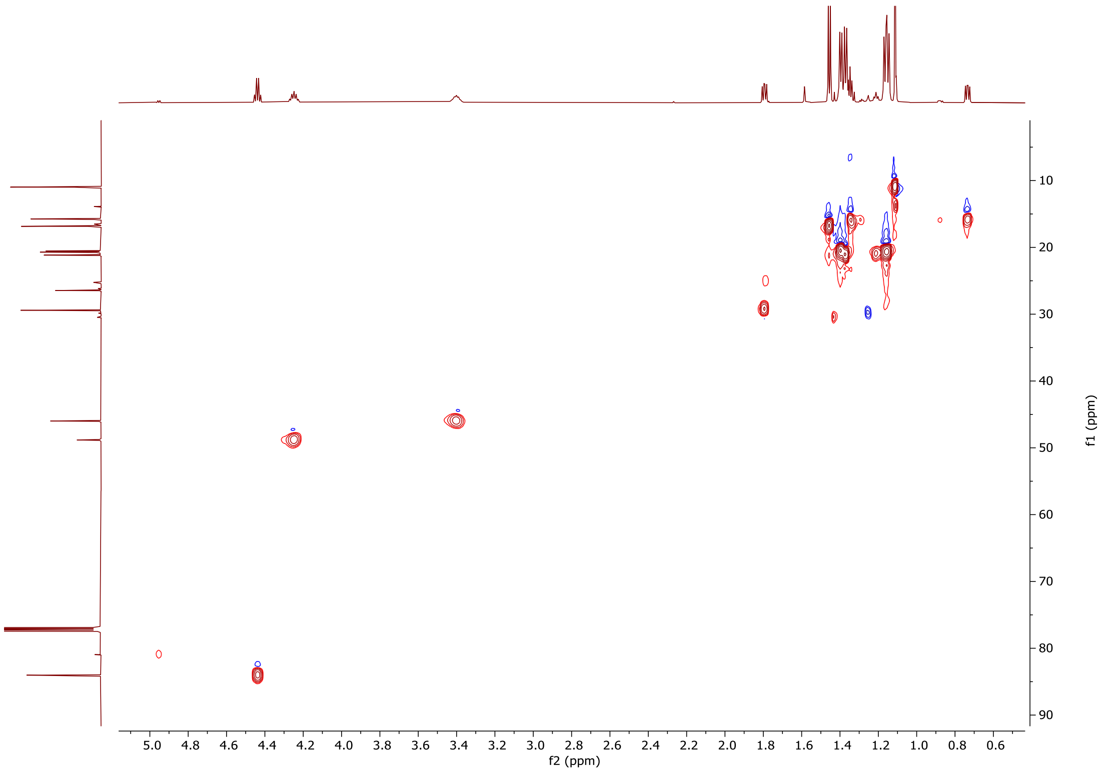

$^{1}H/^{13}C$ **HMBC** (600/151 MHz, Chloroform-*d*)

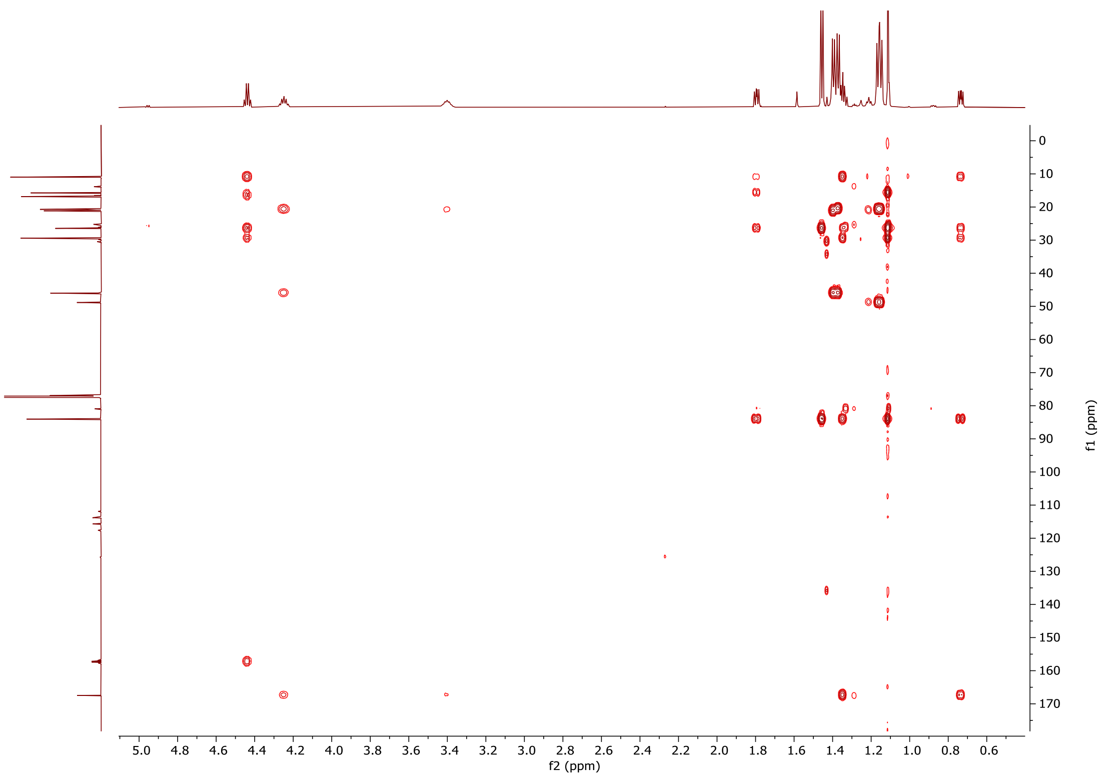

^{1}H **NOSEY** (600 MHz, Chloroform-*d*)

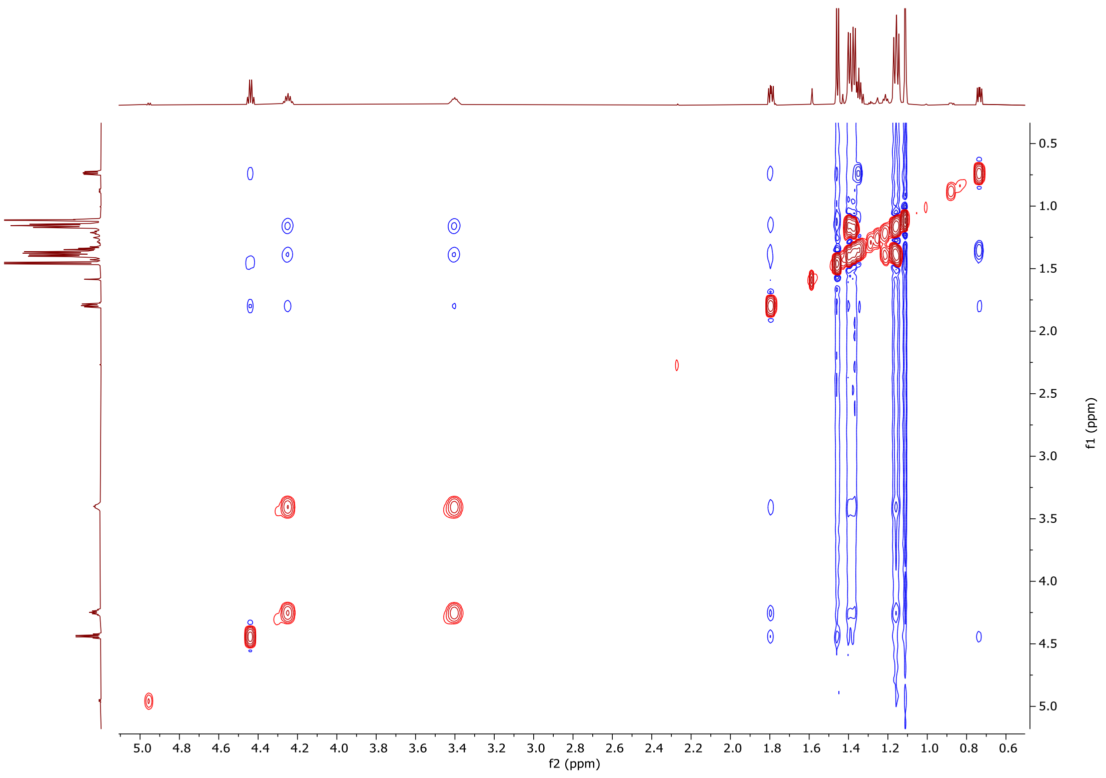

^{19}F NMR (565 MHz, Chloroform-*d*)

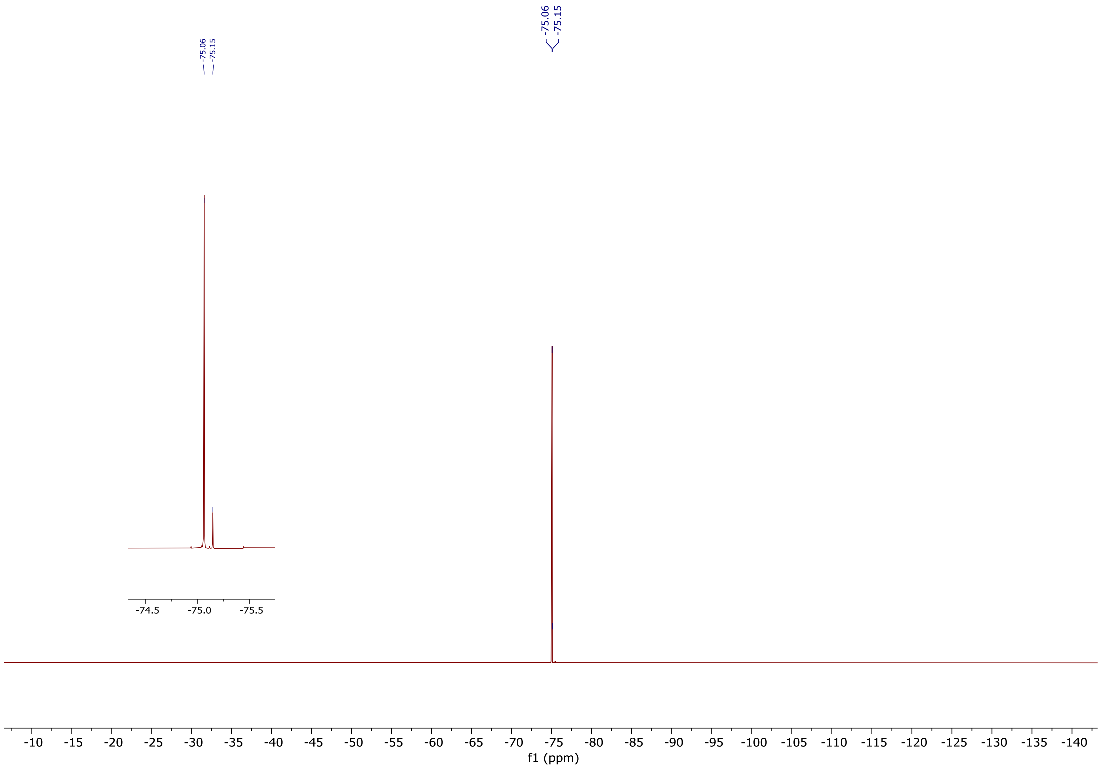

(*R)-1-((1*R**,2*R**)-2-(diisopropylcarbamoyl)-1-methylcyclopropyl)ethyl 4-nitrobenzoate, 3e**

^{1}H NMR (600 MHz, Chloroform-*d*)

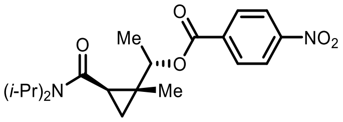

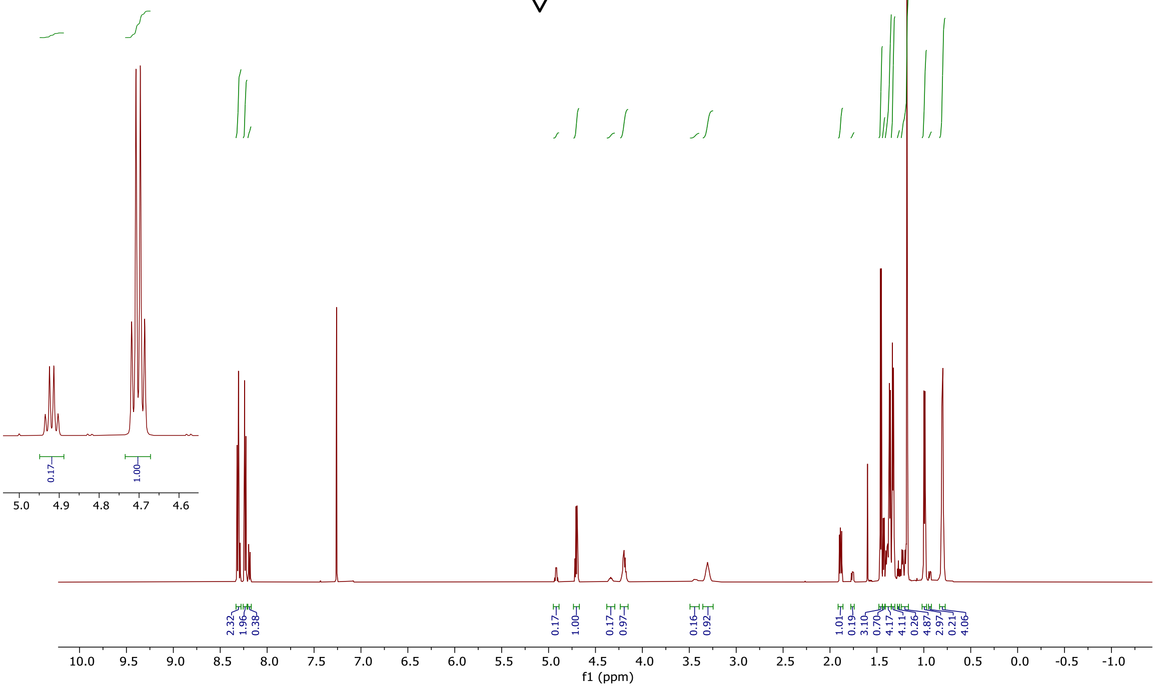

^{13}C NMR (151 MHz, Chloroform-*d*)

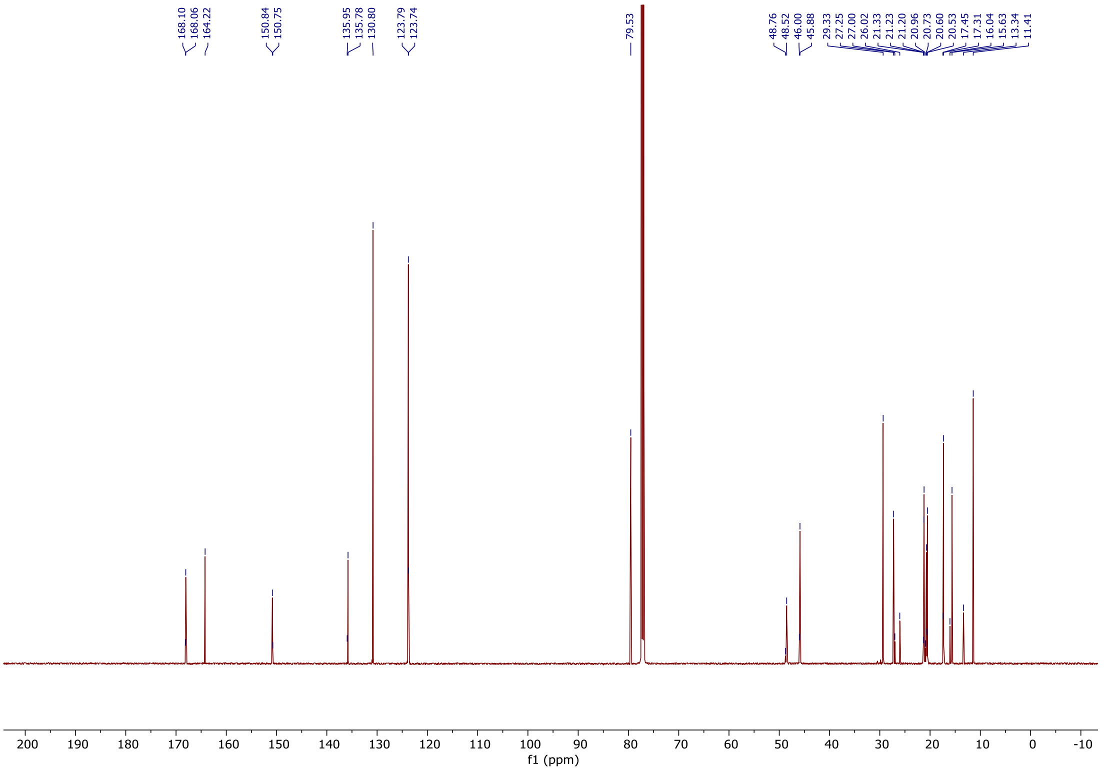

^{1}H COSY (600 MHz, Chloroform-*d*)

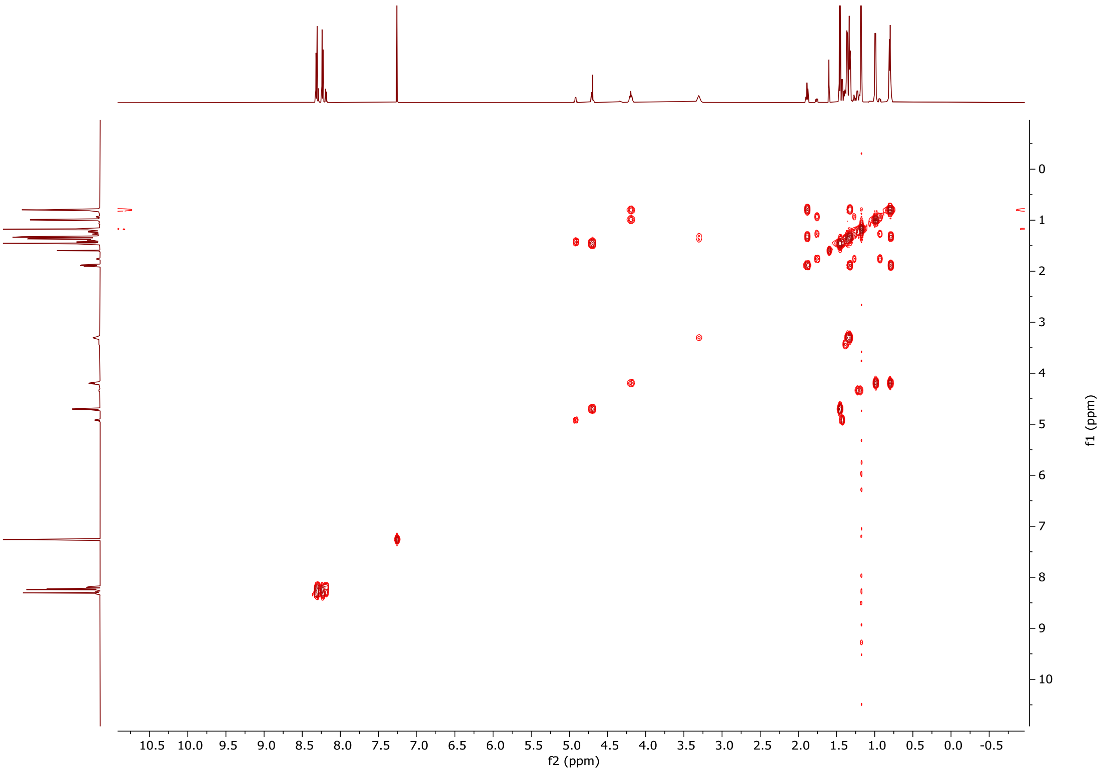

^{1}H/^{13}C HSQC (600/151 MHz, Chloroform-*d*)

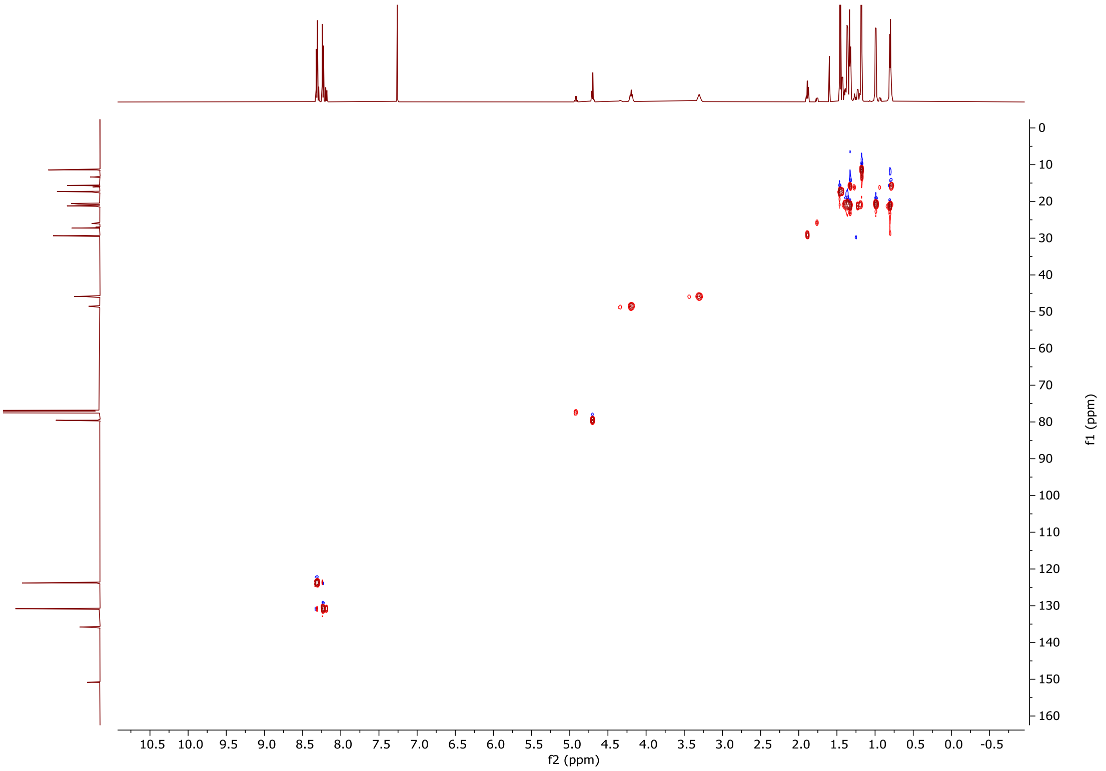

$^{1}H/^{13}C$ HMBC (600/151 MHz, Chloroform-*d*)

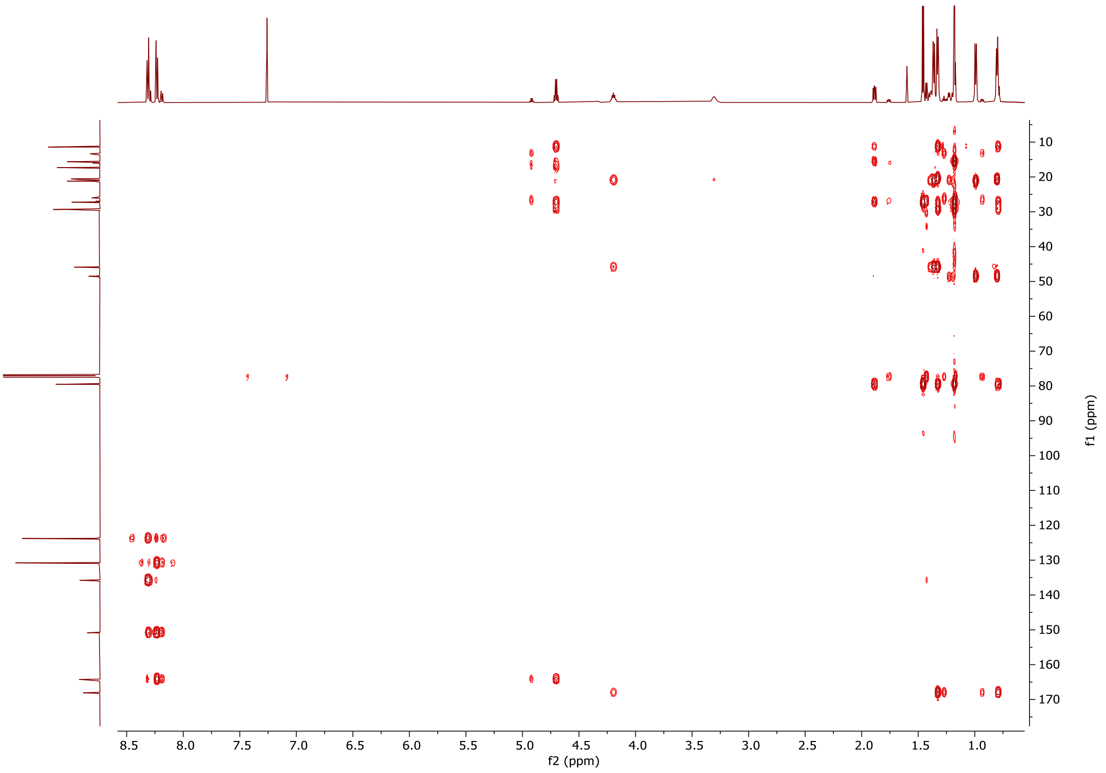

^{1}H NOSEY (600 MHz, Chloroform-*d*)

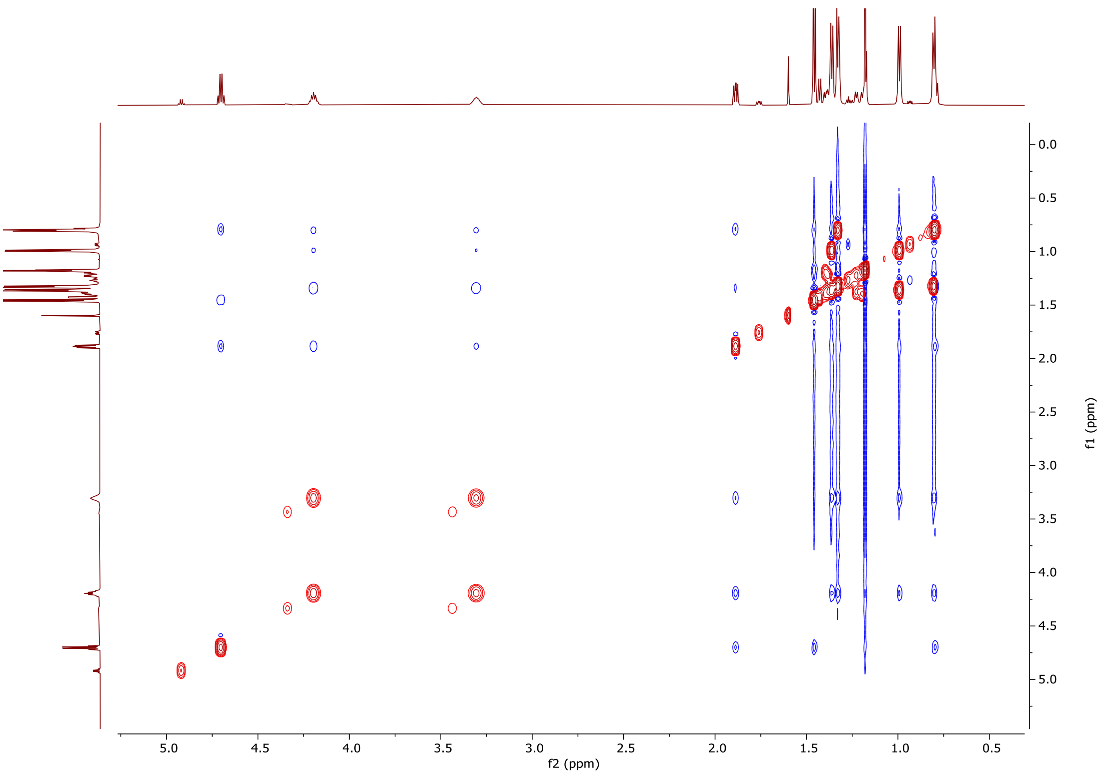

(R^*)-1-((1R^*,2R^*)-2-(diisopropylcarbamoyl)-1-methylcyclopropyl)ethyl 4-methoxybenzoate, 3f

^{1}H NMR (600 MHz, Chloroform-*d*)

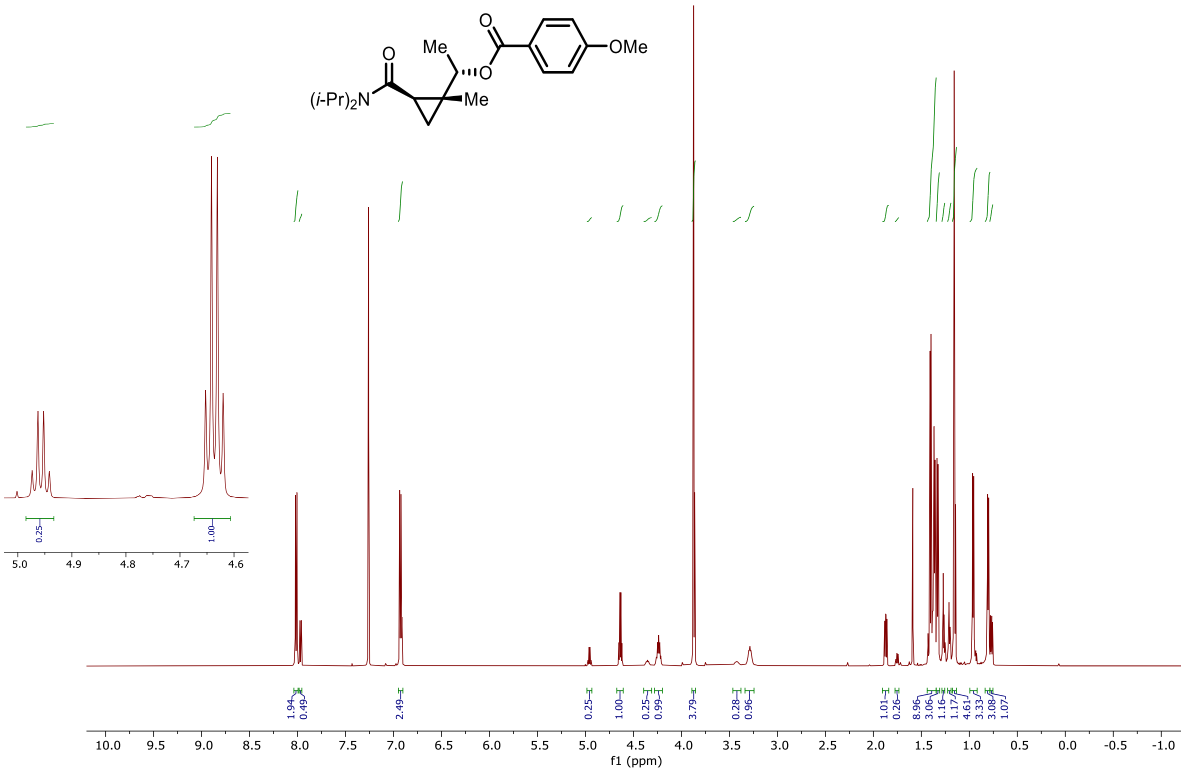

^{13}C NMR (151 MHz, Chloroform-*d*)

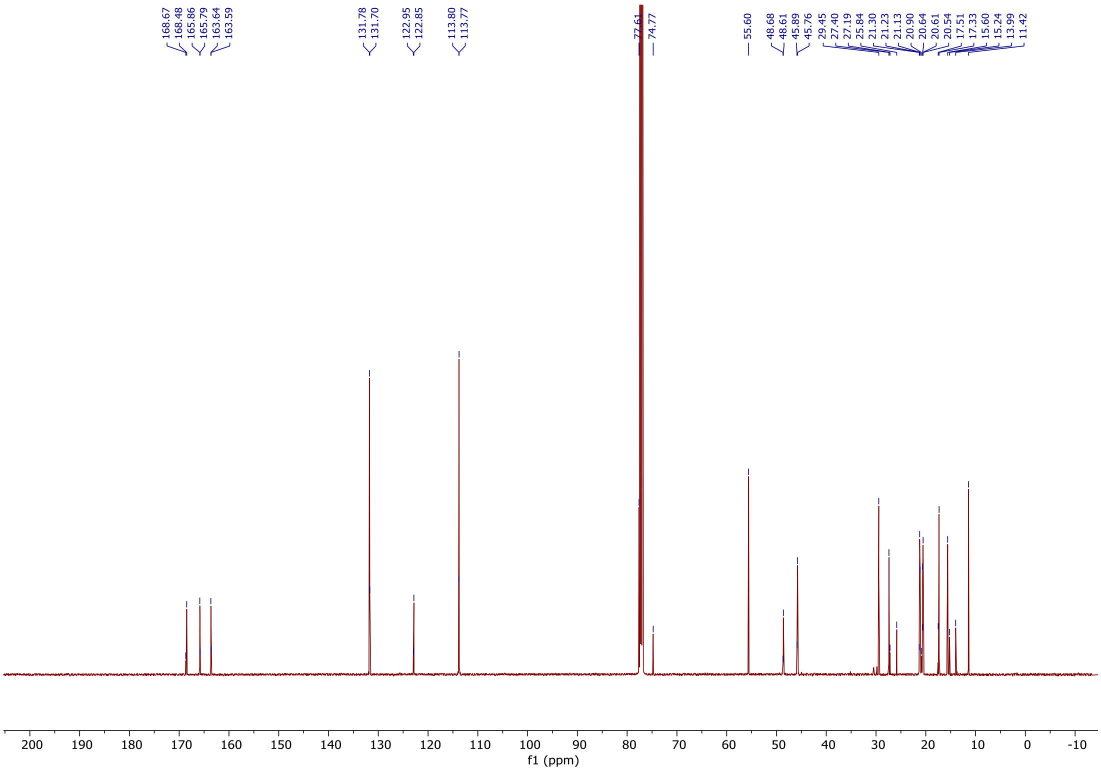

^{1}H COSY (600 MHz, Chloroform-*d*)

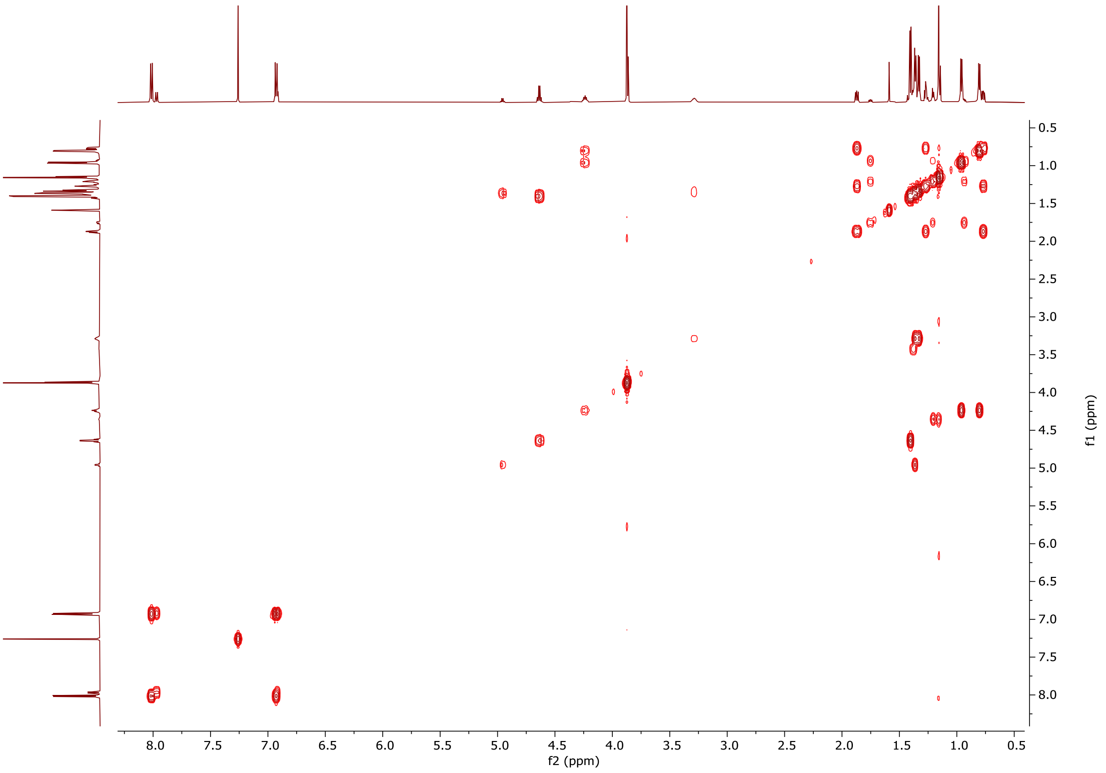

^{1}H/^{13}C HSQC (600/151 MHz, Chloroform-*d*)

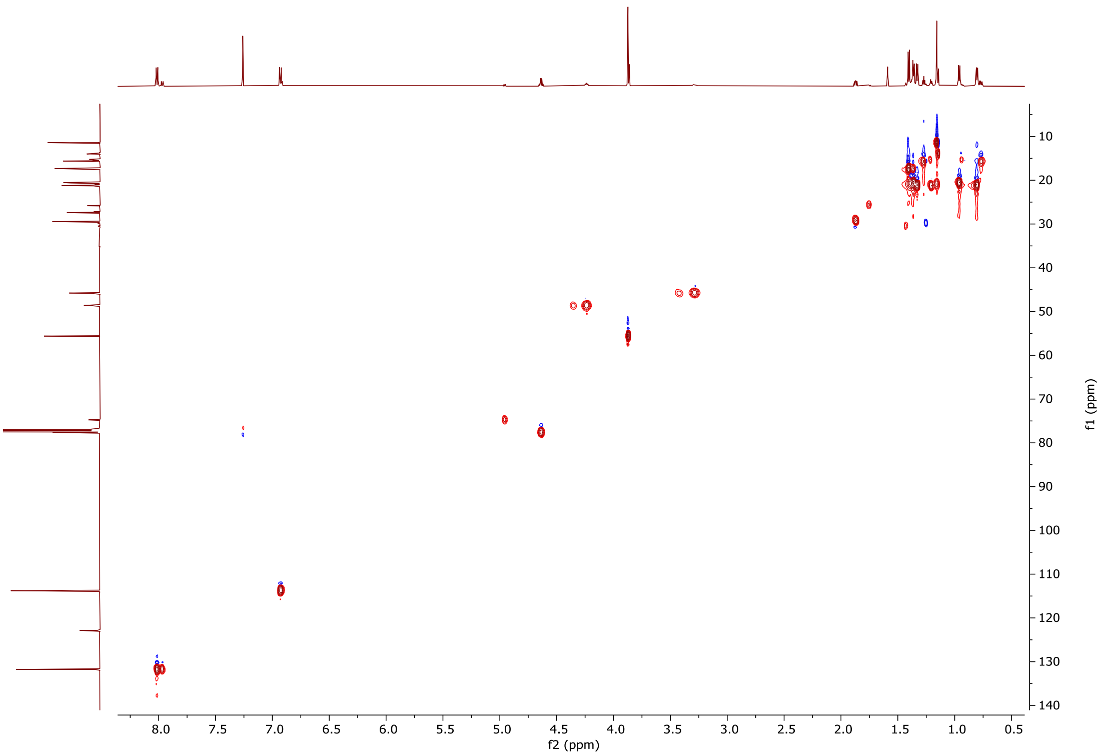

$^{1}H/^{13}C$ **HMBC** (600/151 MHz, Chloroform-*d*)

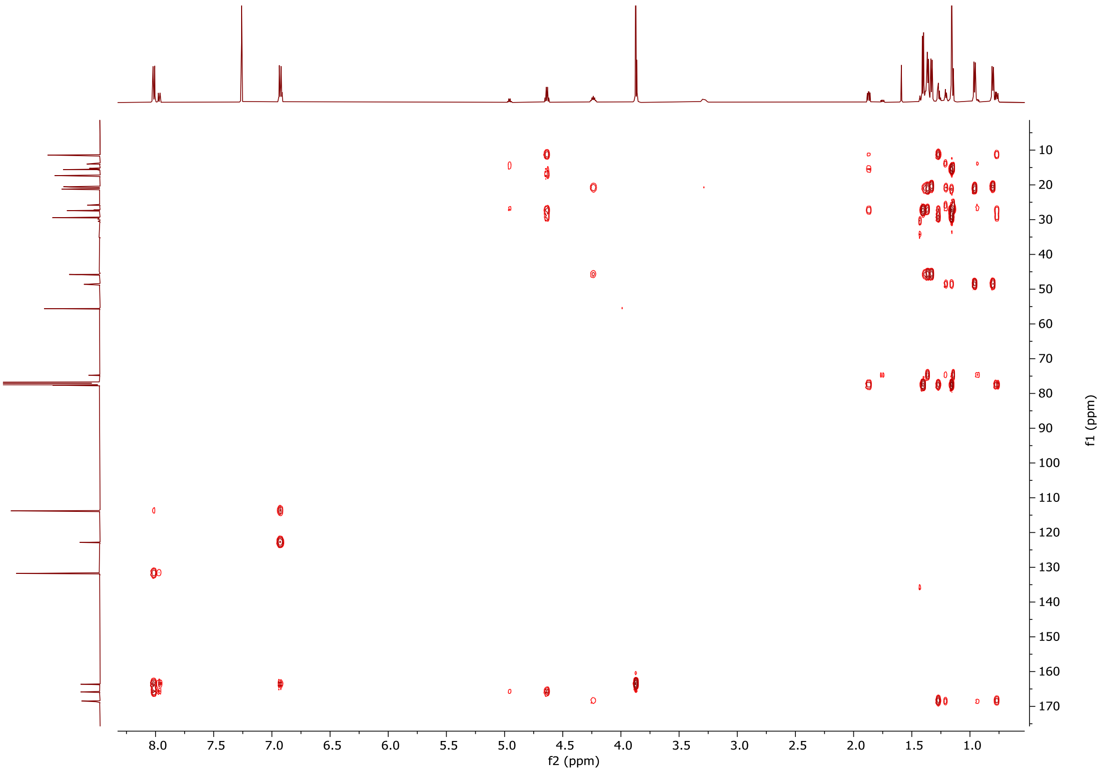

^{1}H **NOSEY** (600 MHz, Chloroform-*d*)

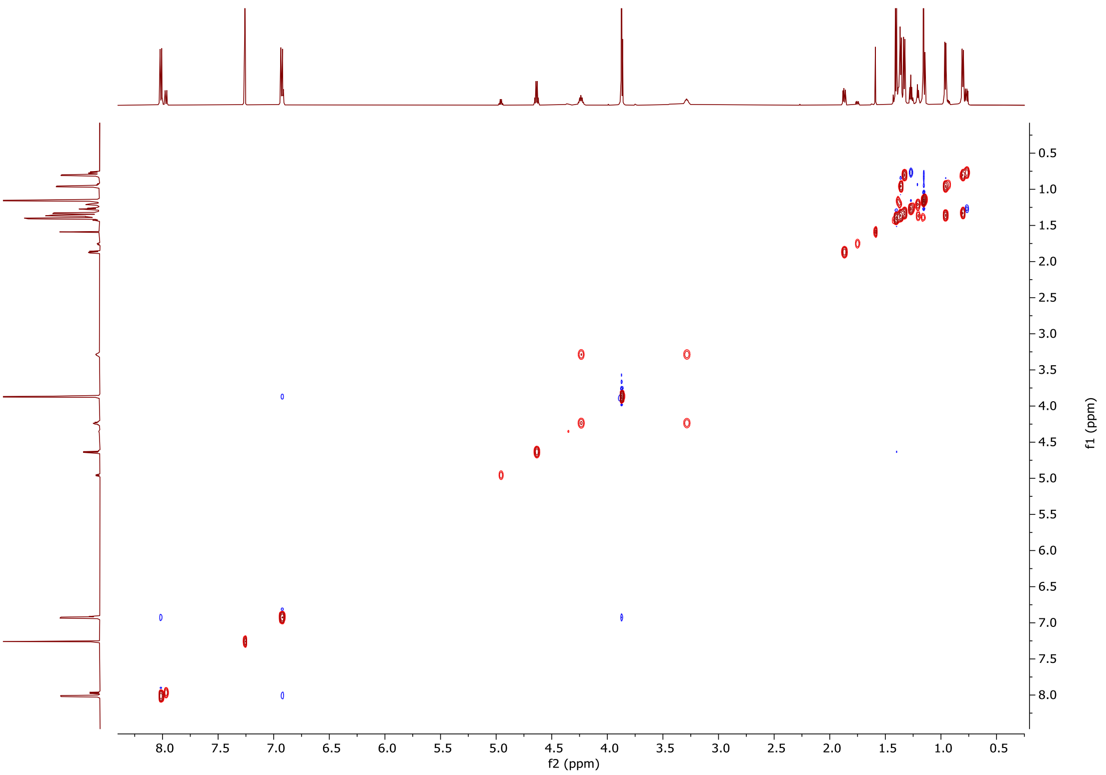

(*R)-1-((1*R**,2*R**)-2-(diisopropylcarbamoyl)-1-methylcyclopropyl)ethyl 1-(2,2-difluorobenzo[d][1,3]dioxol-5-yl)cyclopropane-1-carboxylate, 3g**

^{1}H NMR (600 MHz, Chloroform-*d*)

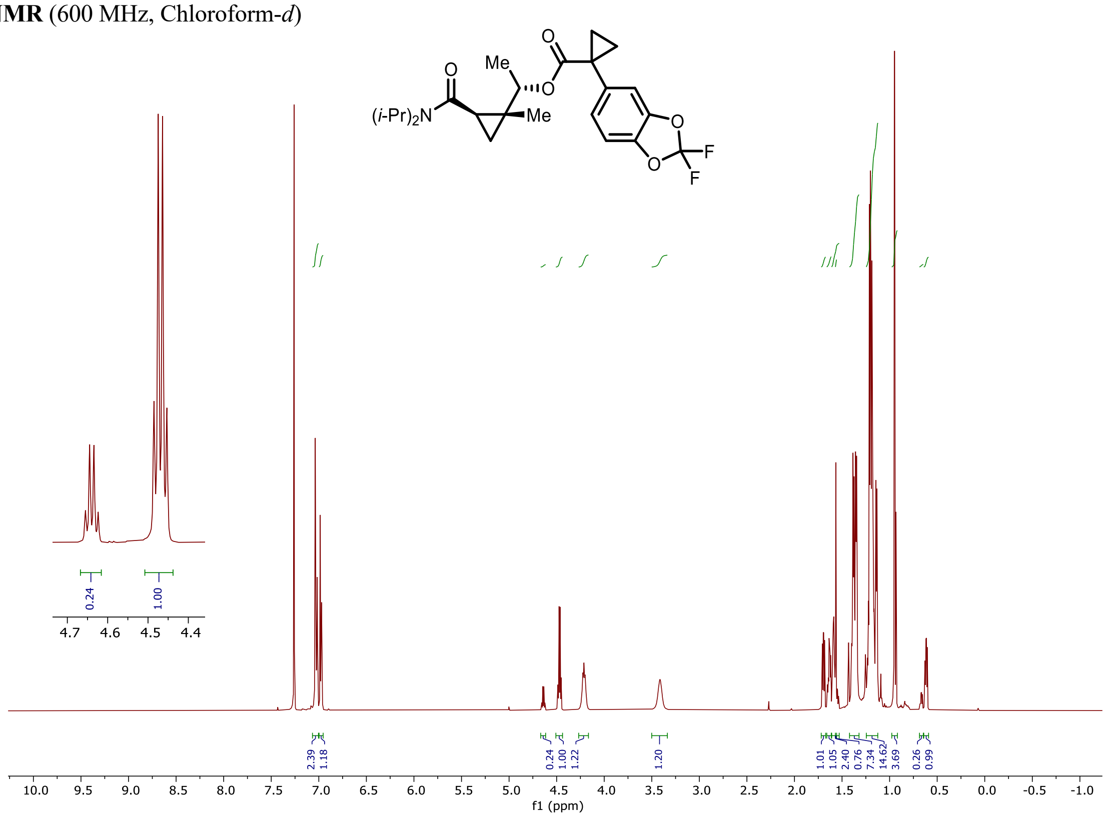

^{13}C NMR (151 MHz, Chloroform-*d*)

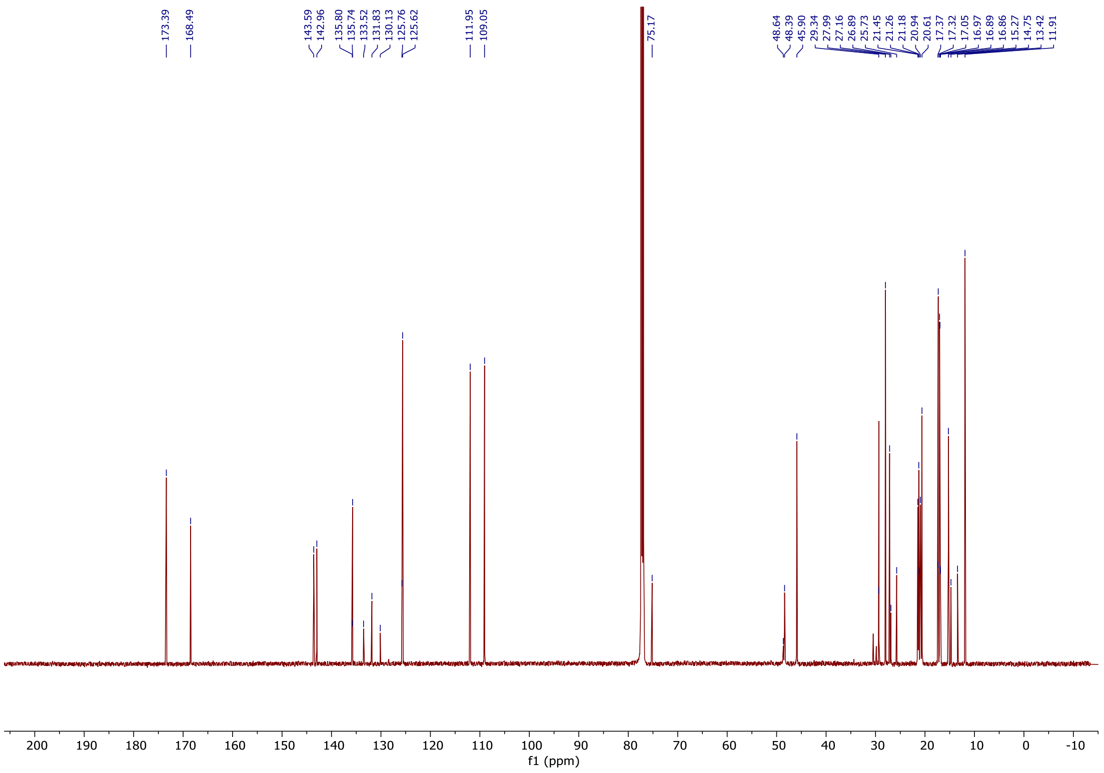

^{1}H COSY (600 MHz, Chloroform-*d*)

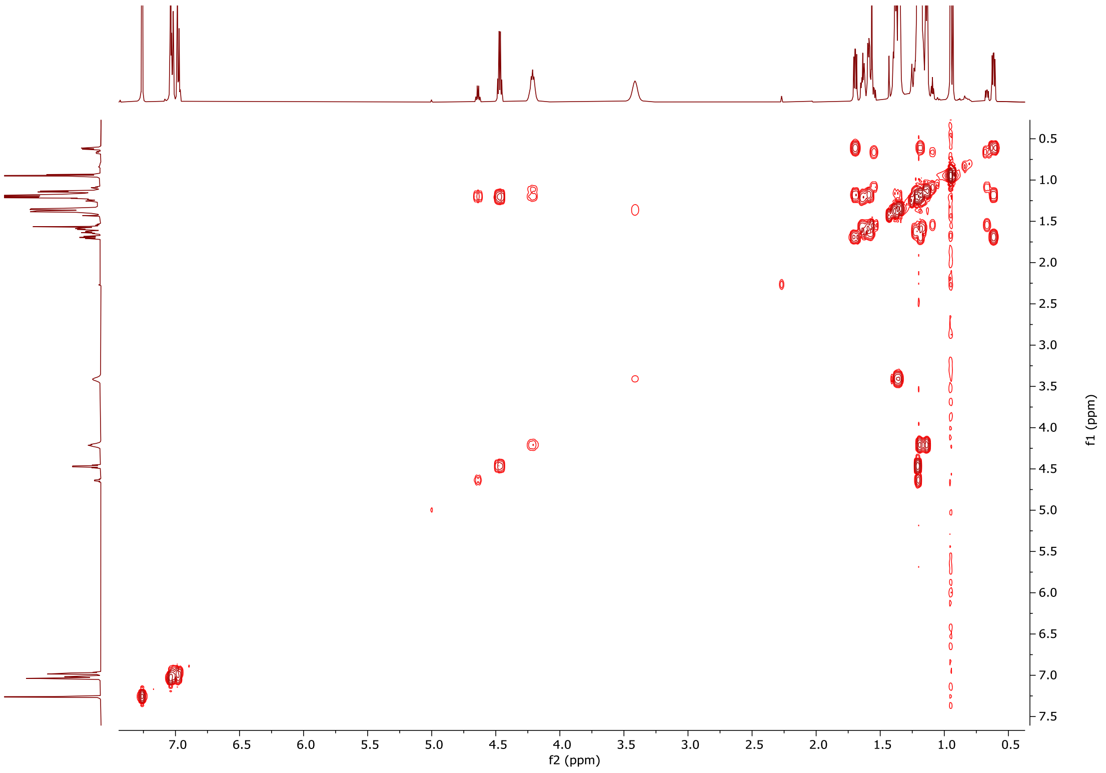

^{1}H/^{13}C HSQC (600/151 MHz, Chloroform-*d*)

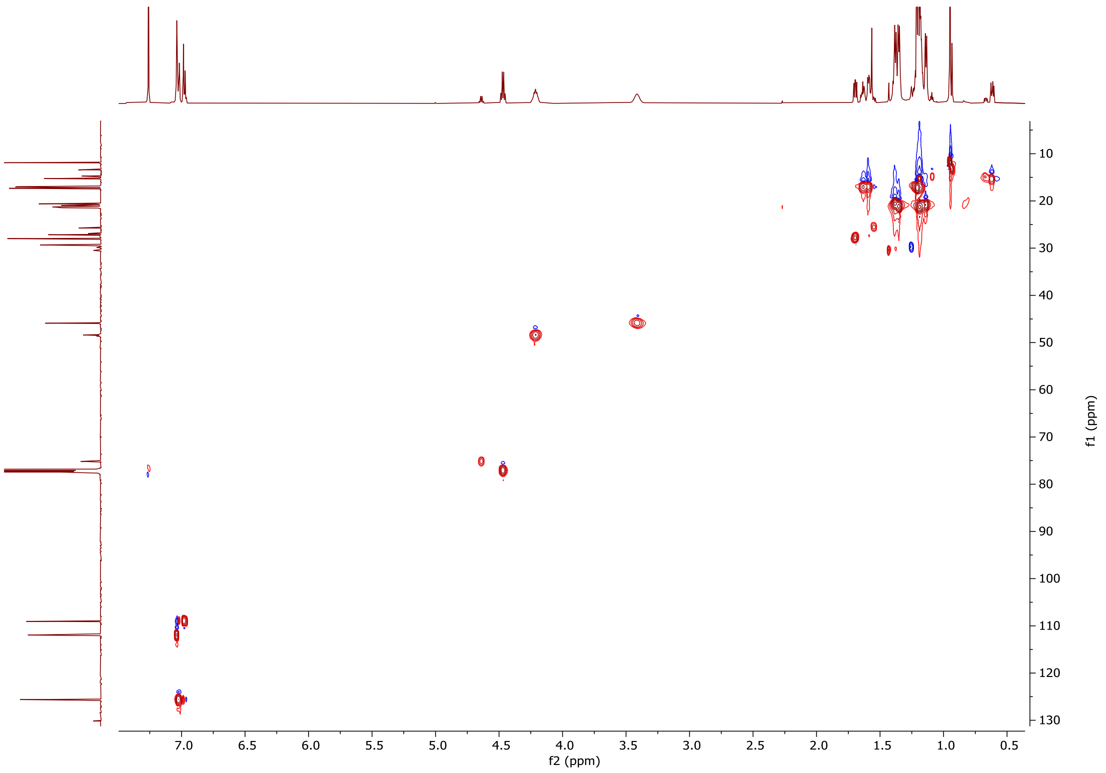

^{1}H/^{13}C HMBC (600/151 MHz, Chloroform-*d*)

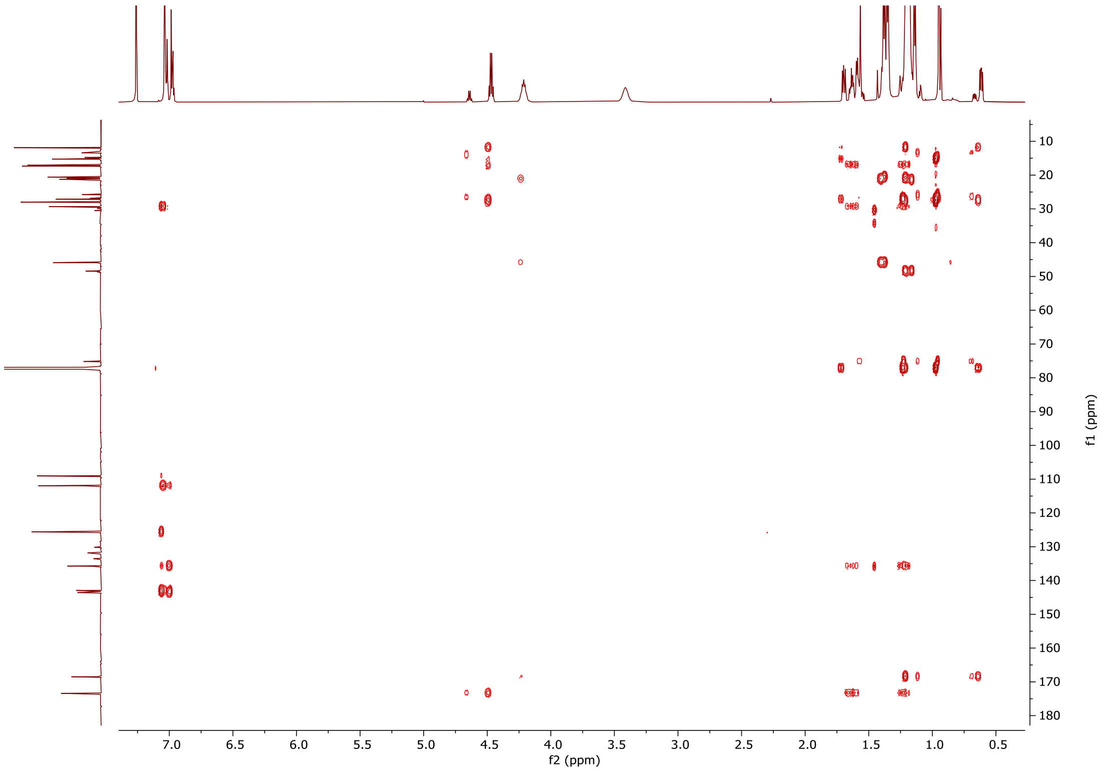

^{1}H NOSEY (600 MHz, Chloroform-*d*)

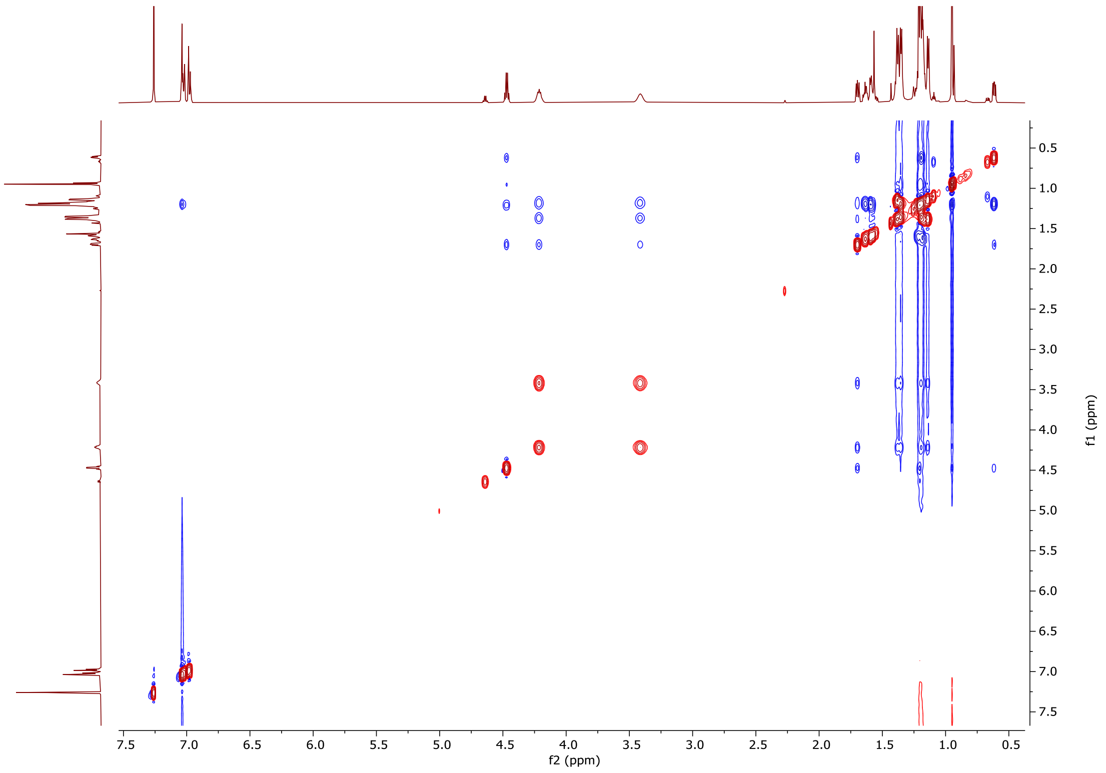

(*R)-1-((1*R**,2*R**)-2-(diisopropylcarbamoyl)-1-methylcyclopropyl)ethyl (2,2,2-trifluoroacetyl)glycinate, 3h**

^{1}**H NMR** (600 MHz, Chloroform-*d*)

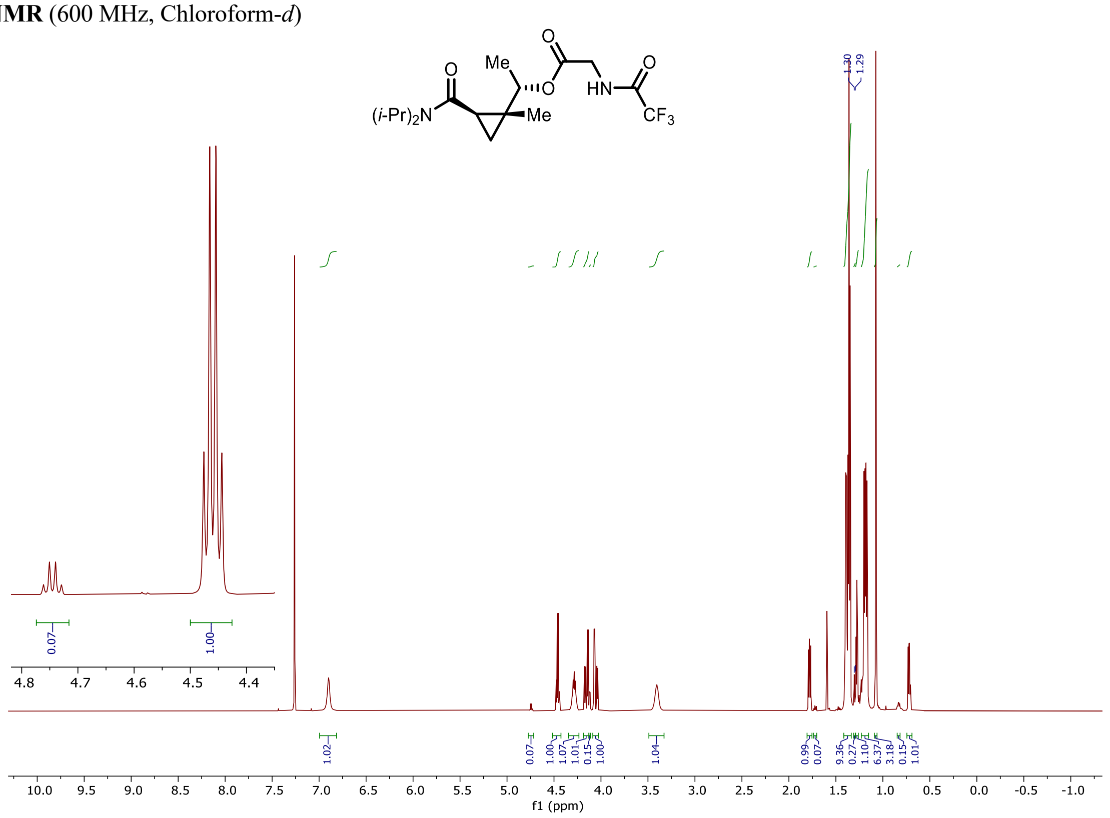

^{13}**C NMR** (151 MHz, Chloroform-*d*)

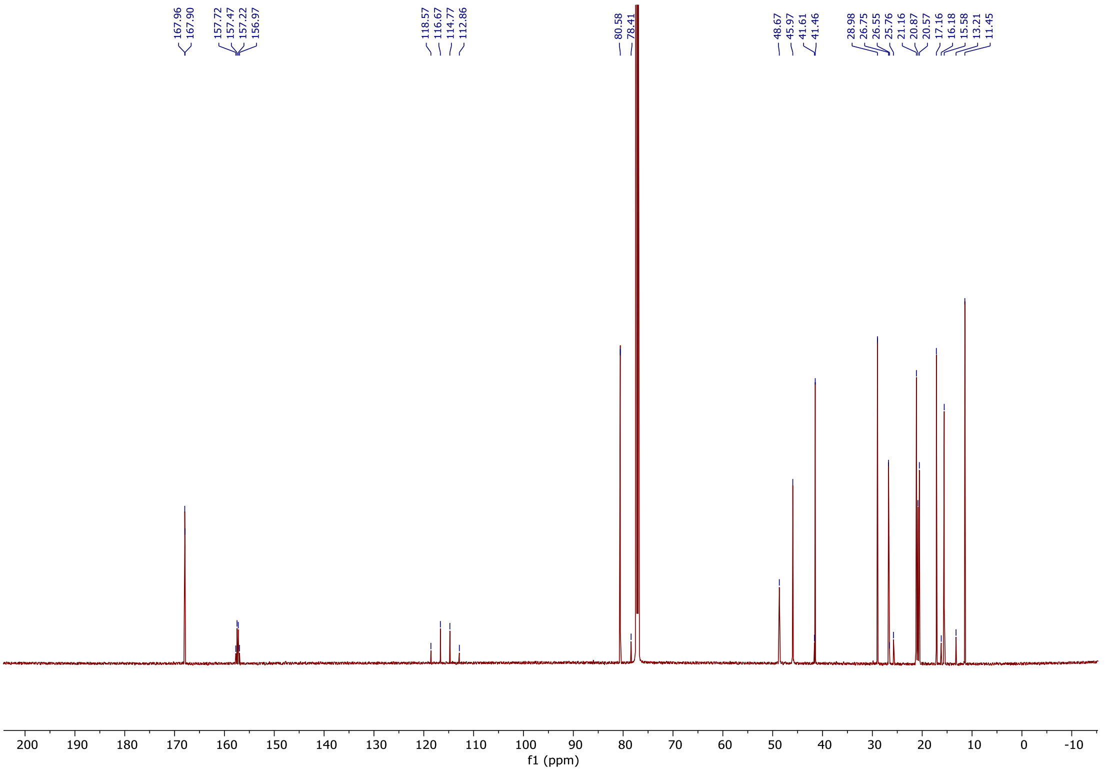

^{1}H COSY (600 MHz, Chloroform-*d*)

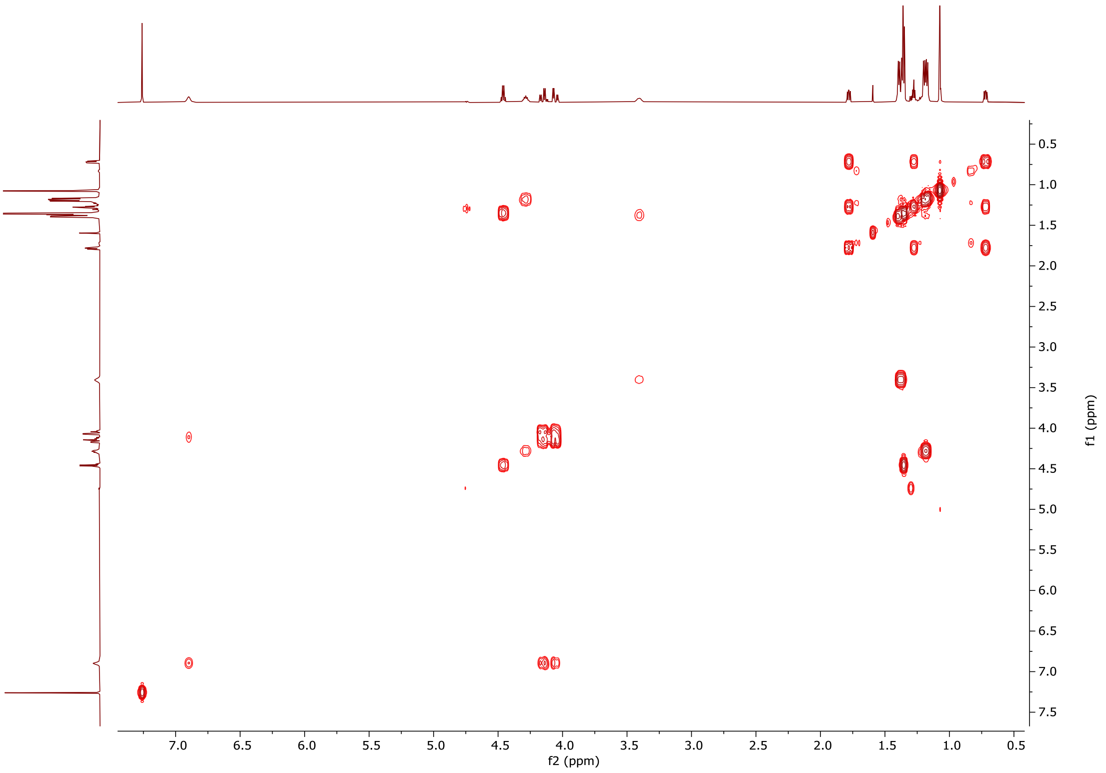

^{1}H/^{13}C HSQC (600/151 MHz, Chloroform-*d*)

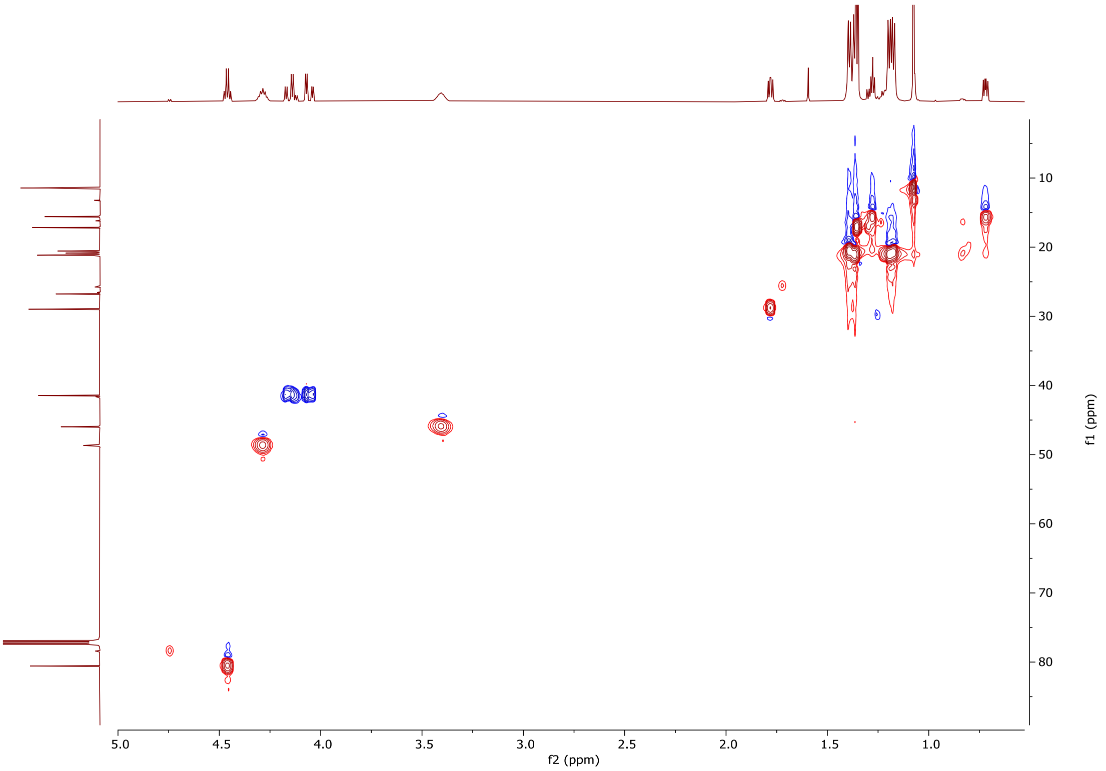

$^{1}H/^{13}C$ HMBC (600/151 MHz, Chloroform-*d*)

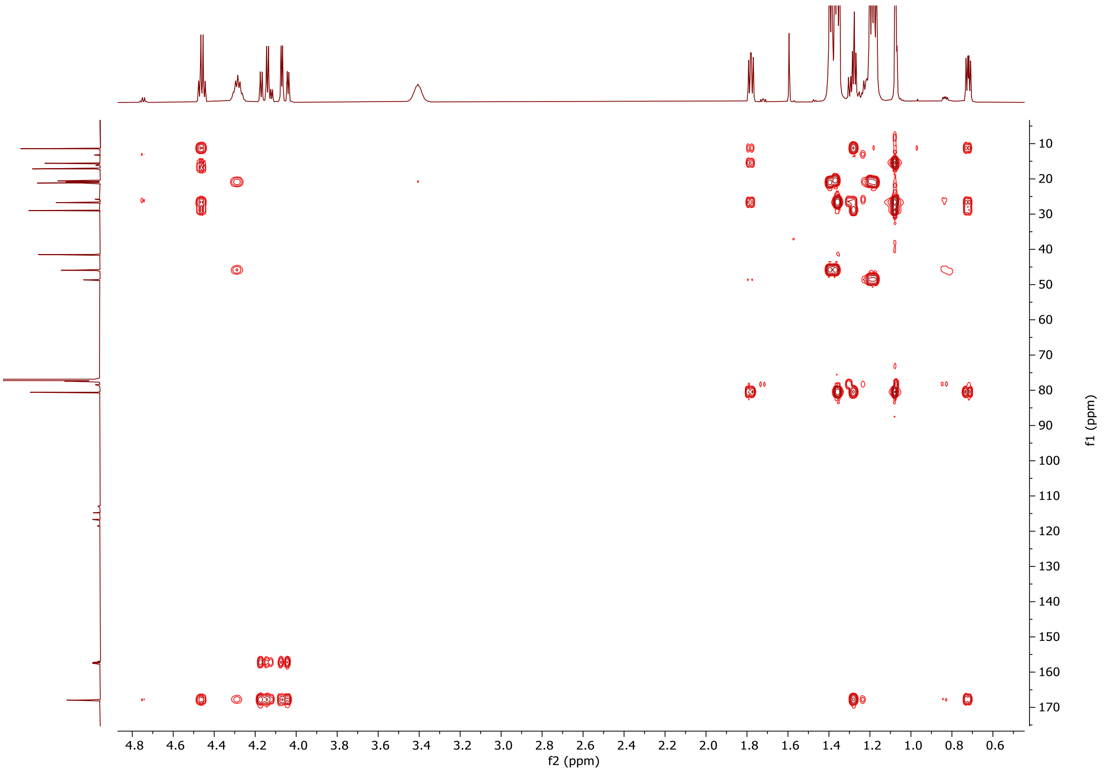

^{1}H NOSEY (600 MHz, Chloroform-*d*)

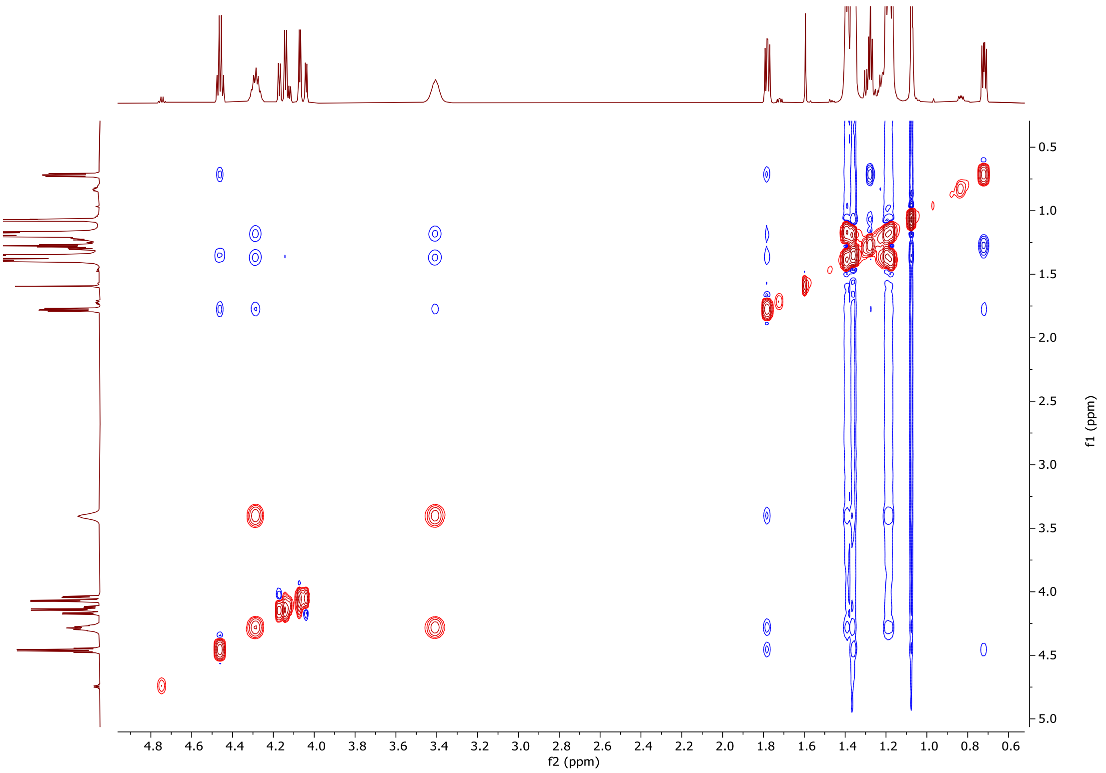

^{19}F NMR (565 MHz, Chloroform-*d*)

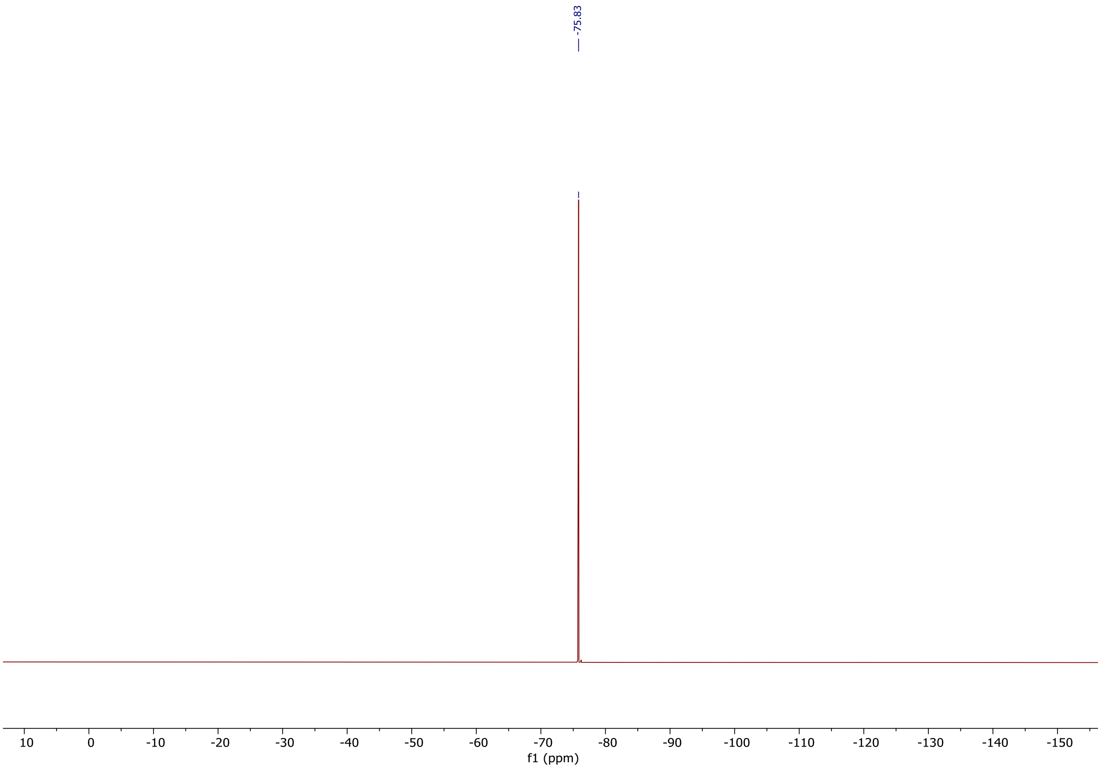

1-((*S)-1-((1*R**,2*R**)-2-(diisopropylcarbamoyl)-1-methylcyclopropyl)ethyl) 3-methyl bicyclo[1.1.1]pentane-1,3-dicarboxylate, 3i**

^{1}H NMR (600 MHz, Chloroform-*d*)

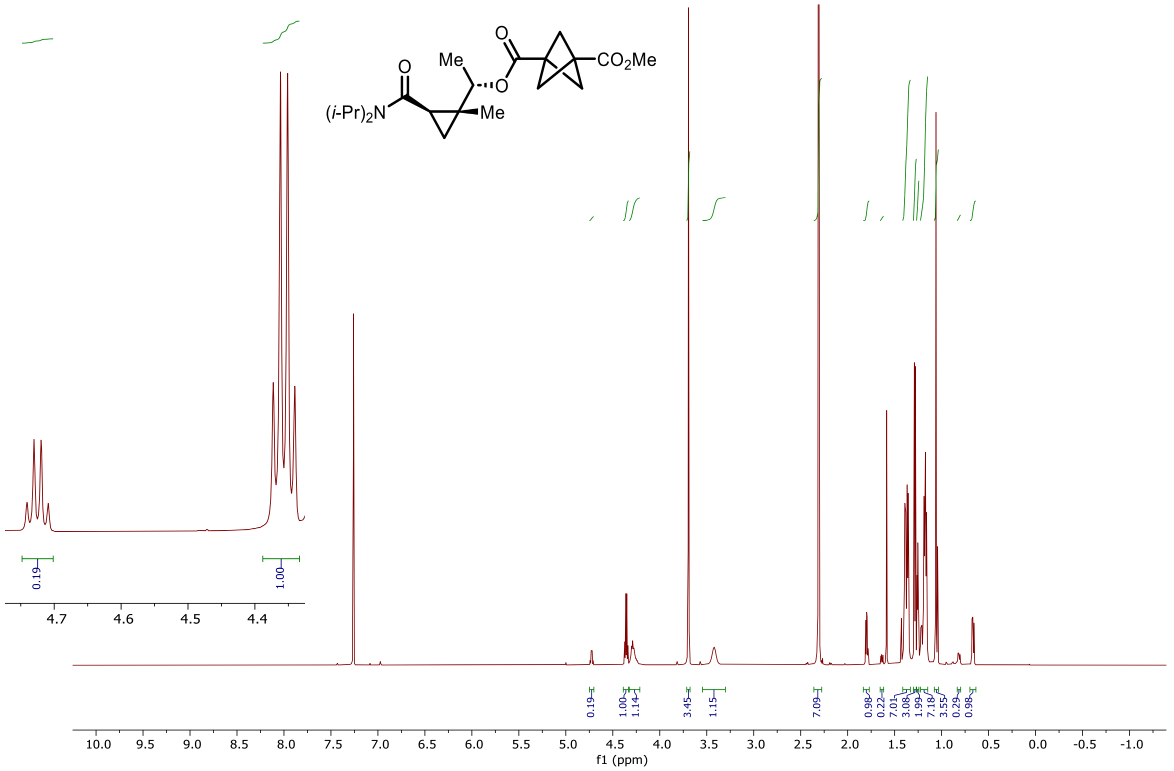

^{13}C NMR (151 MHz, Chloroform-*d*)

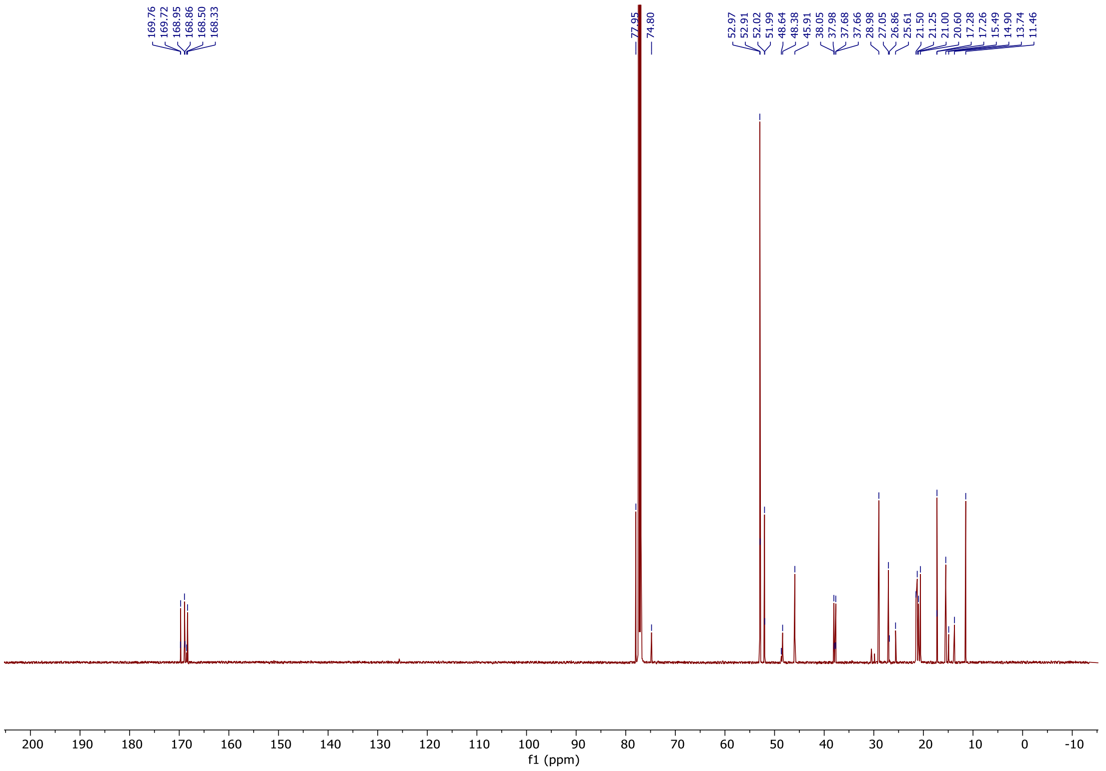

^{1}H COSY (600 MHz, Chloroform-*d*)

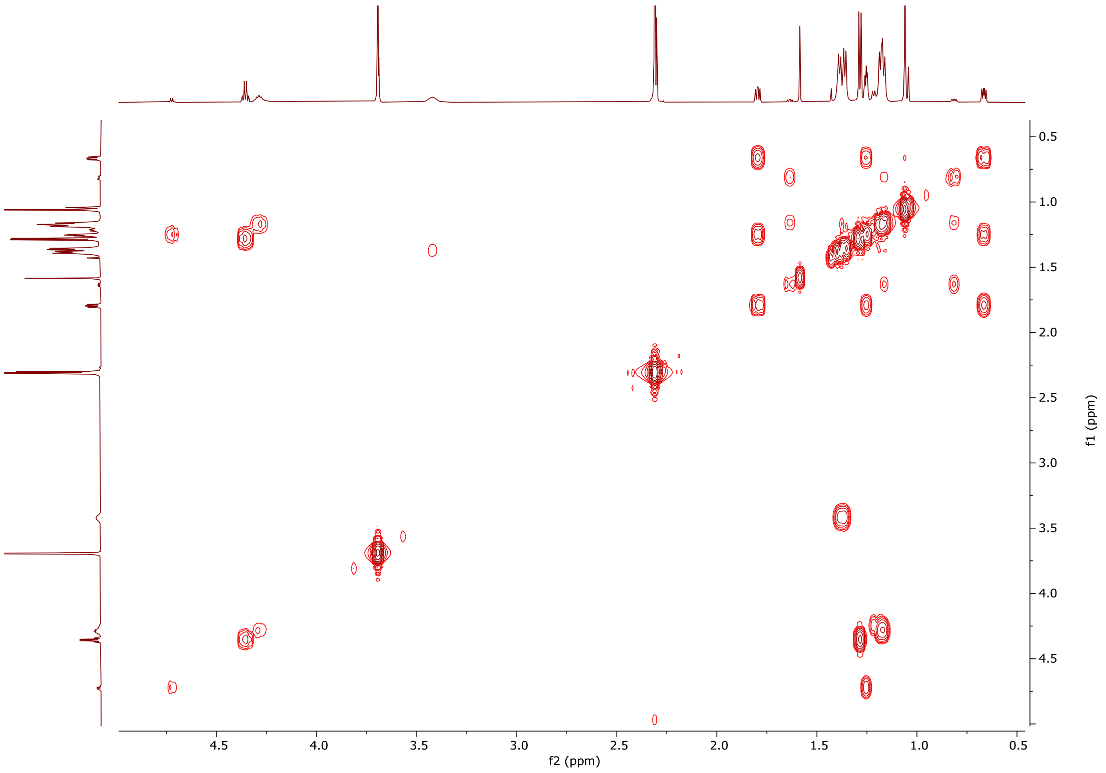

^{1}H/^{13}C HSQC (600/151 MHz, Chloroform-*d*)

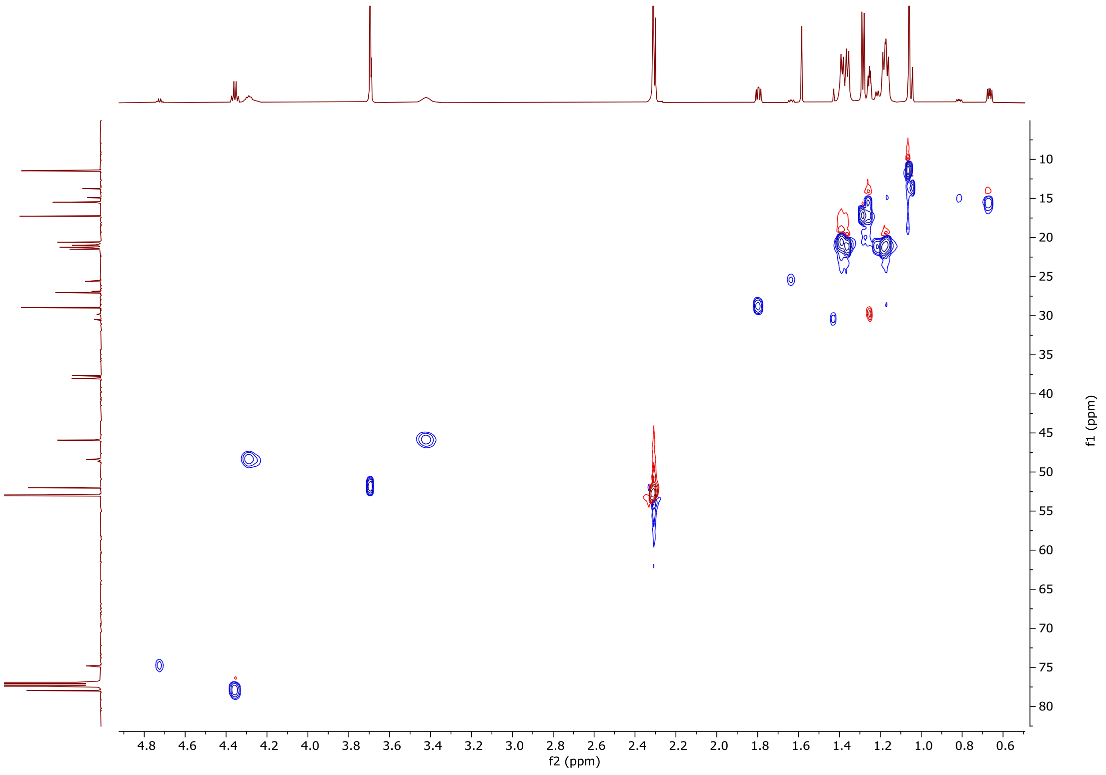

^{1}H/^{13}C HMBC (600/151 MHz, Chloroform-*d*)

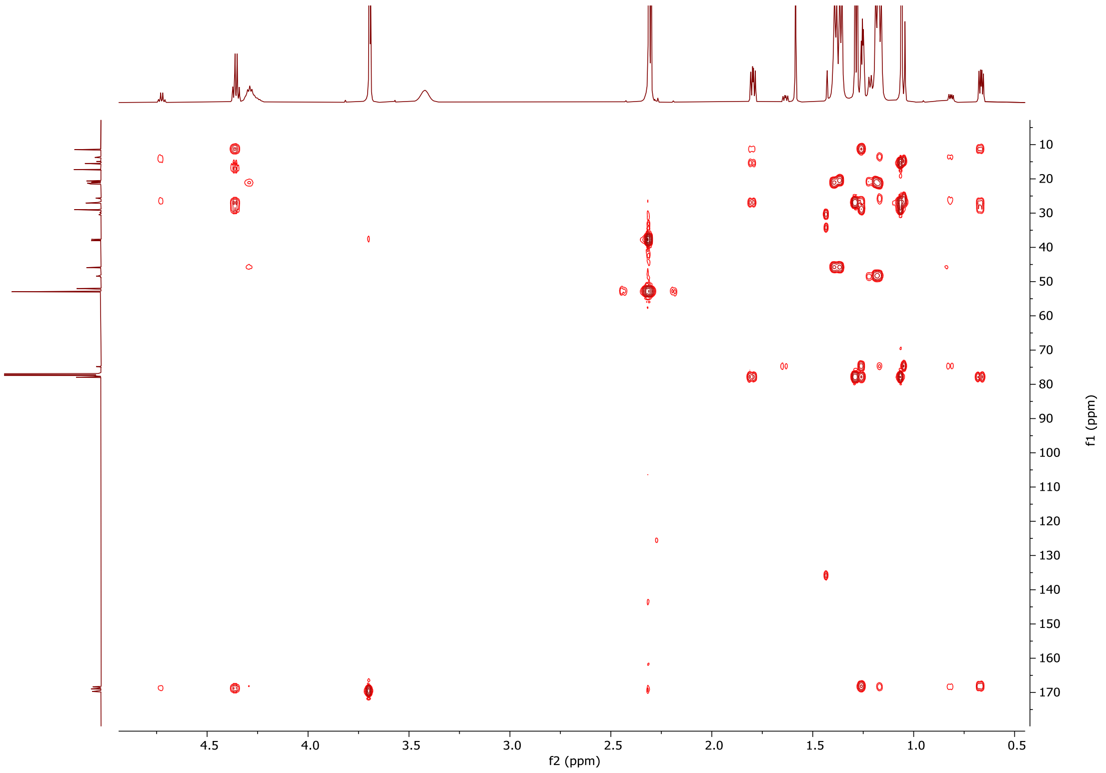

^{1}H NOSEY (600 MHz, Chloroform-*d*)

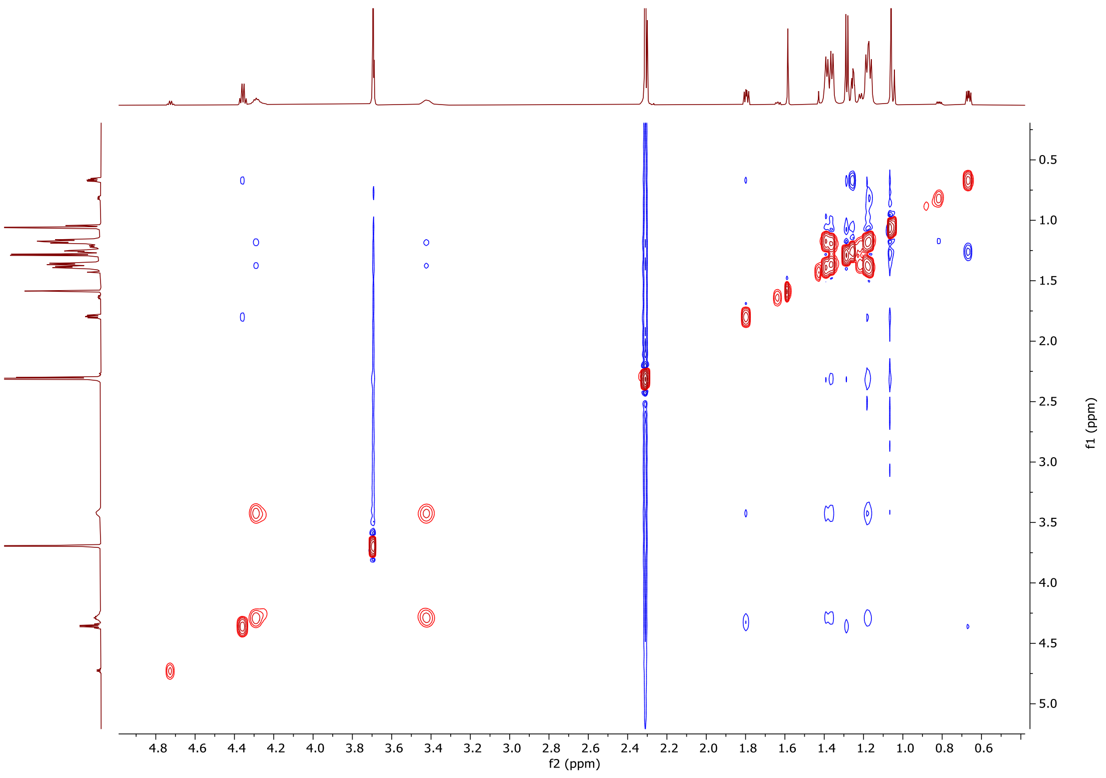

(*R)-1-((1*R**,2*R**)-2-(diisopropylcarbamoyl)-1-methylcyclopropyl)ethyl 2,4,6-triisopropylbenzoate, 3j**

^{1}H NMR (600 MHz, Chloroform-*d*)

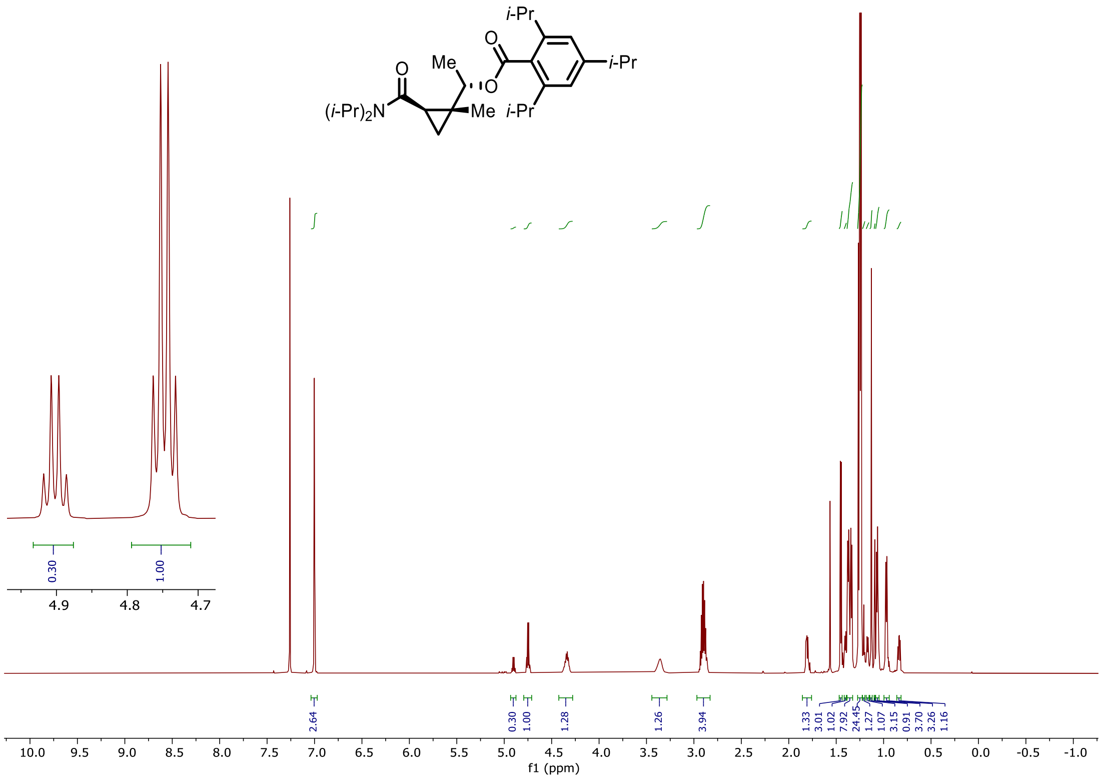

^{13}C NMR (151 MHz, Chloroform-*d*)

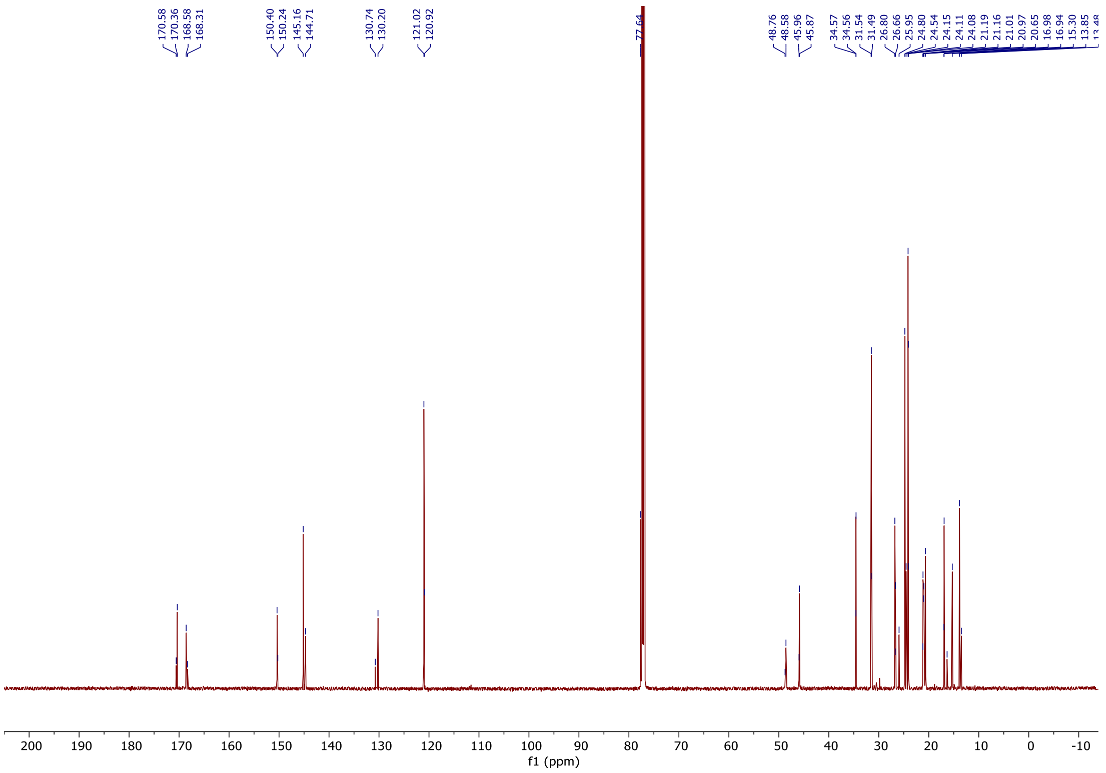

^{1}H COSY (600 MHz, Chloroform-*d*)

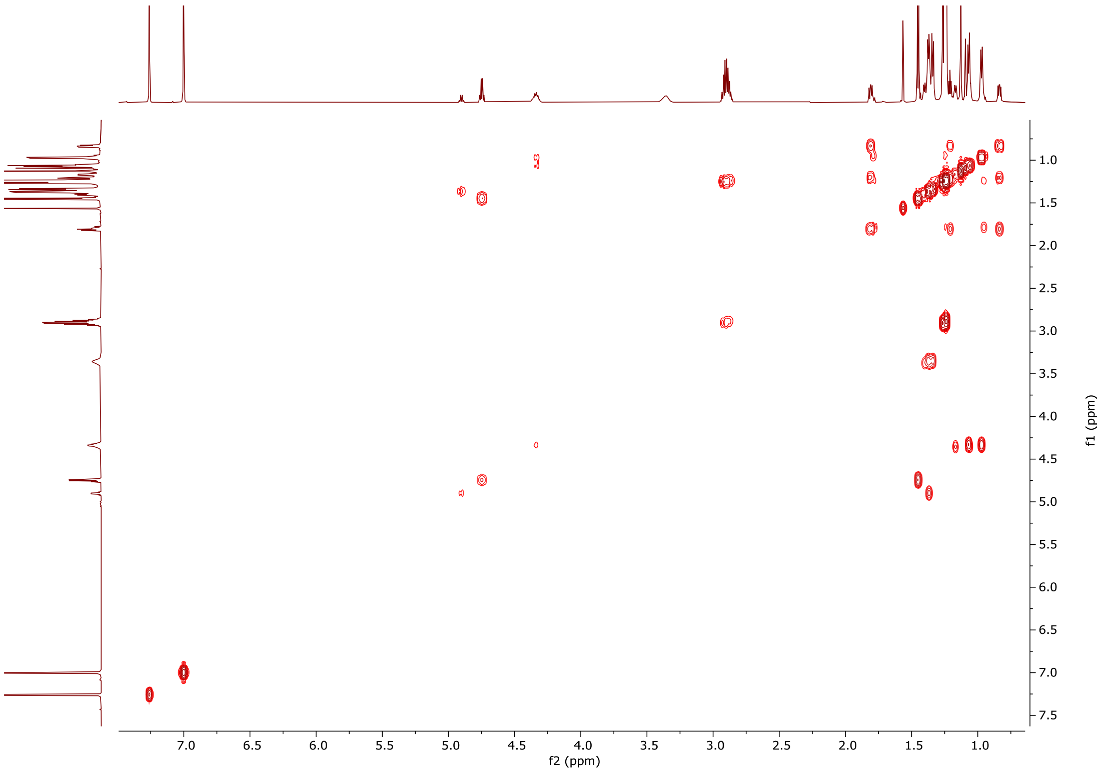

^{1}H/^{13}C HSQC (600/151 MHz, Chloroform-*d*)

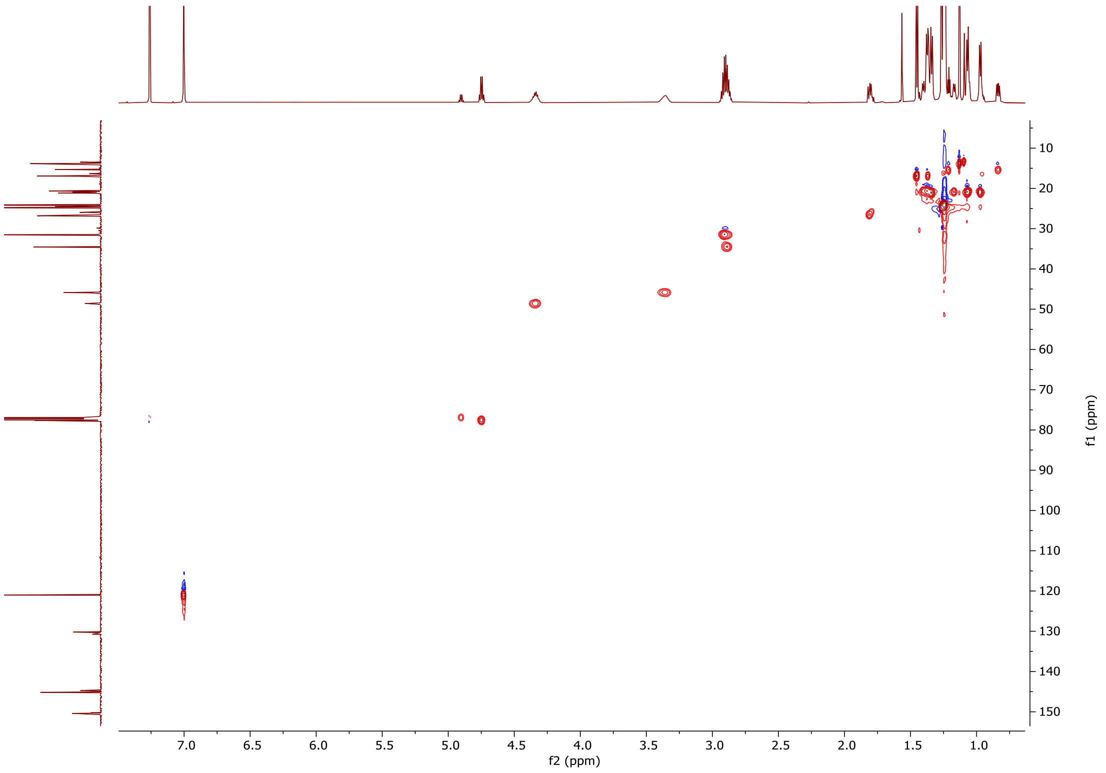

$^{1}H/^{13}C$ HMBC (600/151 MHz, Chloroform-*d*)

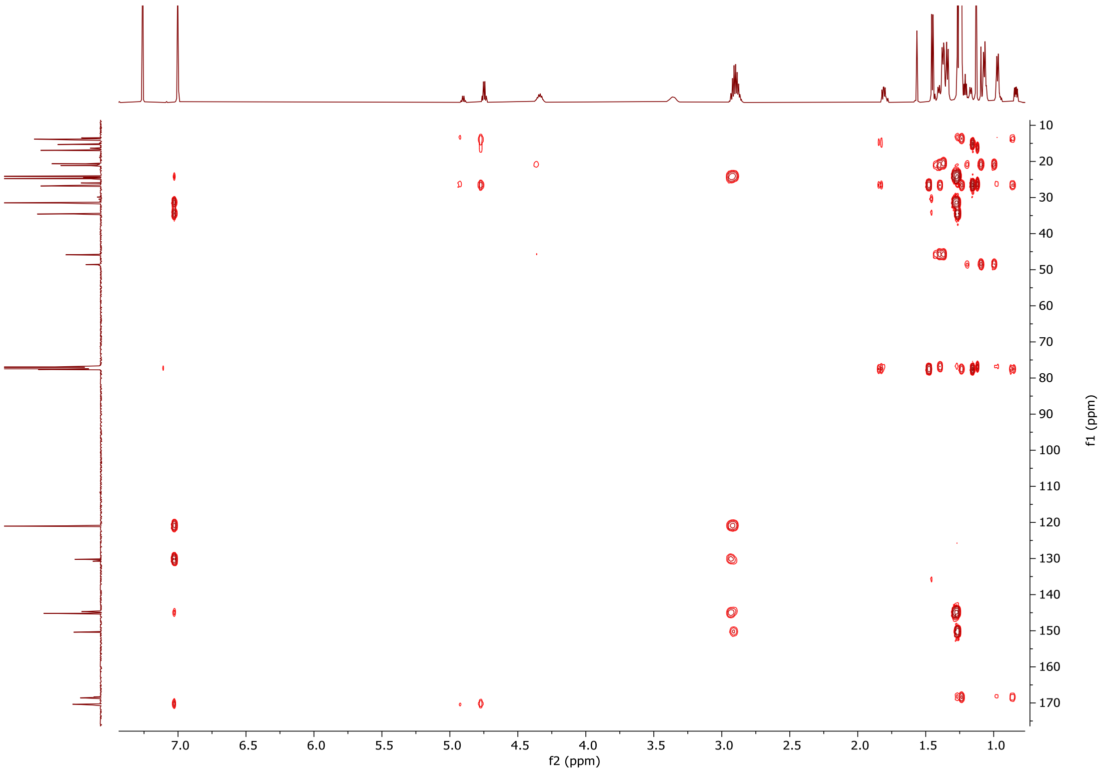

^{1}H NOSEY (600 MHz, Chloroform-*d*)

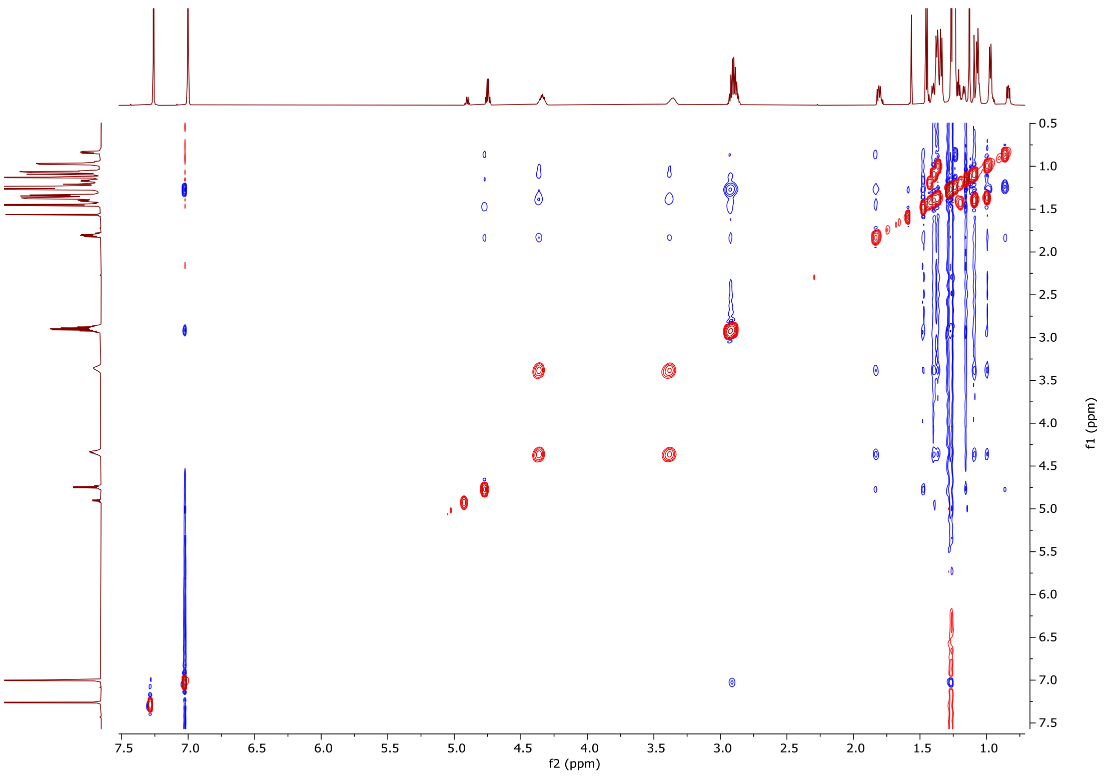

(*R)-1-((1*R**,2*R**)-2-(diisopropylcarbamoyl)-1-methylcyclopropyl)ethyl 2-(1-(4-chlorobenzoyl)-5-methoxy-1H-indol-3-yl)acetate, 3k**

^{1}H NMR (600 MHz, Chloroform-*d*)

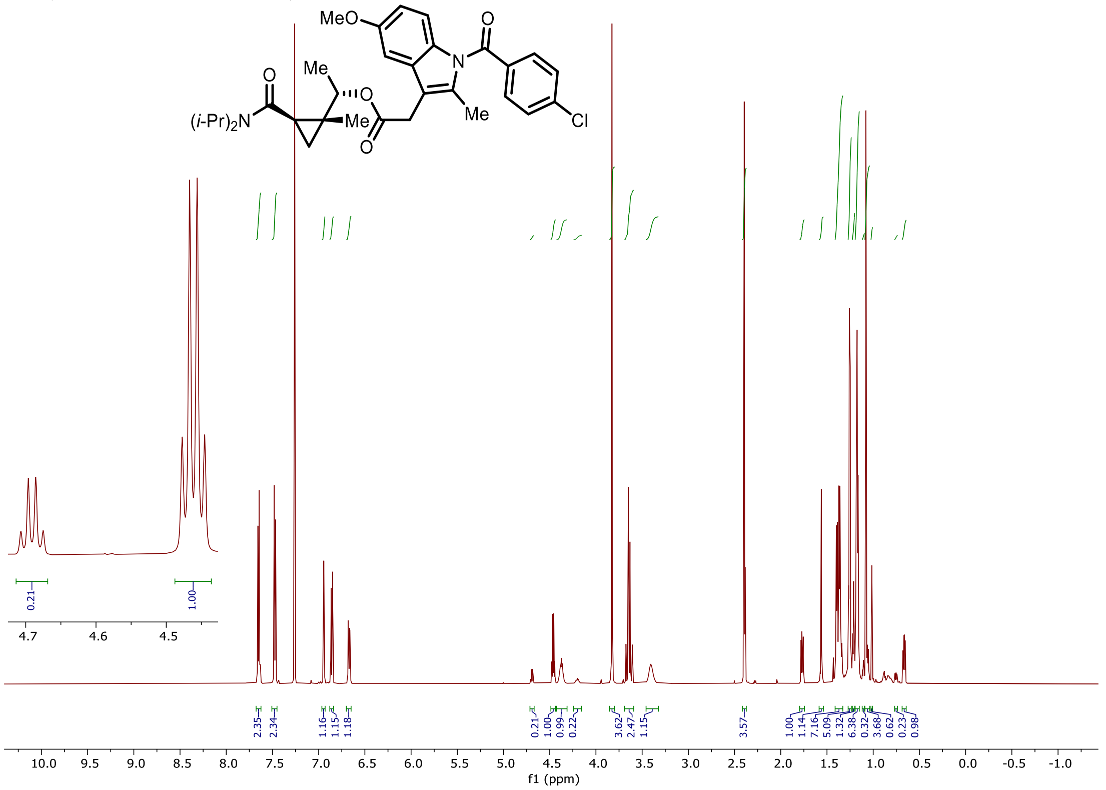

^{13}C NMR (151 MHz, Chloroform-*d*)

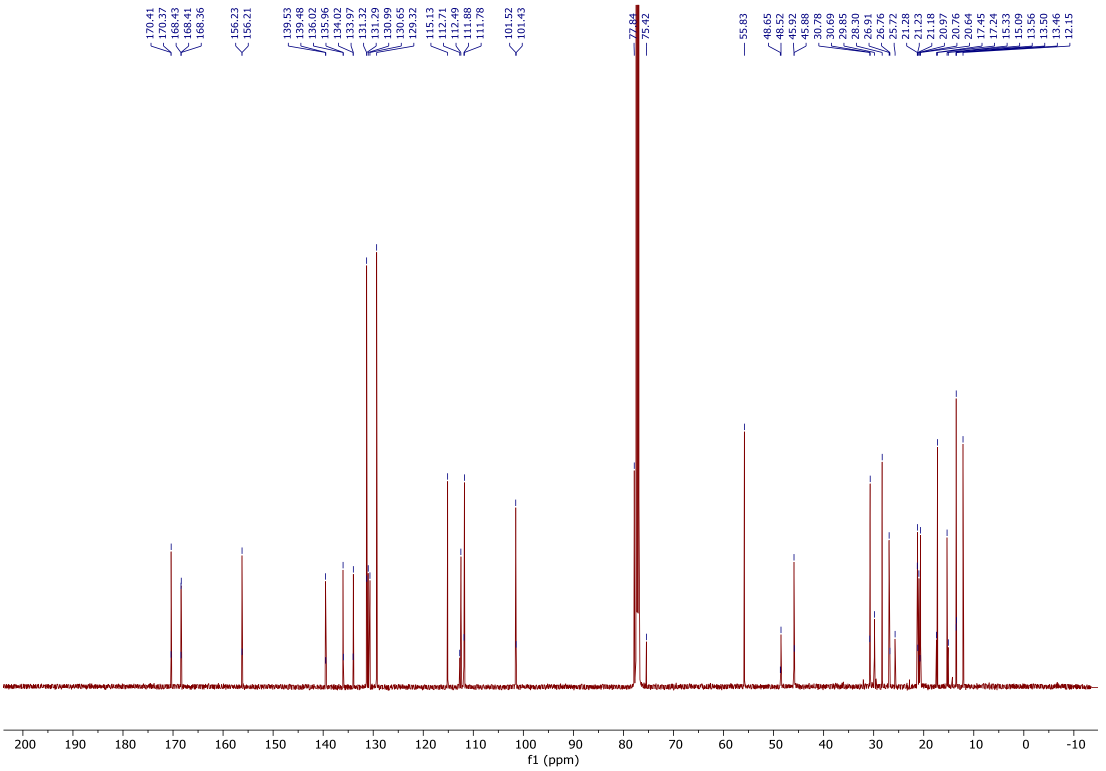

^{1}H COSY (600 MHz, Chloroform-*d*)

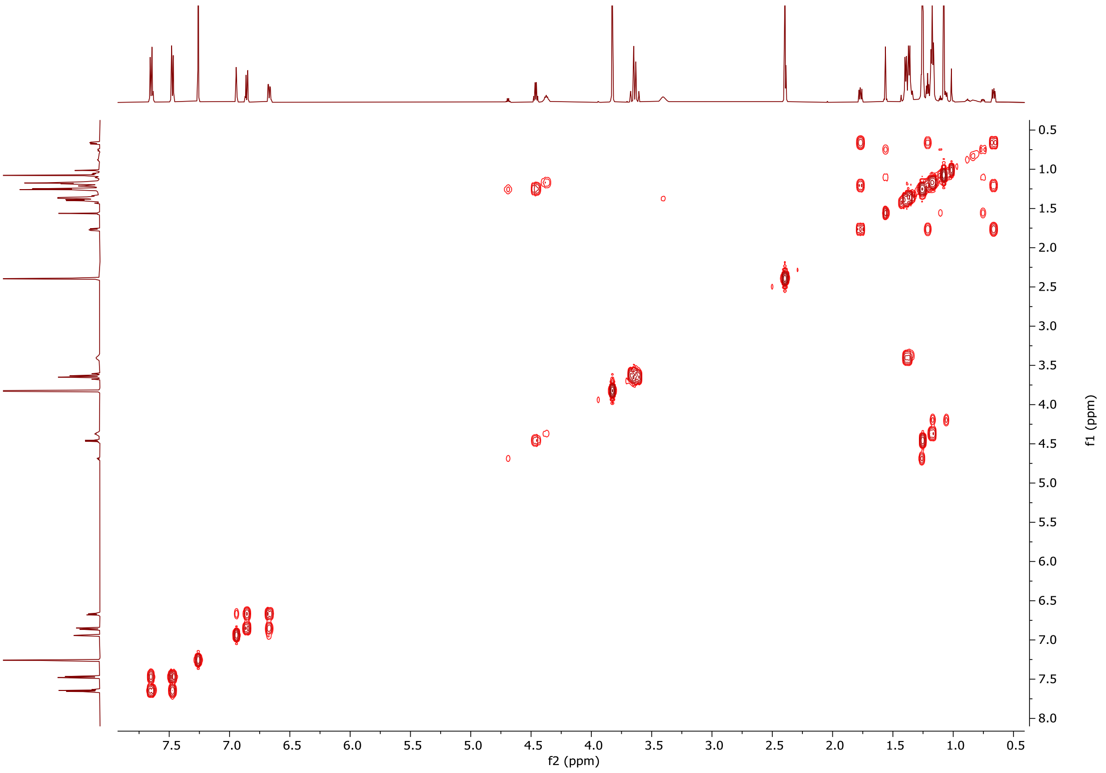

^{1}H/^{13}C HSQC (600/151 MHz, Chloroform-*d*)

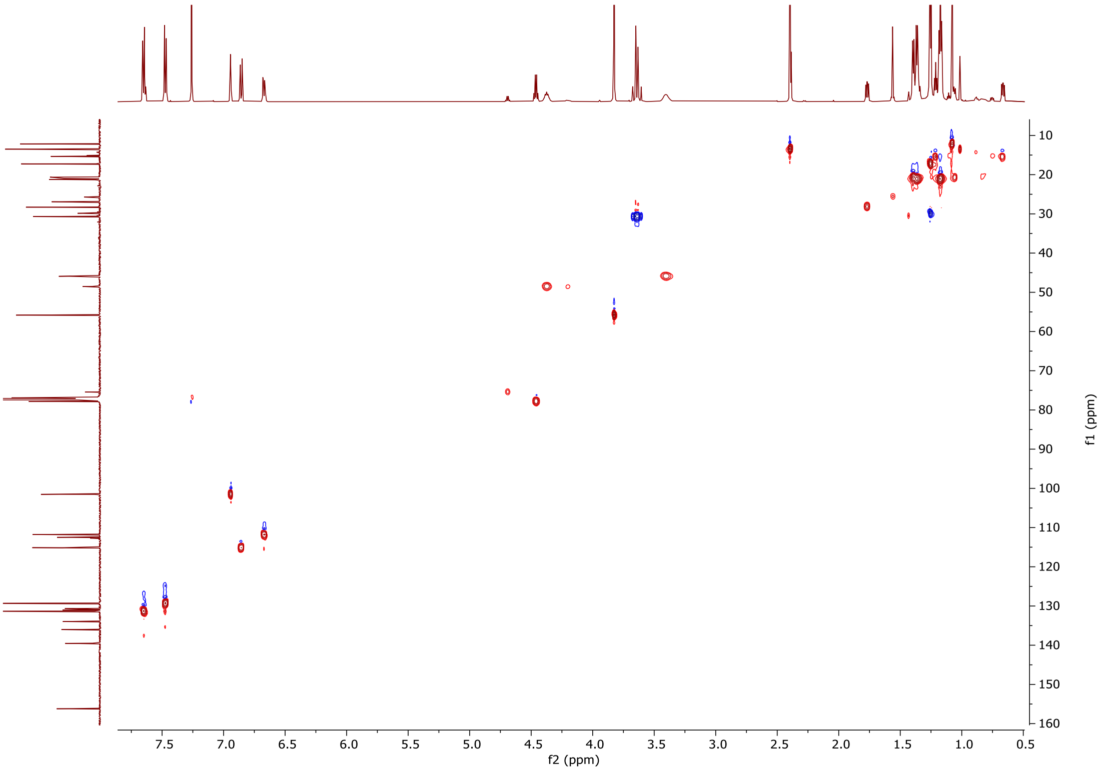

$^{1}H/^{13}C$ **HMBC** (600/151 MHz, Chloroform-*d*)

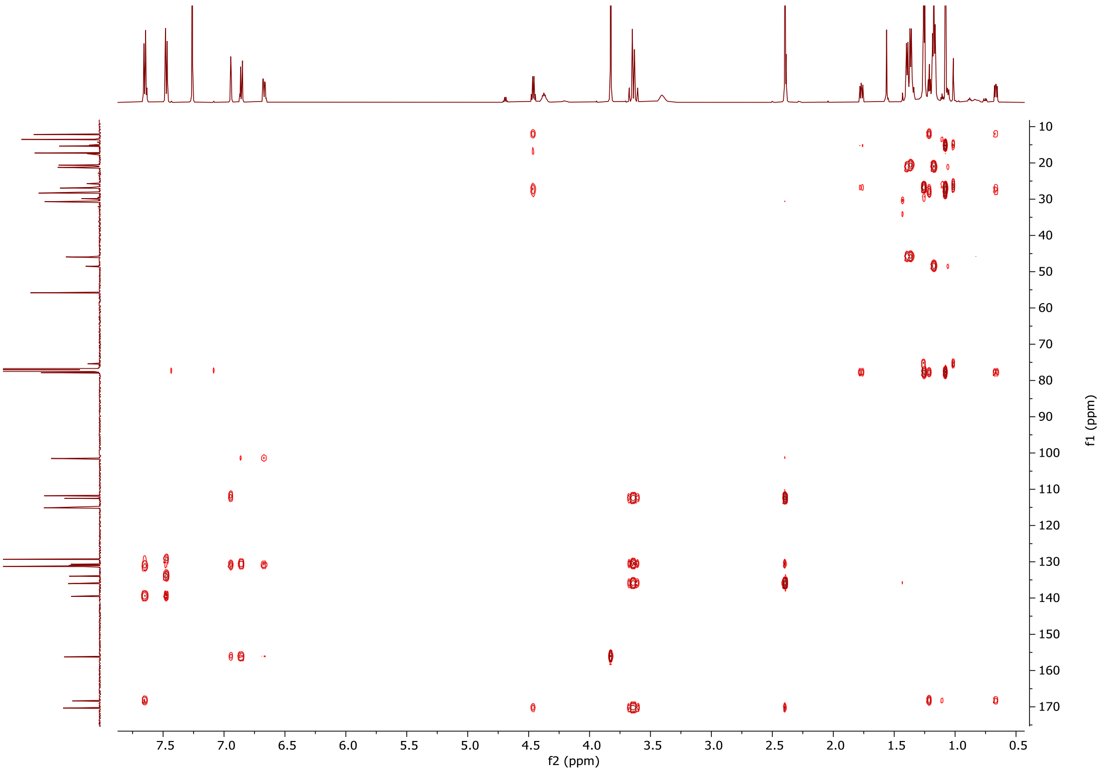

^{1}H **NOSEY** (600 MHz, Chloroform-*d*)

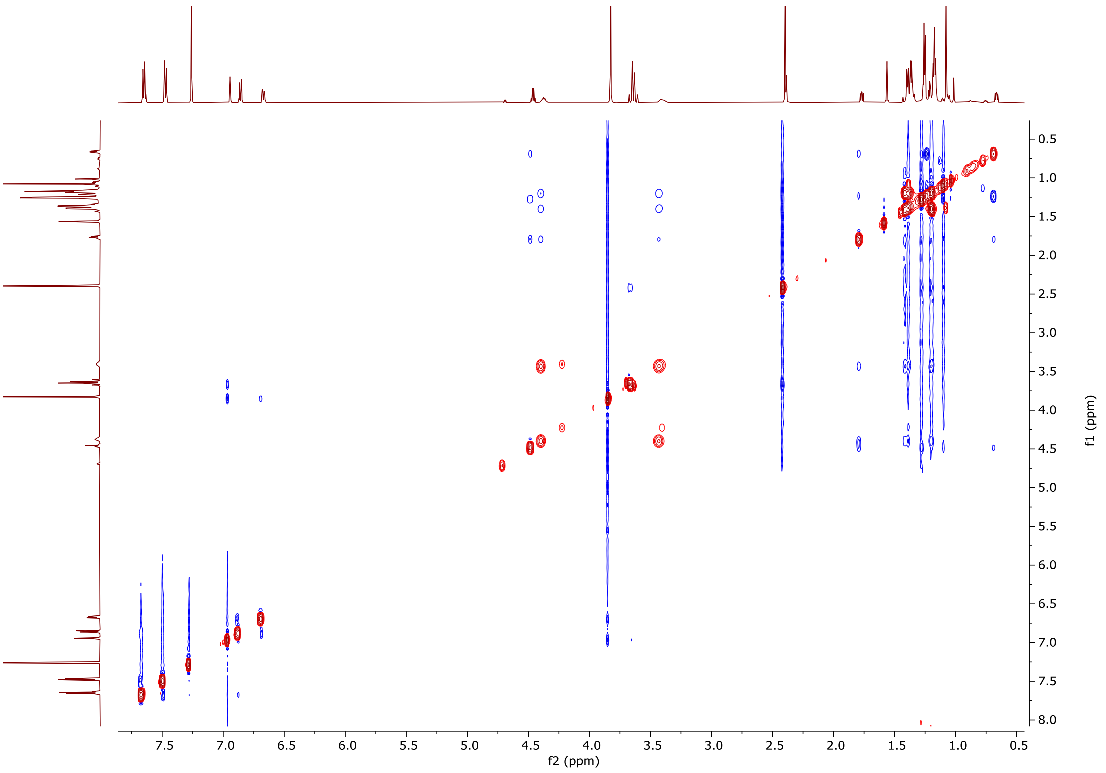

(1*R,2*R**)-2-((*S**)-1-fluoroethyl)-*N*,*N*-diisopropyl-2-methylcyclopropane-1-carboxamide, 3l**

^{1}H NMR (600 MHz, Chloroform-*d*)

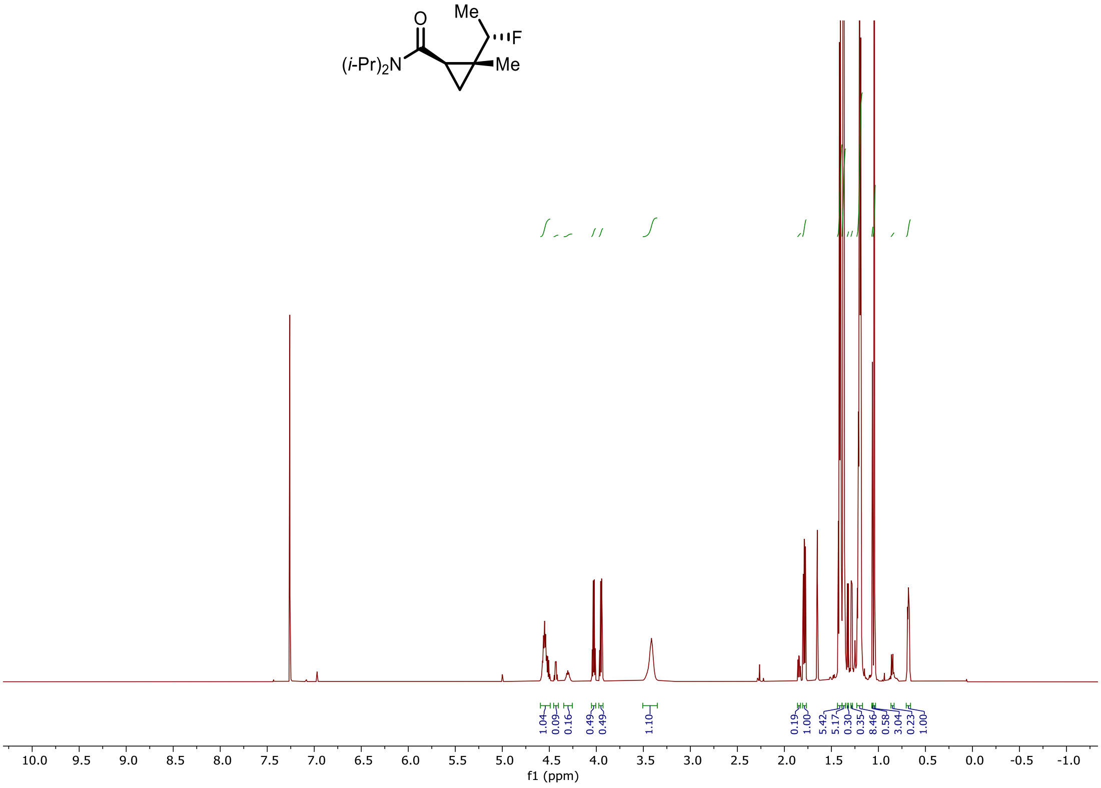

^{13}C NMR (151 MHz, Chloroform-*d*)

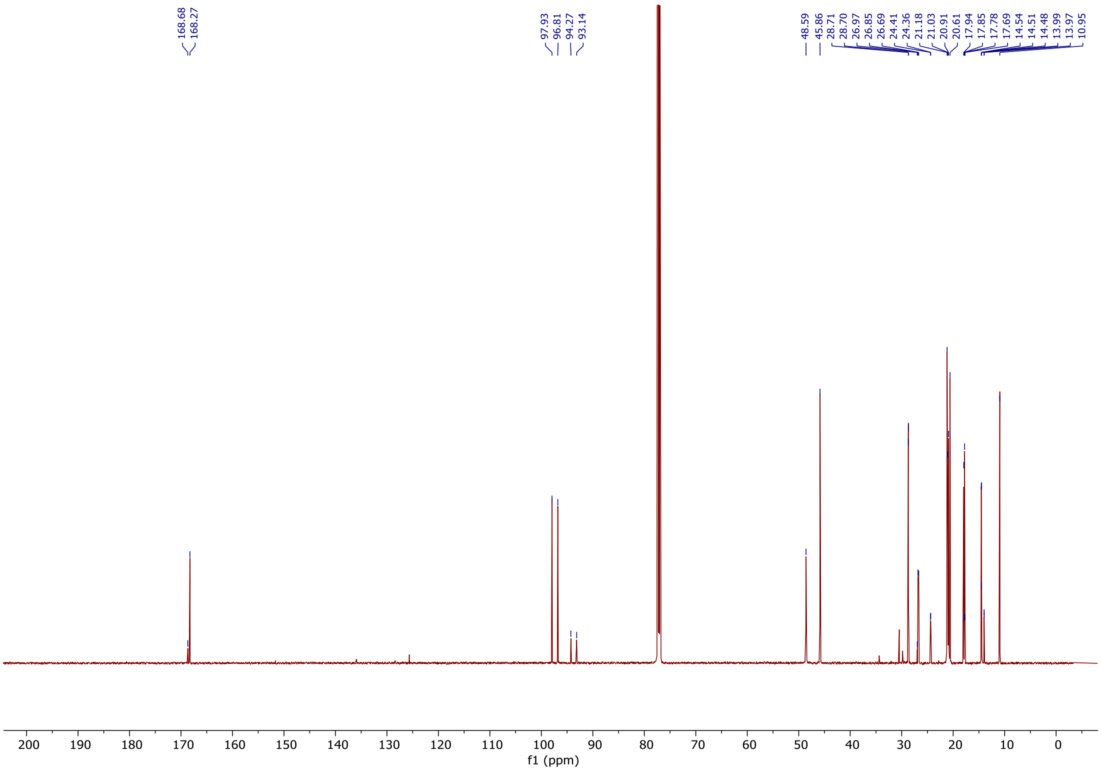

^{1}**H COSY** (600 MHz, Chloroform-*d*)

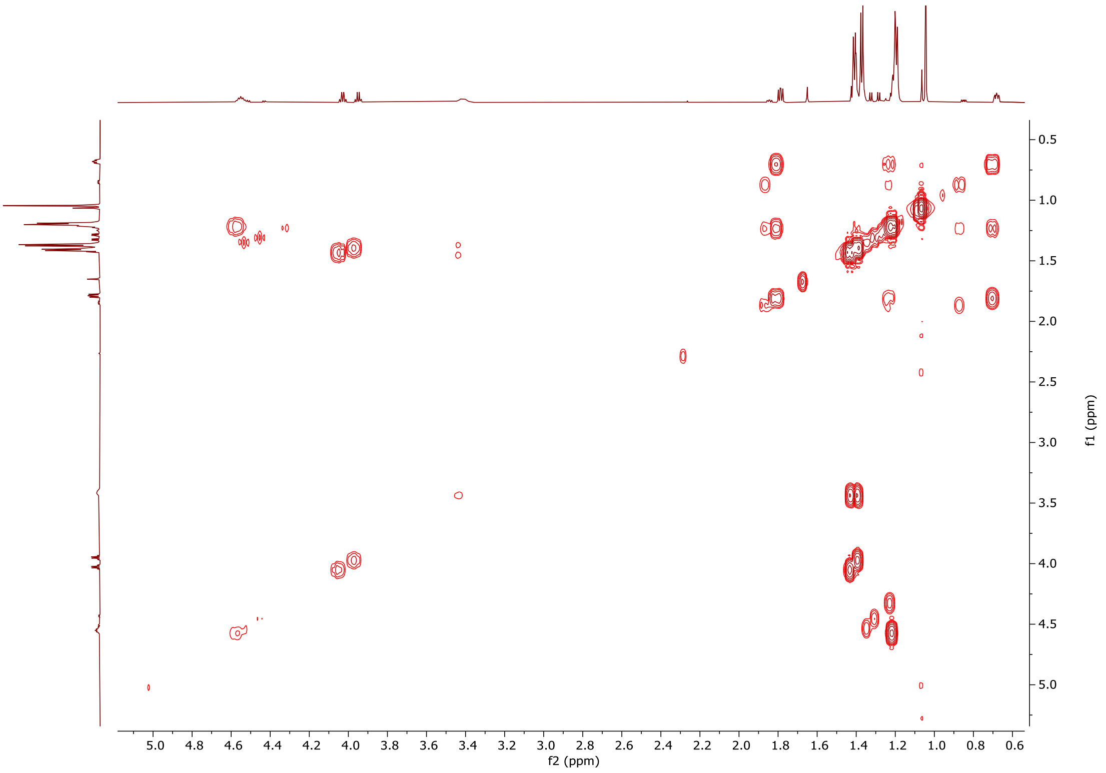

^{1}**H/**^{13}**C HSQC** (600/151 MHz, Chloroform-*d*)

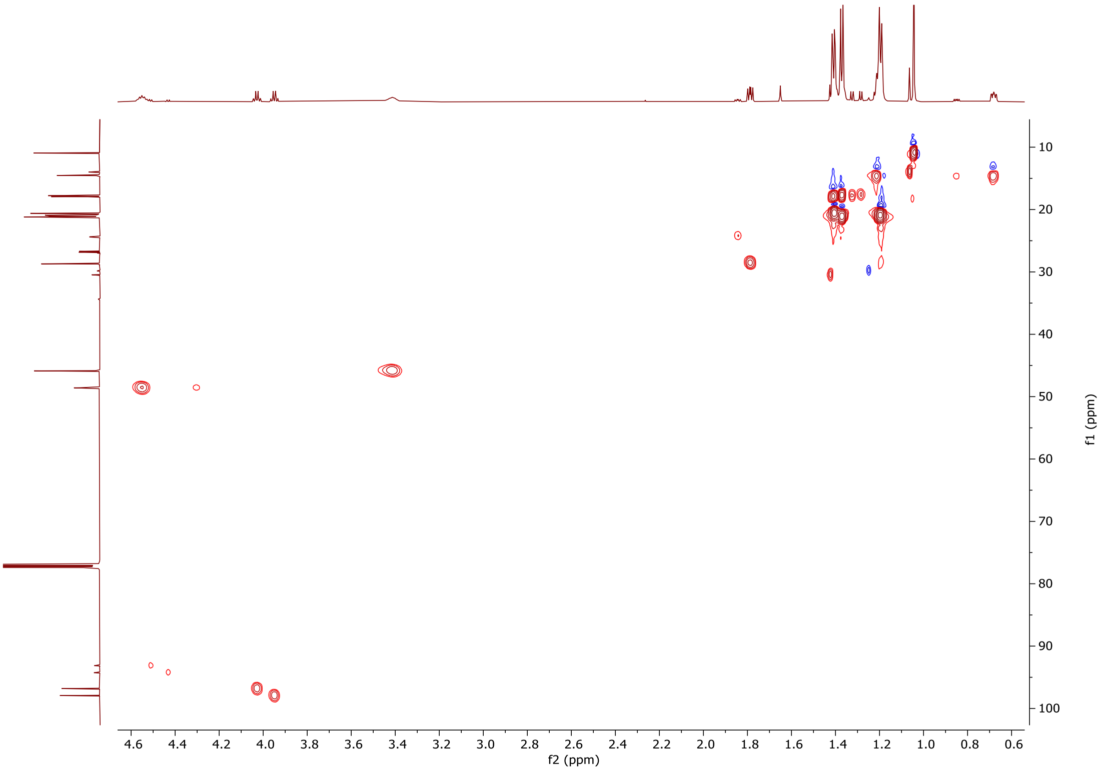

$^{1}H/^{13}C$ HMBC (600/151 MHz, Chloroform-*d*)

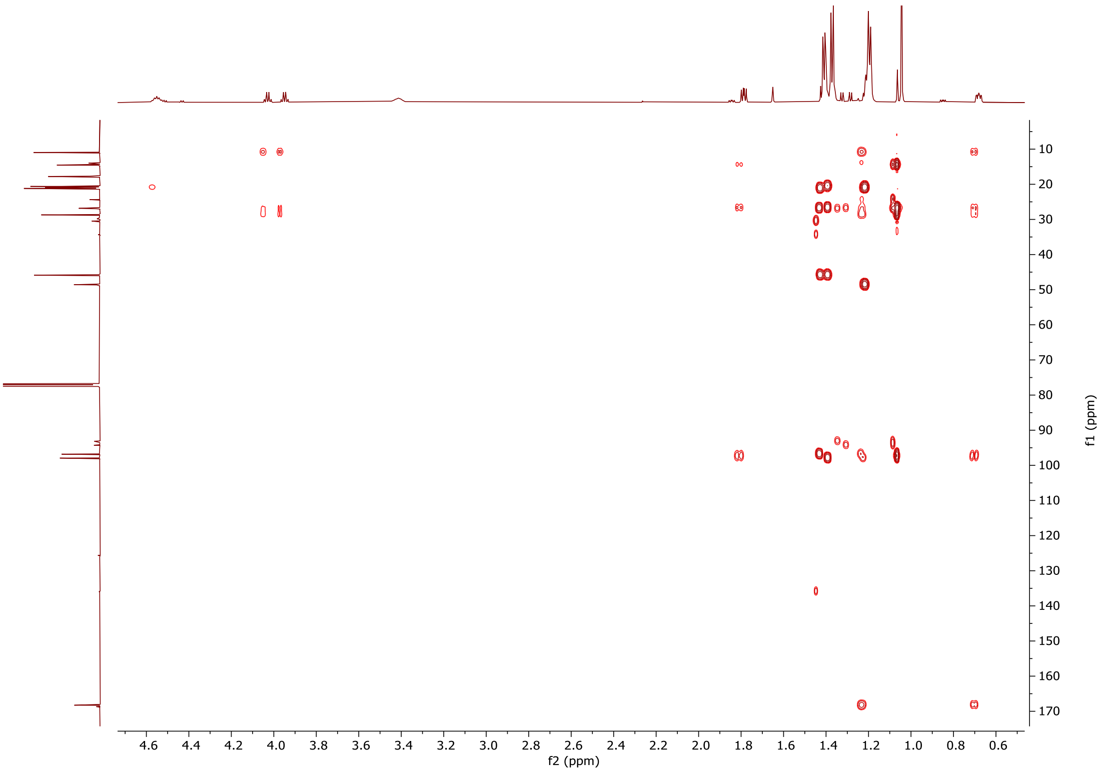

^{1}H NOSEY (600 MHz, Chloroform-*d*)

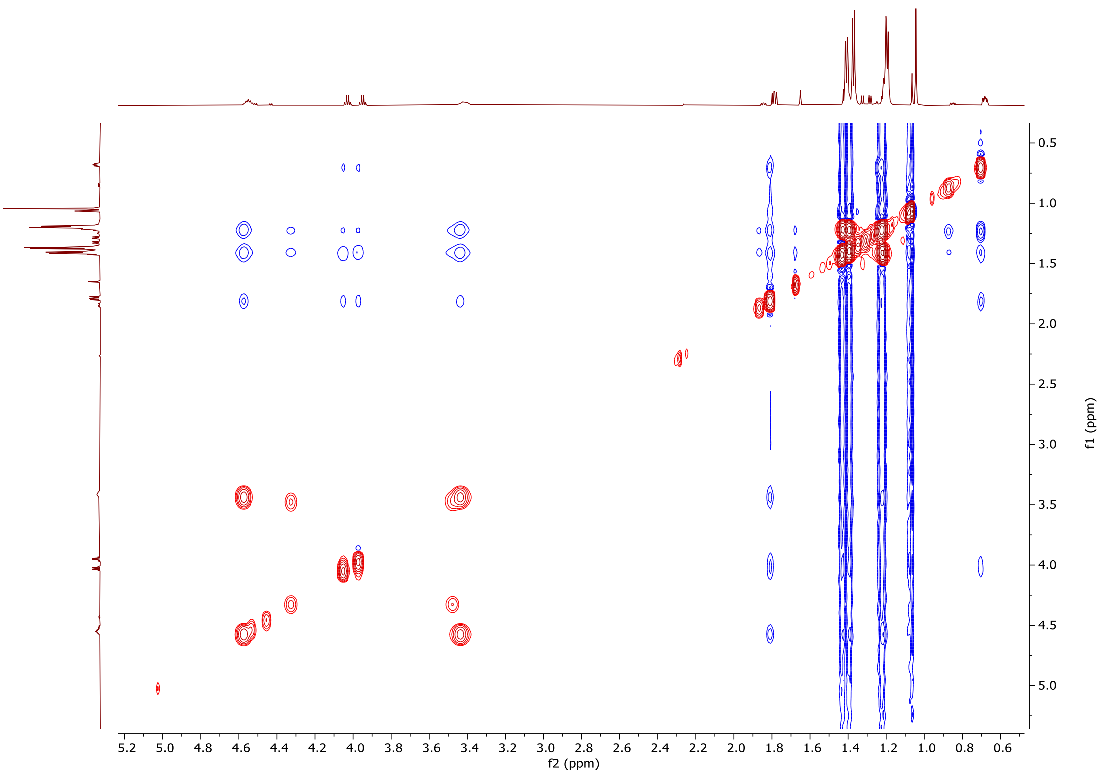

^{19}F NMR (565 MHz, Chloroform-*d*)

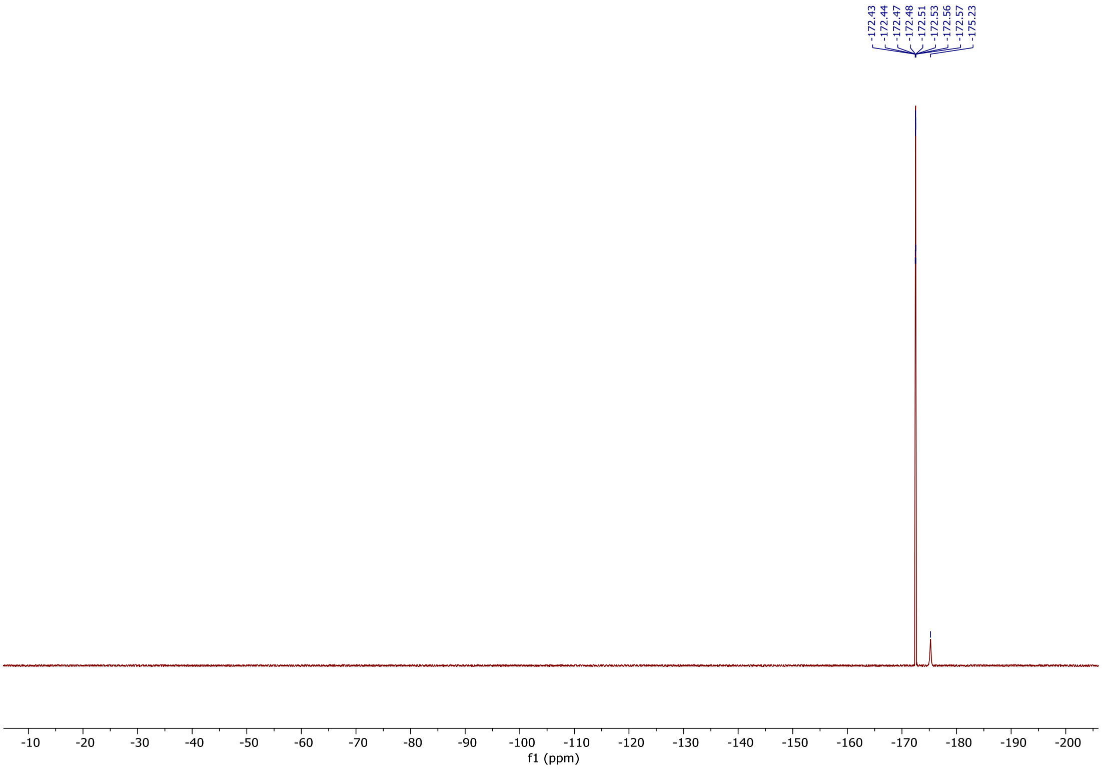

(1*R,2*R**)-2-((*S**)-1-(2,4-Dinitrophenoxy)ethyl)-*N*,*N*-diisopropyl-2-methylcyclopropane-1-carboxamide, 3m**

¹H NMR (600 MHz, Chloroform-*d*)

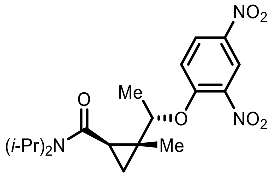

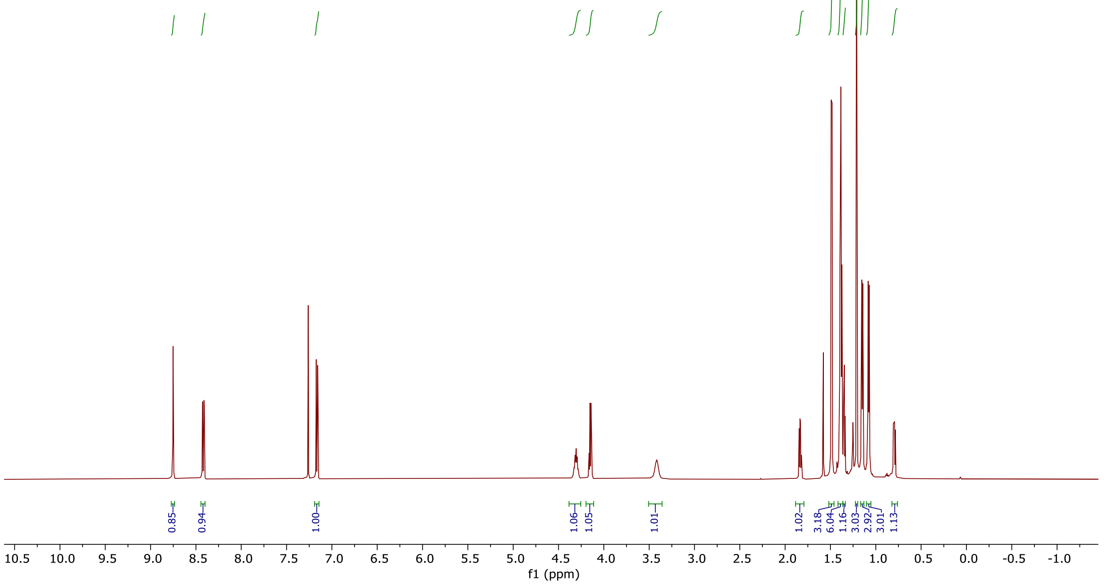

¹³C NMR (151 MHz, Chloroform-*d*)

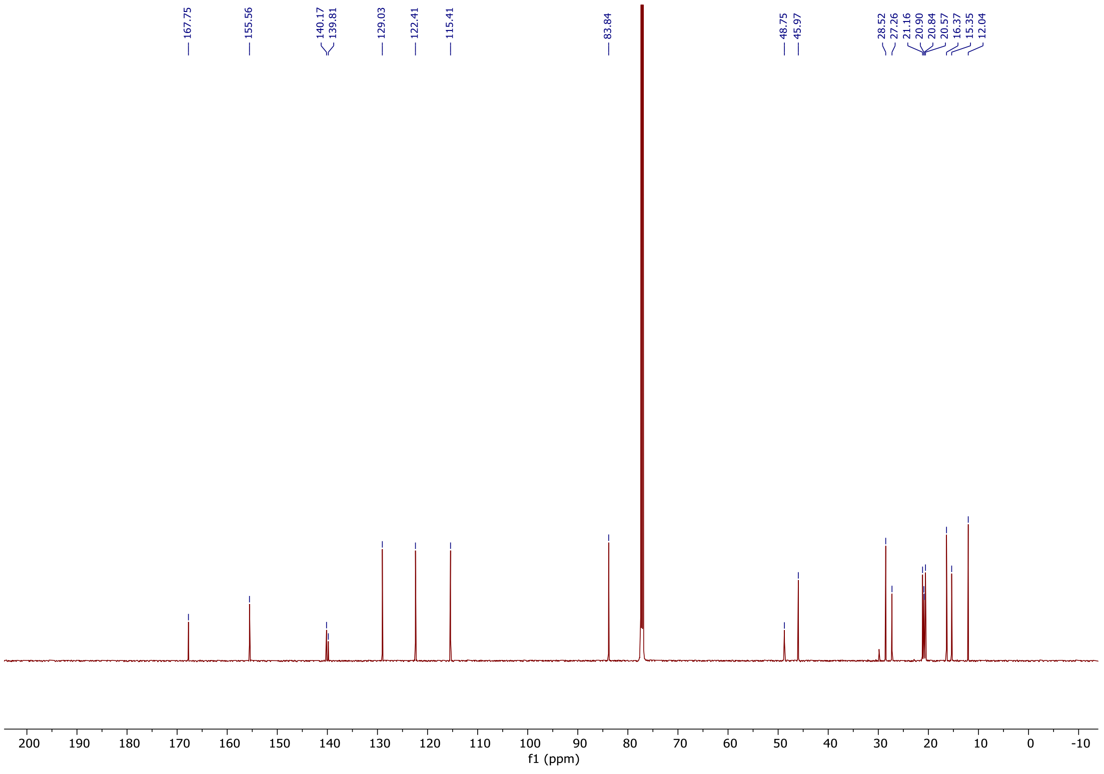

^{1}H COSY (600 MHz, Chloroform-*d*)

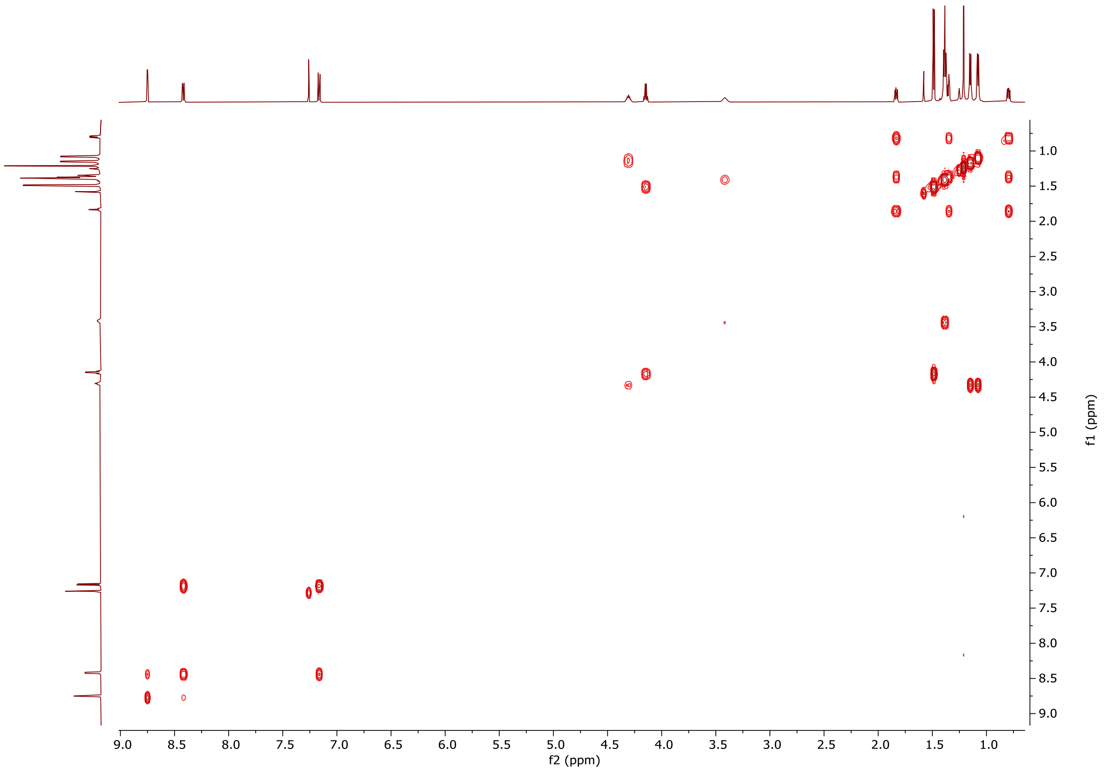

^{1}H/^{13}C HSQC (600/151 MHz, Chloroform-*d*)

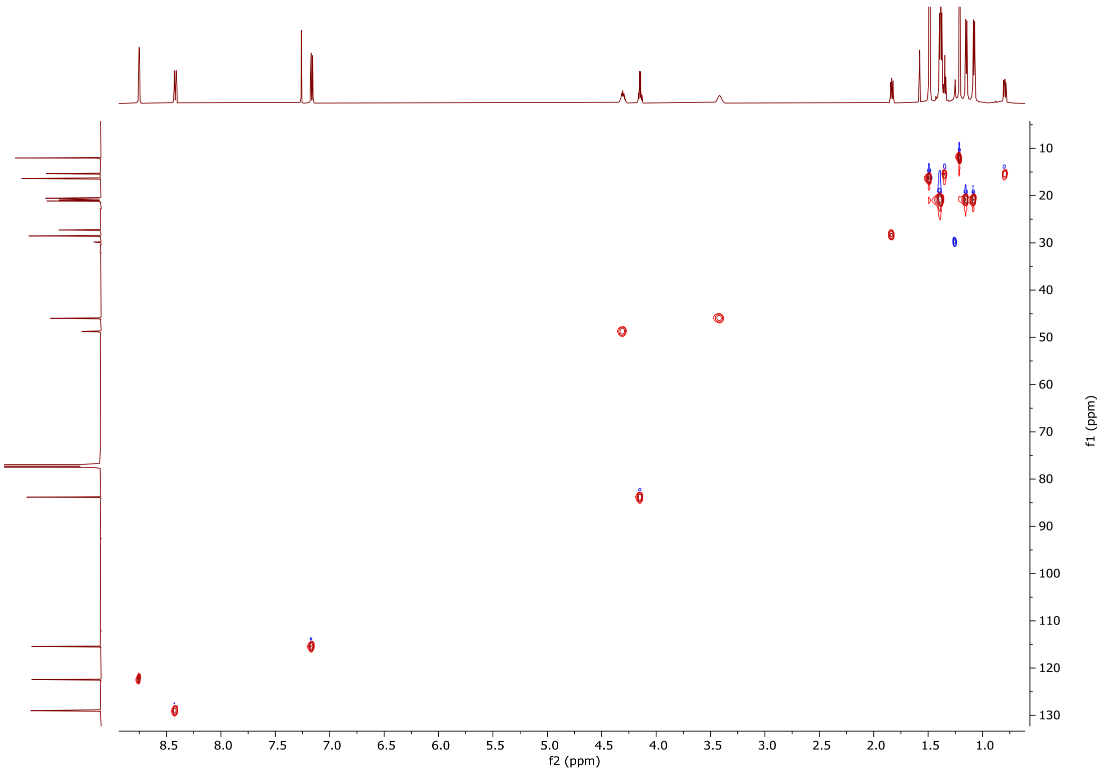

$^{1}H/^{13}C$ **HMBC** (600/151 MHz, Chloroform-*d*)

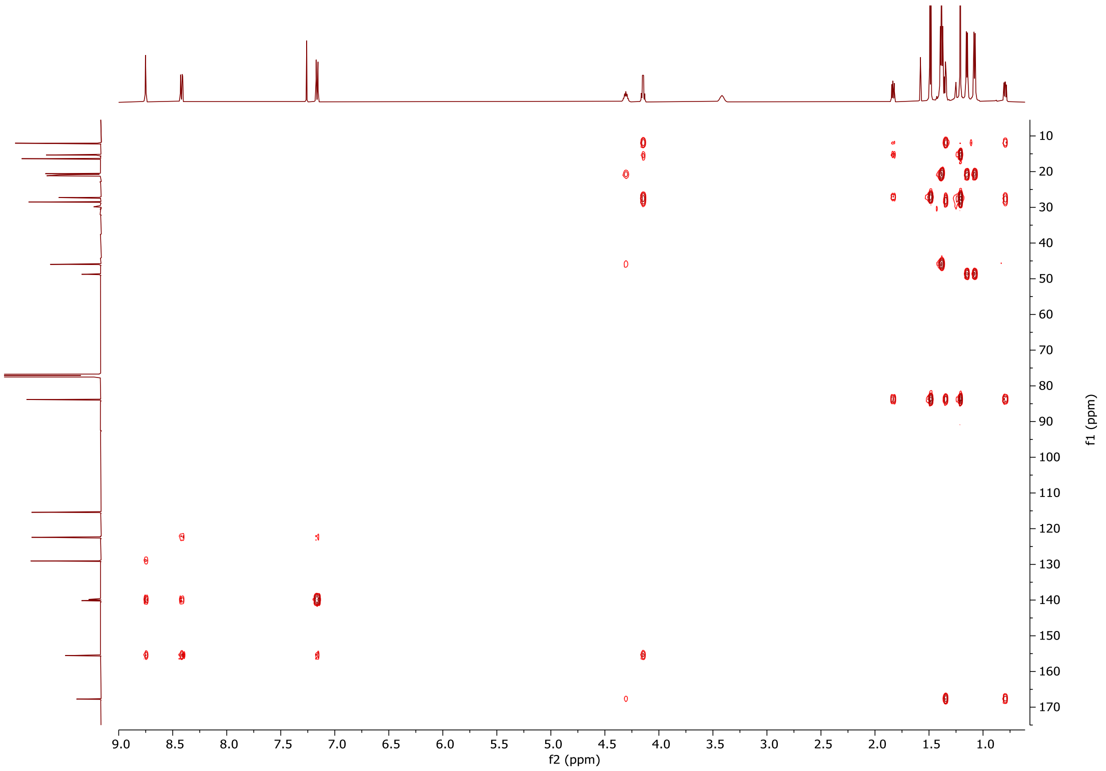

^{1}H **NOSEY** (600 MHz, Chloroform-*d*)

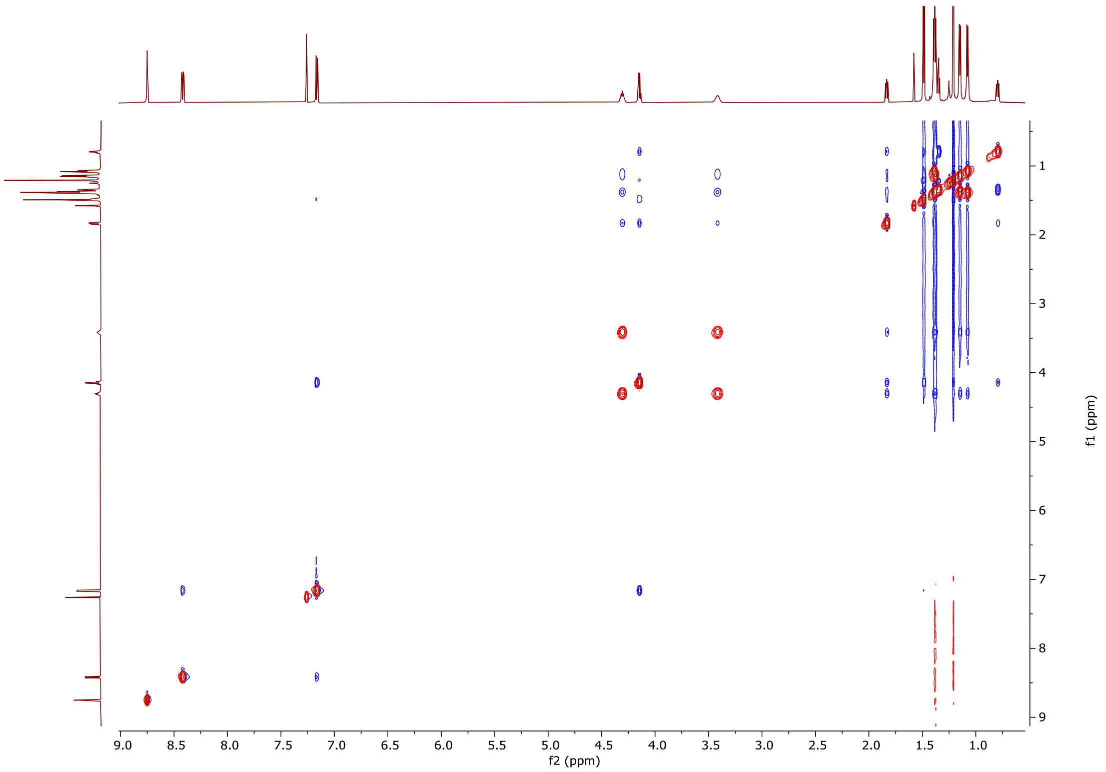

Methyl ((*S)-1-((1*R**,2*R**)-2-(diisopropylcarbamoyl)-1-methylcyclopropyl)ethyl)(tosyl)carbamate, (*S**)-3n**

^{1}H NMR (600 MHz, Chloroform-*d*)

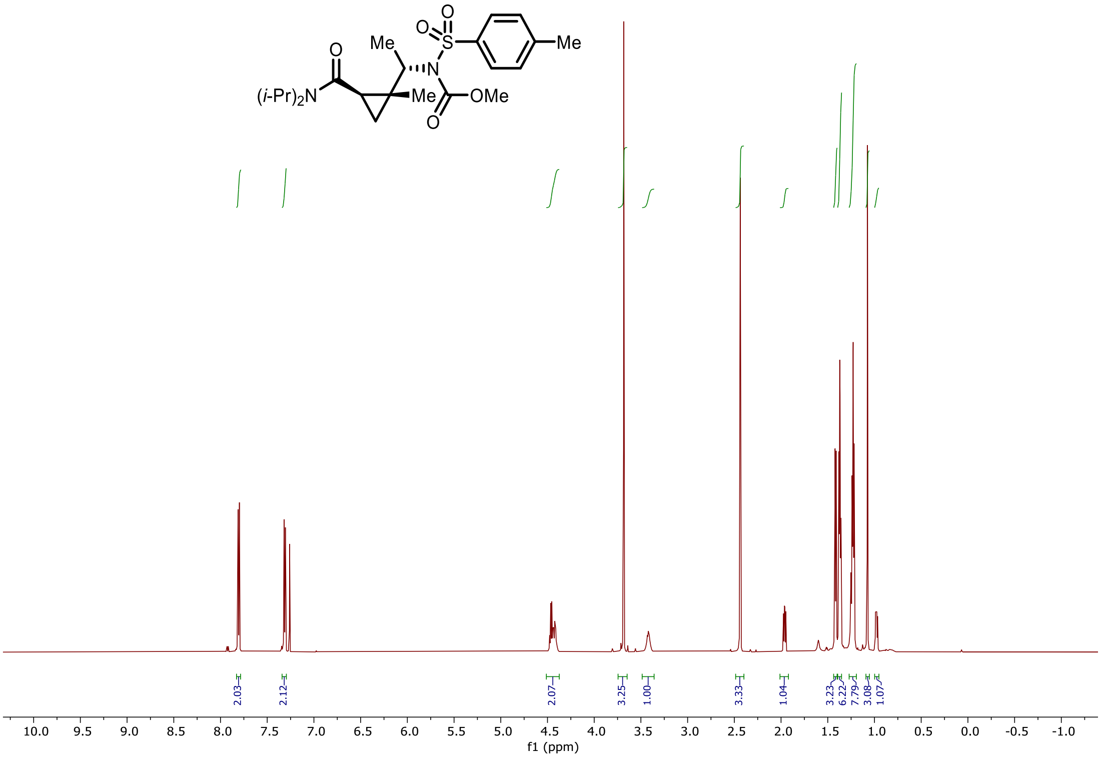

^{13}C NMR (151 MHz, Chloroform-*d*)

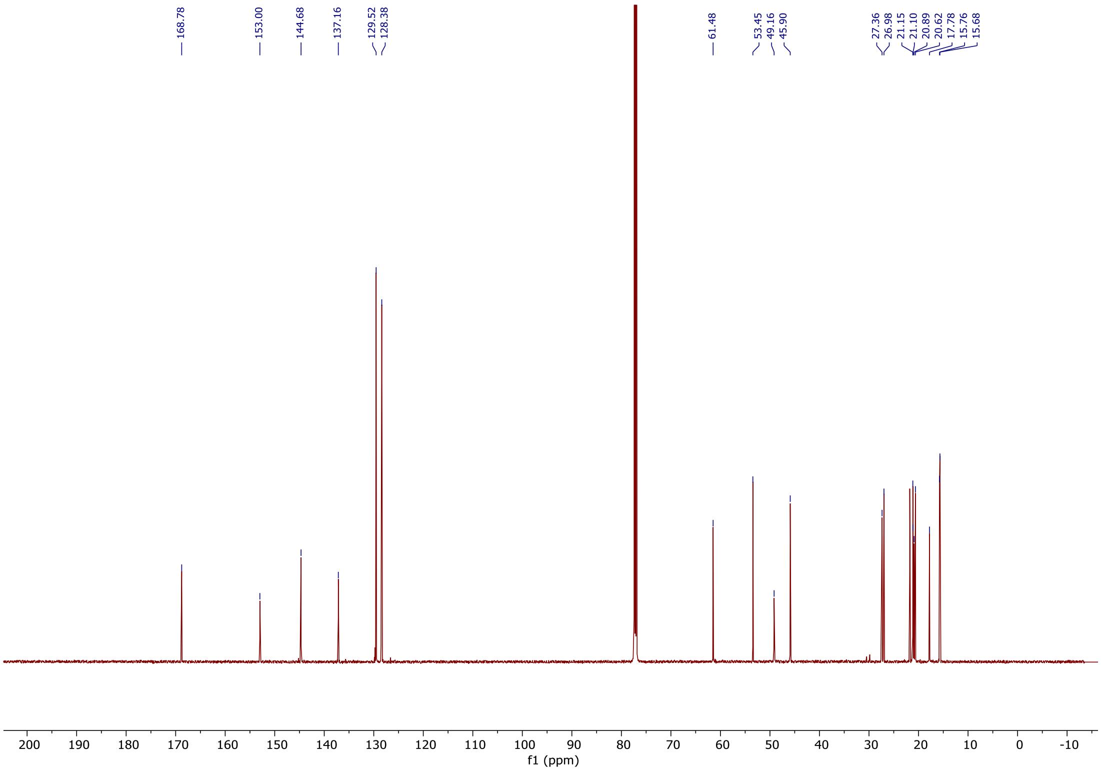

^{1}H COSY (600 MHz, Chloroform-*d*)

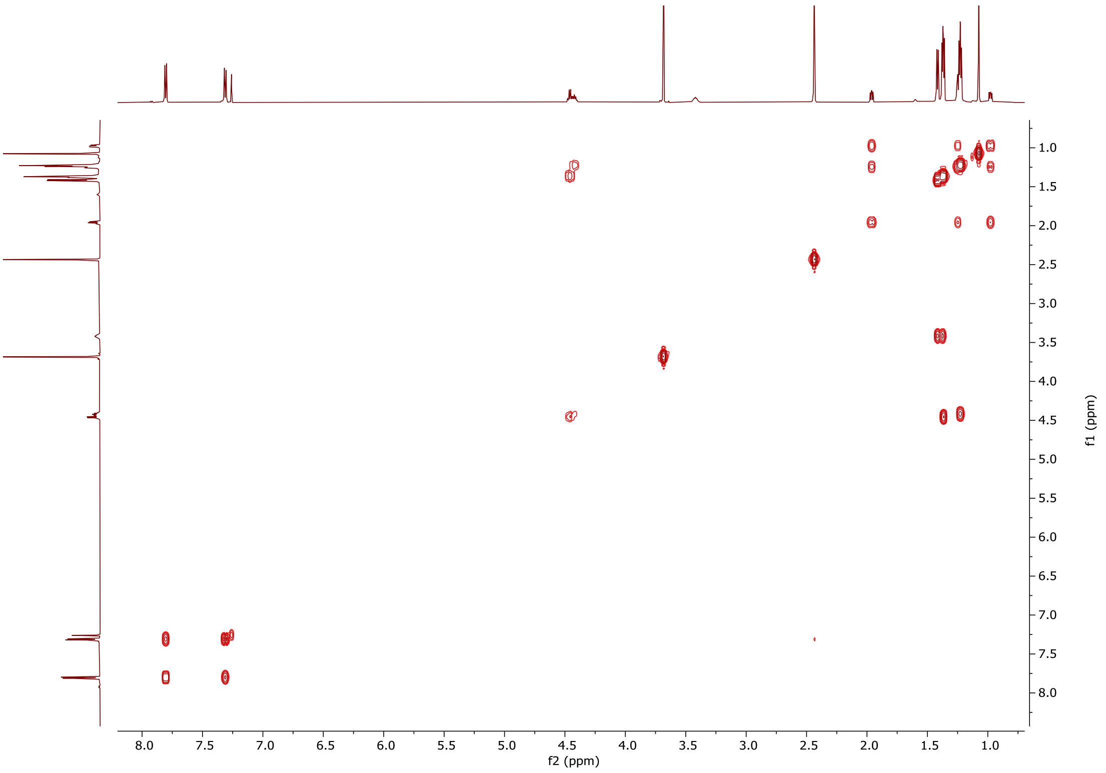

^{1}H/^{13}C HSQC (600/151 MHz, Chloroform-*d*)

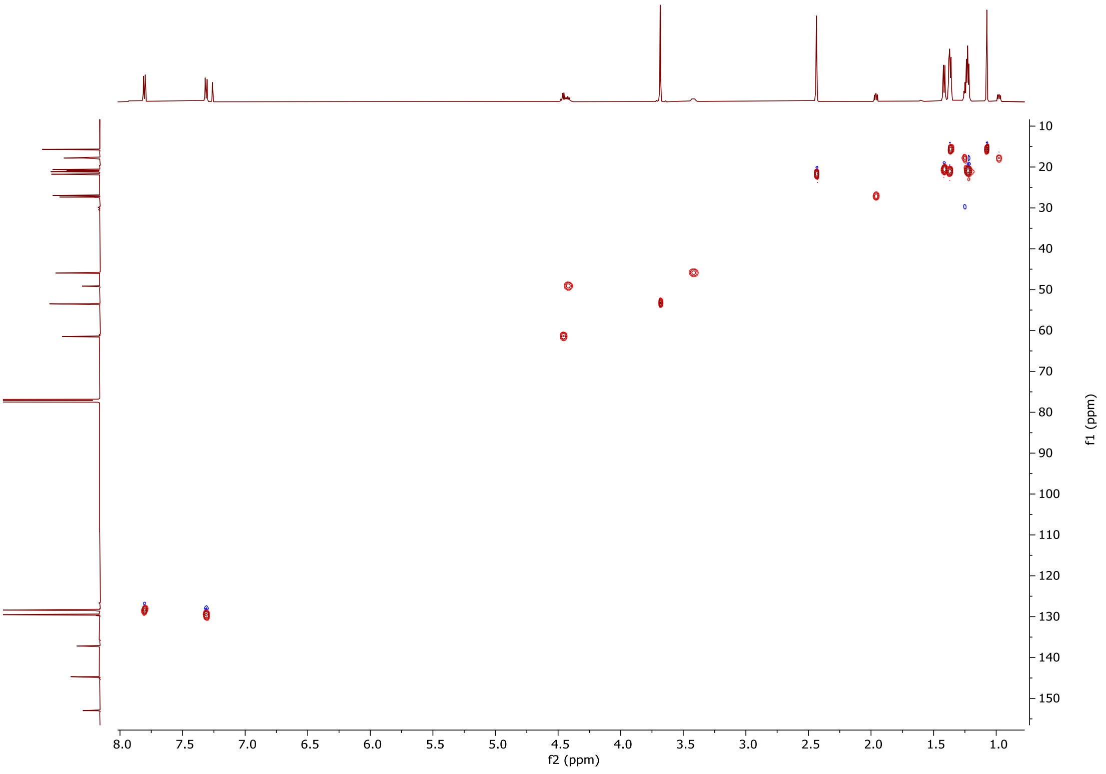

^{1}H/^{13}C HMBC (600/151 MHz, Chloroform-*d*)

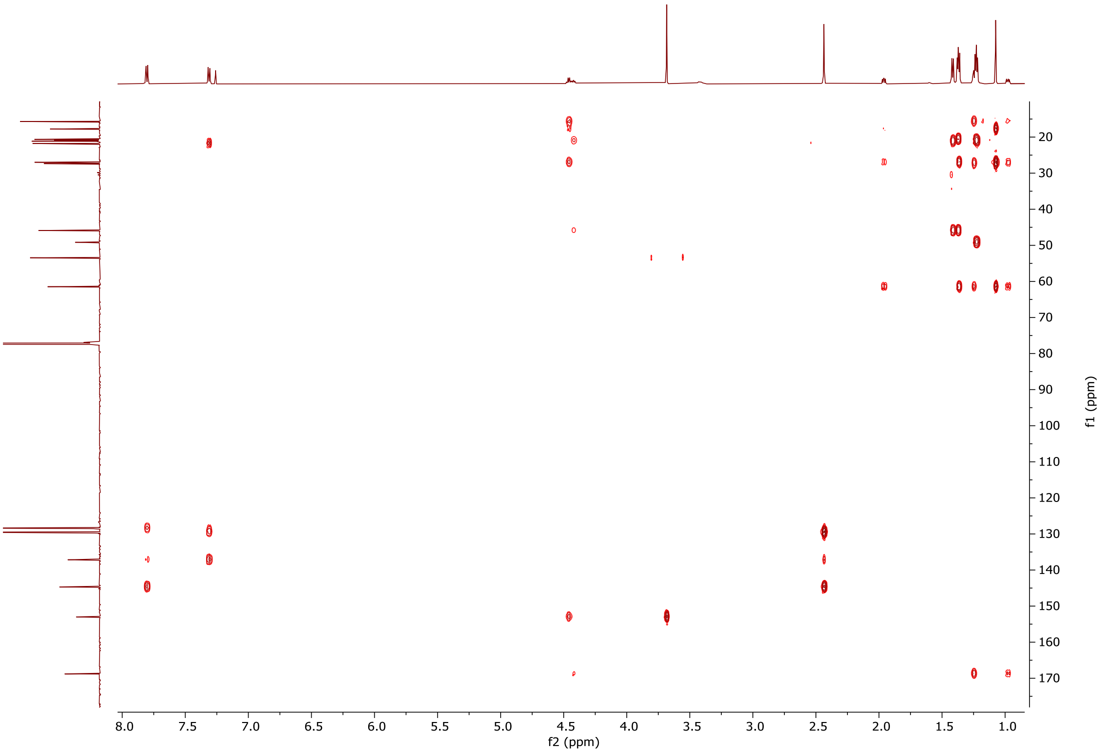

^{1}H NOSEY (600 MHz, Chloroform-*d*)

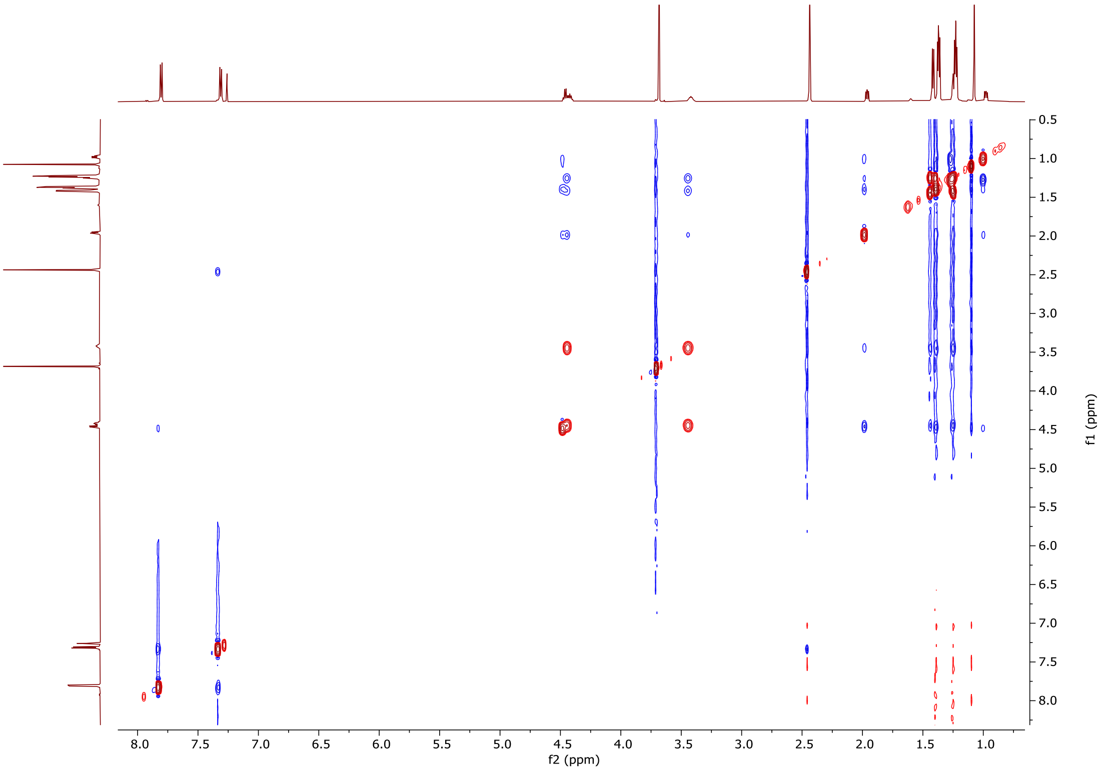

Methyl ((*R)-1-((1*R**,2*R**)-2-(diisopropylcarbamoyl)-1-methylcyclopropyl)ethyl)(tosyl)carbamate, (*R**)-3n**

^{1}H NMR (600 MHz, Chloroform-*d*)

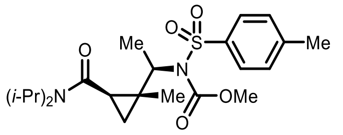

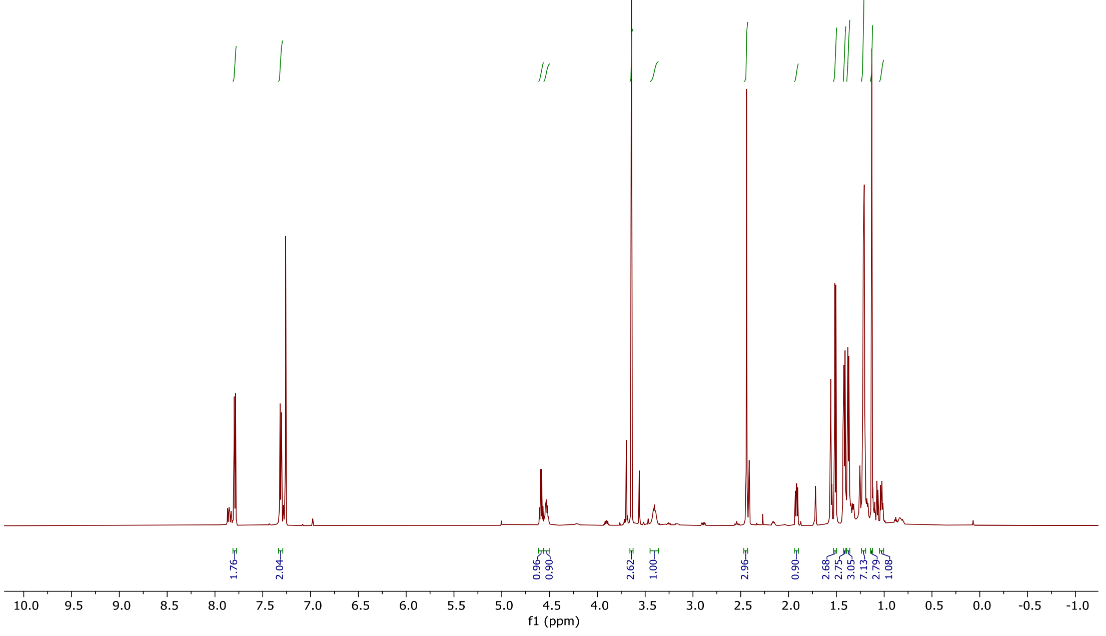

^{13}C NMR (151 MHz, Chloroform-*d*)

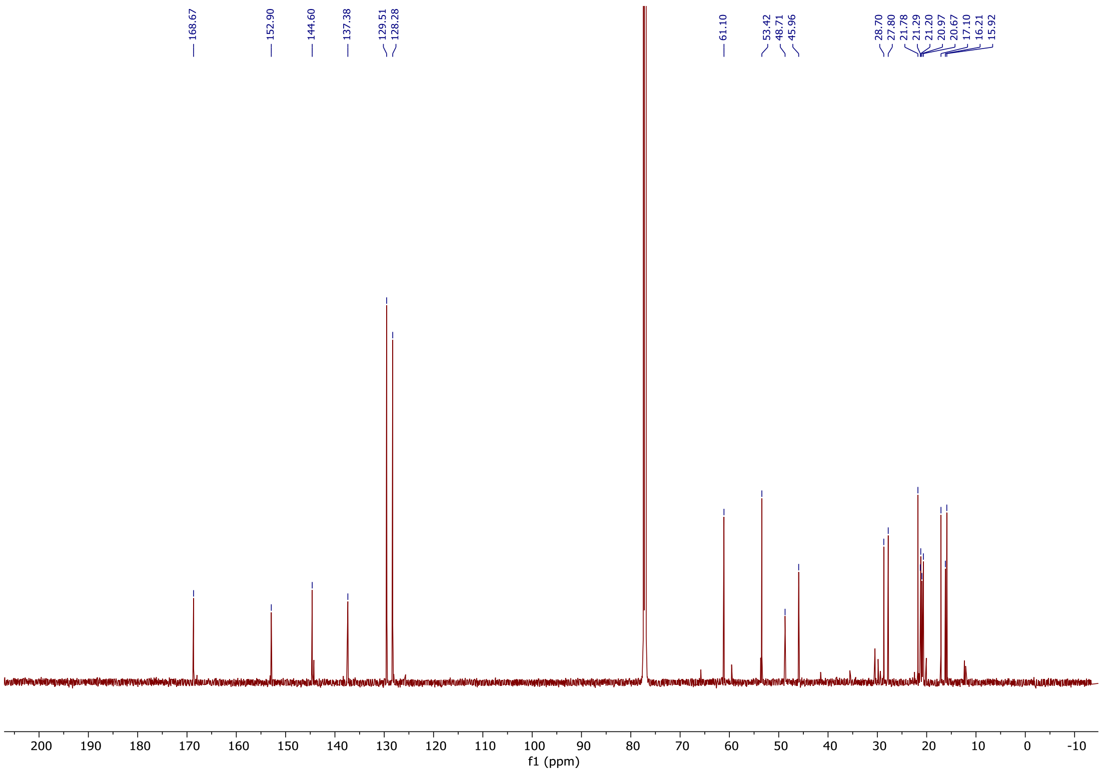

^{1}H COSY (600 MHz, Chloroform-*d*)

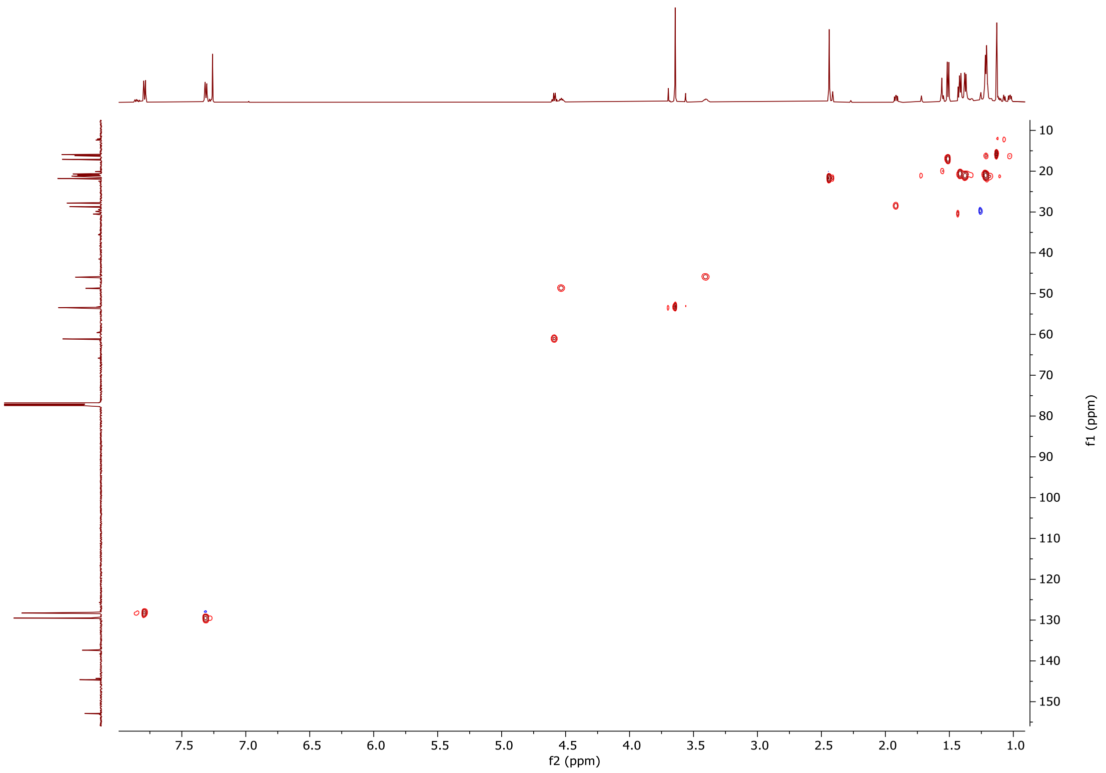

^{1}H/^{13}C HSQC (600/151 MHz, Chloroform-*d*)

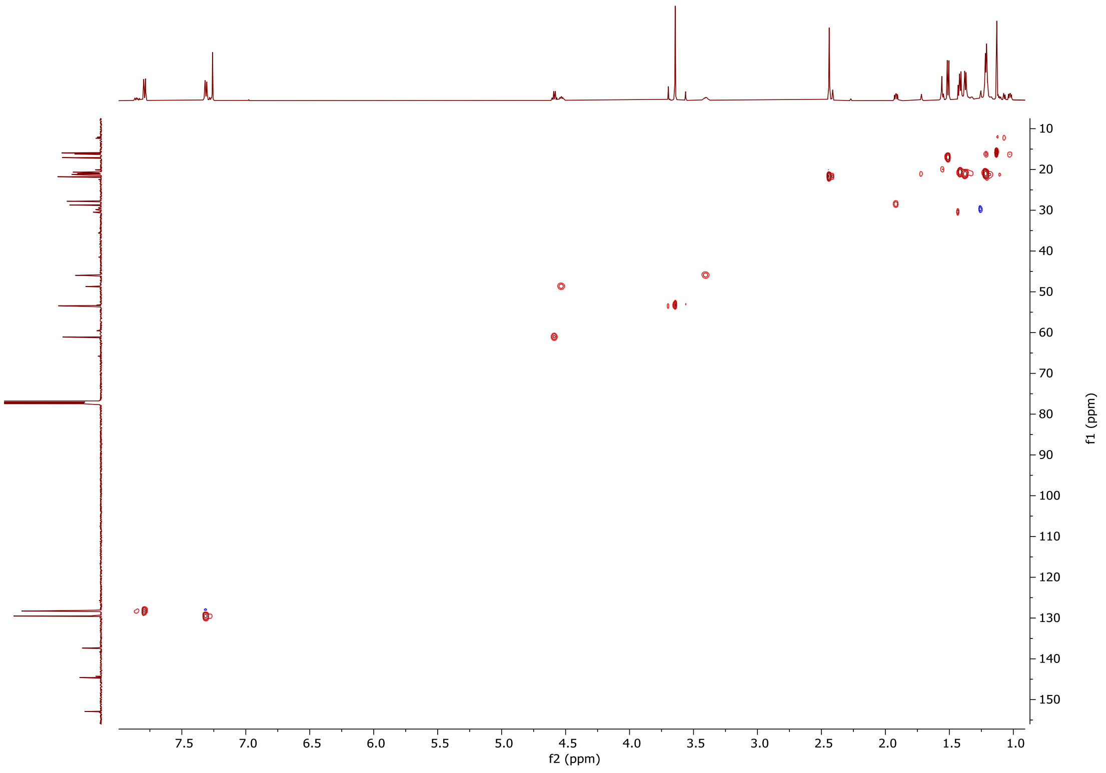

$^{1}H/^{13}C$ HMBC (600/151 MHz, Chloroform-*d*)

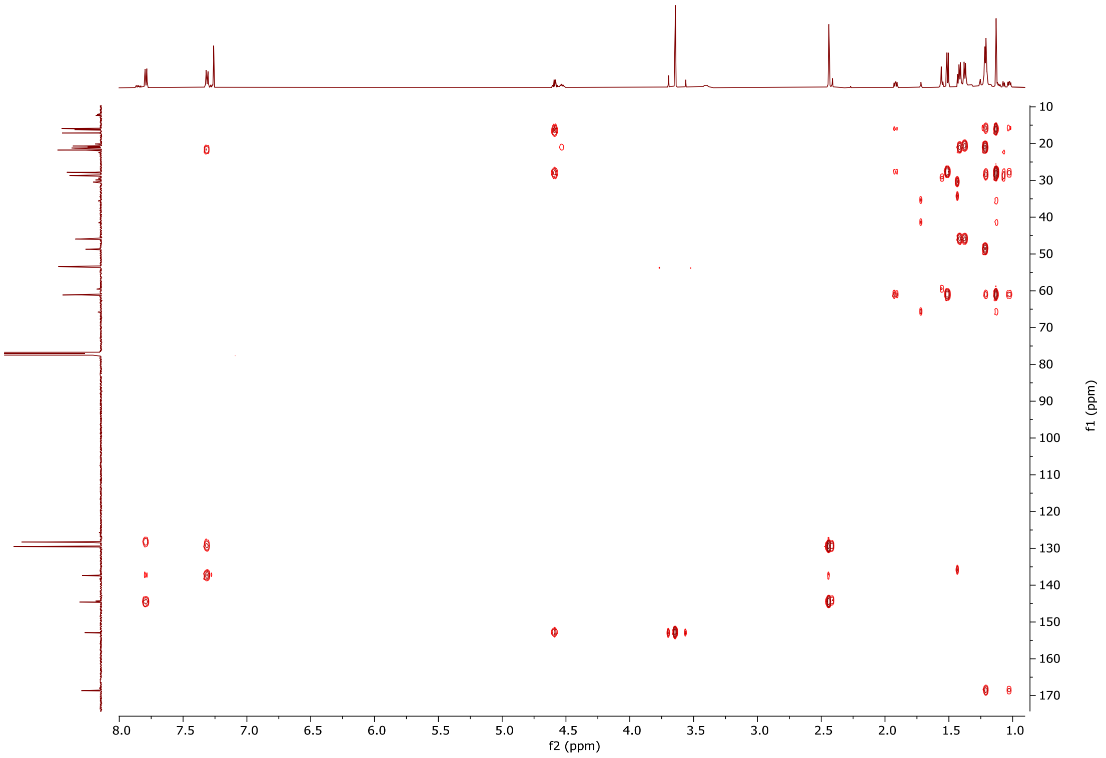

^{1}H NOSEY (600 MHz, Chloroform-*d*)

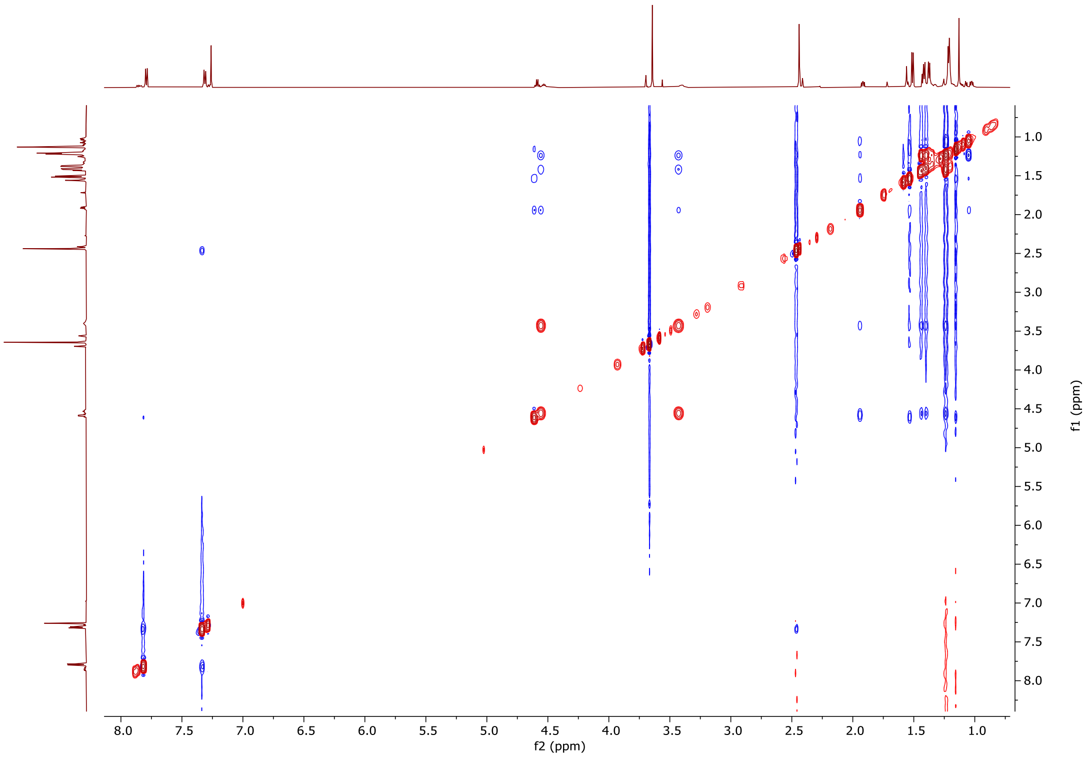

(1*R,2*R**)-2-((*S**)-1-(2*H*-Tetrazol-2-yl)ethyl)-*N*,*N*-diisopropyl-2-methylcyclopropane-1-carboxamide, 3o**

^{1}H NMR (600 MHz, Chloroform-*d*)

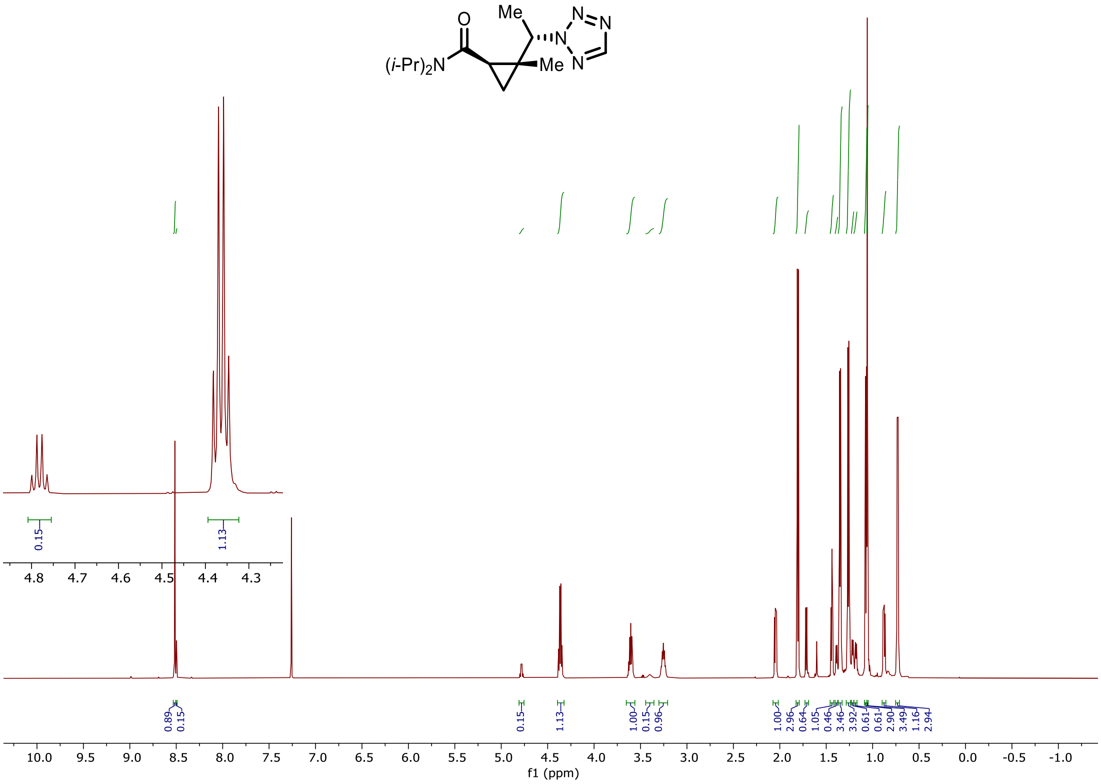

^{13}C NMR (151 MHz, Chloroform-*d*)

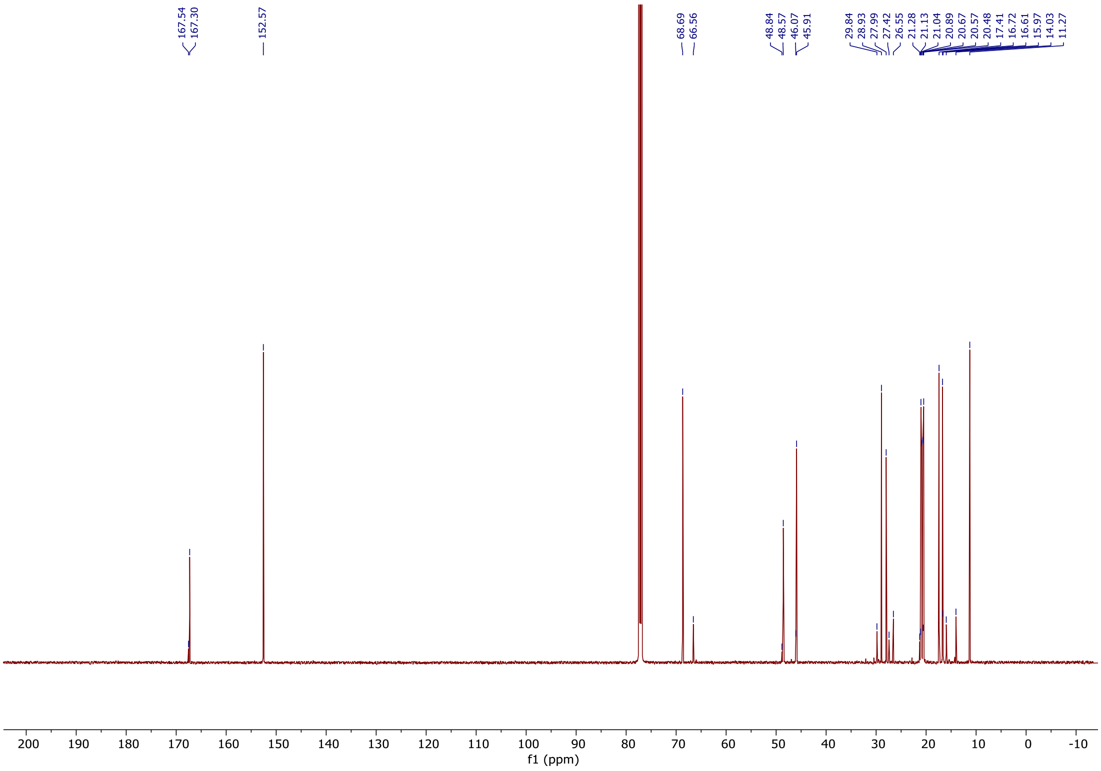

^{1}H COSY (600 MHz, Chloroform-*d*)

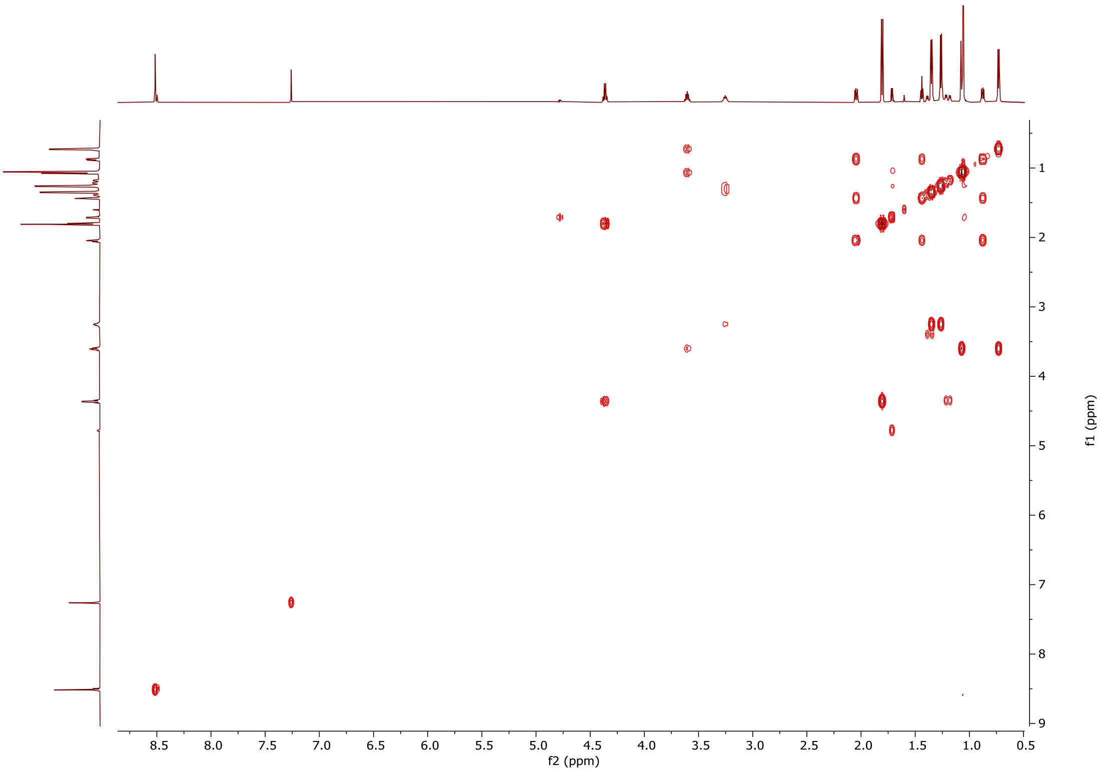

^{1}H/^{13}C HSQC (600/151 MHz, Chloroform-*d*)

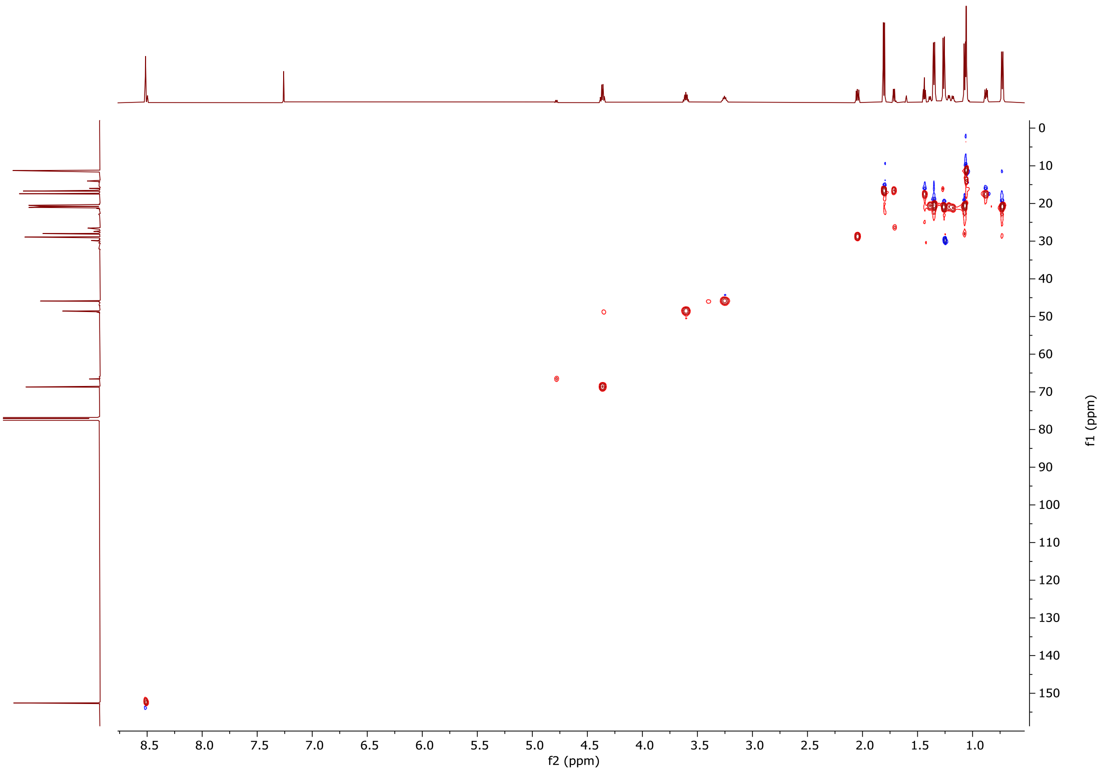

$^{1}H/^{13}C$ HMBC (600/151 MHz, Chloroform-*d*)

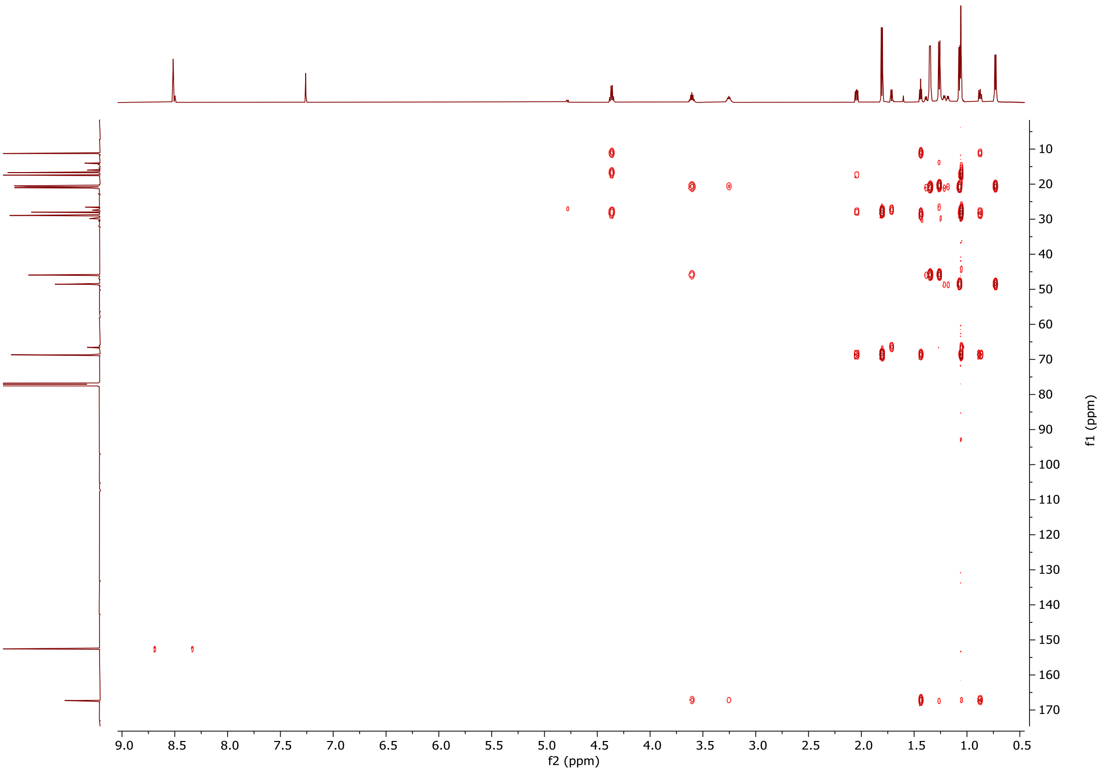

^{1}H NOSEY (600 MHz, Chloroform-*d*)

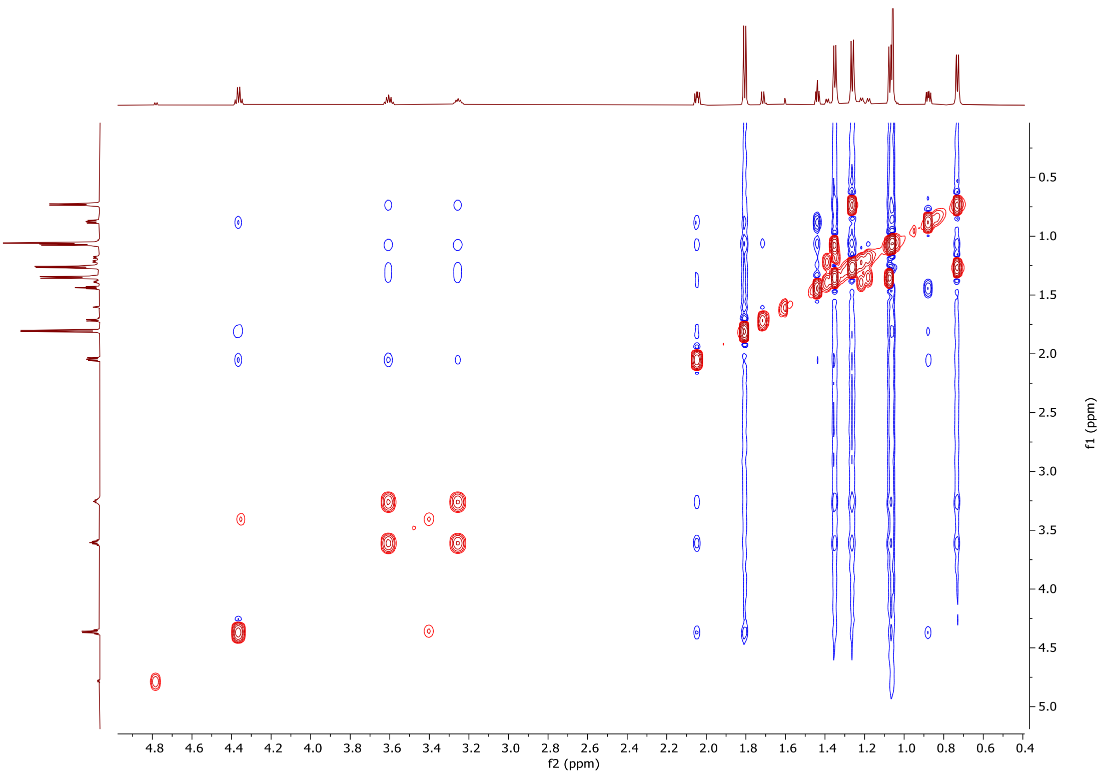

(1*R,2*R**)-*N*,*N*-diisopropyl-2-methyl-2-((*S**)-1-((trifluoromethyl)sulfonamido)ethyl)cyclopropane-1-carboxamide, 3p**

^{1}H NMR (600 MHz, Chloroform-*d*)

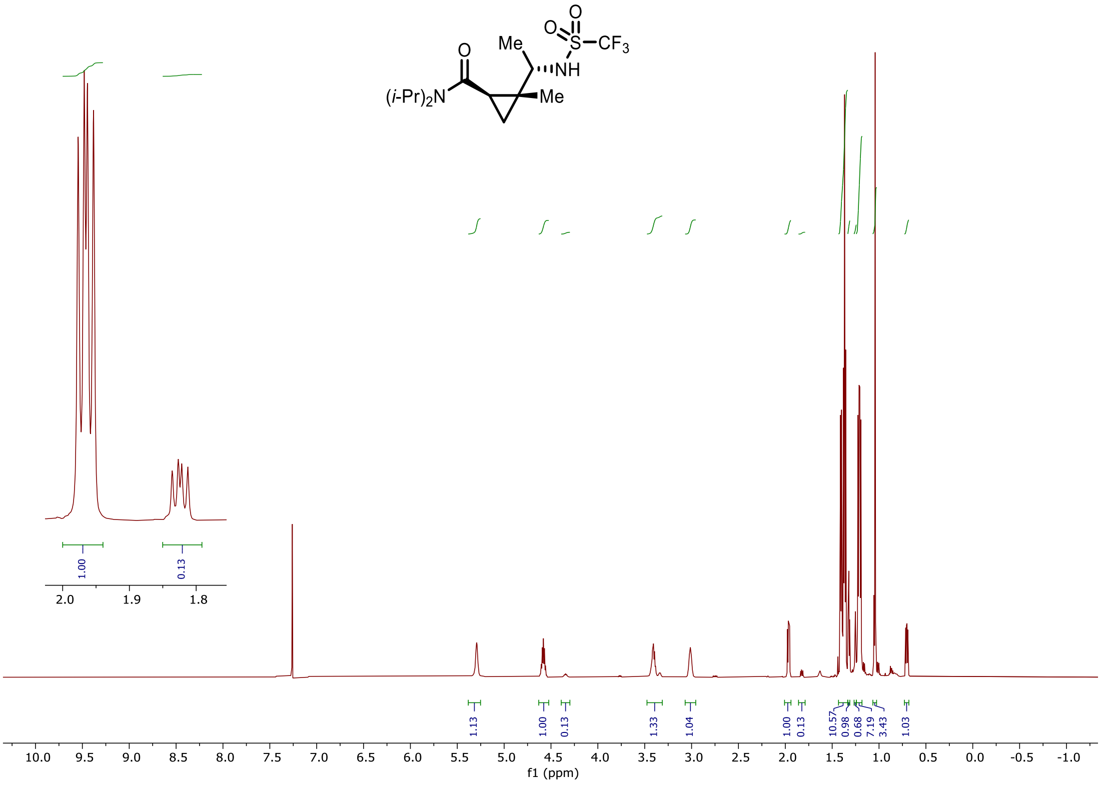

^{13}C NMR (151 MHz, Chloroform-*d*)

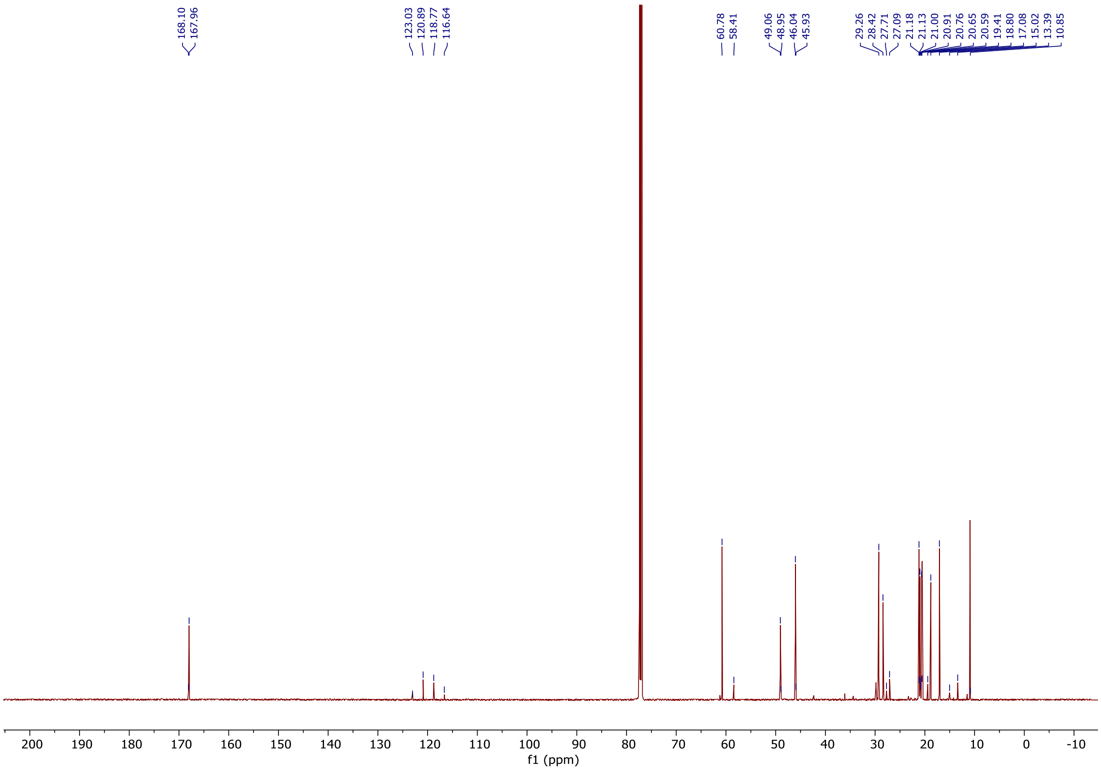

^{1}H COSY (600 MHz, Chloroform-*d*)

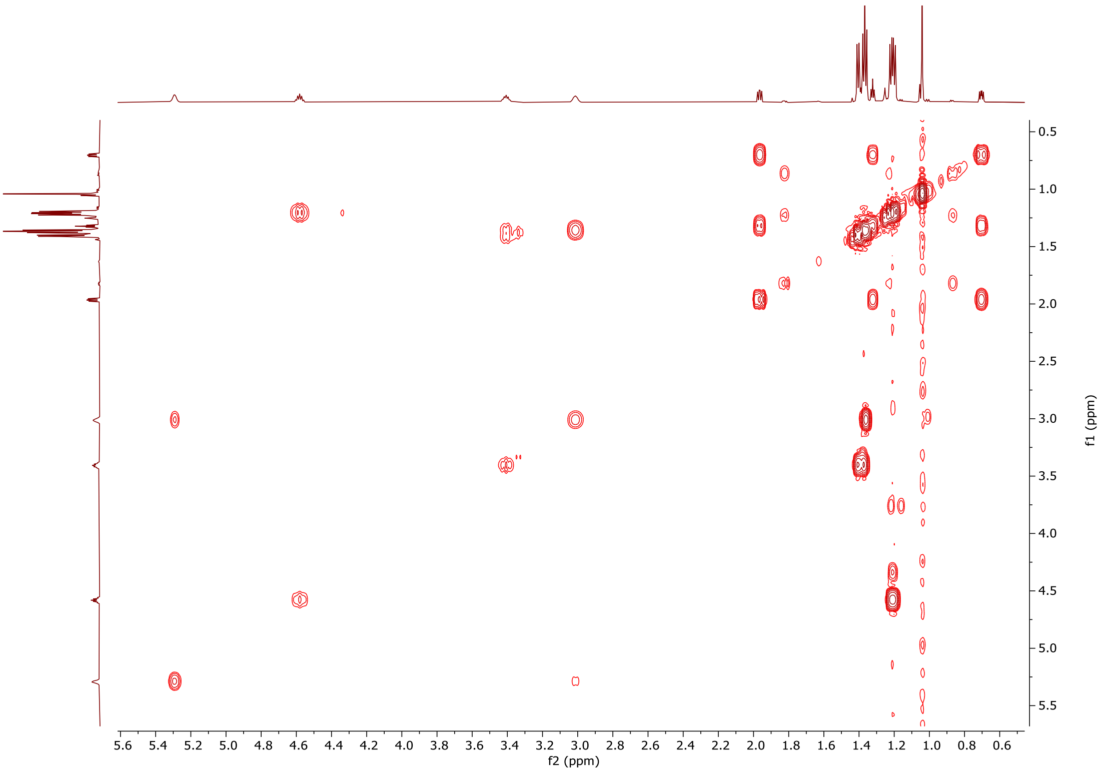

^{1}H/^{13}C HSQC (600/151 MHz, Chloroform-*d*)

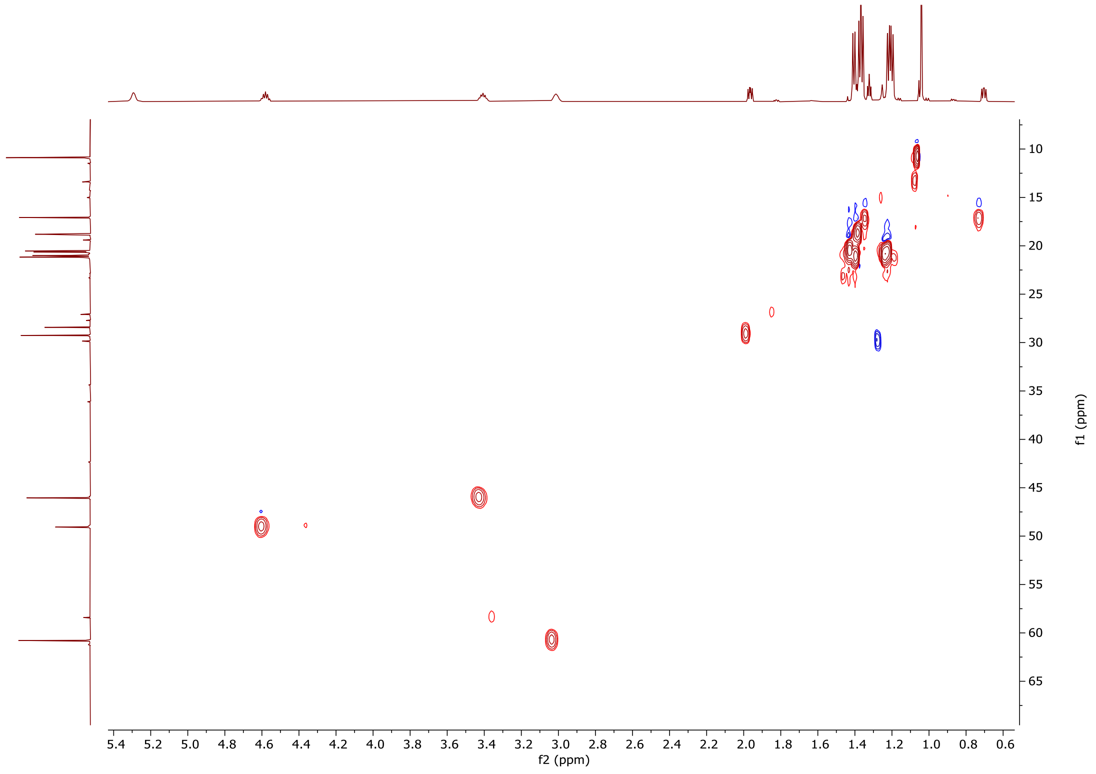

$^{1}H/^{13}C$ **HMBC** (600/151 MHz, Chloroform-*d*)

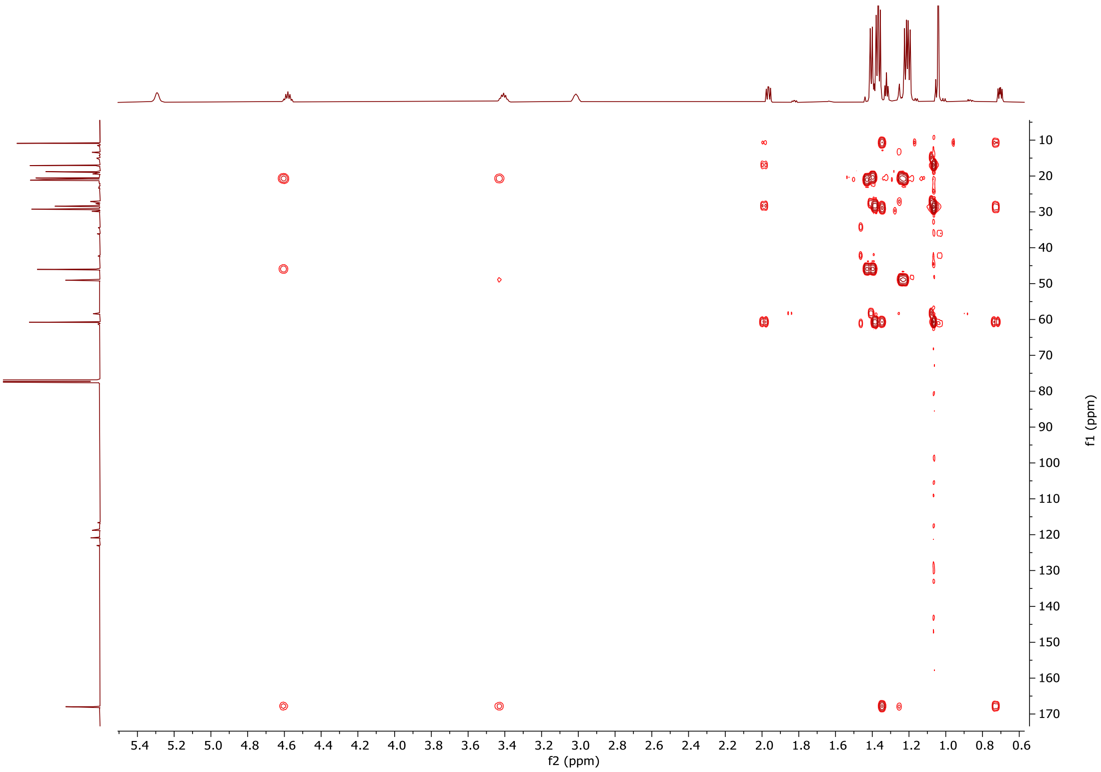

^{1}H **NOSEY** (600 MHz, Chloroform-*d*)

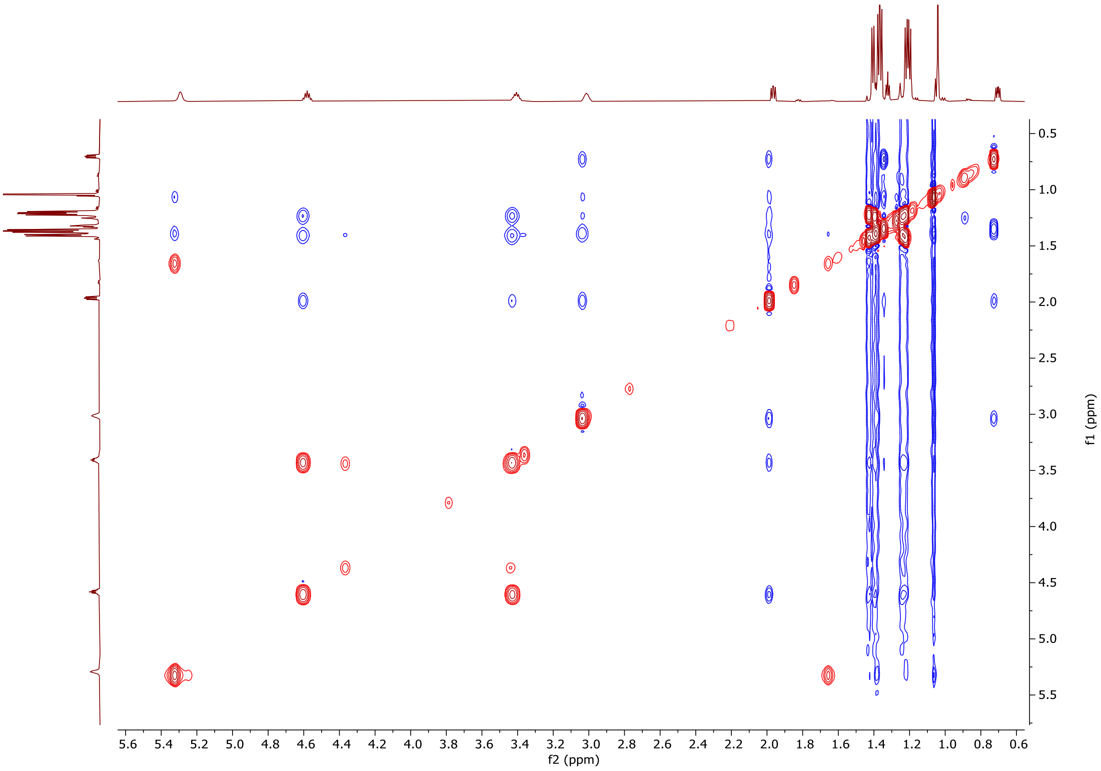

^{19}F NMR (565 MHz, Chloroform-*d*)

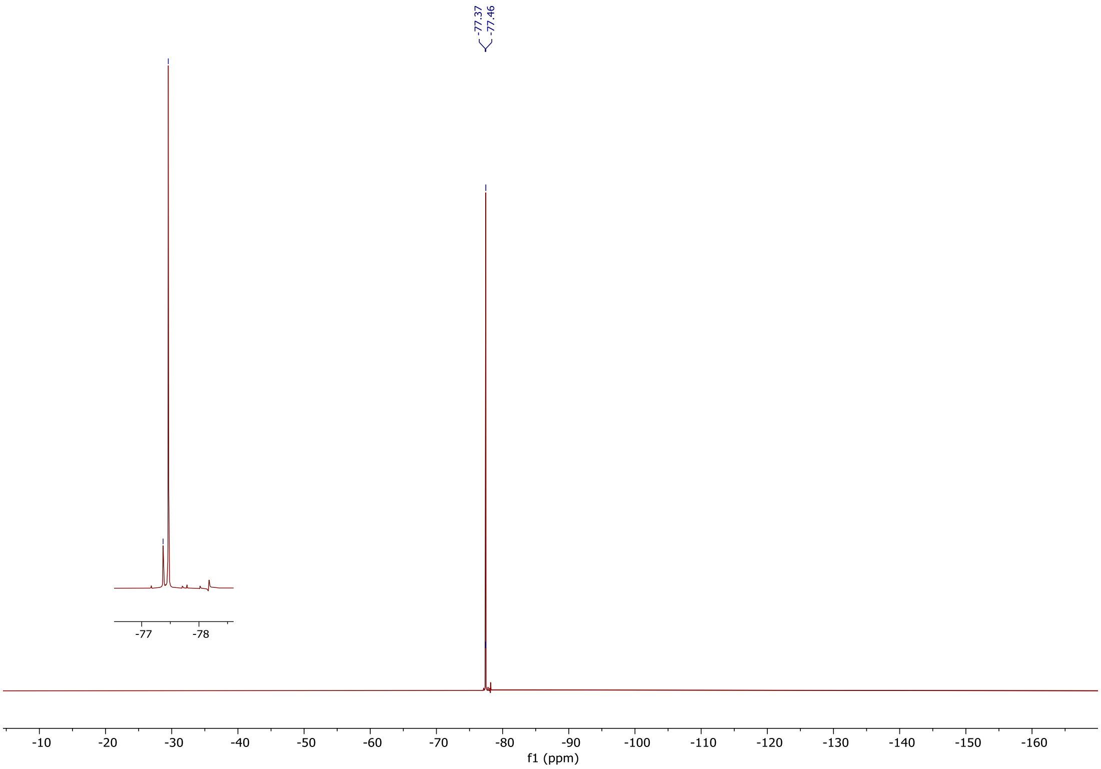

(*S)-1-((1*R**,2*R**)-2-(diisopropylcarbamoyl)-1-methylcyclopropyl)butyl 2,2,2-trifluoroacetate, 3q**

^{1}H NMR (600 MHz, Chloroform-*d*)

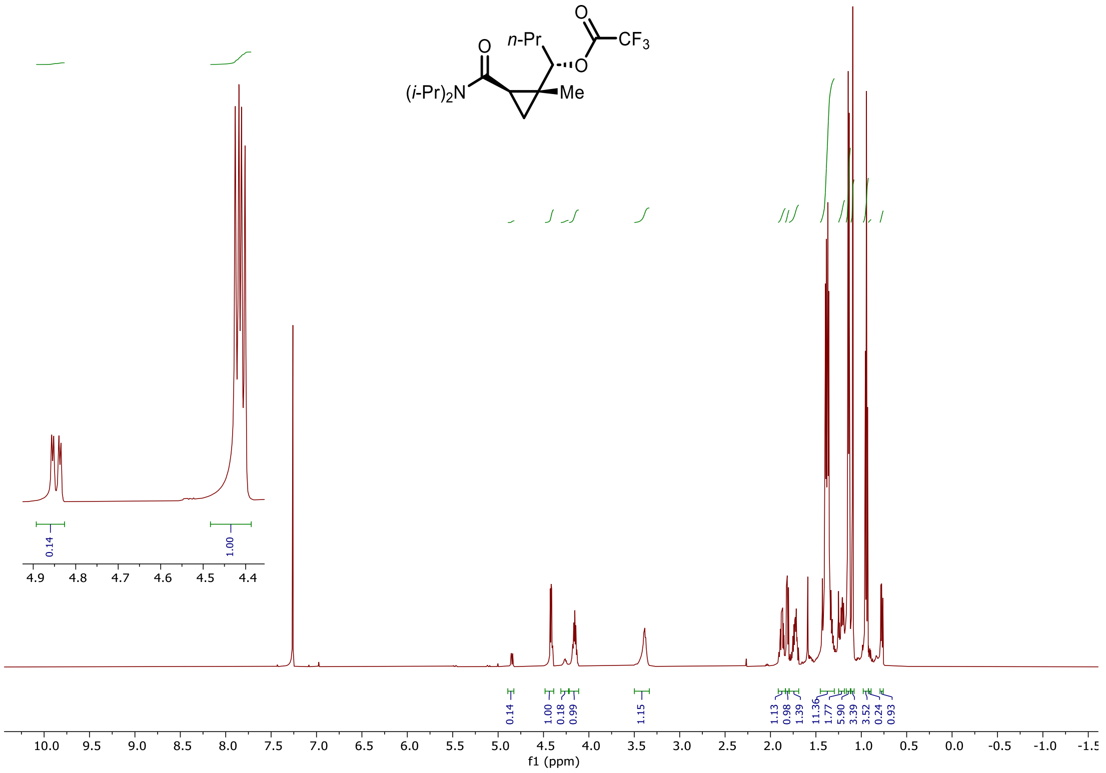

^{13}C NMR (151 MHz, Chloroform-*d*)

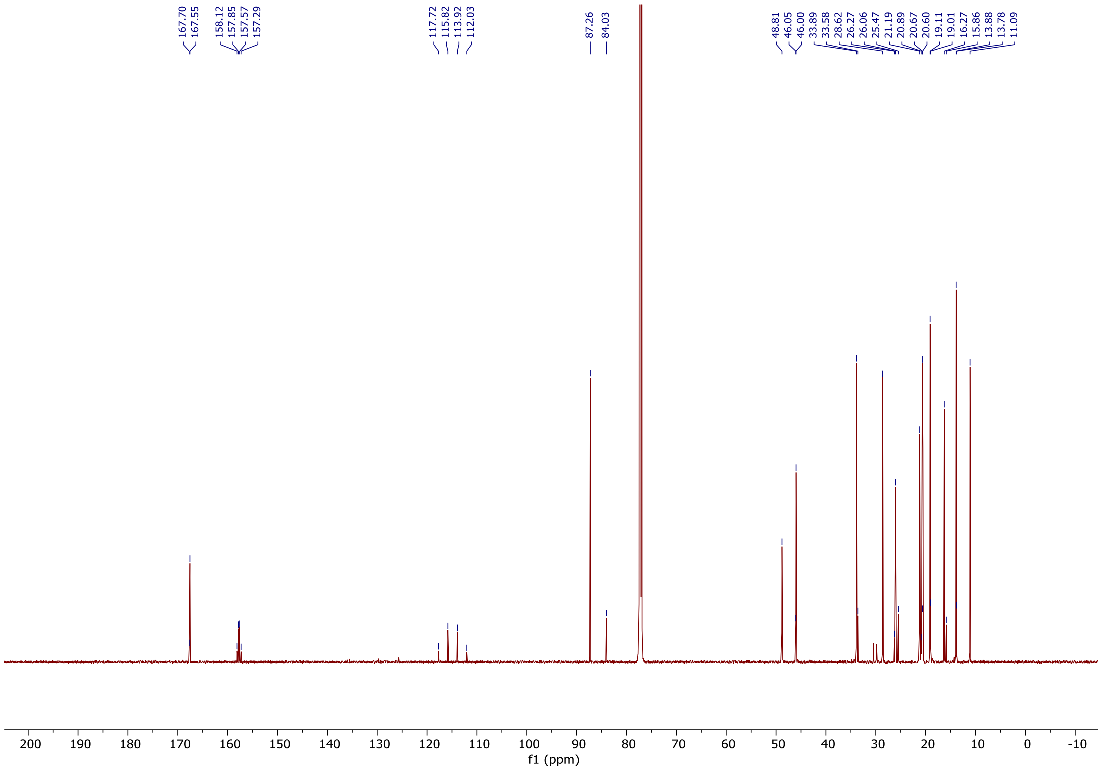

^{1}H COSY (600 MHz, Chloroform-*d*)

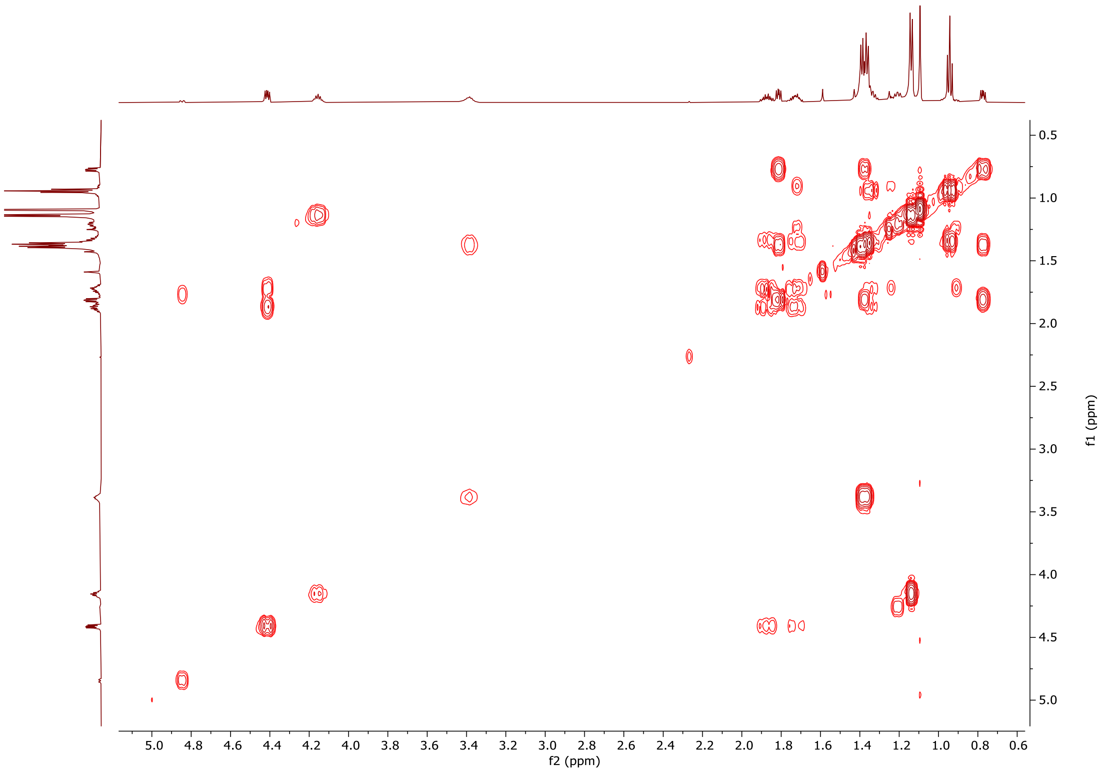

^{1}H/^{13}C HSQC (600/151 MHz, Chloroform-*d*)

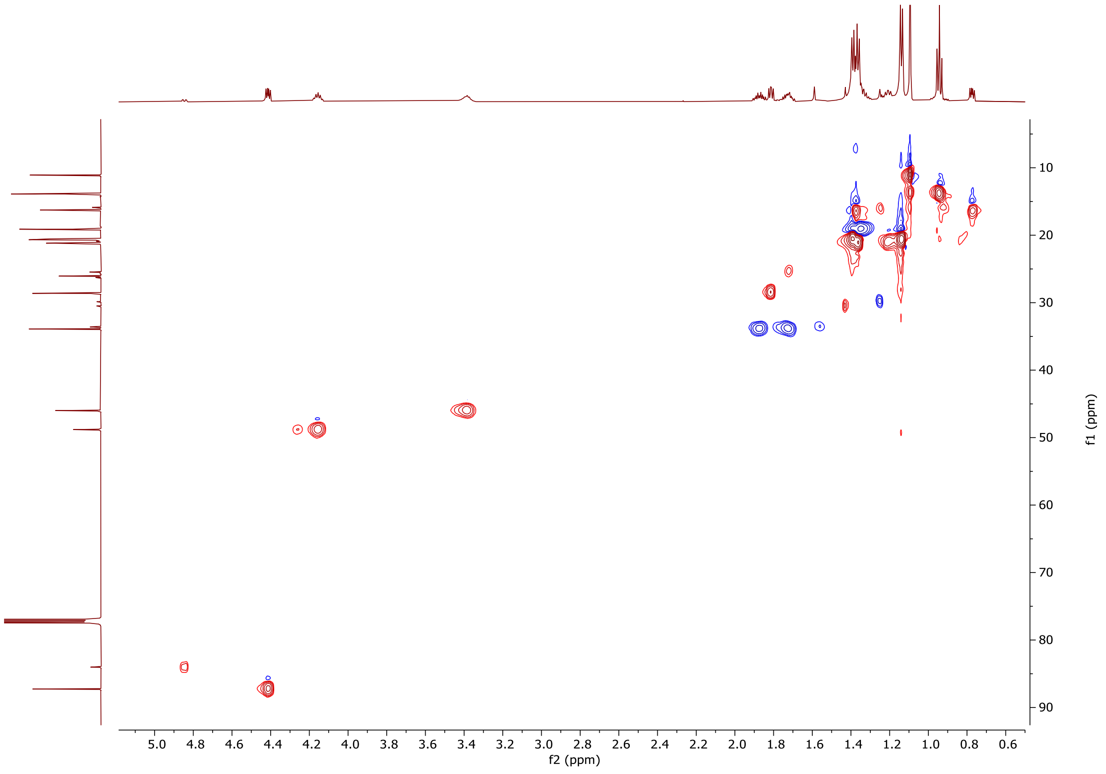

$^{1}H/^{13}C$ HMBC (600/151 MHz, Chloroform-*d*)

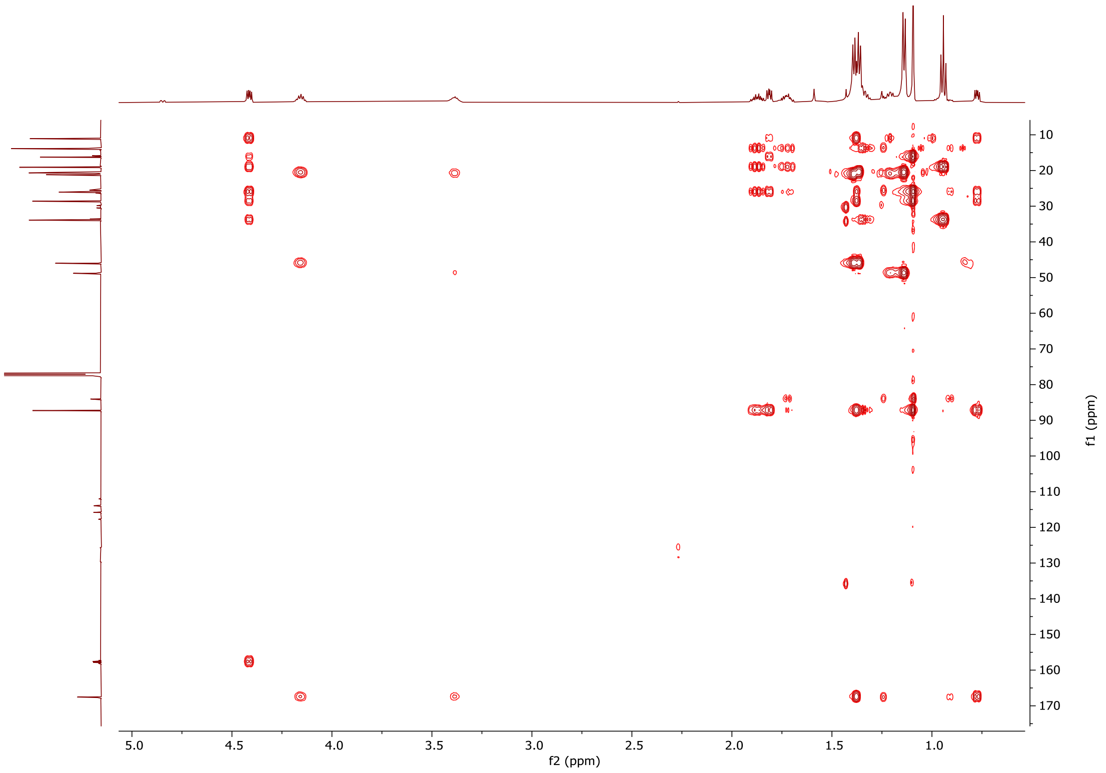

^{1}H NOSEY (600 MHz, Chloroform-*d*)

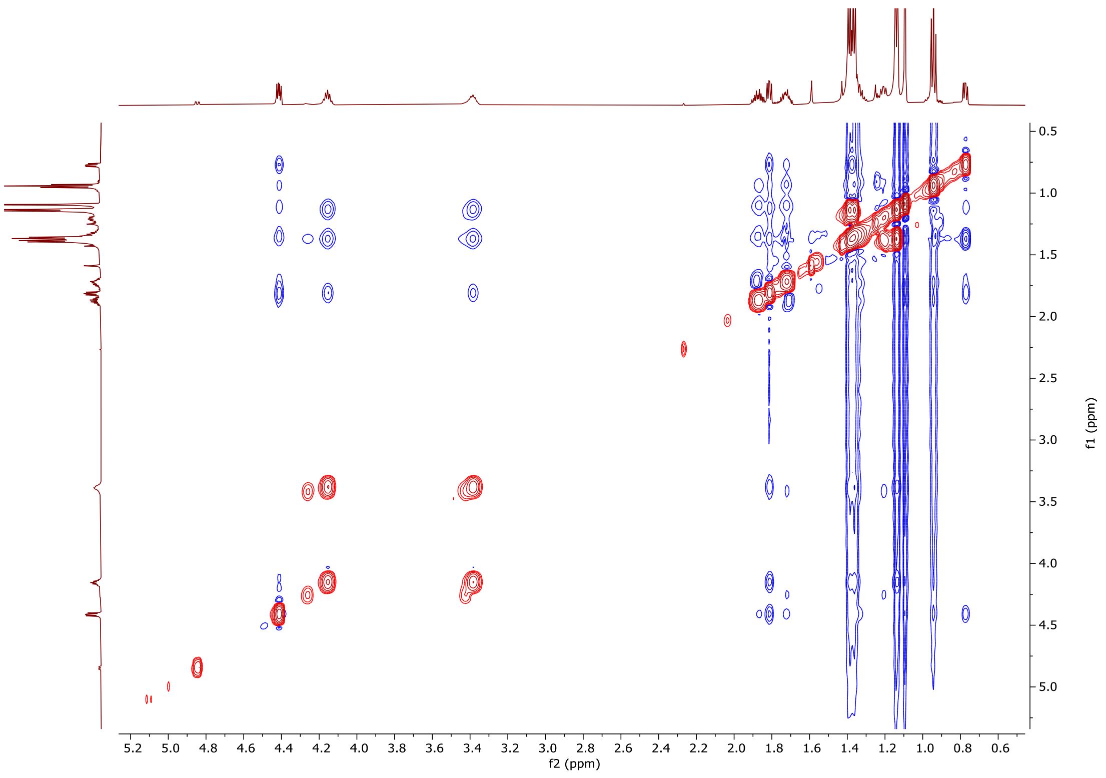

^{19}F NMR (565 MHz, Chloroform-*d*)

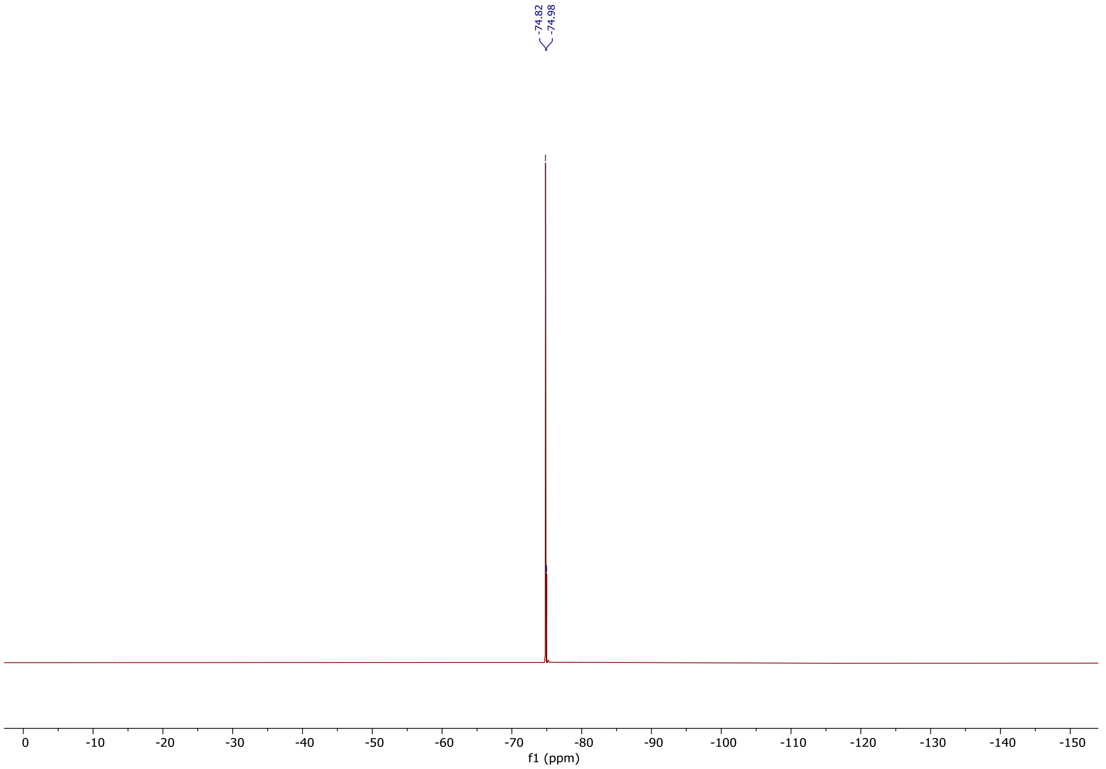

(*S)-1-((1*R**,2*R**)-2-(diisopropylcarbamoyl)-1-methylcyclopropyl)-2-phenylethyl 2,2,2-trifluoroacetate, 3r**

^{1}H NMR (600 MHz, Chloroform-*d*)

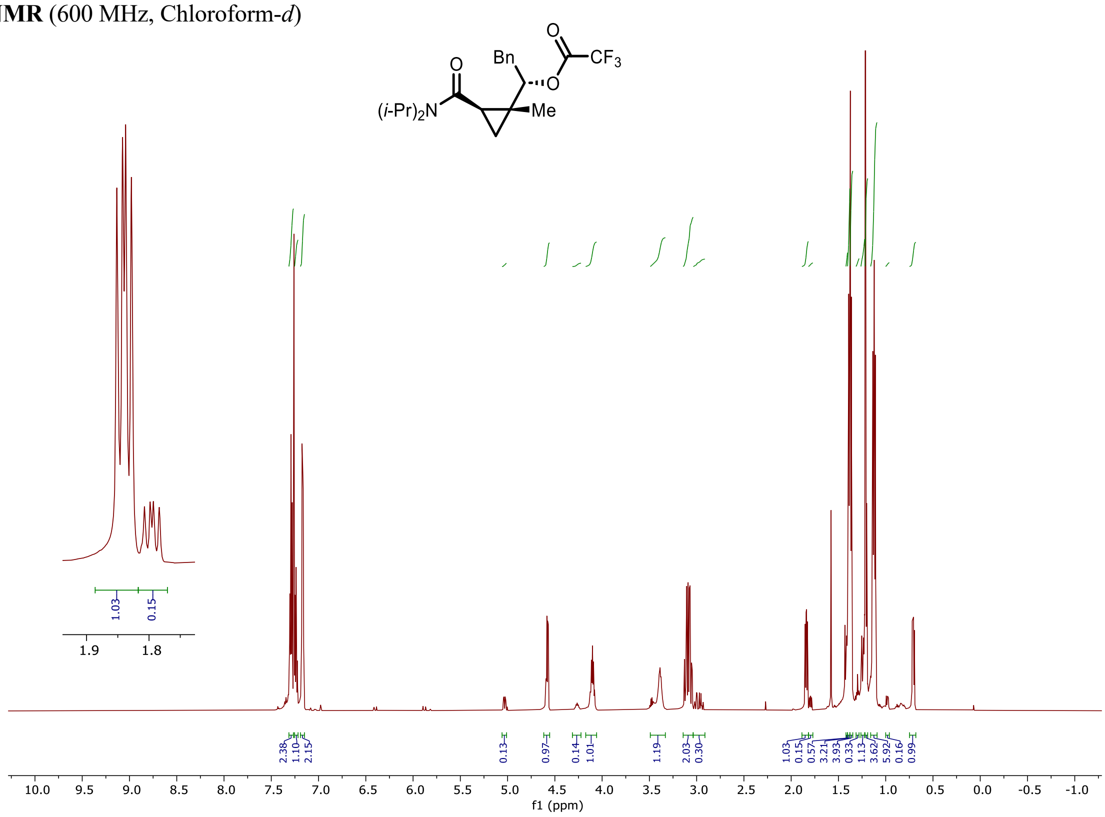

^{13}C NMR (151 MHz, Chloroform-*d*)

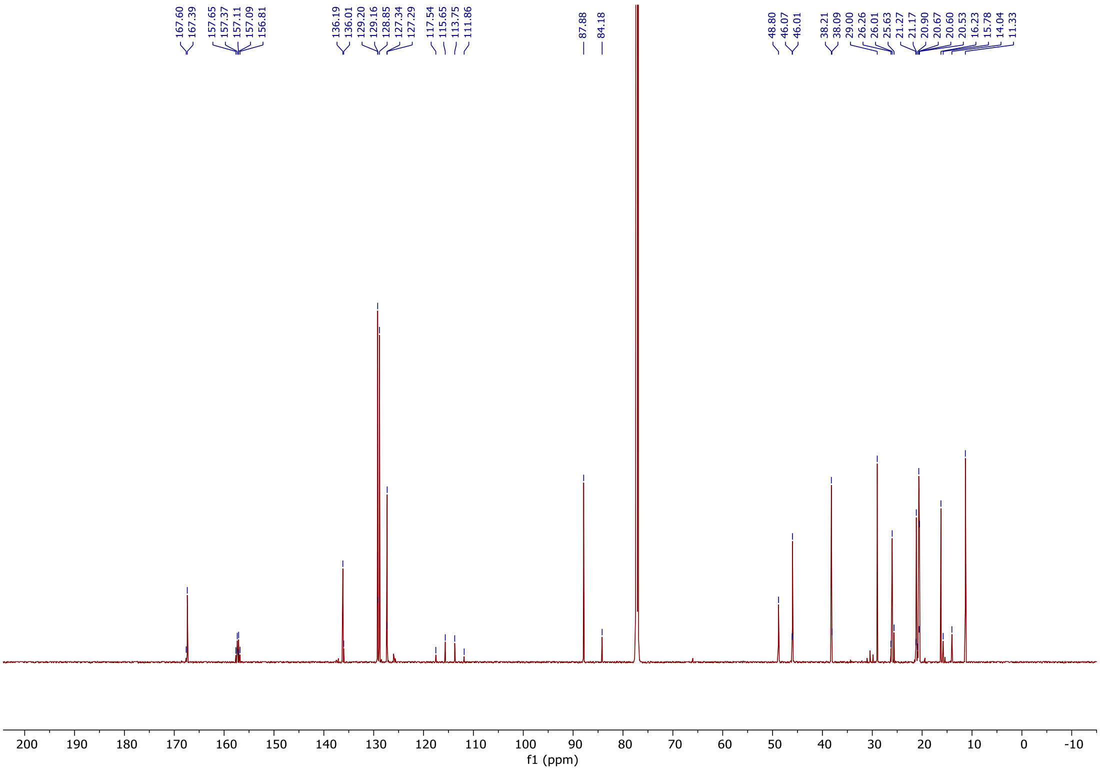

^{1}H COSY (600 MHz, Chloroform-*d*)

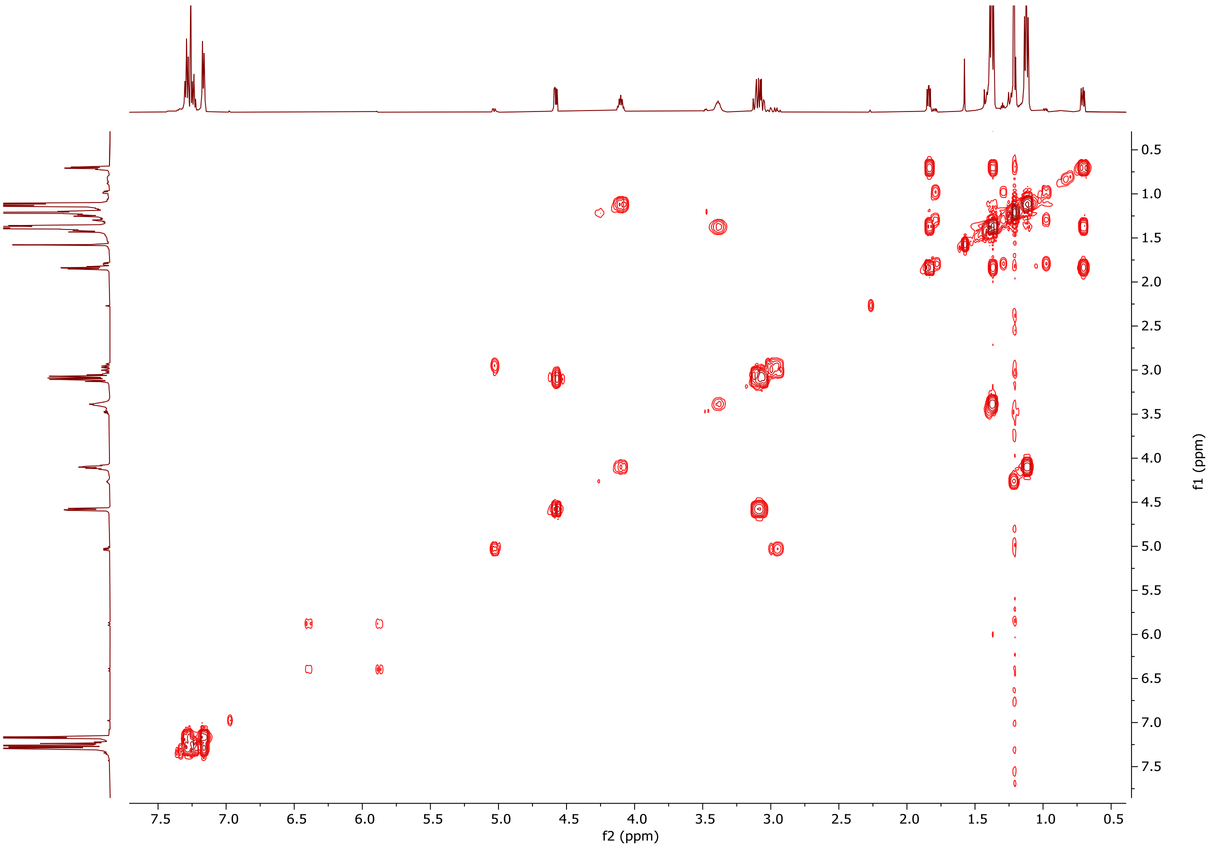

^{1}H/^{13}C HSQC (600/151 MHz, Chloroform-*d*)

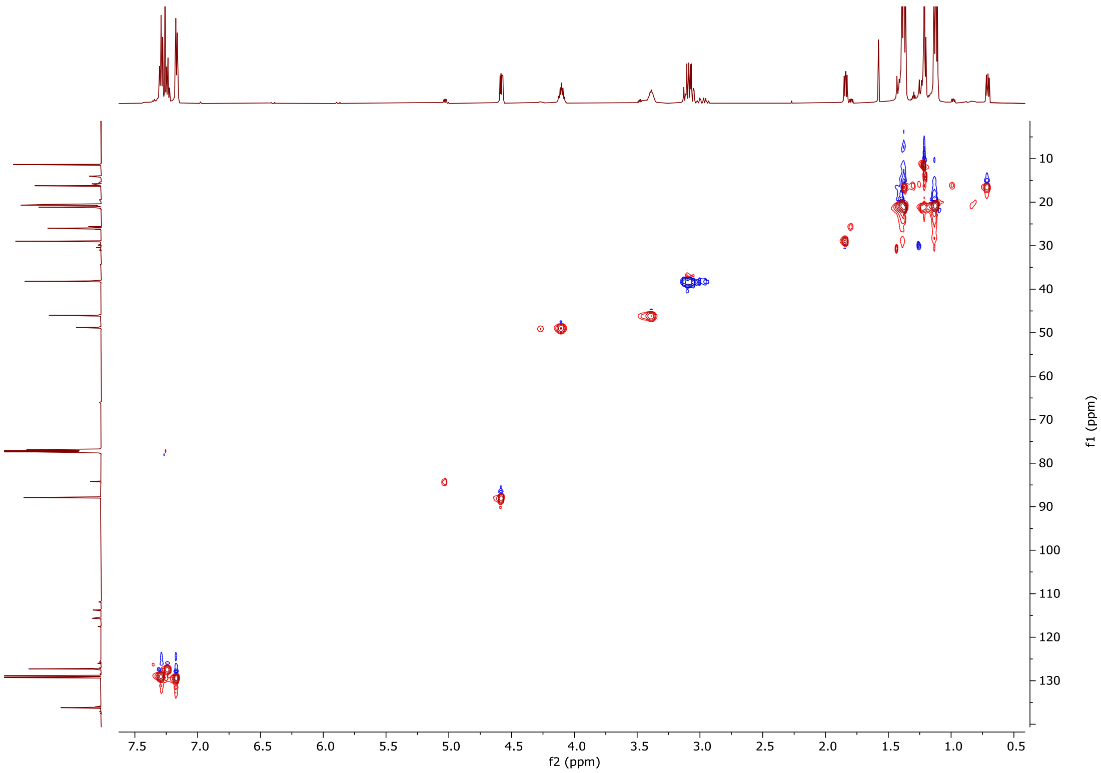

$^{1}H/^{13}C$ HMBC (600/151 MHz, Chloroform-*d*)

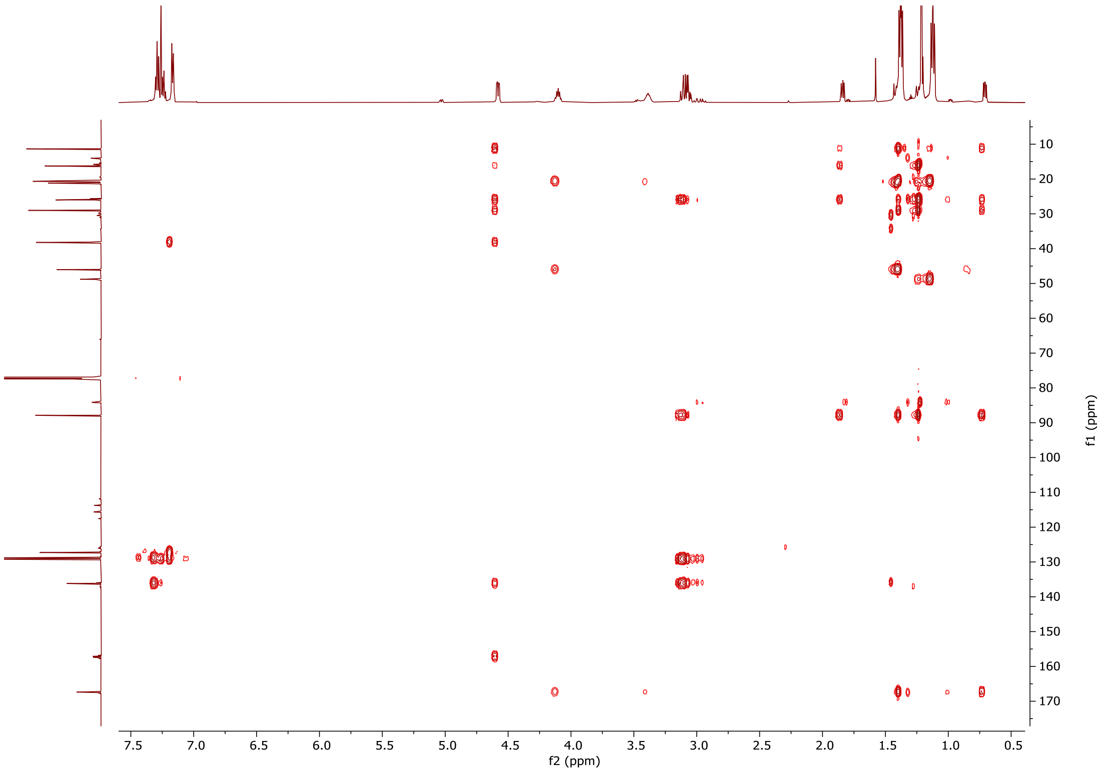

^{1}H NOSEY (600 MHz, Chloroform-*d*)

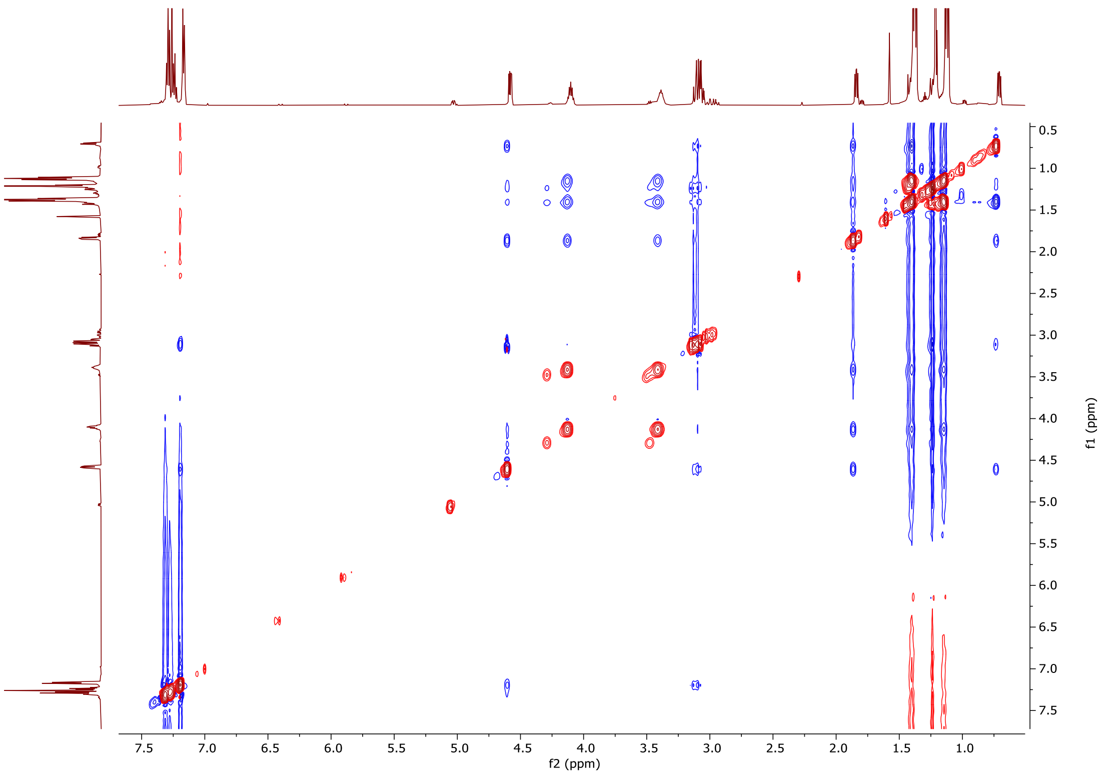

^{19}F NMR (565 MHz, Chloroform-*d*)

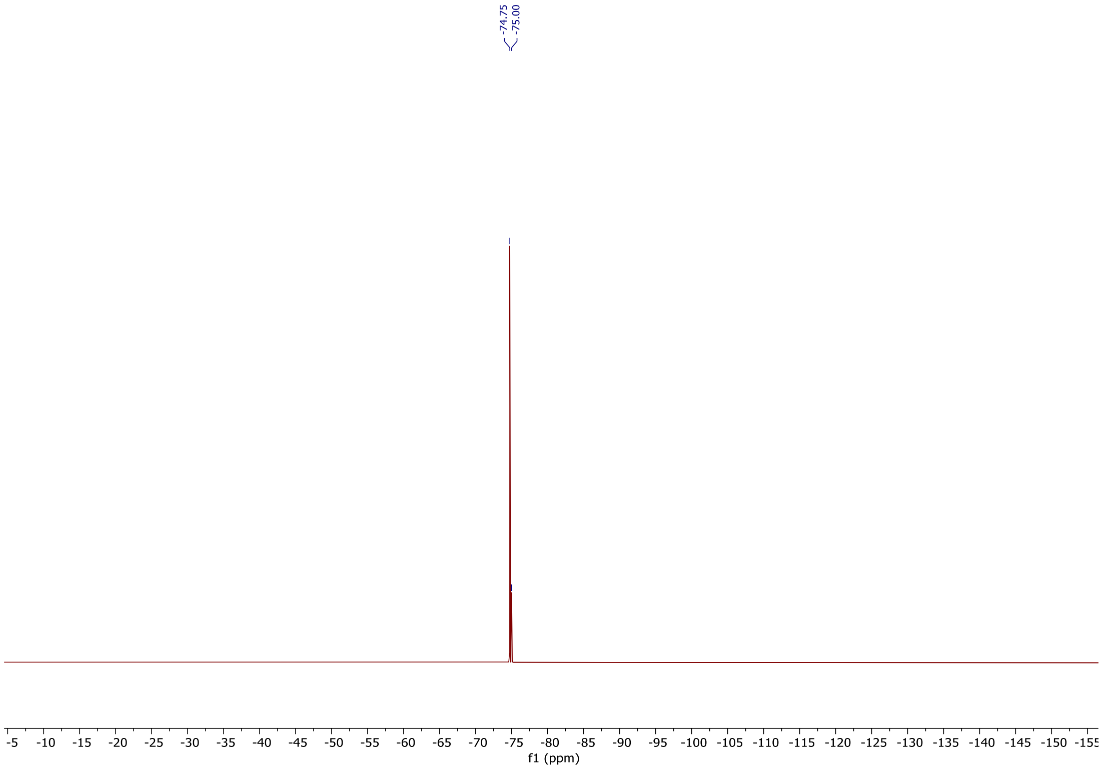

(*R)-((1*R**,2*R**)-2-(diisopropylcarbamoyl)-1-methylcyclopropyl)(phenyl)methyl 2,2,2-trifluoroacetate, 3s**

^{1}H NMR (400 MHz, Chloroform-*d*)

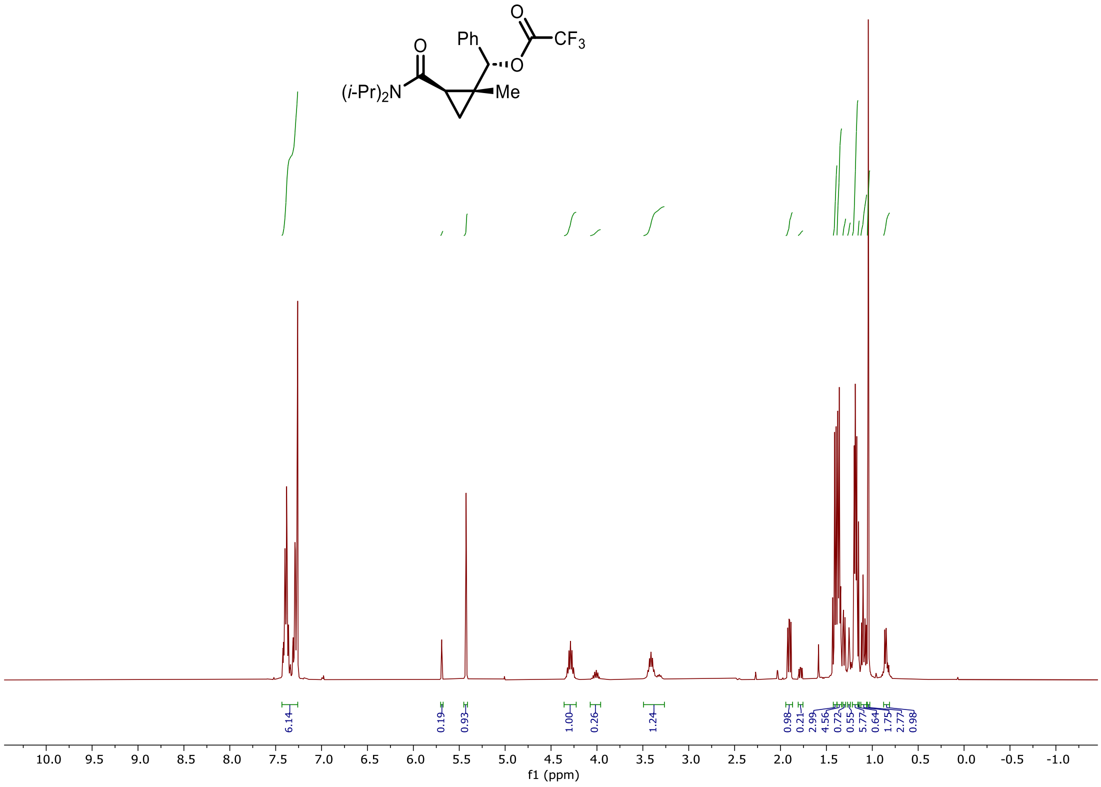

^{13}C NMR (101 MHz, Chloroform-*d*)

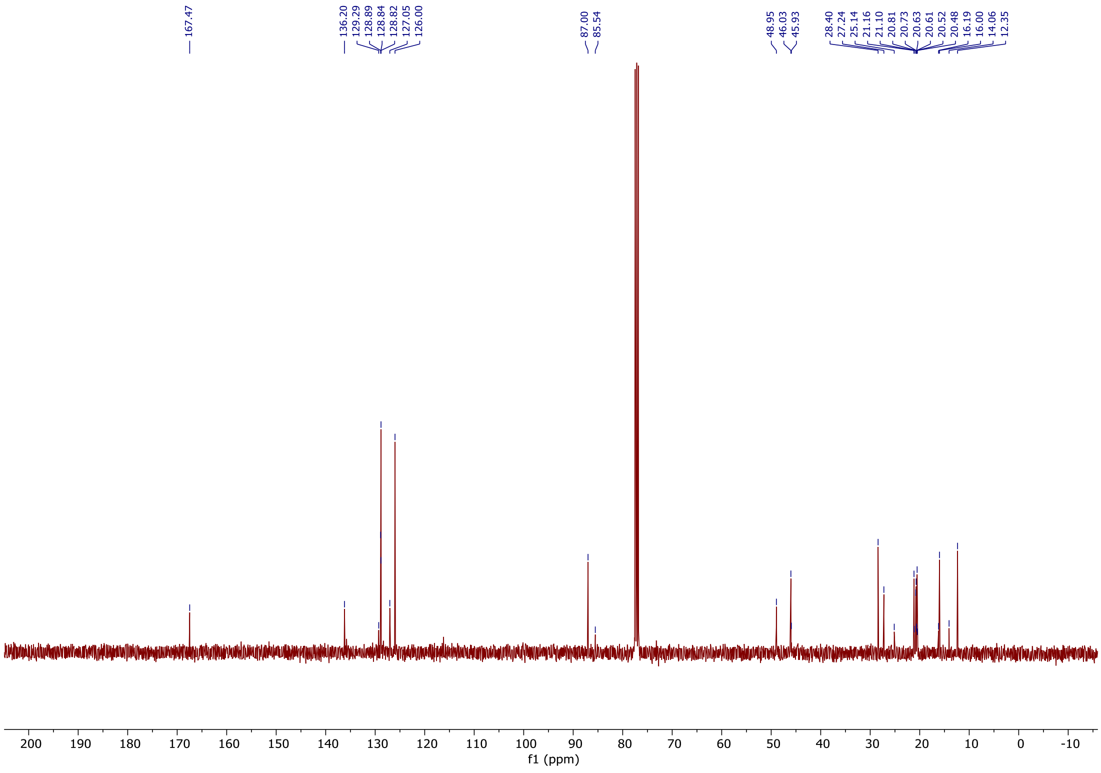

^{1}H COSY (400 MHz, Chloroform-*d*)

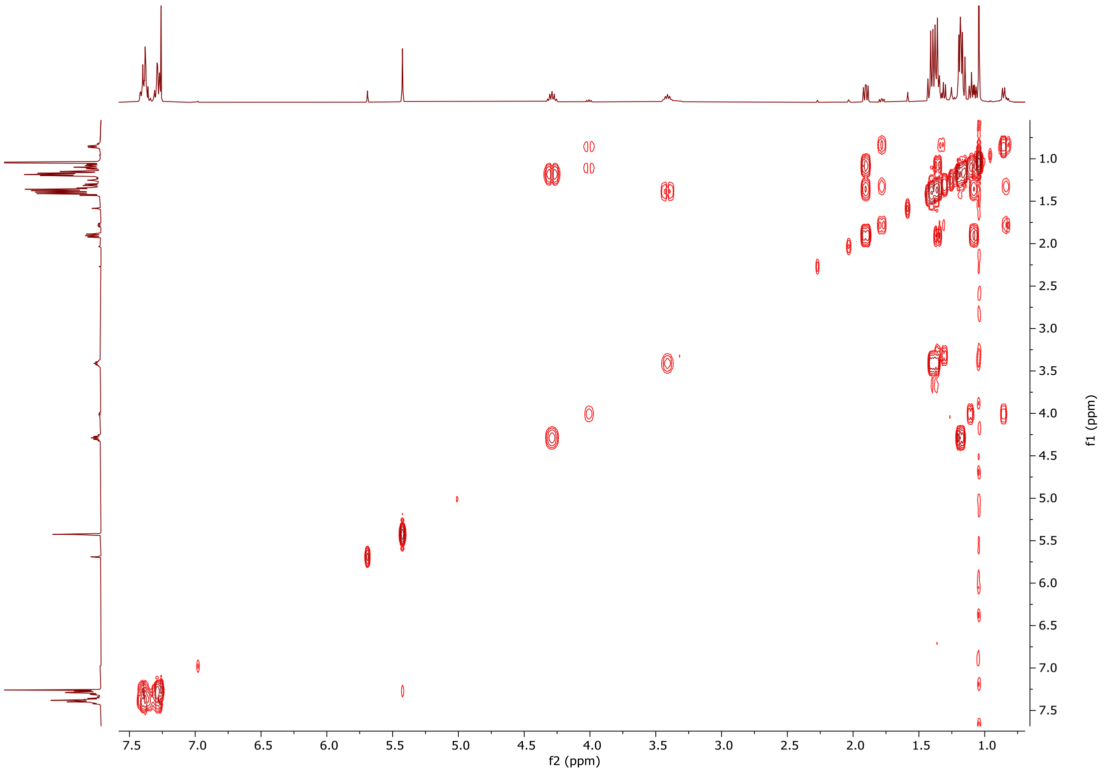

^{1}H/^{13}C HSQC (400/101 MHz, Chloroform-*d*)

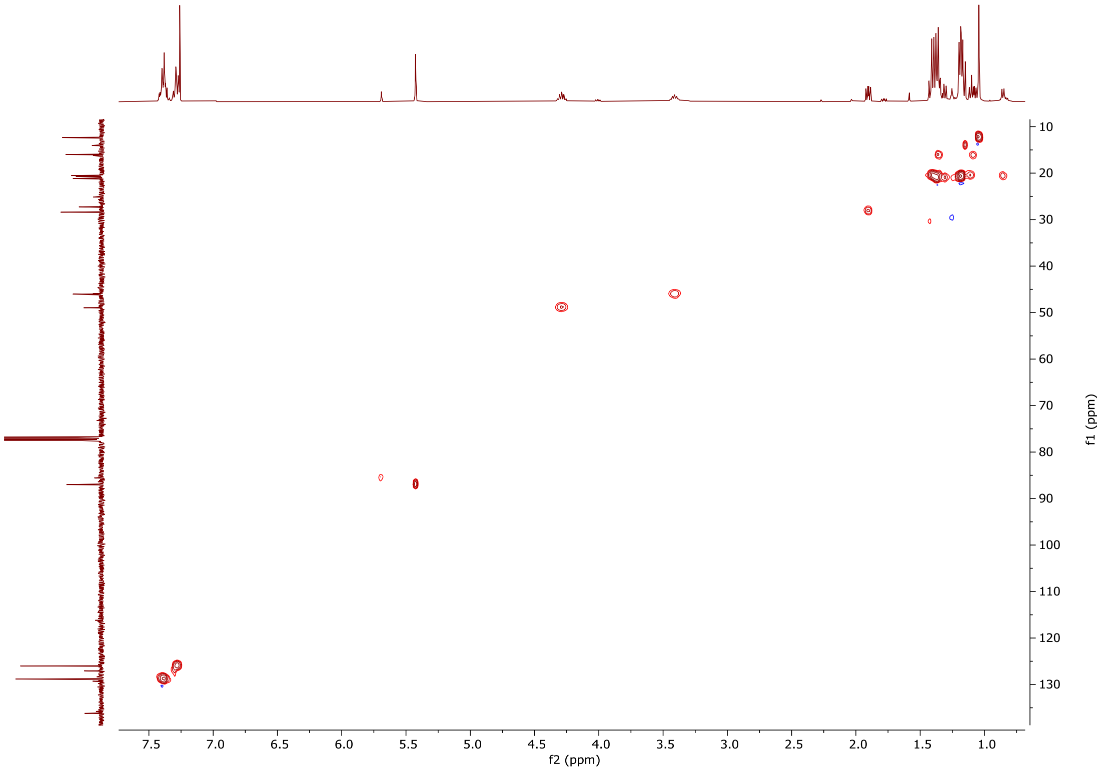

^{19}F NMR (377 MHz, Chloroform-*d*)

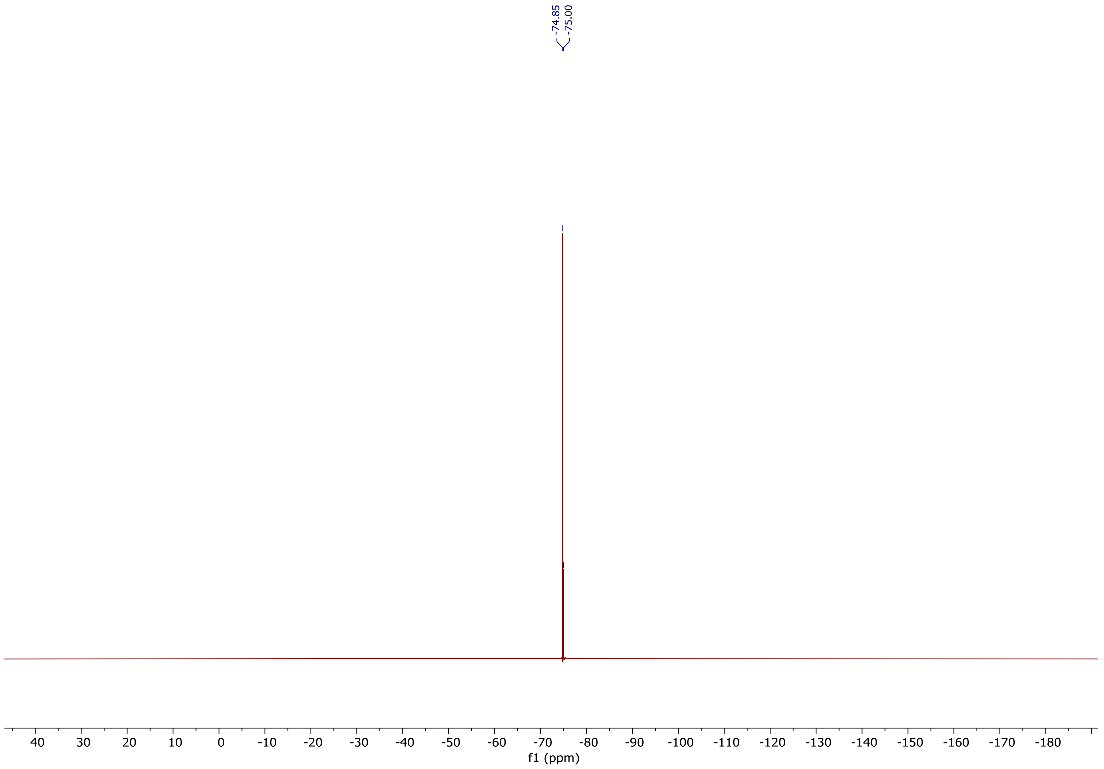

Methyl (1*R,2*R**)-2-((*R**)-phenyl(2,2,2-trifluoroacetoxy)methyl)cyclopropane-1-carboxylate, 3t**

^{1}H NMR (600 MHz, Chloroform-*d*)

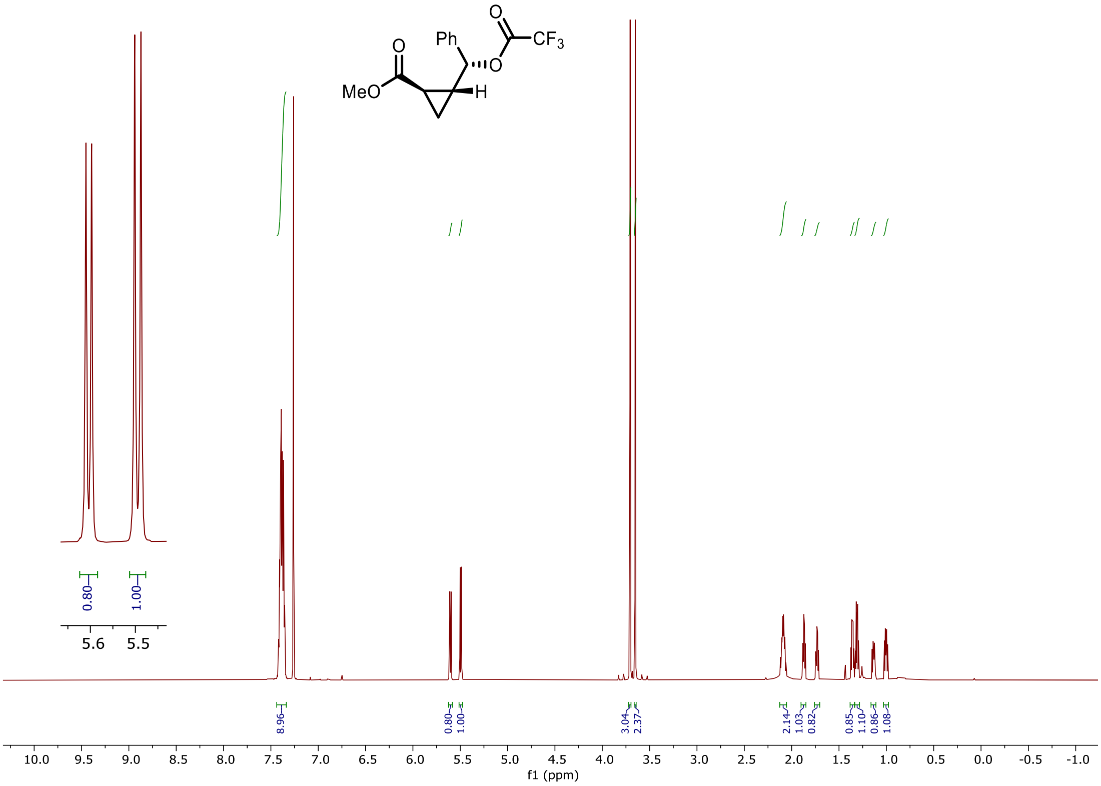

^{13}C NMR (151 MHz, Chloroform-*d*)

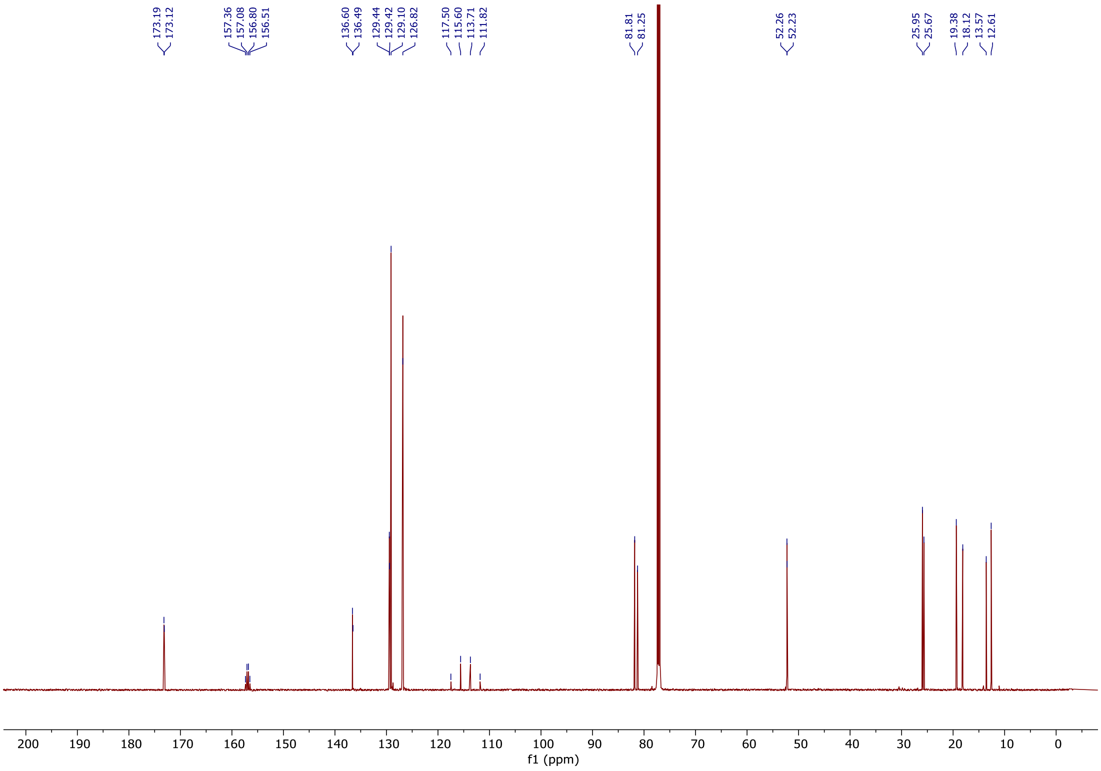

^{1}H COSY (600 MHz, Chloroform-*d*)

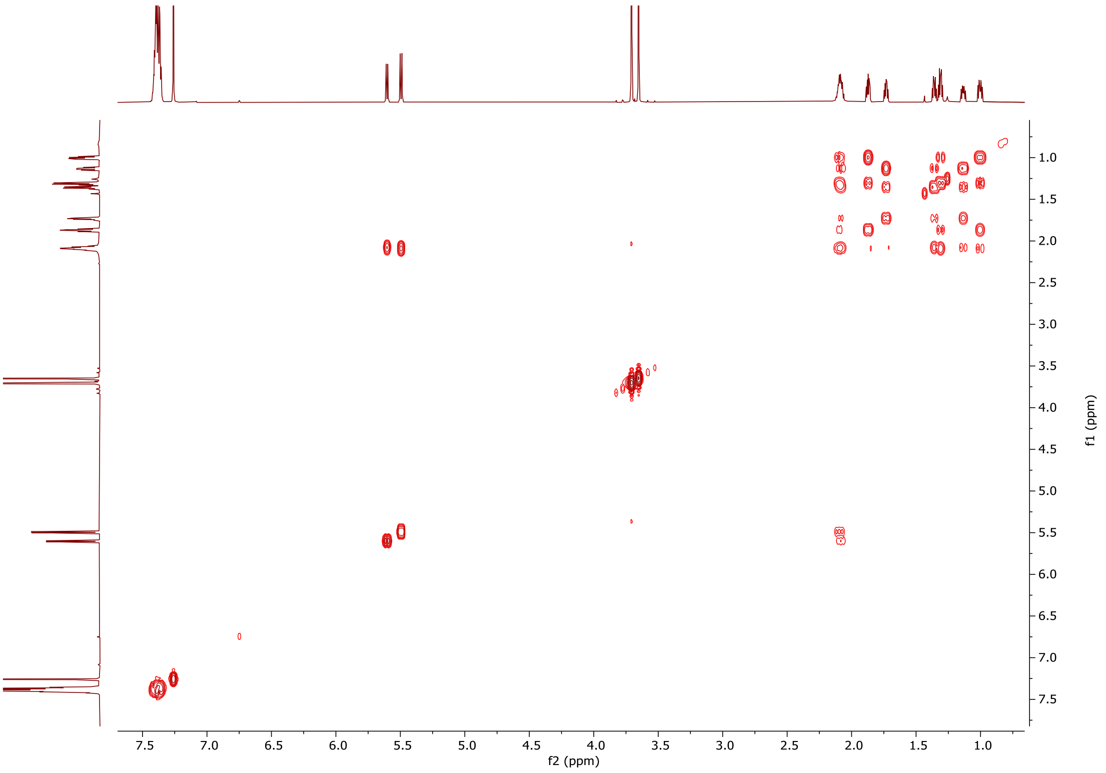

^{1}H/^{13}C HSQC (600/151 MHz, Chloroform-*d*)

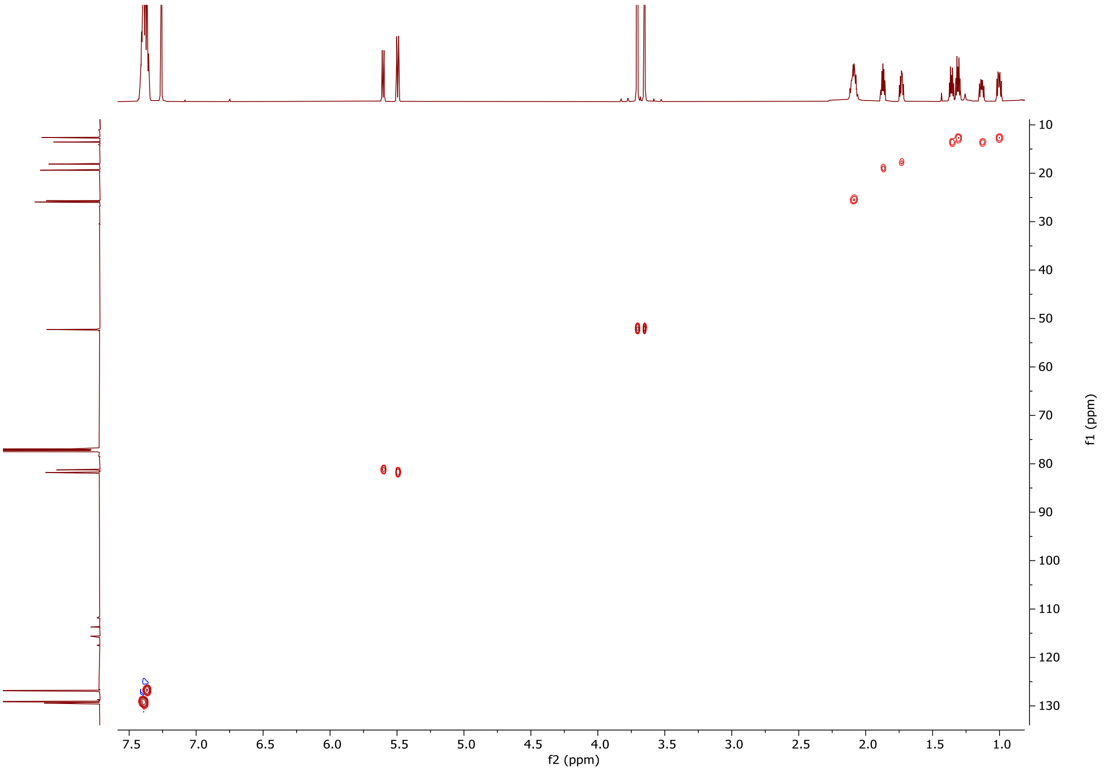

$^1H/^{13}C$ **HMBC** (600/151 MHz, Chloroform-*d*)

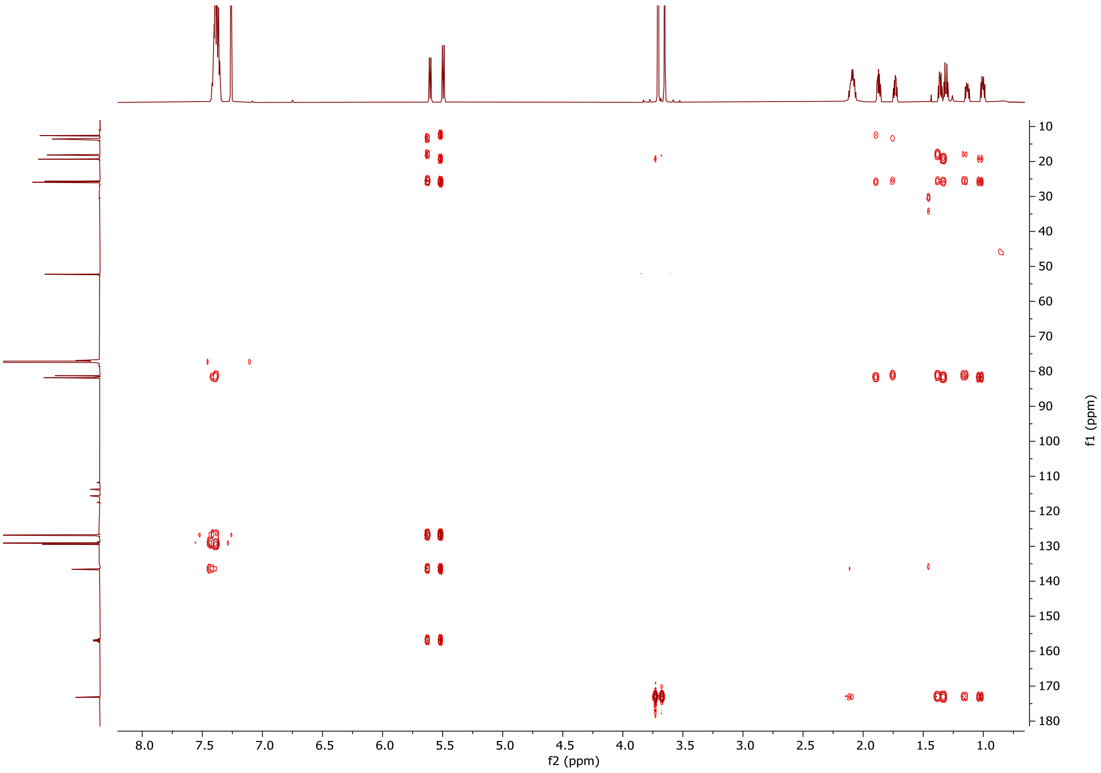

^1H **NOSEY** (600 MHz, Chloroform-*d*)

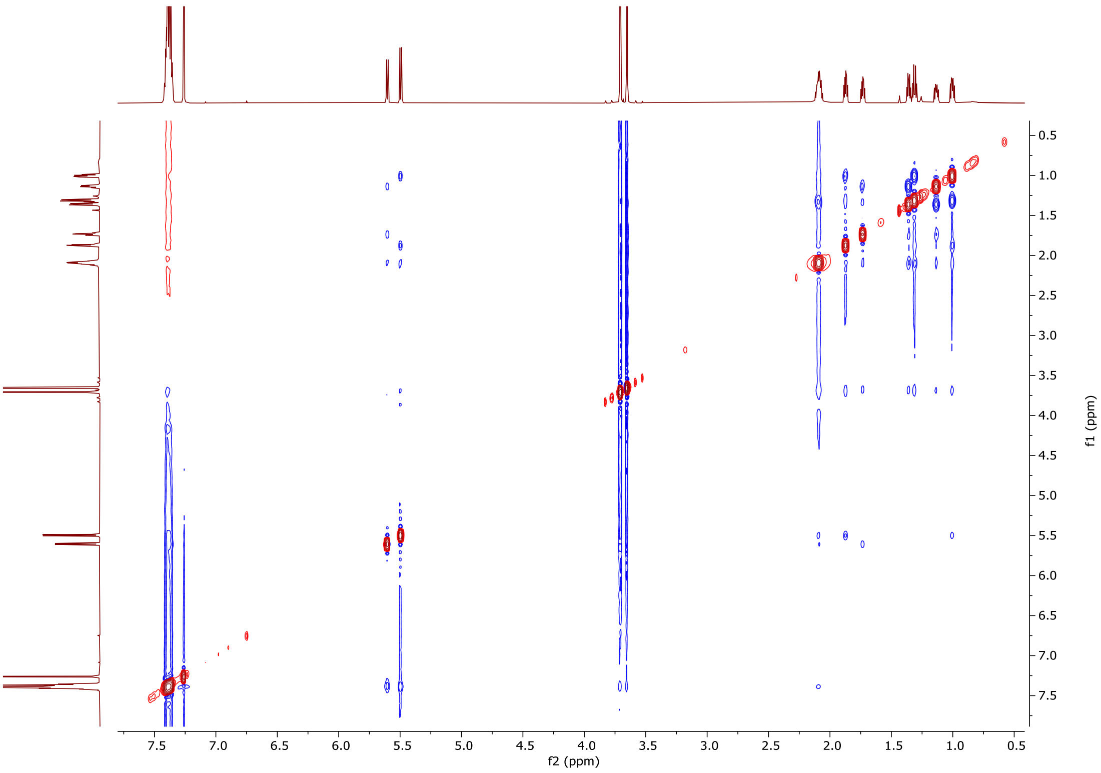

^{19}F NMR (565 MHz, Chloroform-*d*)

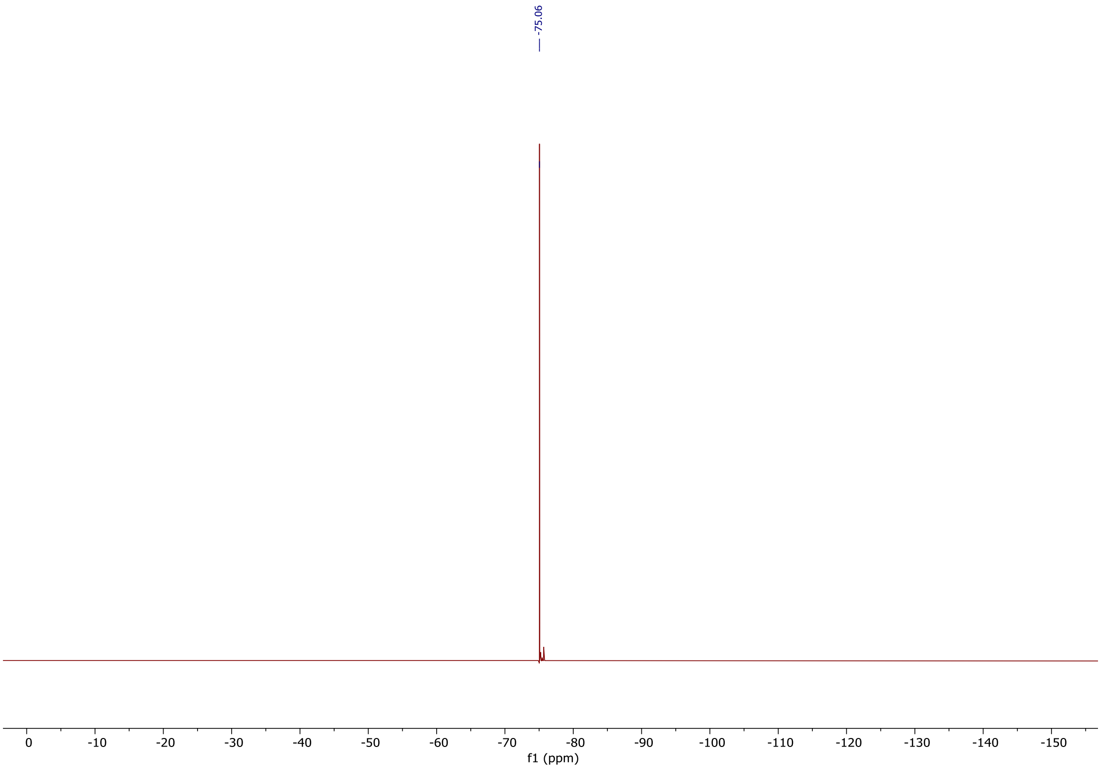

((1*S,2*S**)-2-(diisopropylcarbamoyl)-1-(trimethylsilyl)cyclopropyl)(trimethylsilyl)methyl 2,2,2-trifluoroacetate, 3u**

^{1}H NMR (600 MHz, Chloroform-*d*)

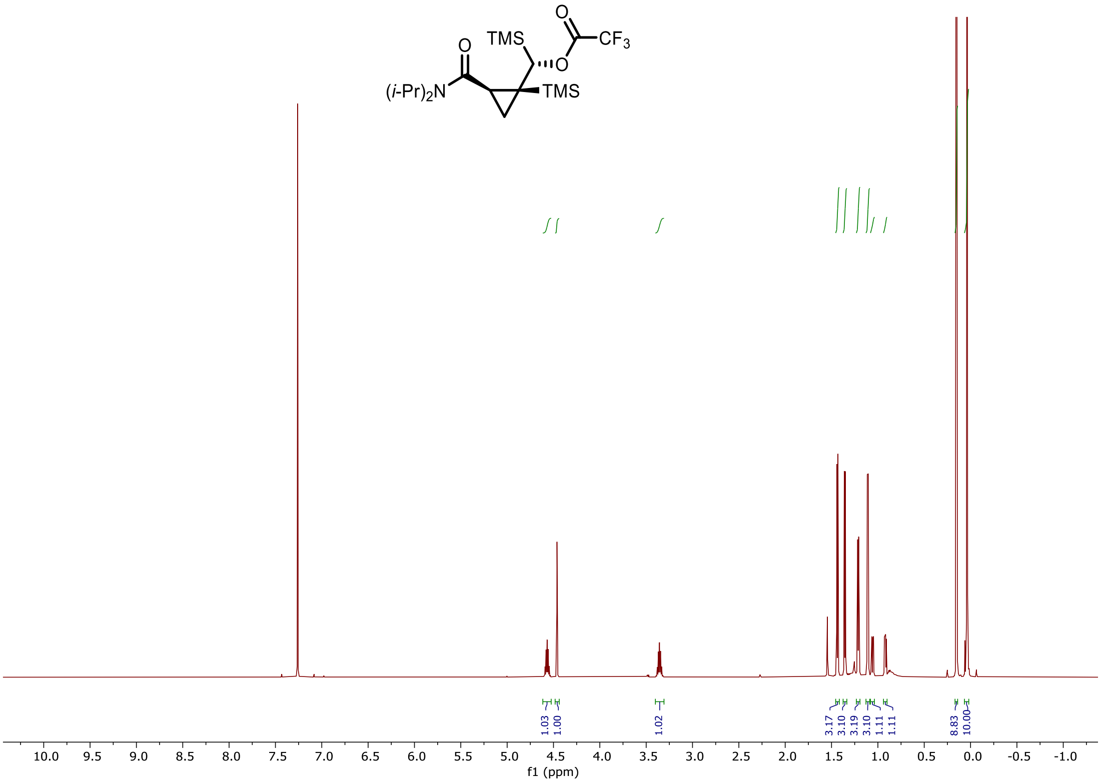

^{13}C NMR (151 MHz, Chloroform-*d*)

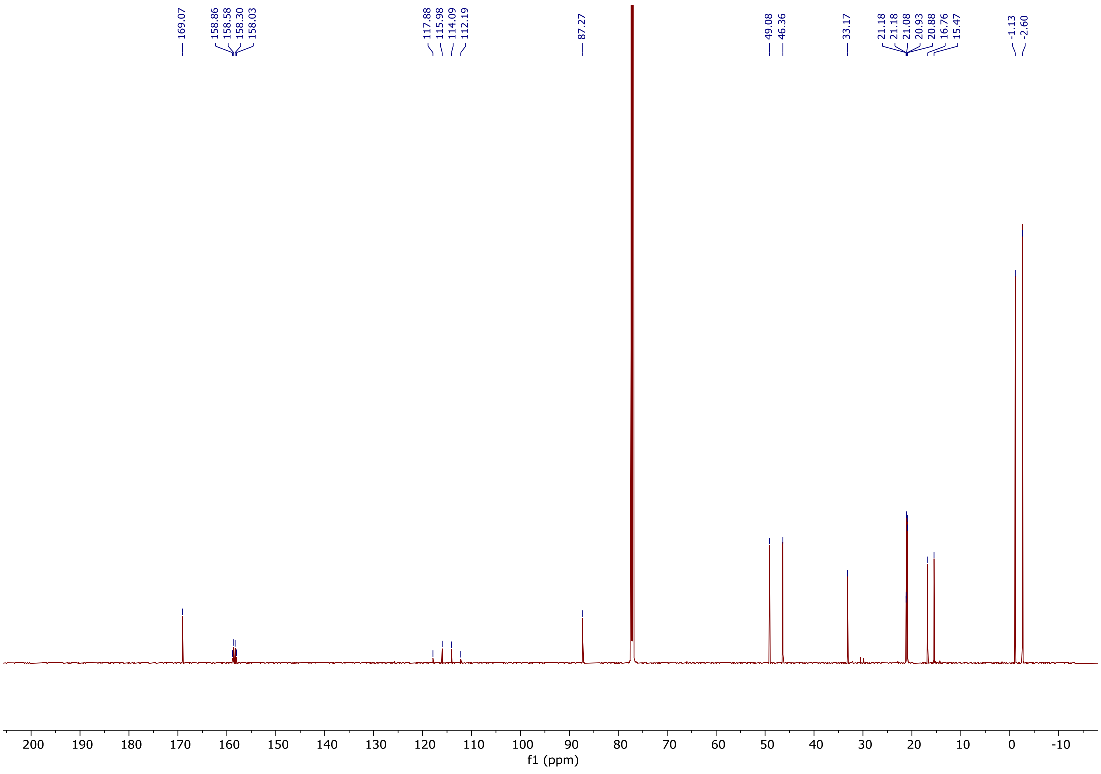

^{1}H COSY (600 MHz, Chloroform-*d*)

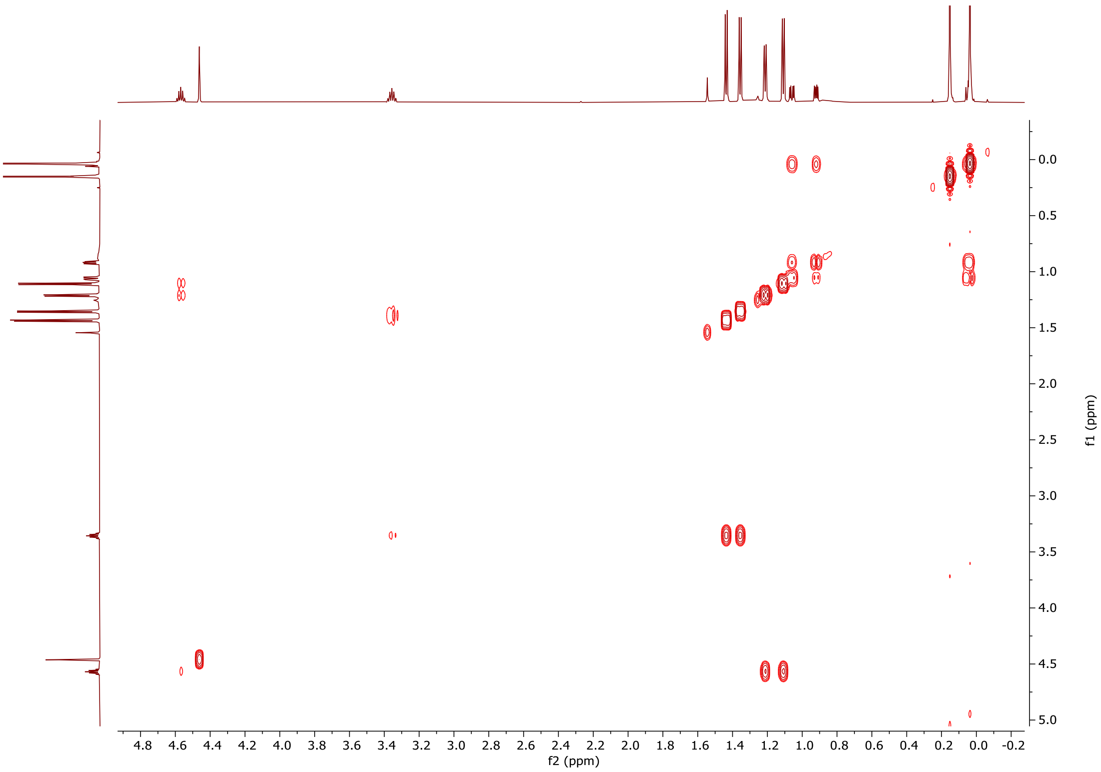

^{1}H/^{13}C HSQC (600/151 MHz, Chloroform-*d*)

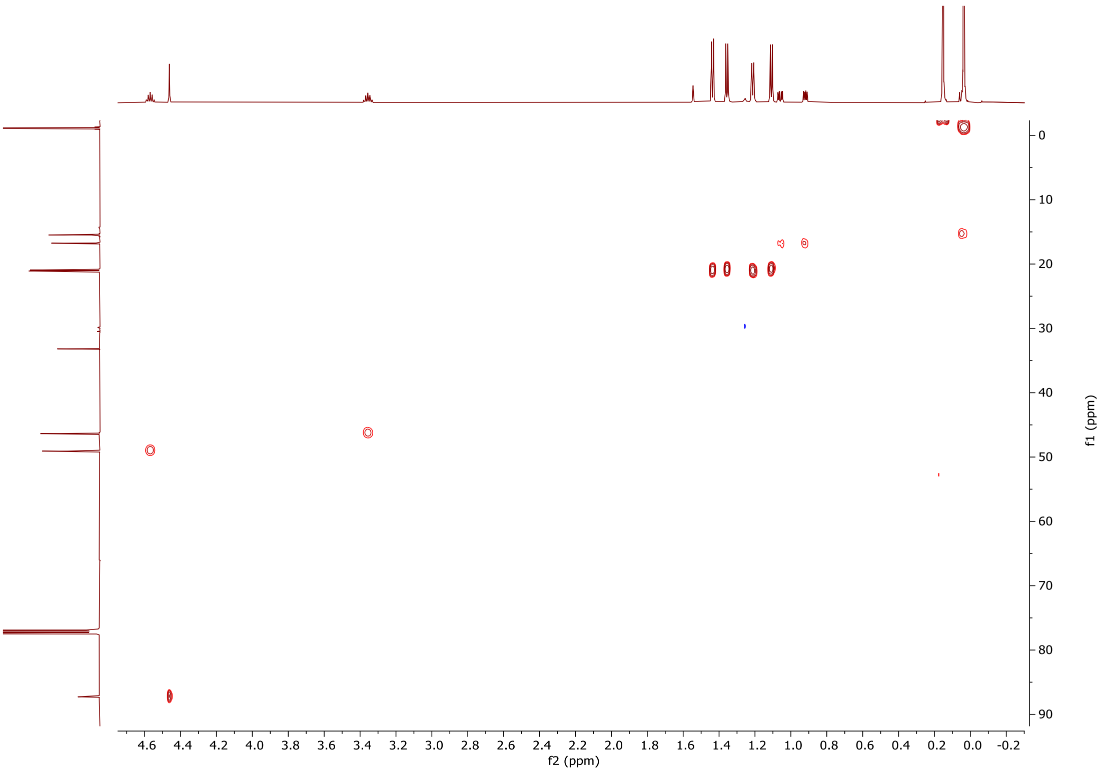

^{1}H/^{13}C HMBC (600/151 MHz, Chloroform-*d*)

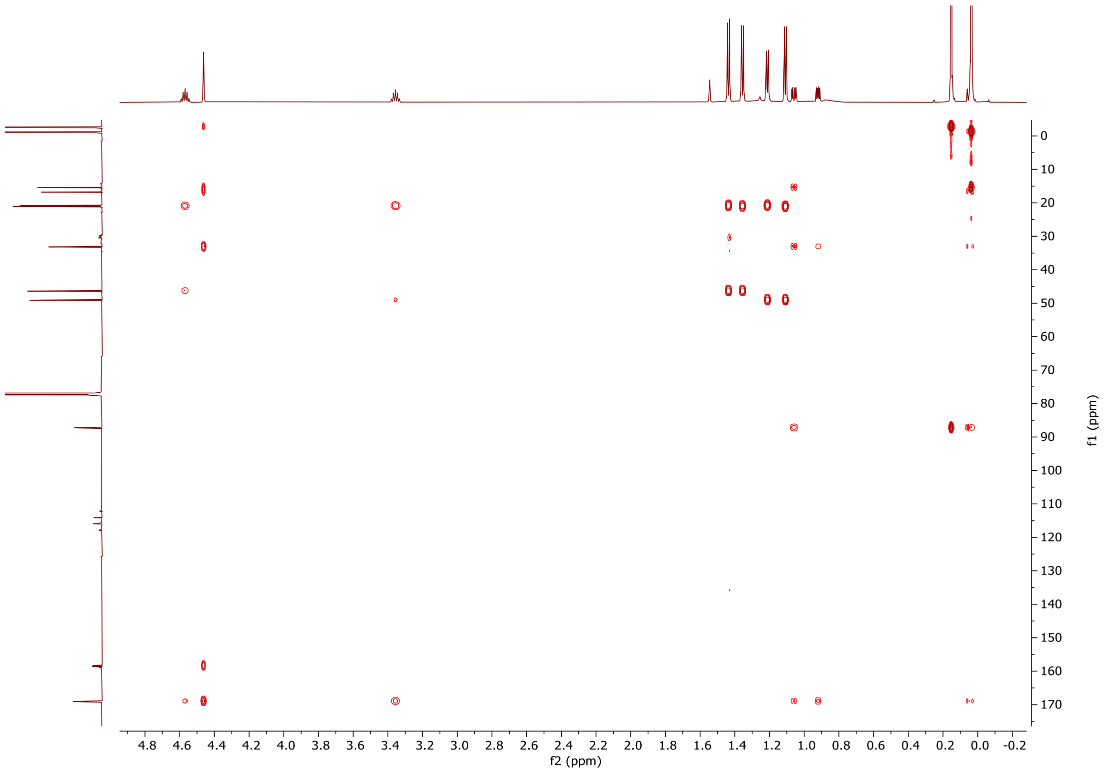

^{1}H NOSEY (600 MHz, Chloroform-*d*)

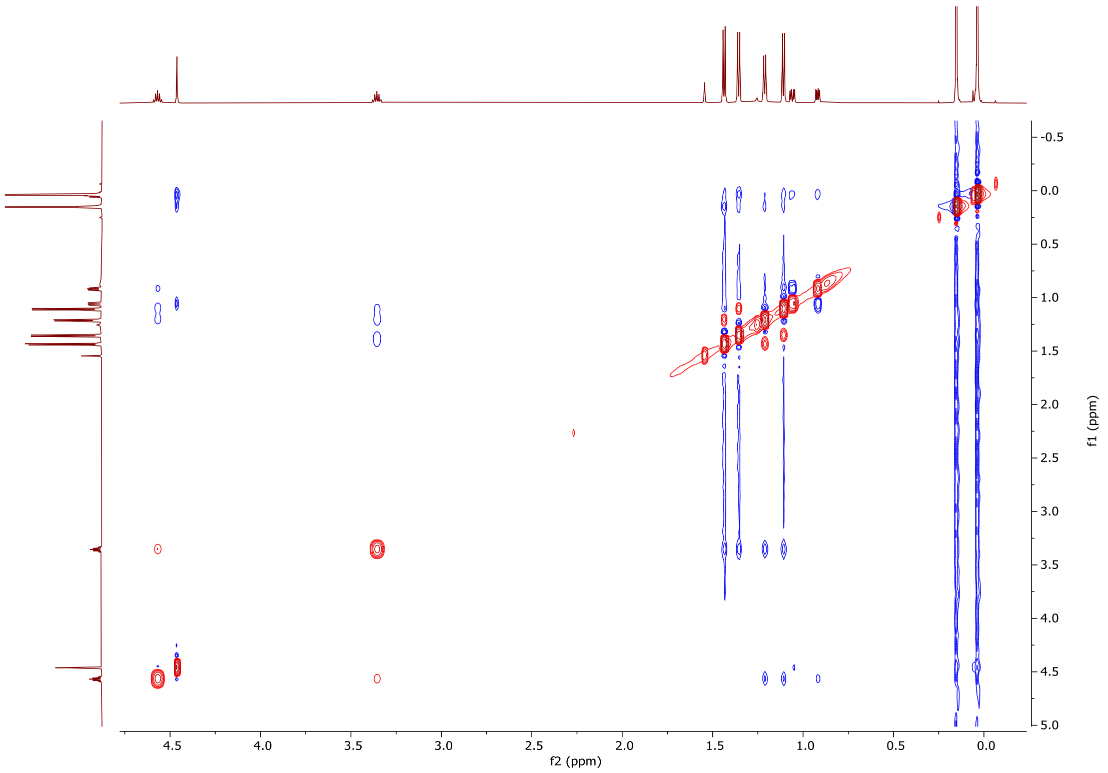

^{19}**F NMR** (565 MHz, Chloroform-*d*)

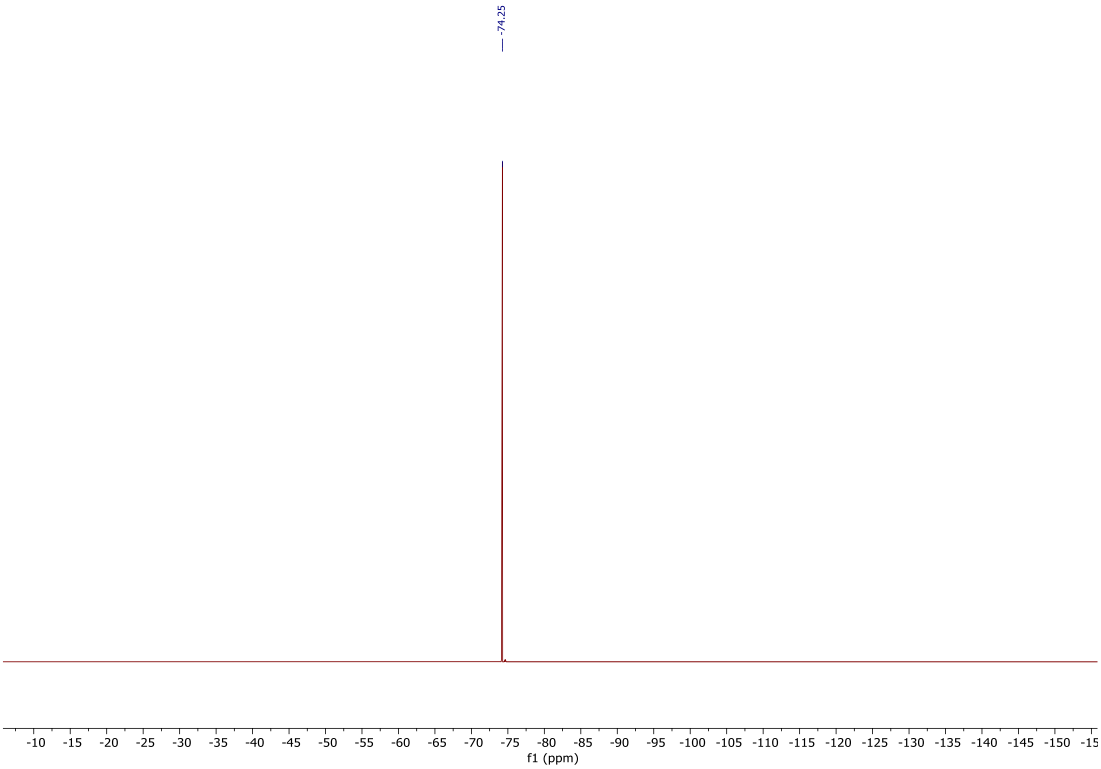

***O*-((*S**)-1-((1*R**,2*R**)-2-(diisopropylcarbamoyl)-1-methylcyclopropyl)ethyl) ethanethioate, 3v**

***S*-((1*R**,2*R**,3*S**)-3-(diisopropylcarbamoyl)-1,2-dimethylcyclobutyl) ethanethioate, S4**

[1]**H NMR** (600 MHz, Chloroform-*d*)

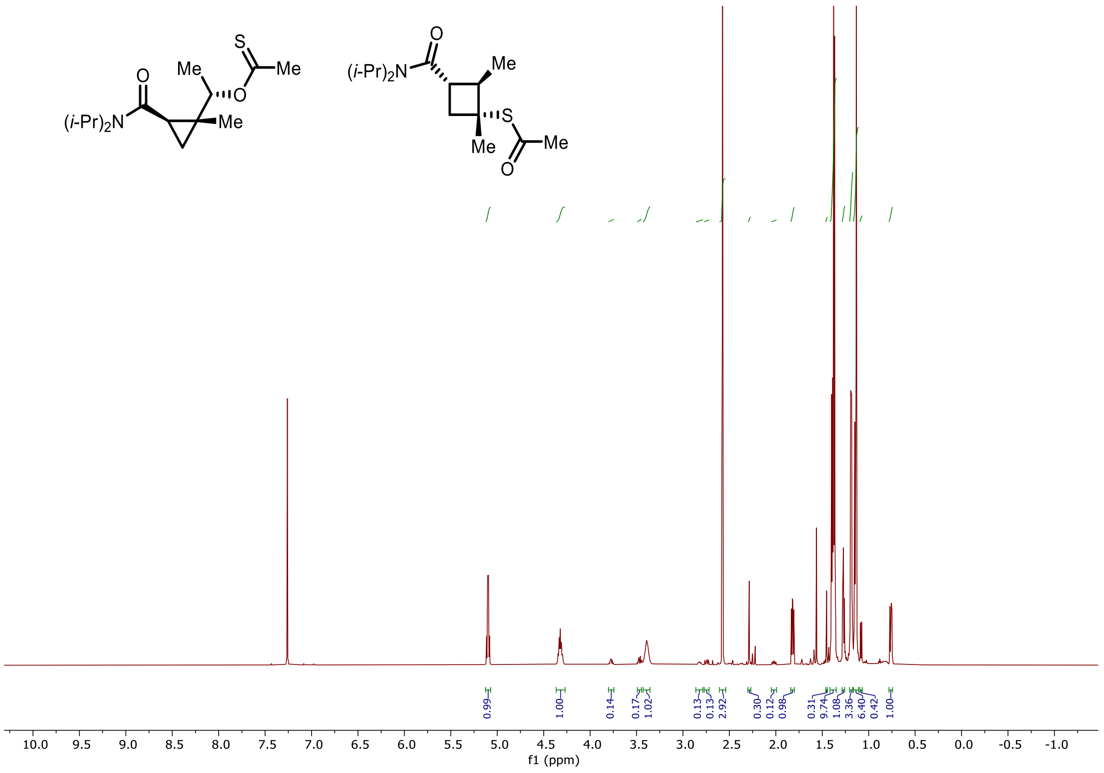

[13]**C NMR** (151 MHz, Chloroform-*d*)

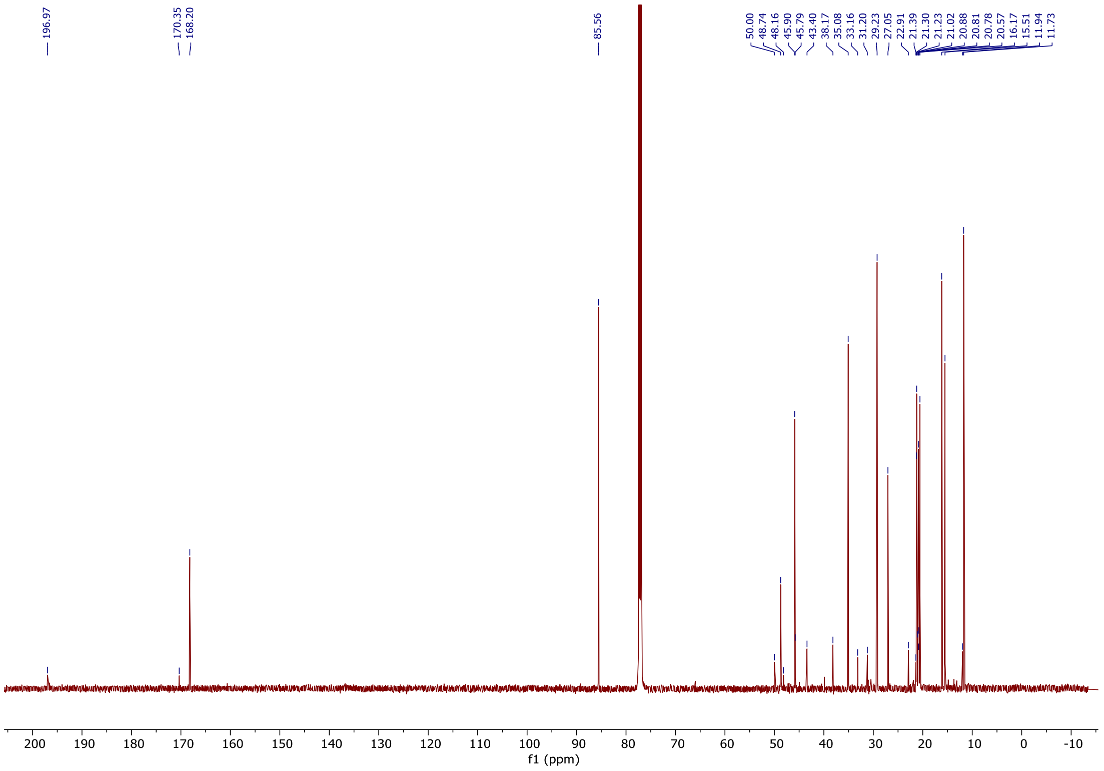

^{1}H COSY (600 MHz, Chloroform-*d*)

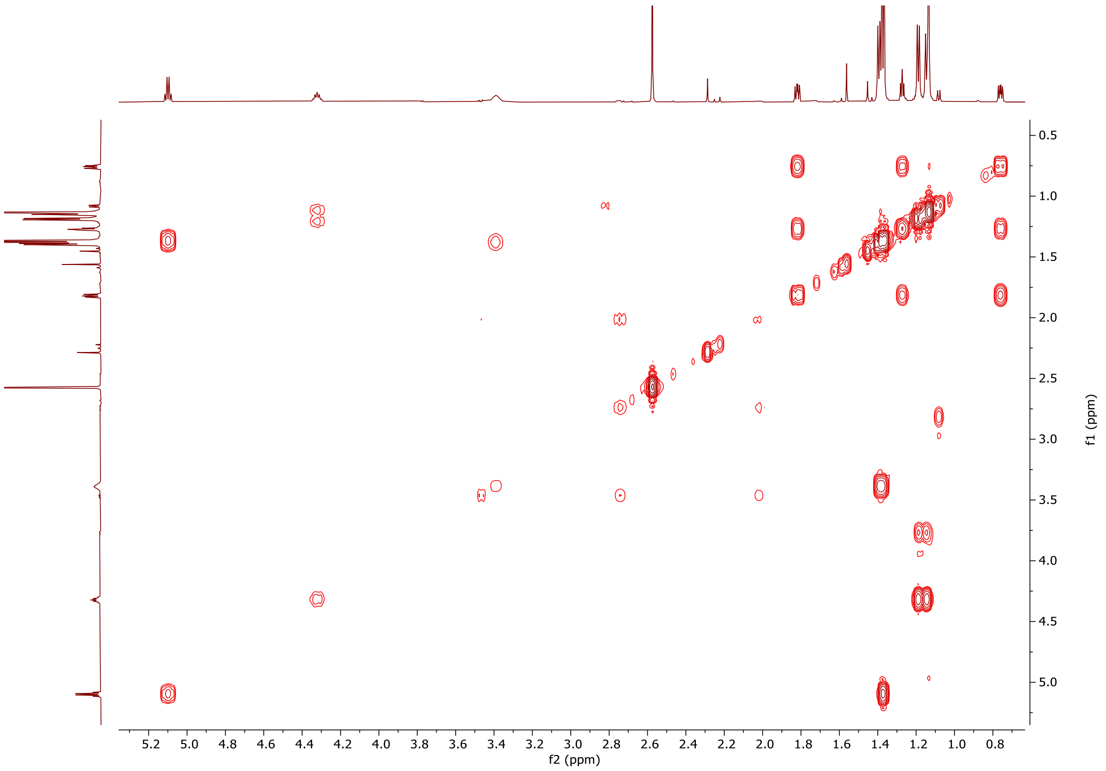

^{1}H/^{13}C HSQC (600/151 MHz, Chloroform-*d*)

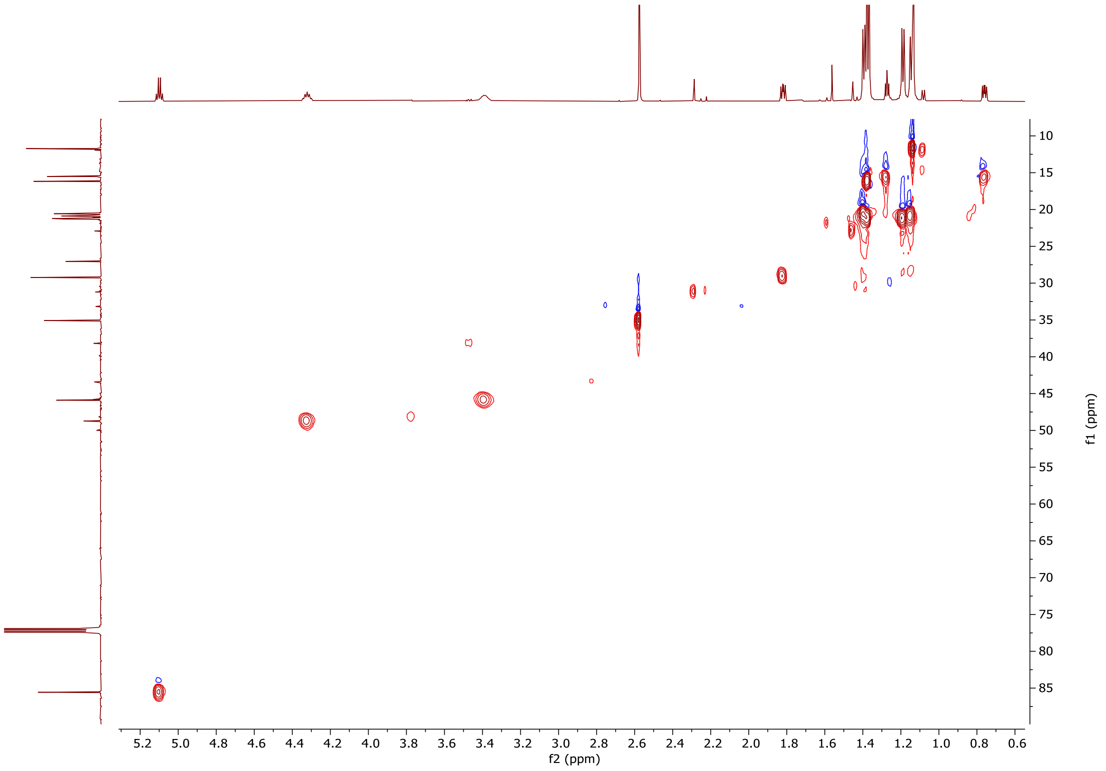

^{1}H/^{13}C HMBC (600/151 MHz, Chloroform-*d*)

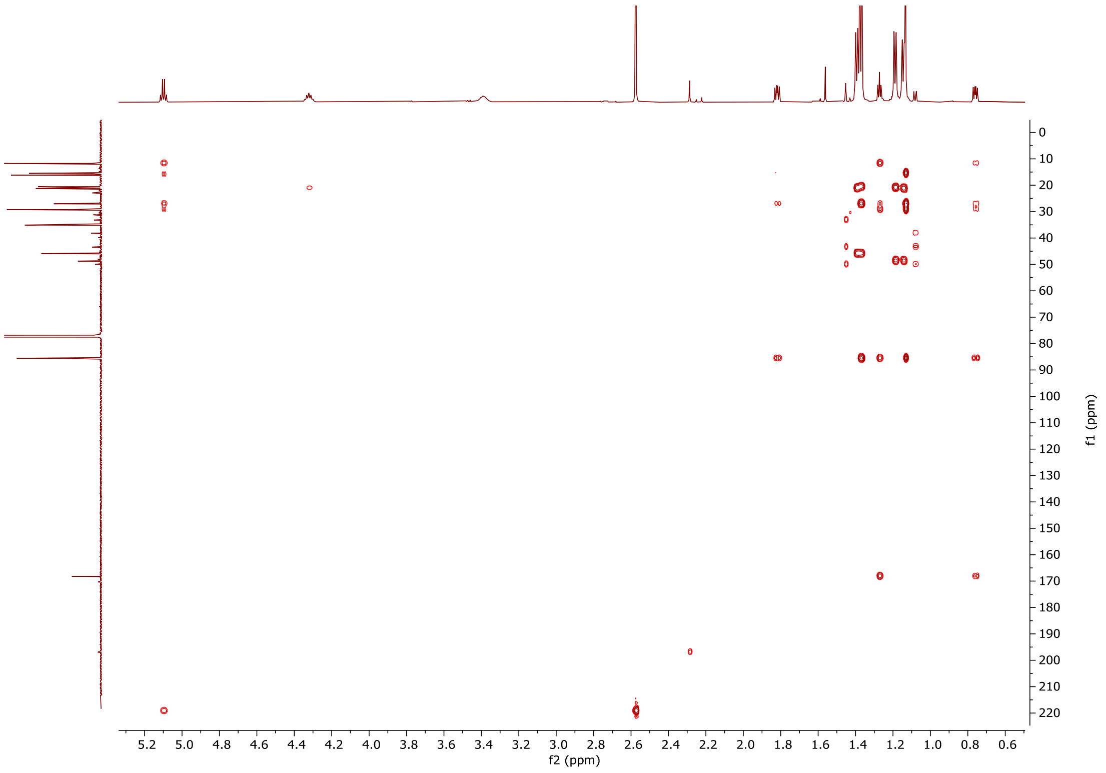

^{1}H NOSEY (600 MHz, Chloroform-*d*)

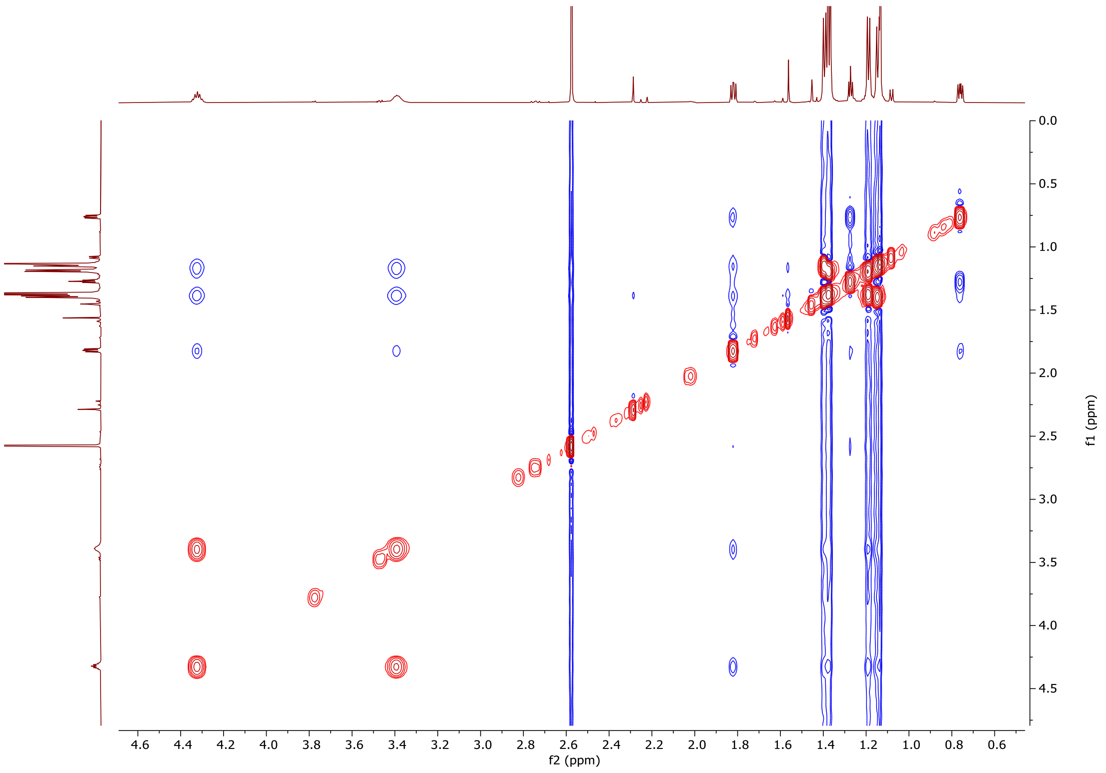

***S*-((*S**)-1-((1*R**,2*R**)-2-(diisopropylcarbamoyl)-1-methylcyclopropyl)ethyl) ethanethioate, 3w**

[1]**H NMR** (600 MHz, Chloroform-*d*)

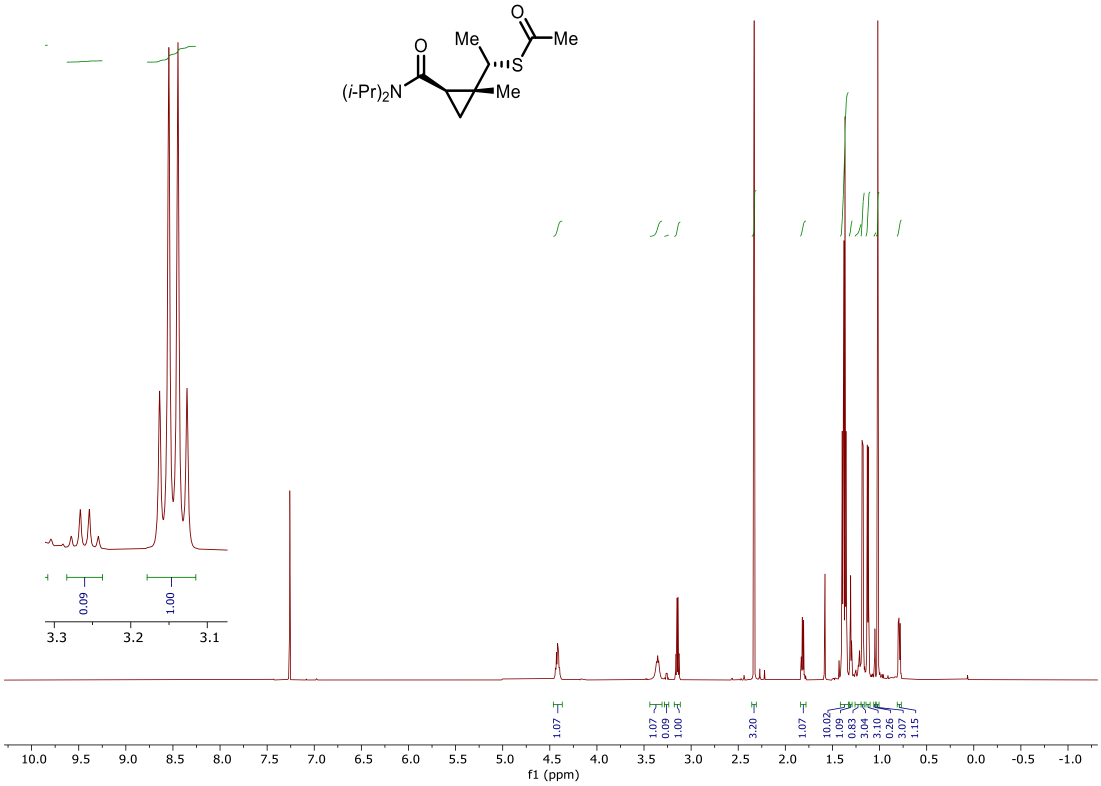

[13]**C NMR** (151 MHz, Chloroform-*d*)

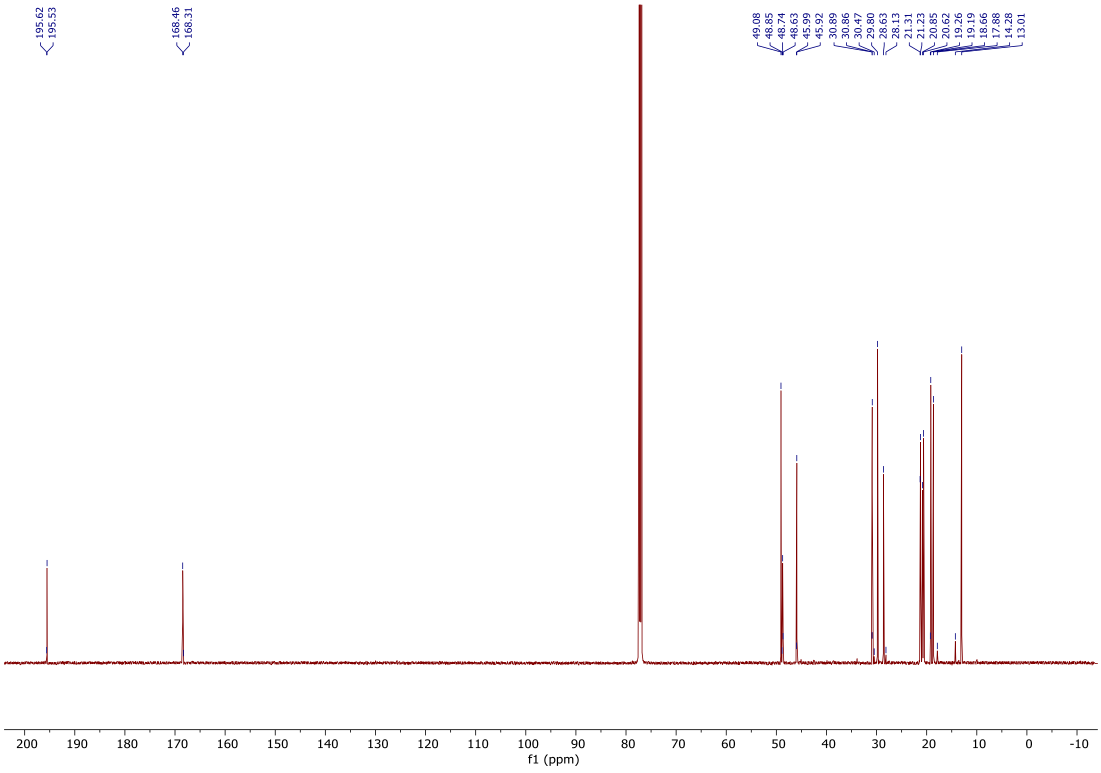

^{1}H COSY (600 MHz, Chloroform-*d*)

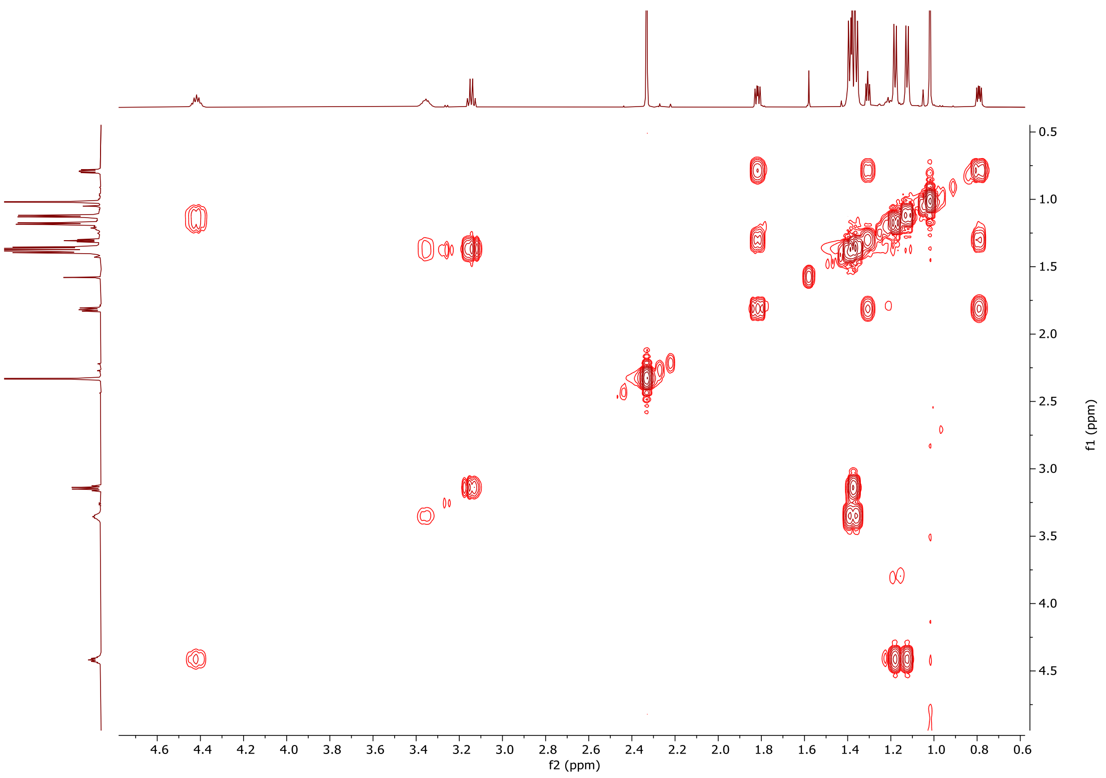

$^{1}H/^{13}C$ HSQC (600/151 MHz, Chloroform-*d*)

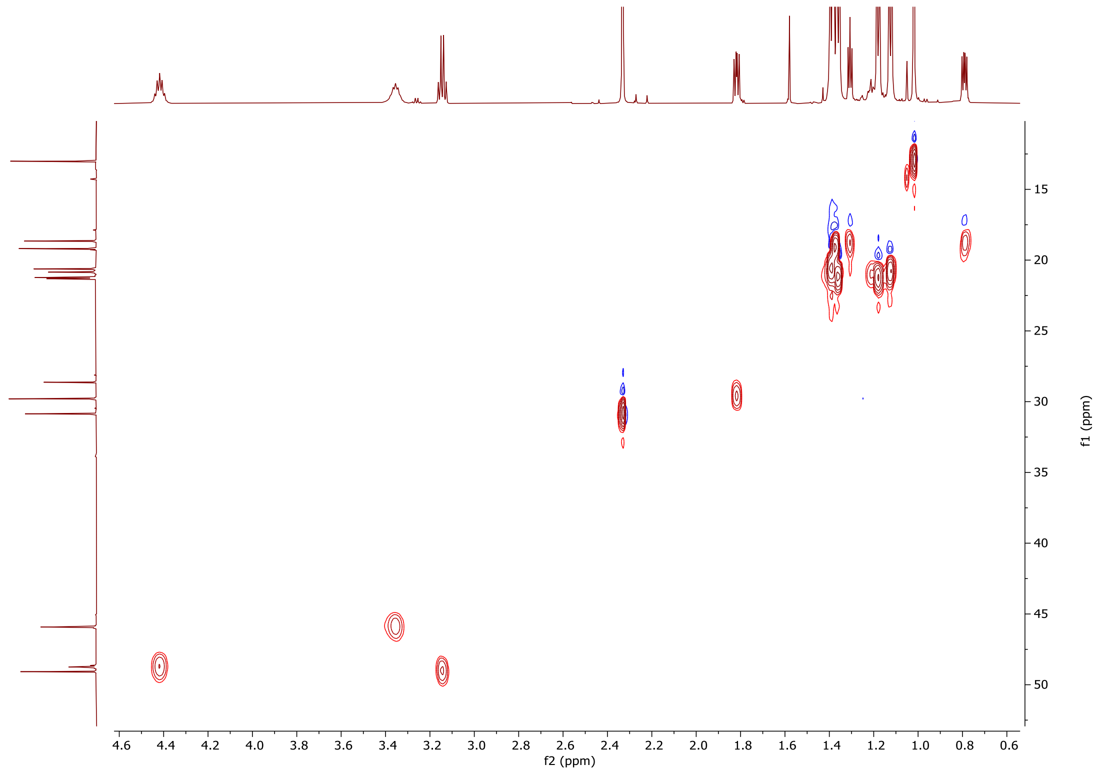

^{1}H/^{13}C HMBC (600/151 MHz, Chloroform-*d*)

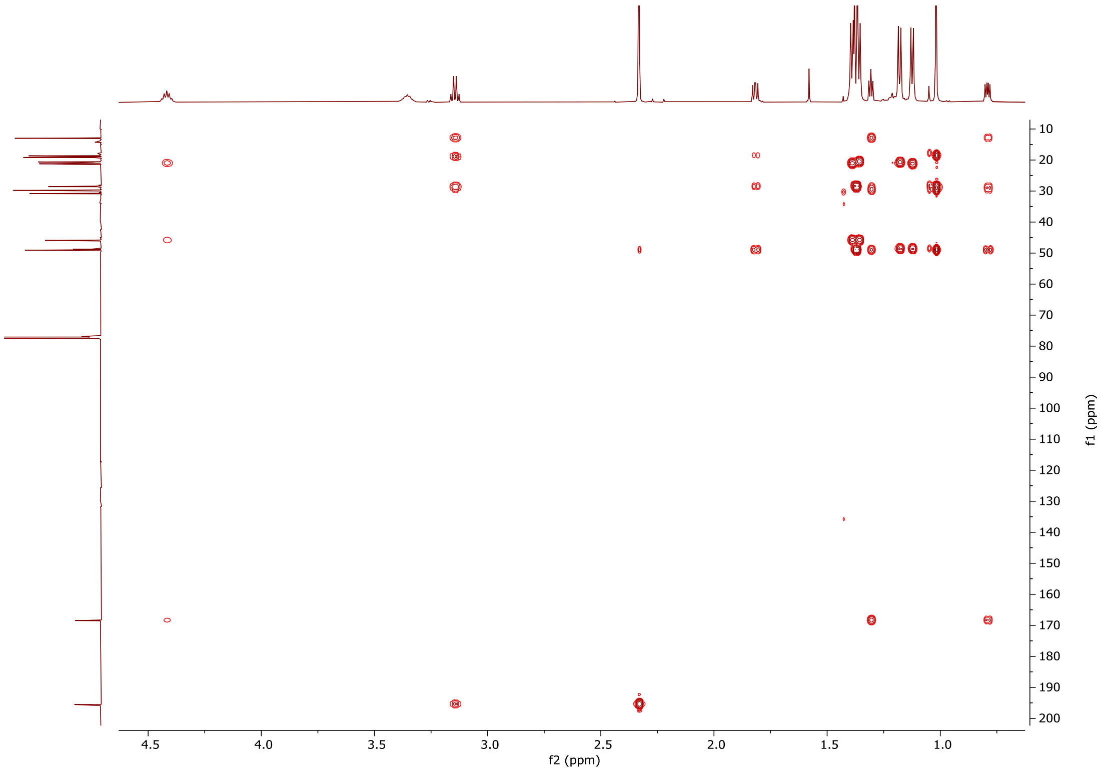

^{1}H NOSEY (600 MHz, Chloroform-*d*)

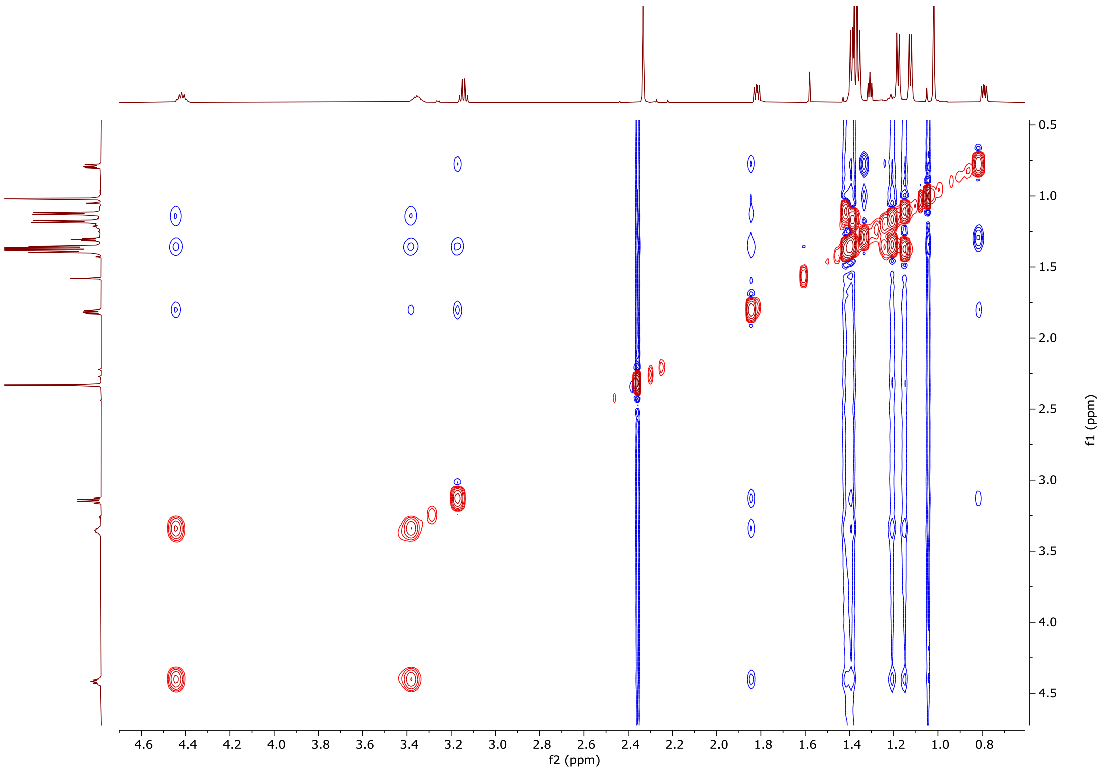

(1*R,2*R**)-2-((*S**)-1-((1,1-dioxidobenzo[d]isothiazol-3-yl)oxy)ethyl)-*N*,*N*-diisopropyl-2-methylcyclopropane-1-carboxamide, 3x**

^{1}H NMR (600 MHz, Chloroform-*d*)

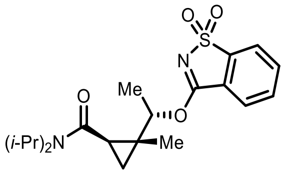

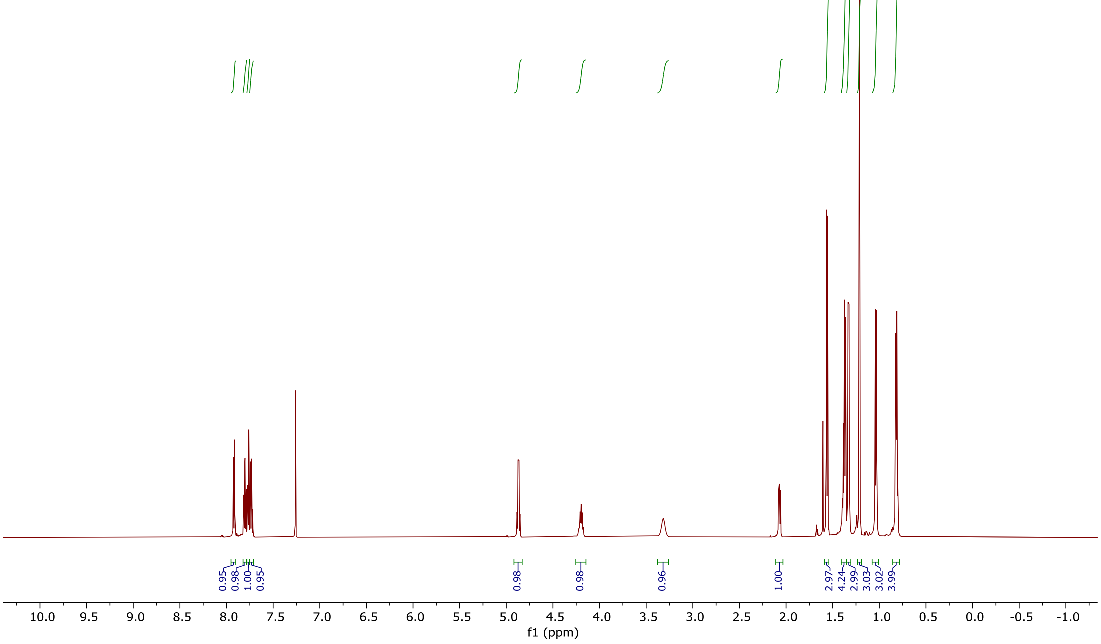

^{13}C NMR (151 MHz, Chloroform-*d*)

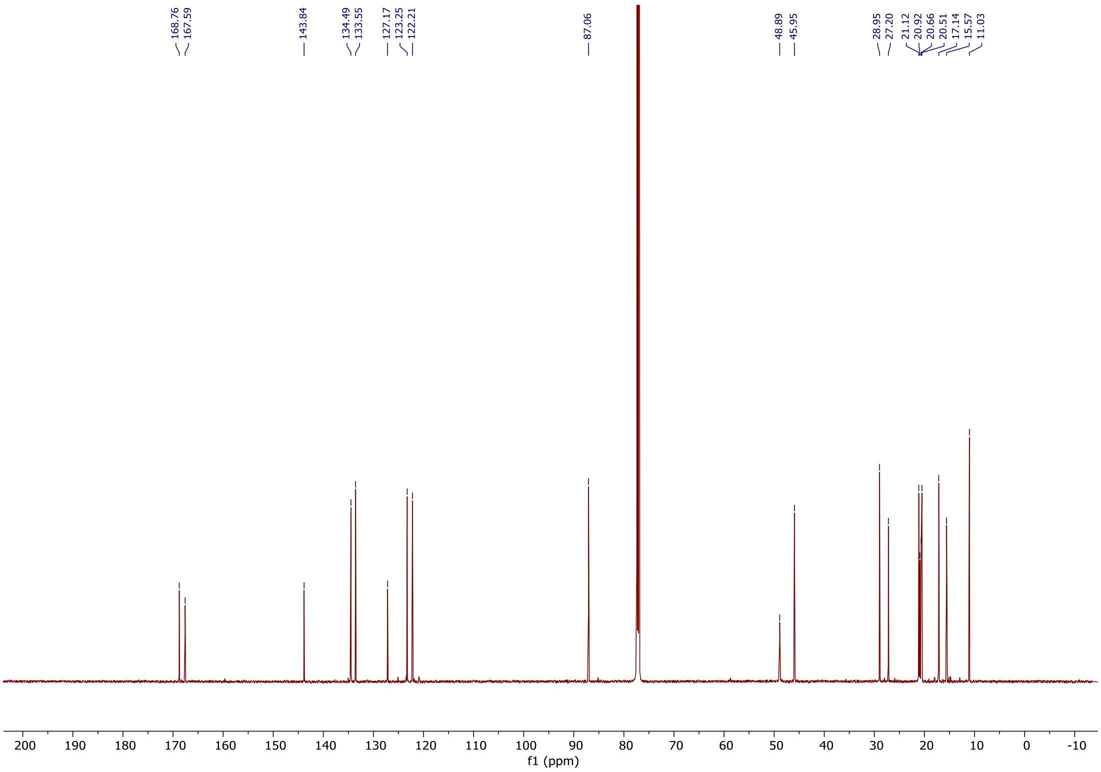

^{1}**H COSY** (600 MHz, Chloroform-*d*)

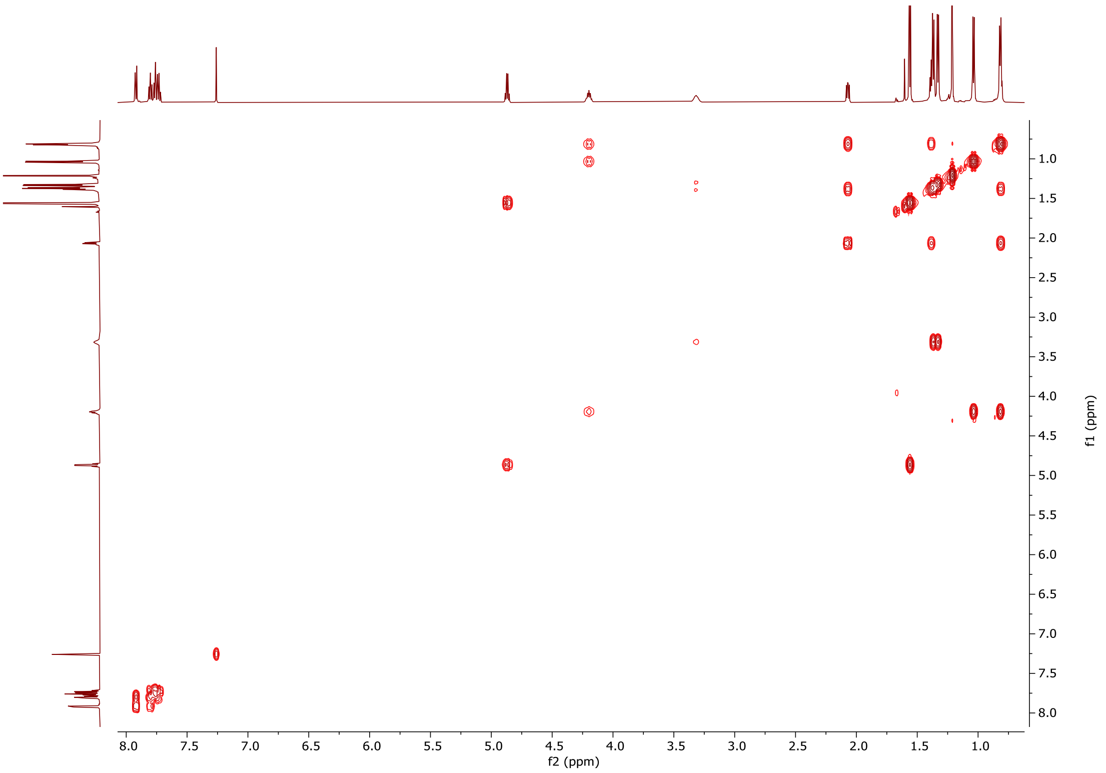

^{1}**H/**^{13}**C HSQC** (600/151 MHz, Chloroform-*d*)

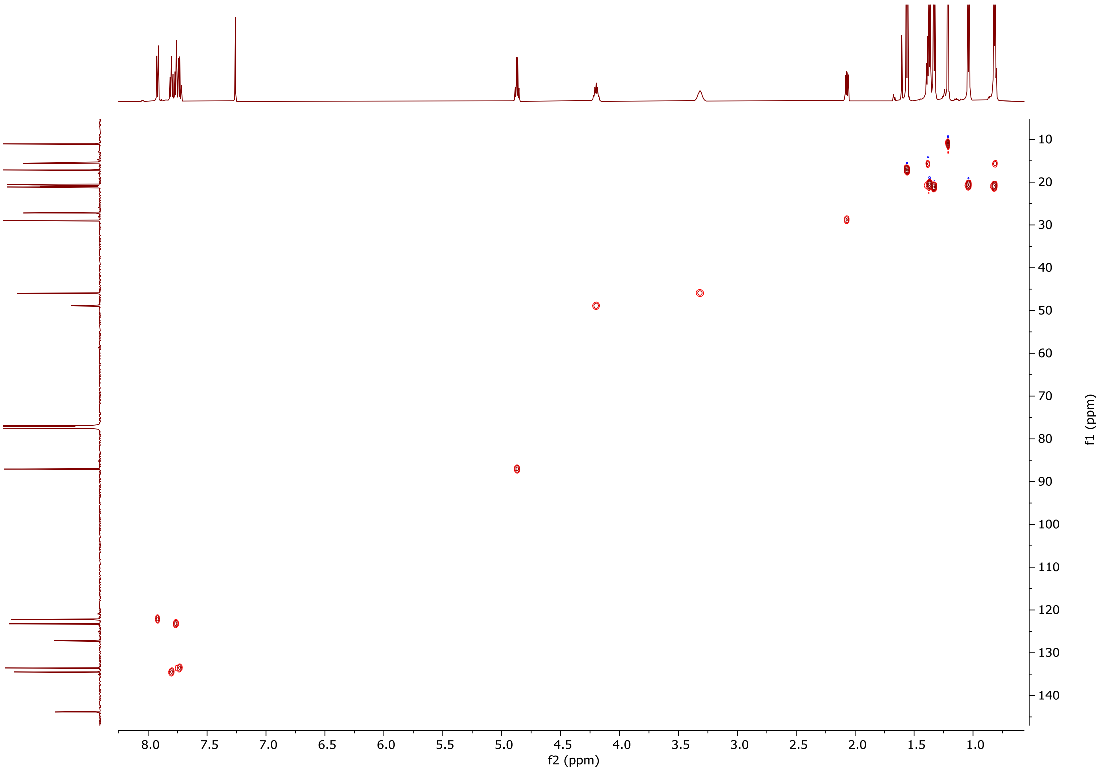

$^{1}H/^{13}C$ **HMBC** (600/151 MHz, Chloroform-*d*)

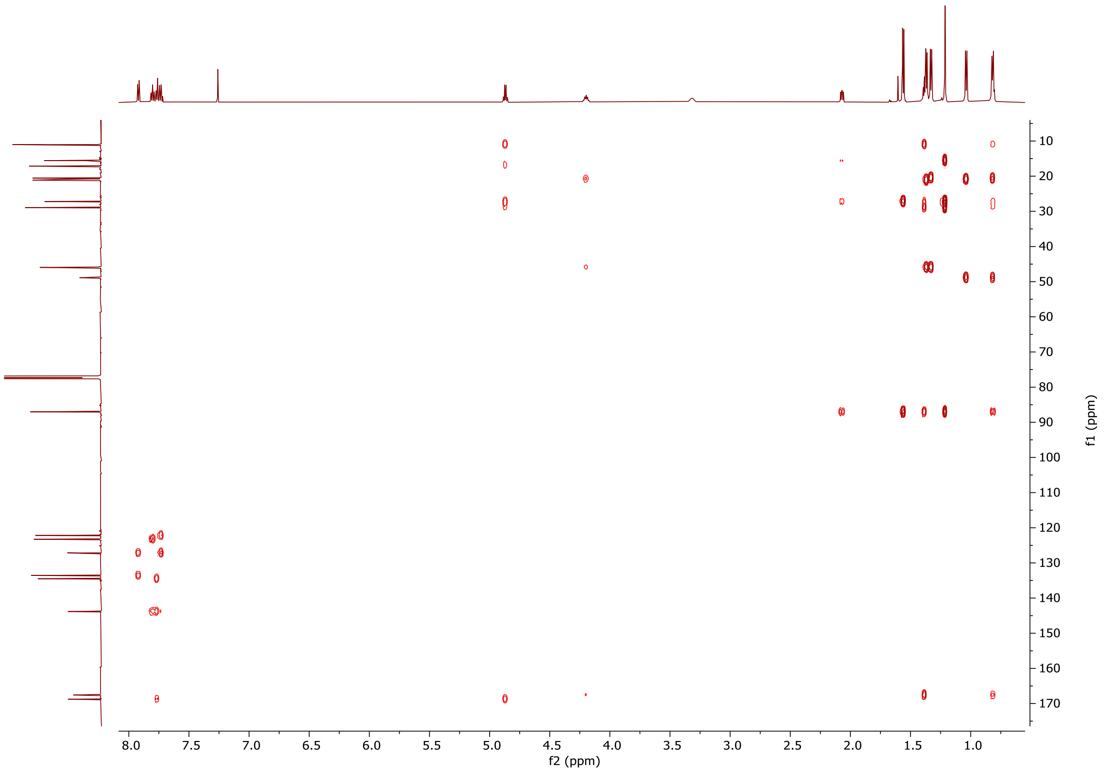

^{1}H **NOSEY** (600 MHz, Chloroform-*d*)

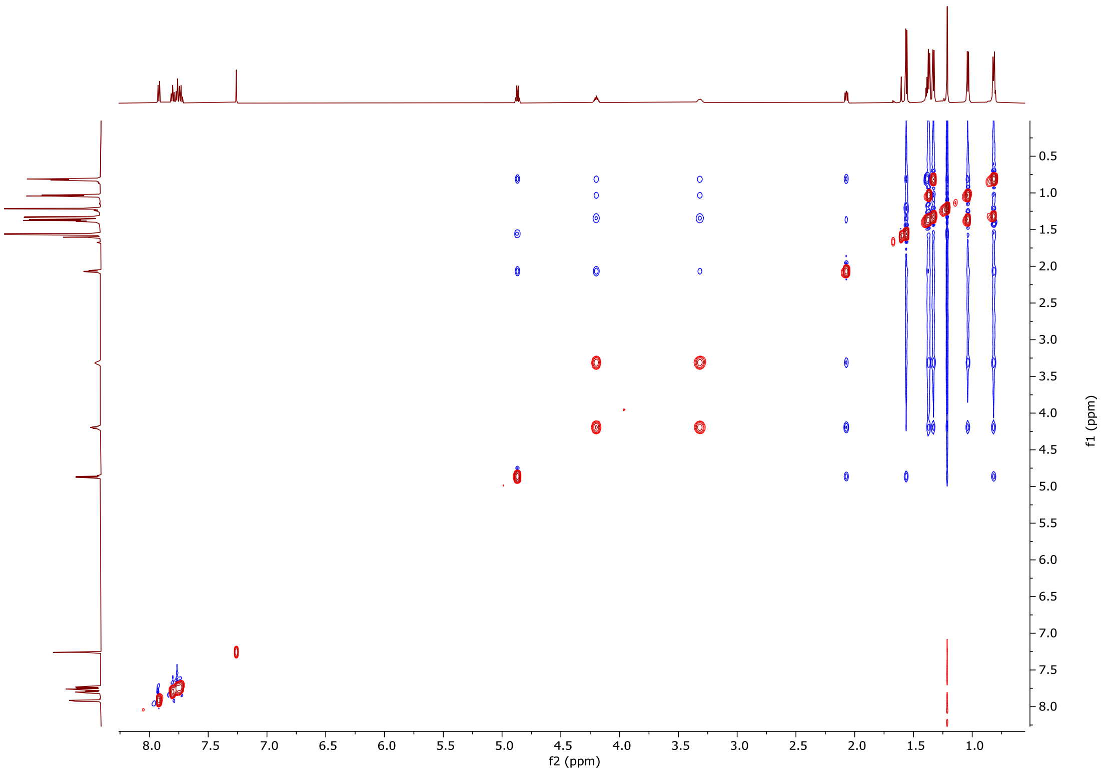

(1*R,2*R**)-2-((*S**)-1-(1,1-Dioxido-3-oxobenzo[*d*]isothiazol-2(3*H*)-yl)ethyl)-*N*,*N*-diisopropyl-2-methylcyclopropane-1-carboxamide, 3y**

^{1}H NMR (600 MHz, Chloroform-*d*)

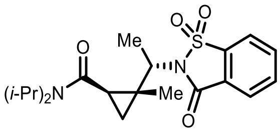

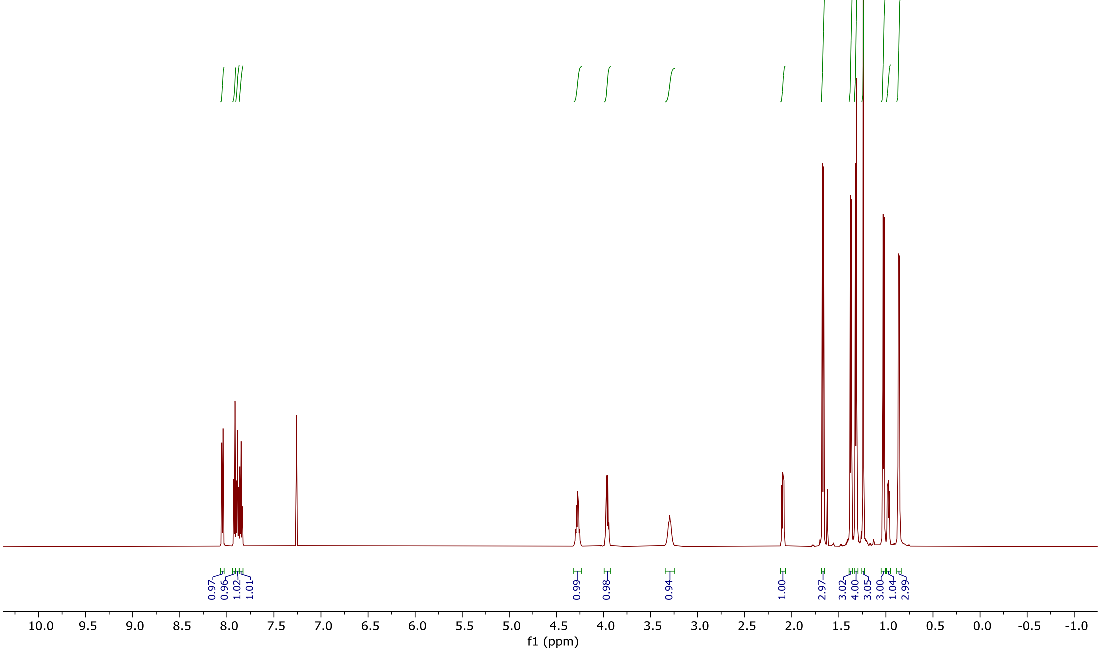

^{13}C NMR (151 MHz, Chloroform-*d*)

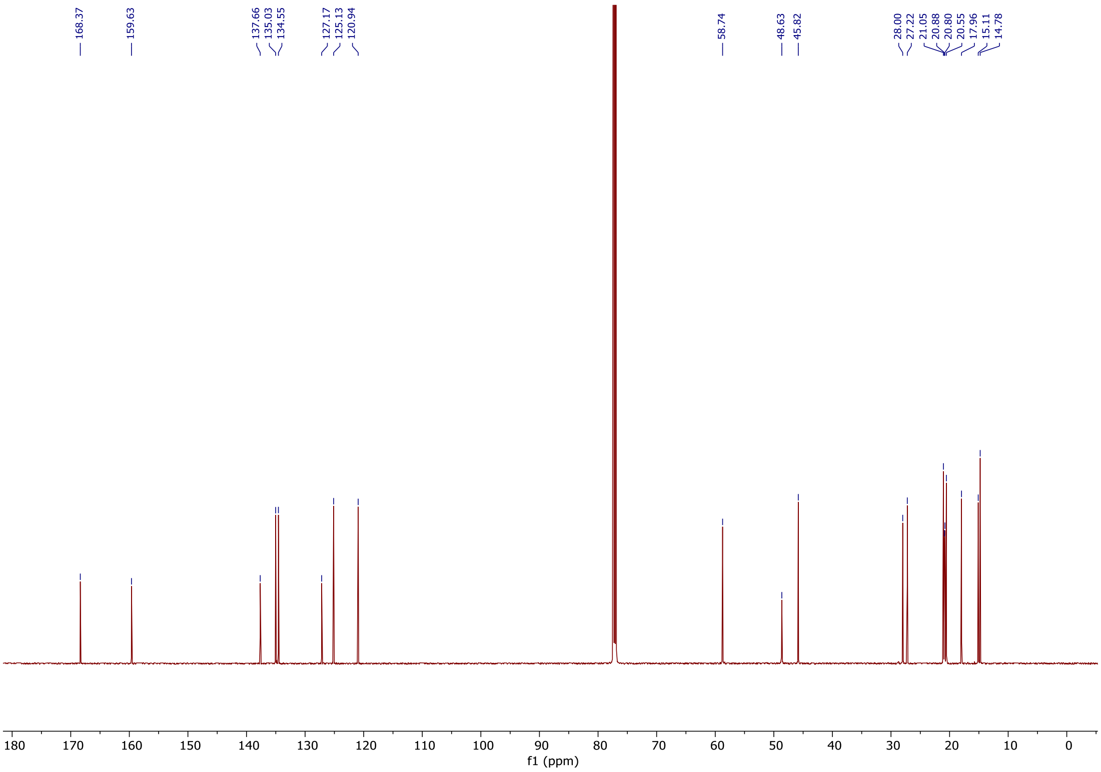

^{1}H COSY (600 MHz, Chloroform-*d*)

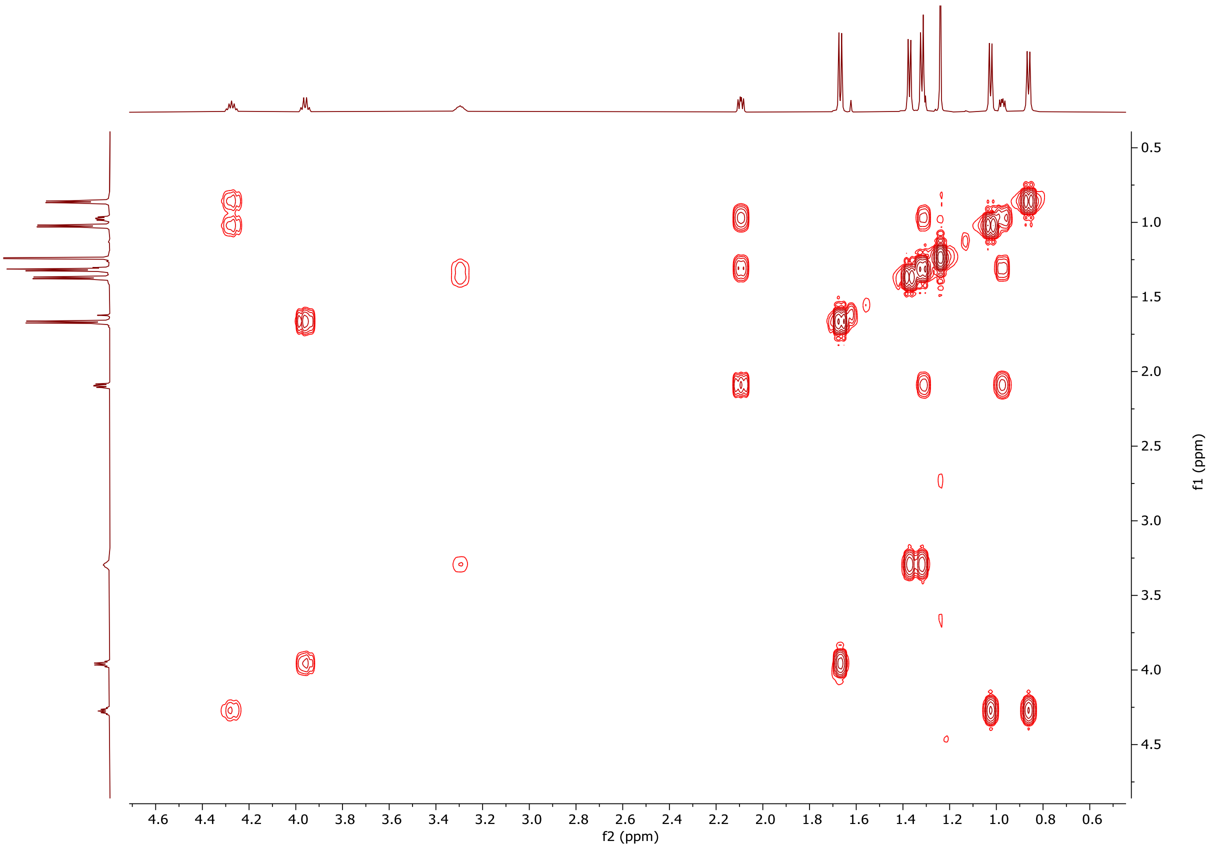

^{1}H/^{13}C HSQC (600/151 MHz, Chloroform-*d*)

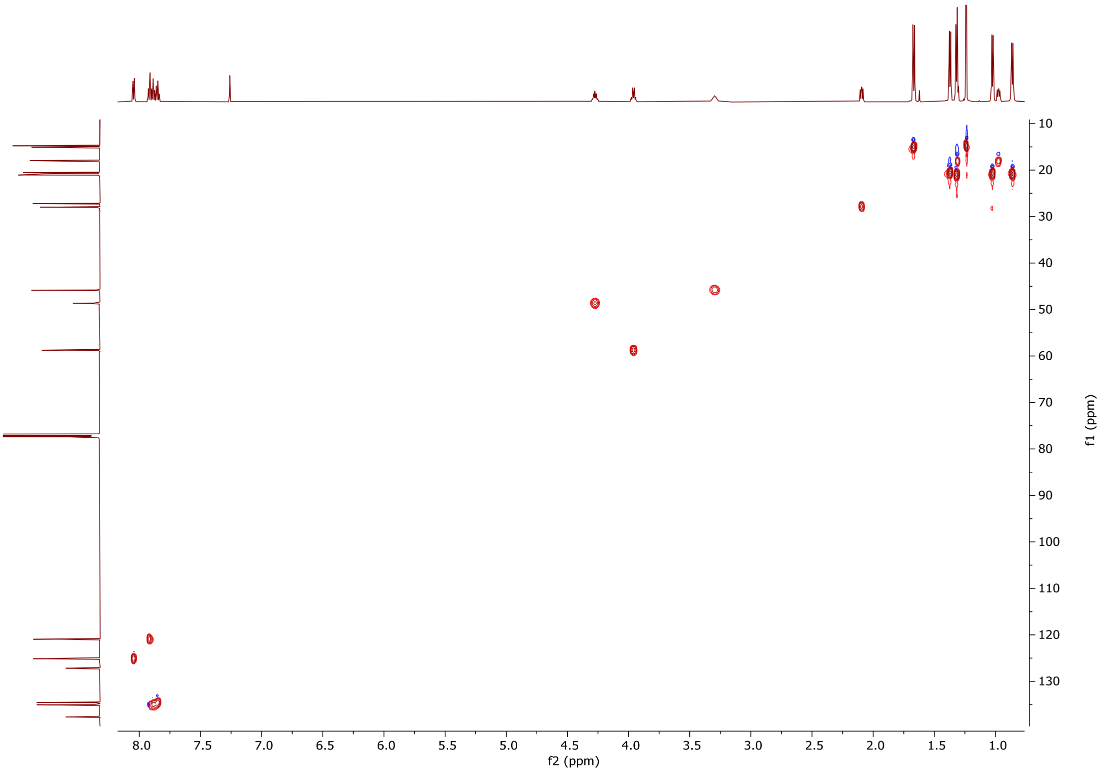

$^{1}H/^{13}C$ **HMBC** (600/151 MHz, Chloroform-*d*)

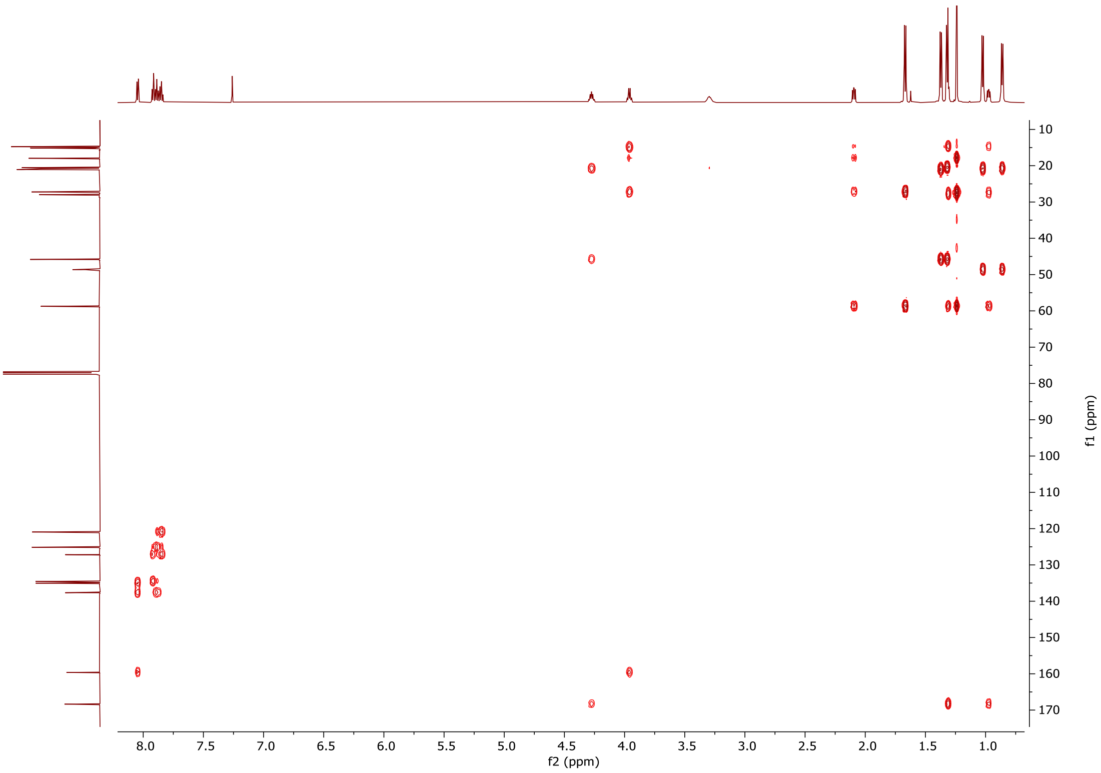

^{1}H **NOSEY** (600 MHz, Chloroform-*d*)

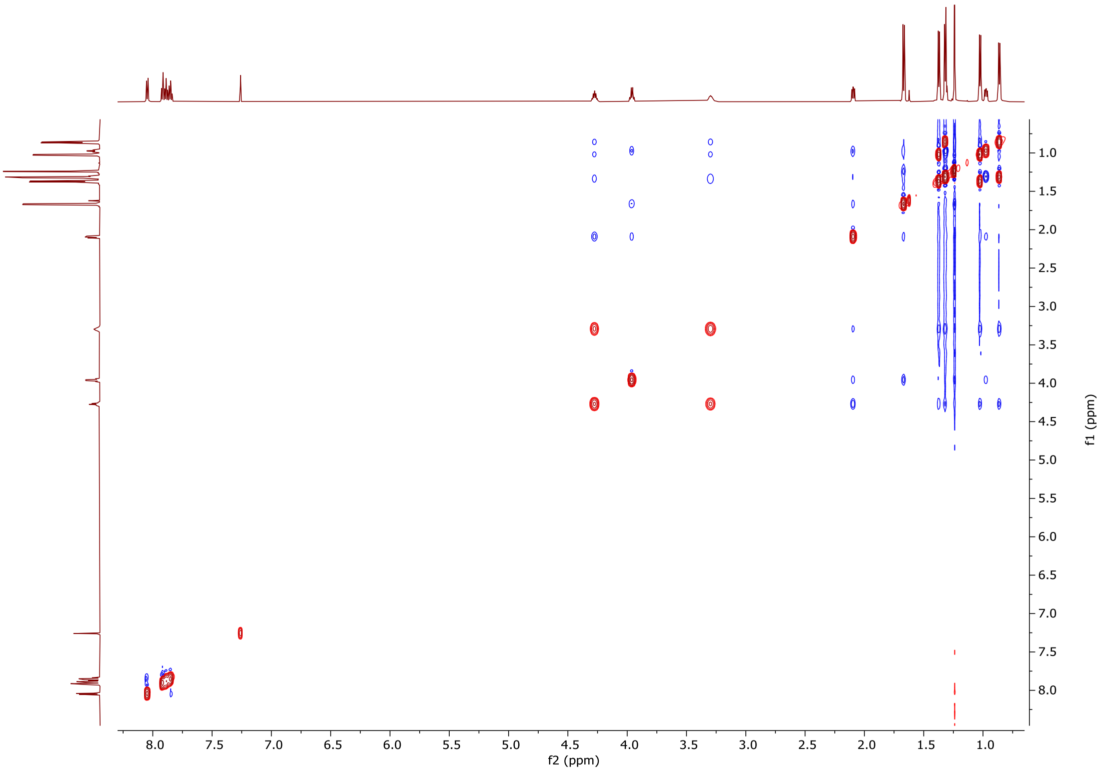

(1*R,2*R**)-*N*,*N*-Diisopropyl-2-methyl-2-((*S**)-1-((1-phenyl-1*H*-tetrazol-5-yl)thio)ethyl)cyclopropane-1-carboxamide, 3z**

^{1}H NMR (600 MHz, Chloroform-*d*)

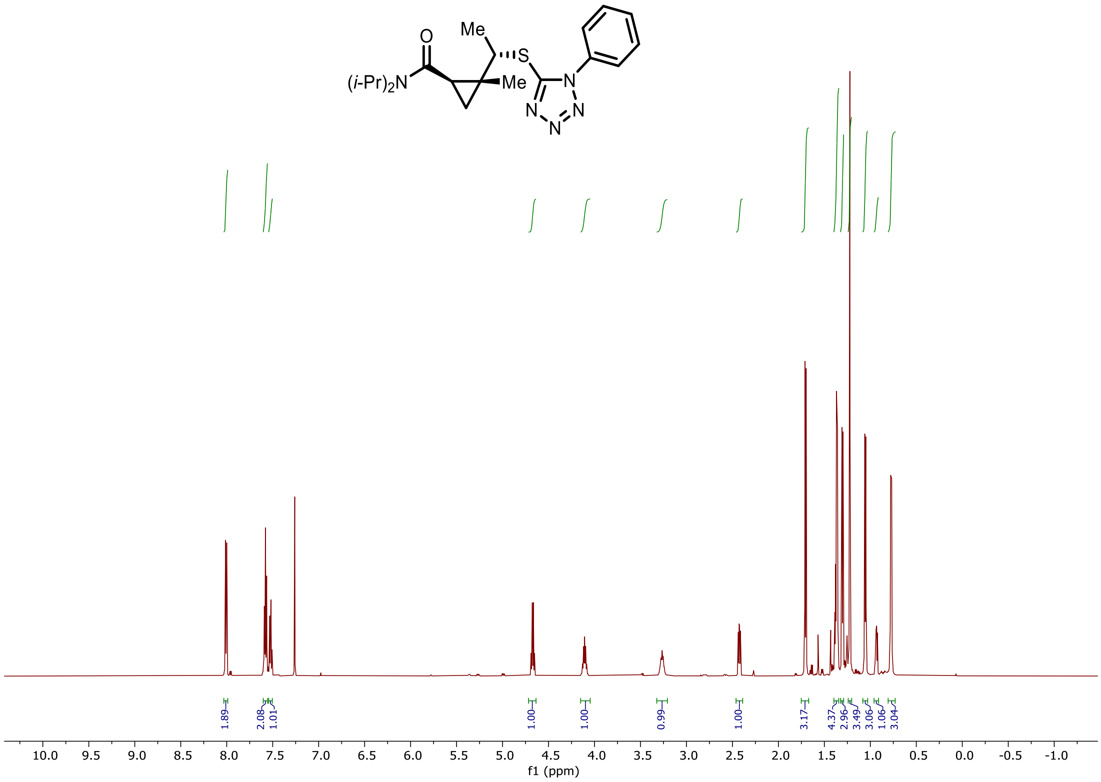

^{13}C NMR (151 MHz, Chloroform-*d*)

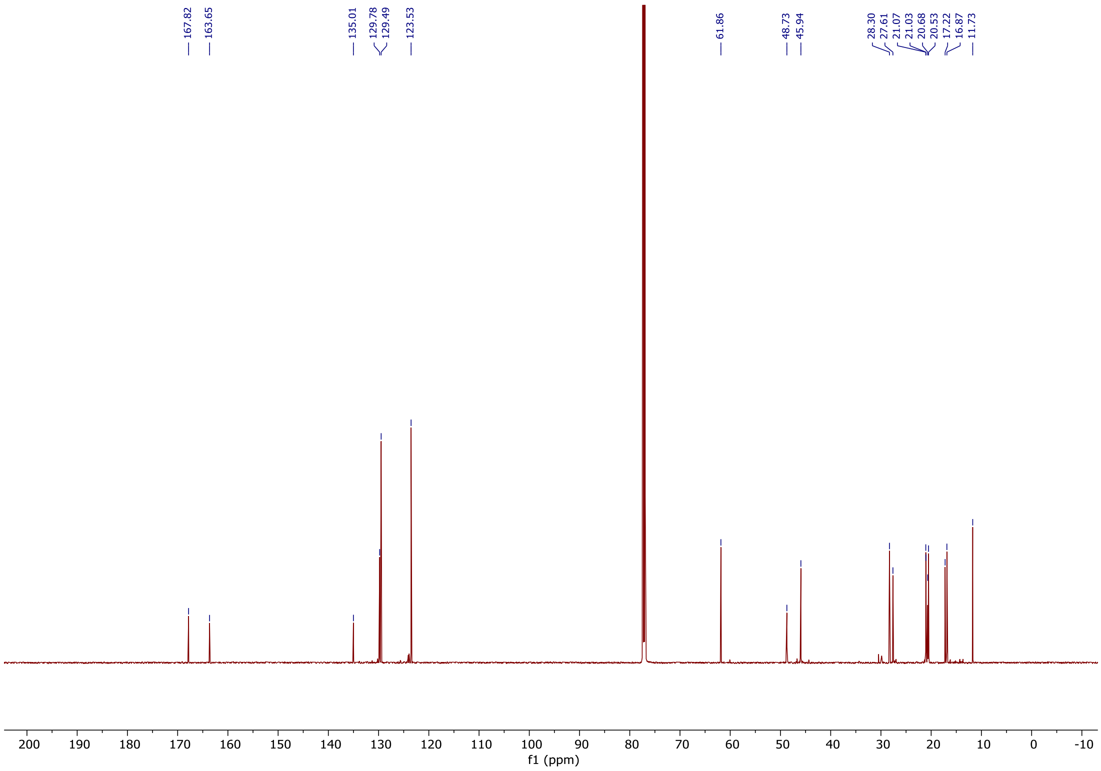

^{1}**H COSY** (600 MHz, Chloroform-*d*)

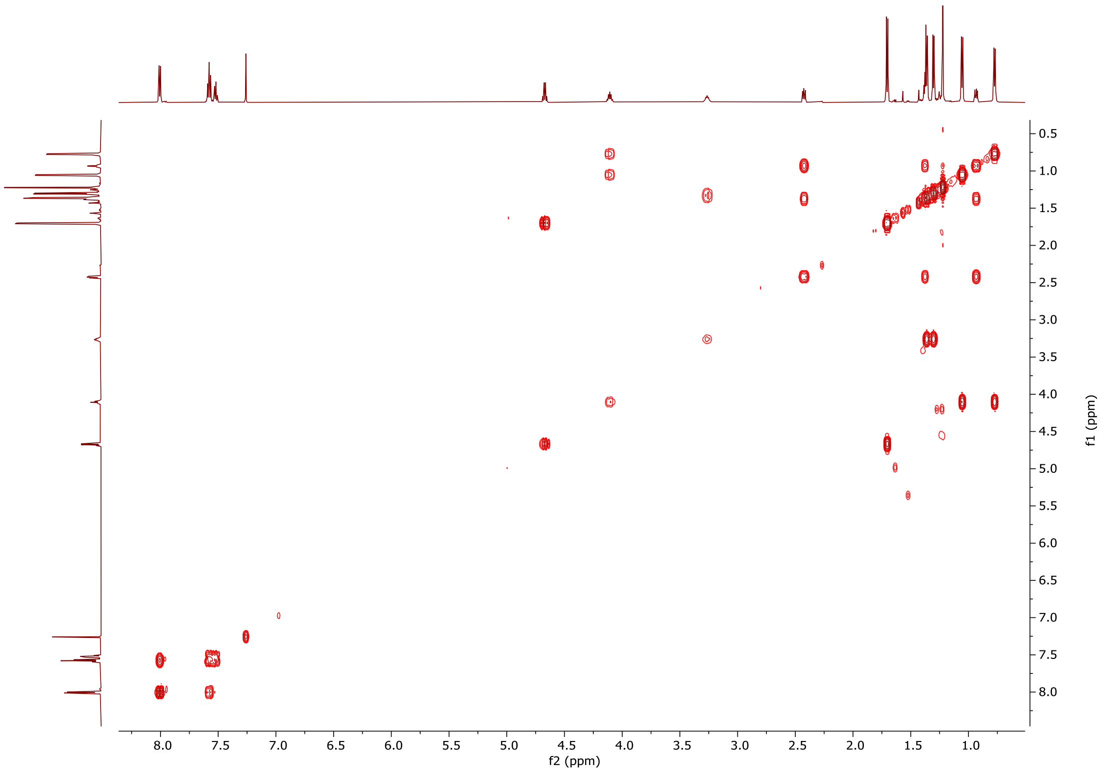

^{1}**H/**^{13}**C HSQC** (600/151 MHz, Chloroform-*d*)

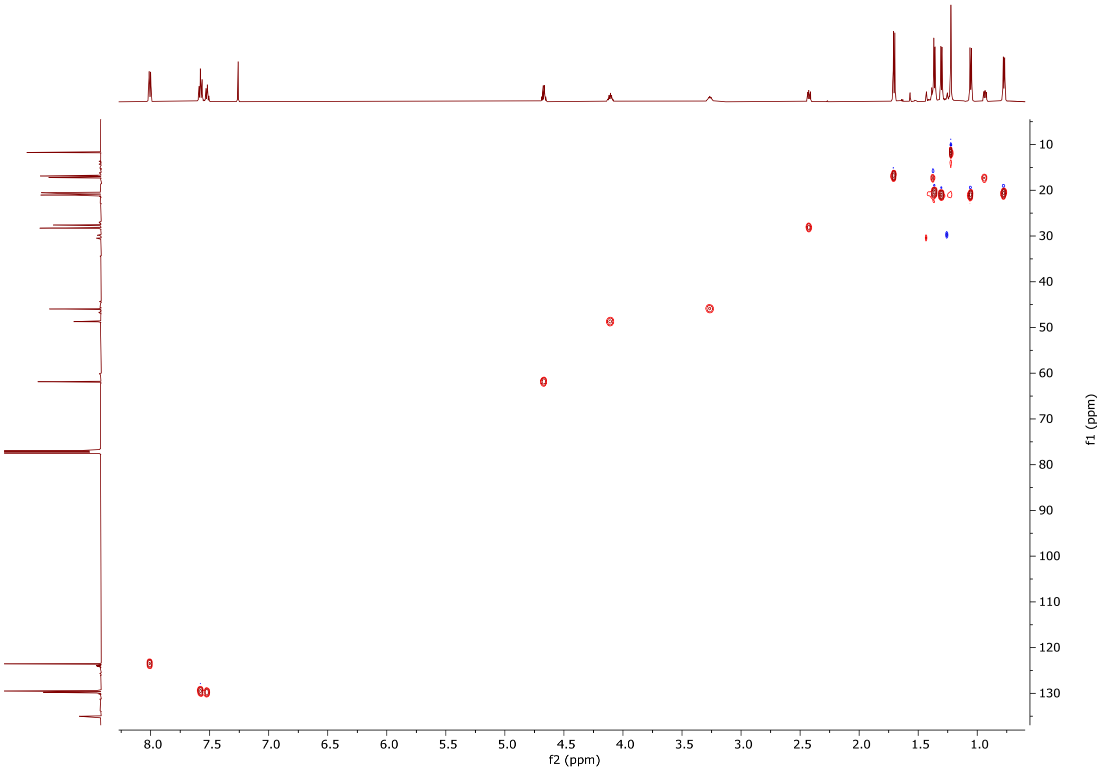

$^{1}H/^{13}C$ **HMBC** (600/151 MHz, Chloroform-*d*)

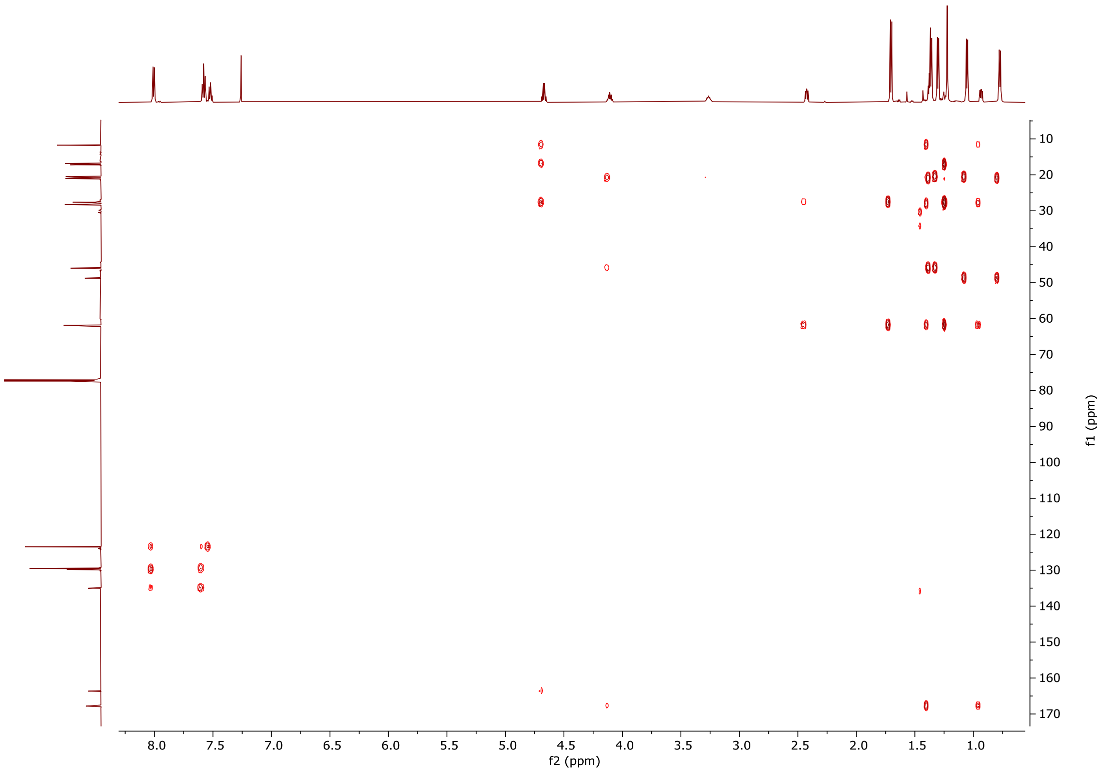

^{1}H **NOSEY** (600 MHz, Chloroform-*d*)

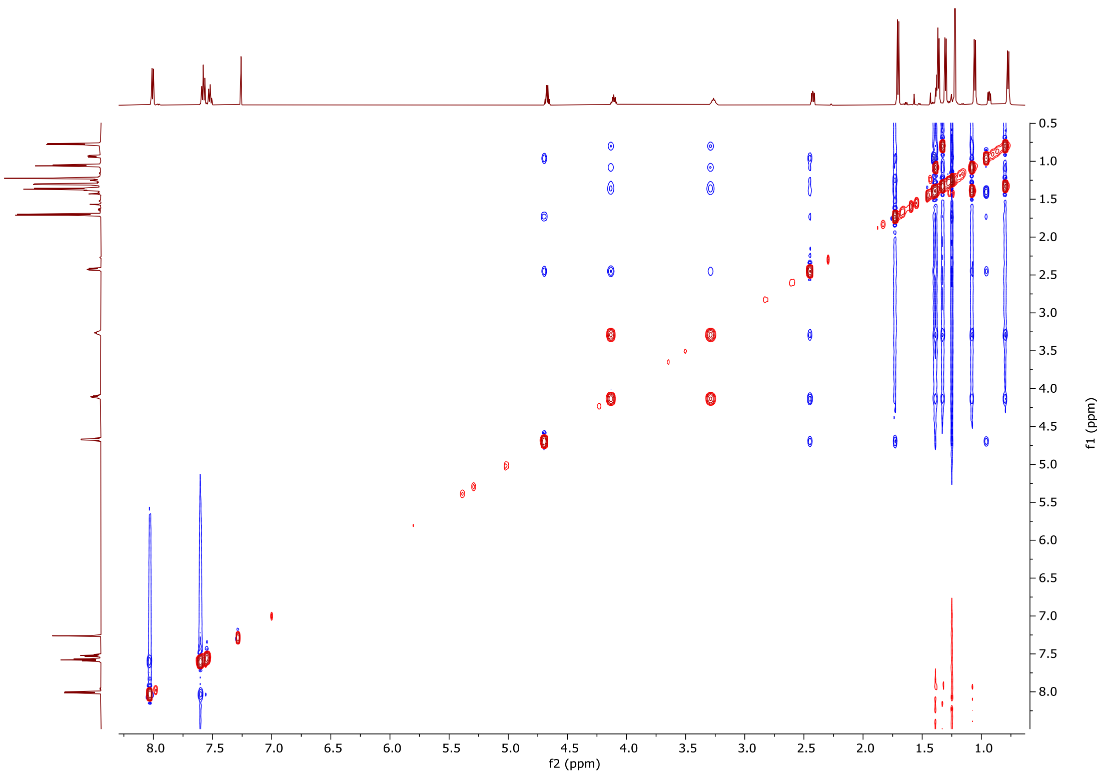

(1*R,2*R**)-*N*,*N*-Diisopropyl-2-methyl-2-((*S**)-1-(4-phenyl-5-thioxo-4,5-dihydro-1*H*-tetrazol-1-yl)ethyl)cyclopropane-1-carboxamide, 3aa**

[1]**H NMR** (600 MHz, Chloroform-*d*)

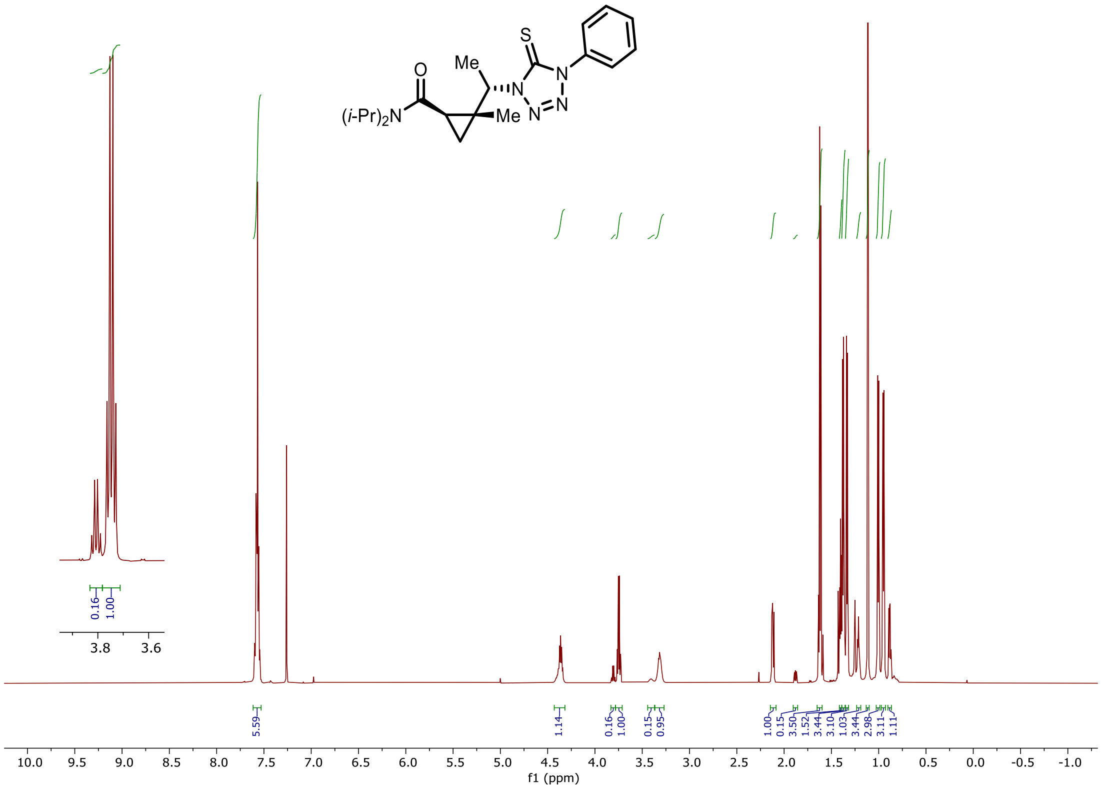

[13]**C NMR** (151 MHz, Chloroform-*d*)

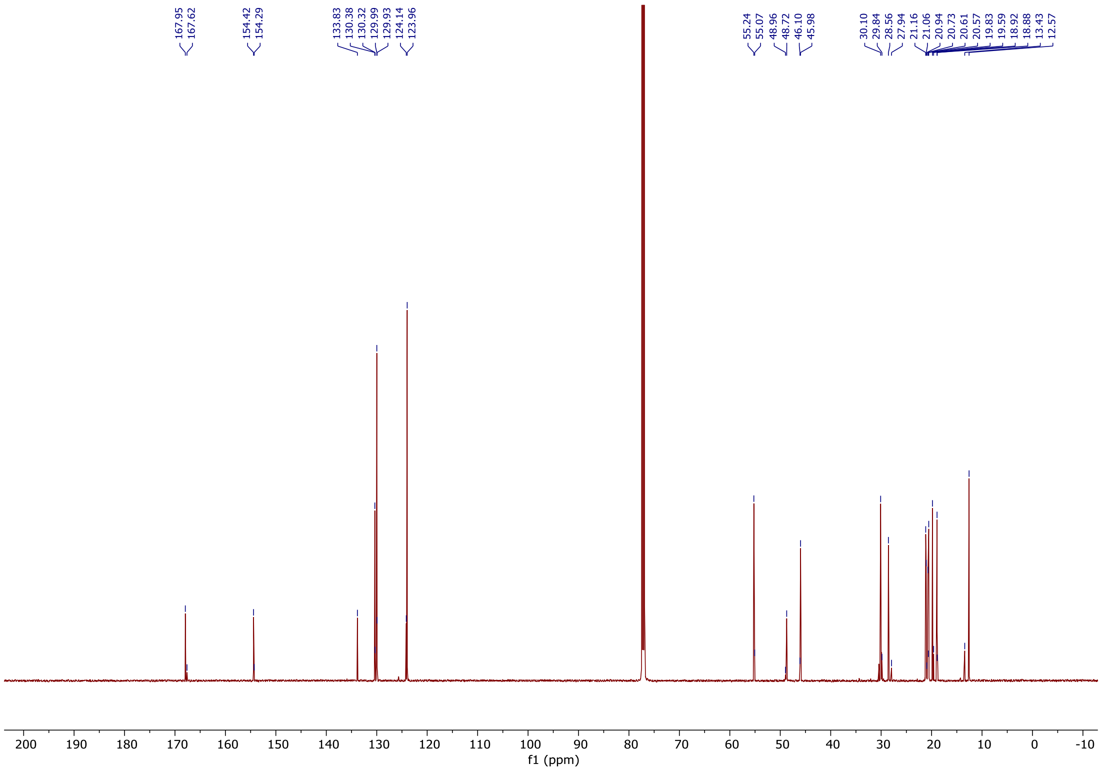

^{1}**H COSY** (600 MHz, Chloroform-*d*)

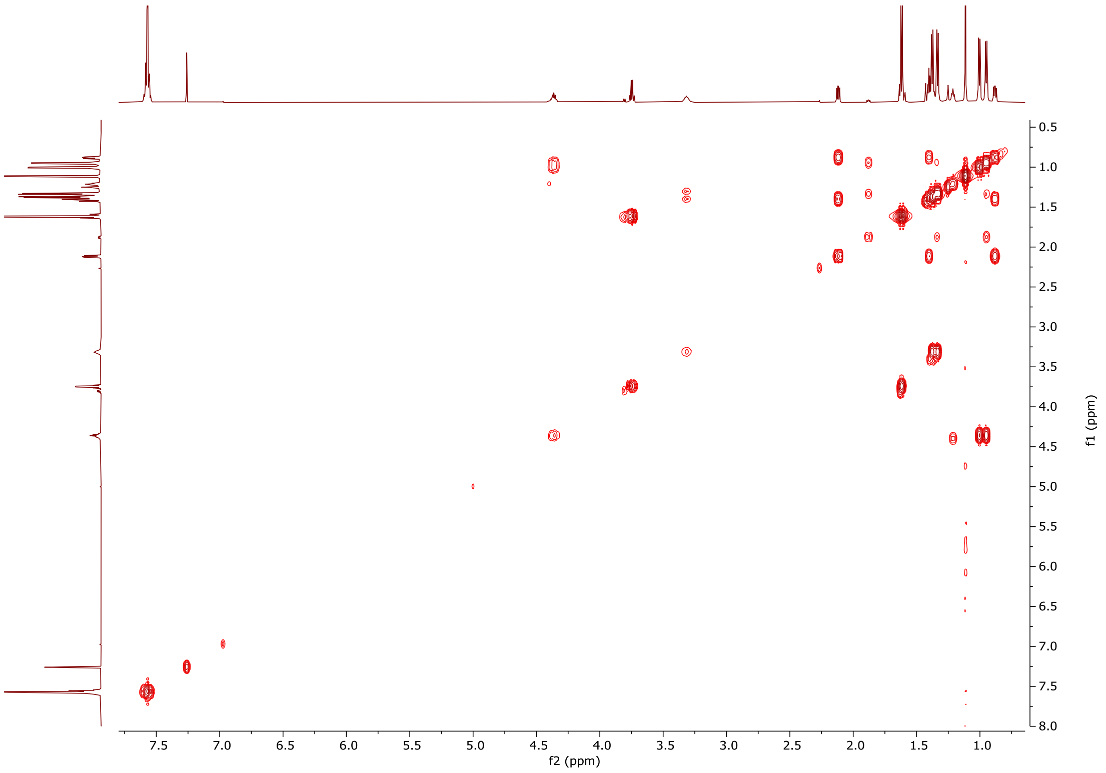

^{1}**H/**^{13}**C HSQC** (600/151 MHz, Chloroform-*d*)

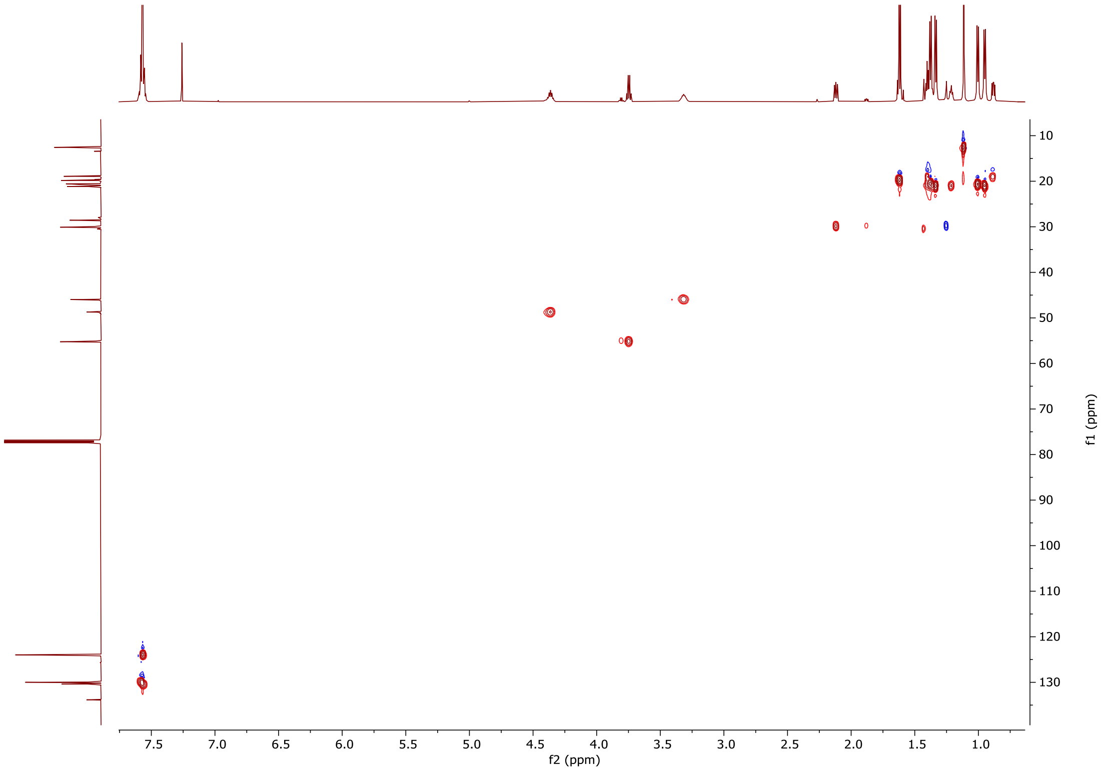

$^{1}H/^{13}C$ HMBC (600/151 MHz, Chloroform-*d*)

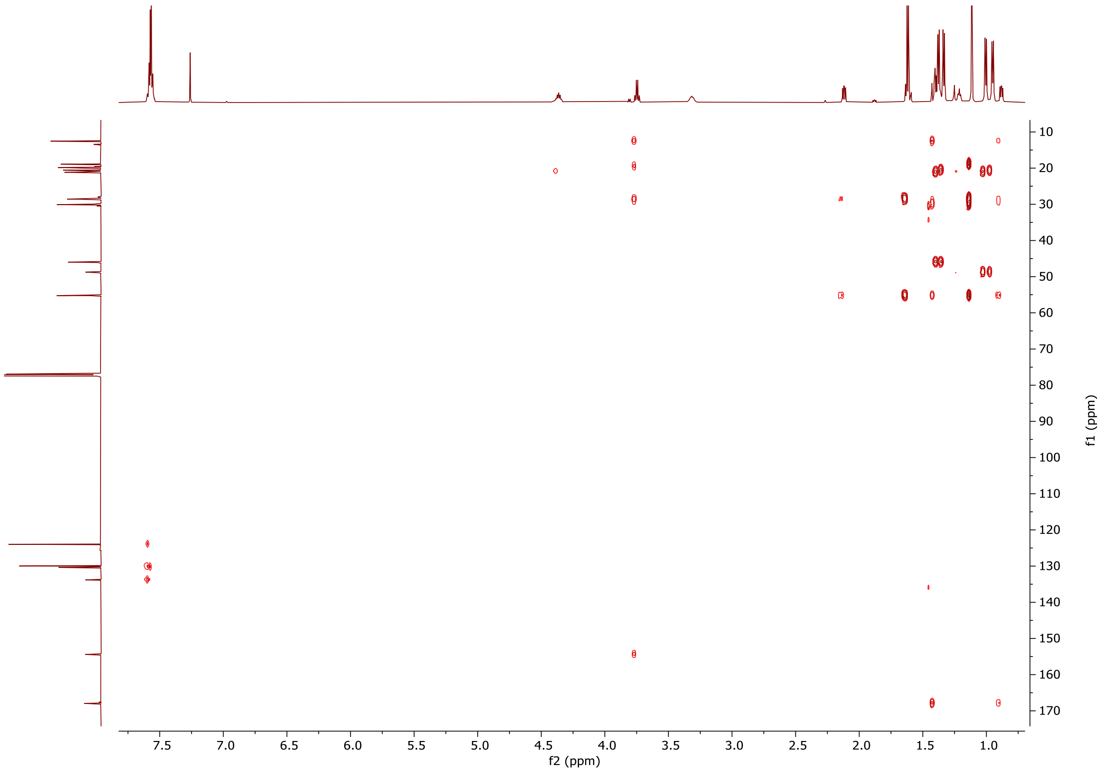

^{1}H NOSEY (600 MHz, Chloroform-*d*)

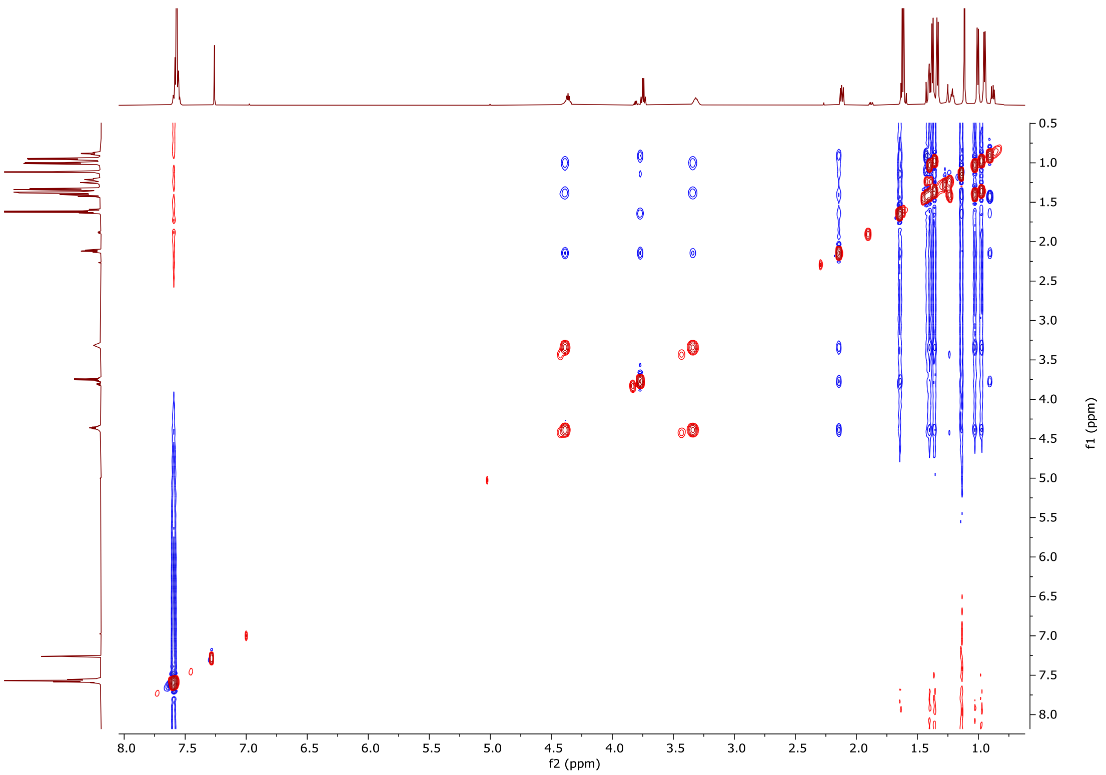

(*R)-1-((1*R**,2*R**)-2-(diisopropylcarbamoyl)-1-methylcyclopropyl-2-*d*)ethyl 2,2,2-trifluoroacetate, 3ab**

^{1}H NMR (600 MHz, Chloroform-*d*)

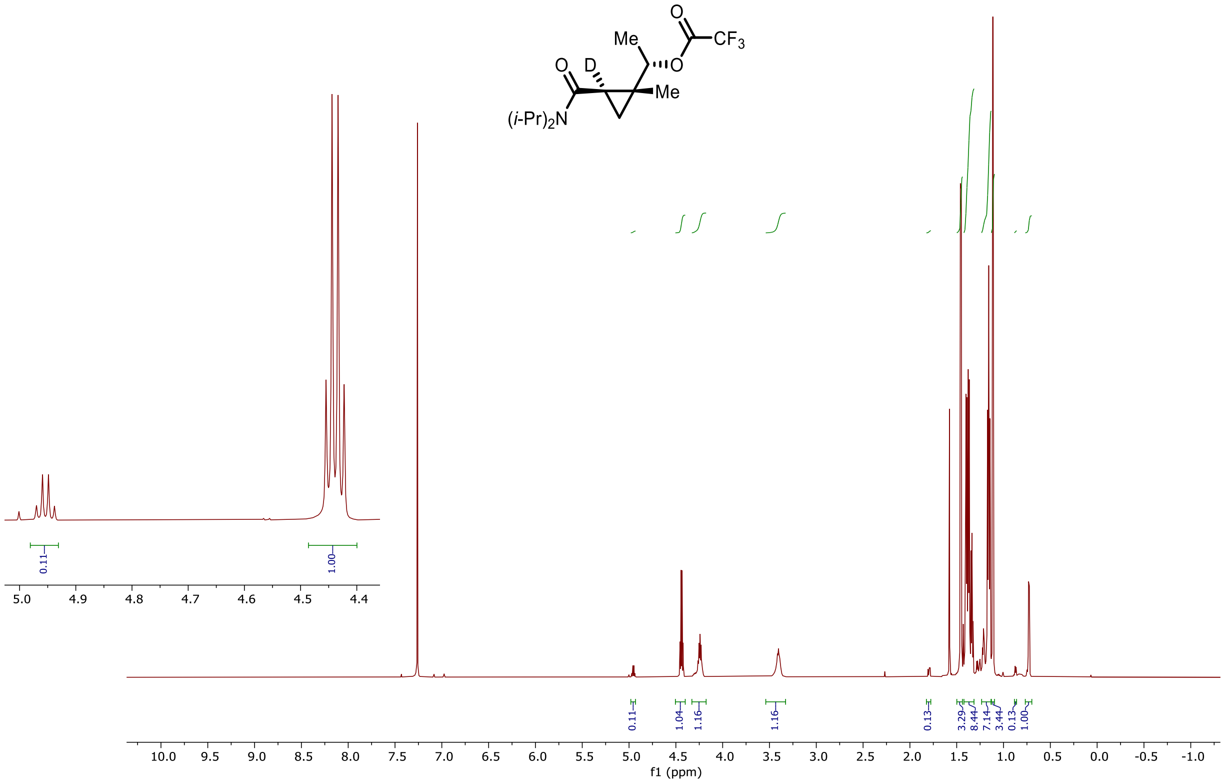

^{13}C NMR (151 MHz, Chloroform-*d*)

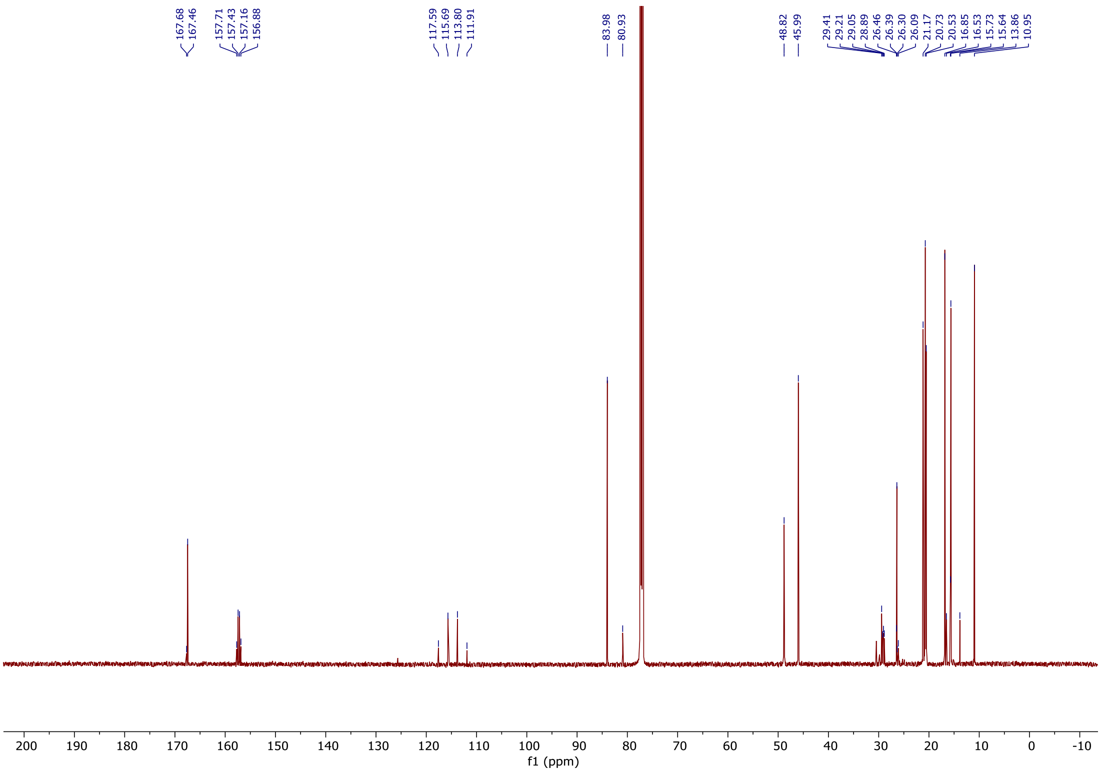

^{1}H COSY (600 MHz, Chloroform-*d*)

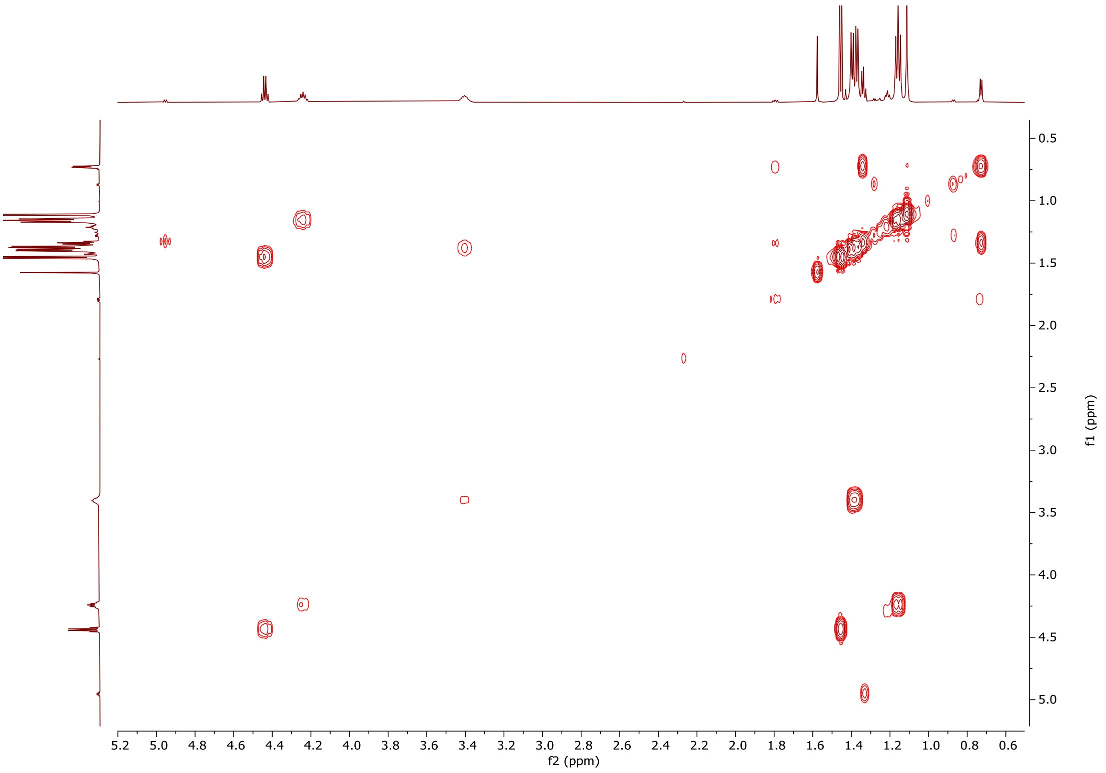

^{1}H/^{13}C HSQC (600/151 MHz, Chloroform-*d*)

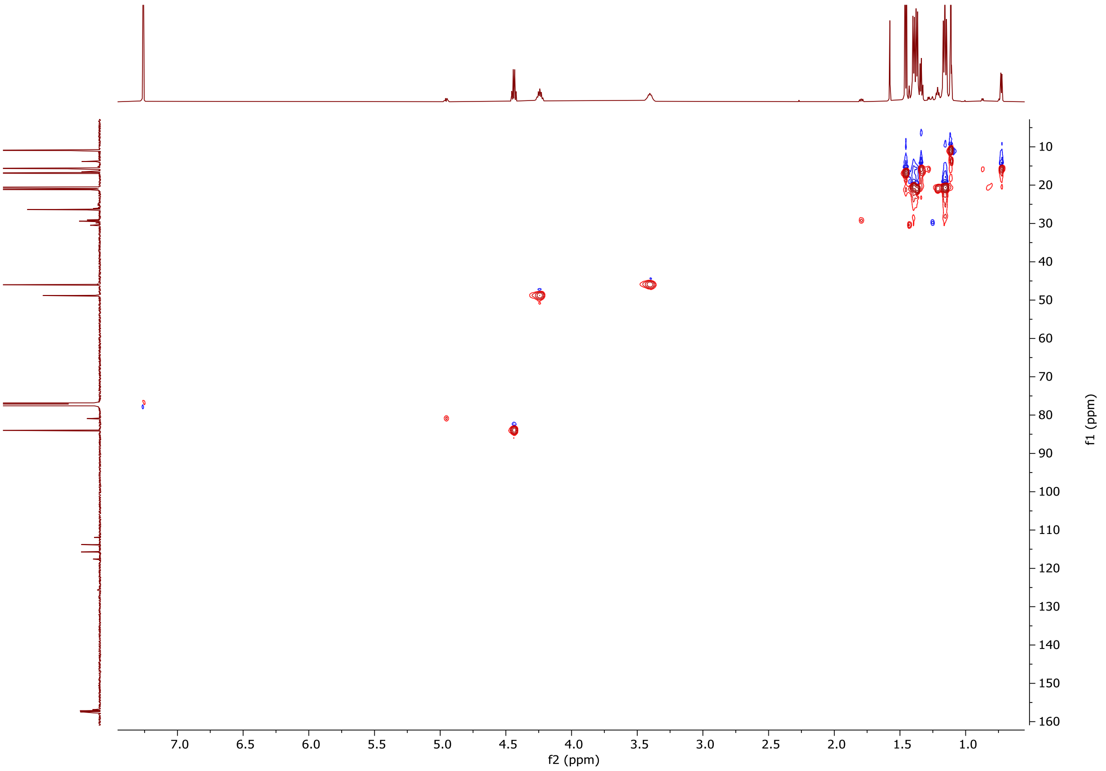

$^{1}H/^{13}C$ **HMBC** (600/151 MHz, Chloroform-*d*)

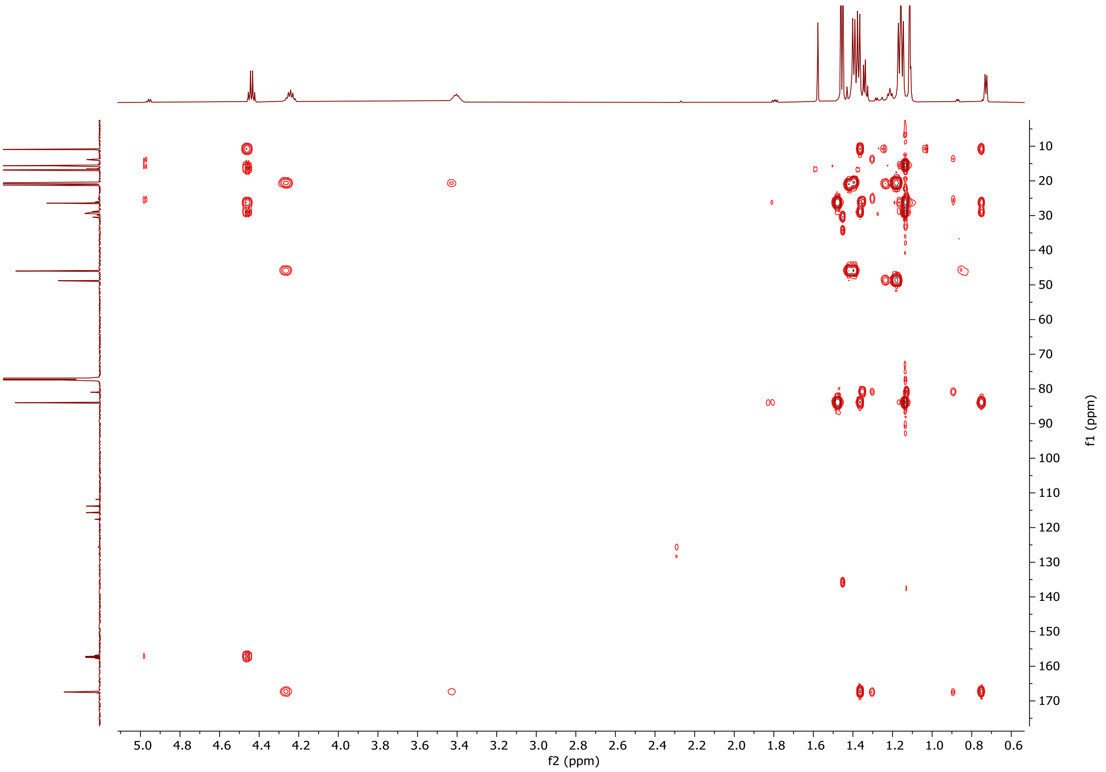

^{1}H **NOSEY** (600 MHz, Chloroform-*d*)

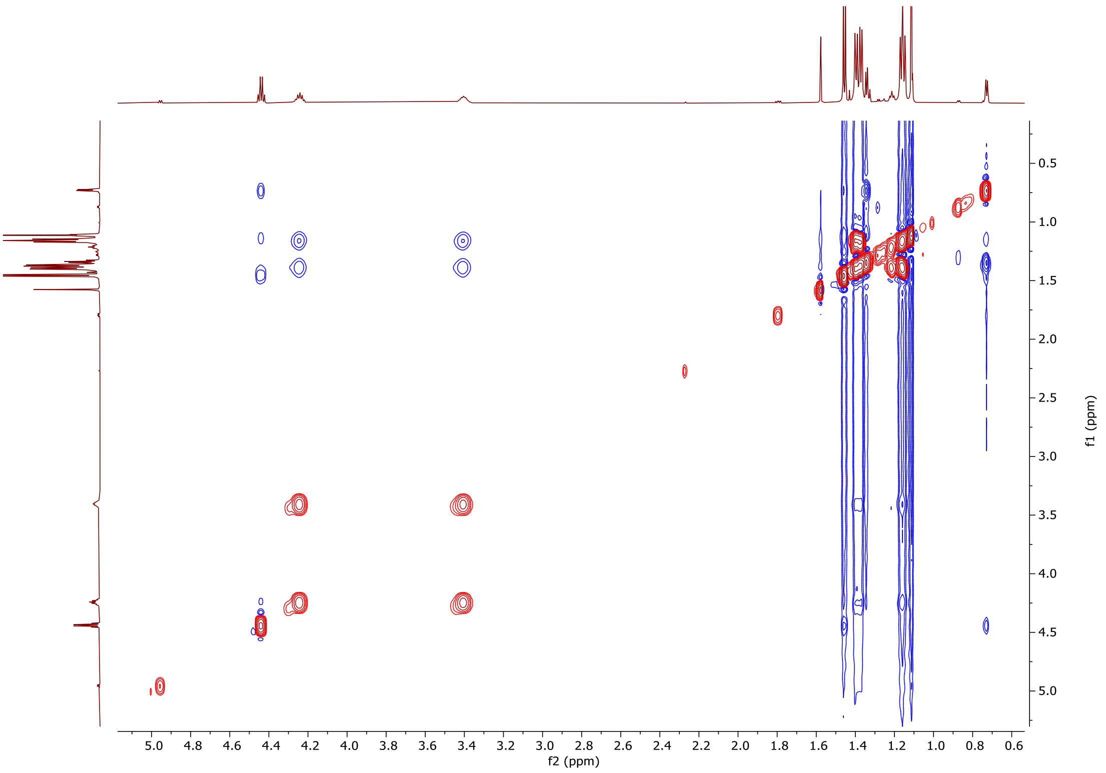

^{19}F NMR (565 MHz, Chloroform-*d*)

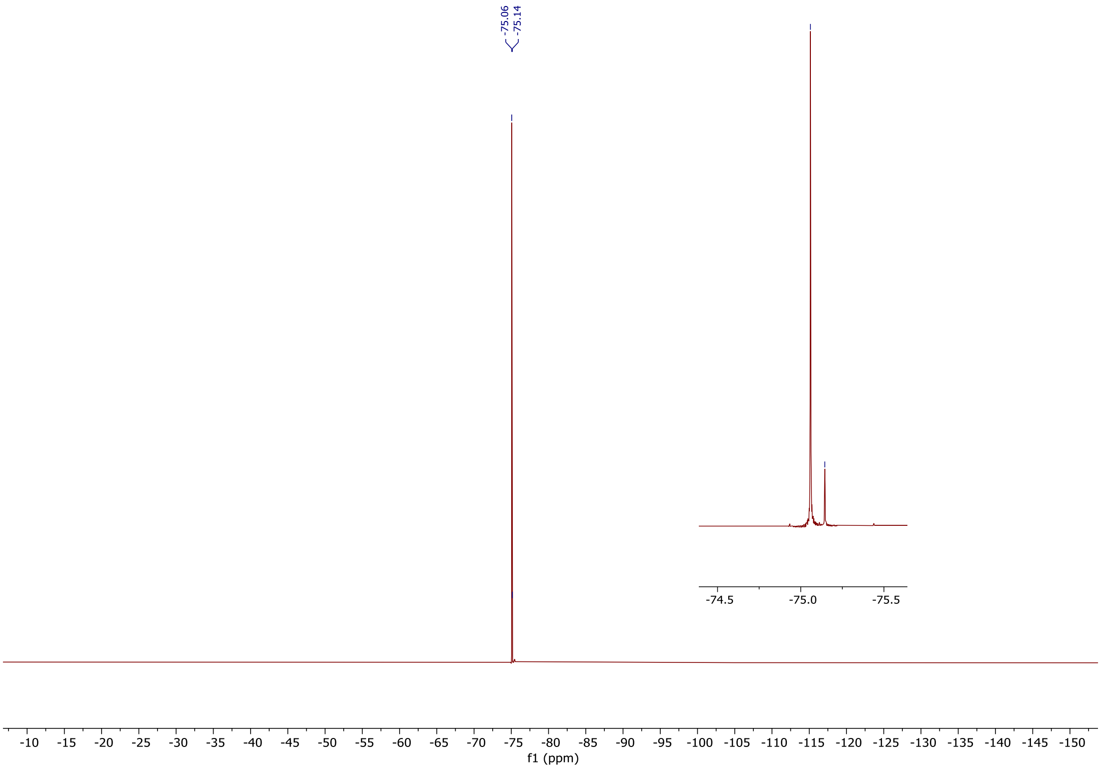

(1*R,2*R**)-*N*,*N*-diisopropyl-2-((*S**)-1-methoxyethyl)-2-methylcyclopropane-1-carboxamide, (*S**)-3ad**

(1*R,2*R**,3*R**)-*N*,*N*-diisopropyl-3-methoxy-2,3-dimethylcyclobutane-1-carboxamide, S7**

^{1}H NMR (600 MHz, Chloroform-*d*)

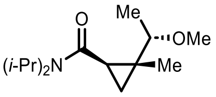

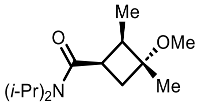

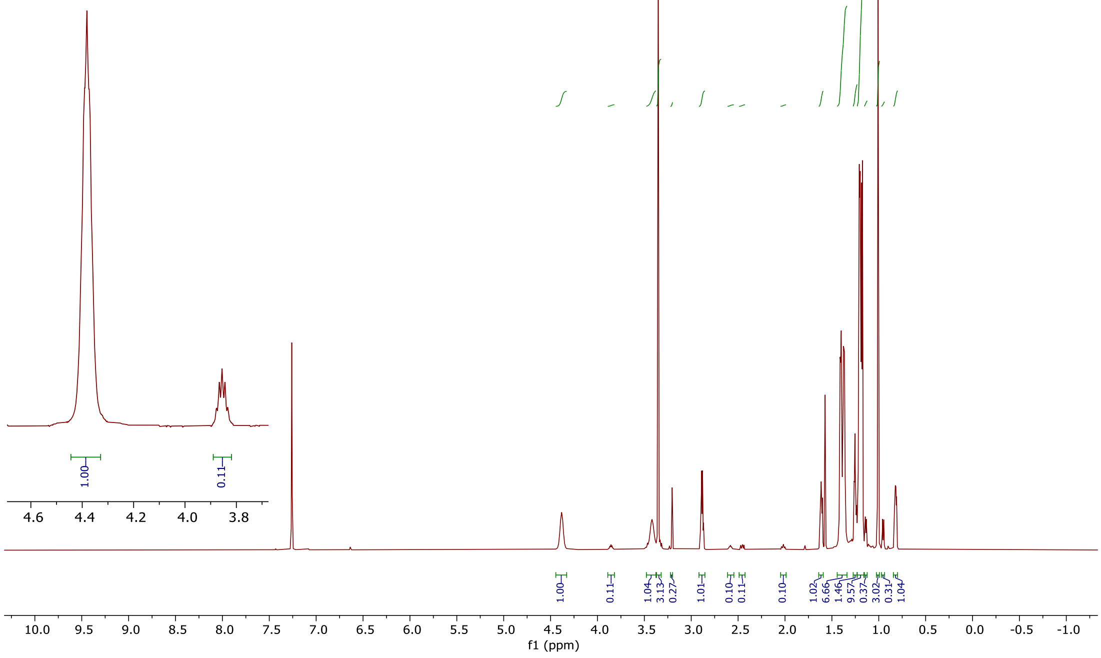

^{13}C NMR (151 MHz, Chloroform-*d*)

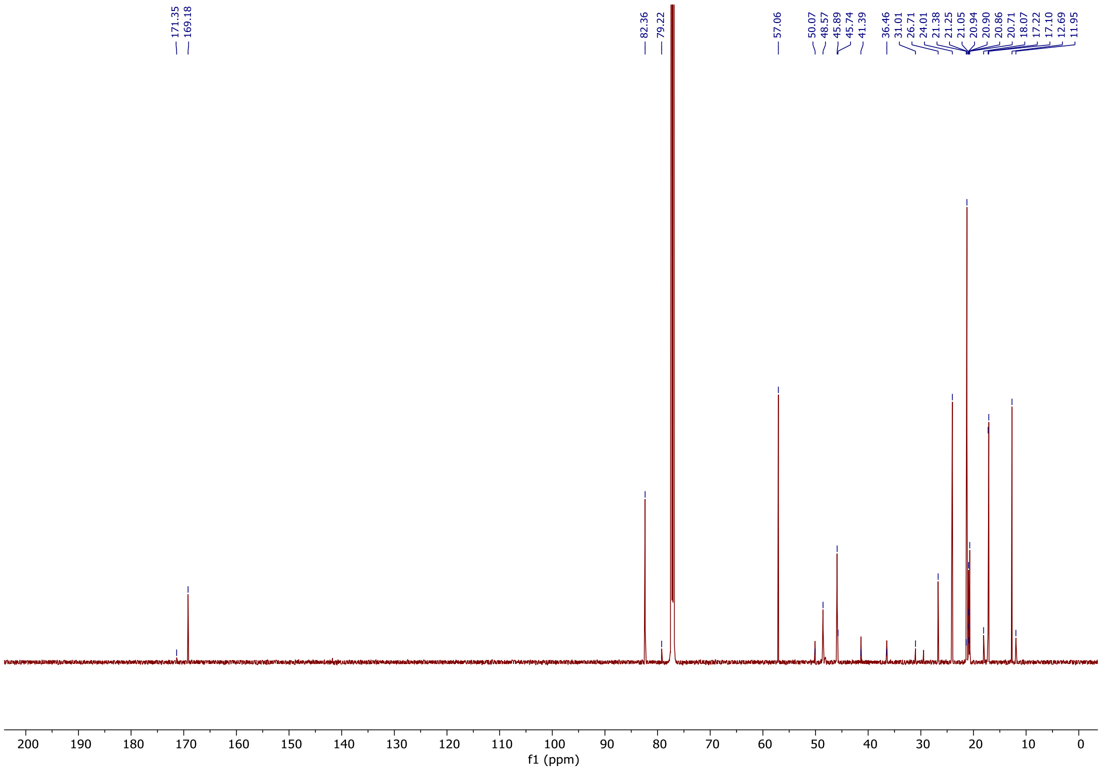

^{1}H COSY (600 MHz, Chloroform-*d*)

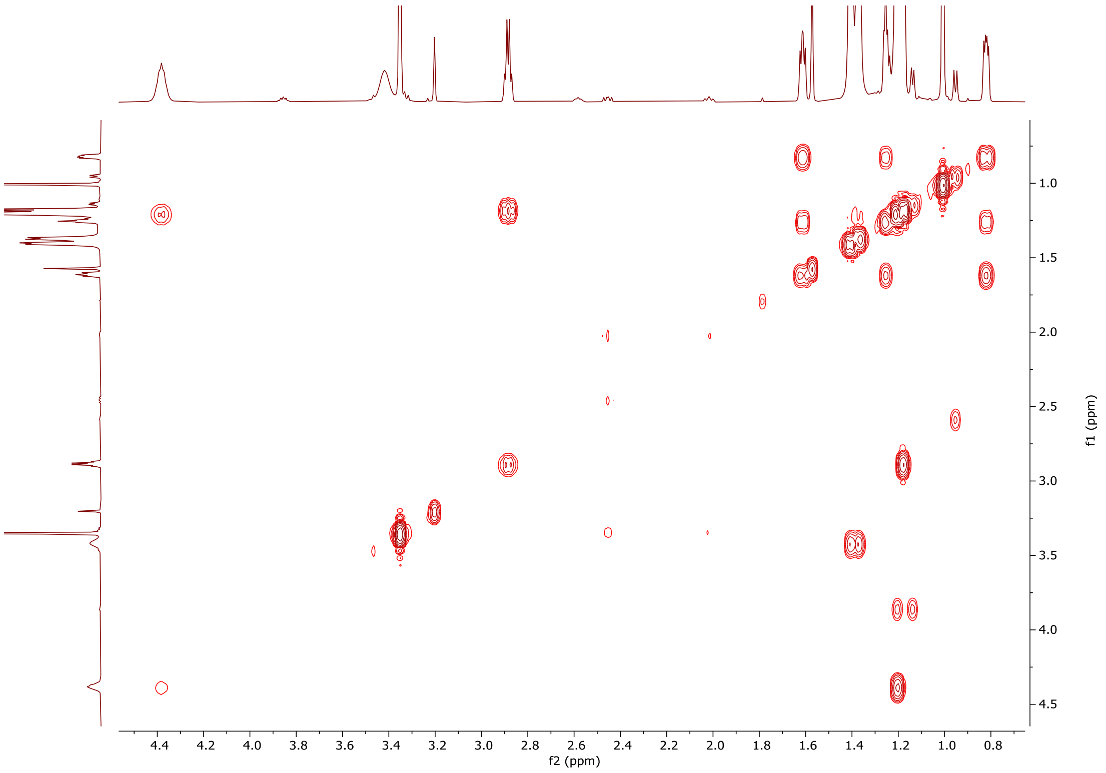

^{1}H/^{13}C HSQC (600/151 MHz, Chloroform-*d*)

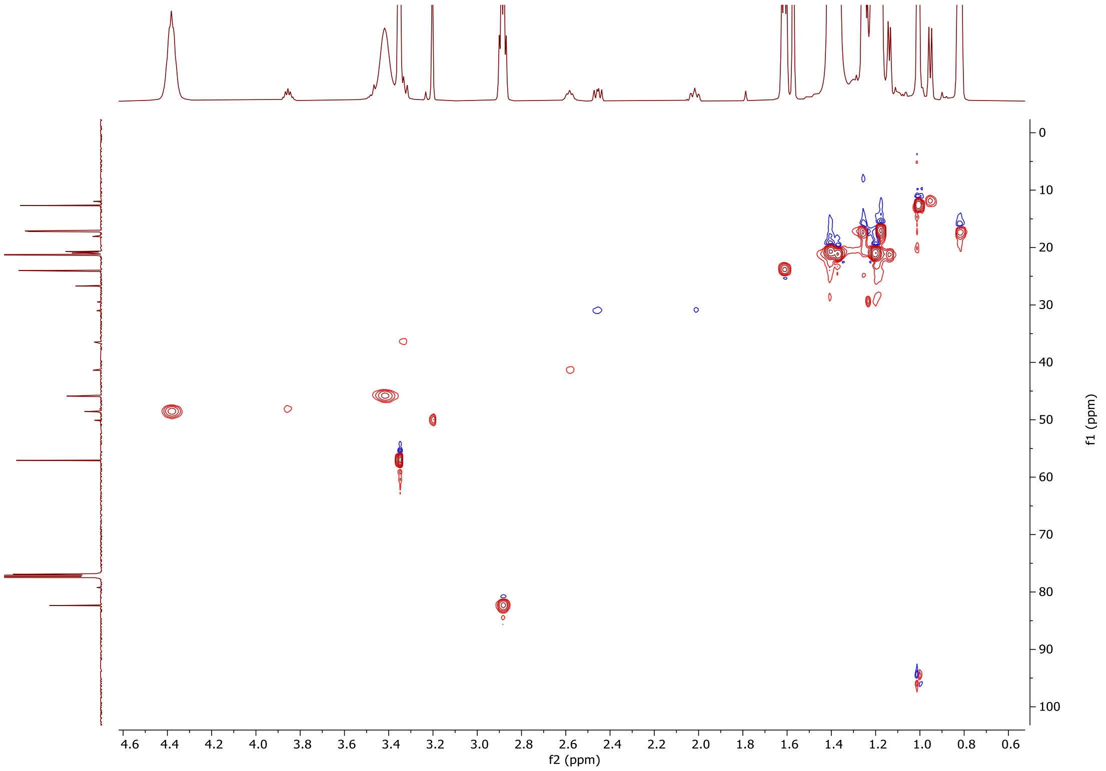

$^{1}H/^{13}C$ HMBC (600/151 MHz, Chloroform-*d*)

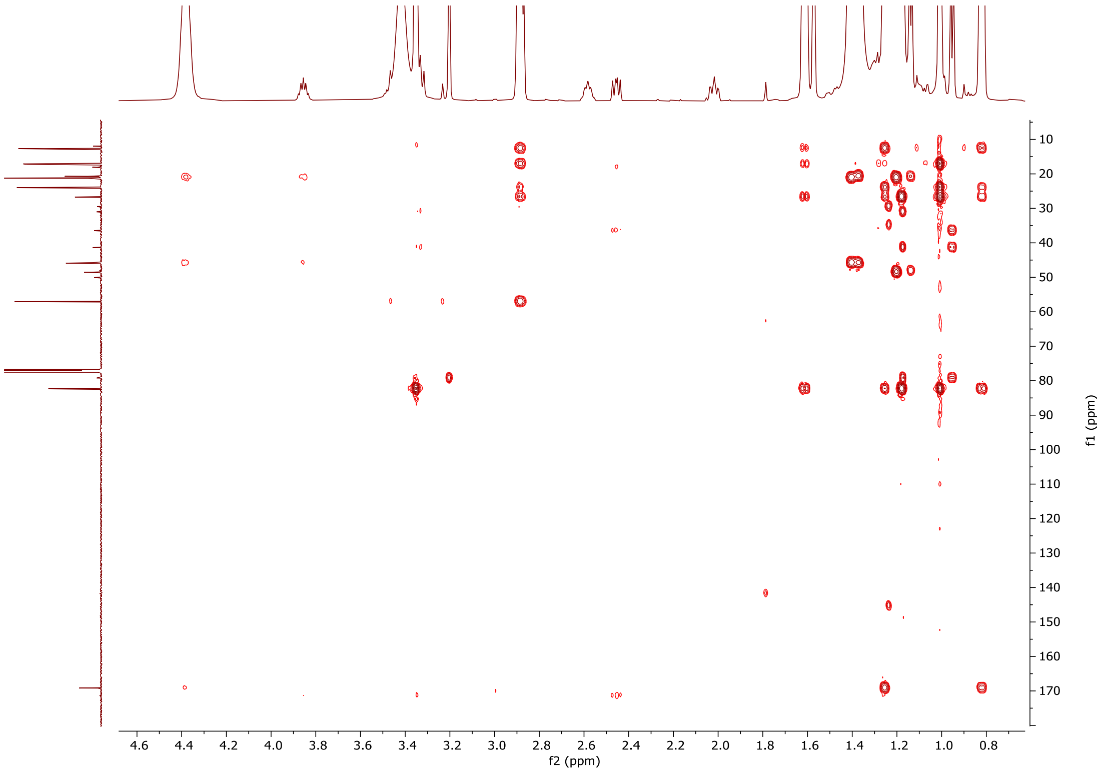

^{1}H NOSEY (600 MHz, Chloroform-*d*)

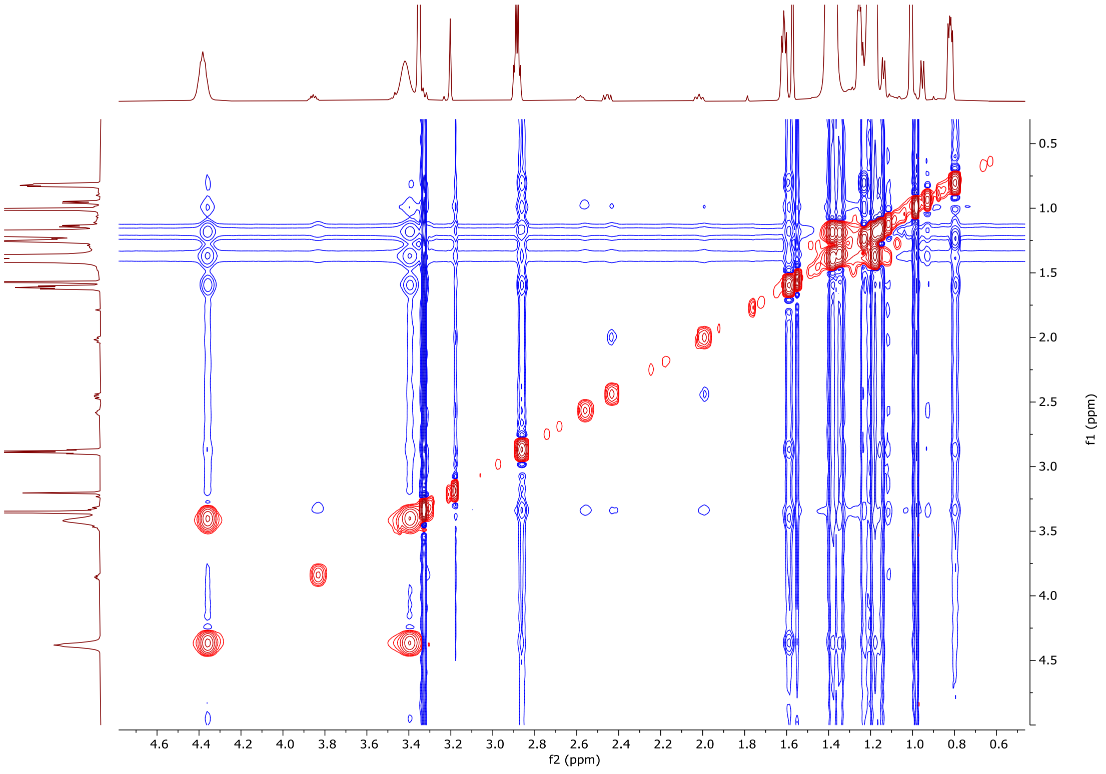

(1*R,2*R**)-*N*,*N*-diisopropyl-2-((*R**)-1-methoxyethyl)-2-methylcyclopropane-1-carboxamide, (*R**)-3ad**

(1*R,2*R**,3*R**)-*N*,*N*-diisopropyl-3-methoxy-2,3-dimethylcyclobutane-1-carboxamide, S7**

[1]**H NMR** (600 MHz, Chloroform-*d*) **contains BHT*

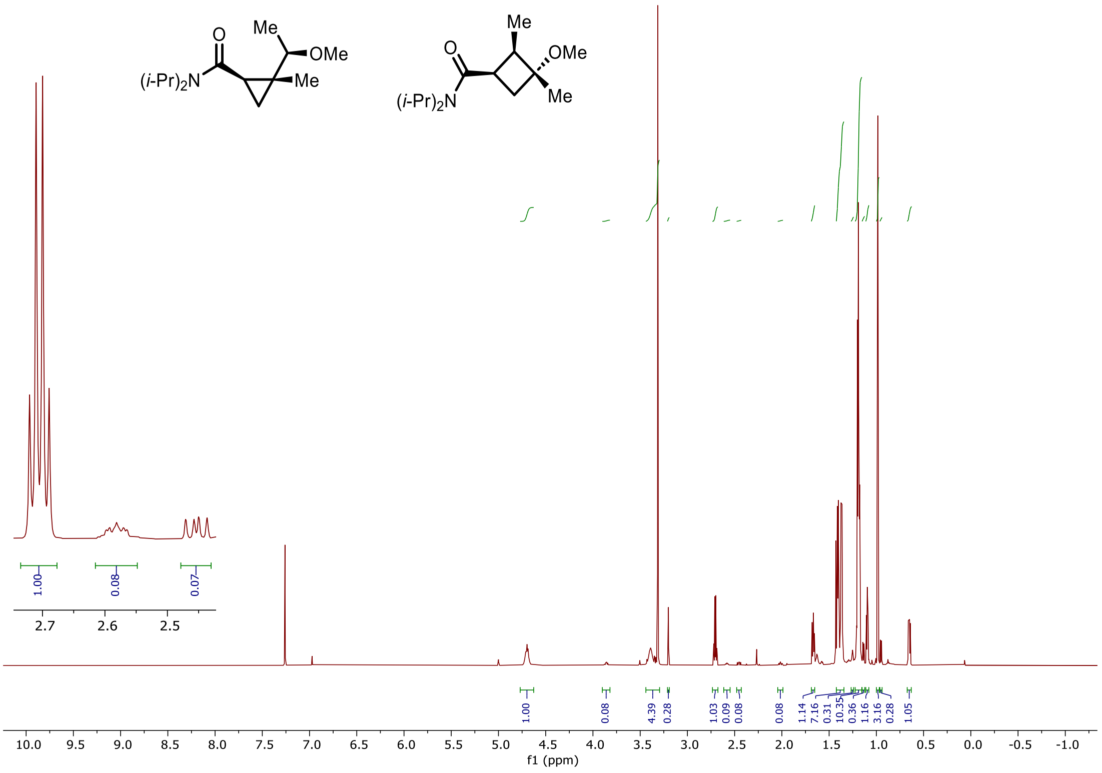

[13]**C NMR** (151 MHz, Chloroform-*d*) **contains BHT*

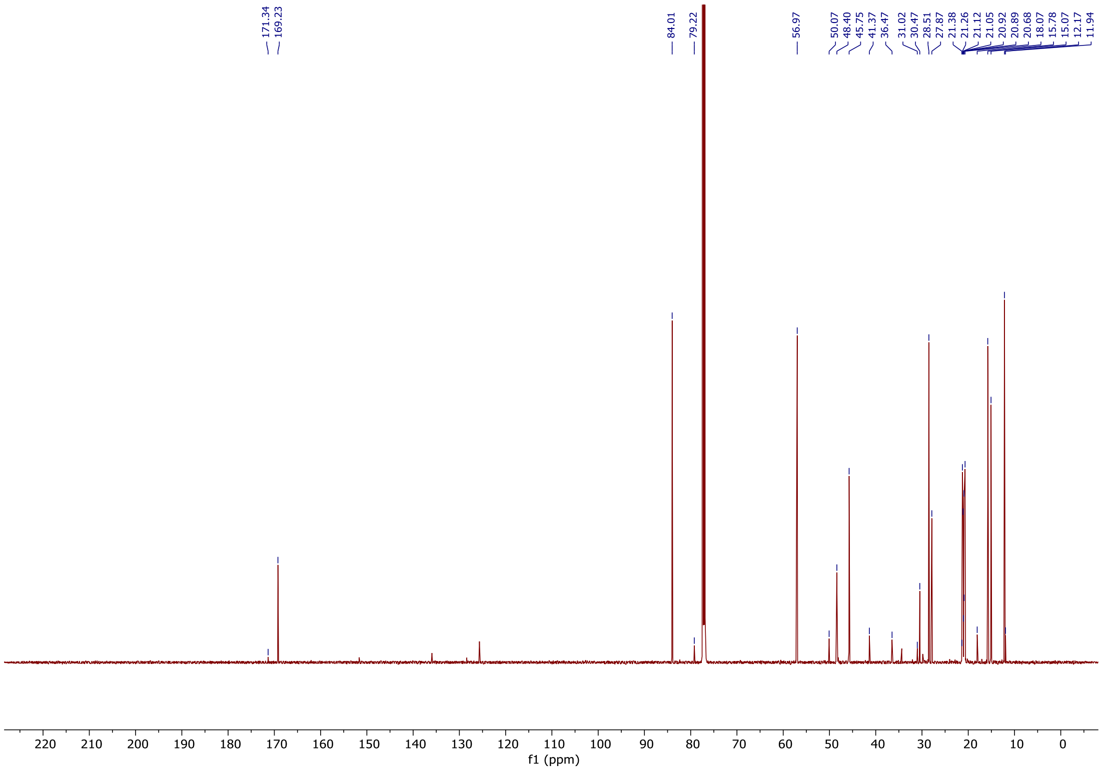

^{1}H COSY (600 MHz, Chloroform-*d*) **contains BHT*

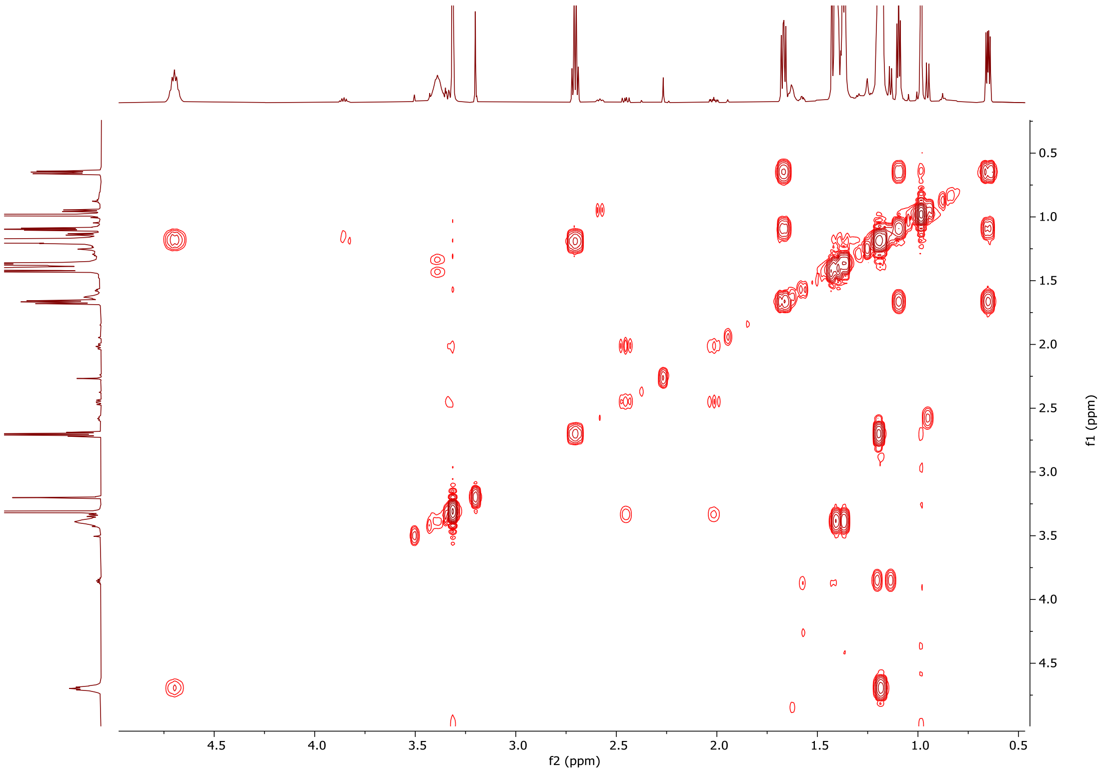

^{1}H/^{13}C HSQC (600/151 MHz, Chloroform-*d*) **contains BHT*

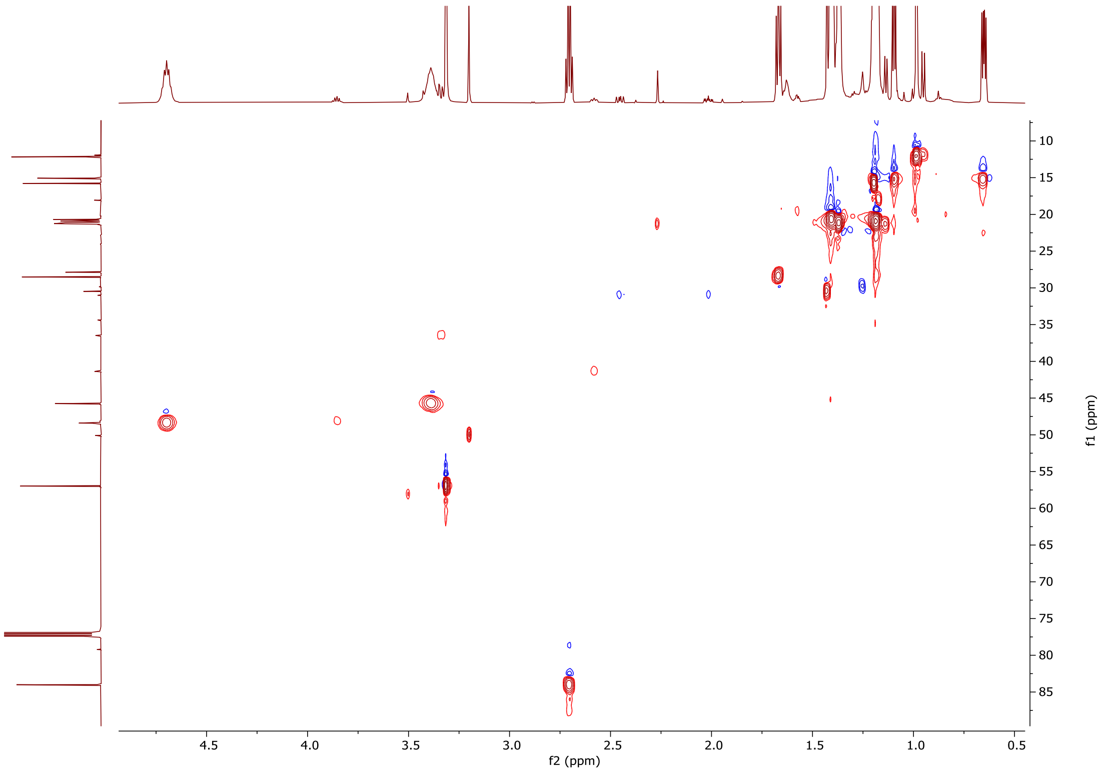

^{1}**H/**^{13}**C HMBC** (600/151 MHz, Chloroform-*d*) **contains BHT*

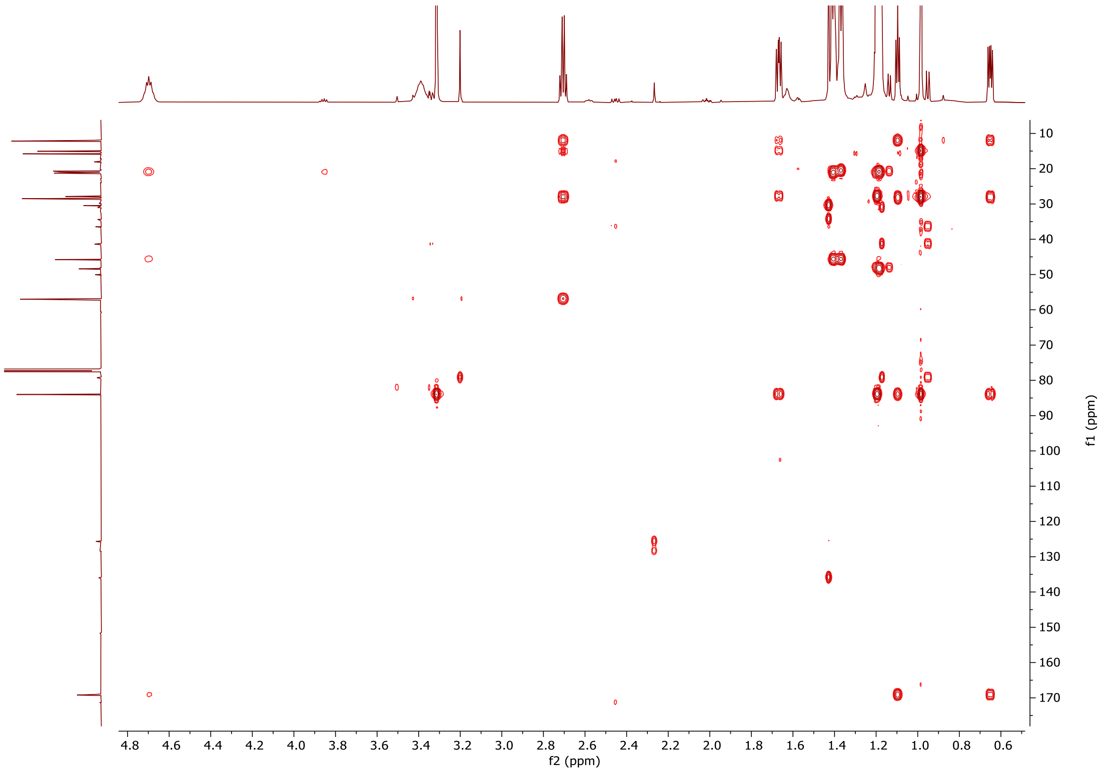

^{1}**H NOSEY** (600 MHz, Chloroform-*d*) **contains BHT*

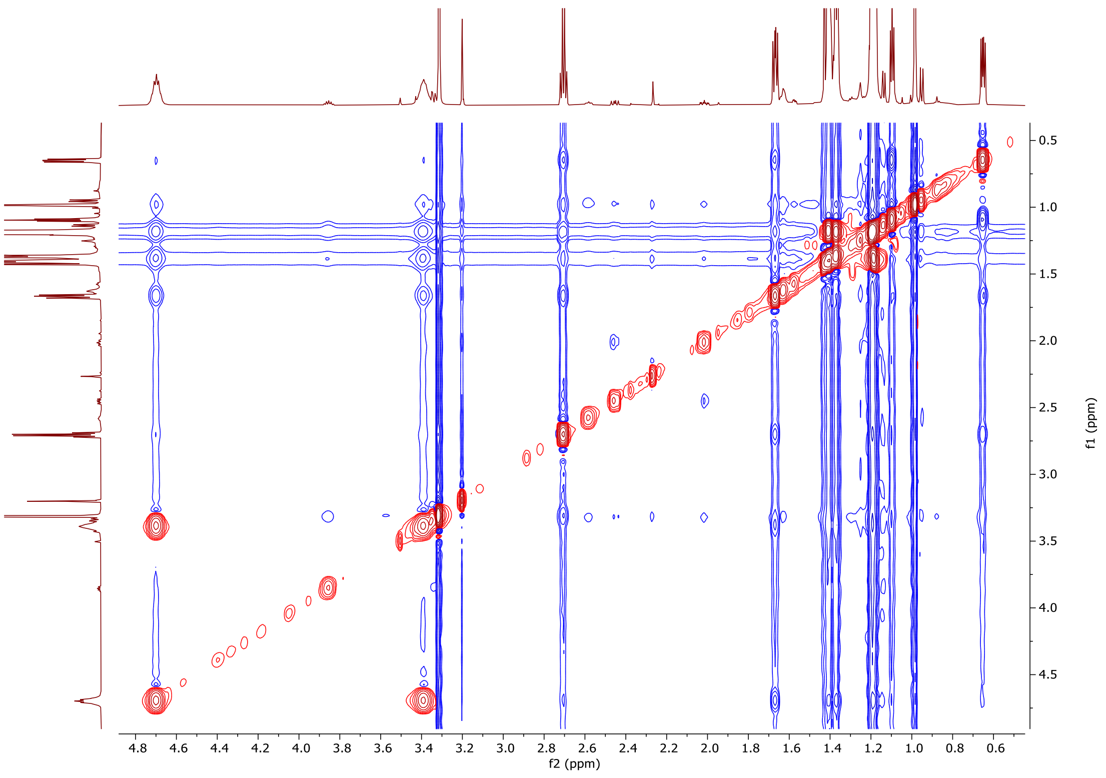

(*S)-1-((1*R**,2R*,3*S**)-2-(diisopropylcarbamoyl)-1,3-dimethylcyclopropyl)ethyl 2,2,2-trifluoroacetate, 3ae**

^{1}H NMR (600 MHz, Chloroform-*d*)

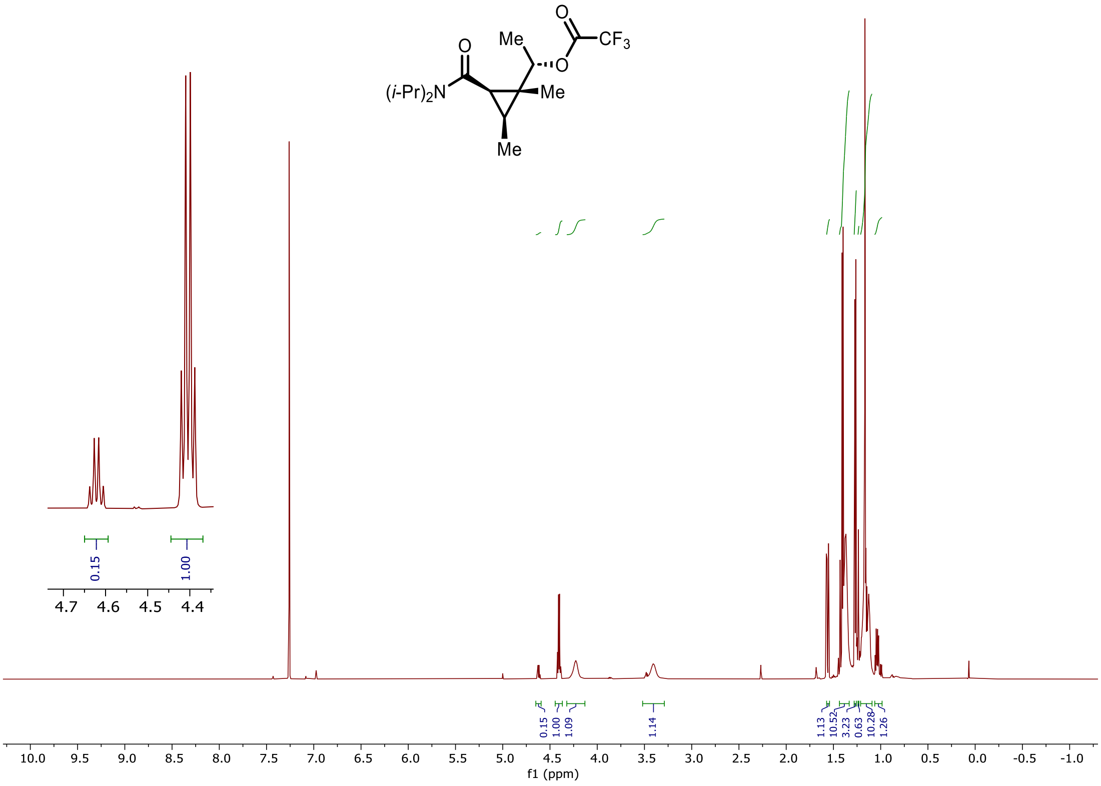

^{13}C NMR (151 MHz, Chloroform-*d*)

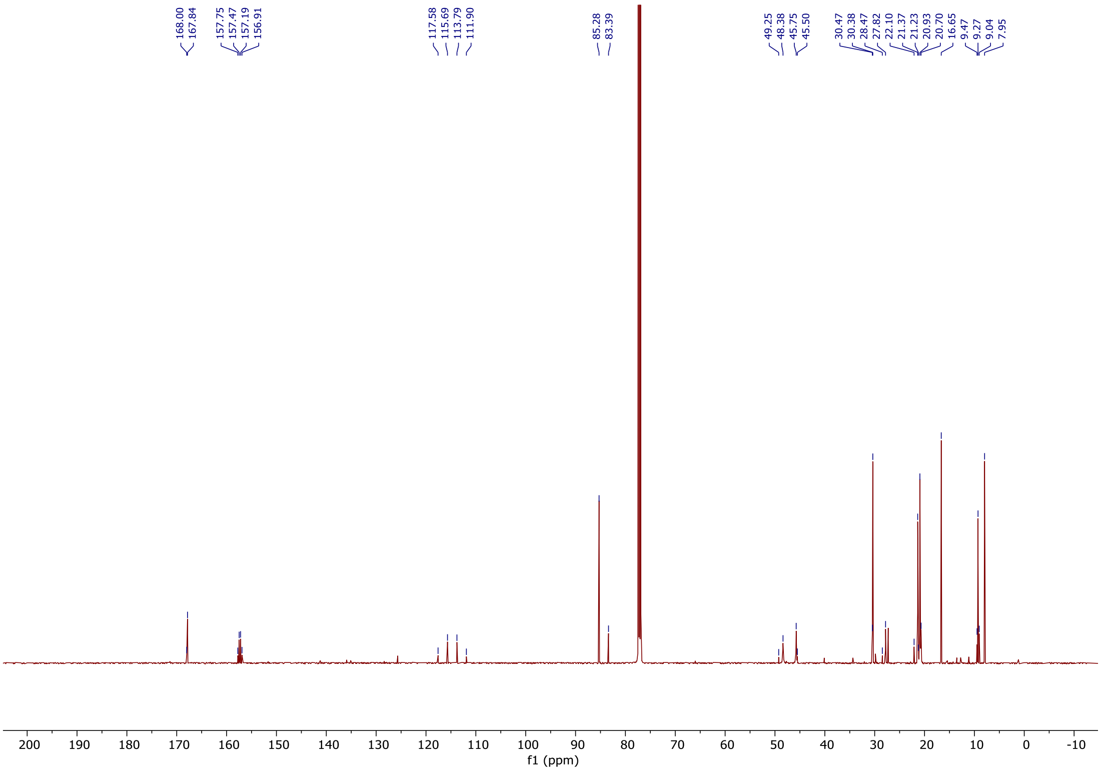

^{1}**H COSY** (600 MHz, Chloroform-*d*)

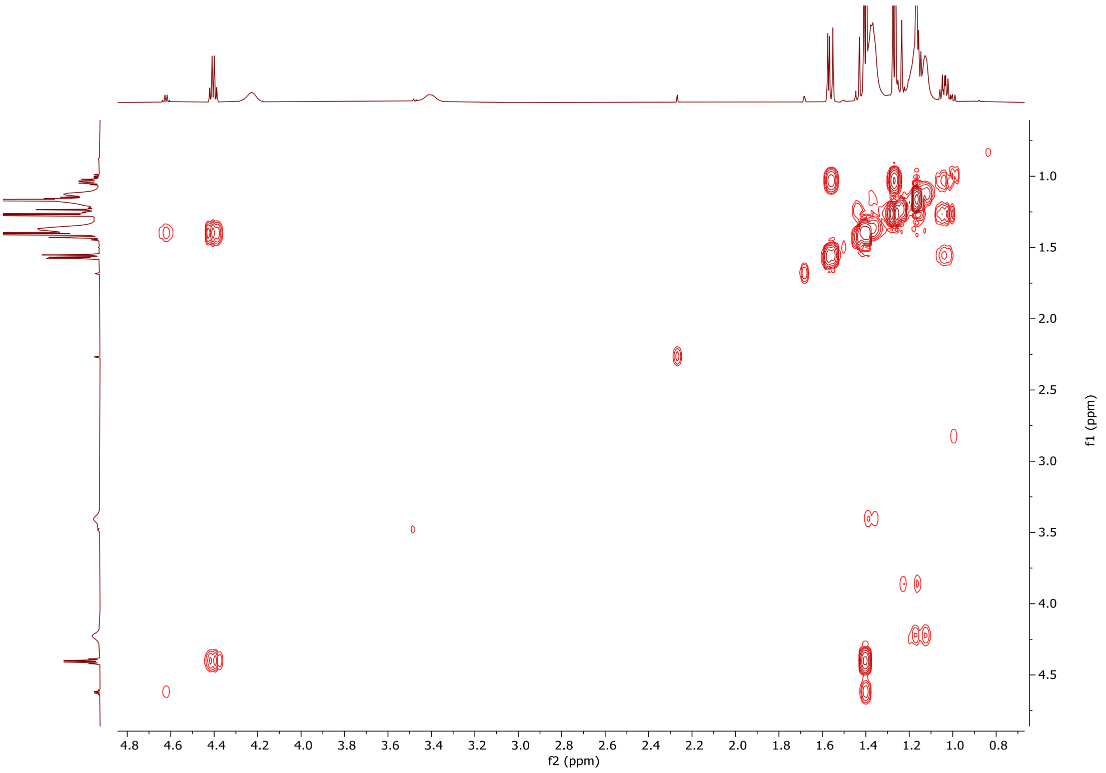

^{1}**H/**^{13}**C HSQC** (600/151 MHz, Chloroform-*d*)

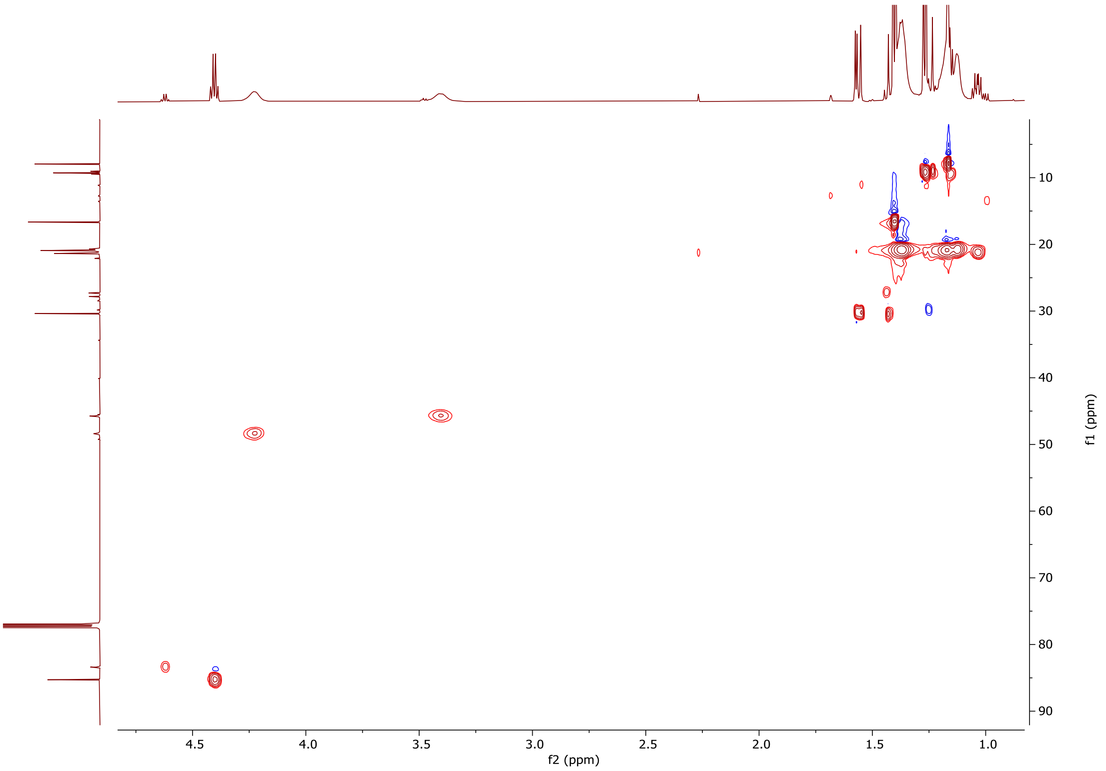

$^{1}H/^{13}C$ HMBC (600/151 MHz, Chloroform-*d*)

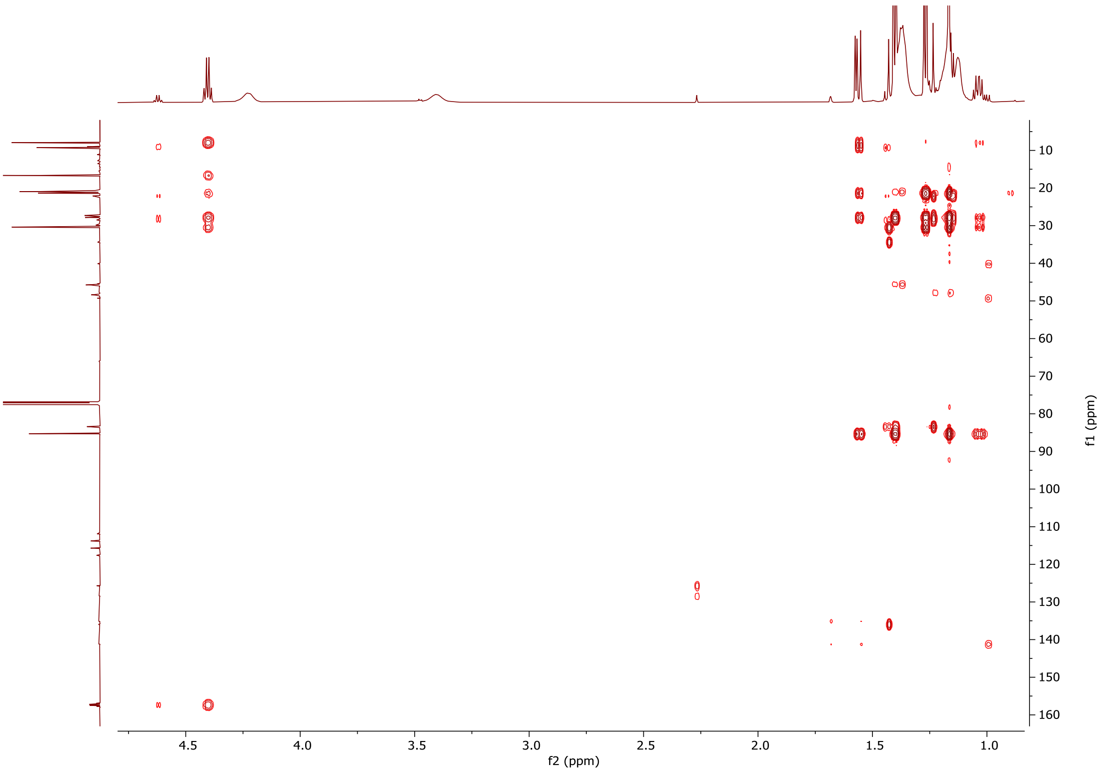

^{1}H NOSEY (600 MHz, Chloroform-*d*)

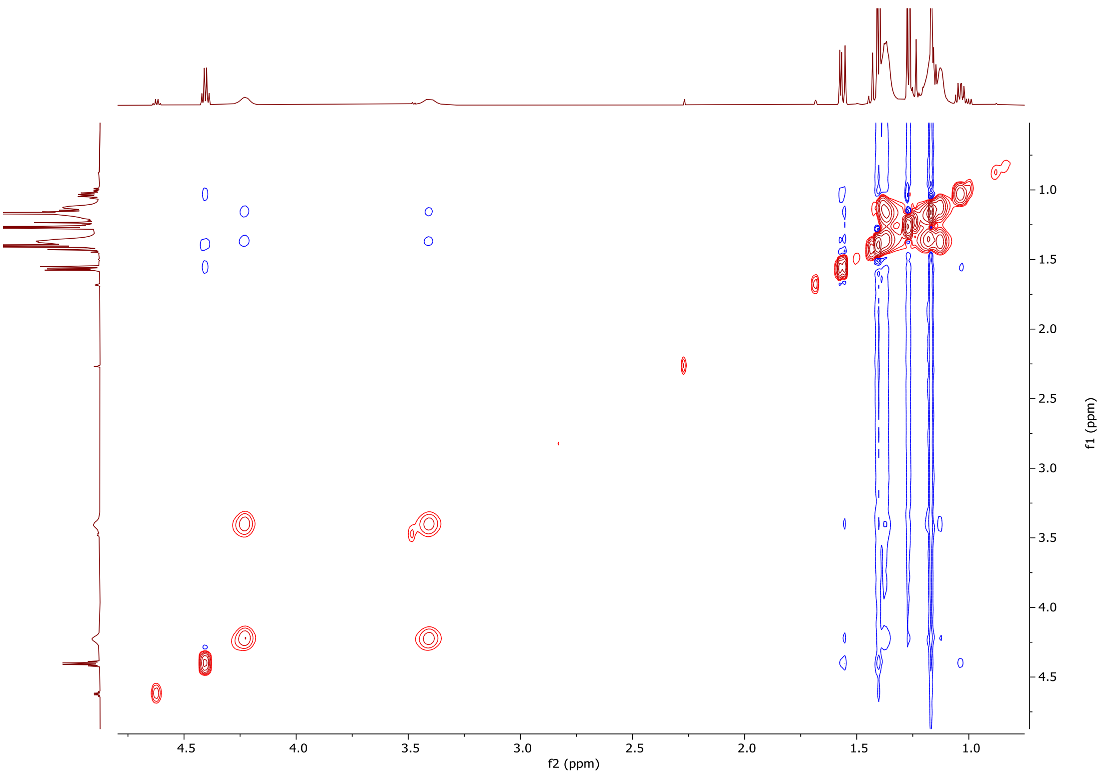

^{19}F NMR (565 MHz, Chloroform-*d*)

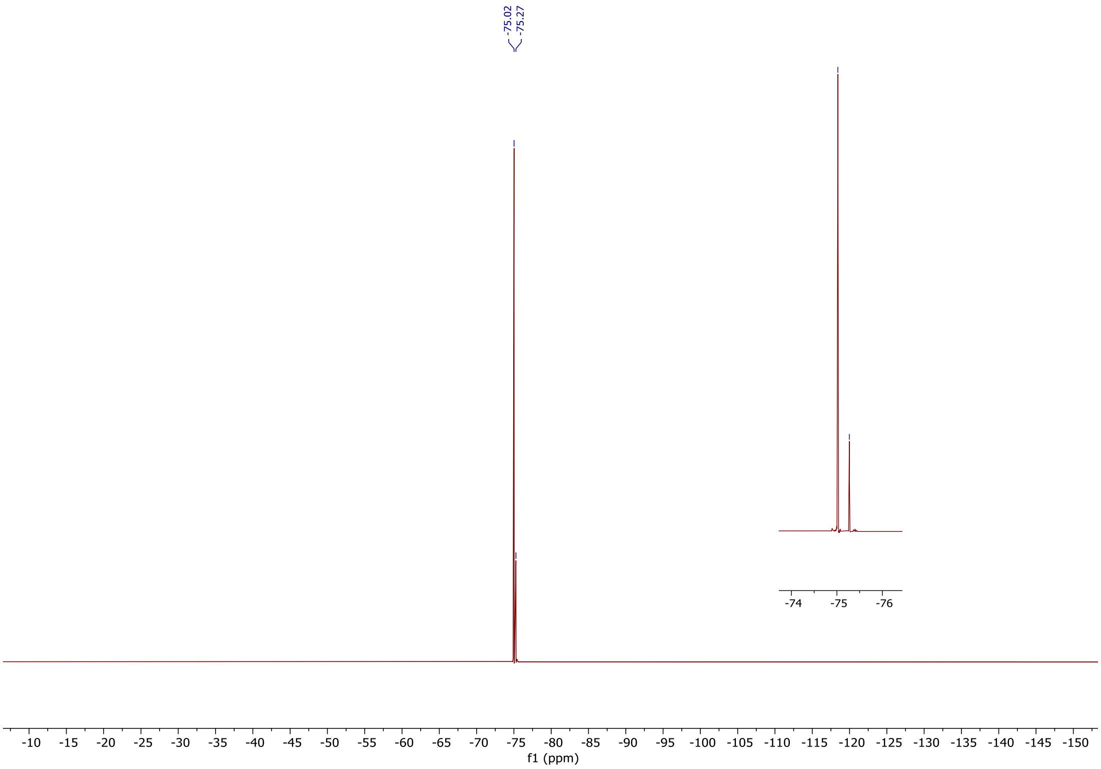

(*S)-1-((1*R**,2*R**)-2-(diisopropylcarbamoyl)cyclopropyl)ethyl benzoate, 3af**

1-(1-(diisopropylcarbamoyl)cyclopropyl)ethyl benzoate, 3ag

^{1}H NMR (600 MHz, Chloroform-*d*)

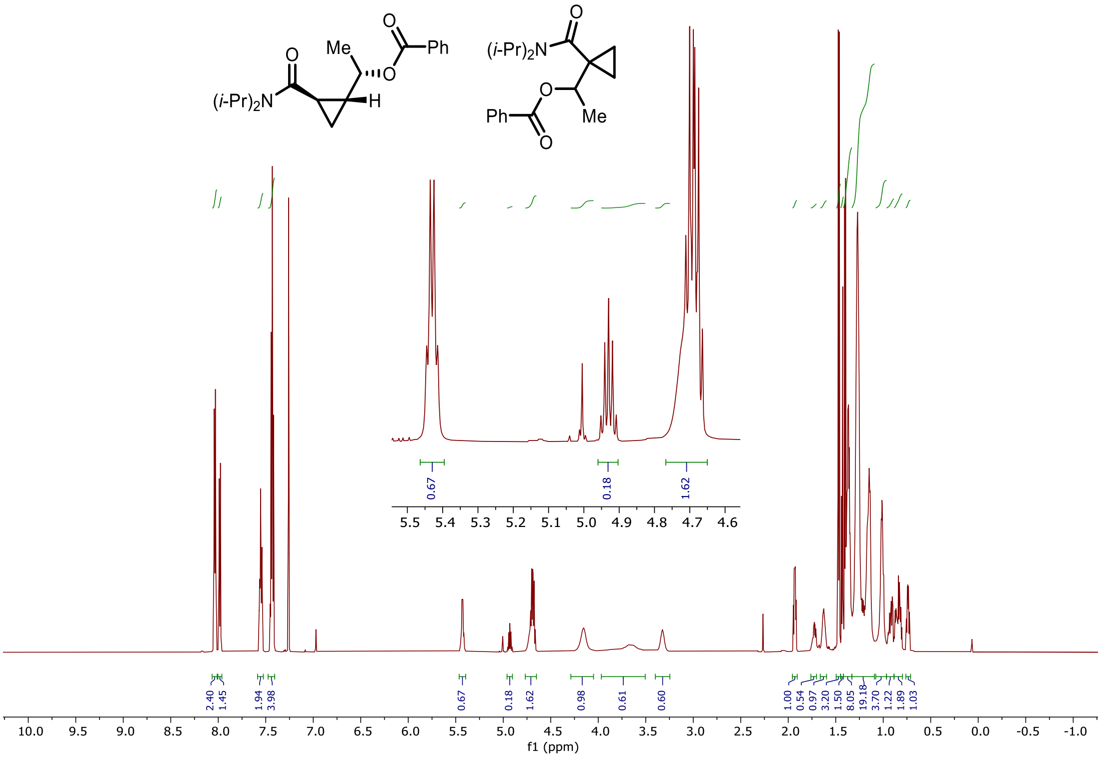

^{13}C NMR (151 MHz, Chloroform-*d*)

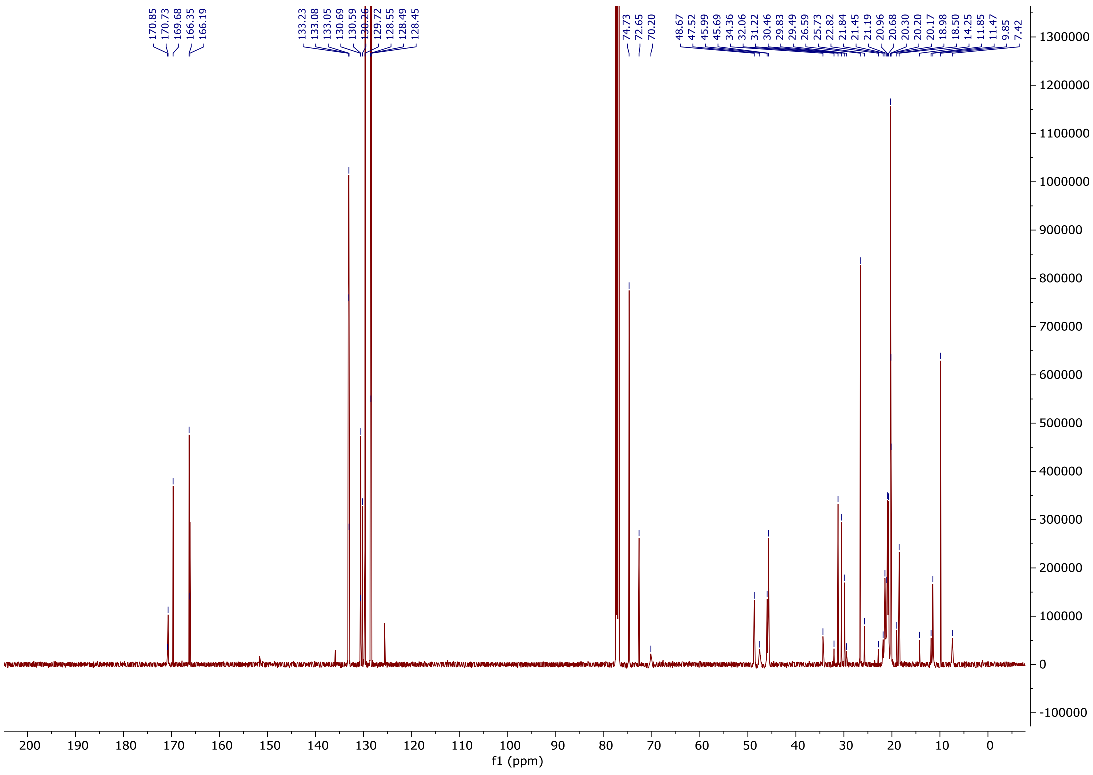

^{1}H COSY (600 MHz, Chloroform-*d*)

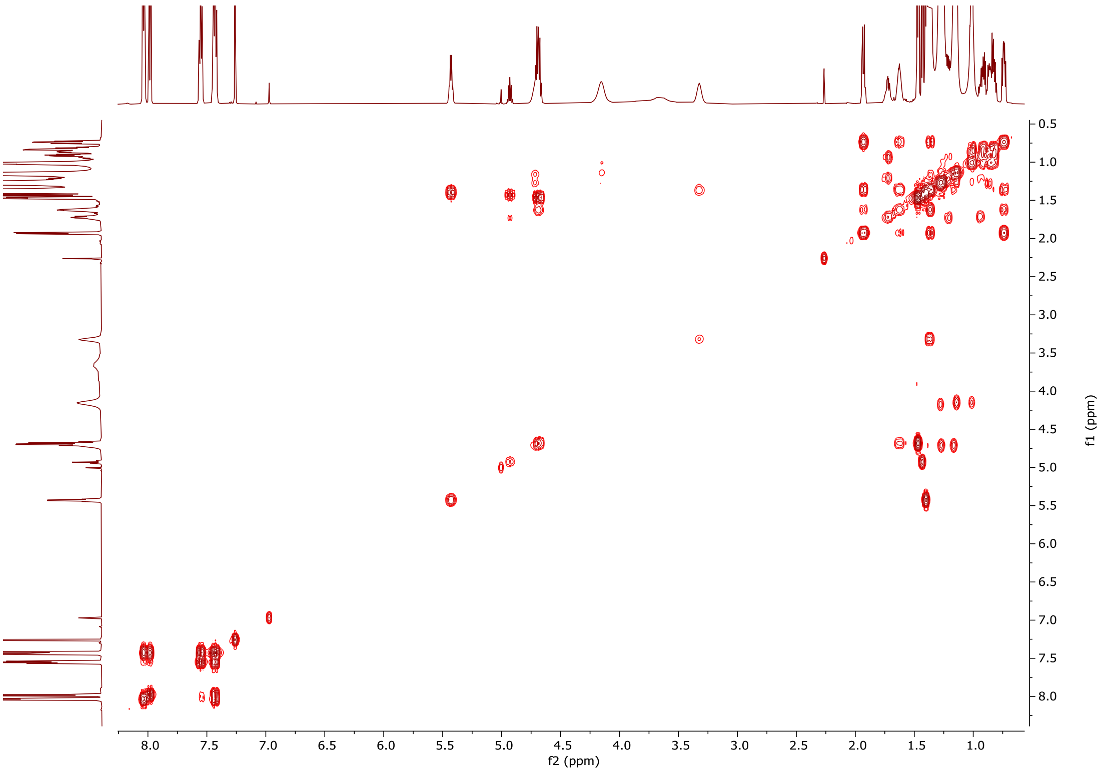

^{1}H/^{13}C HSQC (600/151 MHz, Chloroform-*d*)

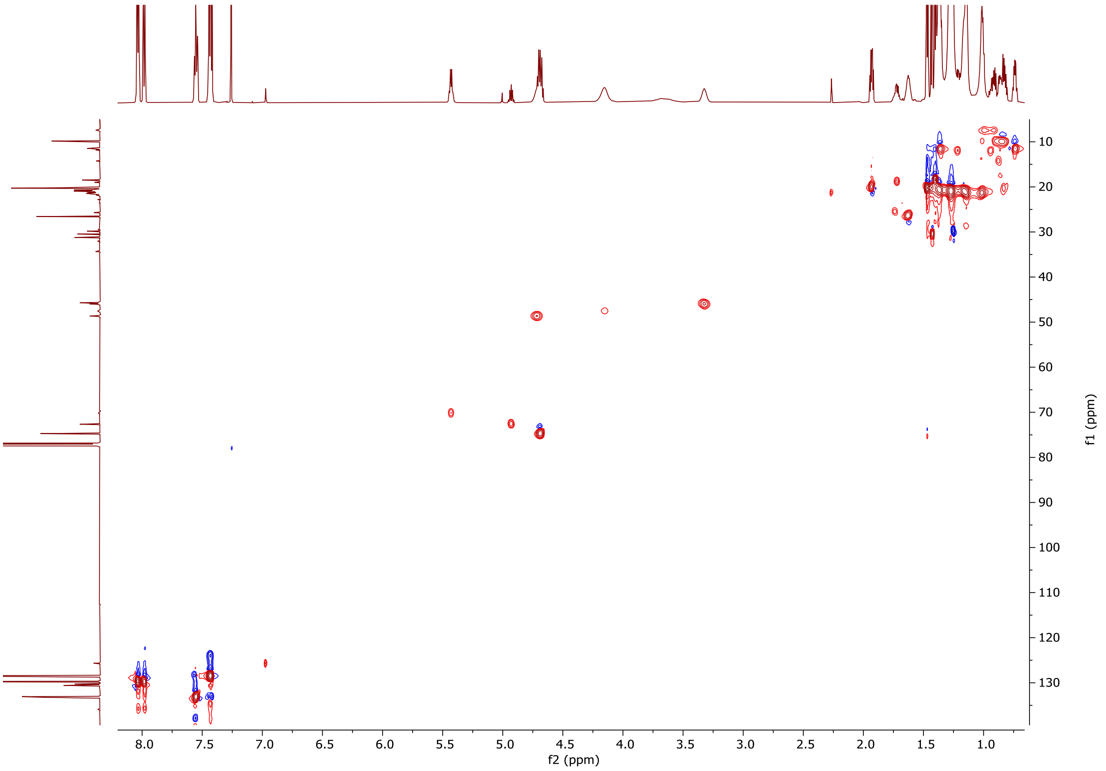

^{1}H/^{13}C HMBC (600/151 MHz, Chloroform-*d*)

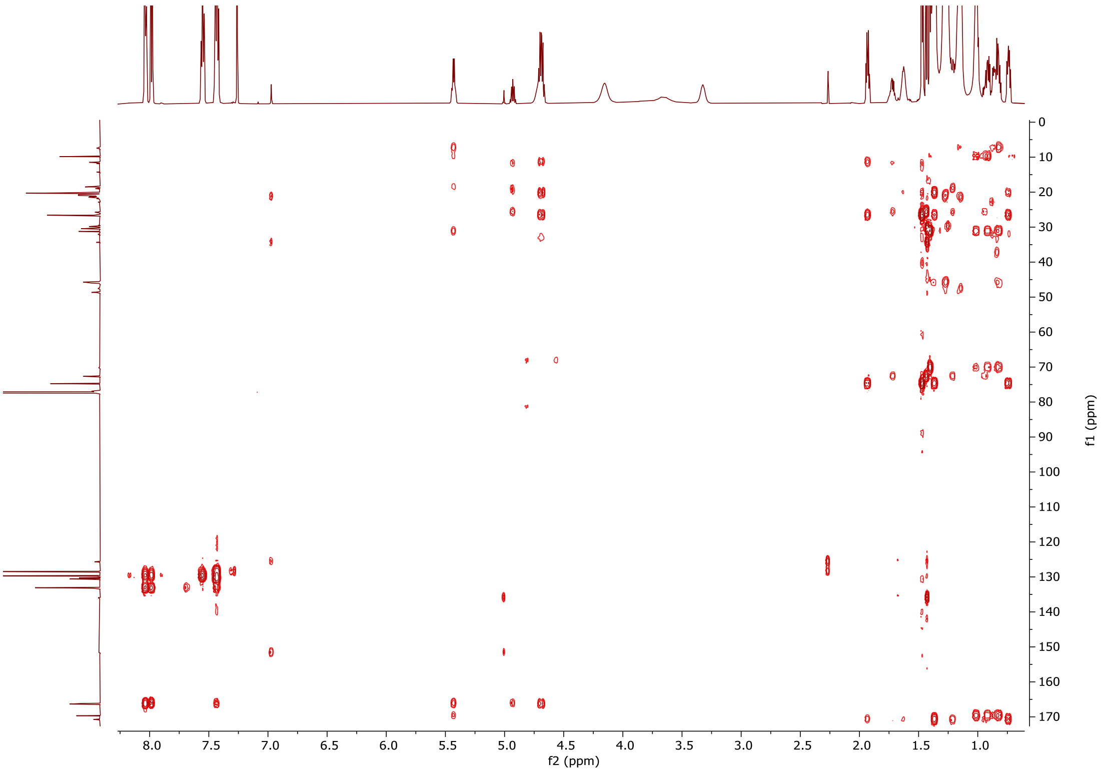

^{1}H NOSEY (600 MHz, Chloroform-*d*)

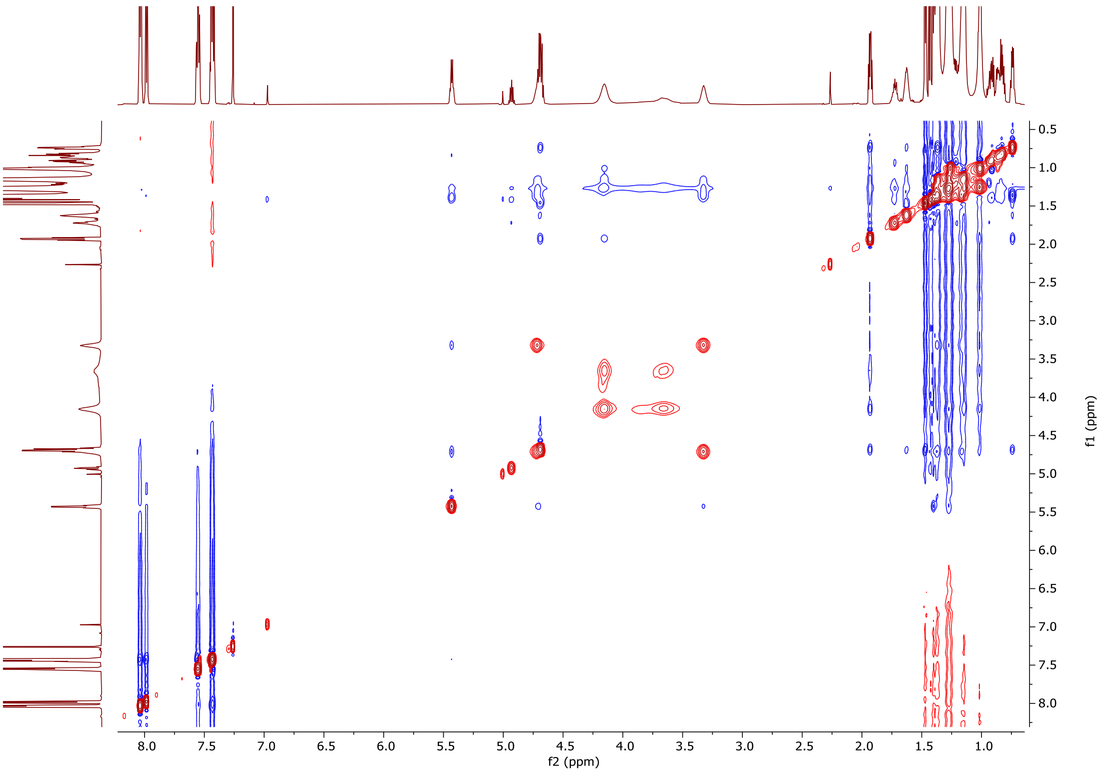

^{1}H NMR (600 MHz, Dimethylsulfoxide-*d*, 298 K)

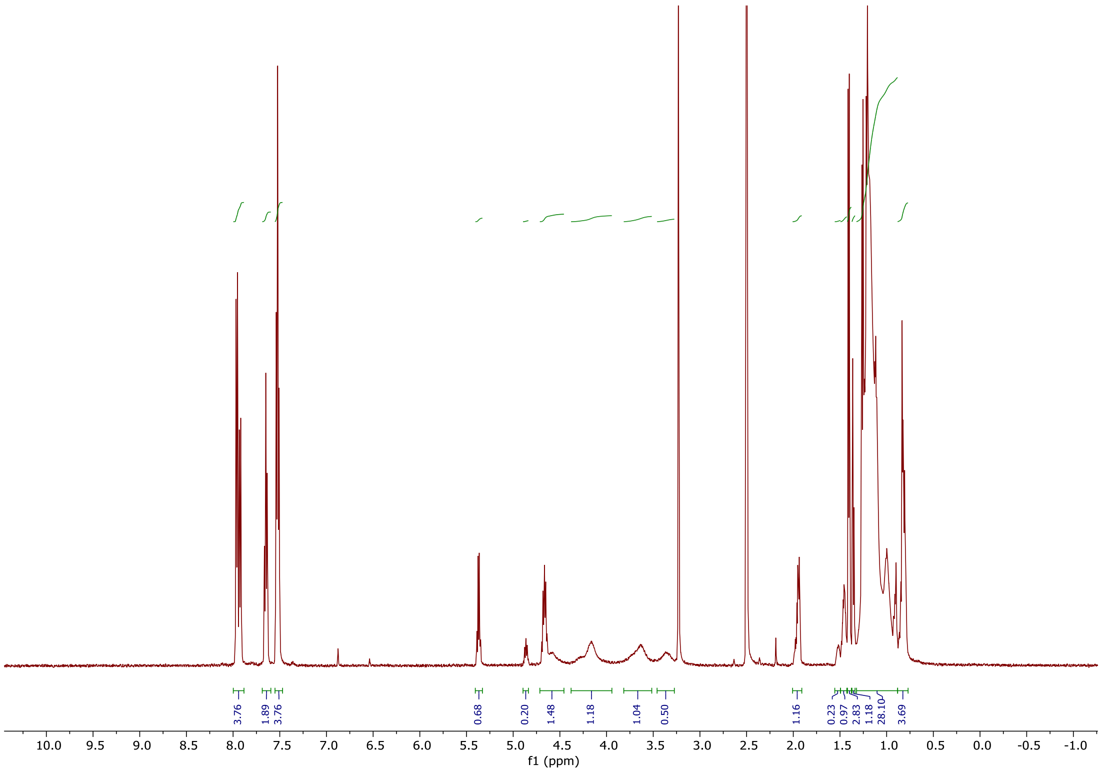

^{1}H NMR (600 MHz, Dimethylsulfoxide-*d*, 393 K)

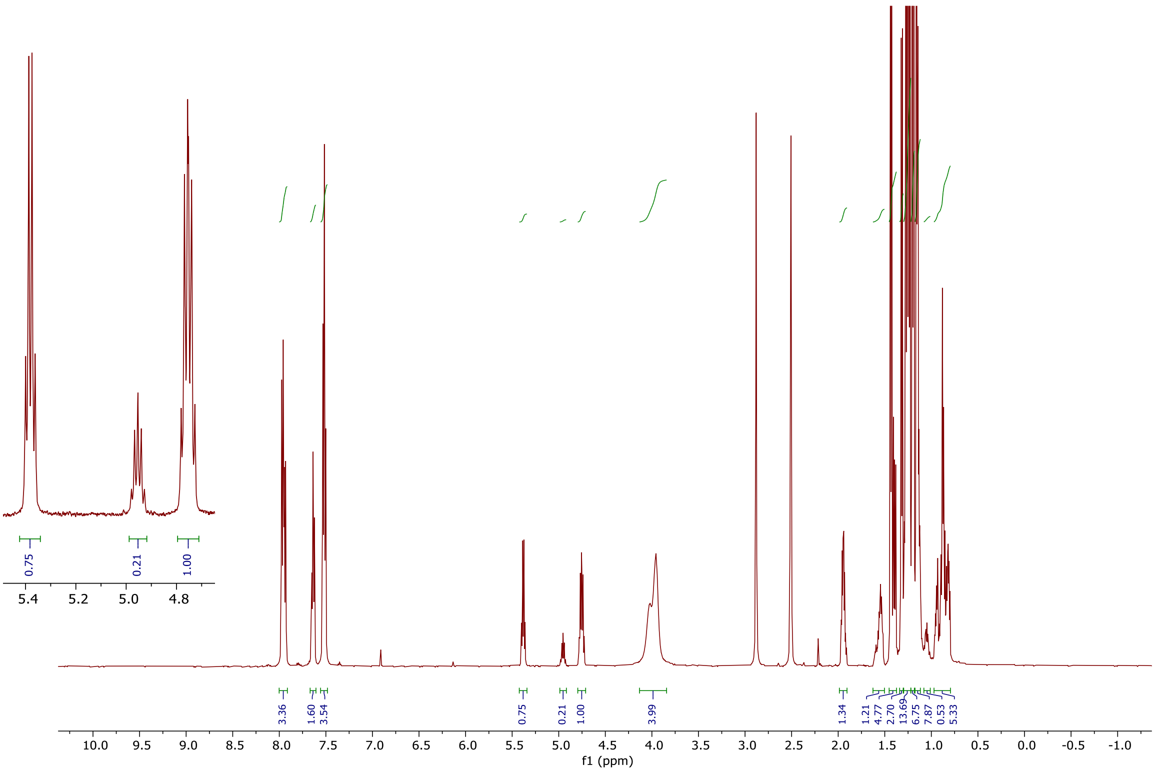

3-(diisopropylcarbamoyl)-1-methylcyclobutyl 2,2,2-trifluoroacetate, 4a

(*R)-*N*,*N*-diisopropyl-3-methylcyclobut-2-ene-1-carboxamide, S5**

^{1}H NMR (600 MHz, Chloroform-*d*)

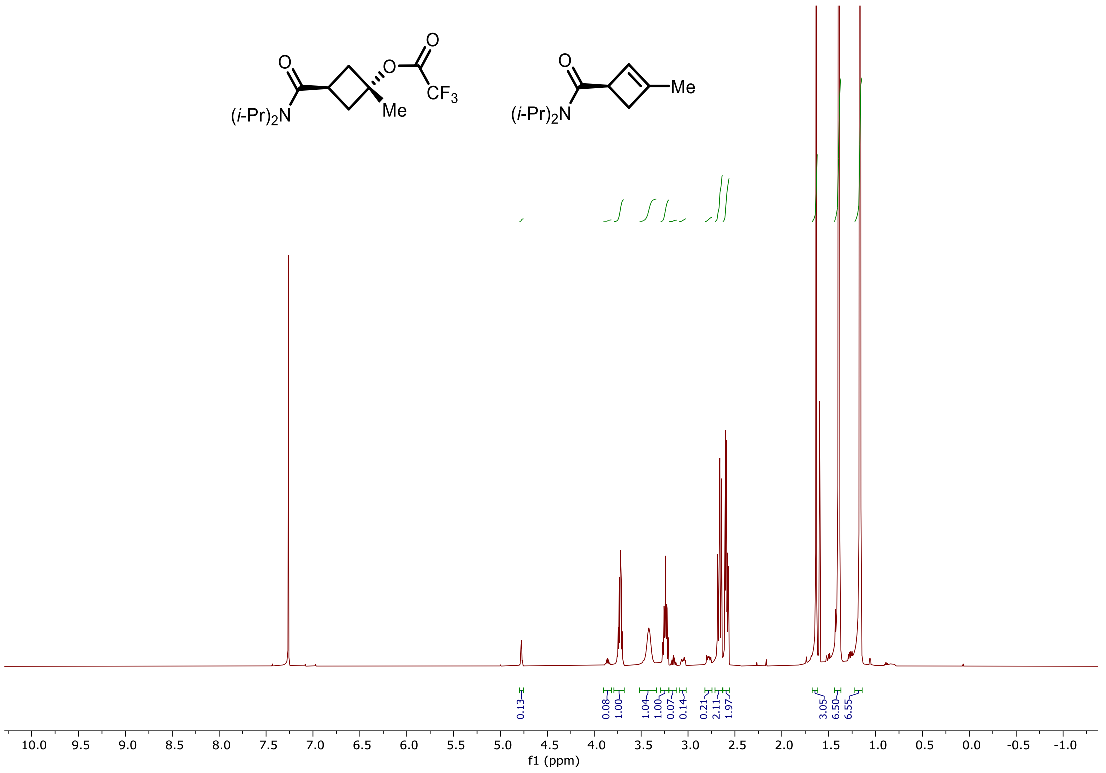

^{13}C NMR (151 MHz, Chloroform-*d*)

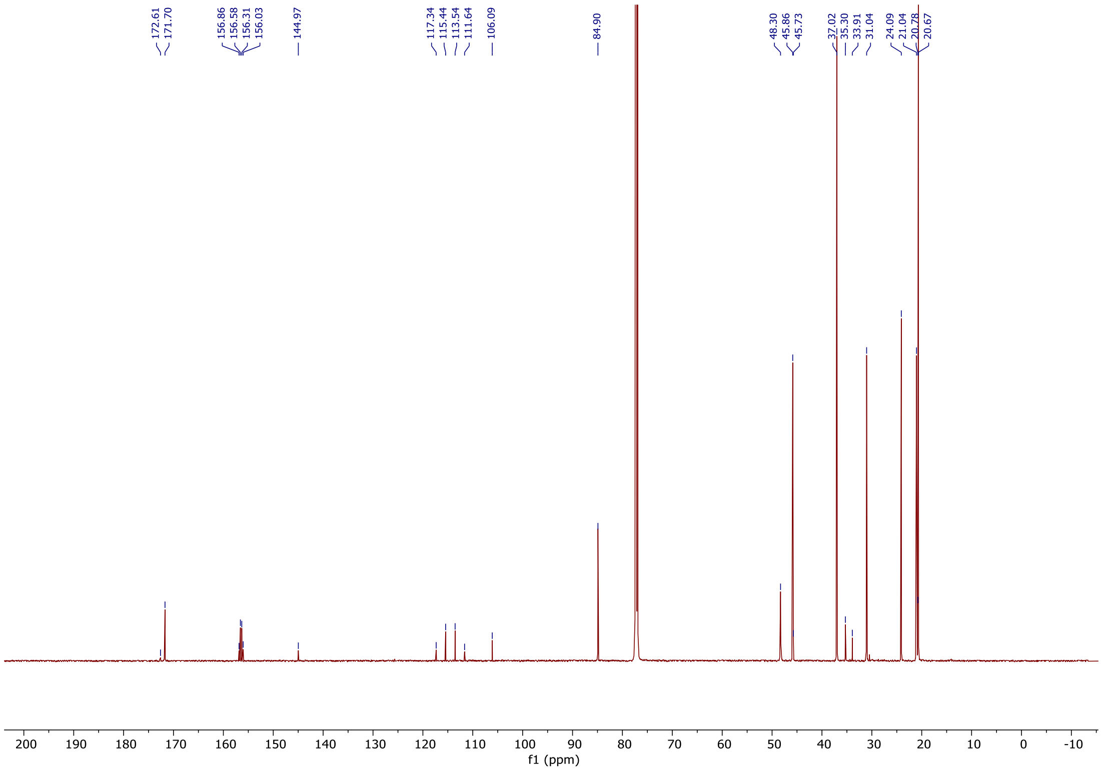

^{1}H COSY (600 MHz, Chloroform-d)

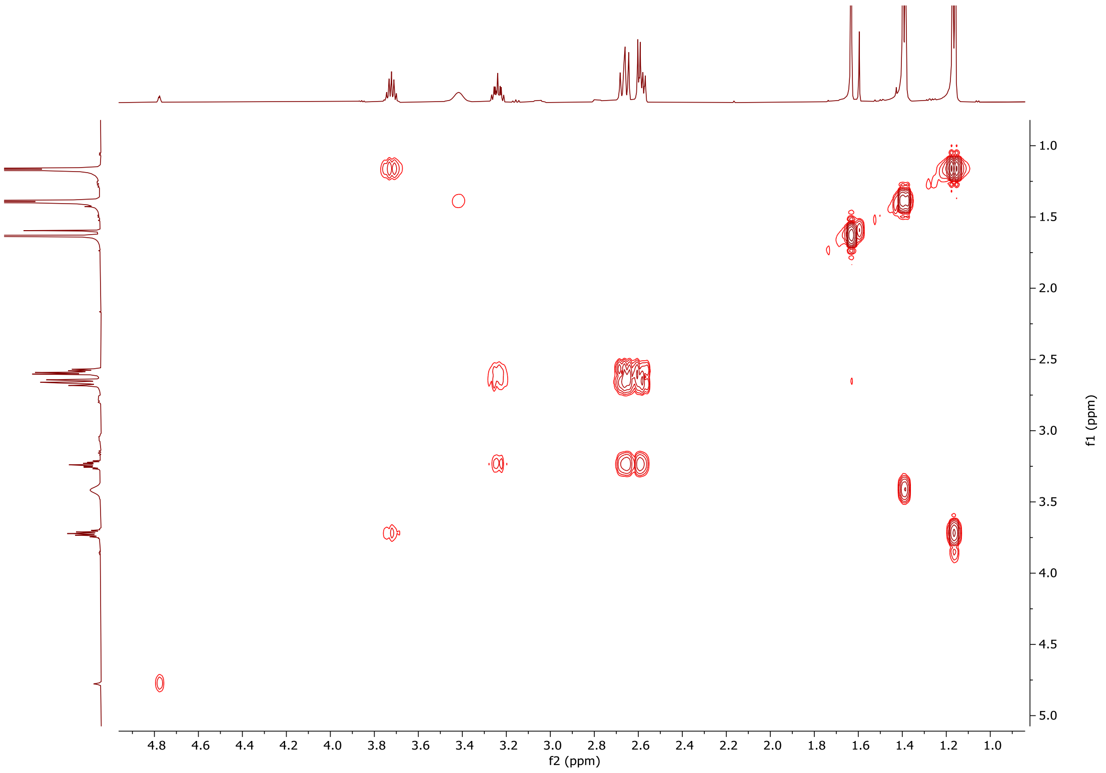

^{1}H/^{13}C HSQC (600/151 MHz, Chloroform-d)

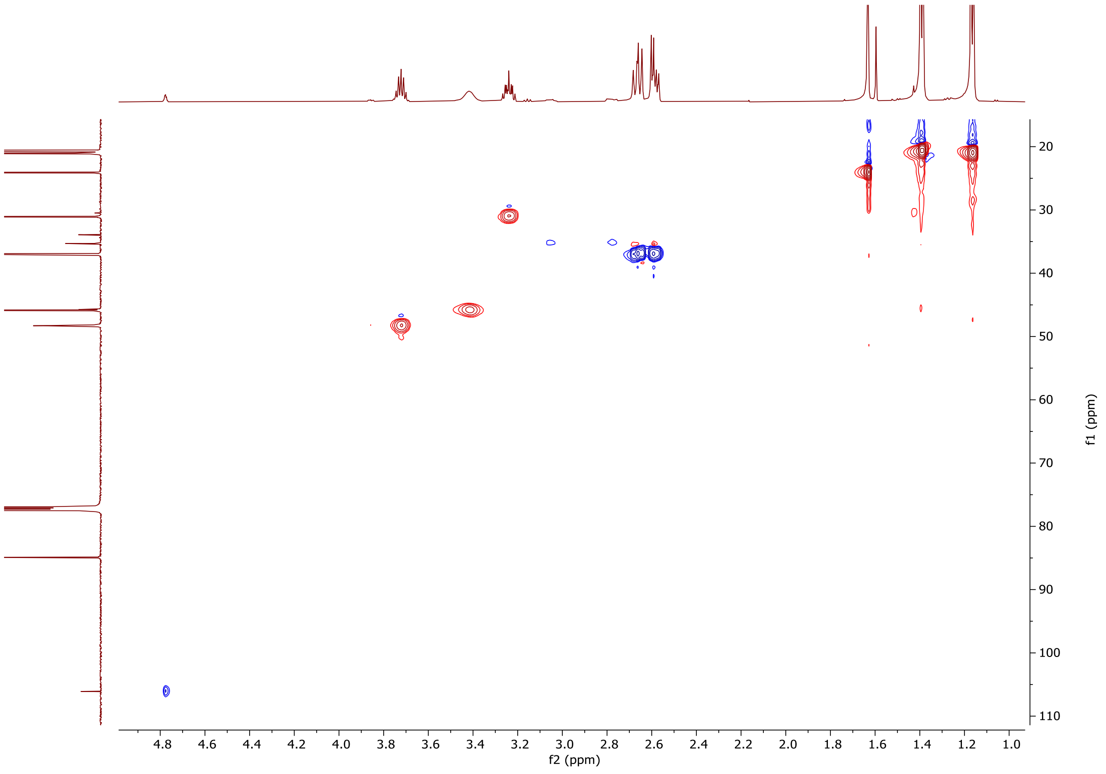

$^1H/^{13}C$ HMBC (600/151 MHz, Chloroform-*d*)

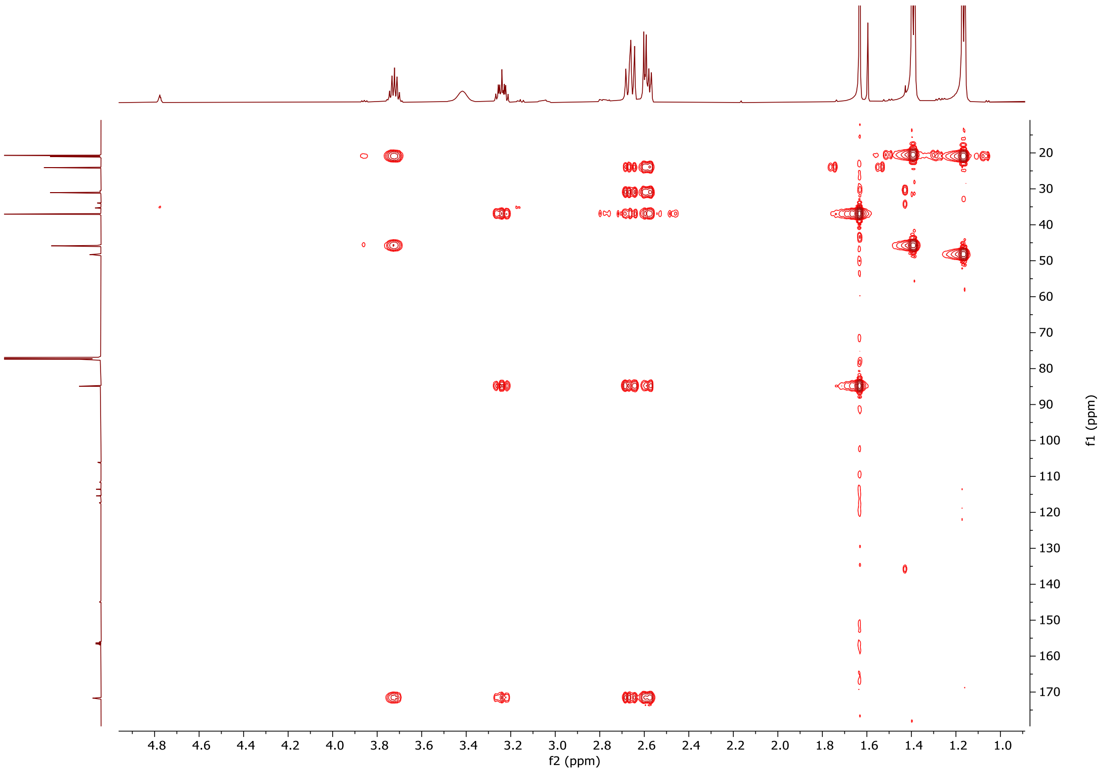

^1H NOSEY (600 MHz, Chloroform-*d*)

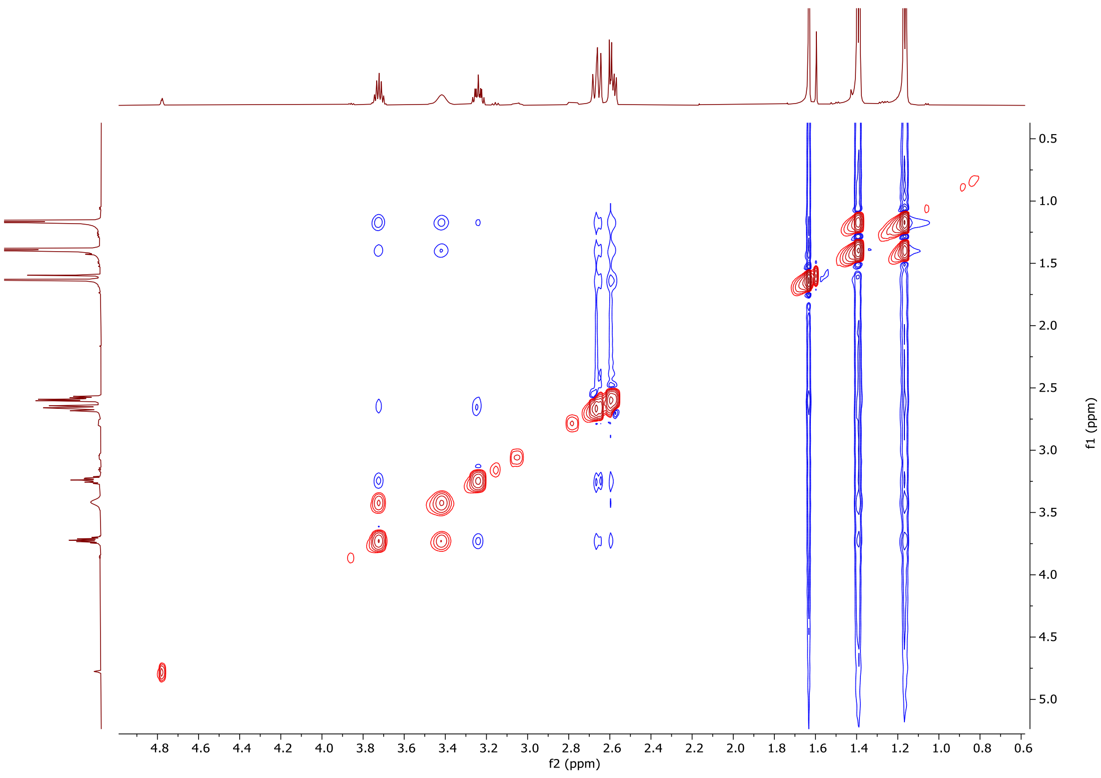

^{19}F NMR (565 MHz, Chloroform-*d*)

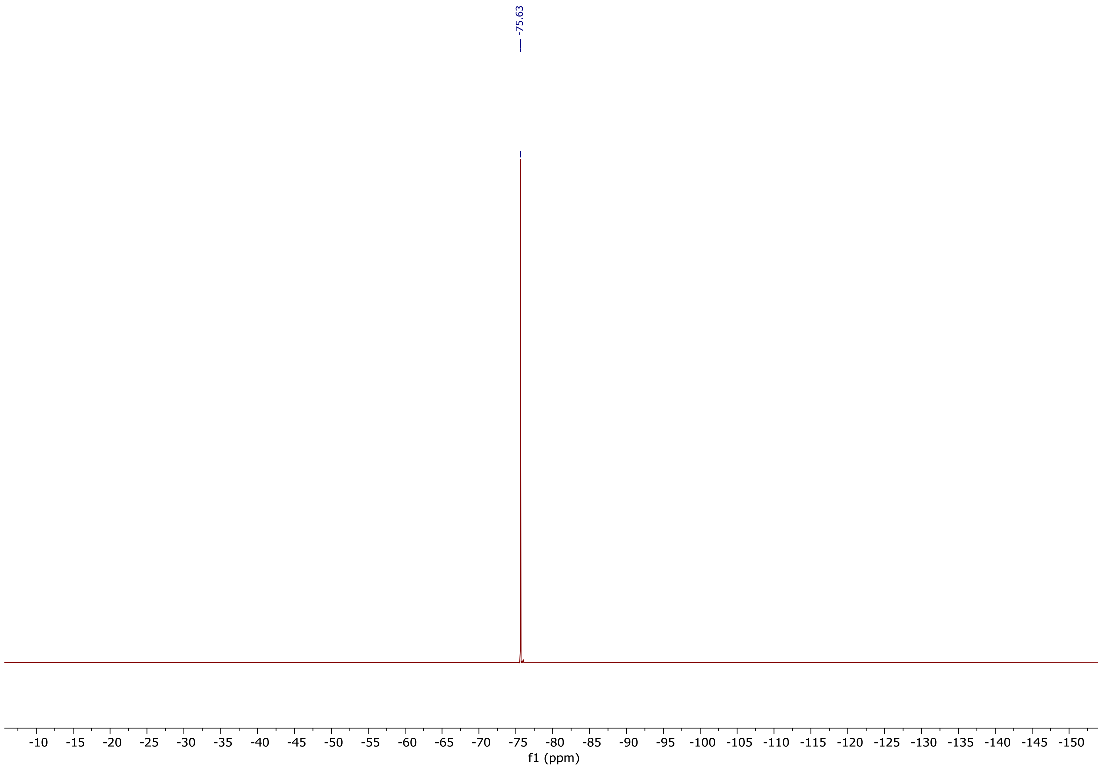

3-(diisopropylcarbamoyl)-1-(trimethylsilyl)cyclobutyl 2,2,2-trifluoroacetate, 4b

^{1}H NMR (600 MHz, Chloroform-*d*)

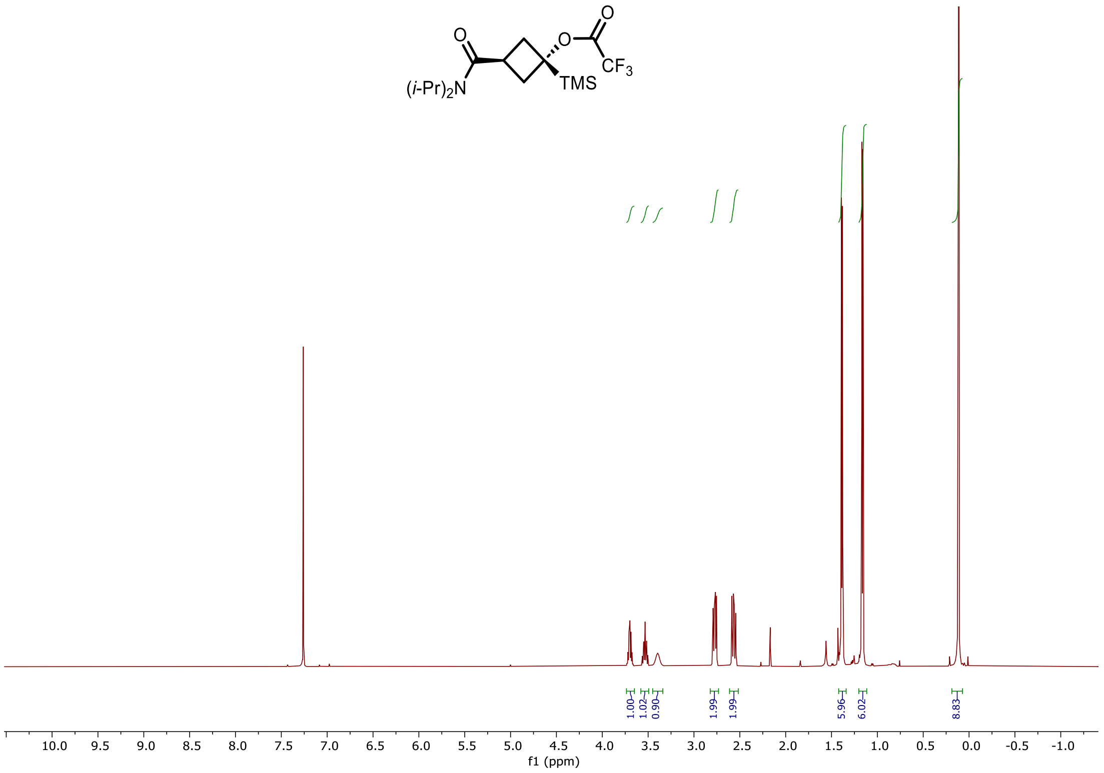

^{13}C NMR (151 MHz, Chloroform-*d*)

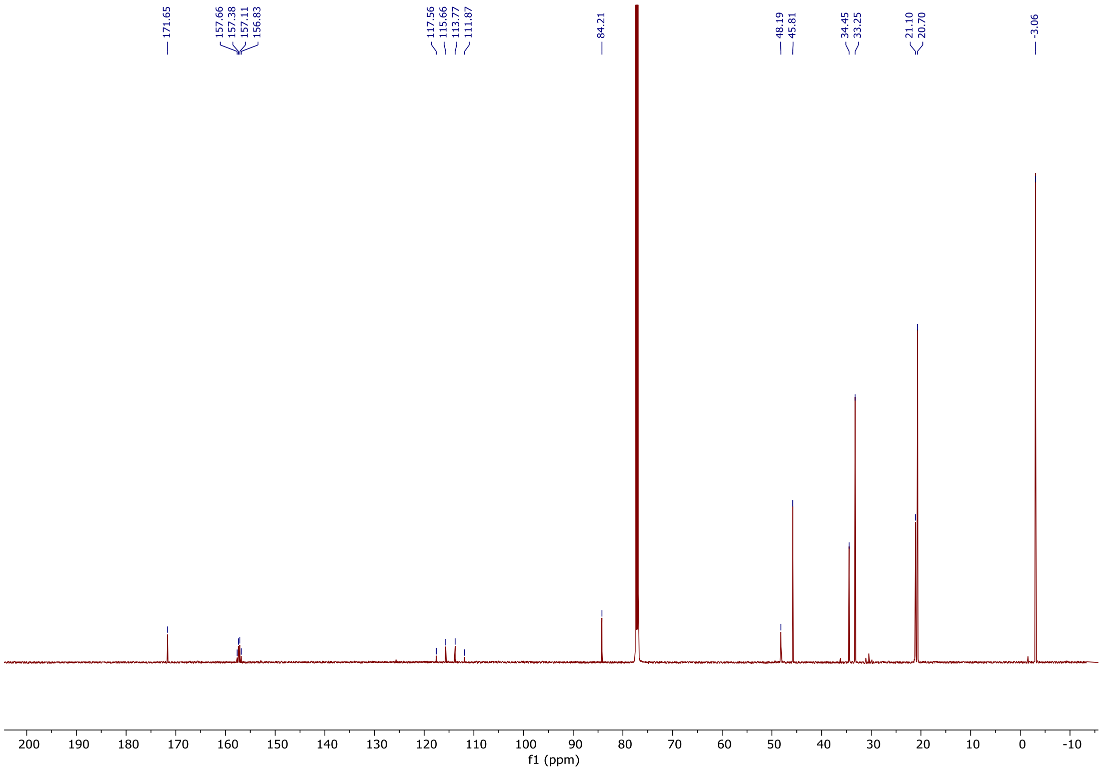

^{1}H COSY (600 MHz, Chloroform-*d*)

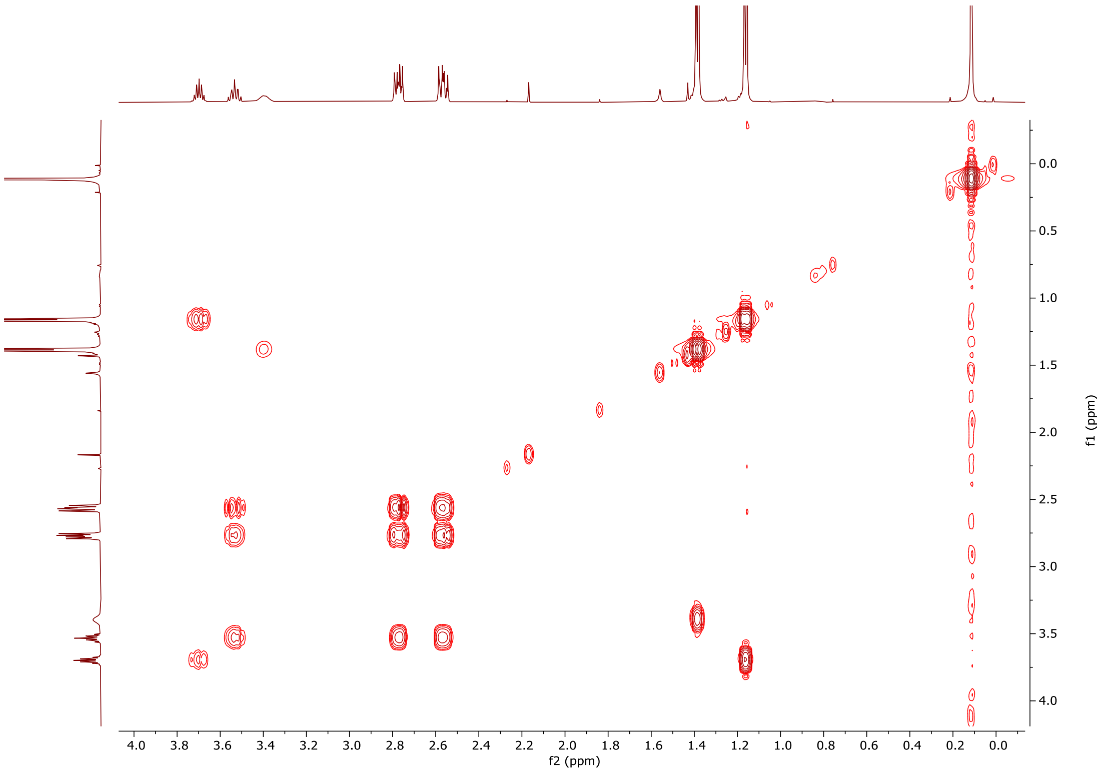

^{1}H/^{13}C HSQC (600/151 MHz, Chloroform-*d*)

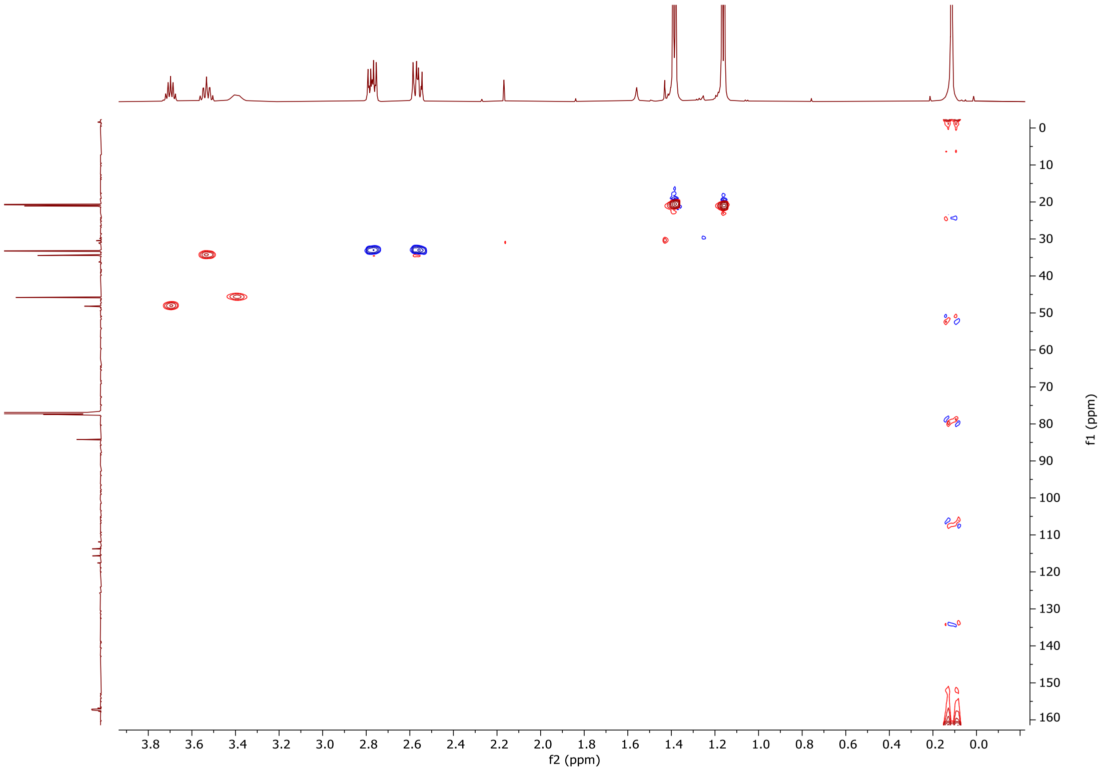

$^{1}H/^{13}C$ HMBC (600/151 MHz, Chloroform-*d*)

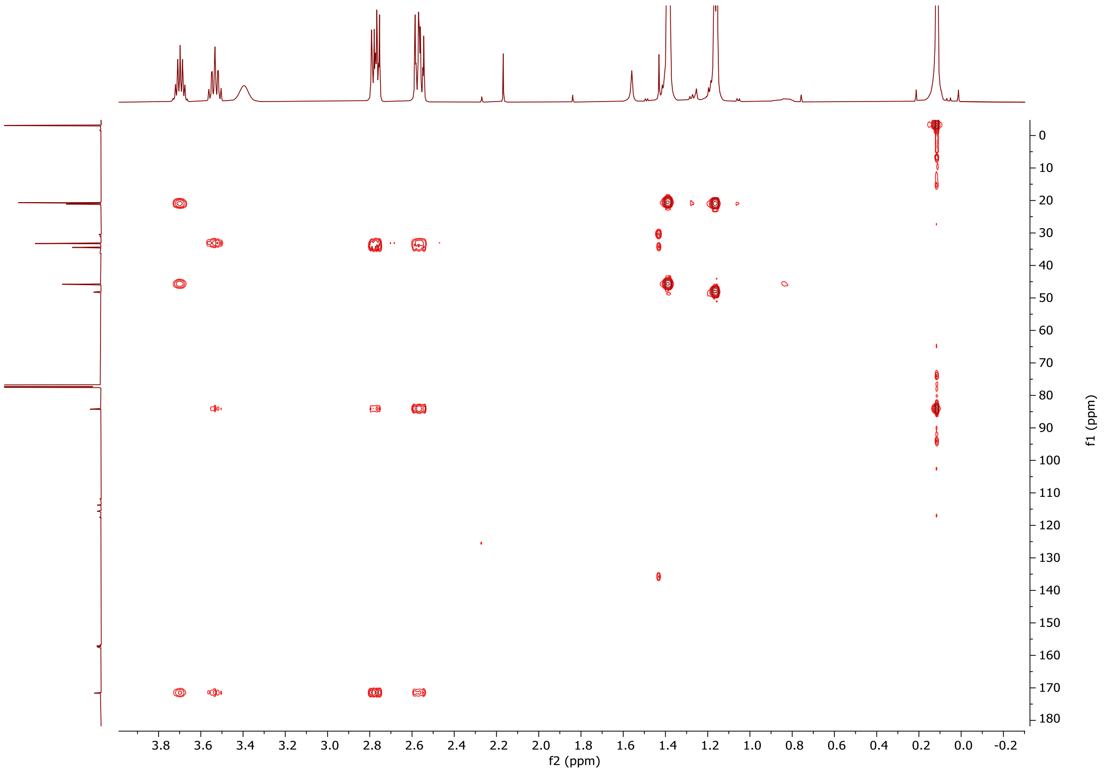

^{1}H NOSEY (600 MHz, Chloroform-*d*)

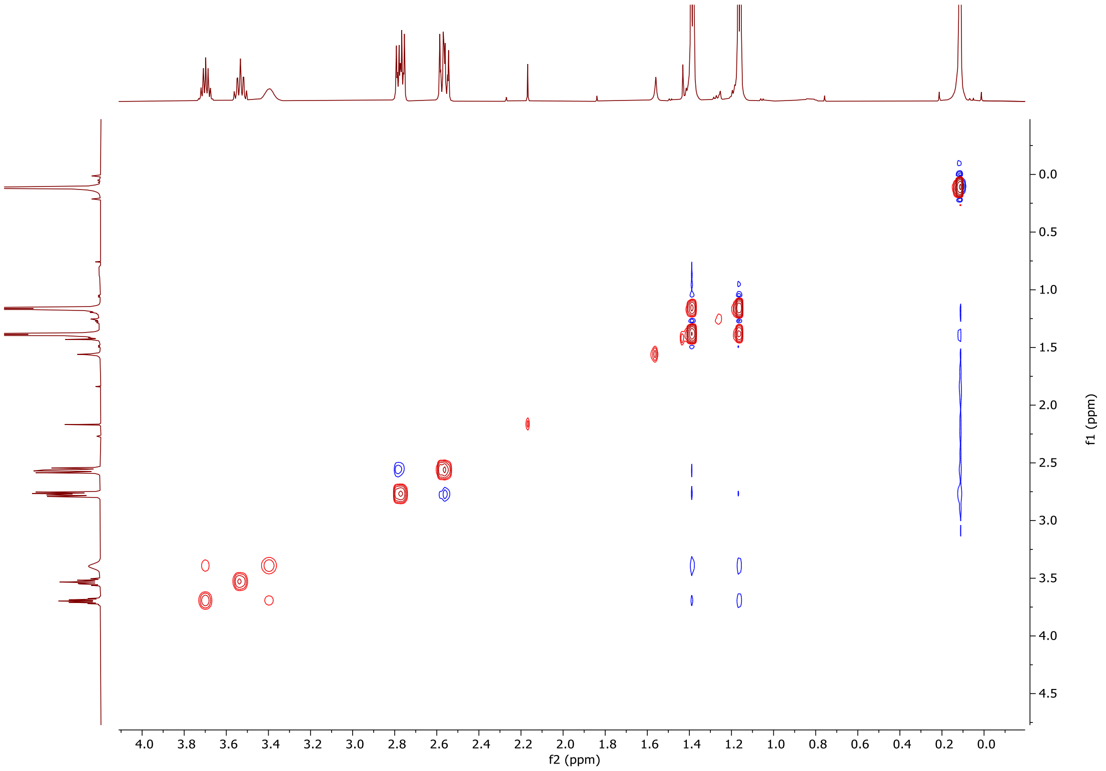

^{19}F NMR (565 MHz, Chloroform-*d*)

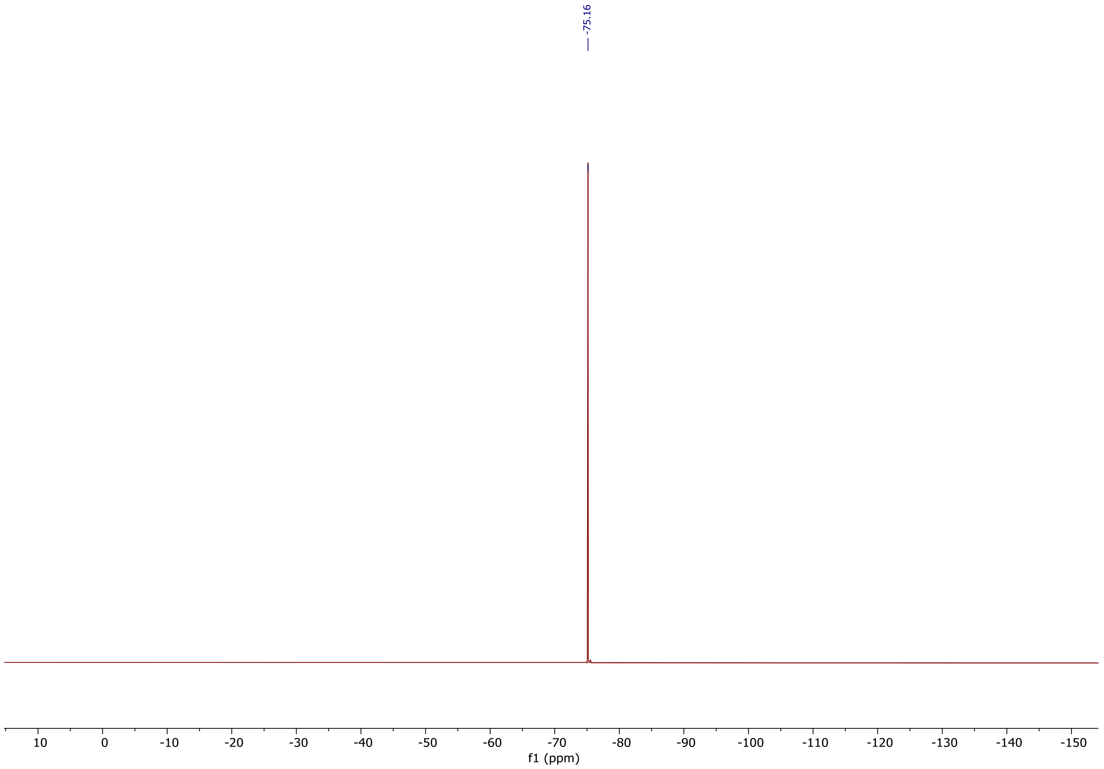

3-(diisopropylcarbamoyl)-1,2,4-trimethylcyclobutyl 2,2,2-trifluoroacetate, 4d

^{1}**H NMR** (600 MHz, Chloroform-*d*)

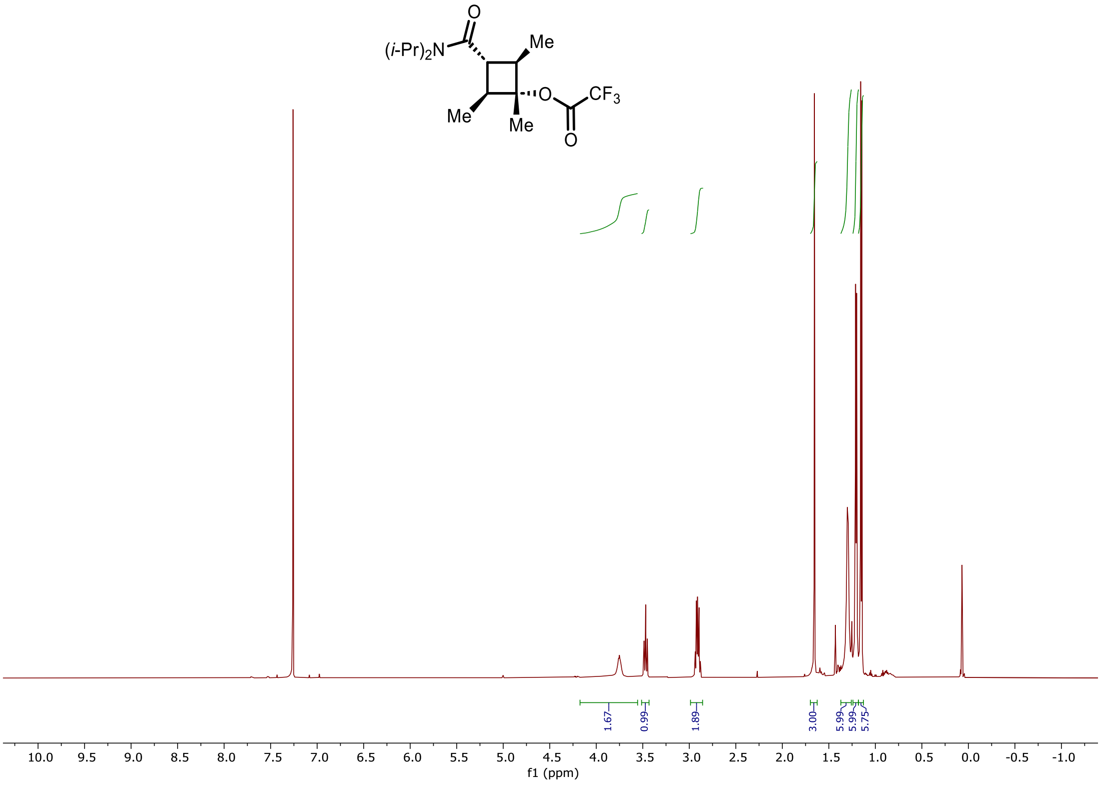

^{13}**C NMR** (151 MHz, Chloroform-*d*)

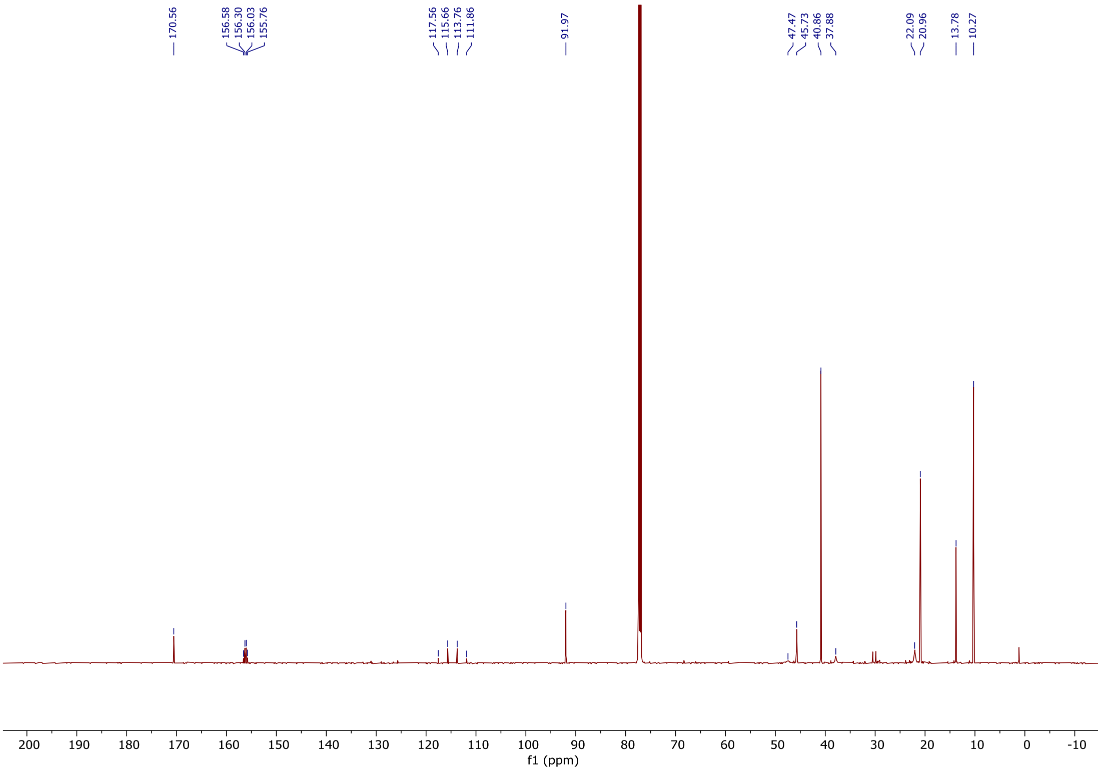

^{1}H COSY (600 MHz, Chloroform-*d*)

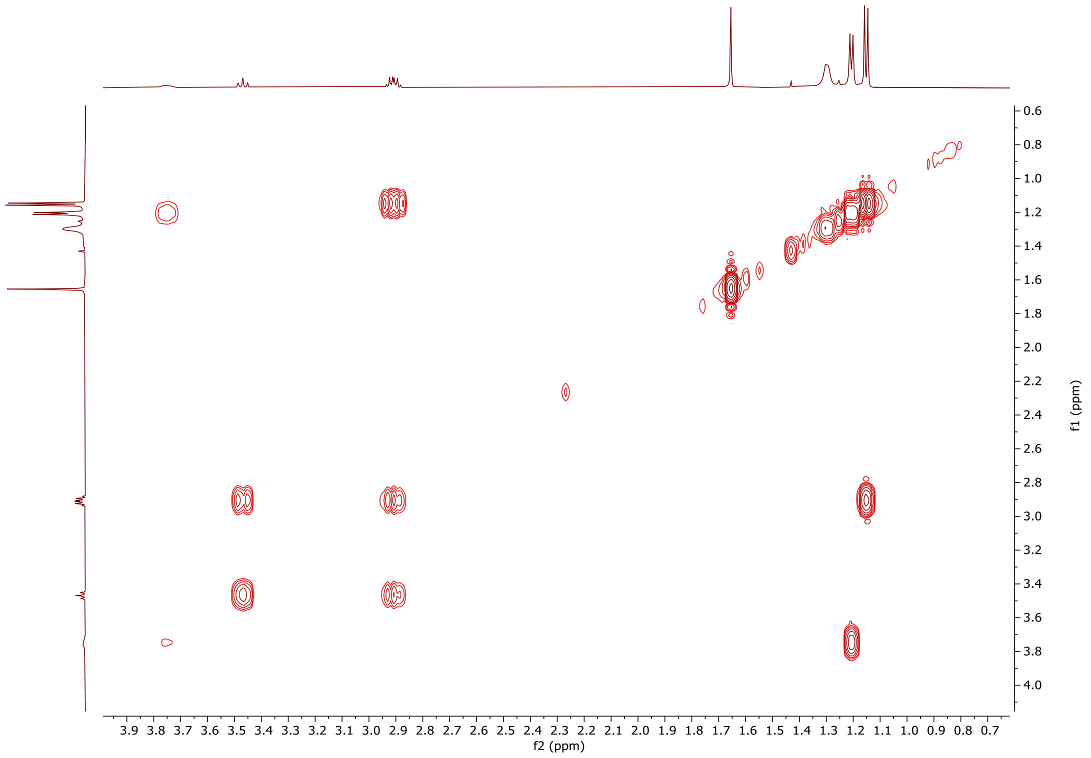

^{1}H/^{13}C HSQC (600/151 MHz, Chloroform-*d*)

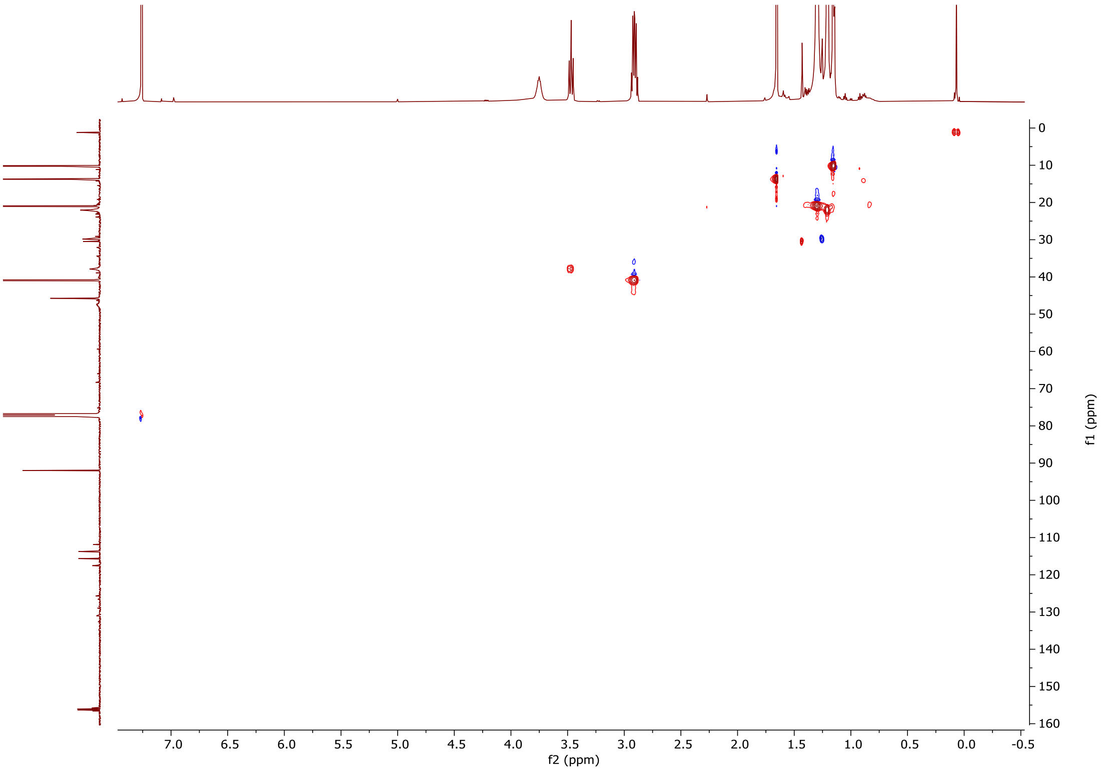

$^{1}H/^{13}C$ HMBC (600/151 MHz, Chloroform-*d*)

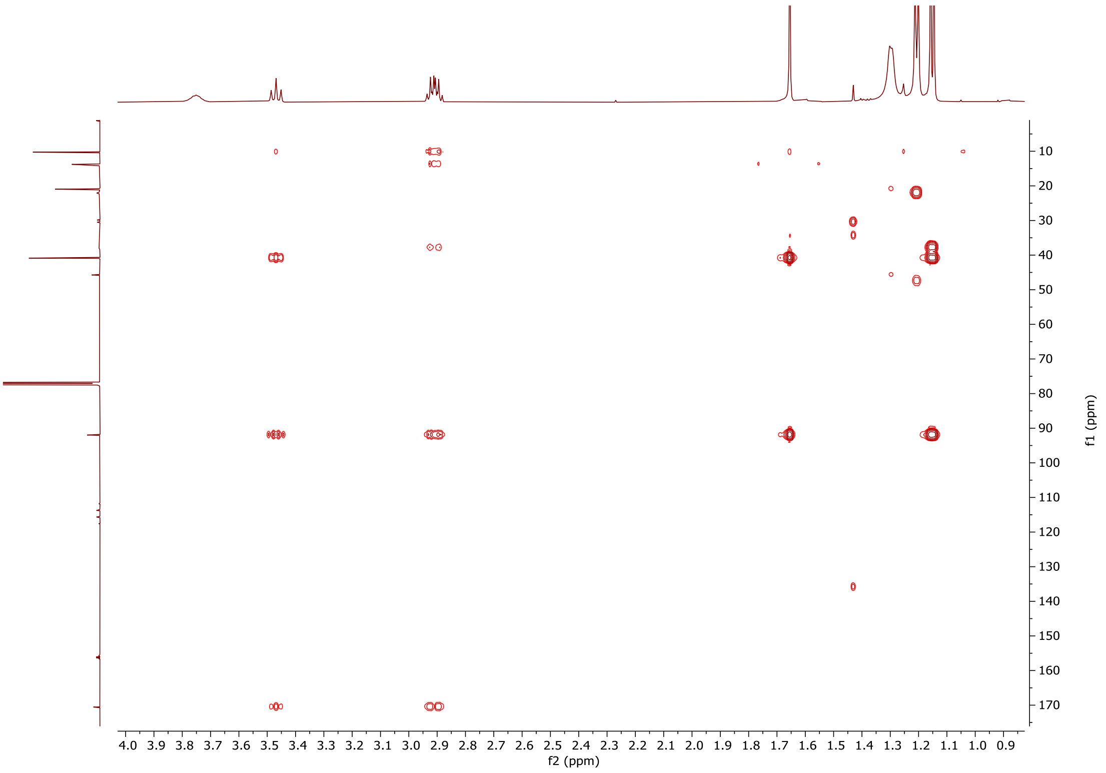

^{1}H-^{1}H NOSEY (600 MHz, Chloroform-*d*)

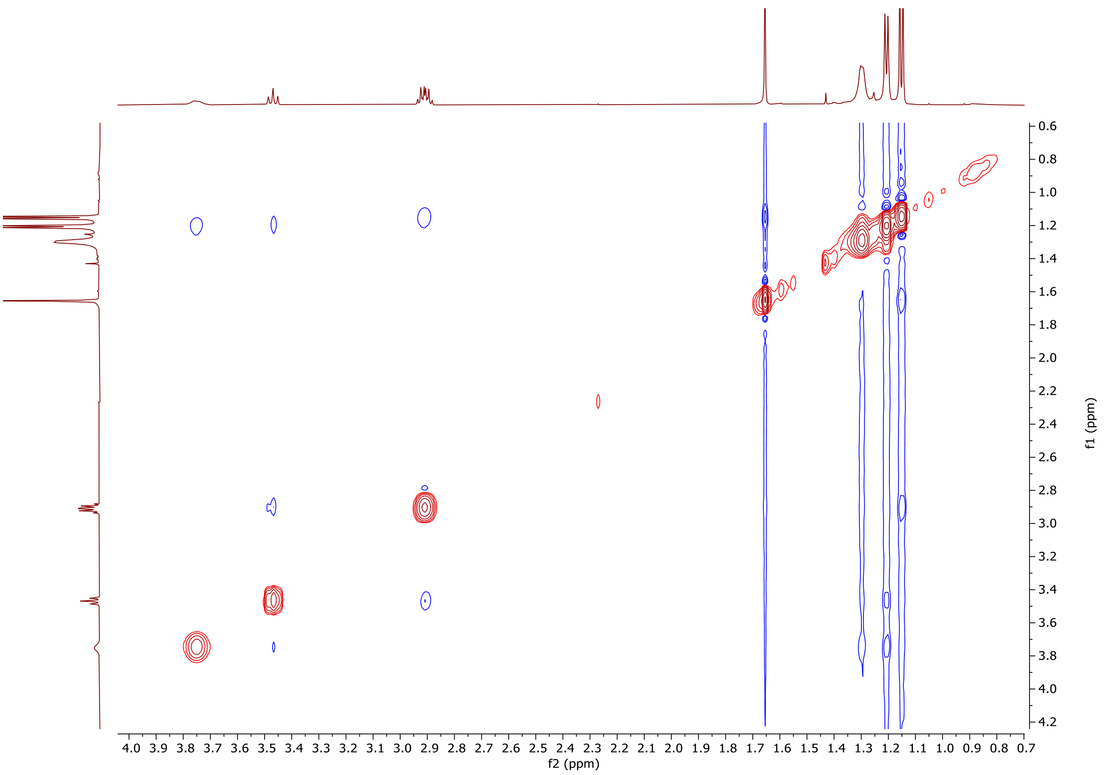

^{19}F NMR (565 MHz, Chloroform-*d*)

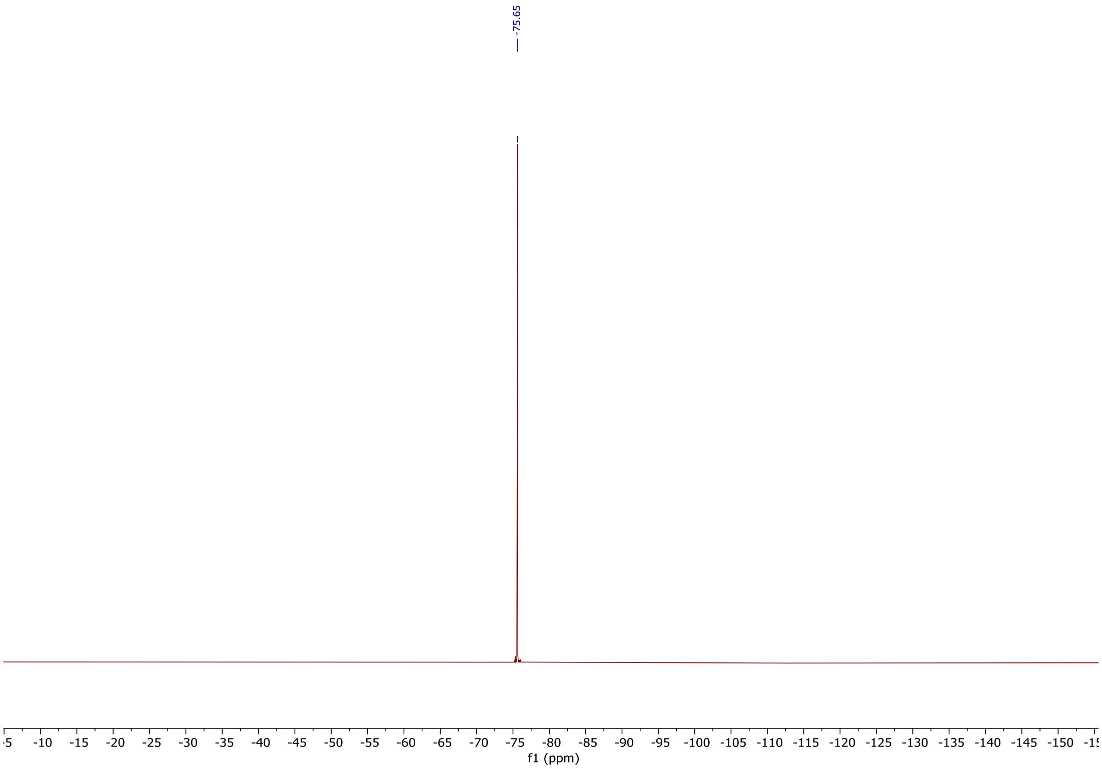

REFERENCES AND NOTES

1. H. C. Brown, *The Nonclassical Ion Problem*. (Springer, 2012).

2. G. A. Olah, 100 years of carbocations and their significance in chemistry. *J. Org. Chem.* **66**, 5943–5957 (2001).

3. H.-U. Siehl, in *Advances in Physical Organic Chemistry*, I. H. Williams, N. H. Williams, Eds. (Academic Press, 2018), vol. **52**, pp. 1–47.

4. P. D. Bartlett, *Nonclassical Ions*. (W. A. Benjamin, 1965).

5. J. D. Roberts, R. H. Mazur, Small-Ring Compounds. IV. Small-ring compounds. IV. Interconversion reactions of cyclobutyl, cyclopropylcarbinyl and allylcarbinyl derivatives. *J. Am. Chem. Soc.* **73**, 2509–2520 (1951).

6. J. D. Roberts, R. H. Mazur, The nature of the intermediate in carbonium ion-type interconversion reactions of cyclobutyl, cyclopropylcarbinyl and allylcarbinyl derivatives. *J. Am. Chem. Soc.* **73**, 3542–3543 (1951).

7. G. A. Olah, C. L. Jeuell, D. P. Kelly, R. D. Porter, Stable carbocations. CXIV. Structure of cyclopropylcarbinyl and cyclobutyl cations. *J. Am. Chem. Soc.* **94**, 146–156 (1972).

8. J. S. Staral, I. Yavari, J. D. Roberts, G. K. S Prakash, D. J. Donovan, G. A. Olah, Low-temperature carbon-13 nuclear magnetic resonance spectroscopic investigation of C4H7+. Evidence for an equilibrium involving the nonclassical bicyclobutonium ion and the bisected cyclopropylcarbinyl cation. *J. Am. Chem. Soc.* **100**, 8016–8018 (1978).

9. G. A. Olah, G. K. Surya Prakash, G. Rasul, Ab initio/GIAO-CCSD(T) study of structures, energies, and ^{13}C NMR chemical shifts of $C_4H_7^+$ and $C_5H_9^+$ ions: Relative stability and dynamic aspects of the cyclopropylcarbinyl vs bicyclobutonium ions. *J. Am. Chem. Soc.* **130**, 9168–9172 (2008).

10. S. Chakladar, Y. Wang, T. Clark, L. Cheng, S. Ko, D. J. Vocadlo, A. J. Bennet, A mechanism-based inactivator of glycoside hydrolases involving formation of a transient non-classical carbocation. *Nat. Commun.* **5**, 5590 (2014).

11. Y. J. Hong, J.-L. Giner, D. J. Tantillo, Bicyclobutonium ions in biosynthesis – Interconversion of cyclopropyl-containing sterols from orchids. *J. Am. Chem. Soc.* **137**, 2085–2088 (2015).

12. Y. J. Hong, D. J. Tantillo, The energetic viability of an unexpected skeletal rearrangement in cyclooctatin biosynthesis. *Org. Biomol. Chem.* **13**, 10273–10278 (2015).

13. D. P. Kranz, S. Chiha, A. Meier zu Greffen, J.-M. Neudörfl, H.-G. Schmalz, Synthesis of B-ring-modified steroids through BF3-promoted rearrangement/substitution of 6β-hydroxy-5,19-cyclosteroids. *Org. Lett.* **14**, 3692–3695 (2012).

14. L. A. Wessjohann, W. Brandt, T. Thiemann, Biosynthesis and metabolism of cyclopropane rings in natural compounds. *Chem. Rev.* **103**, 1625–1648 (2003).

15. V. Mascitti, E. J. Corey, Enantioselective synthesis of pentacycloanammoxic acid. *J. Am. Chem. Soc.* **128**, 3118–3119 (2006).

16. V. Lanke, I. Marek, Nucleophilic substitution at quaternary carbon stereocenters. *J. Am. Chem. Soc.* **142**, 5543–5548 (2020).

17. X. Chen, K. Patel, I. Marek, Stereoselective construction of tertiary homoallyl alcohols and ethers by nucleophilic substitution at quaternary carbon stereocenters. *Angew. Chem. Int. Ed.* **62**, e202212425 (2023).

18. X. Chen, K. Patel, I. Marek, Stereospecific nucleophilic substitution at quaternary carbon stereocenters of cyclopropyl carbinols. *Chem* **9**, 266–279 (2023).

19. R. Properzi, P. S. J. Kaib, M. Leutzsch, G. Pupo, R. Mitra, C. Kanta De, L. Song, P. R. Schreiner, B. List, Catalytic enantiocontrol over a non-classical carbocation. *Nat. Chem.* **12**, 1174–1179 (2020).

20. A. Bauer, G. Di Mauro, J. Li, N. Maulide, An α-cyclopropanation of carbonyl derivatives by oxidative umpolung. *Angew. Chem. Int. Ed.* **59**, 18208–18212 (2020).

21. J. Xie, G. Dong, Cyclopropylcarbinyl cation chemistry in synthetic method development and natural product synthesis: Cyclopropane formation and skeletal rearrangement. *Org. Chem. Front.*, **10**, 2346–2358 (2023).

22. S. P. Larmore, P. A. Champagne, Cyclopropylcarbinyl-to-homoallyl carbocation equilibria influence the stereospecificity in the nucleophilic substitution of cyclopropylcarbinols. *J. Org. Chem.* **88**, 6947–6954 (2023).

23. S. S. Goh, P. A. Champagne, S. Guduguntla, T. Kikuchi, M. Fujita, K. N. Houk, B. L. Feringa, Stereospecific ring contraction of bromocycloheptenes through dyotropic rearrangements via nonclassical carbocation–anion pairs. *J. Am. Chem. Soc.* **140**, 4986–4990 (2018).

24. H. Sato, B.-X. Li, T. Takagi, C. Wang, K. Miyamoto, M. Uchiyama, DFT study on the biosynthesis of verrucosane diterpenoids and mangicol sesterterpenoids: Involvement of secondary-carbocation-free reaction cascades. *JACS Au* **1**, 1231–1239 (2021).

25. C. B. Kelly, J. A. Milligan, L. J. Tilley, T. M. Sodano, Bicyclobutanes: From curiosities to versatile reagents and covalent warheads. *Chem. Sci.* **13**, 11721–11737 (2022).

26. M. A. A. Walczak, T. Krainz, P. Wipf, Ring-strain-enabled reaction discovery: New heterocycles from bicyclo[1.1.0]butanes. *Acc. Chem. Res.* **48**, 1149–1158 (2015).

27. M. Golfmann, J. C. L. Walker, Bicyclobutanes as unusual building blocks for complexity generation in organic synthesis. *Commun. Chem.* **6**, 9 (2023).

28. K. Tokunaga, M. Sato, K. Kuwata, C. Miura, H. Fuchida, N. Matsunaga, S. Koyanagi, S. Ohdo, N. Shindo, A. Ojida, Bicyclobutane carboxylic amide as a cysteine-directed strained electrophile for selective targeting of proteins. *J. Am. Chem. Soc.* **142**, 18522–18531 (2020).

29. B. D. Schwartz, A. P. Smyth, P. E. Nashar, M. G. Gardiner, L. R. Malins, Investigating bicyclobutane–triazolinedione cycloadditions as a tool for peptide modification. *Org. Lett.* **24**, 1268–1273 (2022).

30. J. M. Lopchuk, K. Fjelbye, Y. Kawamata, L. R. Malins, C.-M. Pan, R. Gianatassio, J. Wang, L. Prieto, J. Bradow, T. A. Brandt, M. R. Collins, J. Elleraas, J. Ewanicki, W. Farrell, O. O. Fadeyi, G. M. Gallego, J. J. Mousseau, R. Oliver, N. W. Sach, J. K. Smith, J. E. Spangler, H. Zhu, J. Zhu, P. S. Baran, Strain-release heteroatom functionalization: Development, scope, and stereospecificity. *J. Am. Chem. Soc.* **139**, 3209–3226 (2017).

31. P. Zhang, R. Zhuang, X. Wang, H. Liu, J. Li, X. Su, X. Chen, X. Zhang, Highly efficient and stable strain-release radioiodination for thiol chemoselective bioconjugation. *Bioconjug. Chem.* **29**, 467–472 (2018).

32. P. K. Mykhailiuk, Saturated bioisosteres of benzene: Where to go next? *Org. Biomol. Chem.* **17**, 2839–2849 (2019).

33. K. Livingstone, K. Siebold, S. Meyer, V. Martín-Heras, C. G. Daniliuc, R. Gilmour, Skeletal ring contractions via I(I)/I(III) catalysis: Stereoselective synthesis of cis-α,α-difluorocyclopropanes. *ACS Catal.* **12**, 14507–14516 (2022).

34. R. E. McNamee, A. L. Thompson, E. A. Anderson, Synthesis and applications of polysubstituted bicyclo[1.1.0]butanes. *J. Am. Chem. Soc.* **143**, 21246–21251 (2021).

35. R. E. McNamee, M. M. Haugland, J. Nugent, R. Chan, K. E. Christensen, E. A. Anderson, Synthesis of 1,3-disubstituted bicyclo[1.1.0]butanes via directed bridgehead functionalization. *Chem. Sci.* **12**, 7480–7485 (2021).

36. K. B. Wiberg, G. M. Lampman, R. P. Ciula, D. S. Connor, P. Schertler, J. Lavanish, Bicyclo[1.1.0]butane. *Tetrahedron* **21**, 2749–2769 (1965).

37. L. Guo, A. Noble, V. K. Aggarwal, α-Selective ring-opening reactions of bicyclo[1.1.0]butyl boronic ester with nucleophiles. *Angew. Chem. Int. Ed.* **60**, 212–216 (2021).

38. Y. J. Hong, D. J. Tantillo, C–H···π interactions as modulators of carbocation structure – Implications for terpene biosynthesis. *Chem. Sci.* **4**, 2512–2518 (2013).

39. T. Shono, A. Oku, R. Oda, Small ring compounds—XVI: Solvolysis of α(p-substitutedphenyl)cycloprophylcarbinyl p-nitrobenzoate. *Tetrahedron* **24**, 421–425 (1968).

40. E. N. Peters, H. C. Brown, Solvolysis of 1-aryl-1-cyclopropyl-1-ethyl p-nitrobenzoates. Evidence for major increases in electron supply by the cyclopropyl group with increasing electron demand at the cationic center. *J. Am. Chem. Soc.* **95**, 2397–2398 (1973).

41. K. Oyama, T. T. Tidwell, Cyclopropyl substituent effects on acid-catalyzed hydration of alkenes. Correlation by .sigma.+ parameters. *J. Am. Chem. Soc.* **98**, 947–951 (1976).

42. S. Hoz, M. Livneh, D. Cohen, Cyclobutane-bicyclobutane system. 11. Mechanism and stereochemistry of general acid-catalyzed additions to bicyclobutane. *J. Org. Chem.* **51**, 4537–4544 (1986).

43. G. A. Olah, V. P. Reddy, G. K. S. Prakash, Long-lived cyclopropylcarbinyl cations. *Chem. Rev.* **92**, 69–95 (1992).

44. R. I. Cooper, A. L. Thompson, D. J. Watkin, CRYSTALS enhancements: Dealing with hydrogen atoms in refinement. *J. Appl. Cryst.* **43**, 1100–1107 (2010).

45. P. Parois, R. I. Cooper, A. L. Thompson, Crystal structures of increasingly large molecules: Meeting the challenges with CRYSTALS software. *Chem. Cent. J.* **9**, 30 (2015).

46. E. Vedejs, J. Cabaj, M. J. Peterson, Wittig ethylidenation of ketones: Reagent control of *Z*/*E* selectivity. *J. Org. Chem.* **58**, 6509–6512 (1993).

47. C. Hansch, A. Leo, R. W. Taft, A survey of Hammett substituent constants and resonance and field parameters. *Chem. Rev.* **91**, 165–195 (1991).

48. F. Neese, The ORCA program system. *WIREs Comput. Mol. Sci.* **2**, 73–78 (2012).

49. F. Neese, Software update: The ORCA program system, version 4.0. *WIREs Comput. Mol. Sci.* **8**, e1327 (2017).

50. S. Grimme, Improved second-order Møller–Plesset perturbation theory by separate scaling of parallel- and antiparallel-spin pair correlation energies. *J. Chem. Phys.* **118**, 9095–9102 (2003).

51. R. F. Fink, Spin-component-scaled Møller–Plesset (SCS-MP) perturbation theory: A generalization of the MP approach with improved properties. *J. Chem. Phys.* **133**, 174113 (2010).

52. F. Neese, F. Wennmohs, A. Hansen, U. Becker, Efficient, approximate and parallel Hartree–Fock and hybrid DFT calculations. A 'chain-of-spheres' algorithm for the Hartree–Fock exchange. *Chem. Phys.* **356**, 98–109 (2009).

53. T. H. Dunning Jr., Gaussian basis sets for use in correlated molecular calculations. I. The atoms boron through neon and hydrogen. *J. Chem. Phys.* **90**, 1007–1023 (1989).

54. D. G. Liakos, M. Sparta, M. K. Kesharwani, J. M. Martin, F. Neese, Exploring the accuracy limits of local pair natural orbital coupled-cluster theory. *J. Chem. Theory Comput.* **11**, 1525–1539 (2015).

55. S. Grimme, Supramolecular binding thermodynamics by dispersion-corrected density functional theory. *Chem. A Eur. J.* **18**, 9955–9964 (2012).

56. T. Young, https://github.com/duartegroup/otherm. (2020).

57. I. Mayer, Charge, bond order and valence in the AB initio SCF theory. *Chem. Phys. Lett.* **97**, 270–274 (1983).

58. A. J. Bridgeman, G. Cavigliasso, L. R. Ireland, J. Rothery, The Mayer bond order as a tool in inorganic chemistry*J. Chem. Soc.*, **14**, 2095–2108 (2001).

59. D. H. Aue, Carbocations. *WIREs Comput. Mol. Sci.* **1**, 487–508 (2011).

60. I. Alkorta, J. L. M. Abboud, E. Quintanilla, J. Z. Dávalos, A theoretical study of (old and new) non-classical carbocations derived from cyclic saturated hydrocarbons. *J. Phys. Org. Chem.* **16**, 546–554 (2003).

61. F. Weigend, R. Ahlrichs, Balanced basis sets of split valence, triple zeta valence and quadruple zeta valence quality for H to Rn: Design and assessment of accuracy. *Phys. Chem. Chem. Phys.* **7**, 3297–3305 (2005).

62. H.-U. Siehl, M. Fuss, J. Gauss, The 1-(trimethylsilyl) bicyclobutonium Ion: NMR spectroscopy, isotope effects, and quantum chemical ab initio calculations of a new hypercoordinated carbocation. *J. Am. Chem. Soc.* **117**, 5983–5991 (1995).

63. F. L. Hirshfeld, Bonded-atom fragments for describing molecular charge densities. *Theor. Chim. Acta.* **44**, 129–138 (1977).
